# Multi‐method analysis for the three‐dimensional reconstruction of muscle fascicles from DiceCT datasets

**DOI:** 10.1002/ar.70088

**Published:** 2025-12-04

**Authors:** Aleksandra Ratkiewicz, Michael C. Granatosky, Cassidy E. Davis, Julia Molnar, Adam Hartstone‐Rose, Edwin Dickinson

**Affiliations:** ^1^ Department of Anatomy New York Institute of Technology College of Osteopathic Medicine New York USA; ^2^ Department of Ecology and Evolutionary Biology University of Tennessee Knoxville Knoxville Tennessee USA; ^3^ Duke Lemur Center Duke University Durham North Carolina USA; ^4^ Department of Anthropology The Pennsylvania State University State College Pennsylvania USA; ^5^ Department of Biological Sciences North Carolina State University Raleigh North Carolina USA; ^6^ Department of Anthropology and Archaeology University of Calgary Calgary Alberta Canada

**Keywords:** anatomy, DiceCT, fascicles, myology, tomography

## Abstract

Muscle architecture is a major determinant of muscle performance and, in mammalian lineages, has been correlated with both feeding ecology and locomotor behaviors. Over the past decade, contrast‐enhanced micro‐CT (DiceCT) has emerged as an alternative to traditional dissection‐based measurement. DiceCT allows the collection of myological data without damaging the specimen, and while preserving 3D relationships inside muscular tissues. However, manual segmentation of DiceCT datasets involves a major time investment and requires subjective judgments that can introduce bias. To address these shortcomings, several algorithmic approaches to tracing muscle fascicles have been described; however, these have not yet been rigorously tested in complex vertebrate muscle. Here, we present a standardized protocol for algorithmic fiber tracking using the commercial software extension *XFiber* within the *Amira* suite and compared its performance to manual segmentation, an open source algorithm (*GoodFibes*), and dissection values from the literature. Fascicle length and tortuosity (curvature) were measured in the jaw muscles of eight mammalian species spanning a wide range of cranial morphologies, diets, and body sizes. *XFiber* produced fascicle lengths that were generally similar to both gross dissection and manual segmentation, regardless of muscle identity or taxonomic group. All three digital methods tended to overestimate fascicle lengths relative to dissection, with *XFiber* and manual segmentation performing similarly (~15% overestimation). *GoodFibes* yielded substantially longer fascicle lengths, but its tortuosity values were closer to manual segmentation than those from *XFiber*. Given its major advantages in terms of time investment and inter‐operator reliability, we suggest this workflow may represent a promising method for large‐scale comparative studies.

## 1 INTRODUCTION

The architectural configuration of skeletal muscle is the primary determinant of muscle performance (Dickinson & Hartstone‐Rose, 2023). Muscle is a strongly hierarchical material, comprised internally of fascicles—bundles of intertwined muscle fibers that are themselves composed of serially arranged sarcomeres (Gans, 1982; Gans & Bock, 1965; Lieber & Ward, 2011). These sarcomeres represent the force‐generating unit of muscle, shortening via cross‐interactions between myosin and actin filaments to produce tension (Edman, 1966; Gordon et al., 1966; Lieber et al., 1994; ter Keurs et al., 1978). Since longer fascicles contain a greater number of sarcomeres that shorten simultaneously, increasing the length of a fascicle enhances both contractile velocity (i.e., closing distance per unit of time; Sacks & Roy, 1982) and excursion potential (i.e., total shortening distance; Gans, 1982; Gans & Gaunt, 1991; Lieber & Ward, 2011). In contrast, the magnitude of contractile force a muscle can generate is proportional to the total number of fascicles it contains. This force is calculated as the muscle's physiological cross‐sectional area (PCSA; Brand et al., 1986; Martin et al., 2020). From an architectural perspective, PCSA is maximized by incorporating a high number of shorter fascicles into any given volume. Thus, skeletal muscle is defined by a fundamental trade‐off in function between excursion/velocity and force (Gans, 1982; Lieber & Ward, 2011).

Over the past several decades, numerous efforts have been made to correlate muscle architectural variables (e.g., fascicle length, PCSA) with aspects of ecology, including body size, diet, habitat, and locomotor strategies (Cieri et al., 2022; Dickinson & Hartstone‐Rose, 2023; Grand, 1997; Hartstone‐Rose et al., 2022; Marchi et al., 2018). Such studies have revealed numerous persistent form–function relationships that span vertebrate lineages. For example, within the masticatory apparatus, longer fascicle lengths are observed in species that consume larger food items—an observation independently reported in felids (Hartstone‐Rose et al., 2012), caniforms including musteloids, ursids, and canids (Hartstone‐Rose et al., 2019, 2022; Penrose et al., 2020), bats (Curtis & Santana, 2018), strepsirrhines (Perry et al., 2011), and other primates (Dickinson et al., 2024; Dickinson, Atkinson, et al., 2020; Dickinson, Kolli, et al., 2020; Hartstone‐Rose et al., 2018). Similarly, within the forelimb, arboreality is linked to increased fascicle lengths in both Old World and New World monkeys relative to terrestrial taxa (Leischner et al., 2018), though no relationship is observed with either locomotor speed (Boettcher et al., 2019) or preferred substrate type (Gyambibi & Lemelin, 2013). From a force perspective, the PCSA of jaw adductor muscles is reportedly greater in both carnivorans and bats that consume mechanically challenging diets (Hartstone‐Rose et al., 2022; Herrel et al., 2008; Santana et al., 2010), and similarly increased in raptorial birds that kill prey with their beaks relative to those that utilize their talons (Dickinson et al., 2022; Sustaita & Hertel, 2010).

## 2 DIGITAL APPROACHES TO QUANTIFYING MUSCLE ARCHITECTURE

Despite analyzing drastically different anatomical systems across myriad taxa, all of the architectural studies outlined above share similar methodologies: gross dissection of muscle tissue away from its bony attachments, followed by *ex situ* measurements of muscle weights and fascicle lengths to determine PCSA (e.g., Curtis & Santana, 2018; Dickinson et al., 2021; Herrel et al., 2008). Though widespread, this workflow has several clear shortcomings. Firstly, by its essence, gross dissection is inherently destructive: a specimen must be skinned, and muscles irreversibly removed, to collect data on architectural properties (e.g., Dickinson et al., 2024; Gignac et al., 2016; Holliday et al., 2022; Taylor et al., 2024; Taylor & Vinyard, 2009). Secondly, by removing muscles from their anatomical context prior to measurement, spatial data—particularly that relates to muscle fascicle orientation and curvature/tortuosity—are not preserved, resulting in the loss of potential morphological data that could be collected and compared between species (Dickinson et al., 2024; Gignac et al., 2016; Hartstone‐Rose et al., 2020; Sullivan et al., 2019; Taylor et al., 2024).

In recent years, however, adoption of chemical staining techniques have been coupled with advances in tomographic visualization resolution, resulting in the emergence of a novel toolkit for collecting data on muscle architecture (Dickinson et al., 2024; Gignac et al., 2016; Sullivan et al., 2019). Diffusible iodine‐based contrast‐enhanced computed tomography (DiceCT) employs a staining agent that binds with the glycogen found in muscle fascicles to increase their radiopacity (Gignac et al., 2016). While numerous staining agents have been proposed and utilized within recent years—including Lugol's iodine, phosphotungstic acid (PTA), phosphomolybdic acid (PMA), and osmium tetroxide—iodine‐based solutions have emerged as the most popular tool within this workflow (Degenhardt et al., 2010; Gignac et al., 2016; Jeffery et al., 2011; Metscher, 2009; Pauwels et al., 2013). Specifically, as iodine binds directly to muscle fascicles but not the surrounding collagenous perimysium (Descamps et al., 2014; Gignac et al., 2016; Li et al., 2015; Metscher, 2009), individual fascicles can be discriminated within CT datasets scanned at high enough resolution (Dickinson et al., 2018, 2019, 2024). As a less destructive and substantially reversible technique, DiceCT enables the examination of specimens that would be unfit for dissection under normal circumstances (Early et al., 2020; Lanzetti & Ekdale, 2021; Morhardt & Witmer, 2016), owing either to their fragility or their irreplaceability. Another major benefit of this digital approach is the ability for data sharing, reuse and archiving: while a physical specimen is typically dissected in its entirety by one individual at a given point in time with data collected in a limited fashion (e.g., specific anatomical measurements and photographs), digital datasets can be shared between researchers and reanalyzed years later with no degradation in sample quality (Gignac et al., 2016). Additionally, DiceCT techniques circumvent practical barriers surrounding deep and inaccessible anatomical systems (e.g., the hyolingual musculature; Li & Clarke, 2015; Orsbon et al., 2018, 2020) or small and delicate muscles (e.g., the mimetic or extraocular muscles) that are difficult to study via traditional gross dissection (Dickinson, Atkinson, et al., 2020; Evans & Elder, 2024; Li et al., 2021; Yamashita & Tsuihiji, 2022). Perhaps most importantly, the DiceCT toolkit preserves the fascicular architecture of muscles in situ, maintaining three‐dimensional relationships that would be lost via conventional dissection methods (Dickinson et al., 2018, 2019; Dickinson et al., 2024; Dickinson, Atkinson, et al., 2020).

Fascicles from DiceCT datasets can be individually segmented, with some studies (Dickinson et al., 2018, 2019; Dickinson, Atkinson, et al., 2020) specifically demonstrating good agreement between fascicle lengths derived from segmentation and those collected from gross dissection. However, attempts to automate the process of fascicle identification and segmentation promise to facilitate the analysis of larger numbers of data points in significantly less time (Arbour, 2023; Dickinson et al., 2018; Holliday et al., 2022; Kupczik et al., 2015; Sullivan et al., 2019); though it should be noted that, while factoring in time for staining and scanning, this process is still notably longer than gross dissection. Furthermore, these automated approaches also significantly reduce the potential for human bias in fascicle selection and enhance replicability (Dickinson & Hartstone‐Rose, 2023). The earliest attempt to automatically extract fascicles from DiceCT datasets was performed on a single canine masseter (Kupczik et al., 2015) using a custom‐written software package (imagexd; http://starkrats.de) and employed a twofold approach: after manual segmentation to isolate the muscle body, a textural orientation algorithm was first employed to recognize and trace continuous structures within the muscle (following the underlying premise that lower variation in density would be expected along a continuing fascicle than between a fascicle and its surrounding connective tissue). From the resultant texture map, a vector field was then used to compute 3D streamlines representing the distribution of individual fascicles throughout the muscle volume (Kupczik et al., 2015). This workflow was subsequently applied to three masticatory muscles within a single crab‐eating macaque, wherein estimates of fascicle length and PCSA generated by the algorithmic technique were compared to gross dissection data collected from the contralateral muscles (Dickinson et al., 2018). This comparison demonstrated good concordance for simple muscles (e.g., superficial and deep masseter), but the methodology performed poorly when applied to more three‐dimensionally complex and heterogeneous muscles such as the temporalis (Dickinson et al., 2018). This technique was also shown to be sensitive to the number of seed points used for streamline generation, wherein increasing the number of points reduced the average fascicle length by capturing shorter structures within the muscle volume.

The introduction of *XFiber*, an extension to the Amira/Avizo (http://www.amira.com; Thermo Fischer Scientific, Waltham, MA) software cluster (Stalling et al., 2005), facilitated a secondary pipeline for the extraction of fascicular data from DiceCT datasets (Blanc & Westenberger, 2017). *XFiber* applies a probabilistic, template‐based algorithm to the underlying grayscale map that constitutes CT slices, analyzing variation between adjacent voxels to plot the trajectory of continuous structures which are then traced to produce maps of internal fascicles (Blanc & Westenberger, 2017; Sullivan et al., 2019). This technique was first applied to pectoral muscles within a starling (Sullivan et al., 2019) and subsequently to map masticatory muscle architecture within an alligator (Holliday et al., 2022). These projects yielded in situ fascicle orientation maps that allowed quantitative assessment of intra‐ and intermuscular variation in fascicle configuration comparable to the vector fields generated from manual fascicle segmentation of DiceCT data (Dickinson et al., 2019; Dickinson et al., 2024; Dickinson, Atkinson, et al., 2020) but independent of human bias in fascicle selection or manual error associated with the segmentation process. However, the comparability of fascicle lengths or PCSA generated by *XFiber* to manual segmentation of fascicles was not assessed, and comparisons to gross dissection were limited to a single starling muscle (*M. supracoracoideus*)—yielding a 19% underestimation of fascicle length and a 48% overestimation of PCSA (Sullivan et al., 2019). Thus, the biological efficacy of this workflow remains largely unvalidated. Moreover, as the process of fascicle generation in *XFiber* relies upon numerous user‐controlled variables (including a priori establishment of a reference cylinder length, direction coefficient, and minimum continuation distance, among others), it has been necessary to individually tailor input parameters for each muscle until the algorithm produces qualitatively plausible models that aligned with initial expectations of fascicle orientation (Sullivan et al., 2019). This shortcoming both reduces replicability and relies upon a pre‐existing understanding of fascicle architecture that undercuts some of the theoretical value of this technique (e.g., to understand muscle architecture without a priori assumptions).

As an alternative tool for the algorithmic reconstruction of muscle architecture, Arbour (2023) introduced *GoodFibes*, an R (R Core Team, 2021) package using grayscale value analysis to automatically identify and track muscle fascicles throughout a given muscle body. Following the input of CT images converted into a .png stack, *GoodFibes* places a user‐determined number of seed points into chosen image planes within the muscle, each of which tracks the path of a single muscle fascicle (Arbour, 2023). After the initial generation of these fascicle paths, additional functions allow for the *post‐hoc* removal of fascicles with outlying lengths, as well as permitting the separation or merging of fascicles that were mistakenly combined or terminated prematurely. This data can then be used for the quantification of muscle architectural variables using “fiber.lengths,” “fiber.curve,” and “fiber.angle” functions to calculate fascicular length, curvature (i.e., tortuosity), and orientation relative to a specific axis in the scan, respectively. Additionally, 3D visualizations of these results can be exported as surface files, allowing fascicles to be reconciled with their corresponding bony elements. To date, *GoodFibes* fascicle reconstruction has been empirically tested using the mandibular muscle of the pharaoh ant (*Monomorium pharaonis*) as well as several masticatory muscles of an Egyptian Fruit Bat (*Rousettus aegyptiacus*). Comparisons between fascicle length data collected via gross dissection and those values generated by *GoodFibes* showed generally good agreement in both species, though neither tortuosity nor angle data generated by this method have been reported (Arbour, 2023).

A valuable comparison of several of these techniques was recently published by Katzke et al. (2022), wherein a single muscle (*M. craniomandibularis internus*) of a pharaoh ant (*Monomorium pharaonic*) was comparatively assessed via gross dissection, *imagexd*, and *XFiber*. They reported extremely strong congruence between methods for both fascicle lengths (0.114 ± 0.014 via gross dissection, 0.111 ± 0.026 via imagexd, and 0.106 ± 0.021 via *XFiber*) and PCSA (0.067 via gross dissection, 0.071 via imagexd, and 0.074 via *XFiber*) (Katzke et al., 2022). However, this study used a highly simplified anatomical system (the ant masticatory apparatus), wherein the number of fascicles was extremely low, and boundary separation between adjacent fascicles was clear, allowing the easy discrimination of every fascicle: an idealized state that does not represent typical DiceCT datasets of complex vertebrates—for example, see images from Dickinson et al. (2019, 2024), Gignac et al. (2016), Holliday et al. (2022), Santana et al. (2010), and Sullivan et al. (2019). Thus, broader comparative analyses across a diverse range of muscles, taxa, and body sizes are necessary to truly test the compatibility of these digital fascicle tracing methods and their degree of convergence with traditional architectural analyses.

## 3 AIMS OF THE STUDY

This study addresses two distinct but interrelated aims: First, we conduct a comprehensive sensitivity analysis of fascicle generation using *XFiber*, assessing how individually modifying each input parameter affects the architectural properties of the resultant fascicle maps. From this analysis, we develop a standardized protocol for fascicle generation, outlining a workflow where all input properties are calculated directly from raw DiceCT datasets and their associated metadata. This approach enhances compatibility between datasets, promotes inter‐user replicability, and automates post‐hoc exclusion criteria based on a constant z‐score applied to fascicle orientation, removing biologically implausible outliers.

Second, we perform a broad comparative analysis of methods for calculating muscle fascicle architecture across eight diverse specimens drawn from different mammalian lineages, which vary drastically in size and shape. We compare fascicles generated by the proposed protocol against another algorithmic workflow (*GoodFibes*; Arbour, 2023), manually segmented fascicles, and those collected via a gross dissection protocol. Through this comparison, we evaluate the efficacy of our proposed workflow and provide a detailed perspective on the methodological comparability of current methods for determining muscle fascicle architecture across a morphologically diverse sample.

## 4 MATERIALS AND METHODS

### 4.1 Sample composition and preparation

We selected adult cranial specimens representing eight mammalian species (five carnivorans: *Mustela nivalis*, *Martes americana*, *Mephitis mephitis*, *Procyon lotor*, and *Lontra canadensis*; and three primates: *Otolemur garnettii*, *Eulemur coronatus*, and *Daubentonia madagascariensis*), which span a wide range of cranial morphologies, dietary attributions, and body sizes (~50 g to ~10 kg; Nowak, 1999; Wilson et al., 2009). We chose these specific taxa because gross dissection data have already been published for each species (Dickinson et al., 2024; Hartstone‐Rose et al., 2022), and they occupy well‐described ecological niches that allow for functional interpretation of anatomical observations. All specimens were frozen upon death and stored as such until the onset of the study. All were free of craniodental pathologies and displayed good muscular health with no visible atrophy or wastage of muscle tissue. Primate tissues were sourced from the Duke Lemur Center (Durham, NC), while carnivoran specimens were acquired as by‐products of the taxidermy industry. No animals were euthanized for this project, and cadaveric specimens are considered exempt by the North Carolina State University IACUC, where the specimens were prepared.

We removed overlying superficial tissues (i.e., skin) from each specimen, before fixing each specimen via immersion in 10% neutral buffered formalin (NBF) for 1–2 weeks (depending on specimen size). Fixation was judged based on manual palpation of tissues and the experience of the authors with developing appropriate fixation protocols of like‐sized specimens. We then collected μCT scans using either a North Star Imaging (NSI) μCT system housed at the University of Texas or a Nikon XTH‐225‐ST μCT system housed at Duke University's Shared Materials Instrumentation Facility. We reconstructed the scans into 16‐bit image stacks using isometric voxel sizes (0.019–0.071 mm). After scanning, we transferred each specimen to a 10% w/v sucrose solution for 48 h to expedite the uptake of iodine and reduce the risk of deformation during the staining process (Gignac et al., 2016; Morhardt & Witmer, 2016). Following the sucrose prewash, we moved the specimens into a low‐concentration (1.75%–2.5%) Lugol's iodine (I_2_KI) solution for several weeks until the staining agent fully penetrated all tissues of interest. We used a low‐concentration iodine solution to minimize iodine‐induced changes in muscle volume (Gignac et al., 2016; Vickerton et al., 2013). It should be noted that formalin fixation may lead to some tissue shrinkage within the specimen (Léonard et al., 2004), making it difficult to directly compare muscle volumes between unfixed and formalin‐fixed tissues without a correction factor; though the impact of this shrinkage on fascicle lengths is comparatively lower (Dickinson, Atkinson, et al., 2020; Dickinson, Kolli, et al., 2020; Leonard et al., 2022). After achieving full iodine penetration, we lightly destained each specimen in distilled water for a few days before the final scan to prevent overstaining of superficial layers and ensure an even contrast gradient between deep and superficial tissues. Finally, we collected and reconstructed a second series of μCT scans using the same protocol.

In order to conduct the digital dissection phase of the process on muscles with as even saturation density as possible throughout the entire depth of the specimen, the largest specimens (*P. lotor and L. canadensis*) required two postscans to be collected: the first postscan was conducted to visualize the superficial muscles, following which the specimens were returned to their staining solution for a further month before a second scan was conducted to further visualize the deeper muscle fascicles. Fascicles from these two postscans were then digitally aligned using common landmark registration methods, as described in Dickinson and Hartstone‐Rose (2023).

We analyzed both jaw adductors and abductors. In the three primates, we separated the muscles into 10 distinct divisions: the masseter and temporalis complexes (each with superficial, deep, and zygomatic portions), the medial and lateral pterygoids, and the anterior and posterior digastrics. Since carnivorans possess a simpler temporalis and a fused digastric, we separated their muscles into eight divisions, omitting the zygomatic temporalis and analyzing the digastrics as a single unit.

### 4.2 Methods for collecting fascicular architecture

We followed a previously established protocol for manually segmenting muscle fascicles (Dickinson et al., 2019; Dickinson, Atkinson, et al., 2020). Image stacks were loaded into Amira 6.5 (http://www.amira.com; Thermo Fischer Scientific, Waltham, MA) and segmented using the wand tool when fascicle boundaries were easily discernible or the brush tool when boundaries were unclear and needed manual definition. The number of representative fascicles segmented per muscle varied according to muscle size, ranging from 6 to 50 fascicles per muscle.

For the analysis of muscle fascicles using *GoodFibes*, we followed the outline in Arbour (2023). Before uploading images into R (R Core Team, 2021), we first created sub‐stacks from our original CT dataset to isolate each masticatory muscle division. Since the “good.fibes” function can only trace fascicles in a single image plane, we manually evaluated and reoriented these muscle stacks using Dragonfly to best align the fascicles with the *z*‐axis. After reorientation, we transformed all muscle stacks into .png image stacks using ImageJ (Schneider et al., 2012) before use. For *M. nivalis*, we also adjusted the contrast in ImageJ to improve image quality for analysis by first altering global contrast levels and subsequently making local adjustments to the gray‐value histogram within the muscle itself, in order to maximize the contrast in intensity between individual fascicles and their adjacent connective tissues.

While following the published guidelines from Arbour (2023), we attempted to compare two workflows within the software. First, we ran a standardized set of parameters in which we manually selected five representative image slices for seed point placement, and applied a muscle‐specific intensity threshold to remove other tissues from each slice and leave behind only fascicles. We then used the “good.fibes” function to track muscle fascicles through the images and create a list of three‐dimensional coordinates for each fascicle path. This required input values for both threshold and cutoff values. Threshold values were varied between slices in each muscle to ensure the best local contrast, allowing fascicles to separate as cleanly as possible. For example, for the superficial masseter of *E. coronatus*, we used five different slices distributed evenly throughout the muscle, with threshold values ranging from 0.79 and 0.82; while for the digastric of *M. americana*, threshold values ranged from 0.51 to 0.59. In all cases, the cutoff value for each slice was set at a value 0.1 lower than the threshold value (e.g., for a threshold value of 0.65, a cutoff of 0.55 would be applied).

After this initial fascicle path generation, we ran several cleaning functions to identify and correct any errors in fiber tracing. The “quality.check,” “fuse.fiber,” and “check.overlap” functions excluded low‐quality fascicles (those with high grayscale variability along the fascicle's length compared to the fiber's length), merged incomplete fibers, and deleted fibers produced by seed points dropped on the same fascicle. For the “quality.check” function, we specified only the resolution of the scan and the degrees of freedom (df = 2 in all cases) and chose not to provide a manual cut‐off for minimal fascicle lengths. For the “fuse.fibers” function, we utilized a consistent input of (8,0.25,1) for all muscles in all samples—reflecting a minimum distance of 8 voxels between adjacent fascicles, chosen following recommendations in Arbour (2023) as the cross‐sectional diameter of our fascicles averaged ~10 pixels. The additional values represent the minimum increase in fascicle length (in this case, 25%) considered necessary for this process to be applied, and the degrees of freedom (set to the default value of 1 for all fascicles herein). Finally, for the “check.overlap” function, we used the same minimum voxel distance (8 voxels) as the previous function, and a df value of 2. Once we determined the final fiber paths, we ran “fiber.length” to capture the lengths of each muscle fascicle and “fiber.curve” to determine tortuosity. Finally, we exported the plotted fiber paths from R (R Core Team, 2021) using the “muscle.plot.stl” function to generate 3D images of the fascicles for visual examination (Arbour, 2023).

We then compared this data to a locally optimized series of parameters, in which input parameters were altered for each of the five individual seed‐point slices to maximize the best local resolution. A more aggressive threshold and cutoff filter was also used, which lowered the number of fascicles left within each image to only the clearest fascicles. Additionally, the number of seedpoints were varied between slices to optimize the output fascicles.

For automated segmentation using *XFiber*, we used the same sub‐stacks as previously described, exporting each individual muscle division into independent files. We then isolated the muscle itself within *Amira* and grew the entire muscle volume five times (i.e., by five pixels in each direction) to create an artificial shell that allows *XFiber* to follow the entire length of the muscle fascicle without prematurely stopping at the muscle volume boundaries. This shell was later removed prior to fascicular analysis by applying a mask of the original muscle volume over the final correlation field. We tested muscle volume growth at three times, five times, and seven times, finding that a growth of three times was sufficient for smaller muscles like the lateral pterygoid and the digastric muscles of primates, five times was ideal for most cranial muscles (e.g., medial pterygoid, zygomatic temporalis of primates, superficial masseter, deep masseter, and zygomaticomandibularis), and seven times was reserved for larger, complex multipennate muscles like the deep and superficial temporalis. Smaller muscles—particularly the primate anterior and posterior digastric—did not perform well in *XFiber* when grown excessively, so we limited volume growth to the necessary amount for complete fascicle tracking while avoiding the inclusion of external artifacts. After growing each independent muscle volume adequately, both the grown muscle volumes and the original mask were cropped to the same dimensions to reduce computational time and allow for faster alignment.

For each muscle, we began by applying the cylinder correlation function within *XFiber*. Through use of the visual cylinder tool, we positioned a cylinder along a single representative fascicle, using its length as an input for the cylinder correlation. The radius of the cylinder/fascicle was then determined by adjusting the size of the visual cylinder until it just filled the cross‐sectional outline of the fascicle in the CT scan. As fascicle lengths varied widely across our sample, the absolute inputs for cylinder lengths also varied widely between muscles and species; however, as fascicle and fiber diameters are generally more conservative (Leonard et al., 2022), the cylinder radii exhibited much less variation.

In some instances—for example, when muscles exhibited high heterogeneity in fascicle length across a single volume—it was necessary to utilize multiple reference cylinders for a single muscle. In these instances, we utilized the “Multiple Cylinder Correlation” function, an optional extension to *XFiber* that allows multiple cylinders to be designated as representative fascicles following a protocol outlined by Maksimcuka et al. (2017). In our case, this extension was used for the superficial and deep temporalis, wherein muscle fascicles varied substantially in length between different regions. For such muscles, we instead placed three distinct reference cylinders on fascicles from different portions of the muscle, capturing as much variation as possible.

Upon completion of the Cylinder Correlation function, *XFiber* populated a correlation field and an orientation field. We processed the correlation field through the same algorithmic process as before [a*(*b* > 0)], using the original muscle volume file as a mask over the artificially grown muscle volume. We then used the masked result file in combination with the orientation field to extract individual fascicles using the Trace Correlation Lines function, the most significant post‐production step, allowing the user to alter many functions, which we aimed to standardize as detailed below (see Section 5). Within this function, it was necessary to specify several input variables, which were determined through extensive sensitivity testing across different muscles and scans (see Section 5).

After processing the Trace Correlation Lines function, resultant fascicles were visualized these lines using the Spatial Graph View function, allowing us to gauge if the architecture was representative and adjust the minimum distance as needed for fascicle density. We then analyzed fascicles on the basis of their orientation, utilizing the embedded variable “Orientation Theta” to guide us on the level of homogeneity of fascicle distribution. Due to the fully automated nature of the process, a handful of artificial fascicles were created in almost all cases; however, as these false fascicles differed in spatial orientation from the prevailing fascicle architecture within the muscle, we were able to exclude the majority of these errors by implementing a manual cutoff filter: specifically, a *z*‐score of 1.3 which discarded fascicles outside 1.3 standard deviations of the mean. These fascicles were those which were obviously not physiologically feasible, as they often ran perpendicular to most of the other fascicles visualized. However, it should be noted that this cut‐off criteria occasionally excluded fascicles that were likely part of the true distribution.

We finally exported these fascicles as STL cylinders for 3D rendering, allowing us to color‐code fascicles for each muscle using the Color Management function in *Amira* (Figure 1).

**FIGURE 1:**
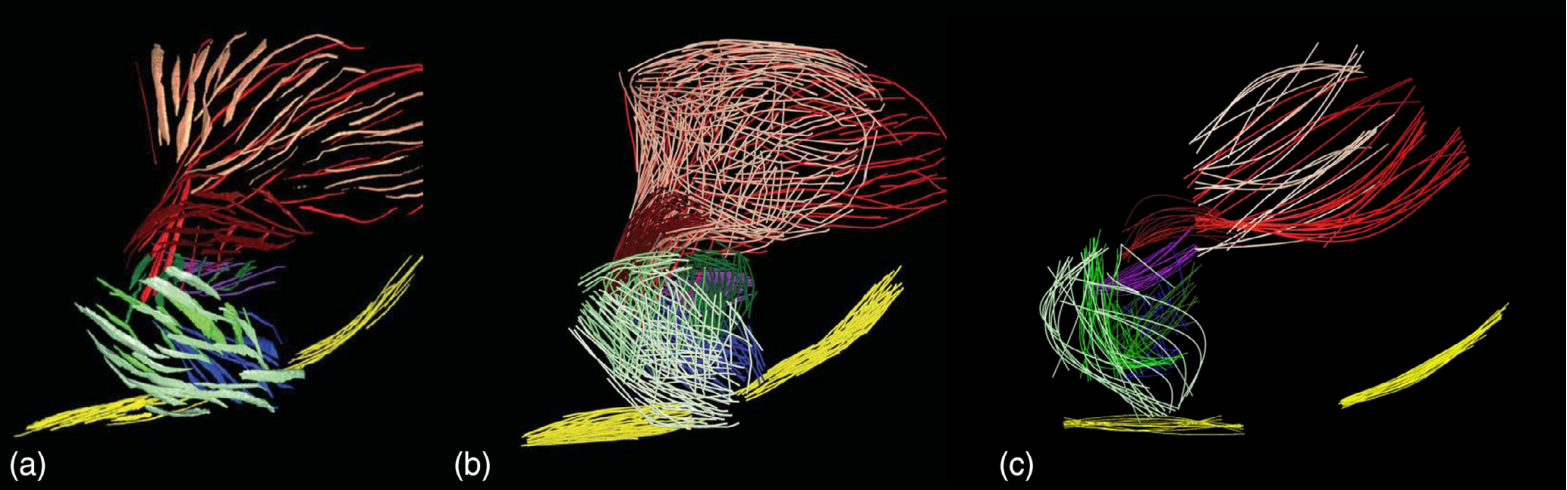
Workflow for isolating jaw muscle fascicles from *Eulemur coronatus* using manual segmentation (a), *XFiber* (b), and *GoodFibes* (Arbour, 2023) (c). The 10 muscles identified are Anterior and Posterior Digastrics in yellow, Superficial Temporalis in light pink, Deep Temporalis in red, Zygomatic Temporalis in maroon, Superficial Masseter in mint green, Deep Masseter in bright green, Zygomaticomanibularis in dark green, Lateral Pterygoid in blue, and Medial Pterygoid in purple. All fascicles represent lengths and tortuosity. Manual segmentation fascicle width represents actual fascicle width, while *XFiber* and *GoodFibes* line width is not representative of width, it is strictly visual. Reconstructed masticatory muscular from *E. coronatus* using manual segmentation is reprinted with permission from Dickinson et al. (2024).

### 4.3 Statistical analyses

To statistically confirm the success of the chosen standardized protocol (see above), we performed a series of *t*‐tests between fascicles generated using optimal settings and fascicles generated with each of the subsequent parameter changes (Table S1). For Minimum Seed Correlation and Minimum Continuation Quality the value was changed to either 50% higher or lower than the ideal value chosen using the scan gray values. For Direction Coefficient, 0.3 was used for most muscles, so we also tested lower values of 0.2 and 0.1 which would yield more artificially straight fascicles. If a value below 0.3 was chosen as optimal then we would test 0.3 for that given muscle as well. The Minimum Length parameter value was optimally set as 70% of the initial Cylinder Length inputted for the algorithmic fascicle correlation, so both the 100% Cylinder Length and 50% Cylinder Length were used for comparison to their fascicle output. Ultimately, the most sensitive variables were Minimum Continuation Quality, Direction Coefficient, and Minimum Length, which each yielded significantly different fascicle lengths when the parameters where manipulated (Table S1).

Data were compared between four methods: gross dissection, manual fascicle segmentation, *XFiber* algorithmic resolution, and *GoodFibes* algorithmic resolution (Appendix S1). The latter three methods yielded directly comparable data, as: (1) the measurements all came from the same CT scan of the same individual, and (2) full data on length and orientation were collected for every fascicle. By contrast, the literature values used to represent gross dissection came from conspecific individuals of that species and reported only a mean fascicle length (with standard deviation) for any given muscle. This hindered direct statistical comparisons across all four groups. Additionally, tortuosity (the measure of each fascicle's inherent curvature) was not reported for gross dissection, as it needs to be measured from in situ fascicles.

To evaluate and compare methodologies, we calculated the percent change between each individually derived fascicle length from manual segmentation, *GoodFibes*, and *XFiber* relative to the mean fascicle length value derived from the literature, where possible (gross dissection were not reported, for example, for the lateral pterygoid or digastric muscle in our sampled carnivores, or for the anterior and posterior digastric muscles of sampled primates). We also calculated the percent change between each individually derived fascicle length and tortuosity measurement from *GoodFibes* and *XFiber*, relative to a mean reference value for manual segmentation. Using this method, positive values for percent change indicate an overestimation of fascicle length or tortuosity relative to the reference value, while negative values indicate an underestimation relative to the reference value. As would be expected, a value closer to zero implies a reduction in the absolute differences between the two samples.

To investigate the factors driving differences between methodologies, we constructed a series of linear mixed‐effects models following Winter (2013) and Bates et al. (2015). All statistical analyses were conducted in R (R Core Team, 2021) using the packages lme4 (Bates et al., 2015), lmerTest (Kuznetsova et al., 2017), ape (Paradis & Schliep, 2019), vegan (Dixon, 2003), and emmeans (Lenth, 2018).

To account for the shared evolutionary history among species, the phylogenetic structure of the dataset was modeled using phylogenetic eigenvectors (PEVs; Table 1), following Diniz‐Filho et al. (2015). In this approach, the shape of the phylogenetic tree is decomposed into a set of orthogonal linear contrasts (i.e., *n* − 1 contrasts for *n* taxa), allowing for the explicit modeling of phylogenetic relationships. Unlike traditional comparative methods that rely on species‐level averages, PEVs allowed us to retain individual‐level variation—an important feature given our dataset includes multiple observations per species and muscle.

**TABLE 1:** Statistical parameters derived from linear mixed effect models demonstrating the statistical importance of various fixed effects on the percent change of fascicle length and tortuosity.

Model	Variable		Estimate	Standard error	Degrees of freedom	*t*‐value	*p*‐value
Model 1	(Intercept)		121.57	12.95	6.01	9.39	<0.001
	Method	Manual segmentation	−115.44	2.90	5032.16	−39.87	<0.001
		*XFiber*	−111.63	2.27	5031.44	−49.28	<0.001
	Phylogenetic eigenvector	PEV1	−0.07	0.13	3.00	−0.57	0.611
		PEV2	0.13	0.35	3.00	0.37	0.734
		PEV3	0.18	0.40	3.00	0.44	0.691
		PEV4	0.52	0.54	3.00	0.96	0.407
Model 2	(Intercept)	(Intercept)	183.16	22.31	6.58	8.21	<0.001
	Method	*XFiber*	−148.54	5.09	5139.13	−29.21	<0.001
	Function	Adductor	−63.15	16.41	10.25	−3.85	0.003
	Phylogenetic eigenvector	PEV1	−0.12	0.21	3.00	−0.55	0.623
		PEV2	−0.40	0.59	3.00	−0.69	0.541
		PEV3	0.35	0.68	3.00	0.51	0.647
		PEV4	0.48	0.92	3.00	0.53	0.635
	Interaction (Method × Function)	*XFiber* × Adductor	38.12	5.70	5138.76	6.69	<0.001
Model 3	(Intercept)		−0.41	0.81	13.02	−0.51	0.621
	Method	*XFiber*	−2.07	0.26	5139.00	−7.87	<0.001
	Function	Adductor	−0.21	0.83	10.42	−0.26	0.803
	Phylogenetic eigenvector	PEV1	−0.01	0.01	3.01	−1.54	0.221
		PEV2	0.00	0.01	2.99	−0.25	0.822
		PEV3	0.03	0.02	3.00	1.44	0.245
		PEV4	0.02	0.02	3.00	1.01	0.389
	Interaction (Method × Function)	*XFiber* × Adductor	−2.15	0.29	5139.00	−7.29	<0.001

*Note*: The first model compared fascicle lengths from digital methods to literature‐derived values based on gross dissection (Dickinson et al., 2024; Hartstone‐Rose et al., 2022). Because these references were limited to adductor muscles, the Function variable was excluded. The second and third models assessed percent differences in fascicle length and tortuosity, respectively, between manual segmentation and the two digital methods, incorporating the Method × Function interaction in both cases. Phylogenetic eigenvectors were included in all models to assess whether phylogeny influenced the ability to accurately reconstruct fascicle length and tortuosity from digital reconstructions. The variable not listed is the reference [i.e., *GoodFibes* (Arbour, 2023) and abductor].

Phylogenetic eigenvectors were generated from a consensus tree with least‐squares branch lengths (Revell, 2012), constructed using data from TimeTree.org (Kumar & Hedges, 2011). A steepness parameter of 0 was applied, modeling trait evolution as gradual change along branches (Guénard et al., 2013). Although this method yields *n* − 1 eigenvectors, only the first four were retained as predictors in the final models. These were selected using a backward model selection approach, based on changes in Akaike Information Criterion and log‐likelihood ratio tests. Including additional eigenvectors did not improve model fit and unnecessarily increased model complexity.

To evaluate sources of methodological discrepancy, we constructed three separate linear mixed‐effects models. In each model, percent difference was the response variable. Fixed effects included Method (i.e., *GoodFibes*, *XFiber*, or manual segmentation), muscle function (i.e., adductor vs. abductor), and the first four phylogenetic eigenvectors (PEV1–PEV4). Muscle function was included in the model because muscles vary in their architectural complexity (Dickinson et al., 2024; Dickinson, Kolli, et al., 2020; Granatosky & Ross, 2020; Hartstone‐Rose et al., 2022). In this dataset, jaw adductors—particularly those in the temporalis region—are generally more functionally complex and heterogeneous than jaw abductors (Castroflorio et al., 2008; Grünheid et al., 2009; Koolstra & van Eijden, 1995; Plesh et al., 1996). This simplicity of primate masticatory abductor architecture has been observed using gross dissection methods as well (Dickinson et al., 2022). Given this complexity, automated reconstruction methods may be more susceptible to error when estimating fascicle architecture in adductor muscles compared to the relatively simpler morphology of abductors. Species and muscle were included as random effects to account for the hierarchical structure and repeated measures. Where applicable, a Method × Function interaction term was added to test whether reconstruction accuracy varied by muscle type.

The first model compared fascicle lengths from digital methods to literature‐derived values based on gross dissection (Dickinson et al., 2024; Hartstone‐Rose et al., 2022). Because these references were limited to adductor muscles, the Function variable was excluded. The second and third models assessed percent differences in fascicle length and tortuosity, respectively, between manual segmentation and the two digital methods, incorporating the Method × Function interaction in both cases. Assumptions of normality and homogeneity of variance were evaluated using Shapiro–Wilk and Levene's tests. Since the data met these assumptions, no transformations were applied. Post‐hoc comparisons of estimated marginal means were conducted using Tukey‐adjusted contrasts in emmeans.

As raw values for fascicle length and tortuosity were available for manual segmentation, *GoodFibes*, and *XFiber*, we conducted additional hypothesis tests. Shapiro–Wilk and Levene's tests were used to evaluate the distributional properties of these data. As the raw values were not normally distributed, Mann–Whitney *U*‐tests were used to assess whether fascicle length and tortuosity values from *GoodFibes* and *XFiber* differed significantly from those obtained via manual segmentation.

## 5 RESULTS

### 5.1 Sensitivity testing of XFiber parameters

As described above, before determining a standardized protocol for *XFiber* (Appendix S2), numerous sensitivity analyses were conducted to quantify the specific effects of each parameter upon the resulting fascicle dataset. For illustrative and comparative purposes, we present data on each variable three ways (a value derived by following the guidelines laid out in methods for their determination, as well as values 50% higher and lower, respectively) to allow for interpretation of both: (1) how sensitive specific values are to change, and (2) the directional effects of changing each value up or down upon the resultant fascicle dataset. Two parameters are presented differently: for Direction Coefficient, values above 0.3 had little to no effect on the data, so we instead present data on coefficient values of 0.1, 0.2 and 0.3 for comparison; additionally, for cylinder length we present values of full reference cylinder length, 70% of reference cylinder length (our optimal parameter), and 50% of reference cylinder length. Details on the determination of each parameter are presented here.

The first necessary input for the Trace Correlation Lines function was a guide for cylinder length. Numerous values were tested, with the most consistently plausible fascicles generated using a cylinder length input 0.7× the length of the reference cylinder used within the initial “Cylinder Correlation” function used to generate the correlation and orientation fields. Increasing cylinder length above this value—particularly as this value approached or exceeded the original length of the reference cylinder—caused the correlation to identify only excessively long fascicles, while choosing a very short length caused the program to split longer fascicles into two or more pieces, yielding much shorter fascicles on average (Figure S1). Increasing the length of the reference cylinder also increased tortuosity (Figure S2), while lowering the reference cylinder length produced less tortuous fascicles. Thus, we emphasize here the need to select a high‐quality reference cylinder, as this decision affects all downstream functions.

As described above, while a single‐fascicle protocol (i.e., use of a single reference cylinder per muscle volume) yields good results for muscles with relatively simple architectural configurations (e.g., parallel‐fibered or unipennate muscles where fascicles run the full muscle length, or symmetrically bipennate muscles), more complex multipennate muscles were difficult to adequately represent with a single fascicle. For this reason, we also tested several variants of the Multiple Cylinder Correlation function, exploring the effects of using two, three, and five fascicles per muscle. We observed diminishing returns from the use of more than three cylinders per muscle and also note that the addition of extra cylinders extends the process time significantly: with some larger muscles taking up to 10 h. For comparison, using smaller muscles and a single reference cylinder, we extracted fascicles in as little as 6 min on a standard desktop computer.

The next required input was for Minimum Seed Correlation, which represents the minimum level of correlation required for a potential seed location. Adjusting this value upwards increased the number of potential seed points but, above a certain threshold, irrelevant (i.e., low correlation) seed points may be included, leading to the generation of artificial fascicles from boundary voxels surrounding each muscle. Higher values included fewer potential boundary errors, reducing the number of fascicles yielded; however, above a certain threshold true fascicles were also dropped from the visualization. Notably, while raising the Minimum Seed Correlation by 50% had a small but directionally variable impact upon tortuosity, lowering Minimum Seed Correlation by 50% removed almost all tortuosity from the resultant fascicles of many muscles. Thus, care should be taken to select a sufficiently high value for this variable to ensure that fascicles can follow biological curvature. To this end, we found that an optimal Minimum Seed Correlation value could be approximated through use of the Isosurface function (native to *Amira*, rather than *XFiber* specifically). Using an isosurface module, it is possible to adjust the intensity value to a point where internal fascicles were visible, but external artificial volume boundaries were not. This intensity number was then directly copied into the Minimum Seed Correlation input for fascicle generation.

Next, the Minimum Continuation Quality value determines the threshold of correlation values needed to continue a line/fascicle. A higher Minimum Continuation Quality value is inherently more conservative and may lead to earlier points of termination (i.e., shorter lines) and fascicles that are too straight (i.e., artificially low tortuosity). However, a Minimum Continuation Quality that is too low may artificially extend fascicles beyond their true length and produce fascicles that are far too tortuous. To determine an input for this field, we used the Orthoslice function (again in base *Amira*, rather than an *XFiber* function) and adjusted the cutoff point to the highest value where all fascicles were still visible, but other connective tissue was no longer visible. This ensured that lines were only traced within areas of the scan occupied by fascicles, preventing false fascicles from being generated within connective tissues such as the perimysium or fascicles continuing through connective tissues. Overall, we observed that opting towards slightly higher values (i.e., increasing the risk of dropping a few, less radiopaque fascicles, or ending fascicles a few pixels early) was more acceptable than choosing lower values (which allowed for high numbers of false fascicles to be included within the results).

The Direction Coefficient determines the degree of curvature (i.e., tortuosity) present within possible fascicles, setting an upper bound on inherent curvature. This variable influences how much deviation from a straight line is allowed. Higher values make it more likely to follow curves (increasing tortuosity), while lower values promote straighter lines (reducing tortuosity). By default, this value is set to 0.3, and we found that in most cases, this proved effective at yielding biologically viable fascicles and excluding potential outliers. However, in some cases, it was necessary to constrain the direction coefficient to lower bounds, as a value of 0.3 occasionally yielded highly curved and unfeasible fascicles (i.e., clear errors) in otherwise architecturally homogenous muscles.

Next, the Minimum Distance function specifies a spatial offset between adjacent centerline, prevents tracing lines within the cylinders defined by other lines and this radius value. This value alters the spacing between adjacent fascicles—essentially governing the density of the resultant fascicle map. Lowering this value allows for more potential fascicles to be generated—but also risks overpopulating the map visually (making it difficult to discern individual structures) or to have multiple lines generated within a single fascicle. We derived the most consistent results by starting with a Minimum Distance value of around 0.5 (i.e., 500 μm spacing, assuming a scan voxel size input in millimeters) and lowering incrementally from this point. Within our datasets, we stayed above a threshold of 150–200 μm as this converges upon the cross‐sectional area of a single fascicle in our sample. However, studies of absolutely smaller organisms, or of neonatal or embryological specimens, may wish to apply a lower minimum threshold.

The final two input values are for the Search Cone and Angle, that specify the maximum step size between two line points, and the full aperture angle of the search cone itself, respectively. These reflect how far and how wide the algorithm would search for the continuation of a fascicle from one point to the next. Through testing within our sample, we found that these base values were suitable for all muscles within our datasets; however, scans of differing quality or studies of highly tortuous structures that changed shape more acutely from one slice to the next may wish to alter these values to allow for broader searching.

### 5.2 Sensitivity testing for GoodFibes

While an established workflow already exists for *GoodFibes* (see Arbour, 2023), we attempted to evaluate our own data by comparing our standard input parameters against a locally optimized workflow that allowed us to vary individual input values for each region within the dataset. The results for this are presented in Table S2 and Figures S3 and S4. Use of the locally optimized input parameters served to reduce fascicle lengths in almost all cases and resulted in fascicle data that more closely approximated other methods (Figure S3; Table S2). By comparison, tortuosity was less affected by changes to the input parameters, though differences were still observed in most muscles, with tortuosity being lower overall within the locally optimized dataset (Figure S4). Thus, the locally optimized dataset was utilized in all subsequent inter‐method comparisons.

### 5.3 Comparisons between methods

Compared to gross dissection, manual segmentation overestimated fascicle length by 12.88% ± 51.42%, *XFiber* by 10.93% ± 48.54%, and *GoodFibes* by 124.17% ± 116.59% (Figure 2). Linear mixed‐effects modeling (with muscle and species as random effects, and Method and the first four phylogenetic eigenvectors as fixed effects) confirmed significant differences between digital methods (*p* < 0.001). None of the PEVs significantly predicted variation in percent error.

**FIGURE 2:**
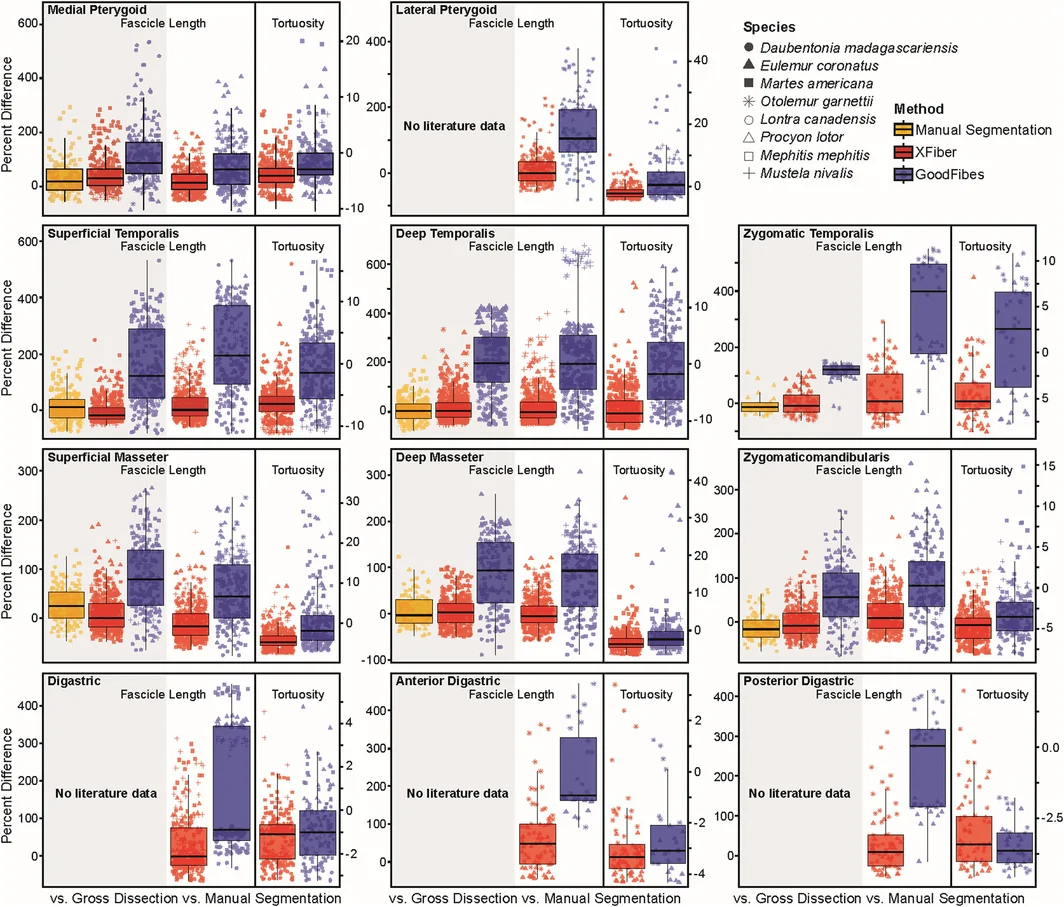
Percent difference in methodology between gross dissection, manual segmentation, *XFiber*, and GoodFibes when measuring fascicle lengths (left) and fascicular tortuosity (right). Methods were only compared against gross dissection when conspecific literature values were available. Gross dissection values for primates were collected from Dickinson et al. (2024) and carnivores from Hartstone‐Rose et al. (2022). Note that two major differences exist within the gross dissection data collected from primates and carnivores. First, a zygomatic portion of the temporalis was observed within primates, but not carnivores. Secondly, data from the masseteric and temporalis complex were aggregated together within carnivores (i.e., presented at the “masseter” and “temporalis” levels); whereas data were collected for each individual component (e.g., superficial masseter, deep masseter, zygomaticomandibularis, superficial temporalis, and deep temporalis) in primates, allowing for more direct comparison of muscle architectural properties between individual muscle layers within these species.

Fascicle lengths derived from *GoodFibes* were also substantially higher than those from manual segmentation for both abductors and adductors. Specifically, for manual segmentation–based comparisons, *GoodFibes* overestimated fiber length by 172.52% ± 140.52% for jaw abductors and 119.16% ± 130.51% for jaw adductors. In contrast, *XFiber* overestimated values by 25.81% ± 72.51% (abductors) and 7.58% ± 49.56% (adductors). Post‐hoc pairwise comparisons confirmed these effects, with significant differences between *GoodFibes* and *XFiber* across both functional groups (*p* < 0.0001). A significant interaction between method and function was also observed (*p* < 0.0001), with greater overestimation in abductors than adductors. None of the PEVs significantly predicted variation in percent error.

Tortuosity values exhibited a different pattern. *GoodFibes* slightly overestimated tortuosity compared to manual segmentation for abductors (0.23% ± 5.96%) and adductors (−0.89% ± 6.30%), while *XFiber* consistently underestimated values (−1.79% ± 1.79% for abductors; −5.18% ± 3.3% for adductors). These differences in how the methods performed were also statistically significant (*p* < 0.0001), with a strong interaction between method and jaw function evident in the model. As with fascicle length, PEVs had no significant explanatory power in explaining error in tortuosity.

A direct comparison between fascicle lengths derived from manual segmentation and *XFiber* revealed that 47.76% of comparisons were significantly different. In contrast, 95.38% of tortuosity comparisons between *XFiber* and manual segmentation were significantly different, indicating substantial divergence in estimating fascicle path complexity (Table S5). Comparisons between manual segmentation and *GoodFibes* showed even greater differences in fascicle length estimates, with 93.94% of comparisons being significantly different. For tortuosity, 77.27% of comparisons between *GoodFibes* and manual segmentation were significantly different (Table S6). These findings suggest that, while both digital methods diverge from manual segmentation, *XFiber* tends to yield more similar fascicle length estimates, whereas *GoodFibes* shows somewhat better agreement in tortuosity estimates. However, the consistently high rates of statistical differences underscore systematic discrepancies between automated and manual approaches, especially in muscles with complex architecture.

## 6 DISCUSSION

Overall, *XFiber* shows promise as an algorithmic digital dissection tool. The fascicles generated by *XFiber* were visually consistent with those obtained through manual segmentation and remained within physiologically feasible limits. However, *XFiber* tended towards fascicle length overestimation—by approximately 11% compared to gross dissection and 17% compared to manual segmentation. While differences between this technique and gross dissection can be attributed to intraspecific variation (as data were collected on conspecifics; see Section 7), the observed difference relative to manual segmentation reflects true methodological differentiation, as the same CT scans were used in each case. Additionally, *XFiber* struggled to accurately represent fascicles with higher tortuosity, leading to an underestimation of fascicular curvature by about 3.5%.

### 6.1 XFiber parameterization and biological challenges

Our default *XFiber* parameters were chosen to constrain the user bias and set post‐correlation values derived from the gray values of the CT scan itself. Before running a cylinder correlation, the user only needs to input a single example fascicle length and width, both inputs that are guided by visually tracing out a fascicle on the scan. As such, choosing a clear representative fascicle is incredibly important, as this affects all downstream elements of the workflow.

As discussed above, extensive sensitivity testing was conducted on all input variables. This provided a clear indication of how each variable altered the generated fascicle field, as well as the variables that contributed most significantly to variation in the final dataset. For example, our findings suggest that changes to Minimum Continuation Quality and Direction Coefficient generally yield large changes to the resultant data, whereas the parameters Minimum Distance and Minimum Seed Correlation were less sensitive and resulted in fewer changes to the resultant data (Table S1 and Figure S1).

However, even once a standardized operating procedure (Appendix S2) with optimal parameter guidelines was established, the curvature and complexity of fascicles within the mammalian jaw musculature still posed difficulties for the program, often yielding a handful of fascicles that ran perpendicular to the majority of the distribution. Additionally, these oddly oriented lines were most often found towards the outer perimeter of the model. On this basis, we conclude that they are artifacts of the program that do not represent muscle fascicles, but rather connective tissue or an artificial shell of the muscle volume generated by the process of isolating individual muscles from a larger CT stack. To limit these errors, we have been applying a post‐hoc *z*‐score of 1.3 standard deviations onto the Orientation Theta variable, excluding fascicles whose orientation falls outside of this range. It is important to reiterate here that *XFiber* resolves better with simpler muscles, but with this *z*‐score we are able to continue to keep user bias to a minimum instead of allowing the user to individually select fascicles to remove from the final model.

In addition to assessing the efficacy of this workflow, we also explored potential sources of methodological variation. As jaw muscle architecture is reported as more complex among primates than carnivorans (Davis et al., 2024; Davis & Williams, 2017; Gorniak & Gans, 1980; Granatosky et al., 2019; Laird et al., 2020, 2023), we assessed whether reconstruction methods would encounter more errors within the former sample. This was not the case. Our analysis suggests that *XFiber* can be applied to similar success across a broad phylogenetic range. Nevertheless, greater phylogenetic diversity, including species outside Mammalia, is needed to verify this claim. Additionally, although our sample included taxa spanning a large body size range (~50 g to ~10 kg), this range is dwarfed by the diversity found in extant mammals. Further testing is required to evaluate the full influence of body size on automated reconstruction methodologies.

We also explored differences between myological groups. Jaw adductors (i.e., the medial pterygoid, superficial, deep, and zygomatic portions of the masseter) exhibit more complex 3D architectural variation than jaw abductors (i.e., the lateral pterygoid and digastric muscles). We therefore expected automated muscle architecture methods to be more error‐prone when reconstructing jaw adductors. This expectation was partially supported. Compared to fascicle lengths derived from manual segmentation (i.e., data collected from the same CT scans, ensuring observed differences reflect only methodology), both *XFiber* and *GoodFibes* did yield better estimations of fascicle length for the jaw adductors (7.58% and 119.16%, respectively) than for the jaw abductors (25.81% and 172.52%, respectively). However, for tortuosity, both *XFiber* and *GoodFibes* yielded better predictions for the jaw abductors (differences of 1.79% and 0.23%, respectively) than for the jaw adductors (5.18% and 0.89%, respectively).

This variation suggests the potential need for a sliding scale of parameters depending on muscle pennation, fascicle length, or tortuosity, as opposed to a single, universal protocol. Although the current *z*‐score cutoff within *XFiber* adjusts for orientation theta values to avoid perpendicular artifacts generated by the reconstruction algorithm (which occur primarily in multipennate complex muscles), we might be overlooking the need to address algorithmic inaccuracies that are not immediately visually apparent. While cross‐hatching fascicles are identified upon model generation, muscles that appear to have fascicles running in the same direction might be concealing other *XFiber* errors, such as attaching fascicles end to end when they should be cut off. Future work may require different *z*‐scores applied not to orientation theta, but to curve length values themselves. Alternatively, we might need to standardize the expected fascicle ranges. Although some variation is natural, physiologically, fascicles in simple parallel‐fibered muscles that do not require extensive complex fan shapes would be expected to have a more constrained range of lengths than those with greater architectural complexity.

### 6.2 Comparisons between automated protocols

Both *XFiber* and *GoodFibes* apply algorithmic protocols to the resolution of fascicle architecture, though we observed herein that *GoodFibes* produced fascicle lengths far longer than other methods (either manual or algorithmic). However, in terms of tortuosity, *GoodFibes* more closely approximated the arc‐to‐straight length ratio compared to manual segmentation than *XFiber* did. This likely reflects differences in the application of a directional constraint within this protocol compared to *XFiber*, in which the user must specify a priori a direction coefficient that curtails potential tortuosity within the sample. GoodFibes, by comparison, utilizes a whole‐integer degrees of freedom approach (that was set to 2 in all cases within our dataset, as a value of 1 produced only straight fascicles, and a value of 3 or above introduced biologically unreasonable degrees of tortuosity in many fascicles).

It should be clearly stated that comparisons made between methods here are not definitive. Firstly, due to the use of literature data from conspecific individuals (see limitations), comparisons between any digital method and gross dissection should not be considered a one‐to‐one methodological appraisal. In addition, the parameter testing conducted within *XFiber* was not exhaustive: for instance, the search cone and angle functions were not altered between scans, but fine‐tuning of these parameters could improve the quality of reconstruction for specific scans. Most notably, the number of parameter conditions tested here for *XFiber* drastically exceed those of *GoodFibes*, which likely accounts for some discrepancies between the findings of the two methods. Similarly, extensive testing of individual parameters across the *GoodFibes* reconstruction workflow would be invaluable in future work to ascertain how comparable these streams may become under idealized settings. While the authors made every effort to follow steps outlined by Arbour (2023) in the publication of this method, their experience in this technique is admittedly lower than with *Amira* and its extensions such as *XFiber*. As the comparisons between the two *GoodFibes* workflows analyzed herein reveal, even relatively minor modification of the protocol to suit individual stack properties can yield improvements to the resultant architectural variables. Thus, we reserve any judgment on the optimal techniques for use in automated architectural reconstruction workflows until such time as a similarly rigorous sensitivity assessment is conducted across all techniques.

### 6.3 How much error is acceptable?

Fundamentally, our findings highlight a pervasive mismatch between methodological approaches to architectural resolution. Some of this—for example, the differences between gross dissection and all digital methods—can be ascribed to differences in the sample itself. However, the same isolated muscle stacks, extracted from the same underlying CT datasets, were used for manual segmentation, *XFiber*, and *GoodFibes*—yielding three ultimately different results.

As such, we must consider a number of broader, overarching questions. Firstly: what is our gold standard? Of the three digital techniques, manual segmentation certainly offers the user most control over the resultant data—both in the selection of which fascicles to reconstruct, and in determining their start and end points (Dickinson et al., 2018, 2024). However, this inherent subjectivity may also be the greatest flaw in this method: it requires a good understanding of gross and fascicular myological anatomy, reducing inter‐user replicability (Dickinson, Atkinson, et al., 2020). In addition, the method requires substantial investment of time; while algorithmic resolution of a medium‐sized muscle from our sample (e.g., the deep masseter) can be done in 15 min (the majority of which is unsupervised processing time), the same procedure manually would take several hours of involved user input. Given these shortcomings, is this necessarily the optimal method for comparative studies—particularly if a large sample of specimens is required?

By contrast, *XFiber* yields somewhat similar data with a fraction of the required user input time. Yet the method is not without shortcomings: the potential for false fascicles to remain within the sample is clear, even when following our suggested protocol (Appendix S2) of a post‐hoc filter based on orientation. Of course, known erroneous fascicles could then be manually cleaned up (a process which was not used here, to more objectively compare methods)—but this introduces elements of subjectivity into the workflow once more.

The lack of clarity regarding the “optimal” method for data collection obscures our ability to address a similarly pertinent question: how much error should be acceptable? Comparative anatomical studies, even in best‐case scenarios, represent an approximation of muscle function—for example, during gross dissection, typically a subset of muscle fascicles are measured and averaged out to represent the muscle at large (Boettcher et al., 2019; Dickinson et al., 2021; Hartstone‐Rose et al., 2022; Leischner et al., 2018). This inherently introduces one form of error: by not measuring every fascicle, we are accepting a loss in data resolution. Indeed, a recent methodological appraisal by Charles et al. (2022) suggests that analyzing small (<25) numbers of fibers to represent a muscle's architectural configuration can yield large errors in their characterization.

Among gross dissection methods, a recent methodological comparison between two popular methods (i.e., chemical digestion and in situ sectioning) suggested strong convergence between the two approaches: with the former yielding longer fascicle lengths in 53% of samples, and the latter the remaining 47%—suggesting that there is no consistent directionality in which one method promotes longer fascicle measurements than the other (Taylor et al., 2024). That sample compared the different methods using different individuals for each method, confounding the methodological comparison in the same way that our digital data cannot be directly compared to fascicular data from the literature—even data that we collected ourselves (Dickinson et al., 2024; Hartstone‐Rose et al., 2019)—given that these data were also collected on different specimens from the same species.

With that said, the fact that differences *do* occur between methods—whether they are the result of intraspecific confound or directionality one way of the other even on data collected from the same individuals—suggests a meaningful contribution of method on the final dataset.

In this context, then, we suggest that the field offers numerous approaches to the resolution of fascicle architecture, including multiple methods of fascicle analysis through gross dissection, as well as various techniques for the digital resolution of fascicle arrangement (Arbour, 2023; Dickinson et al., 2018; Dickinson, Atkinson, et al., 2020; Hartstone‐Rose et al., 2022; Kupczik et al., 2015; Sullivan et al., 2019). Yet, we still lack a consensus regarding the “best” way to do this. It should also be noted that the time investment of each method varies drastically: while gross dissection data can be collected within a day or two (~1–2 h for dissection, ~6–24 passive hours for acid digestion, ~2–4 h for manual fascicle separation, and ~1–2 h for fascicle photography and the digital measurement of fascicles), contrast‐enhanced staining requires at least 1 month of (passive) time investment during the staining process, as well as subsequent time to collect and digitally process the resultant CT data. While algorithmic approaches to fascicle reconstruction substantially lower the active time relative to manual segmentation (with small muscles resolving in under 10 min, and larger muscles taking a handful of hours, compared to the ~3–10 h per muscle needed for manual fascicle tracing), this additional time requirement quickly becomes problematic for large‐sample, comparative studies that would feature dozens of specimens—that is, the kinds of samples compiled using gross dissection to evaluate adaptive correlates of these architectural variables.

We suggest that, for comparative studies, the key may therefore be consistency: as *in situ* sectioning, chemical digestion, manual segmentation, and the *XFiber* automated protocol outlined here all produce results that are broadly convergent (i.e., within ~10%–15% within a given muscle), choosing a particular method that is convenient for the sample at hand may be sufficient. However, there still exists a major obstacle: namely that this inter‐methodological variation curtails our ability to confidently compare data that were collected between methods, at least in circumstances where the effect size is relatively small, such that the signal to noise ratio is unacceptable. However, as digital methods seem to be getting closer to reproducing anatomy with three‐dimensional spatial relationships intact, and with substantially less damage to often important specimens, it seems that these methods will potentially overtake more traditional methods, especially once the cost of imagery and digital processing becomes sufficiently cheap that it is no longer an accessibility barrier.

## 7 LIMITATIONS

There are several inherent limitations to this study. Firstly, as discussed above, gross dissection data used here as a comparison were collected from the literature using conspecific individuals—a shortcoming of other recent methodological comparisons (e.g., Taylor et al., 2024). This causes two downstream problems. First, it inherently introduces more variation, as no two animals express an identical architectural profile. Thus, uncertainty abounds as to what extent the differences observed between digital data and gross dissection data represent intraspecific variation, versus differences introduced by the methods themselves. Future studies should address this by comparing gross dissection data and digitally reconstructed data within the same specimen. The second limitation from the use of literature values comes from the nature of the data itself: literature values are typically reported as means (typically a single value plus standard deviation) rather than distributions, complicating statistical comparisons with the distributions quantified by other methods.

Additionally, both manual segmentation and our algorithmic methods depend on high‐quality microCT scans for optimal results (Arbour, 2023; Dickinson et al., 2018; Dickinson, Atkinson, et al., 2020; Gignac et al., 2016; Sullivan et al., 2019). These scans require precise specimen staining concentrations and timing, as well as access to high‐powered CT scanners. Fascicles must be fully stained with iodine (or another contrast‐enhancing agent, though it should be noted that only iodine was assessed within this study), and the scanner needs to be powerful enough to clearly distinguish muscle from connective tissue. Understaining makes this distinction impossible, while overstaining may lead to volumetric shrinkage of the muscle, altering the underlying fascicular anatomy (Léonard et al., 2004). One potential alternative to this technique would be the use of microMRI, as opposed to microCT, as an imaging medium. This would potentially remove the need for contrast‐enhanced agents to be introduced to the tissue, since high‐quality microMRI can theoretically facilitate the discrimination of individual fascicles in raw scans. Additionally, microMRI allows the visualization of other tissues (e.g., tendons) within the muscles that are difficult to infer from contrast‐enhanced CT—particularly when iodine (which does not bind readily to collagen) is used as the contrast agent. Indeed, studies that have sought to delineate tendinous tissues from iodine‐enhanced microCT (e.g., Dickinson et al., 2019) have done so through the use of negative space, as the tendons themselves do not uptake the radioopaque solution and are instead visible as dark sheets of low intensity running within the muscle. However, microMRI scanners that produce both enough power to differentiate deep, adjacent tissues and have sufficiently large chamber sizes to allow the inclusion of entire anatomical regions are currently not widely accessible. Nevertheless, it is hoped that within the near future, increased access to this technology will permit new techniques for in situ muscle fascicle discrimination.

Algorithmic methods also present specific challenges. As we have outlined above, changing individual parameters within the reconstruction process can alter the resultant dataset meaningfully (Arbour, 2023; Kupczik et al., 2015; Sullivan et al., 2019). In an attempt to provide a solution to this obvious issue, we have conducted extensive sensitivity testing (all presented here) that attempts to summarize these effects, as well as publish a single protocol that can be applied as a starting point (Appendix S2). Of course, by generating this “standardized” protocol, we are accepting that not every specimen will be perfectly optimized. However, we argue that having standardization across the method carries inherent value, being far more reproducible and less reliant on user subjectivity. As algorithmic approaches improve, it is our hope that the subjectivity inherent in these parameter decisions will be reduced. Once this happens, reproducibility and inter‐investigator reliability will surely improve—a hope for improvement that is unrealistic for traditional gross methods even if protocol descriptions are refined and made as precise as possible.

## 8 CONCLUSION

Here, we outline a standardized algorithmic protocol for the reconstruction of muscle fascicle architecture using the *XFiber* extension in *Amira* (Appendix S2). This extension has been used by several authors over the past few years, yet we herein present the first consistent fascicle generation workflow. We demonstrate that *XFiber* shows promising potential in the growing field of algorithmic digital dissection, producing data broadly comparable to both gross dissection and manual fascicle segmentation. Unlike gross dissection, digital dissection methodologies are less destructive and allow three‐dimensional assessment of muscle fascicles in situ. Moreover, compared to manual methods of digital fascicle generation, this technique is highly replicable, using a standardized set of inputs that can be broadly applied. While numerous competing methodologies exist, we propose that this workflow may be added to the comparative myological toolkit and may offer a meaningful benefit by both reducing time investment and minimizing user bias and subjectivity within the process of fascicle selection and tracing. While highly reliant on a few key features—including high‐quality input scans and the selection of a representative reference fascicle—this toolkit offers a viable option to comparative anatomists who wish to understand muscle structure in 3D space.

## AUTHOR CONTRIBUTIONS

**Aleksandra Ratkiewicz:** Investigation; methodology; validation; visualization; writing – original draft; formal analysis. **Michael C. Granatosky:** Conceptualization; investigation; writing – review and editing; formal analysis; supervision; resources; project administration; data curation; methodology. **Cassidy E. Davis:** Investigation; writing – original draft; methodology; validation; formal analysis. **Julia Molnar:** Conceptualization; investigation; writing – review and editing; validation; methodology; supervision; resources. **Adam Hartstone‐Rose:** Investigation; writing – review and editing; resources; data curation; formal analysis; methodology. **Edwin Dickinson:** Conceptualization; investigation; writing – original draft; writing – review and editing; visualization; validation; methodology; formal analysis; supervision.

## FUNDING INFORMATION

This study received funding from the Center for Biomedical Innovation, New York Institute of Technology College of Osteopathic Medicine, Old Westbury, NY, USA.

## CONFLICT OF INTEREST STATEMENT

The authors have no other conflicts of interest to report.

## DISCLOSURE

Appropriate permissions have been acquired to reproduce a portion of a previously published figure from Dickinson et al. (2024).

## Supporting information


**FIGURE S1.** Box and whisker plots represent the distribution of fascicle lengths depending on which parameter was changed from the optimal set. In each graph, the optimal boxplot is shown in red on the left, followed by gray boxplots named after the altered singular parameter. The abbreviations represent the following: Min. Seed Corr. +50%, Minimum Seed Correlation 50% greater than optimal; Min. Seed Corr. −50%, Minimum Seed Correlation 50% lower than optimal; Min. Cont. Qual. +50%, Minimum Continuation Quality 50% greater than optimal; Min. Cont. Qual. −50%, Minimum Continuation Quality 50% lower than optimal; Dir. Coeff. 0.3, Direction Coefficient set to 0.3; Dir. Coeff. 0.2, Direction Coefficient set to 0.2; Dir. Coeff. 0.1, Direction Coefficient set to 0.1; 100% Cyl. Length, 100% of Cylinder Length value used; 50% Cyl. Length, 50% of Cylinder Length value used. If a graph is missing Minimum Continuation Quality or Minimum Seed Correlation boxplots, it is because the altered parameter could not generate any fascicles.

**FIGURE S2.** Box and whisker plots represent the distribution of tortuosity values depending on which parameter was changed from the optimal set. In each graph, the optimal boxplot is shown in red on the left, followed by gray boxplots named after the altered singular parameter. The abbreviations represent the following: Min. Seed Corr. +50%, Minimum Seed Correlation 50% greater than optimal; Min. Seed Corr. −50%, Minimum Seed Correlation 50% lower than optimal; Min. Cont. Qual. +50%, Minimum Continuation Quality 50% greater than optimal; Min. Cont. Qual. −50%, Minimum Continuation Quality 50% lower than optimal; Dir. Coeff. 0.3, Direction Coefficient set to 0.3; Dir. Coeff. 0.2, Direction Coefficient set to 0.2; Dir. Coeff. 0.1, Direction Coefficient set to 0.1; 100% Cyl. Length, 100% of Cylinder Length value used; 50% Cyl. Length, 50% of Cylinder Length value used. If a graph is missing Minimum Continuation Quality or Minimum Seed Correlation boxplots, it is because the altered parameter could not generate any fascicles.

**FIGURE S3.** Comparison of fascicle lengths between the standardized *GoodFibes* protocol and a locally optimized protocol (as described in Section 4).

**FIGURE S4.** Comparison of tortuosity between the standardized *GoodFibes* protocol and a locally optimized protocol (as described in Section 4).

**FIGURE S5.** Histogram showing changes in fascicle length values at each stage of the *GoodFibes* fascicle reconstruction protocol. Steps were always followed in the same order as presented herein: first an initial reconstruction was applied, followed by a quality check, fusion check, overlap check, and finally a curvature check. Parameters employed at each stage are detailed in the main text.

**FIGURE S6.** Reconstructed fascicles from *Eulemur coronatus* from three techniques: manual segmentation (a), *XFiber* algorithmic reconstruction (b), and *GoodFibes* algorithmic reconstruction (c). Fascicle colors as follows: superficial masseter (pale green), deep masseter (green), zygomaticomandibularis (dark green), superficial temporalis (pink), deep temporalis (red), zygomatic temporalis (maroon), medial pterygoid (blue), lateral pterygoid (purple), digastric (yellow).

**APPENDIX S1:** All data necessary to reconstruct the analyses presented in this study.

**APPENDIX S2:** Standardized protocol for fascicle generation, outlining a workflow where all input properties are calculated directly from raw DiceCT datasets and their associated metadata.

**TABLE S1.** Statistical results derived from comparisons of fiber length and tortuosity generated from the various *XFiber* parameters. Each box provides the *t*‐value; degrees of freedom; *p*‐value; and confidence interval (minimum–maximum). Any missing comparisons mean that fascicles that were not generated. The abbreviations represent the following: Min. Seed Corr. +50%, Minimum Seed Correlation 50% greater than optimal; Min. Seed Corr. −50%, Minimum Seed Correlation 50% lower than optimal; Min. Cont. Qual. +50%, Minimum Continuation Quality 50% greater than optimal; Min. Cont. Qual. −50%, Minimum Continuation Quality 50% lower than optimal; Dir. Coeff. 0.3, Direction Coefficient set to 0.3; Dir. Coeff. 0.2, Direction Coefficient set to 0.2; Dir. Coeff. 0.1, Direction Coefficient set to 0.1; 100% Cyl. Length, 100% of Cylinder Length value used; 50% Cyl. Length, 50% of Cylinder Length value used.
**TABLE S2:** Statistical results derived from comparisons of fascicle length and tortuosity between a standardized *GoodFibes* parameter input and locally optimized input variables that utilized slice‐specific threshold values for each muscle region and applied a more aggressive upper limit to the cutoff criteria (see Section 4).
**TABLE S3:** Means and standard deviations of fascicle length and tortuosity for the various reconstruction methods assessed in this study. Gross dissection values for primates were collected from Dickinson et al. (2024) and carnivores from Hartstone‐Rose et al. (2022). Note that two major differences exist within the gross dissection data collected from primates and carnivores. First, a zygomatic portion of the temporalis was observed within primates, but not carnivores. Secondly, data from the masseteric and temporalis complex were aggregated together within carnivores (i.e., presented at the “masseter” and “temporalis” levels); whereas data were collected for each individual component (e.g., superficial masseter, deep masseter, zygomaticomandibularis, superficial temporalis, and deep temporalis) in primates, allowing for more direct comparison of muscle architectural properties between individual muscle layers within these species.
**TABLE S4:** Means and standard deviations of percent change (%) subdivided by species and muscle for the various reconstruction methods assessed in this study.
**TABLE S5:** Results from Mann–Whitney *U*‐tests comparing whether fascicle lengths and tortuosity estimated from *XFiber* differed significantly from those derived from manual segmentation.
**TABLE S6:** Results from Mann–Whitney *U*‐tests comparing whether fascicle lengths and tortuosity estimated from *GoodFibes* (Arbour, 2023) differed significantly from those derived from manual segmentation. Blank spaces represent muscles that could not be algorithmically generated by *GoodFibes*.

## Data Availability

All data used in this study is available as Appendices S1 and S2.
