# Supplementary material for: Multi‐method analysis for the three‐dimensional reconstruction of muscle fascicles from DiceCT datasets
Source: Anat Rec (Hoboken). 2025 Dec 4;309(12):3491–510. doi: 10.1002/ar.70088 (PMC13749328; doi:10.1002/ar.70088)
Supplement: Supplementary file 1 [file AR-309-3491-s001.pdf]

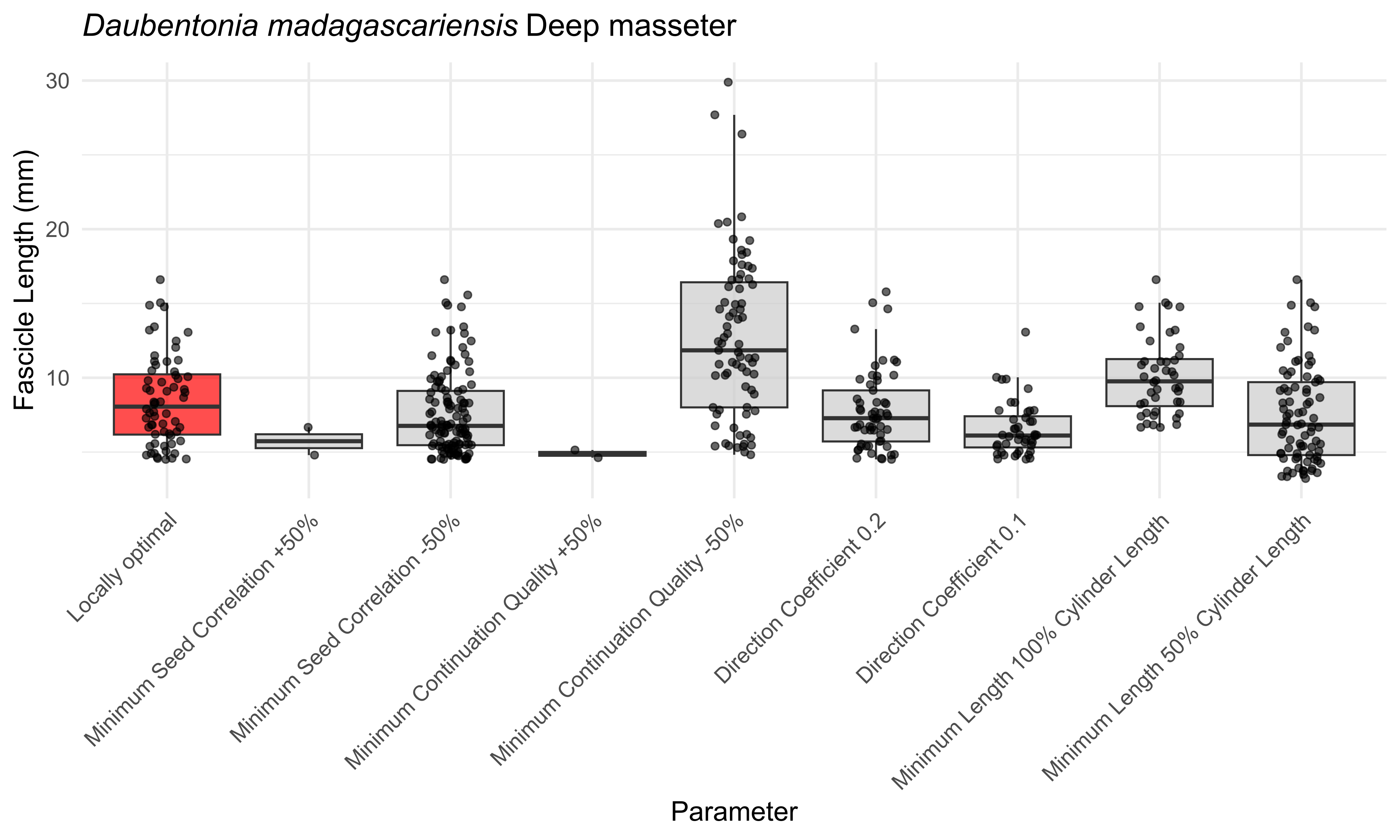

Daubentonia madagascariensis Deep masseter
Fascicle Length (mm)
30
20
10
Locally optimal
Minimum Seed Correlation +50%
Minimum Seed Correlation -50%
Minimum Continuation Quality +50%
Minimum Continuation Quality -50%
Direction Coefficient 0.2
Direction Coefficient 0.1
Minimum Length 100% Cylinder Length
Minimum Length 50% Cylinder Length
Parameter

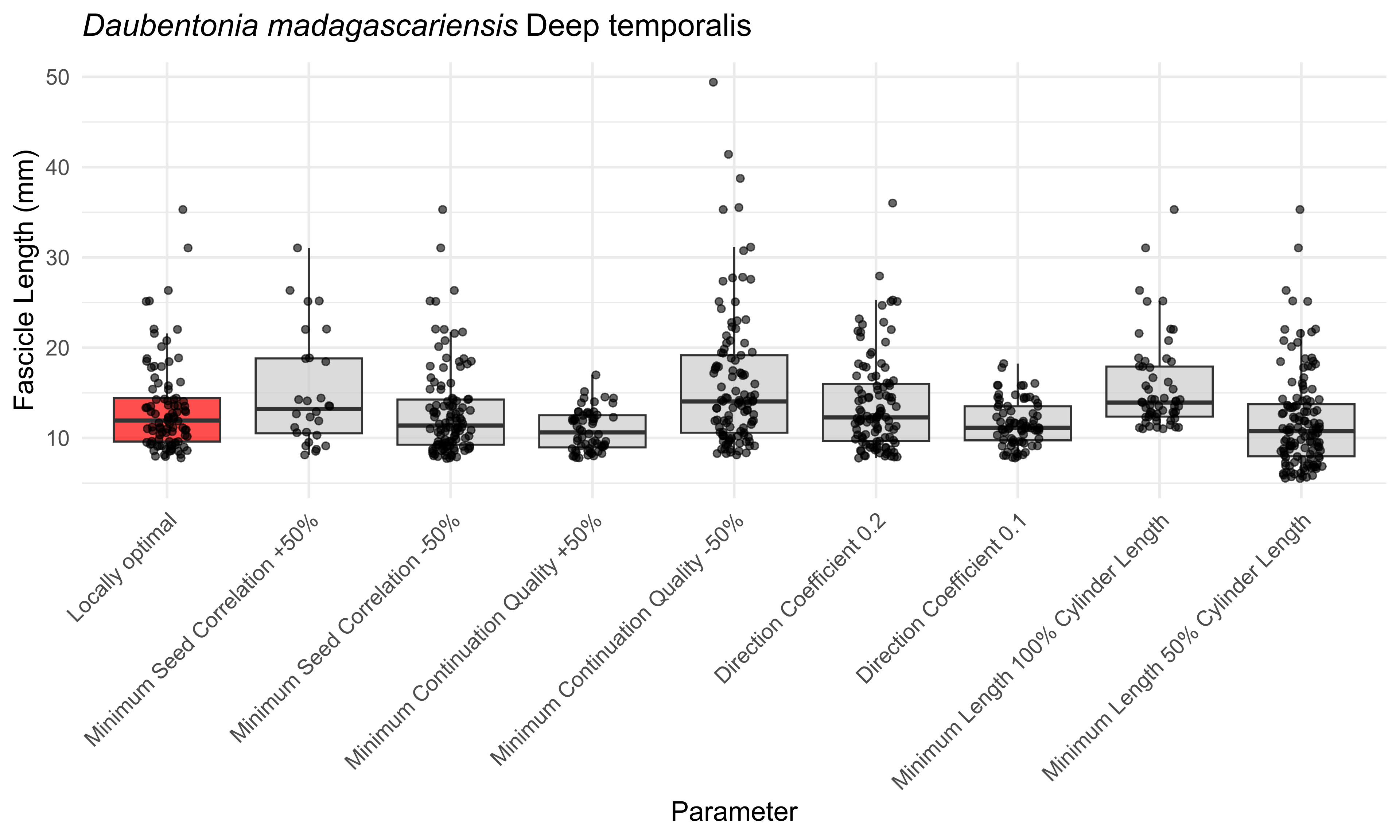

Daubentonia madagascariensis Deep temporalis
Fascicle Length (mm)
50
40
30
20
10
Locally optimal
Minimum Seed Correlation +50%
Minimum Seed Correlation -50%
Minimum Continuation Quality +50%
Minimum Continuation Quality -50%
Direction Coefficient 0.2
Direction Coefficient 0.1
Minimum Length 100% Cylinder Length
Minimum Length 50% Cylinder Length
Parameter

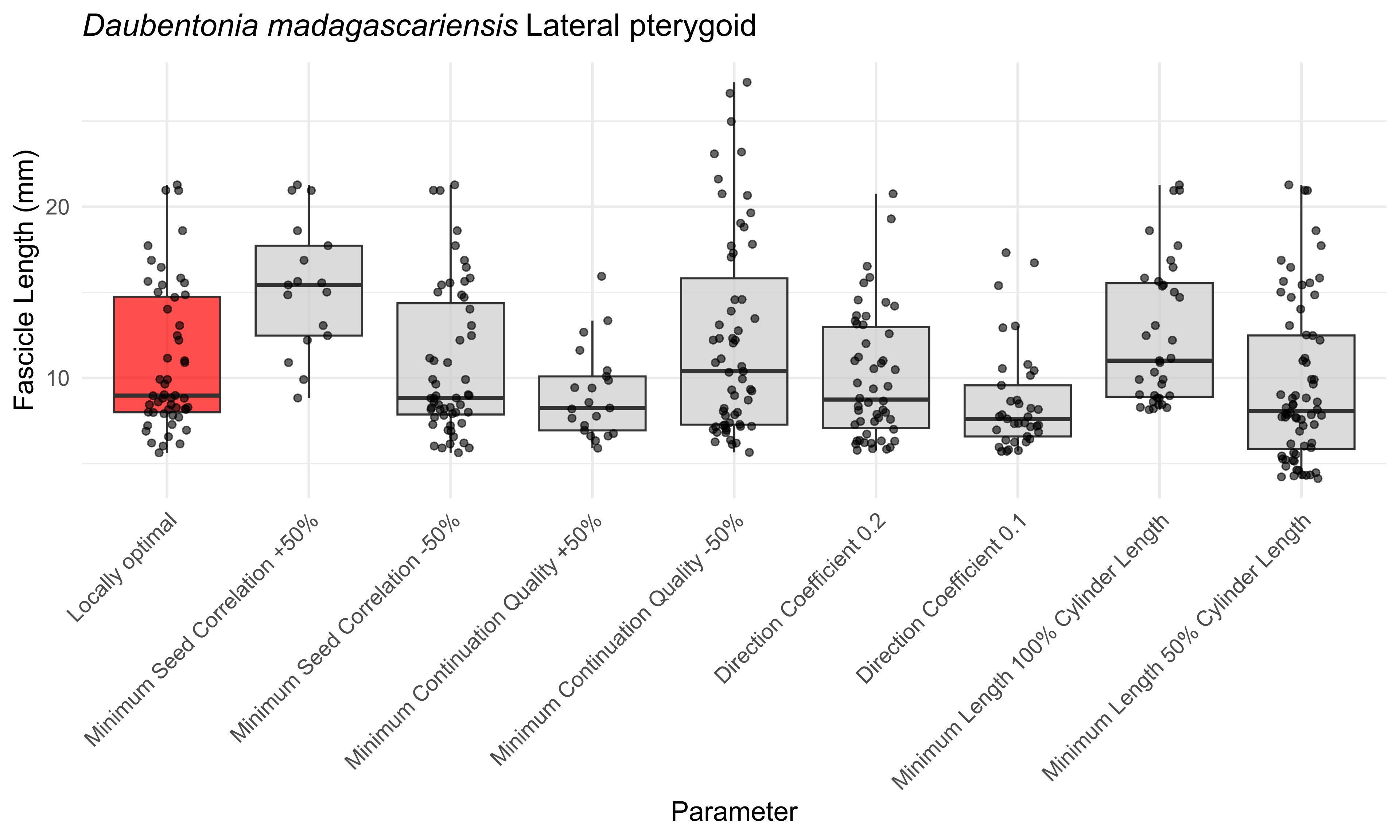

Daubentonia madagascariensis Lateral pterygoid
Fascicle Length (mm)
20
10
Locally optimal
Minimum Seed Correlation +50%
Minimum Seed Correlation -50%
Minimum Continuation Quality +50%
Minimum Continuation Quality -50%
Direction Coefficient 0.2
Direction Coefficient 0.1
Minimum Length 100% Cylinder Length
Minimum Length 50% Cylinder Length
Parameter

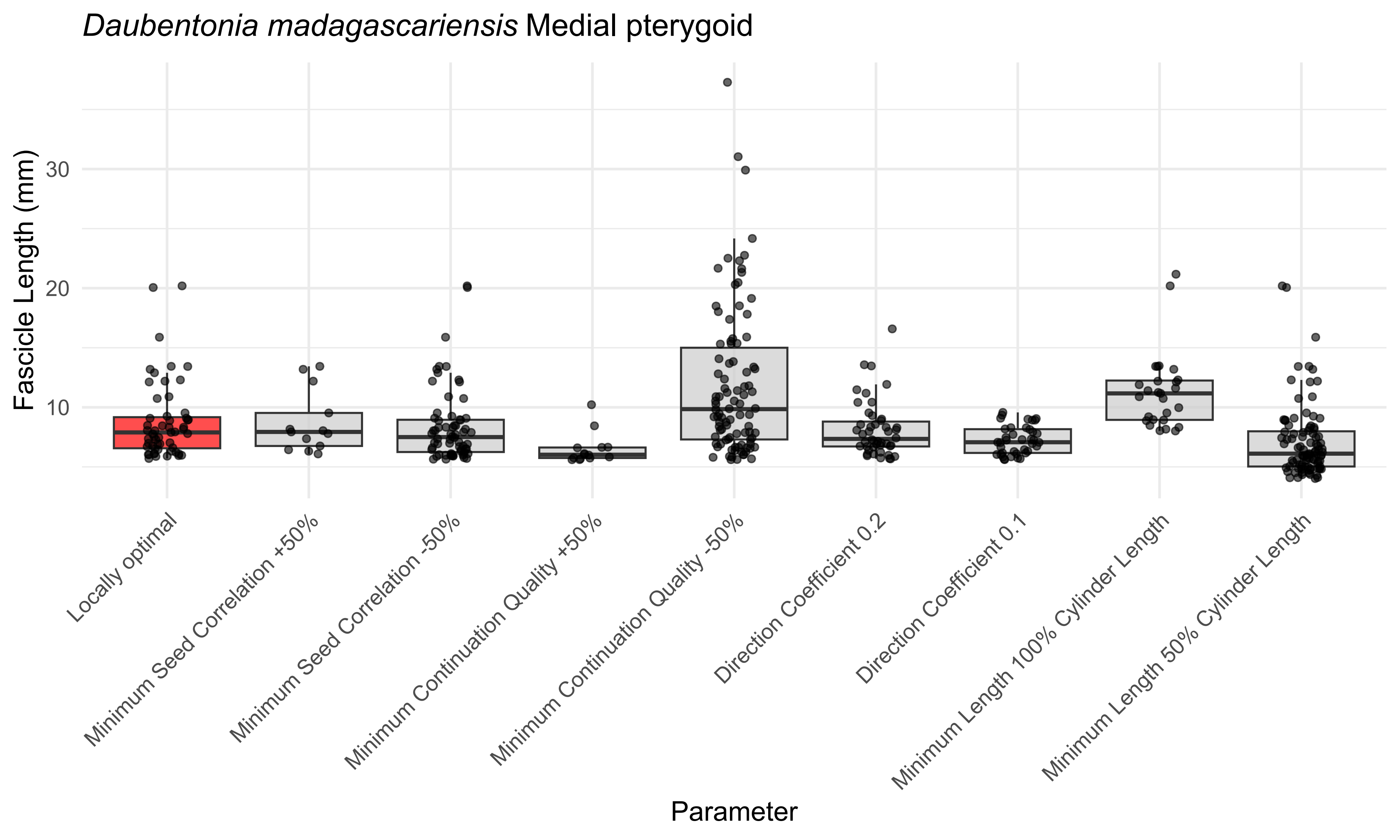

Daubentonia madagascariensis Medial pterygoid
Fascicle Length (mm)
30
20
10
Locally optimal
Minimum Seed Correlation +50%
Minimum Seed Correlation -50%
Minimum Continuation Quality +50%
Minimum Continuation Quality -50%
Direction Coefficient 0.2
Direction Coefficient 0.1
Minimum Length 100% Cylinder Length
Minimum Length 50% Cylinder Length
Parameter

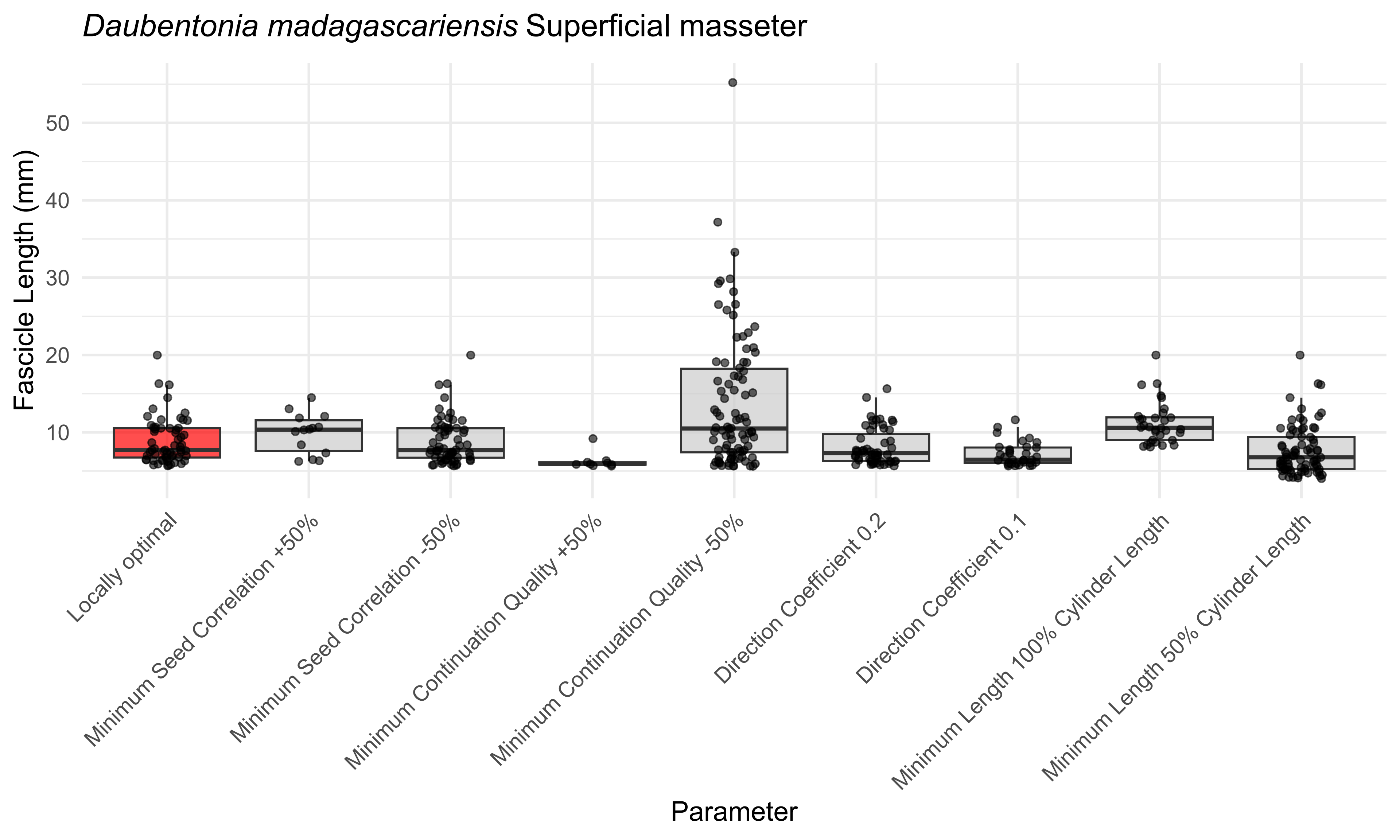
Daubentonia madagascariensis Superficial masseter
Fascicle Length (mm)
50
40
30
20
10
Locally optimal
Minimum Seed Correlation +50%
Minimum Seed Correlation -50%
Minimum Continuation Quality +50%
Minimum Continuation Quality -50%
Direction Coefficient 0.2
Direction Coefficient 0.1
Minimum Length 100% Cylinder Length
Minimum Length 50% Cylinder Length
Parameter

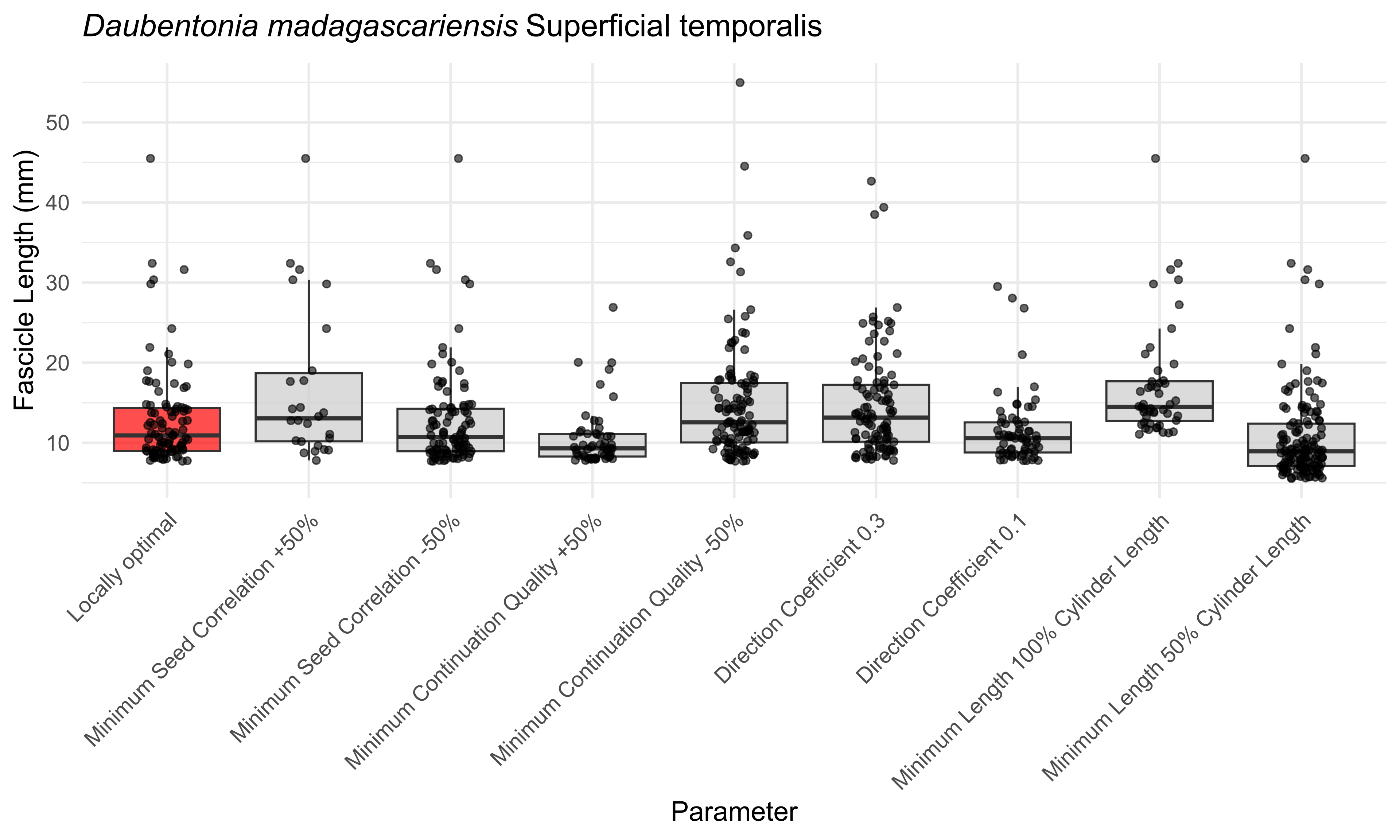

Daubentonia madagascariensis Superficial temporalis
Fascicle Length (mm)
10
20
30
40
50
Locally optimal
Minimum Seed Correlation +50%
Minimum Seed Correlation -50%
Minimum Continuation Quality +50%
Minimum Continuation Quality -50%
Direction Coefficient 0.3
Direction Coefficient 0.1
Minimum Length 100% Cylinder Length
Minimum Length 50% Cylinder Length
Parameter

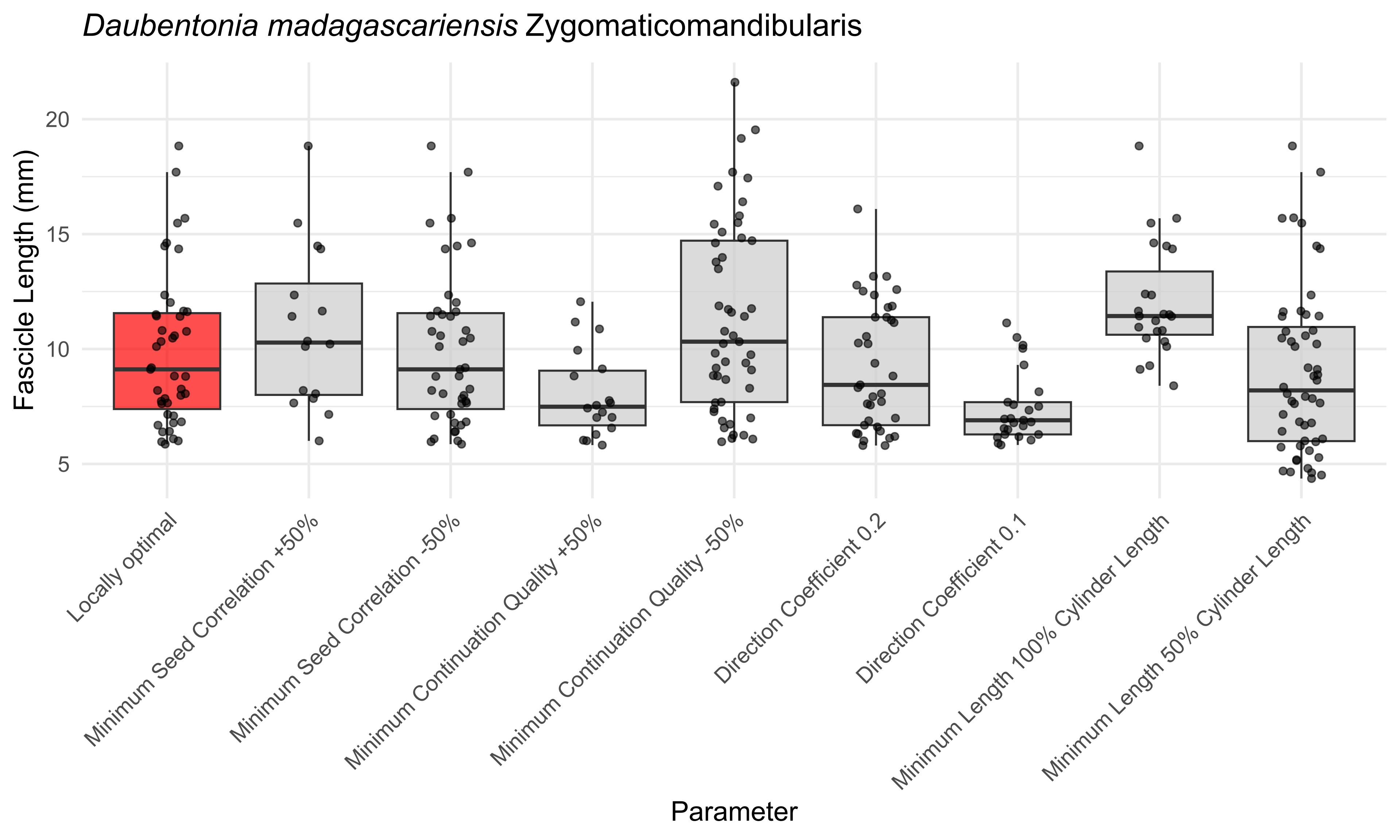

Daubentonia madagascariensis Zygomaticomandibularis
Fascicle Length (mm)
20
15
10
5
Locally optimal
Minimum Seed Correlation +50%
Minimum Seed Correlation -50%
Minimum Continuation Quality +50%
Minimum Continuation Quality -50%
Direction Coefficient 0.2
Direction Coefficient 0.1
Minimum Length 100% Cylinder Length
Minimum Length 50% Cylinder Length
Parameter

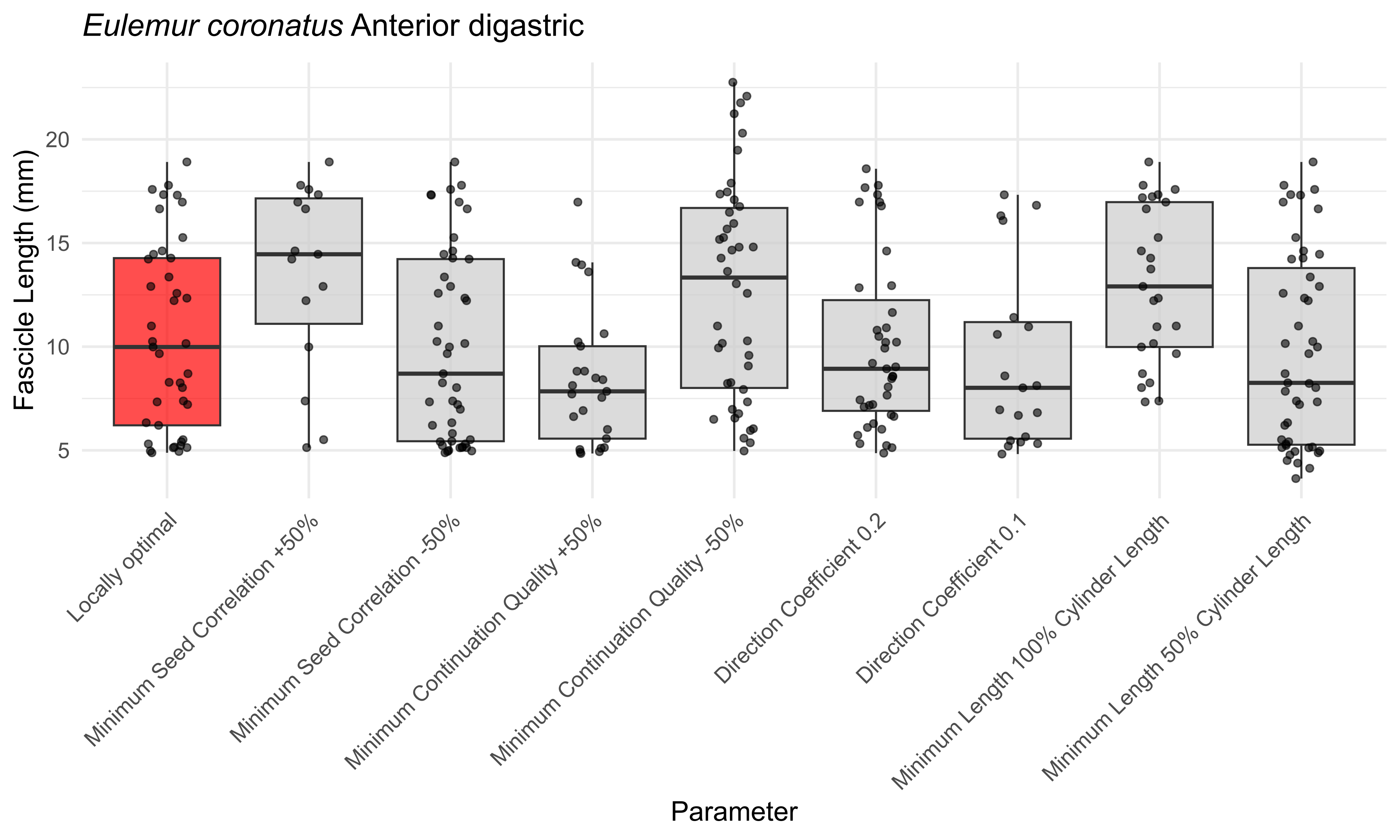

Eulemur coronatus Anterior digastric
Fascicle Length (mm)
20
15
10
5
Locally optimal
Minimum Seed Correlation +50%
Minimum Seed Correlation -50%
Minimum Continuation Quality +50%
Minimum Continuation Quality -50%
Direction Coefficient 0.2
Direction Coefficient 0.1
Minimum Length 100% Cylinder Length
Minimum Length 50% Cylinder Length
Parameter

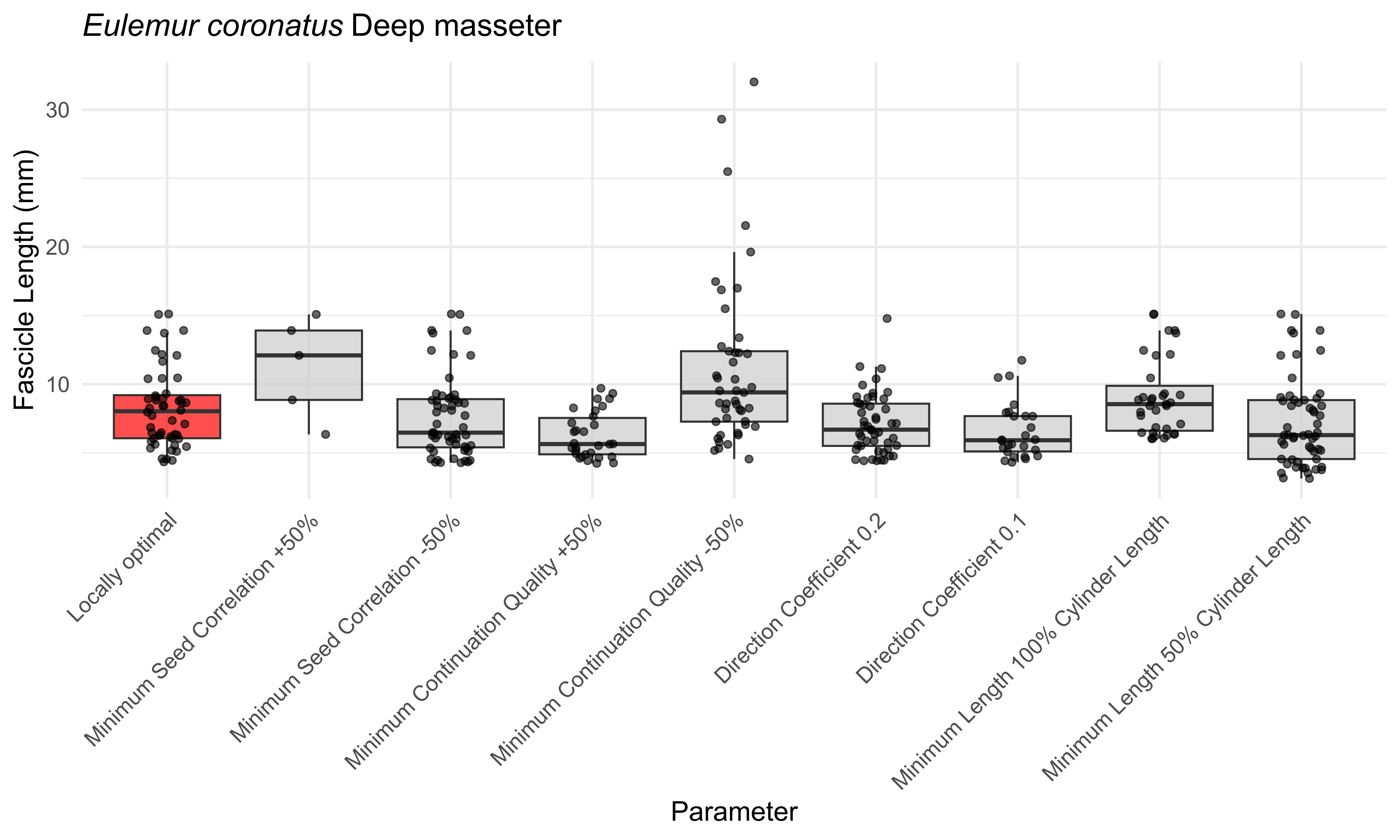
Eulemur coronatus Deep masseter
Fascicle Length (mm)
30
20
10
Locally optimal
Minimum Seed Correlation +50%
Minimum Seed Correlation -50%
Minimum Continuation Quality +50%
Minimum Continuation Quality -50%
Direction Coefficient 0.2
Direction Coefficient 0.1
Minimum Length 100% Cylinder Length
Minimum Length 50% Cylinder Length
Parameter

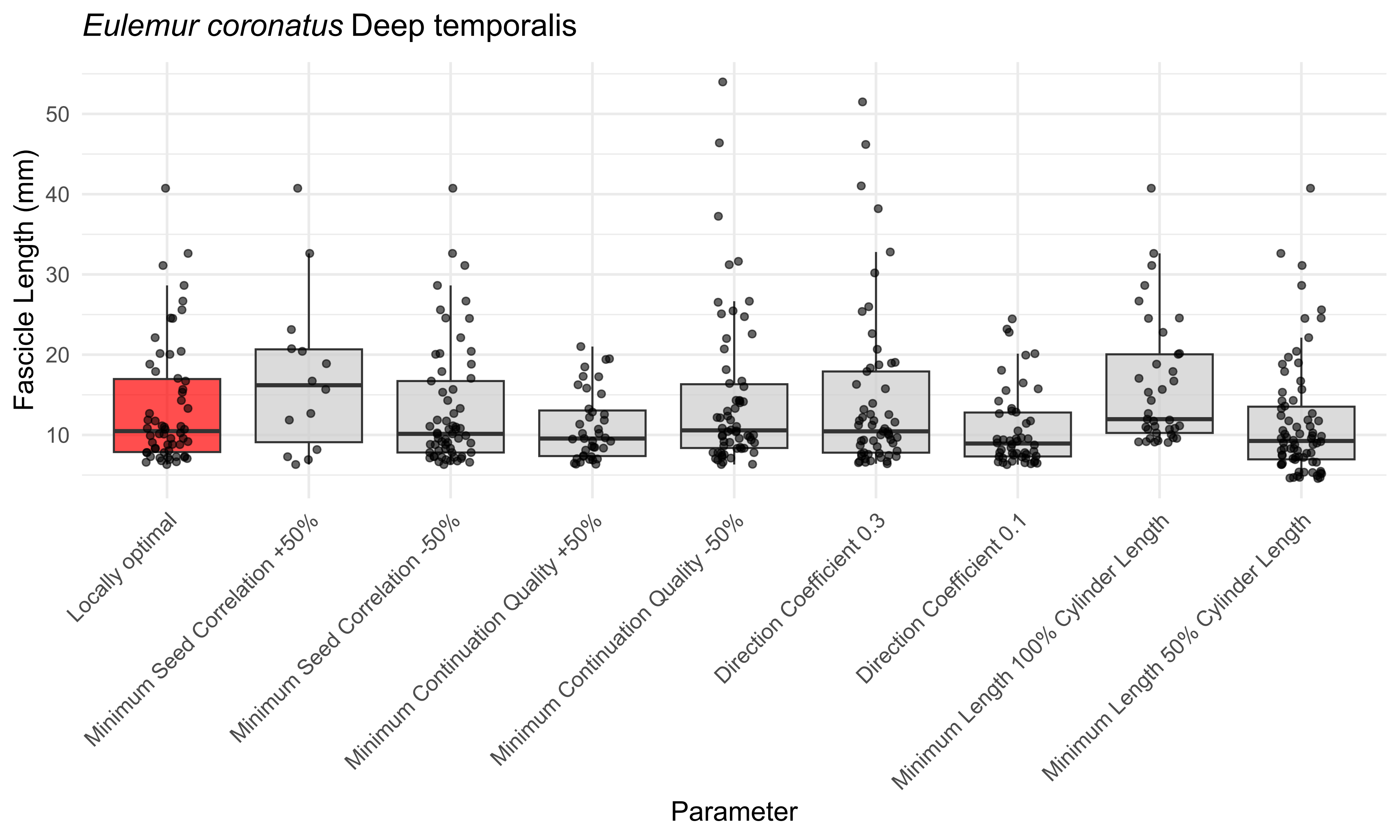

Eulemur coronatus Deep temporalis
Fascicle Length (mm)
10
20
30
40
50
Locally optimal
Minimum Seed Correlation +50%
Minimum Seed Correlation -50%
Minimum Continuation Quality +50%
Minimum Continuation Quality -50%
Direction Coefficient 0.3
Direction Coefficient 0.1
Minimum Length 100% Cylinder Length
Minimum Length 50% Cylinder Length
Parameter

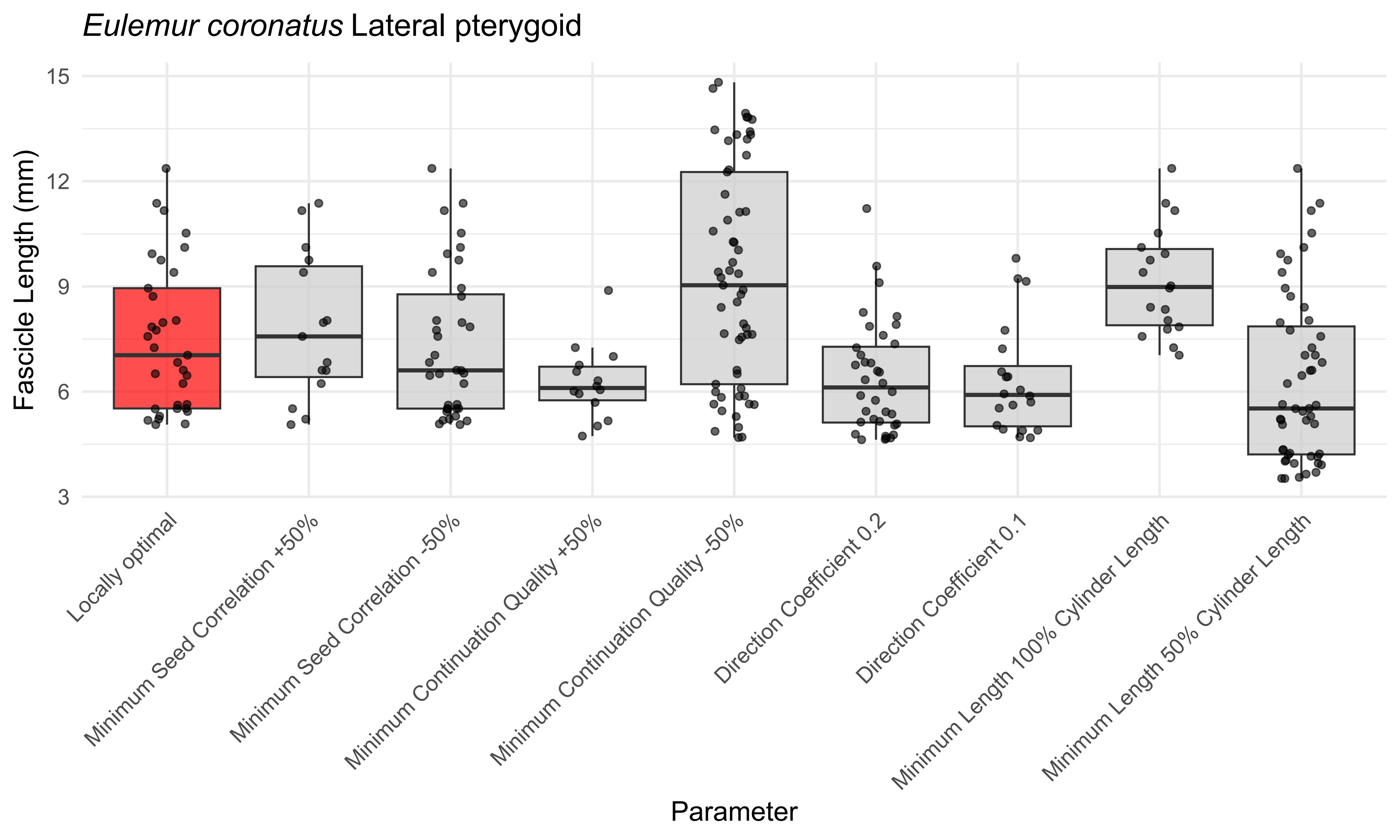

Eulemur coronatus Lateral pterygoid
Fascicle Length (mm)
15
12
9
6
3
Locally optimal
Minimum Seed Correlation +50%
Minimum Seed Correlation -50%
Minimum Continuation Quality +50%
Minimum Continuation Quality -50%
Direction Coefficient 0.2
Direction Coefficient 0.1
Minimum Length 100% Cylinder Length
Minimum Length 50% Cylinder Length
Parameter

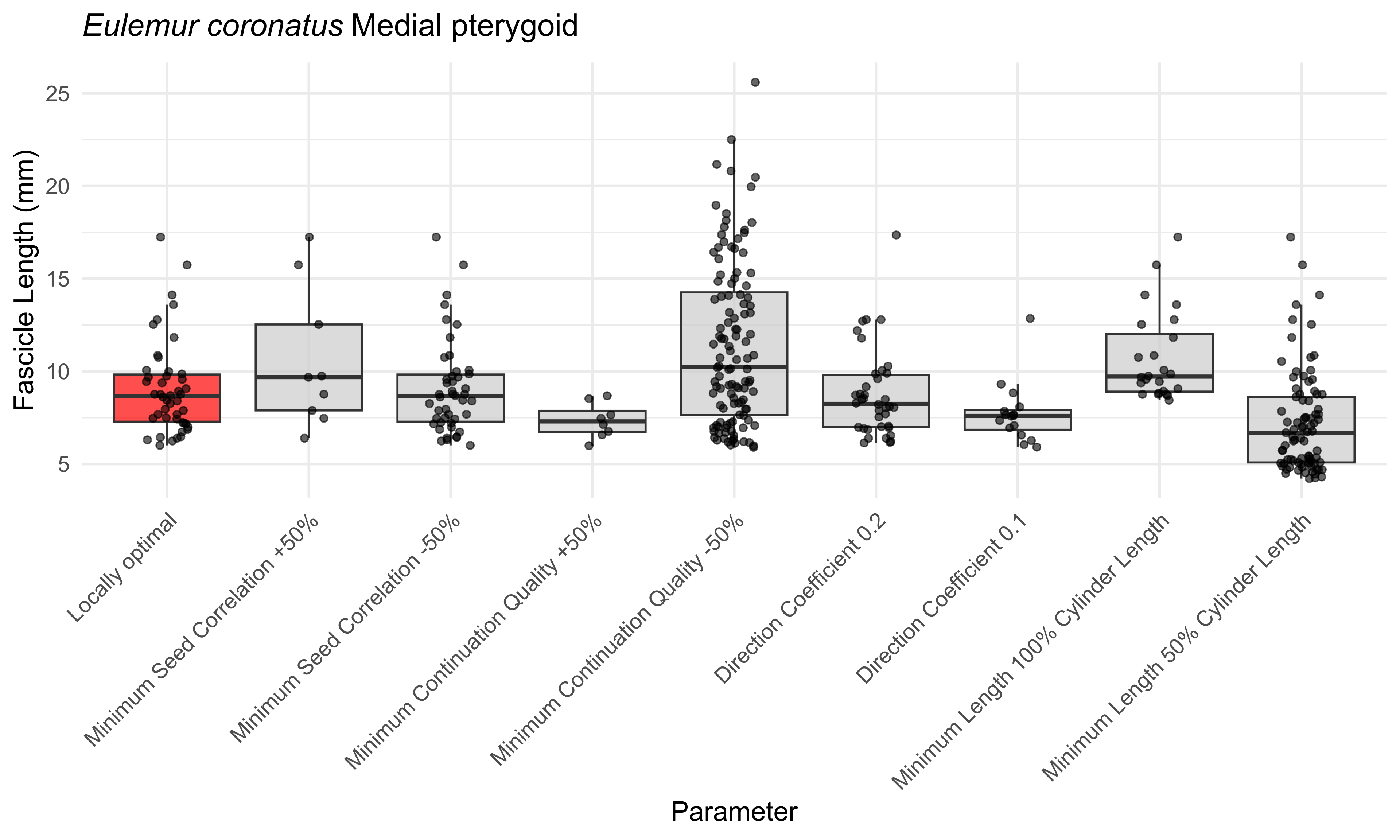

Eulemur coronatus Medial pterygoid
Fascicle Length (mm)
25
20
15
10
5
Locally optimal
Minimum Seed Correlation +50%
Minimum Seed Correlation -50%
Minimum Continuation Quality +50%
Minimum Continuation Quality -50%
Direction Coefficient 0.2
Direction Coefficient 0.1
Minimum Length 100% Cylinder Length
Minimum Length 50% Cylinder Length
Parameter

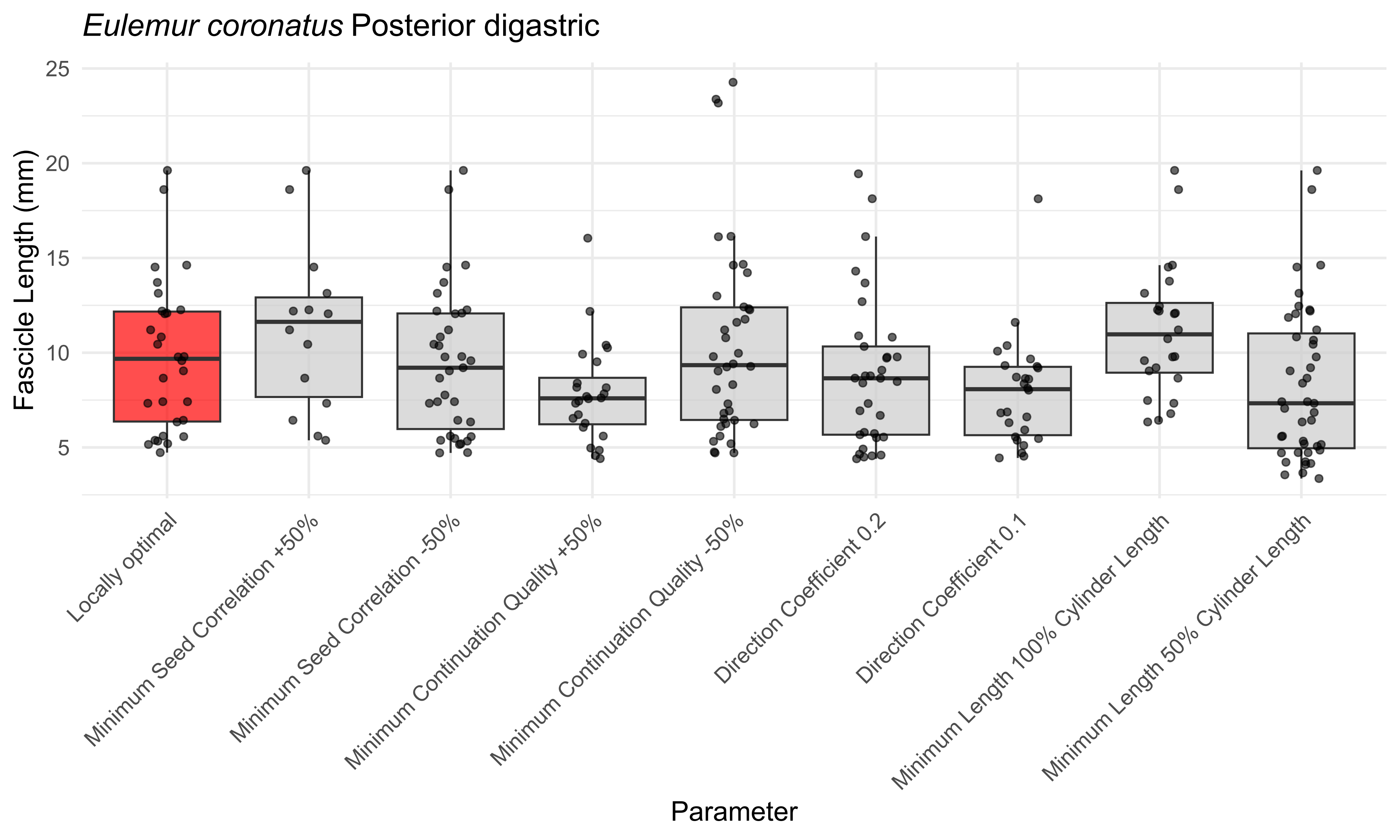

Eulemur coronatus Posterior digastric
Fascicle Length (mm)
25
20
15
10
5
Locally optimal
Minimum Seed Correlation +50%
Minimum Seed Correlation -50%
Minimum Continuation Quality +50%
Minimum Continuation Quality -50%
Direction Coefficient 0.2
Direction Coefficient 0.1
Minimum Length 100% Cylinder Length
Minimum Length 50% Cylinder Length
Parameter

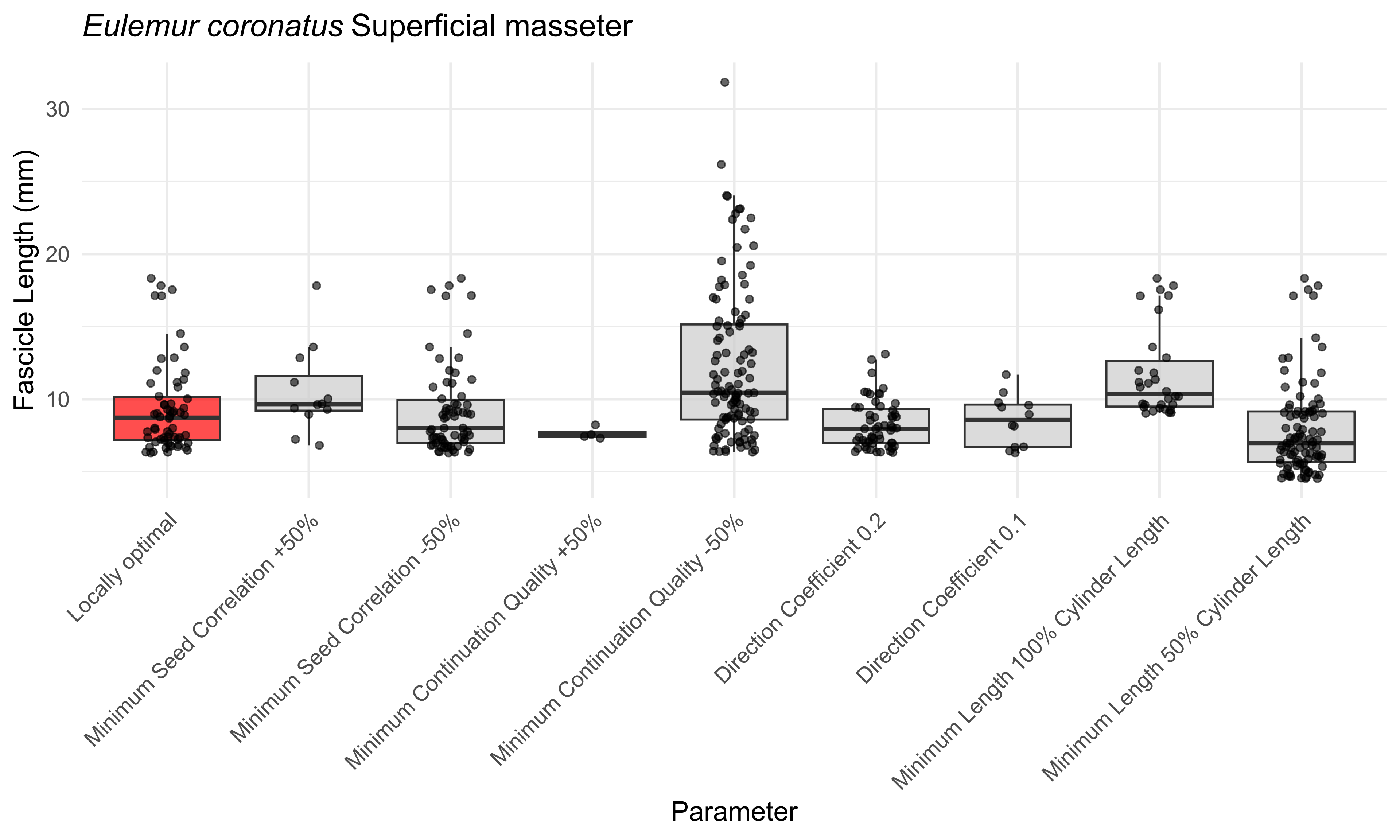

Eulemur coronatus Superficial masseter
Fascicle Length (mm)
30
20
10
Locally optimal
Minimum Seed Correlation +50%
Minimum Seed Correlation -50%
Minimum Continuation Quality +50%
Minimum Continuation Quality -50%
Direction Coefficient 0.2
Direction Coefficient 0.1
Minimum Length 100% Cylinder Length
Minimum Length 50% Cylinder Length
Parameter

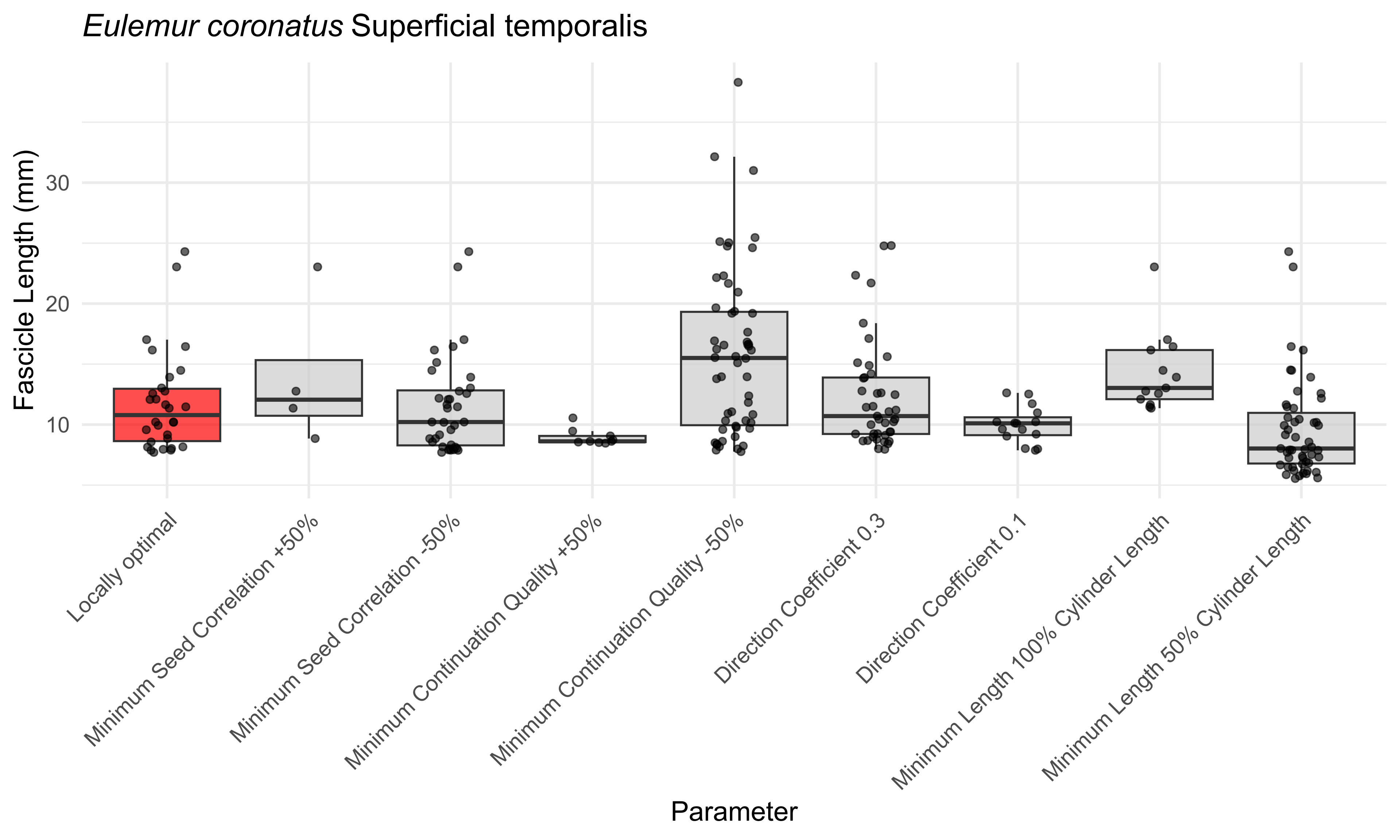
Eulemur coronatus Superficial temporalis
Fascicle Length (mm)
10
20
30
Locally optimal
Minimum Seed Correlation +50%
Minimum Seed Correlation -50%
Minimum Continuation Quality +50%
Minimum Continuation Quality -50%
Direction Coefficient 0.3
Direction Coefficient 0.1
Minimum Length 100% Cylinder Length
Minimum Length 50% Cylinder Length
Parameter

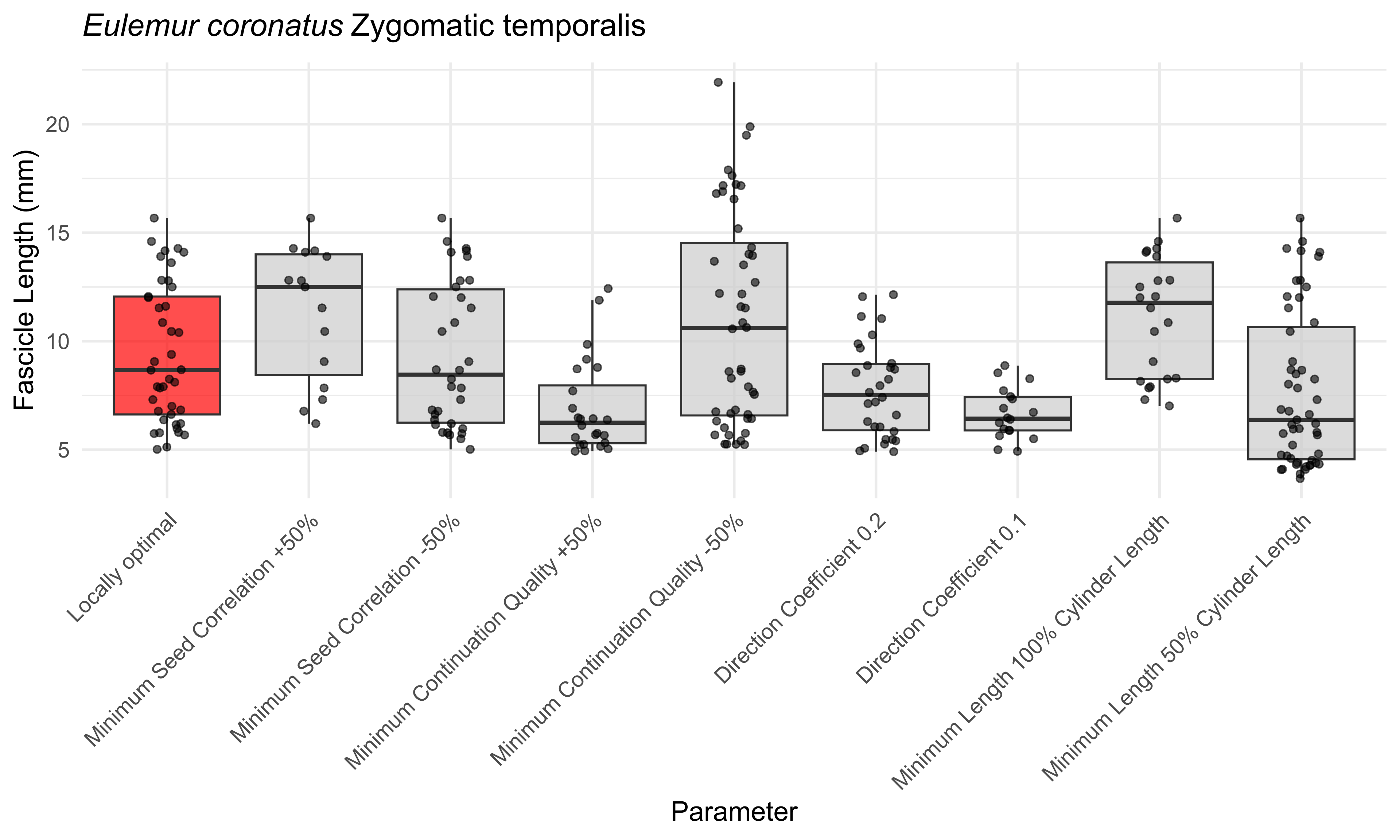

Eulemur coronatus Zygomatic temporalis
Fascicle Length (mm)
20
15
10
5
Locally optimal
Minimum Seed Correlation +50%
Minimum Seed Correlation -50%
Minimum Continuation Quality +50%
Minimum Continuation Quality -50%
Direction Coefficient 0.2
Direction Coefficient 0.1
Minimum Length 100% Cylinder Length
Minimum Length 50% Cylinder Length
Parameter

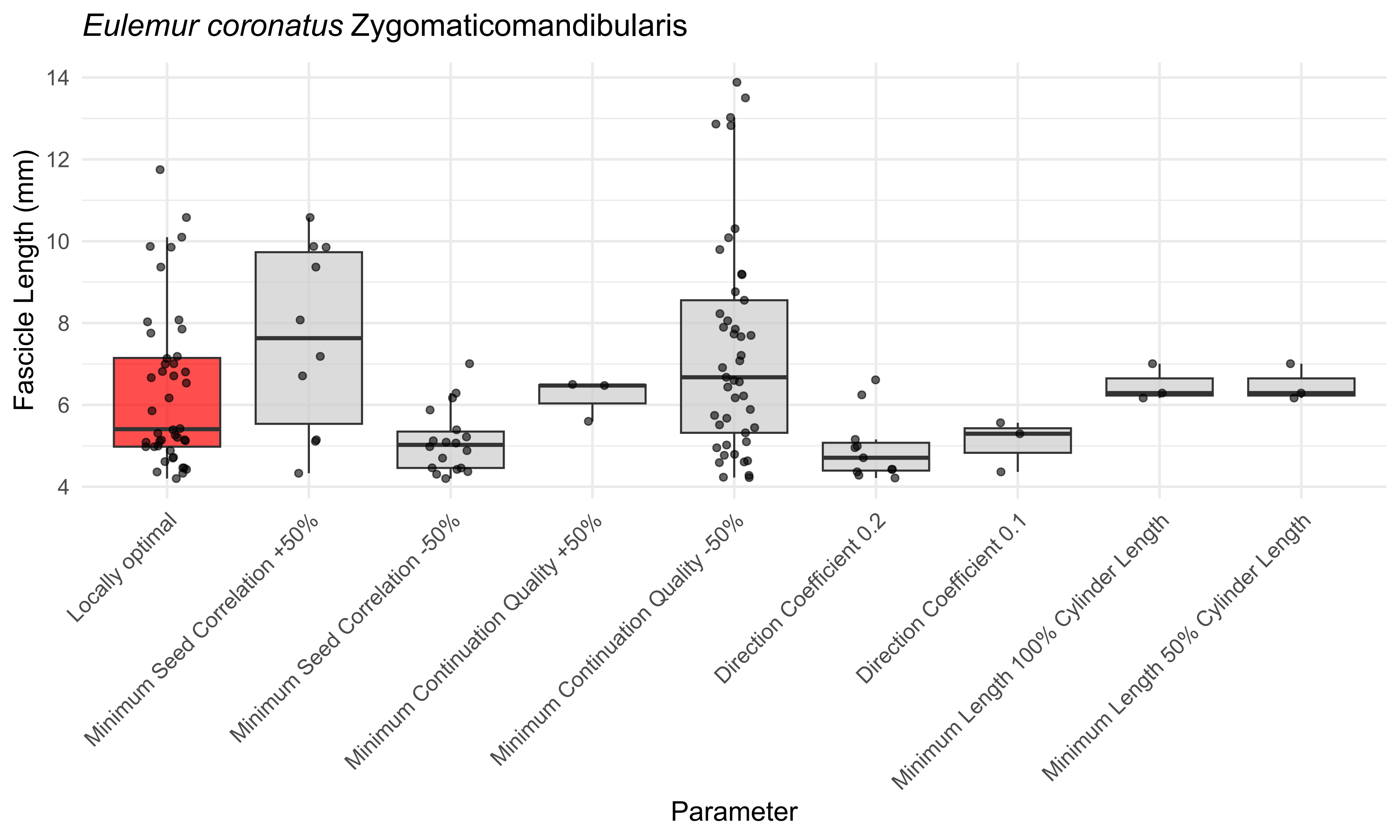

Eulemur coronatus Zygomaticomandibularis
Fascicle Length (mm)
14
12
10
8
6
4
Locally optimal
Minimum Seed Correlation +50%
Minimum Seed Correlation -50%
Minimum Continuation Quality +50%
Minimum Continuation Quality -50%
Direction Coefficient 0.2
Direction Coefficient 0.1
Minimum Length 100% Cylinder Length
Minimum Length 50% Cylinder Length
Parameter

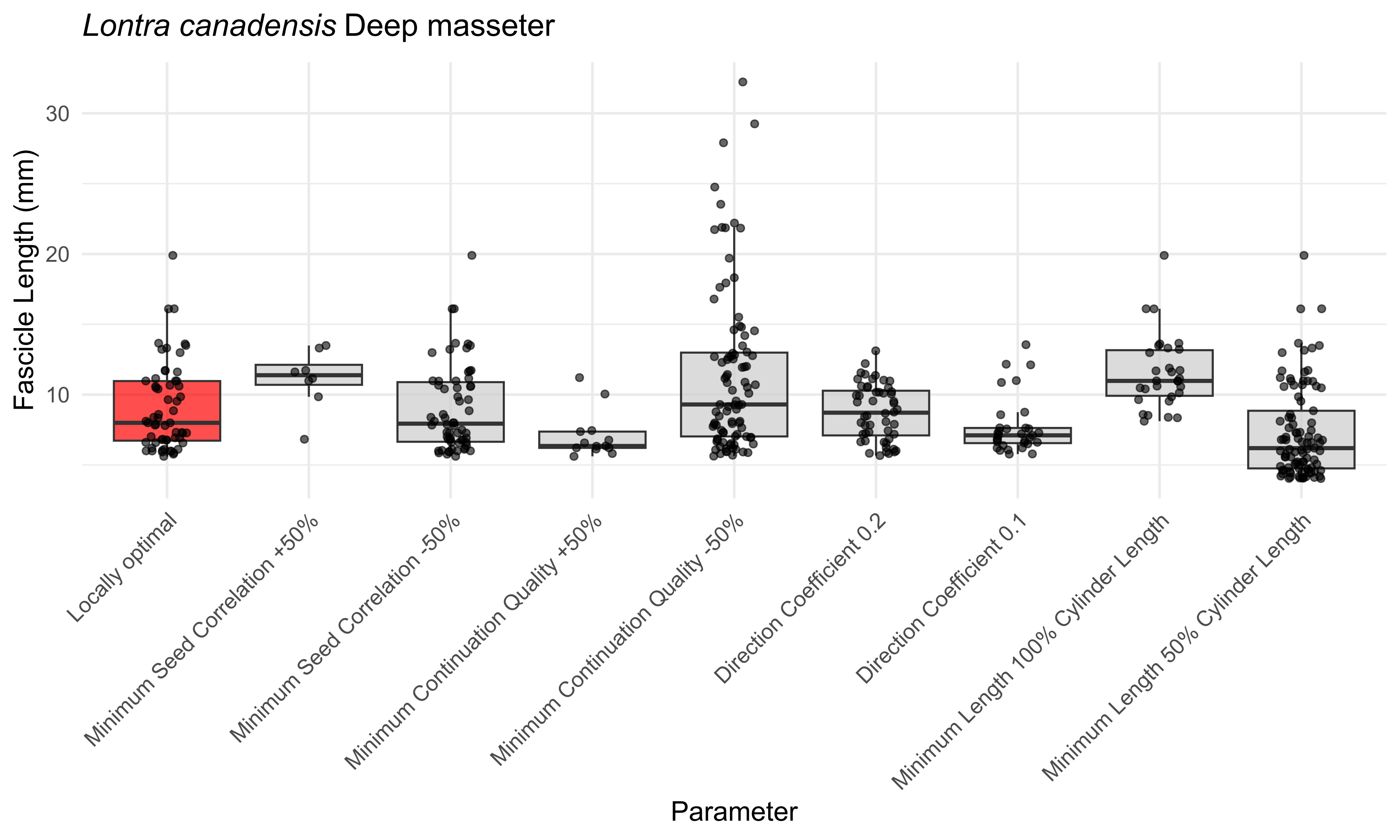

Lontra canadensis Deep masseter
Fascicle Length (mm)
30
20
10
Locally optimal
Minimum Seed Correlation +50%
Minimum Seed Correlation -50%
Minimum Continuation Quality +50%
Minimum Continuation Quality -50%
Direction Coefficient 0.2
Direction Coefficient 0.1
Minimum Length 100% Cylinder Length
Minimum Length 50% Cylinder Length
Parameter

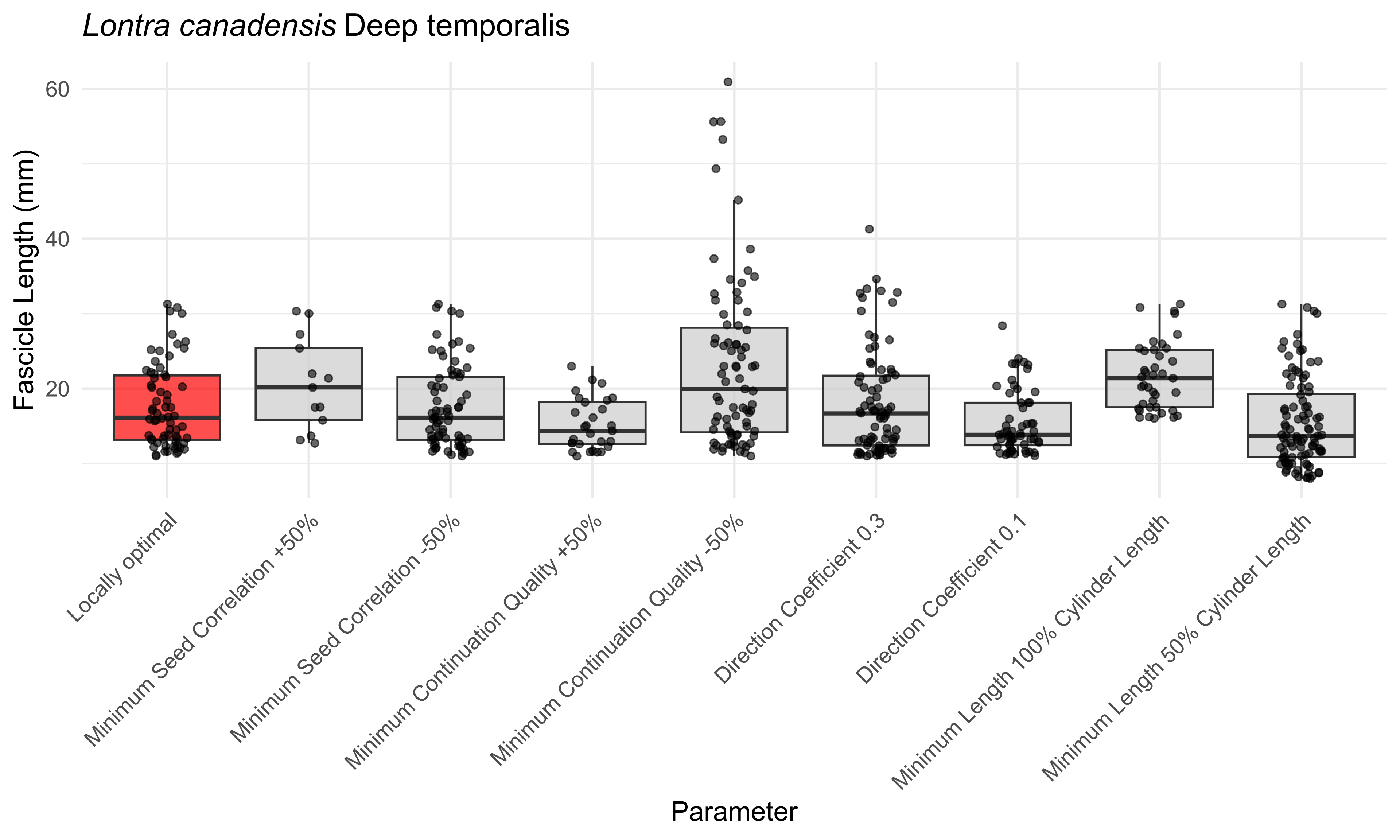

Lontra canadensis Deep temporalis
Fascicle Length (mm)
60
40
20
Locally optimal
Minimum Seed Correlation +50%
Minimum Seed Correlation -50%
Minimum Continuation Quality +50%
Minimum Continuation Quality -50%
Direction Coefficient 0.3
Direction Coefficient 0.1
Minimum Length 100% Cylinder Length
Minimum Length 50% Cylinder Length
Parameter

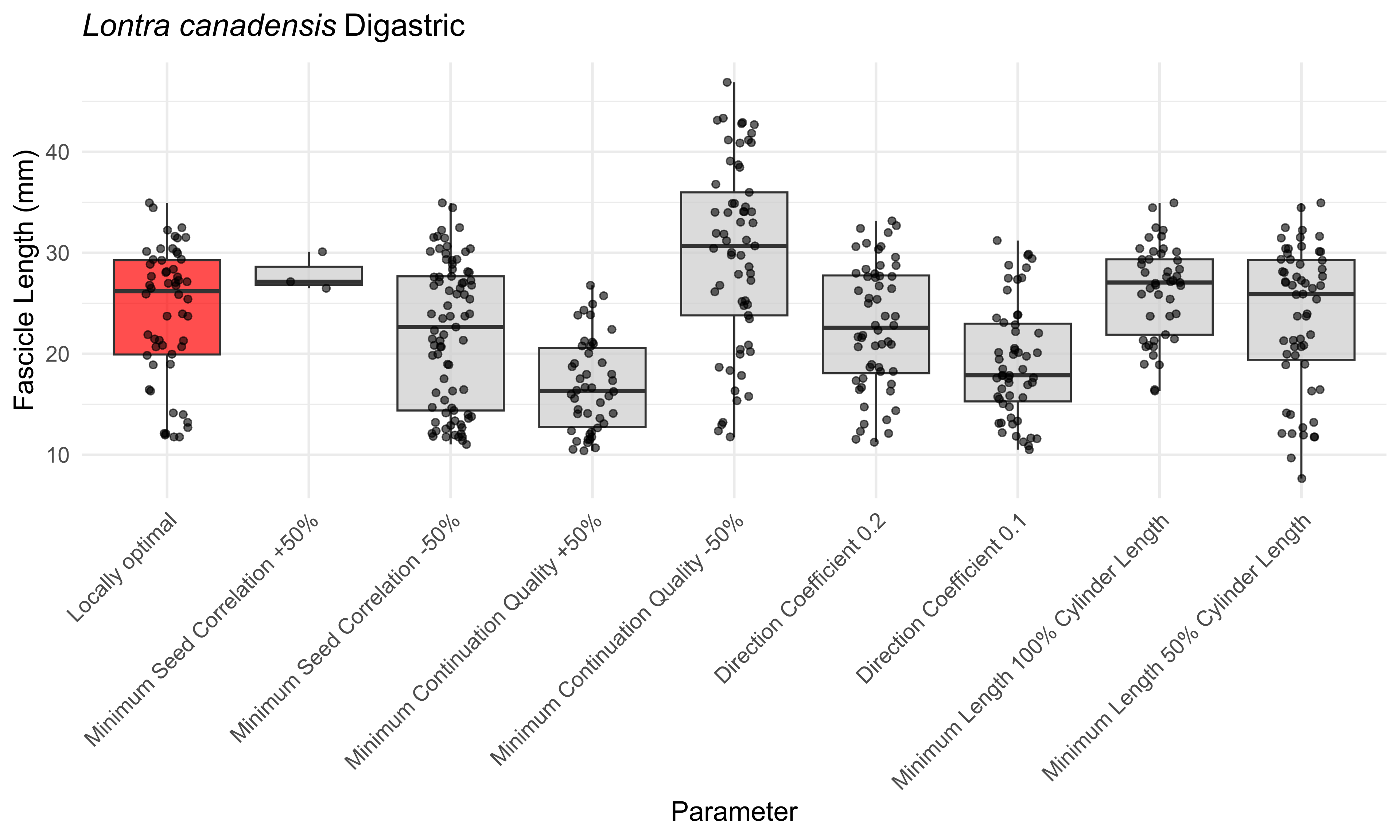

Lontra canadensis Digastric
Fascicle Length (mm)
10
20
30
40
Locally optimal
Minimum Seed Correlation +50%
Minimum Seed Correlation -50%
Minimum Continuation Quality +50%
Minimum Continuation Quality -50%
Direction Coefficient 0.2
Direction Coefficient 0.1
Minimum Length 100% Cylinder Length
Minimum Length 50% Cylinder Length
Parameter

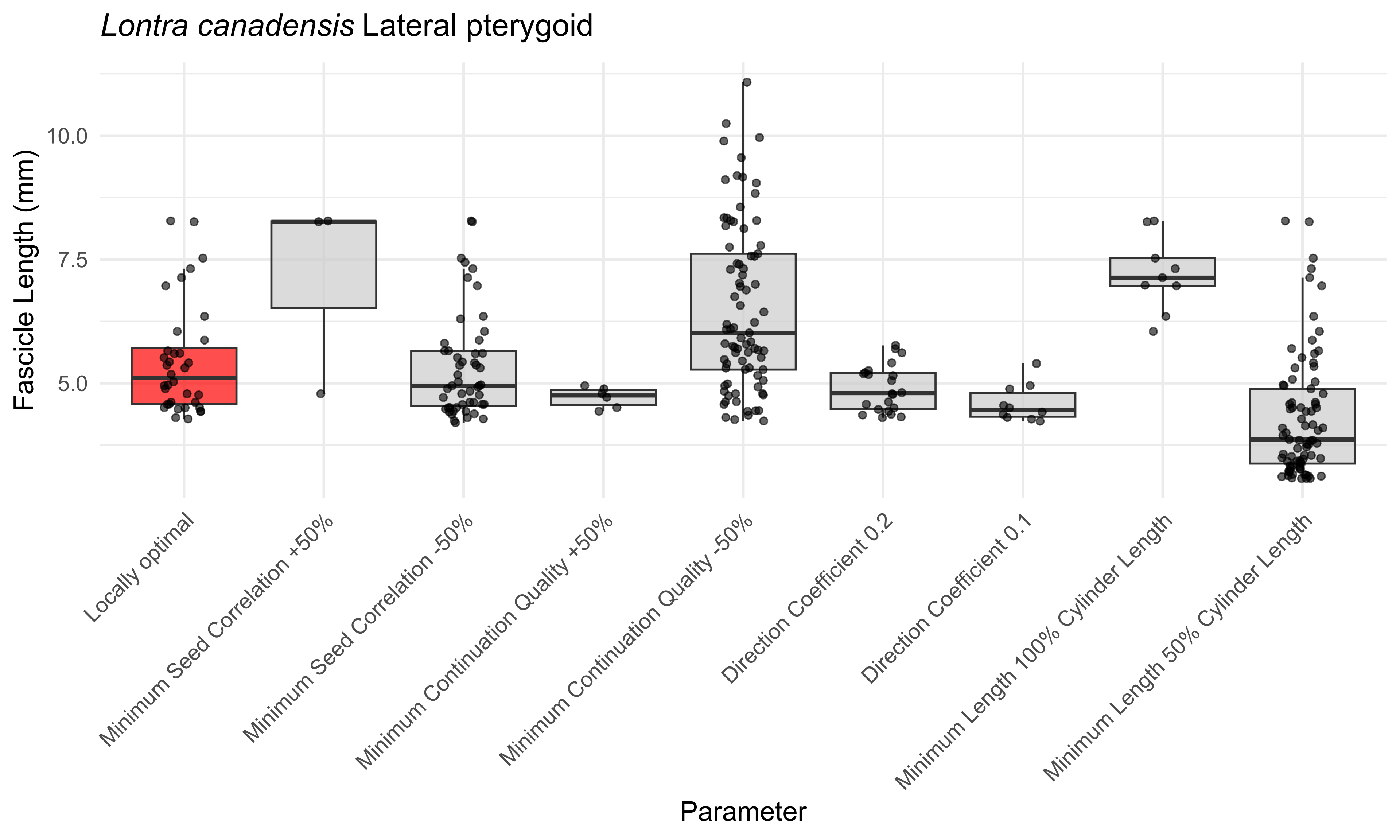
Lontra canadensis Lateral pterygoid
Fascicle Length (mm)
10.0
7.5
5.0
Locally optimal
Minimum Seed Correlation +50%
Minimum Seed Correlation -50%
Minimum Continuation Quality +50%
Minimum Continuation Quality -50%
Direction Coefficient 0.2
Direction Coefficient 0.1
Minimum Length 100% Cylinder Length
Minimum Length 50% Cylinder Length
Parameter

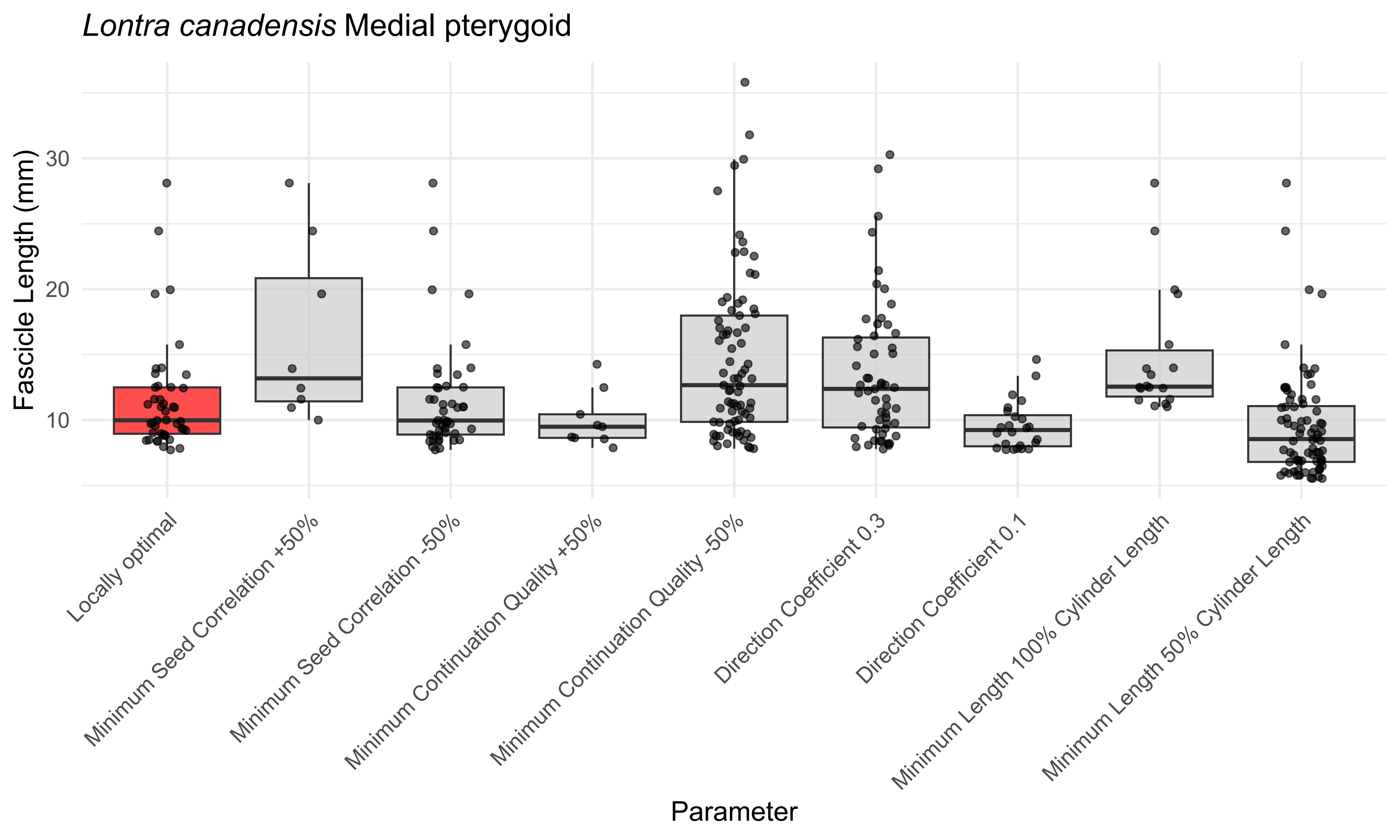
Lontra canadensis Medial pterygoid
Fascicle Length (mm)
30
20
10
Locally optimal
Minimum Seed Correlation +50%
Minimum Seed Correlation -50%
Minimum Continuation Quality +50%
Minimum Continuation Quality -50%
Direction Coefficient 0.3
Direction Coefficient 0.1
Minimum Length 100% Cylinder Length
Minimum Length 50% Cylinder Length
Parameter

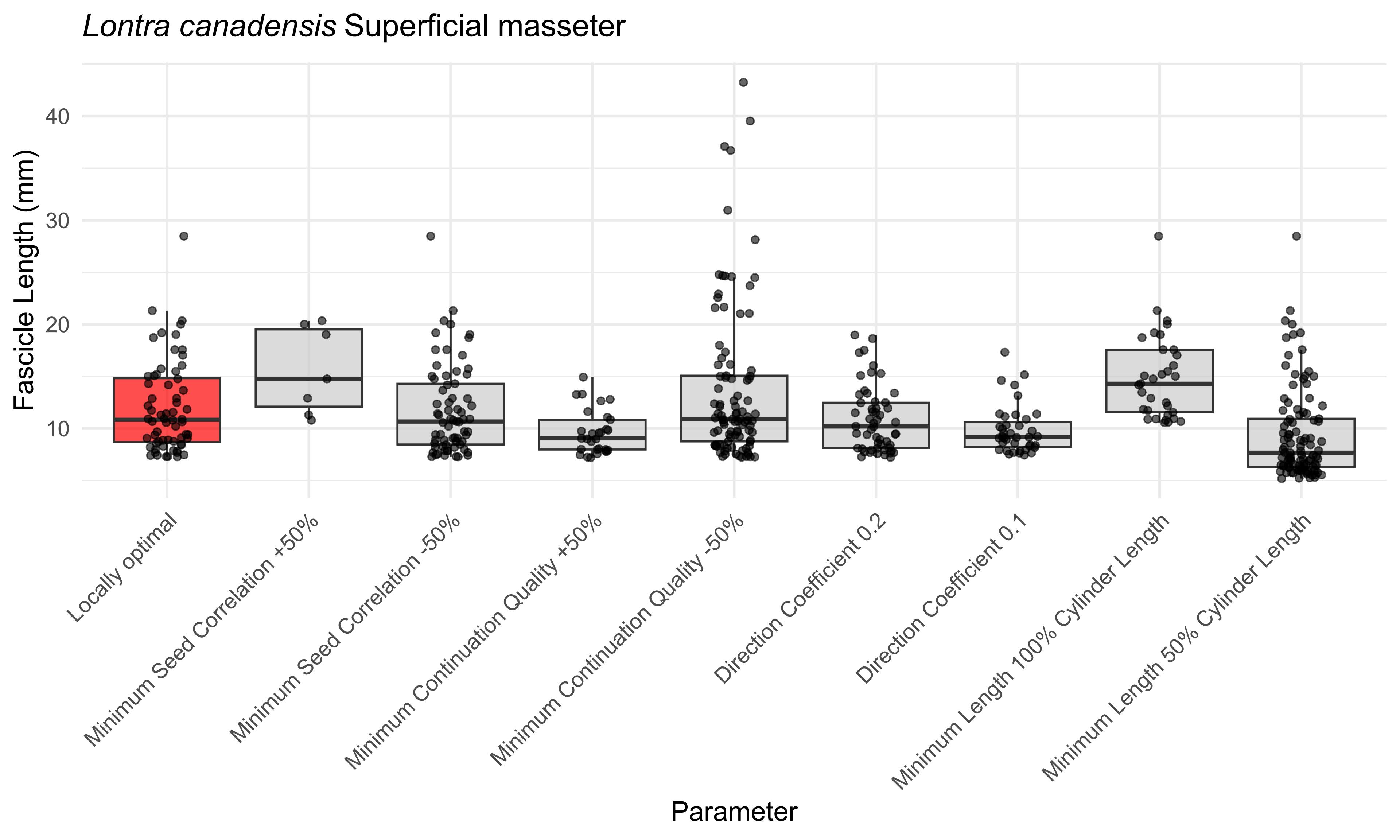

Lontra canadensis Superficial masseter
Fascicle Length (mm)
40
30
20
10
Locally optimal
Minimum Seed Correlation +50%
Minimum Seed Correlation -50%
Minimum Continuation Quality +50%
Minimum Continuation Quality -50%
Direction Coefficient 0.2
Direction Coefficient 0.1
Minimum Length 100% Cylinder Length
Minimum Length 50% Cylinder Length
Parameter

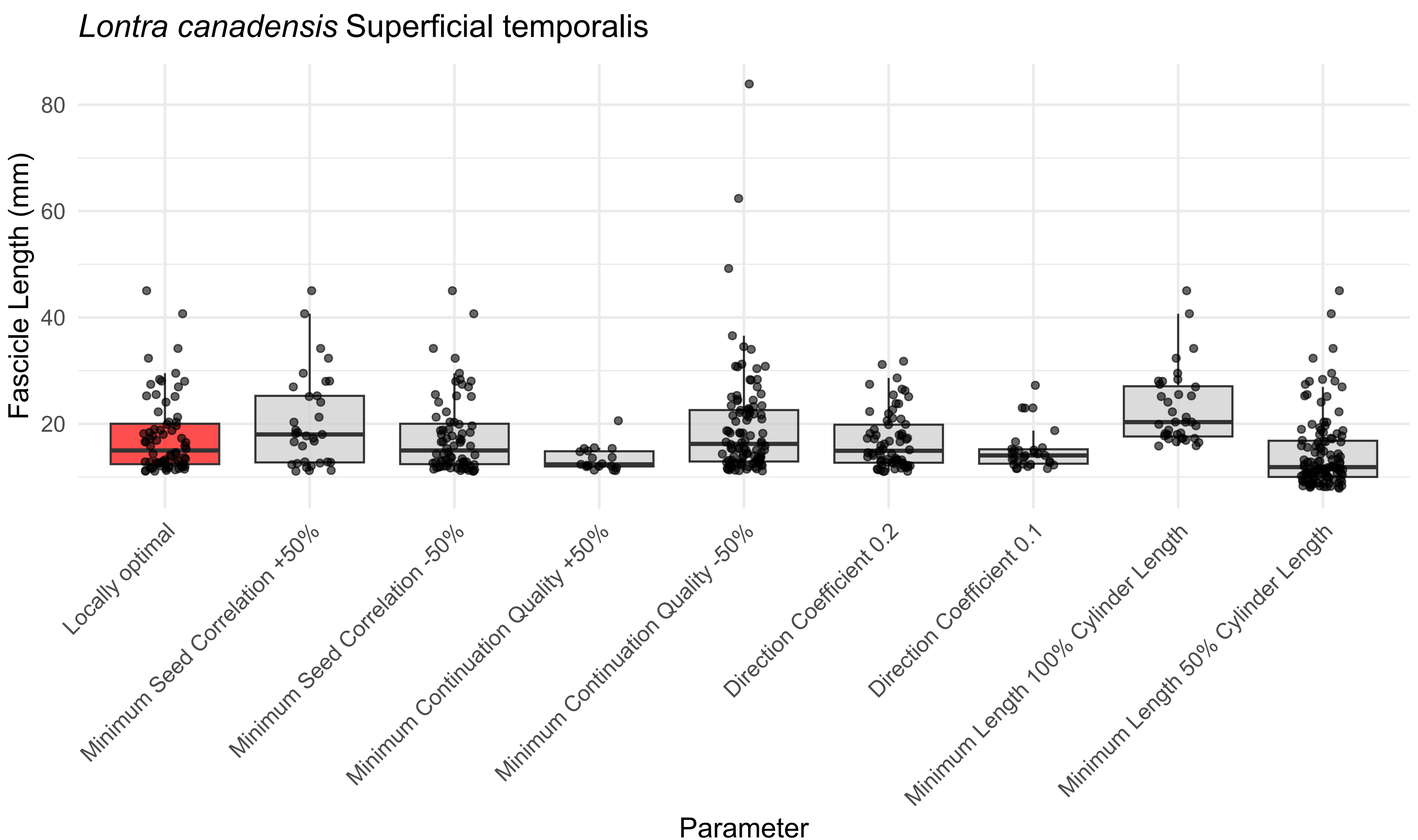
Lontra canadensis Superficial temporalis
Fascicle Length (mm)
80
60
40
20
Locally optimal
Minimum Seed Correlation +50%
Minimum Seed Correlation -50%
Minimum Continuation Quality +50%
Minimum Continuation Quality -50%
Direction Coefficient 0.2
Direction Coefficient 0.1
Minimum Length 100% Cylinder Length
Minimum Length 50% Cylinder Length
Parameter

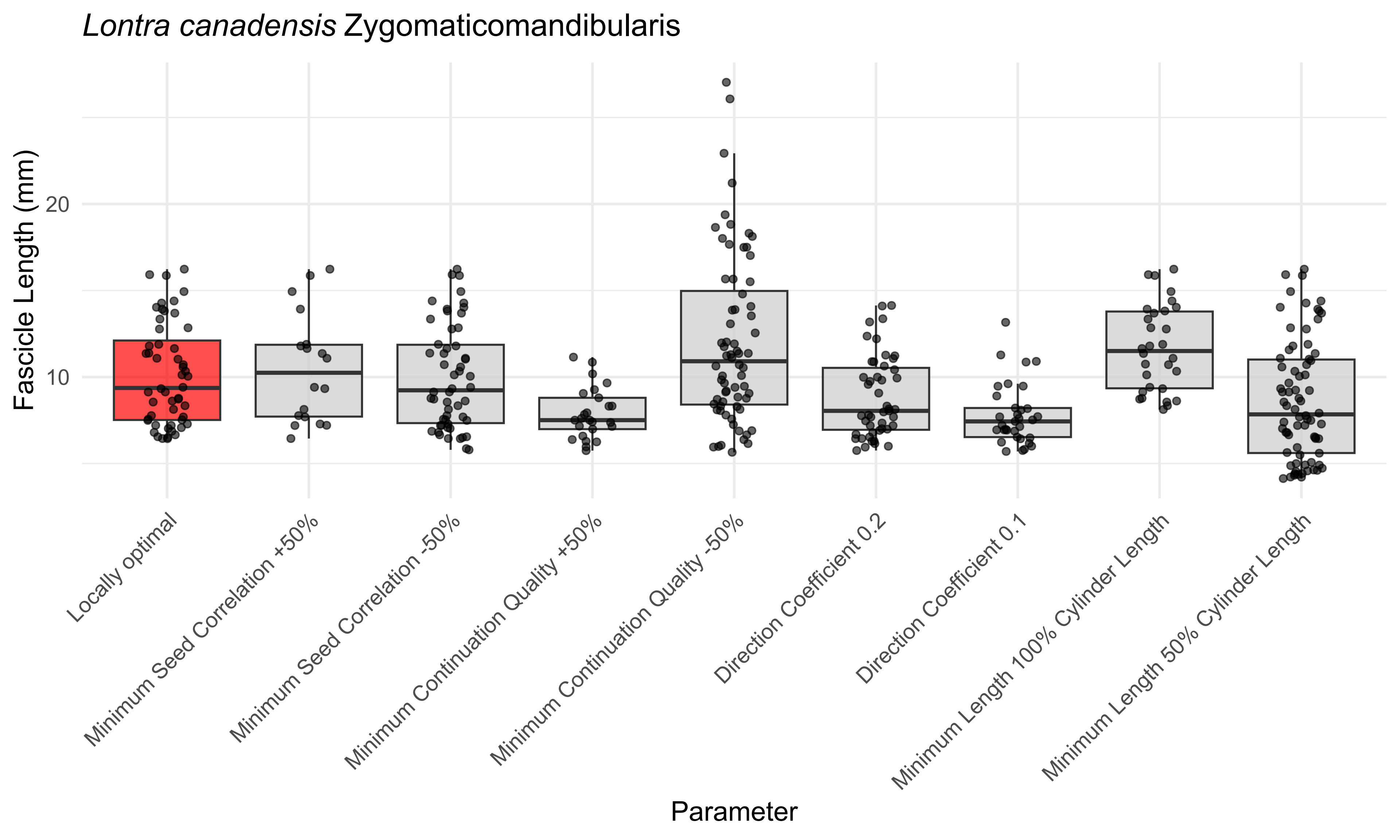

Lontra canadensis Zygomaticomandibularis
Fascicle Length (mm)
20
10
Locally optimal
Minimum Seed Correlation +50%
Minimum Seed Correlation -50%
Minimum Continuation Quality +50%
Minimum Continuation Quality -50%
Direction Coefficient 0.2
Direction Coefficient 0.1
Minimum Length 100% Cylinder Length
Minimum Length 50% Cylinder Length
Parameter

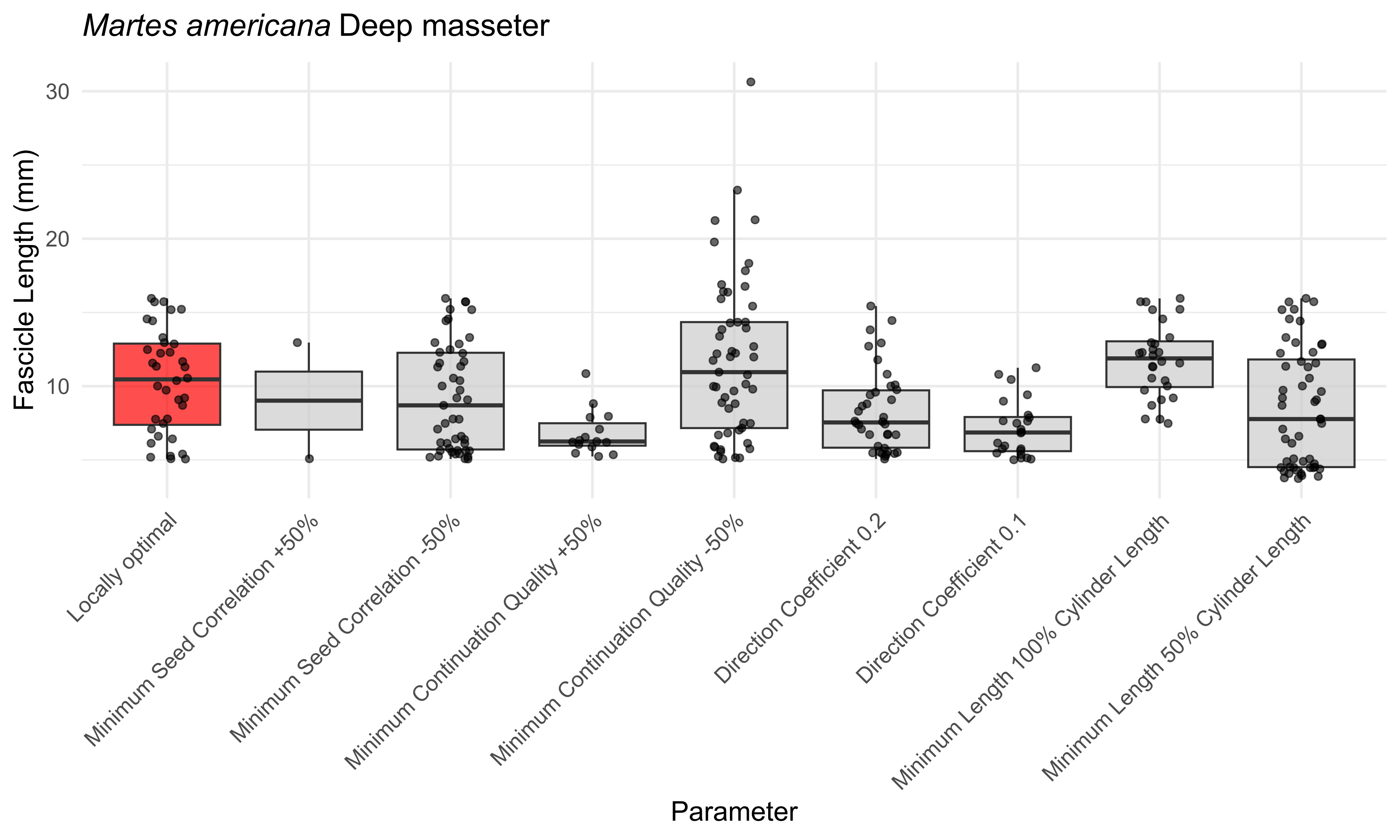

Martes americana Deep masseter
Fascicle Length (mm)
30
20
10
Locally optimal
Minimum Seed Correlation +50%
Minimum Seed Correlation -50%
Minimum Continuation Quality +50%
Minimum Continuation Quality -50%
Direction Coefficient 0.2
Direction Coefficient 0.1
Minimum Length 100% Cylinder Length
Minimum Length 50% Cylinder Length
Parameter

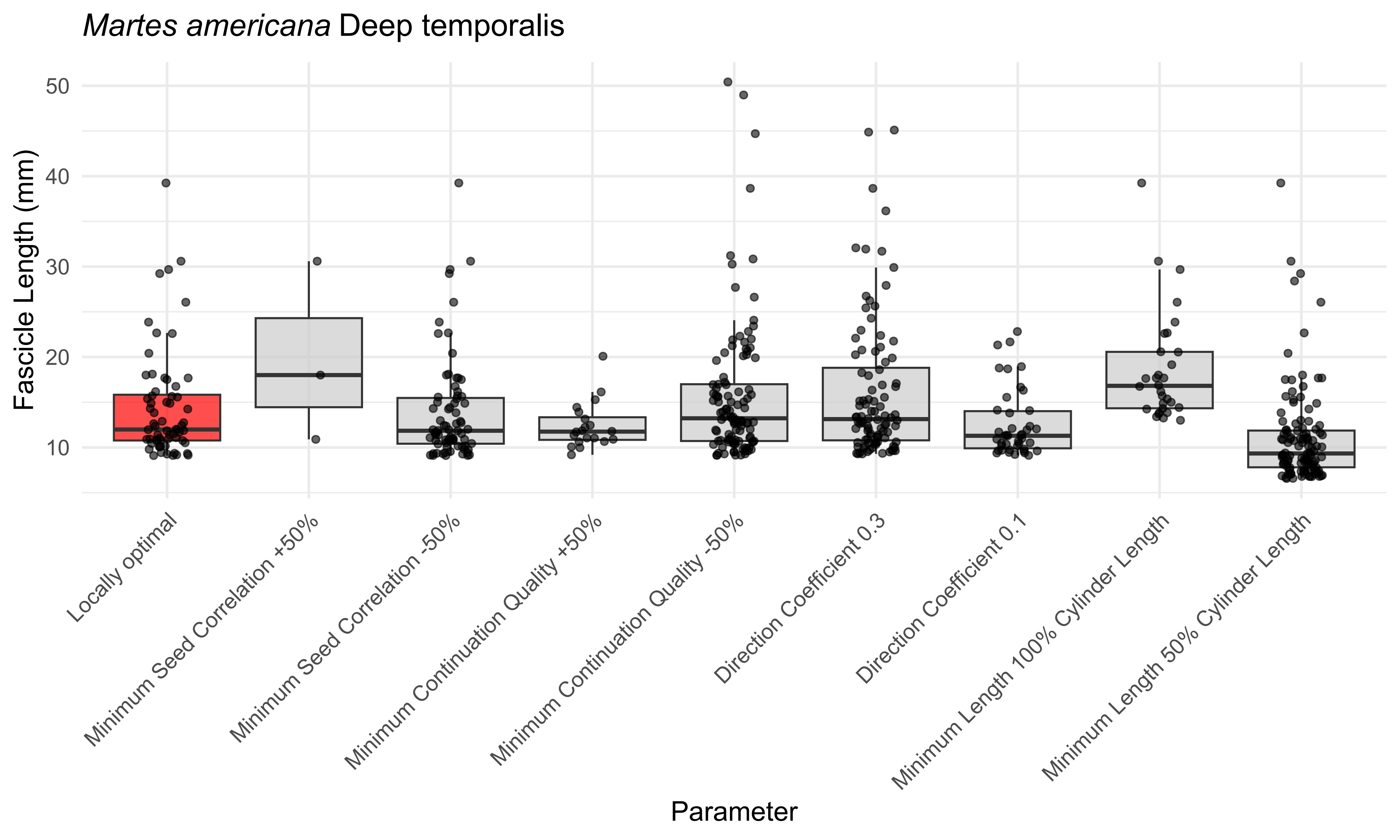
Martes americana Deep temporalis
Fascicle Length (mm)
50
40
30
20
10
Locally optimal
Minimum Seed Correlation +50%
Minimum Seed Correlation -50%
Minimum Continuation Quality +50%
Minimum Continuation Quality -50%
Direction Coefficient 0.3
Direction Coefficient 0.1
Minimum Length 100% Cylinder Length
Minimum Length 50% Cylinder Length
Parameter

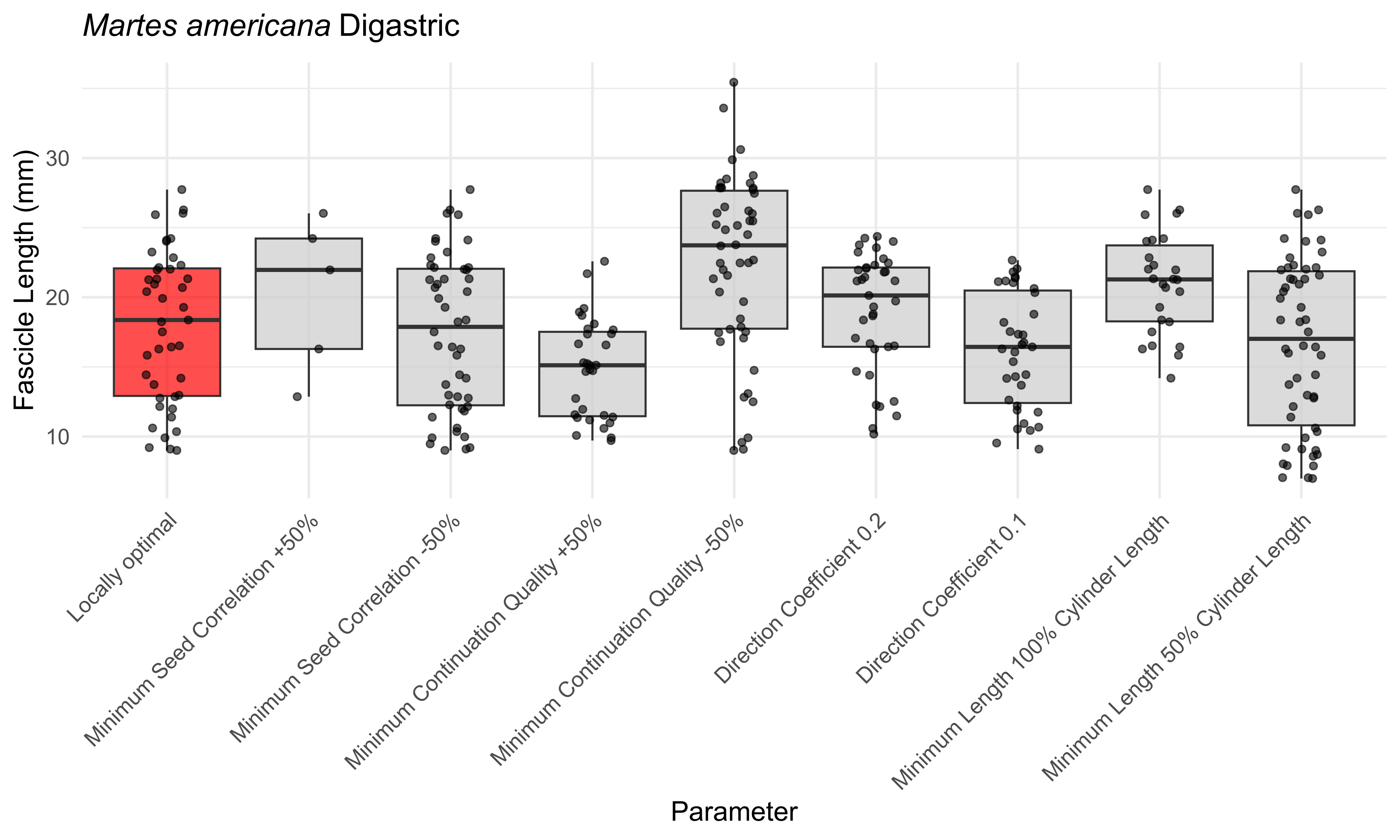

Martes americana Digastric
Fascicle Length (mm)
30
20
10
Locally optimal
Minimum Seed Correlation +50%
Minimum Seed Correlation -50%
Minimum Continuation Quality +50%
Minimum Continuation Quality -50%
Direction Coefficient 0.2
Direction Coefficient 0.1
Minimum Length 100% Cylinder Length
Minimum Length 50% Cylinder Length
Parameter

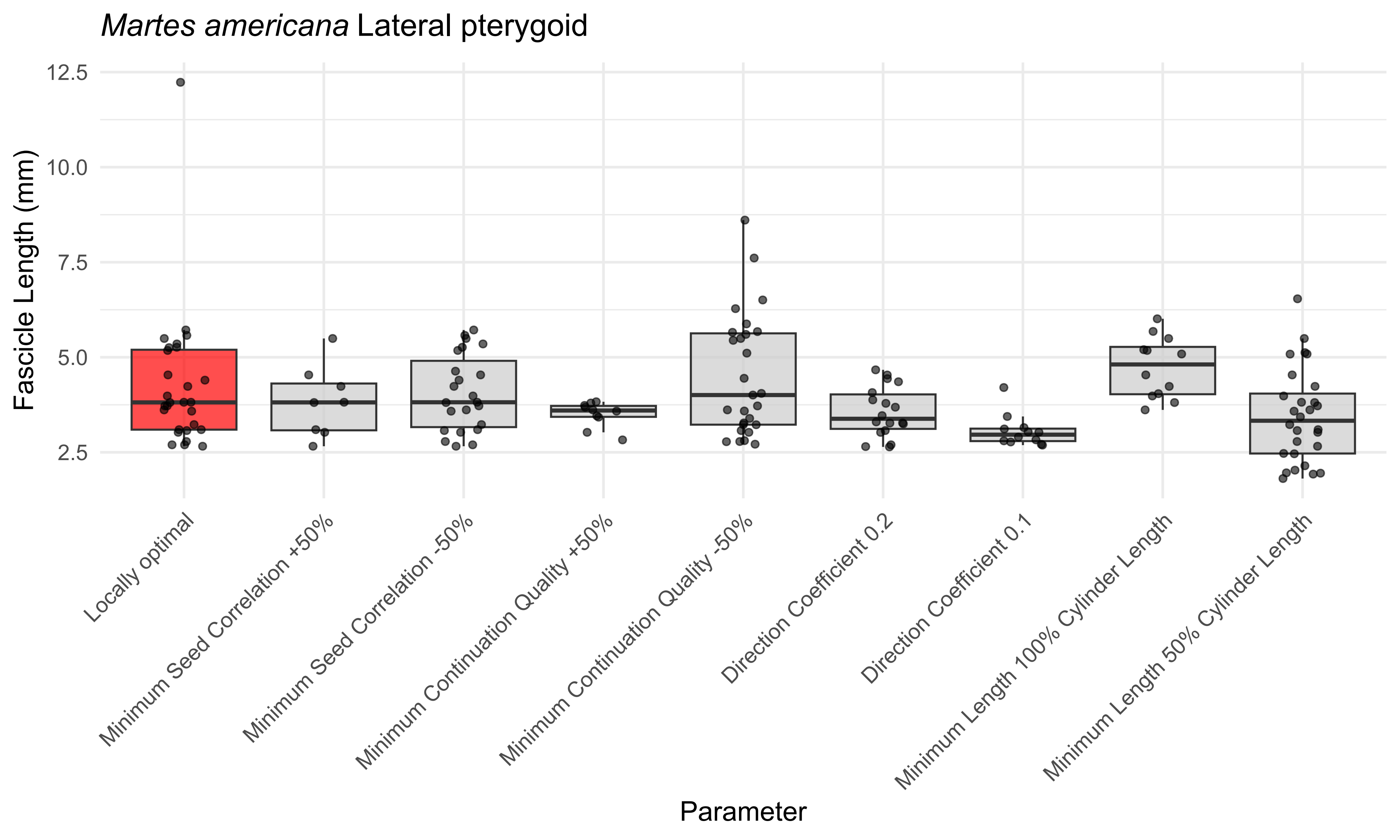
Martes americana Lateral pterygoid
Fascicle Length (mm)
12.5
10.0
7.5
5.0
2.5
Locally optimal
Minimum Seed Correlation +50%
Minimum Seed Correlation -50%
Minimum Continuation Quality +50%
Minimum Continuation Quality -50%
Direction Coefficient 0.2
Direction Coefficient 0.1
Minimum Length 100% Cylinder Length
Minimum Length 50% Cylinder Length
Parameter

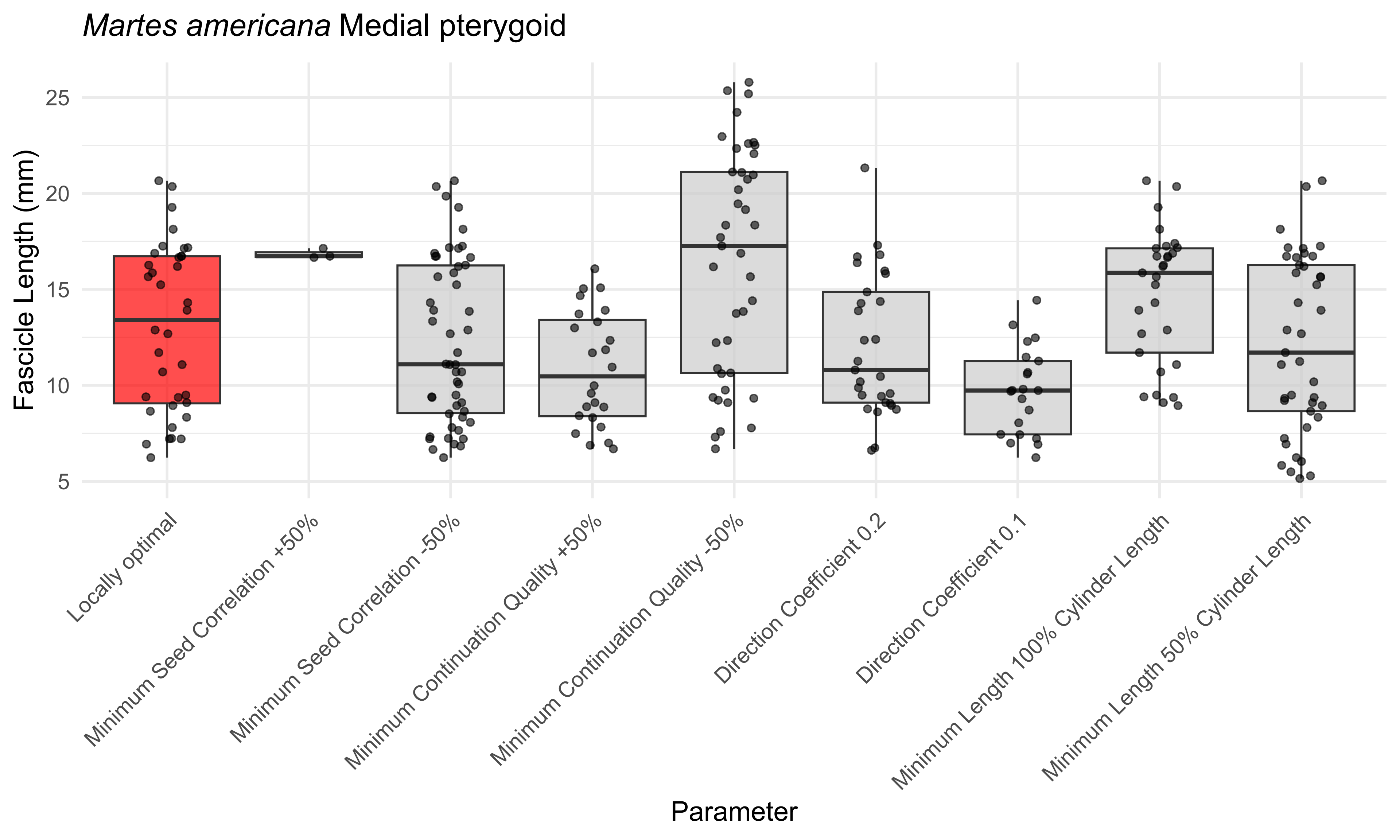

Martes americana Medial pterygoid
Fascicle Length (mm)
25
20
15
10
5
Locally optimal
Minimum Seed Correlation +50%
Minimum Seed Correlation -50%
Minimum Continuation Quality +50%
Minimum Continuation Quality -50%
Direction Coefficient 0.2
Direction Coefficient 0.1
Minimum Length 100% Cylinder Length
Minimum Length 50% Cylinder Length
Parameter

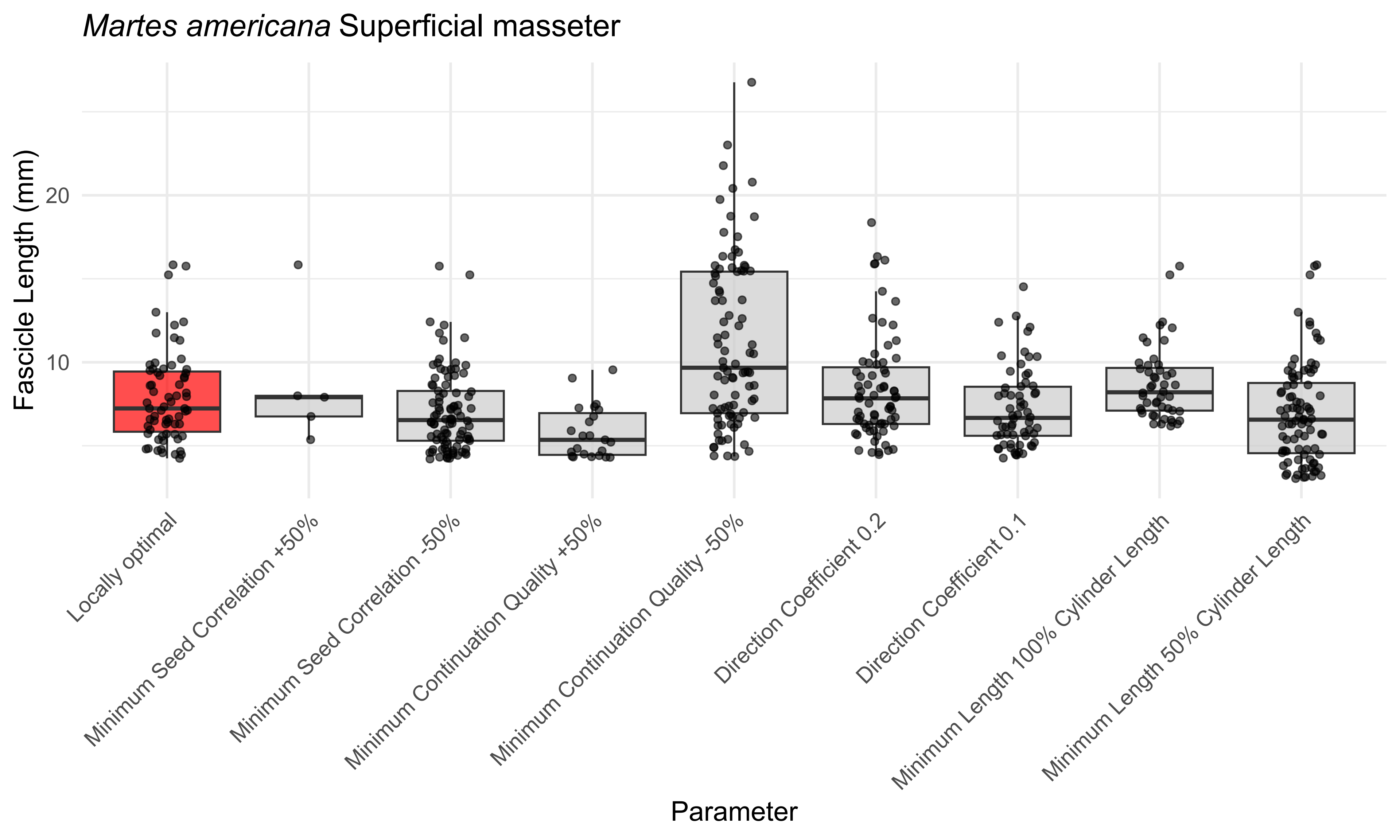

Martes americana Superficial masseter
Fascicle Length (mm)
20
10
Locally optimal
Minimum Seed Correlation +50%
Minimum Seed Correlation -50%
Minimum Continuation Quality +50%
Minimum Continuation Quality -50%
Direction Coefficient 0.2
Direction Coefficient 0.1
Minimum Length 100% Cylinder Length
Minimum Length 50% Cylinder Length
Parameter

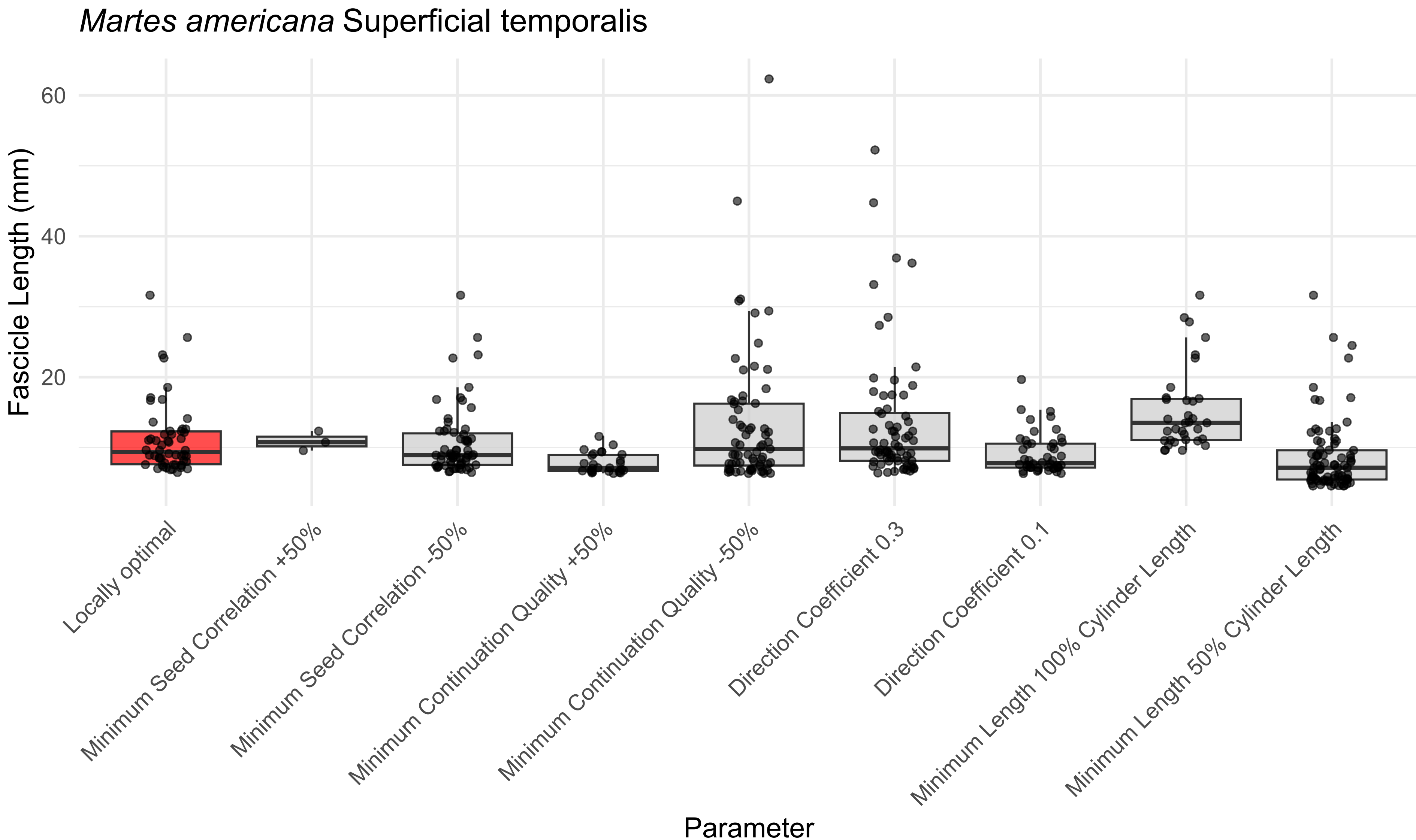
Martes americana Superficial temporalis
Fascicle Length (mm)
60
40
20
Locally optimal
Minimum Seed Correlation +50%
Minimum Seed Correlation -50%
Minimum Continuation Quality +50%
Minimum Continuation Quality -50%
Direction Coefficient 0.3
Direction Coefficient 0.1
Minimum Length 100% Cylinder Length
Minimum Length 50% Cylinder Length
Parameter

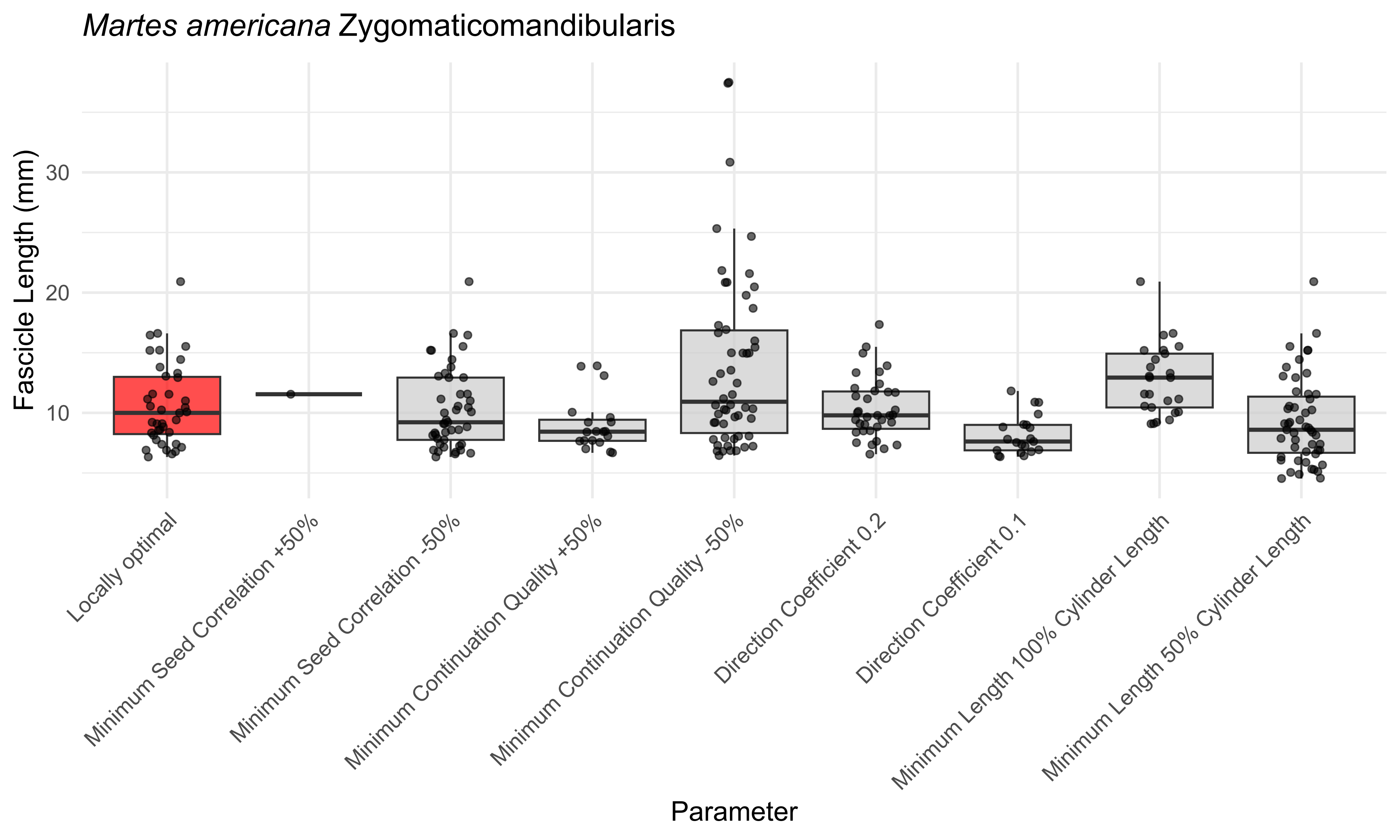
Martes americana Zygomaticomandibularis
Fascicle Length (mm)
30
20
10
Locally optimal
Minimum Seed Correlation +50%
Minimum Seed Correlation -50%
Minimum Continuation Quality +50%
Minimum Continuation Quality -50%
Direction Coefficient 0.2
Direction Coefficient 0.1
Minimum Length 100% Cylinder Length
Minimum Length 50% Cylinder Length
Parameter

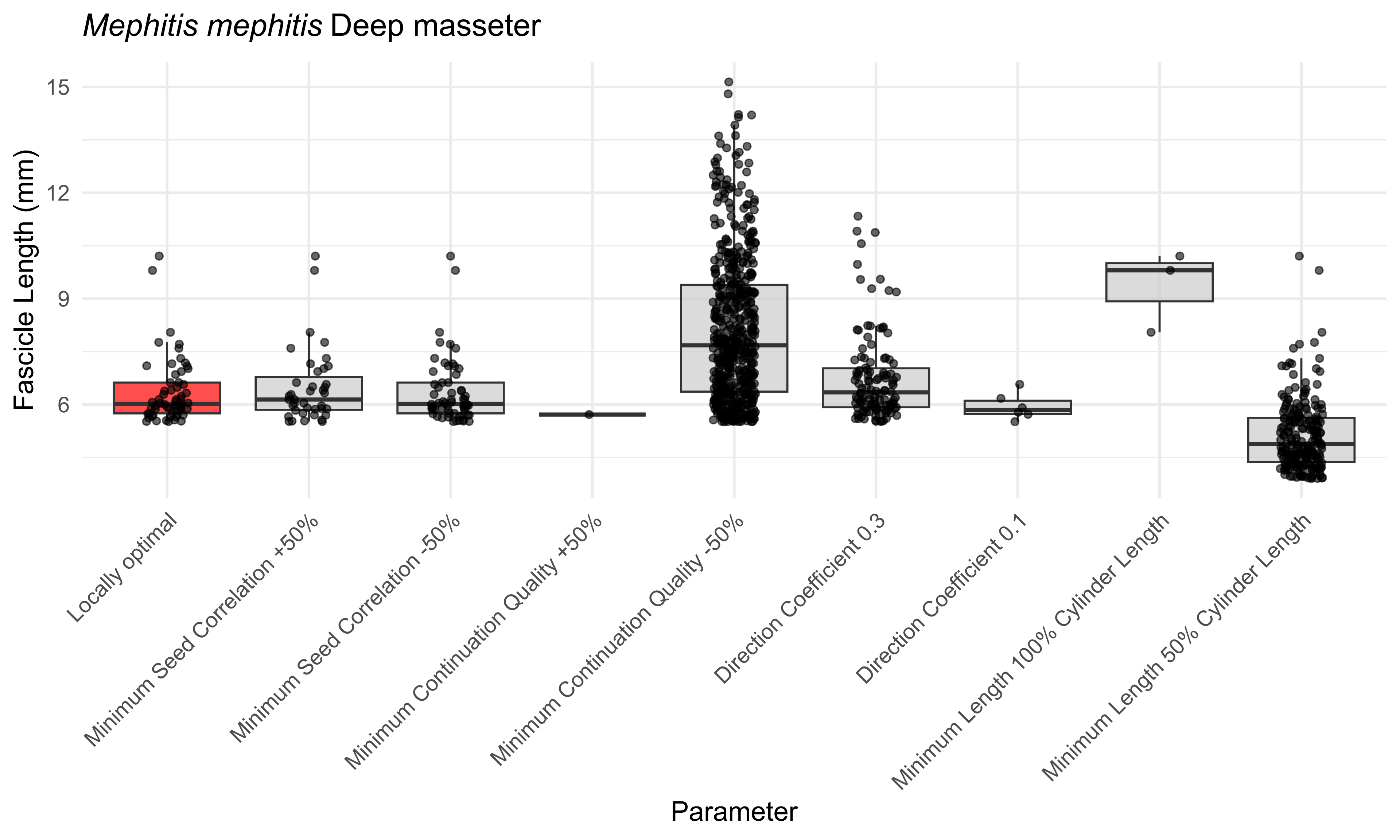
Mephitis mephitis Deep masseter
Fascicle Length (mm)
15
12
9
6
Locally optimal
Minimum Seed Correlation +50%
Minimum Seed Correlation -50%
Minimum Continuation Quality +50%
Minimum Continuation Quality -50%
Direction Coefficient 0.3
Direction Coefficient 0.1
Minimum Length 100% Cylinder Length
Minimum Length 50% Cylinder Length
Parameter

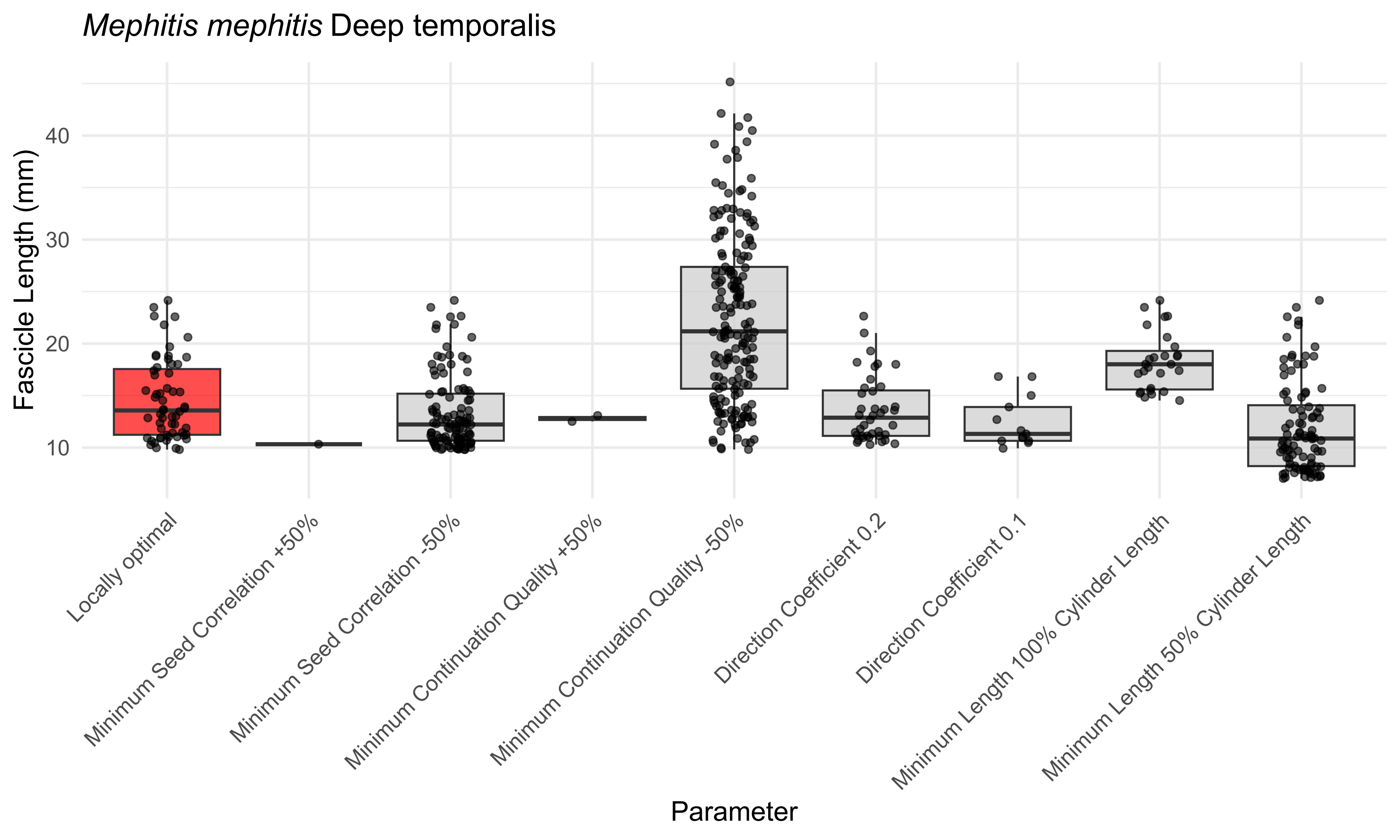

Mephitis mephitis Deep temporalis
Fascicle Length (mm)
40
30
20
10
Locally optimal
Minimum Seed Correlation +50%
Minimum Seed Correlation -50%
Minimum Continuation Quality +50%
Minimum Continuation Quality -50%
Direction Coefficient 0.2
Direction Coefficient 0.1
Minimum Length 100% Cylinder Length
Minimum Length 50% Cylinder Length
Parameter

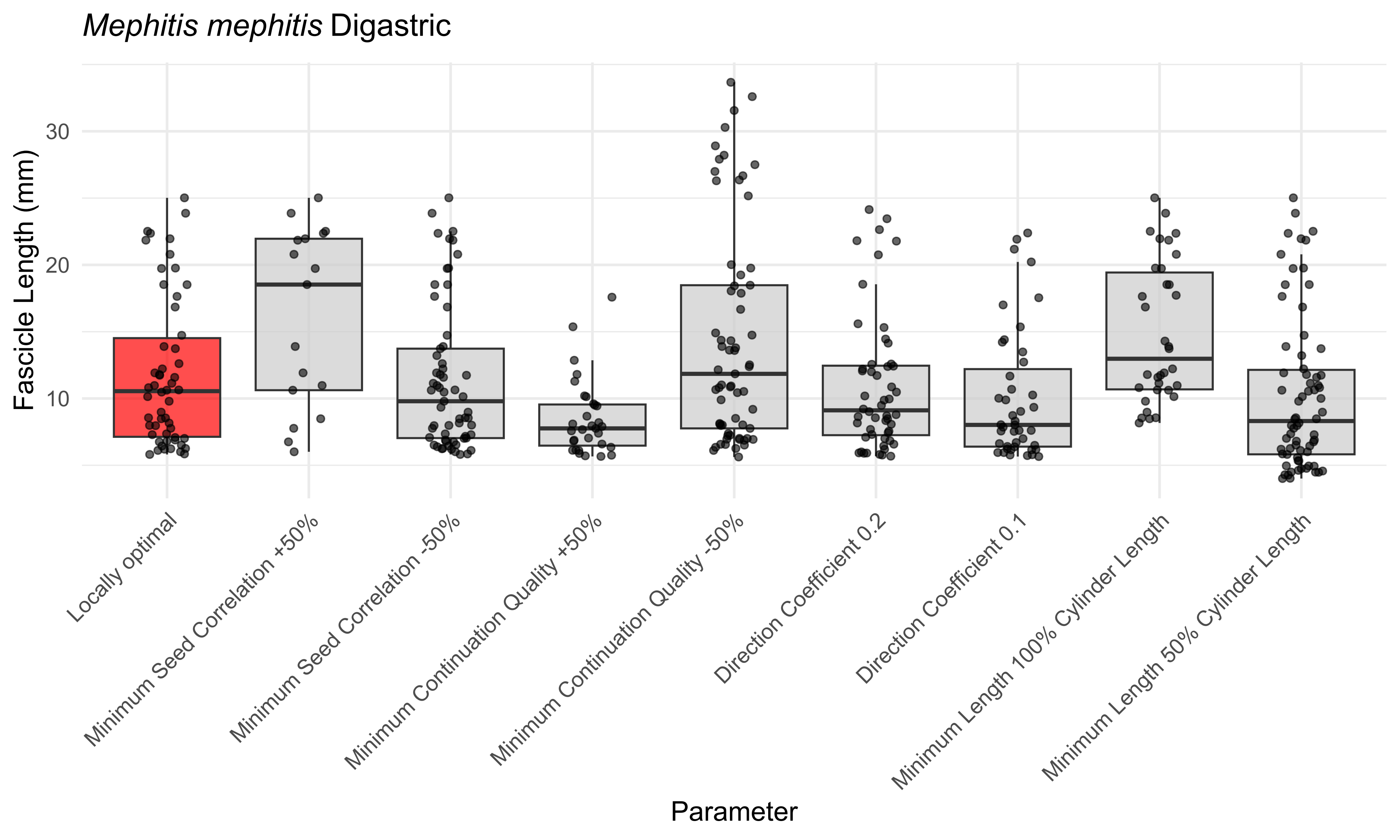

Mephitis mephitis Digastric
Fascicle Length (mm)
30
20
10
Locally optimal
Minimum Seed Correlation +50%
Minimum Seed Correlation -50%
Minimum Continuation Quality +50%
Minimum Continuation Quality -50%
Direction Coefficient 0.2
Direction Coefficient 0.1
Minimum Length 100% Cylinder Length
Minimum Length 50% Cylinder Length
Parameter

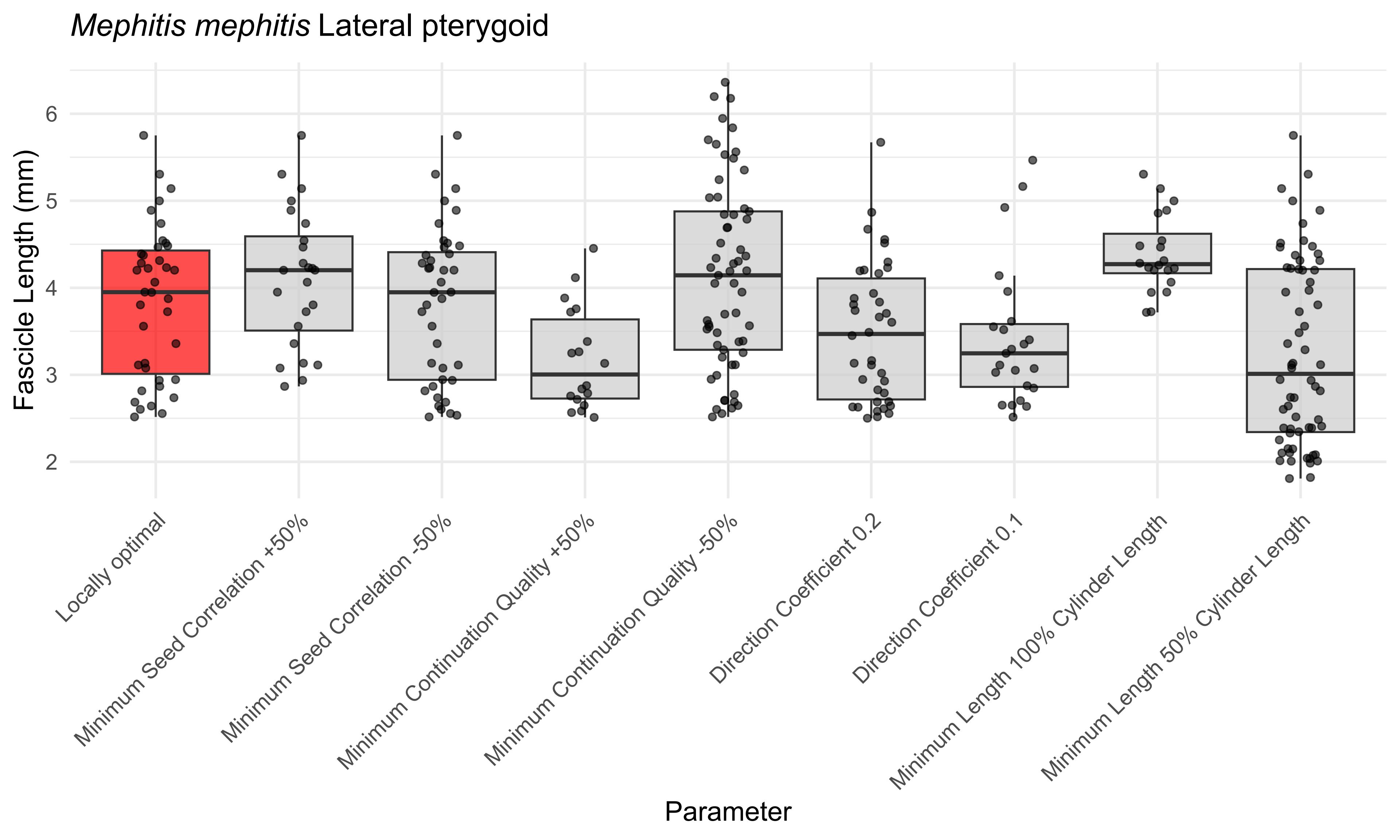

Mephitis mephitis Lateral pterygoid
Fascicle Length (mm)
6
5
4
3
2
Locally optimal
Minimum Seed Correlation +50%
Minimum Seed Correlation -50%
Minimum Continuation Quality +50%
Minimum Continuation Quality -50%
Direction Coefficient 0.2
Direction Coefficient 0.1
Minimum Length 100% Cylinder Length
Minimum Length 50% Cylinder Length
Parameter

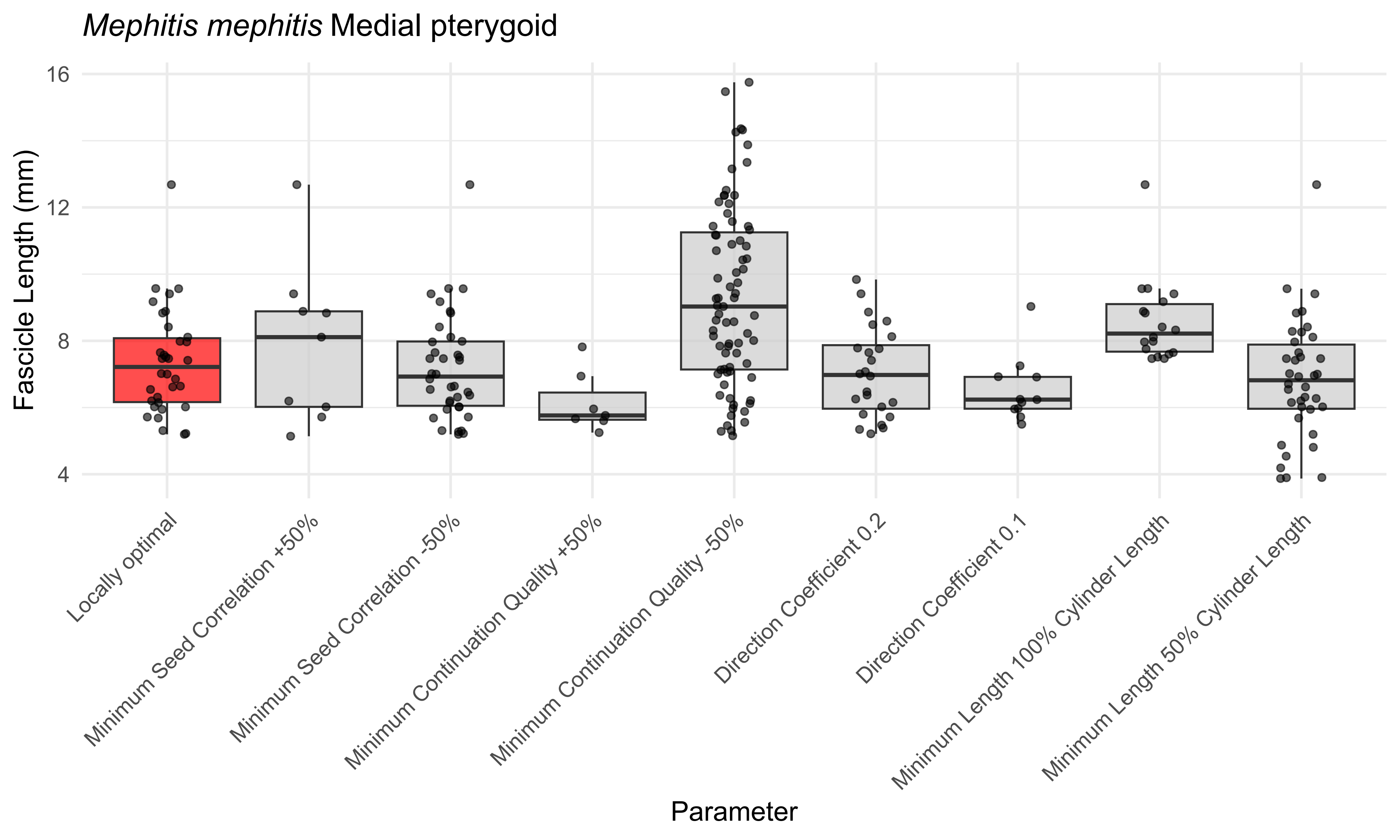

Mephitis mephitis Medial pterygoid
Fascicle Length (mm)
16
12
8
4
Locally optimal
Minimum Seed Correlation +50%
Minimum Seed Correlation -50%
Minimum Continuation Quality +50%
Minimum Continuation Quality -50%
Direction Coefficient 0.2
Direction Coefficient 0.1
Minimum Length 100% Cylinder Length
Minimum Length 50% Cylinder Length
Parameter

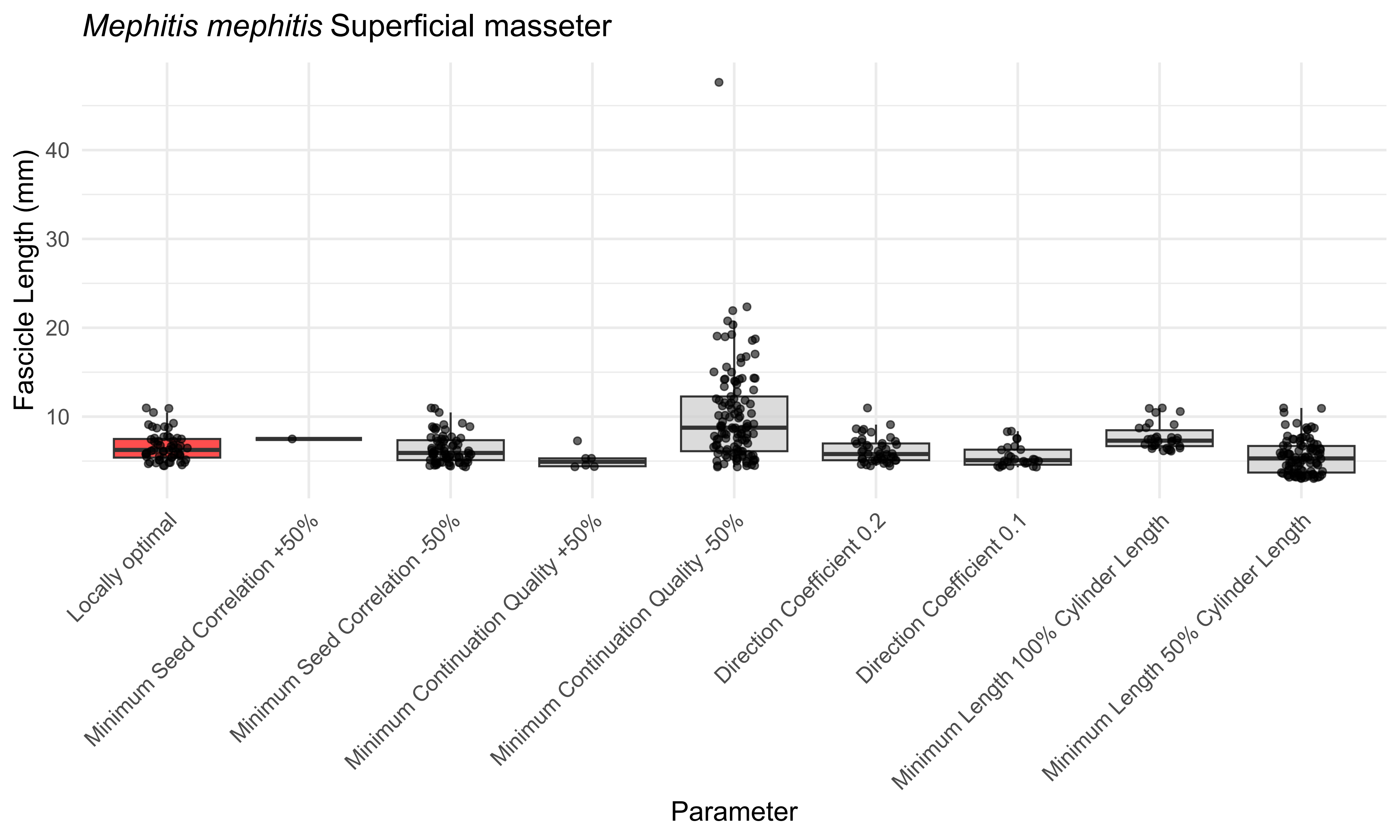
Mephitis mephitis Superficial masseter
Fascicle Length (mm)
40
30
20
10
Locally optimal
Minimum Seed Correlation +50%
Minimum Seed Correlation -50%
Minimum Continuation Quality +50%
Minimum Continuation Quality -50%
Direction Coefficient 0.2
Direction Coefficient 0.1
Minimum Length 100% Cylinder Length
Minimum Length 50% Cylinder Length
Parameter

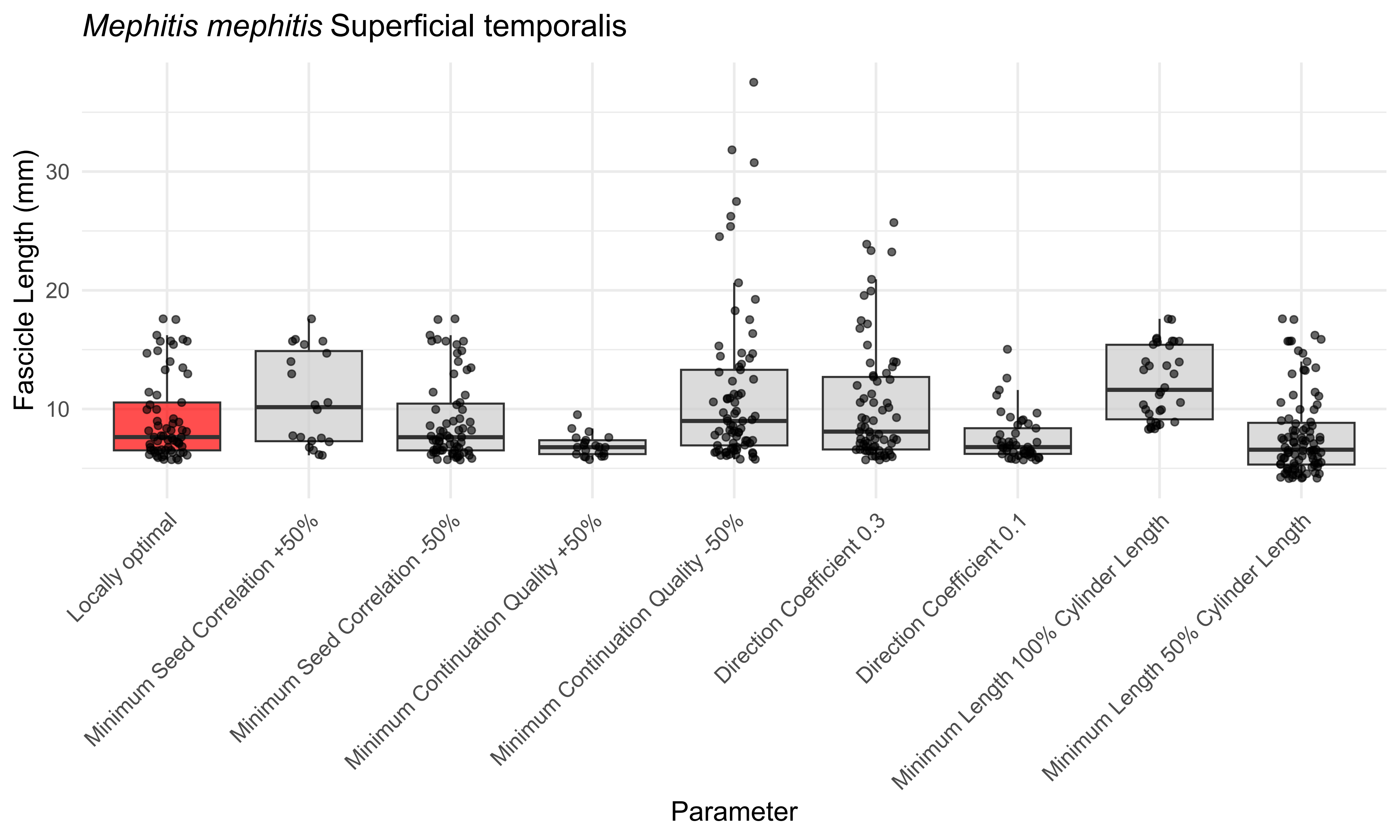

Mephitis mephitis Superficial temporalis
Fascicle Length (mm)
30
20
10
Locally optimal
Minimum Seed Correlation +50%
Minimum Seed Correlation -50%
Minimum Continuation Quality +50%
Minimum Continuation Quality -50%
Direction Coefficient 0.3
Direction Coefficient 0.1
Minimum Length 100% Cylinder Length
Minimum Length 50% Cylinder Length
Parameter

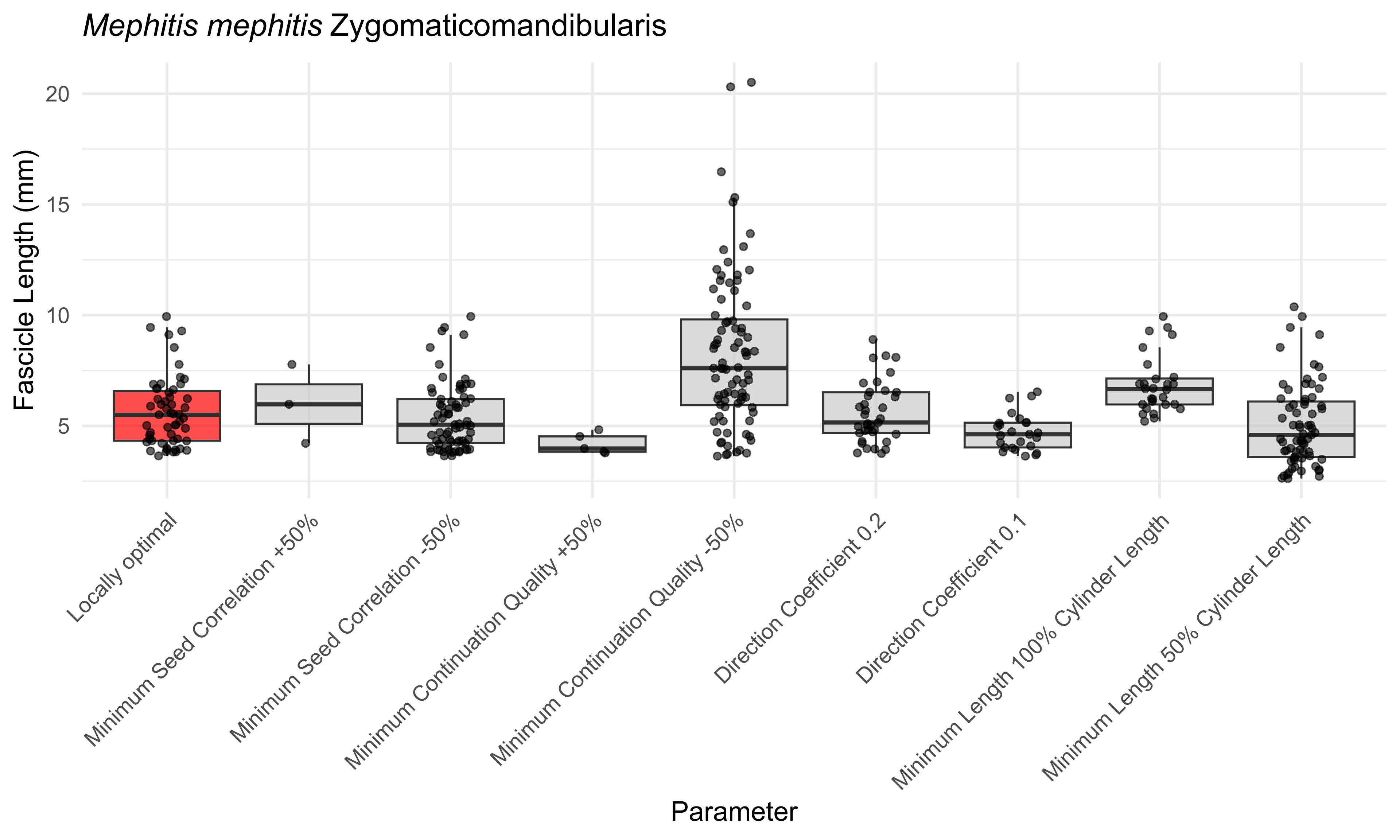

Mephitis mephitis Zygomaticomandibularis
Fascicle Length (mm)
20
15
10
5
Locally optimal
Minimum Seed Correlation +50%
Minimum Seed Correlation -50%
Minimum Continuation Quality +50%
Minimum Continuation Quality -50%
Direction Coefficient 0.2
Direction Coefficient 0.1
Minimum Length 100% Cylinder Length
Minimum Length 50% Cylinder Length
Parameter

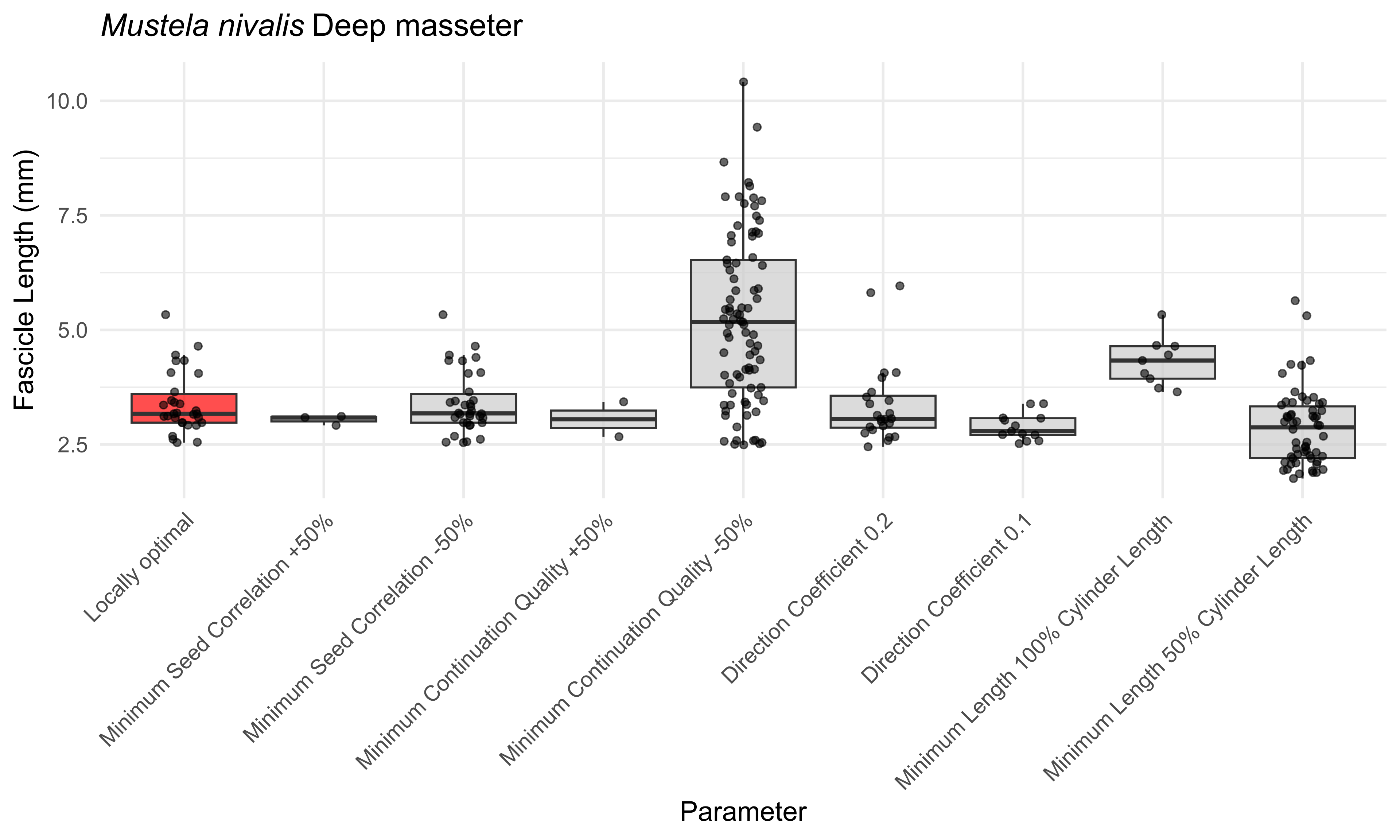

Mustela nivalis Deep masseter
Fascicle Length (mm)
10.0
7.5
5.0
2.5
Locally optimal
Minimum Seed Correlation +50%
Minimum Seed Correlation -50%
Minimum Continuation Quality +50%
Minimum Continuation Quality -50%
Direction Coefficient 0.2
Direction Coefficient 0.1
Minimum Length 100% Cylinder Length
Minimum Length 50% Cylinder Length
Parameter

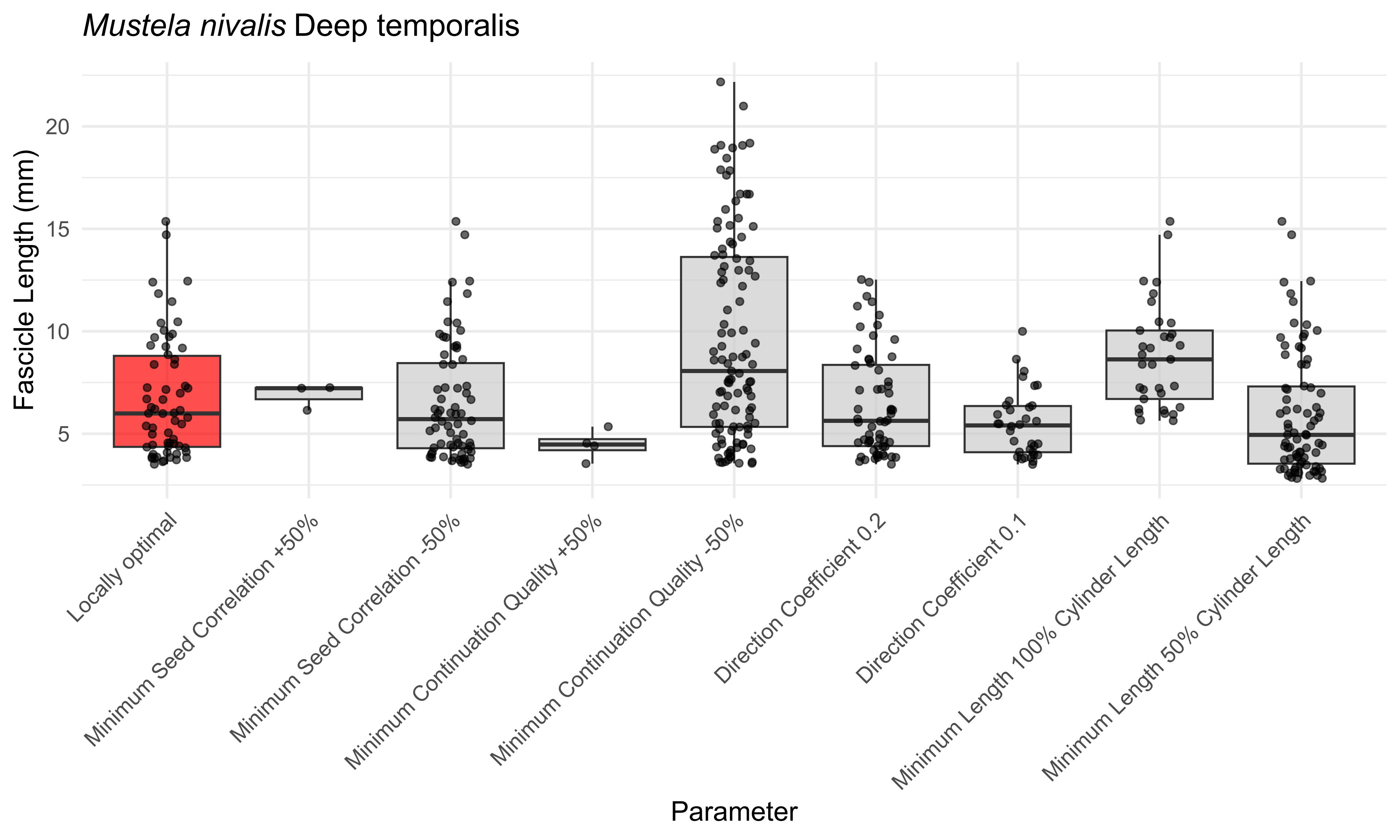

Mustela nivalis Deep temporalis
Fascicle Length (mm)
20
15
10
5
Locally optimal
Minimum Seed Correlation +50%
Minimum Seed Correlation -50%
Minimum Continuation Quality +50%
Minimum Continuation Quality -50%
Direction Coefficient 0.2
Direction Coefficient 0.1
Minimum Length 100% Cylinder Length
Minimum Length 50% Cylinder Length
Parameter

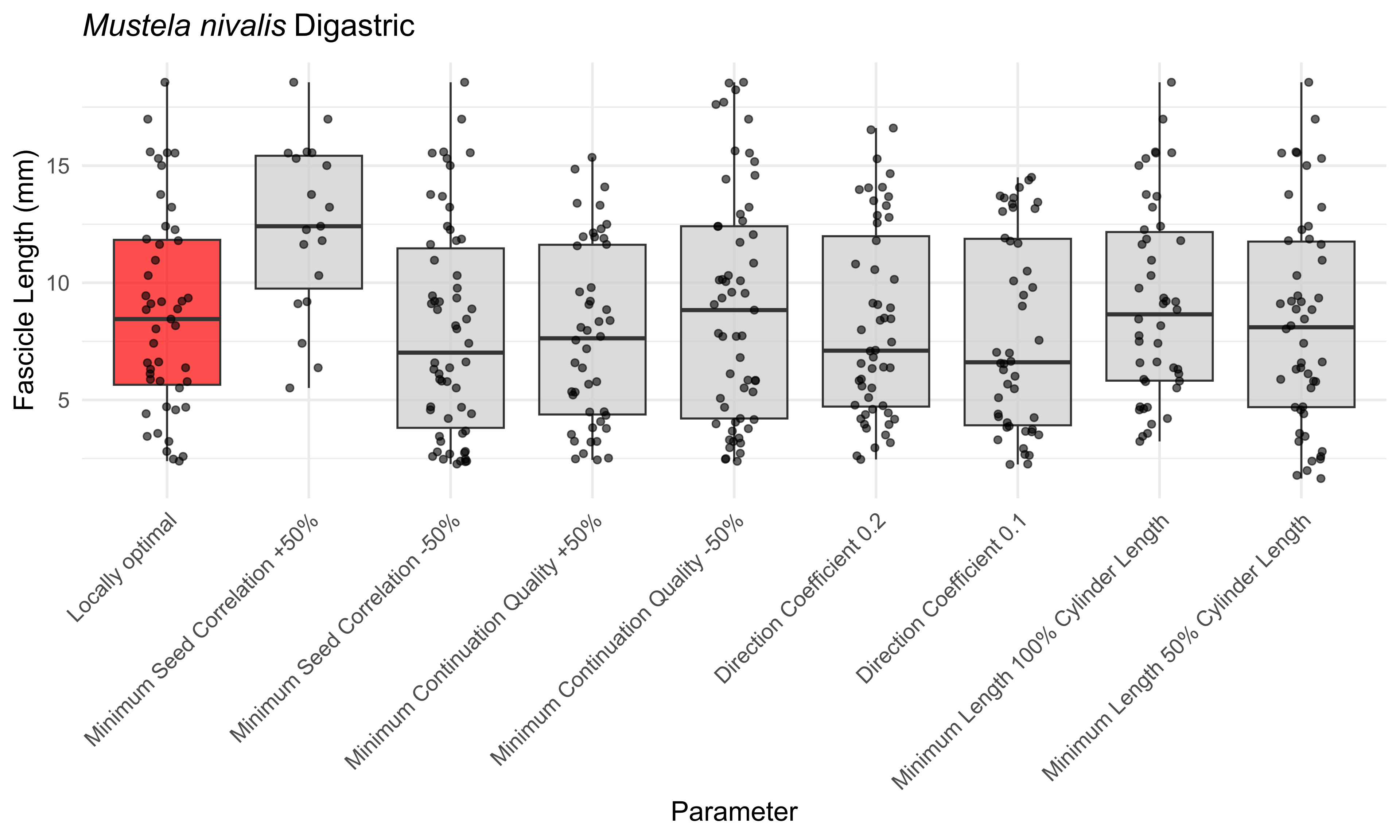

Mustela nivalis Digastric
Fascicle Length (mm)
5
10
15
Locally optimal
Minimum Seed Correlation +50%
Minimum Seed Correlation -50%
Minimum Continuation Quality +50%
Minimum Continuation Quality -50%
Direction Coefficient 0.2
Direction Coefficient 0.1
Minimum Length 100% Cylinder Length
Minimum Length 50% Cylinder Length
Parameter

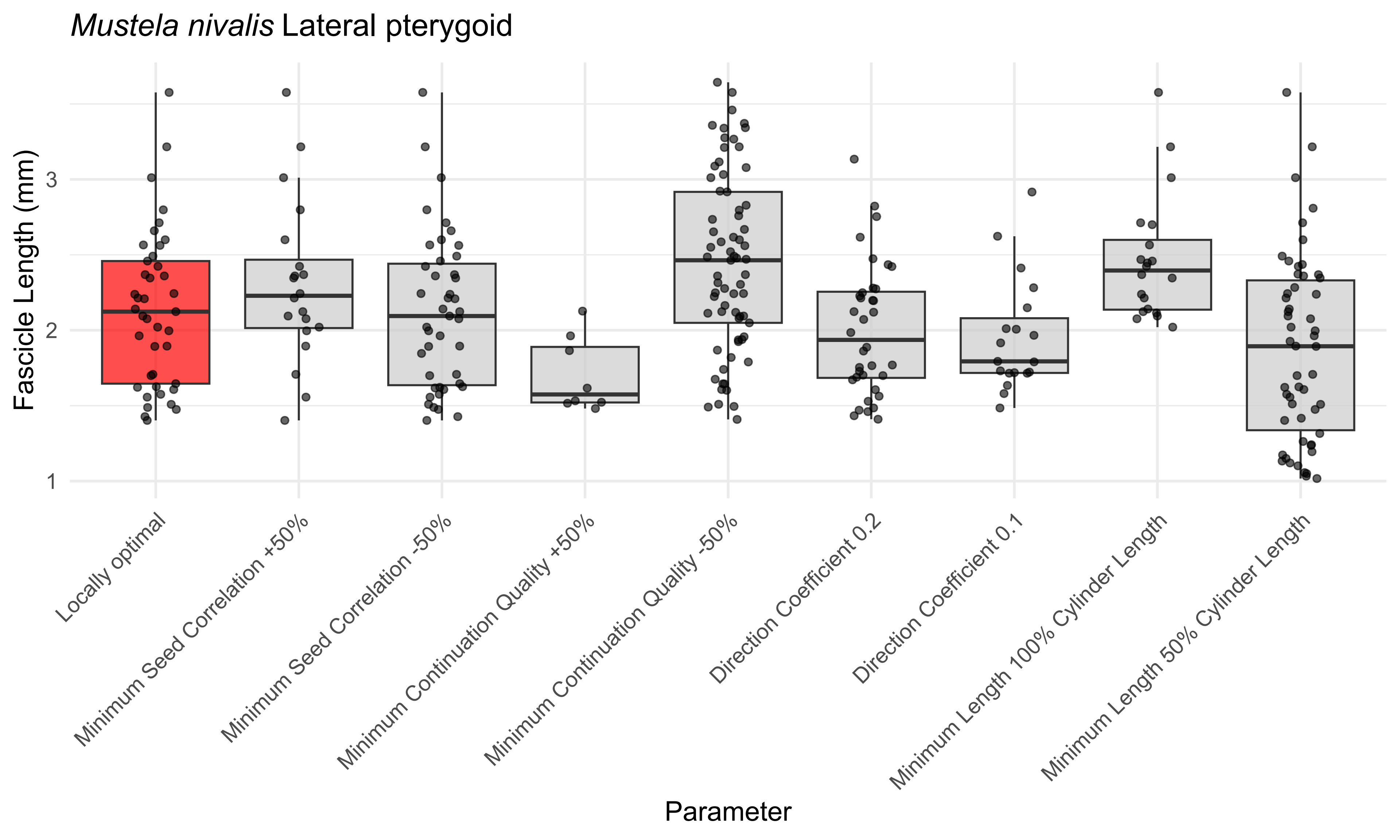
Mustela nivalis Lateral pterygoid
Fascicle Length (mm)
1
2
3
Locally optimal
Minimum Seed Correlation +50%
Minimum Seed Correlation -50%
Minimum Continuation Quality +50%
Minimum Continuation Quality -50%
Direction Coefficient 0.2
Direction Coefficient 0.1
Minimum Length 100% Cylinder Length
Minimum Length 50% Cylinder Length
Parameter

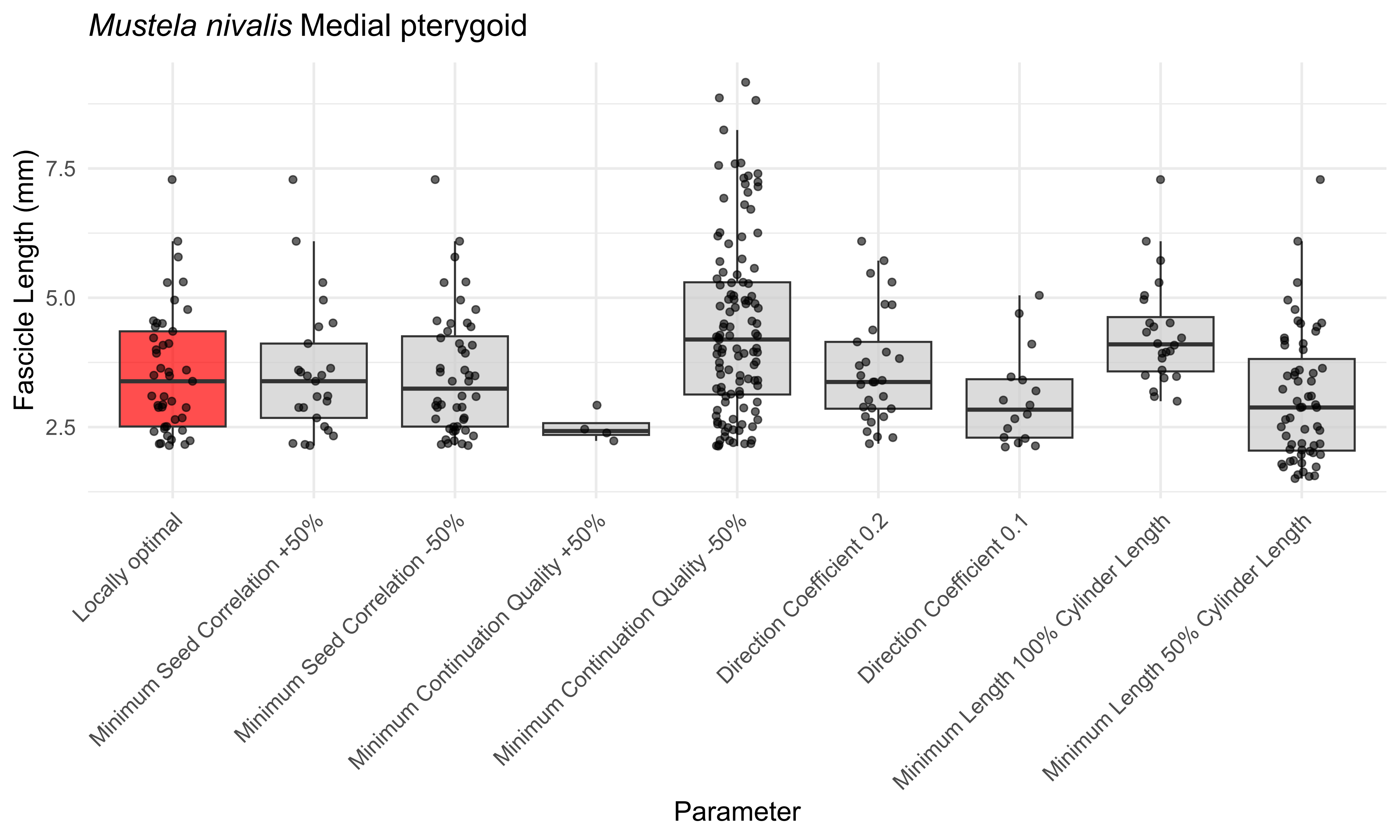

Mustela nivalis Medial pterygoid
Fascicle Length (mm)
7.5
5.0
2.5
Locally optimal
Minimum Seed Correlation +50%
Minimum Seed Correlation -50%
Minimum Continuation Quality +50%
Minimum Continuation Quality -50%
Direction Coefficient 0.2
Direction Coefficient 0.1
Minimum Length 100% Cylinder Length
Minimum Length 50% Cylinder Length
Parameter

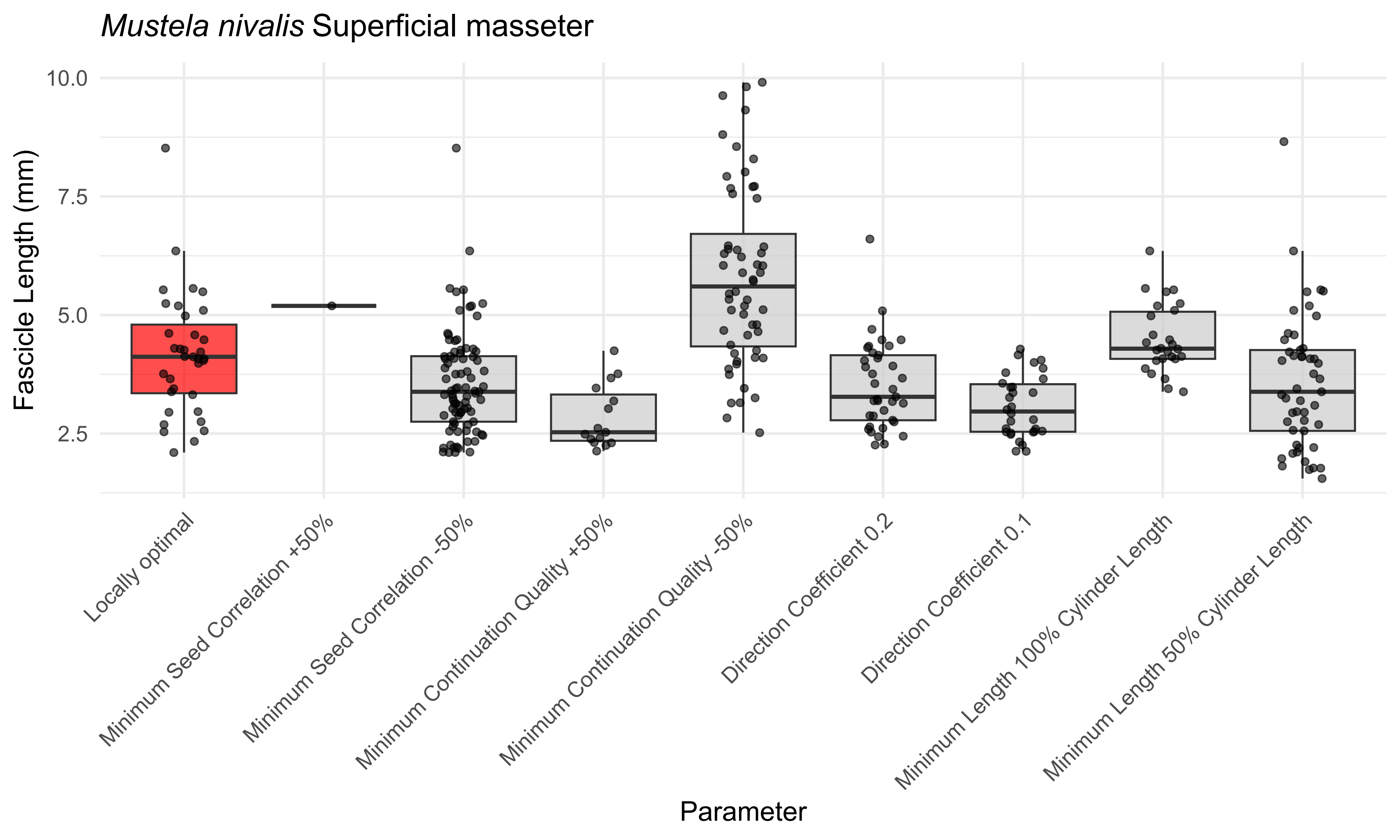

Mustela nivalis Superficial masseter
Fascicle Length (mm)
10.0
7.5
5.0
2.5
Locally optimal
Minimum Seed Correlation +50%
Minimum Seed Correlation -50%
Minimum Continuation Quality +50%
Minimum Continuation Quality -50%
Direction Coefficient 0.2
Direction Coefficient 0.1
Minimum Length 100% Cylinder Length
Minimum Length 50% Cylinder Length
Parameter

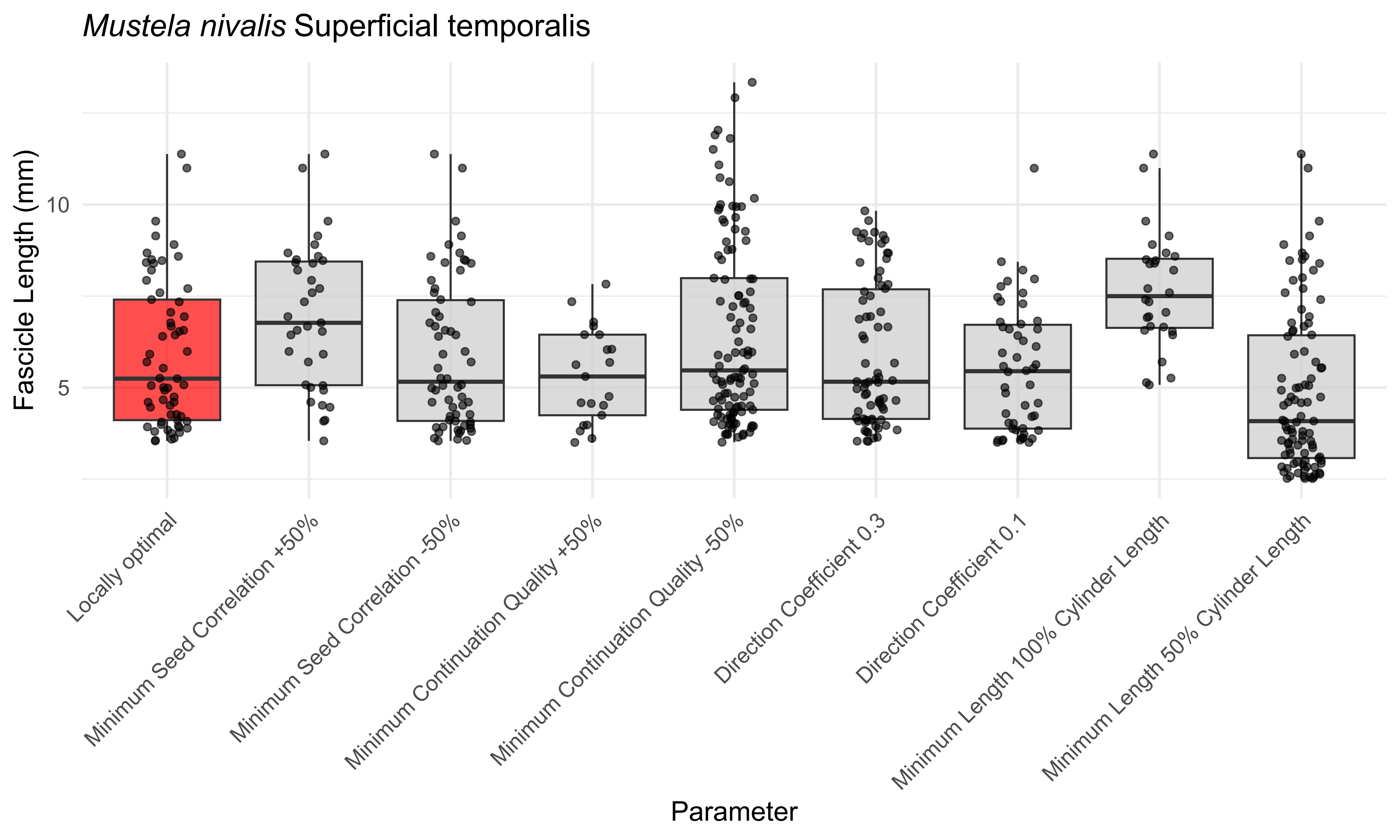

Mustela nivalis Superficial temporalis
Fascicle Length (mm)
10
5
Locally optimal
Minimum Seed Correlation +50%
Minimum Seed Correlation -50%
Minimum Continuation Quality +50%
Minimum Continuation Quality -50%
Direction Coefficient 0.3
Direction Coefficient 0.1
Minimum Length 100% Cylinder Length
Minimum Length 50% Cylinder Length
Parameter

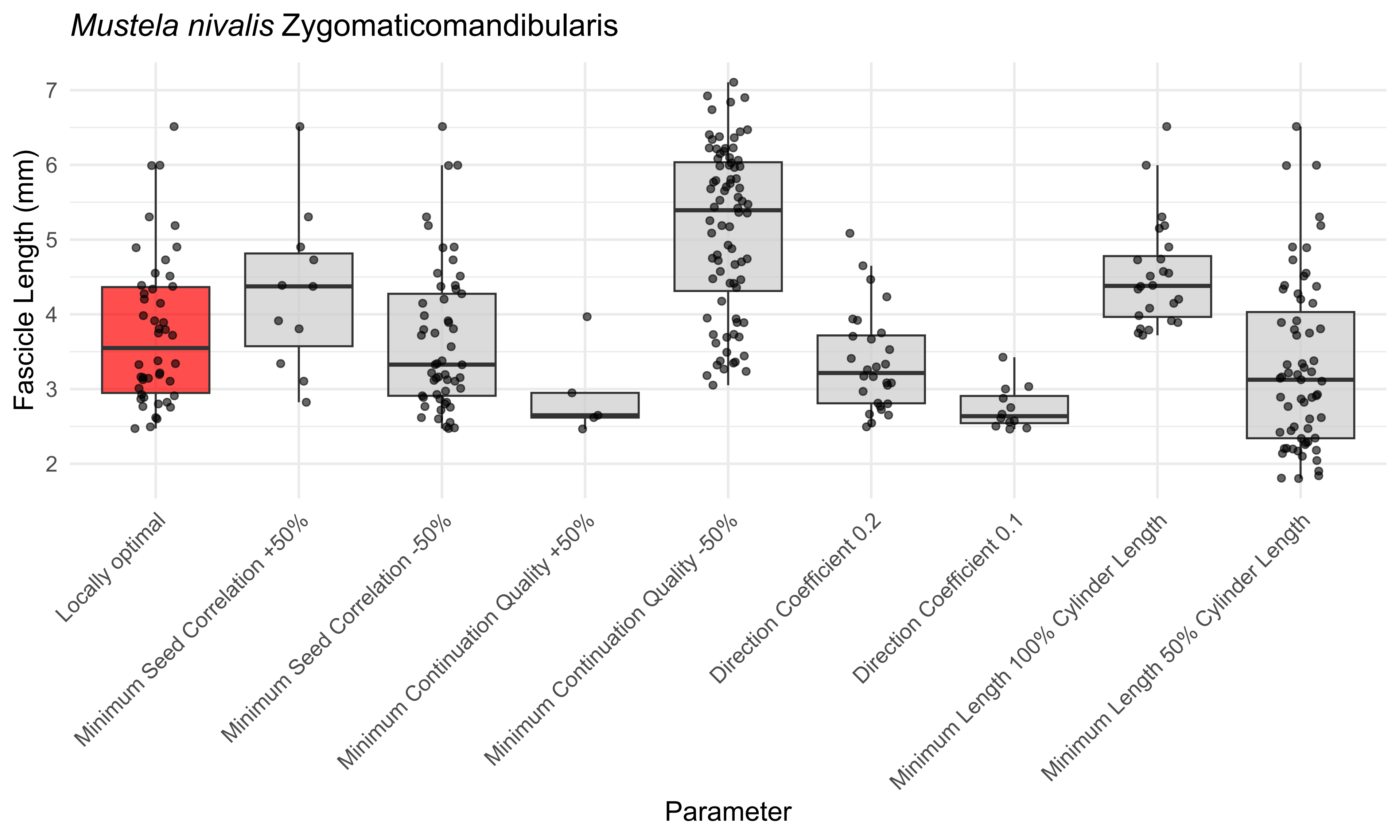
Mustela nivalis Zygomaticomandibularis
Fascicle Length (mm)
7
6
5
4
3
2
Locally optimal
Minimum Seed Correlation +50%
Minimum Seed Correlation -50%
Minimum Continuation Quality +50%
Minimum Continuation Quality -50%
Direction Coefficient 0.2
Direction Coefficient 0.1
Minimum Length 100% Cylinder Length
Minimum Length 50% Cylinder Length
Parameter

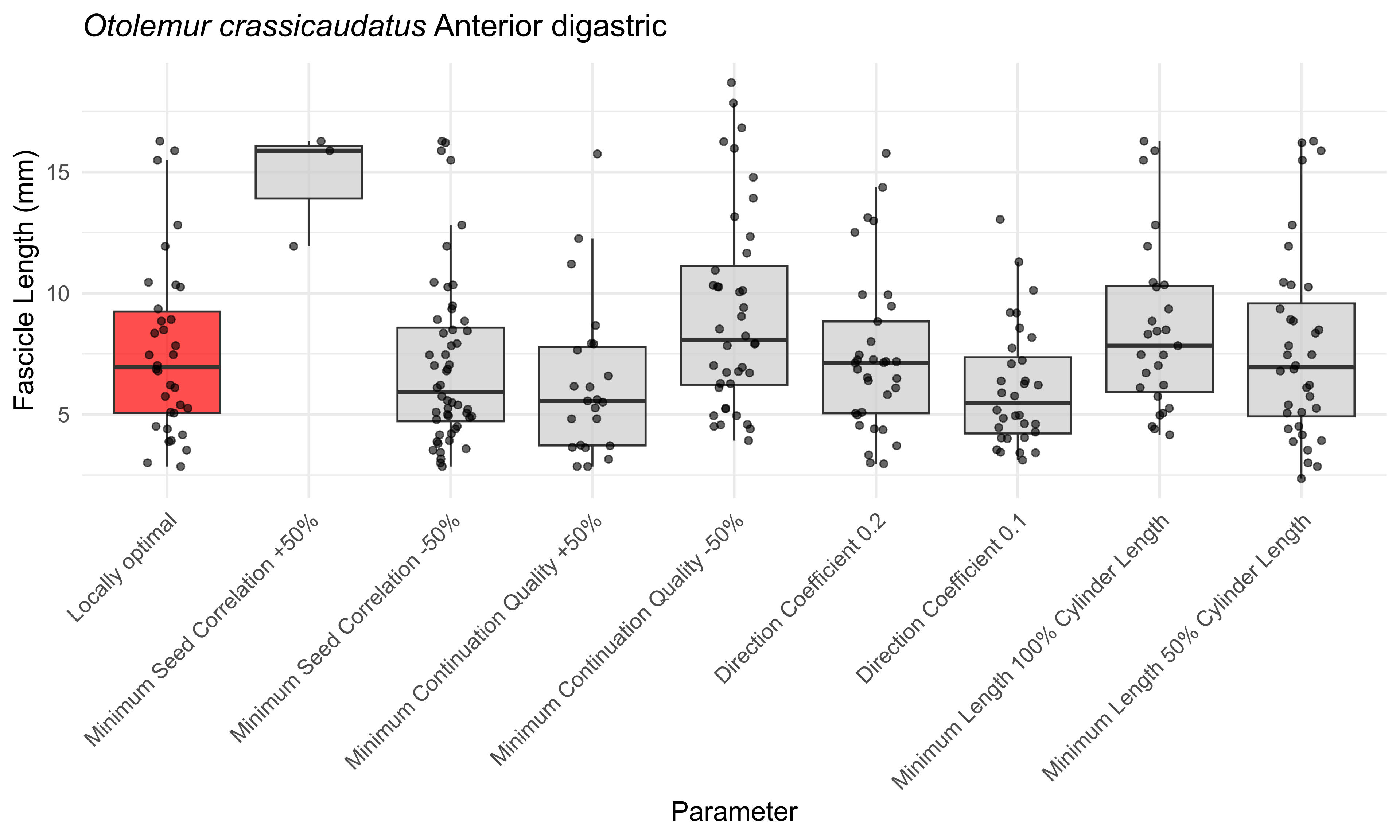

Otolemur crassicaudatus Anterior digastric
Fascicle Length (mm)
15
10
5
Locally optimal
Minimum Seed Correlation +50%
Minimum Seed Correlation -50%
Minimum Continuation Quality +50%
Minimum Continuation Quality -50%
Direction Coefficient 0.2
Direction Coefficient 0.1
Minimum Length 100% Cylinder Length
Minimum Length 50% Cylinder Length
Parameter

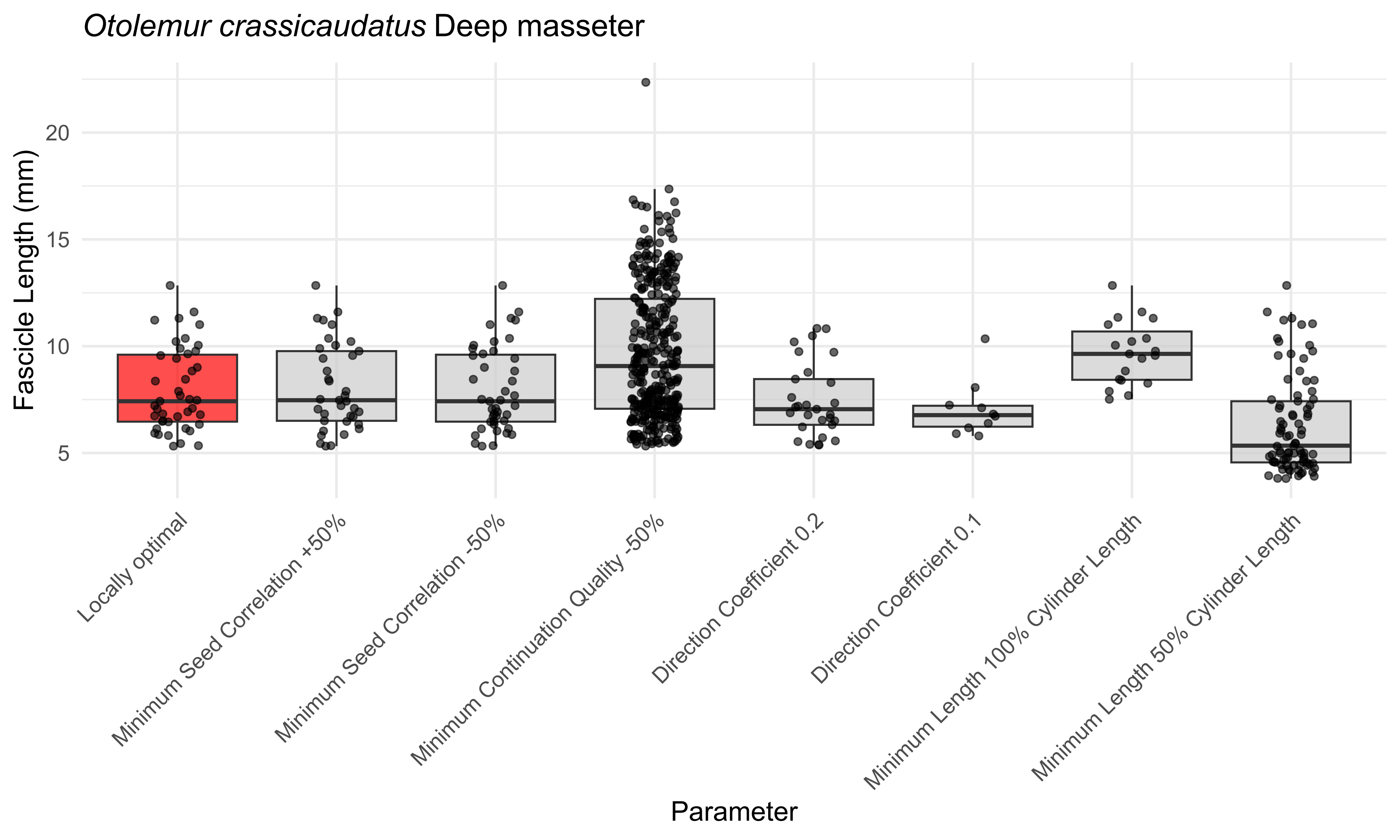

Otolemur crassicaudatus Deep masseter
Fascicle Length (mm)
5
10
15
20
Locally optimal
Minimum Seed Correlation +50%
Minimum Seed Correlation -50%
Minimum Continuation Quality -50%
Direction Coefficient 0.2
Direction Coefficient 0.1
Minimum Length 100% Cylinder Length
Minimum Length 50% Cylinder Length
Parameter

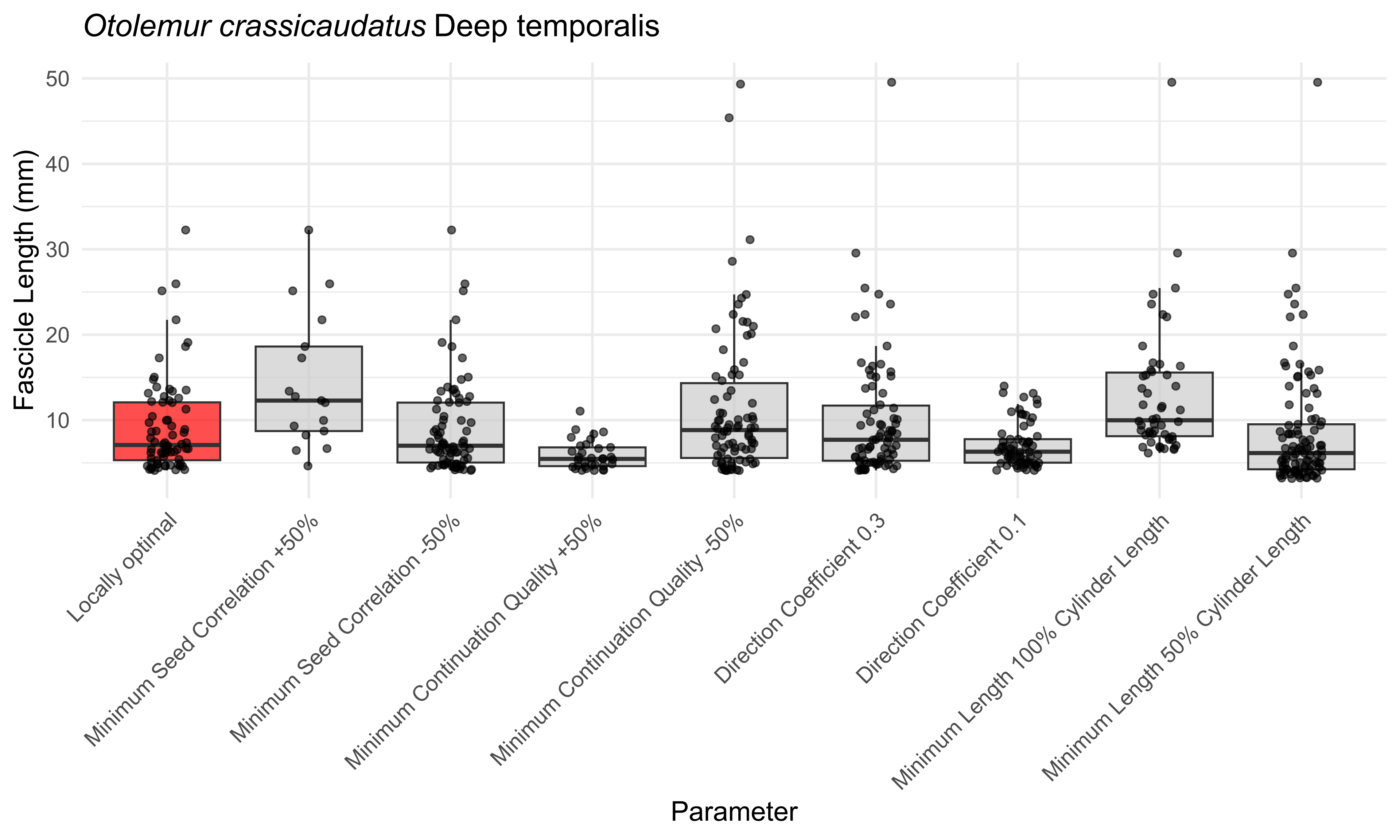

Otolemur crassicaudatus Deep temporalis
Fascicle Length (mm)
50
40
30
20
10
Locally optimal
Minimum Seed Correlation +50%
Minimum Seed Correlation -50%
Minimum Continuation Quality +50%
Minimum Continuation Quality -50%
Direction Coefficient 0.3
Direction Coefficient 0.1
Minimum Length 100% Cylinder Length
Minimum Length 50% Cylinder Length
Parameter

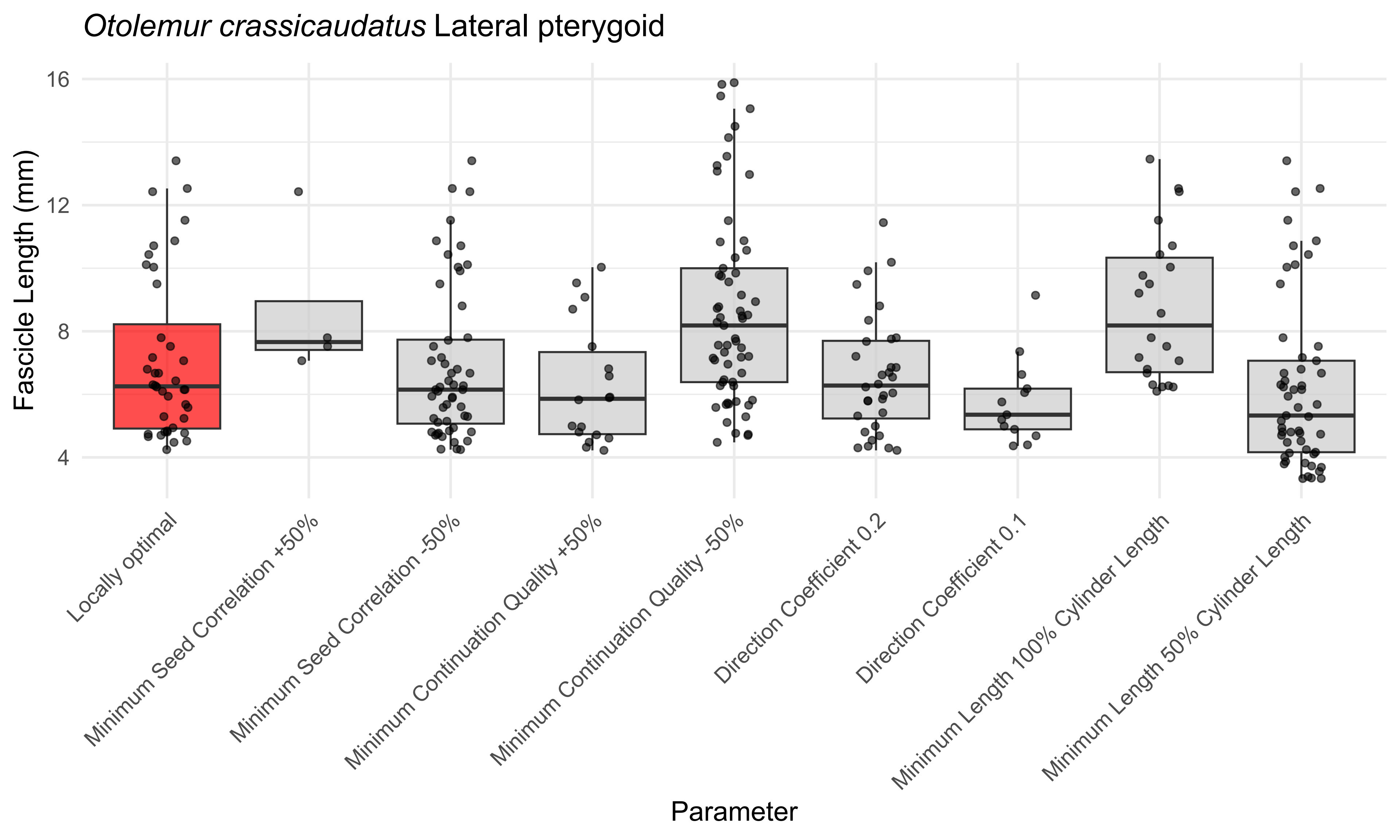
Otolemur crassicaudatus Lateral pterygoid
Fascicle Length (mm)
16
12
8
4
Locally optimal
Minimum Seed Correlation +50%
Minimum Seed Correlation -50%
Minimum Continuation Quality +50%
Minimum Continuation Quality -50%
Direction Coefficient 0.2
Direction Coefficient 0.1
Minimum Length 100% Cylinder Length
Minimum Length 50% Cylinder Length
Parameter

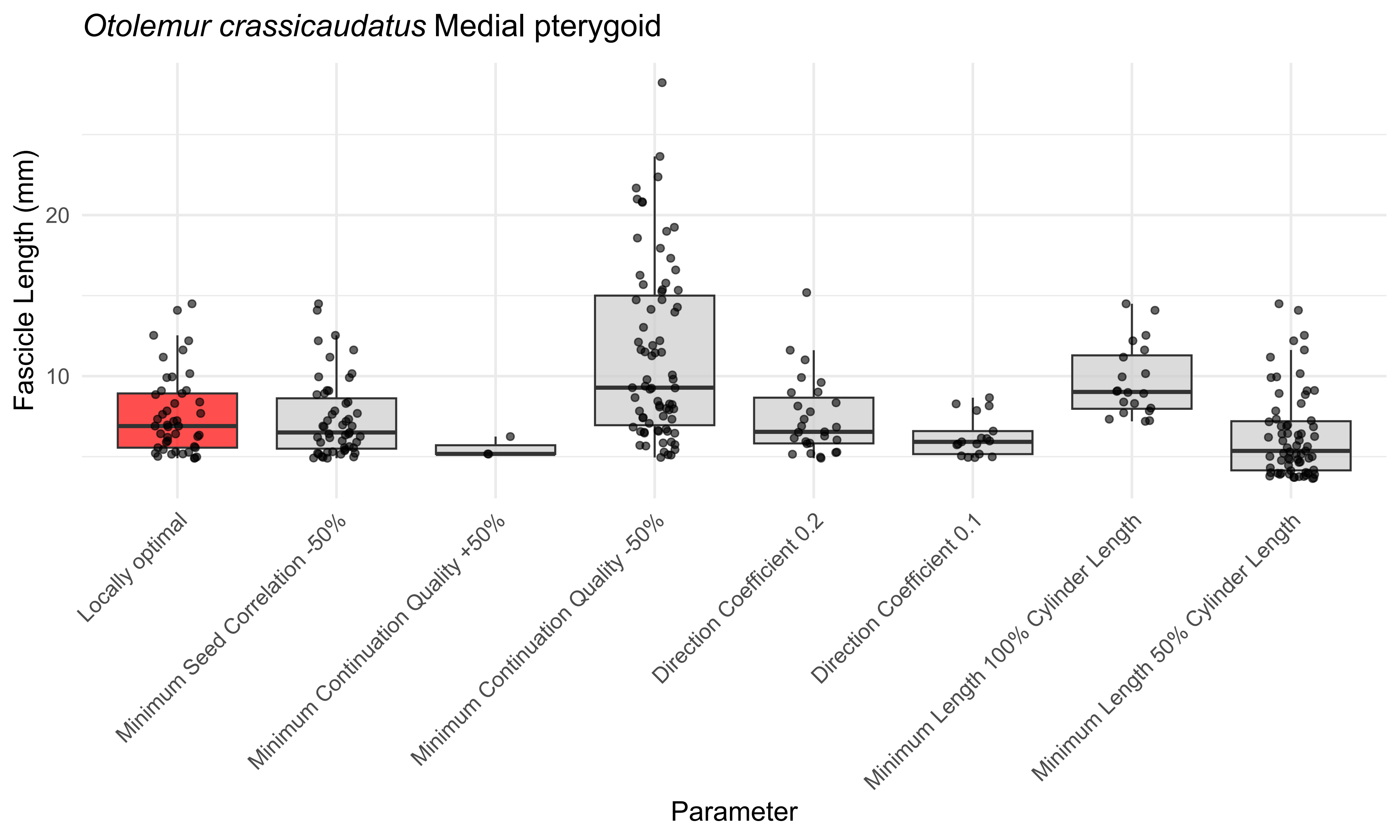

Otolemur crassicaudatus Medial pterygoid
Fascicle Length (mm)
20
10
Locally optimal
Minimum Seed Correlation -50%
Minimum Continuation Quality +50%
Minimum Continuation Quality -50%
Direction Coefficient 0.2
Direction Coefficient 0.1
Minimum Length 100% Cylinder Length
Minimum Length 50% Cylinder Length
Parameter

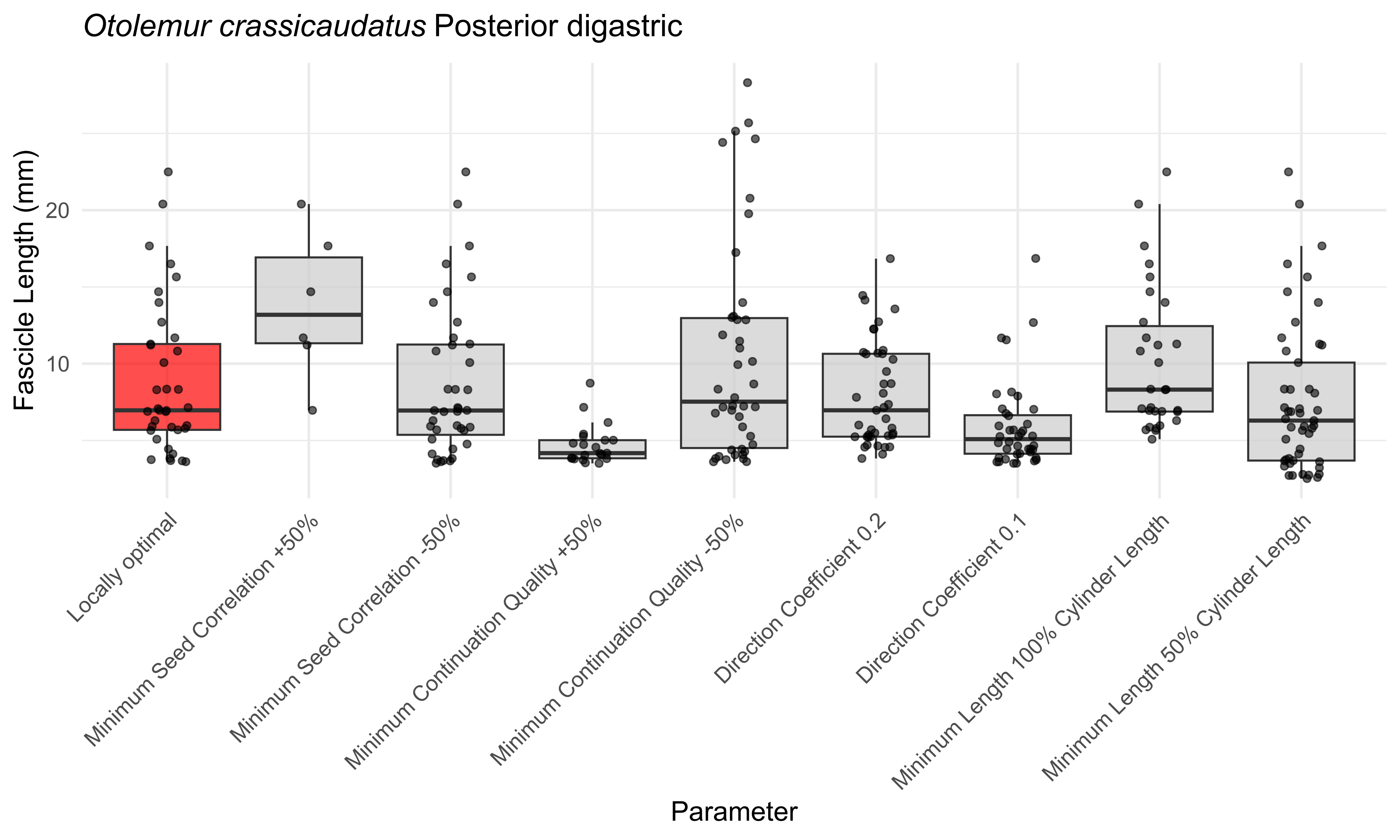

Otolemur crassicaudatus Posterior digastric
Fascicle Length (mm)
20
10
Locally optimal
Minimum Seed Correlation +50%
Minimum Seed Correlation -50%
Minimum Continuation Quality +50%
Minimum Continuation Quality -50%
Direction Coefficient 0.2
Direction Coefficient 0.1
Minimum Length 100% Cylinder Length
Minimum Length 50% Cylinder Length
Parameter

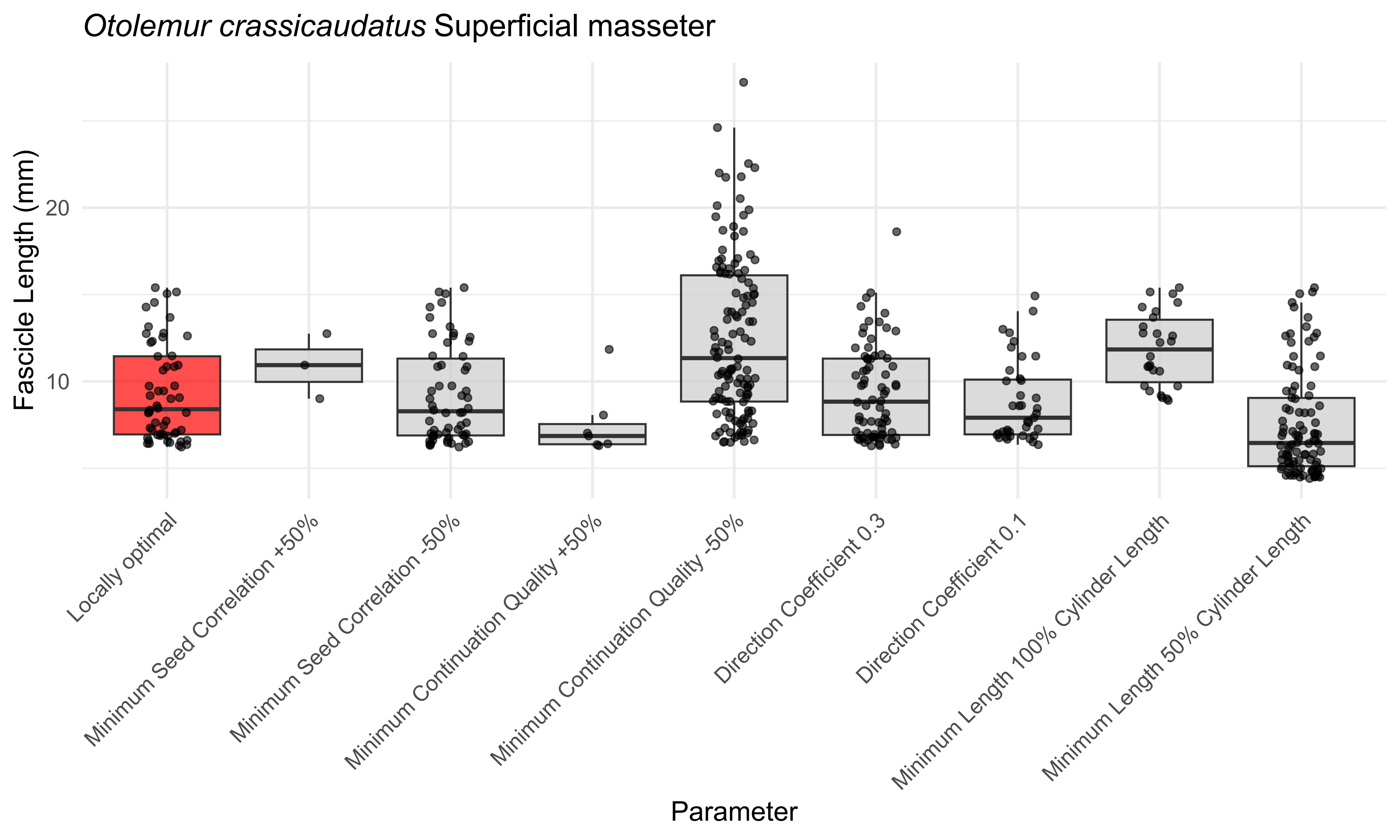

Otolemur crassicaudatus Superficial masseter
Fascicle Length (mm)
20
10
Locally optimal
Minimum Seed Correlation +50%
Minimum Seed Correlation -50%
Minimum Continuation Quality +50%
Minimum Continuation Quality -50%
Direction Coefficient 0.3
Direction Coefficient 0.1
Minimum Length 100% Cylinder Length
Minimum Length 50% Cylinder Length
Parameter

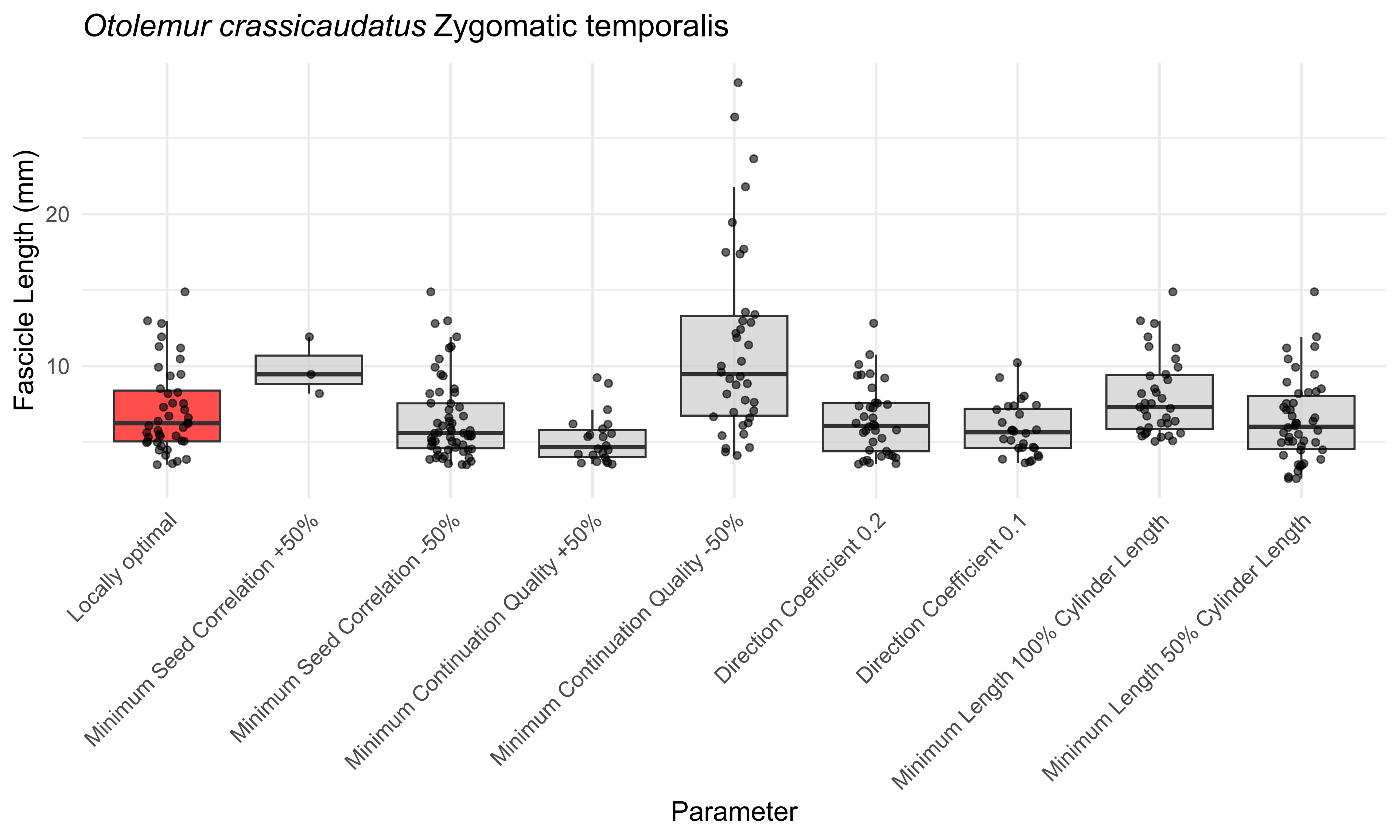

Otolemur crassicaudatus Zygomatic temporalis
Fascicle Length (mm)
20
10
Locally optimal
Minimum Seed Correlation +50%
Minimum Seed Correlation -50%
Minimum Continuation Quality +50%
Minimum Continuation Quality -50%
Direction Coefficient 0.2
Direction Coefficient 0.1
Minimum Length 100% Cylinder Length
Minimum Length 50% Cylinder Length
Parameter

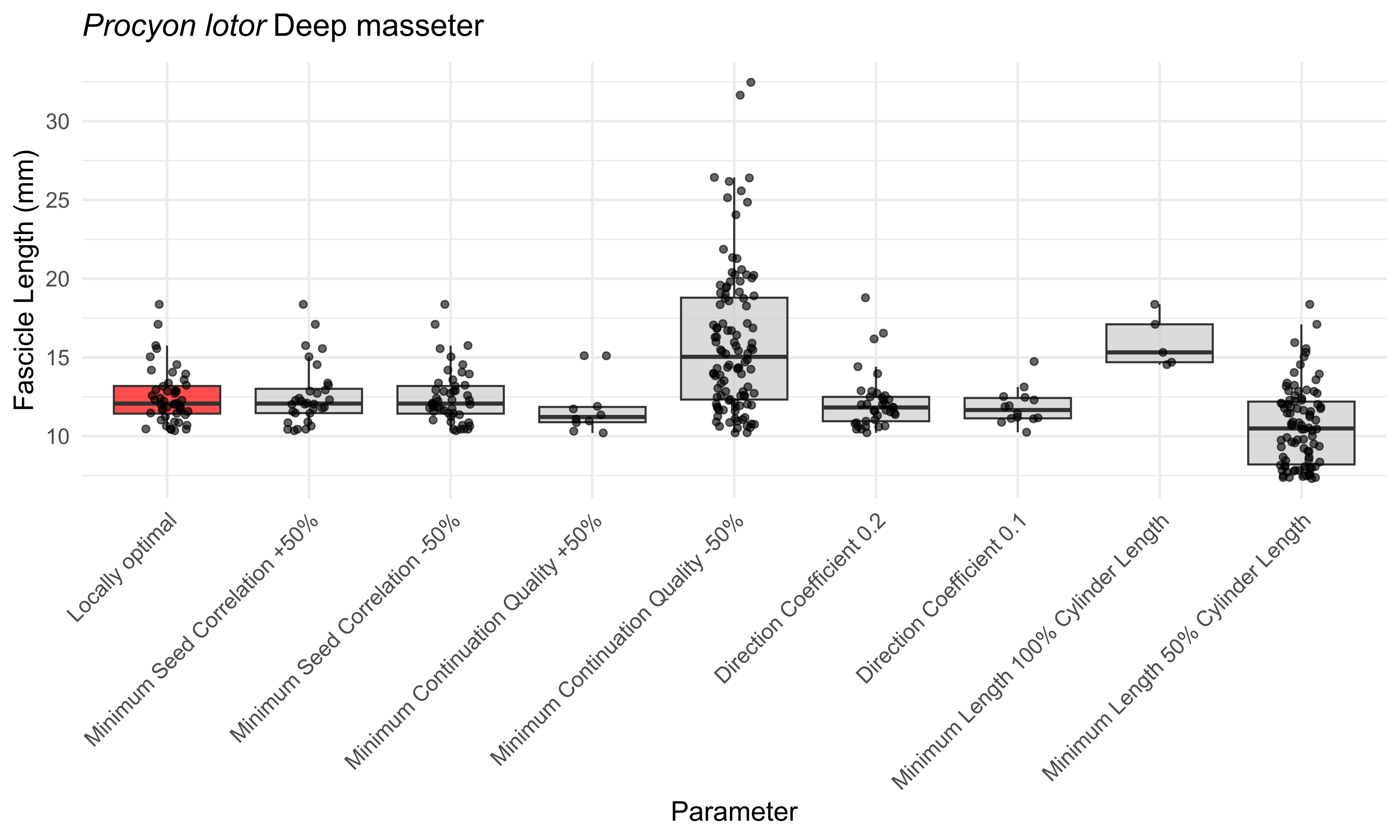

Procyon lotor Deep masseter
Fascicle Length (mm)
30
25
20
15
10
Locally optimal
Minimum Seed Correlation +50%
Minimum Seed Correlation -50%
Minimum Continuation Quality +50%
Minimum Continuation Quality -50%
Direction Coefficient 0.2
Direction Coefficient 0.1
Minimum Length 100% Cylinder Length
Minimum Length 50% Cylinder Length
Parameter

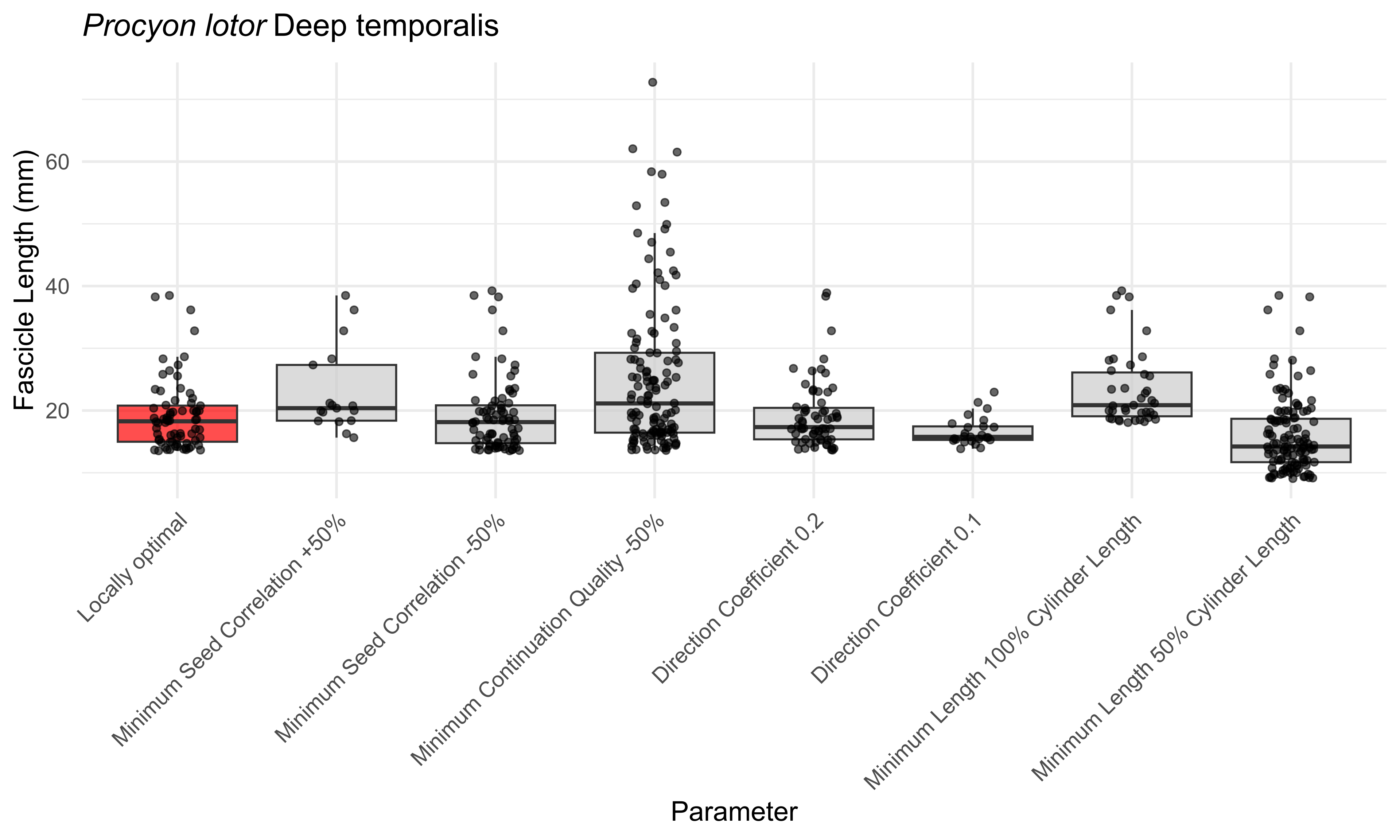

Procyon lotor Deep temporalis
Fascicle Length (mm)
60
40
20
Locally optimal
Minimum Seed Correlation +50%
Minimum Seed Correlation -50%
Minimum Continuation Quality -50%
Direction Coefficient 0.2
Direction Coefficient 0.1
Minimum Length 100% Cylinder Length
Minimum Length 50% Cylinder Length
Parameter

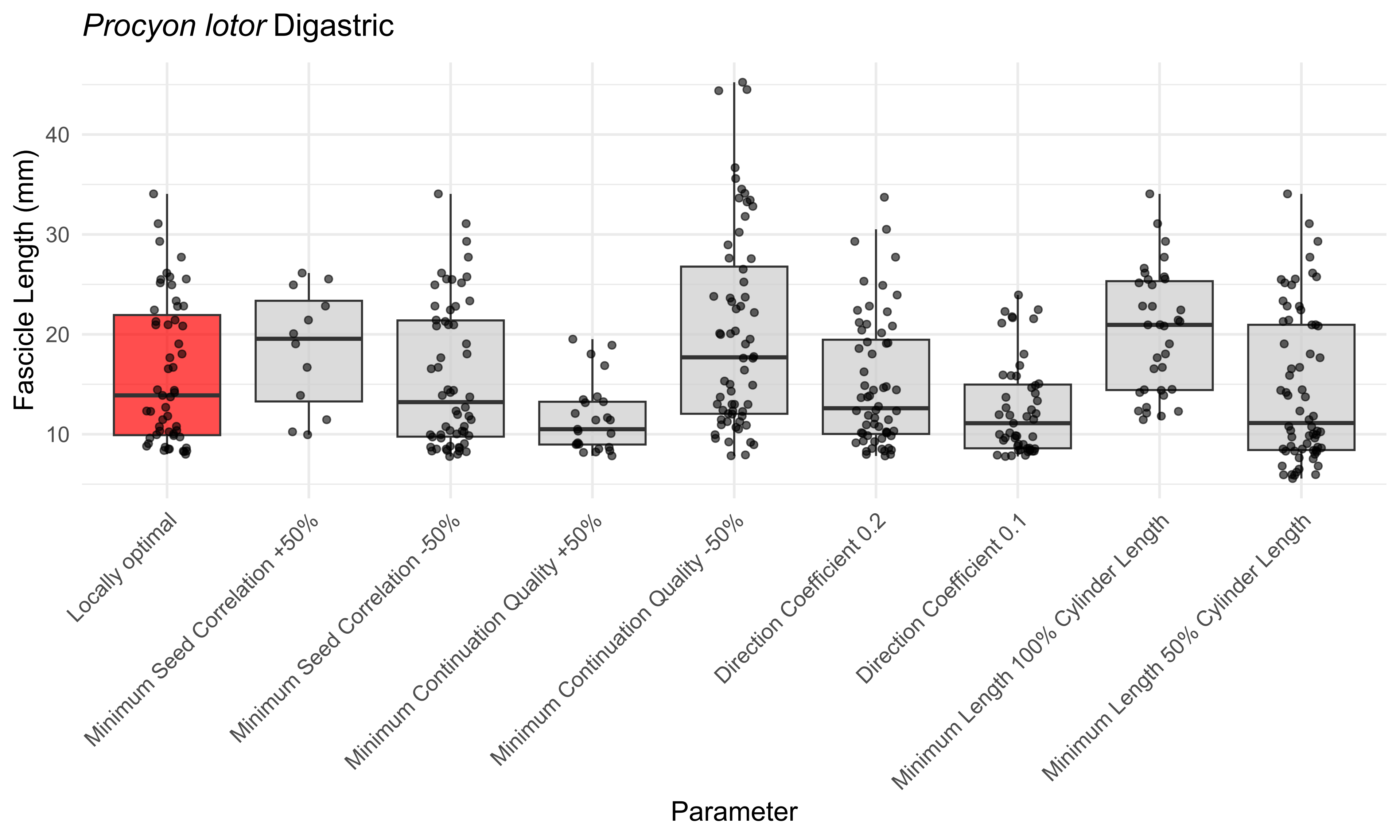
Procyon lotor Digastric
Fascicle Length (mm)
40
30
20
10
Locally optimal
Minimum Seed Correlation +50%
Minimum Seed Correlation -50%
Minimum Continuation Quality +50%
Minimum Continuation Quality -50%
Direction Coefficient 0.2
Direction Coefficient 0.1
Minimum Length 100% Cylinder Length
Minimum Length 50% Cylinder Length
Parameter

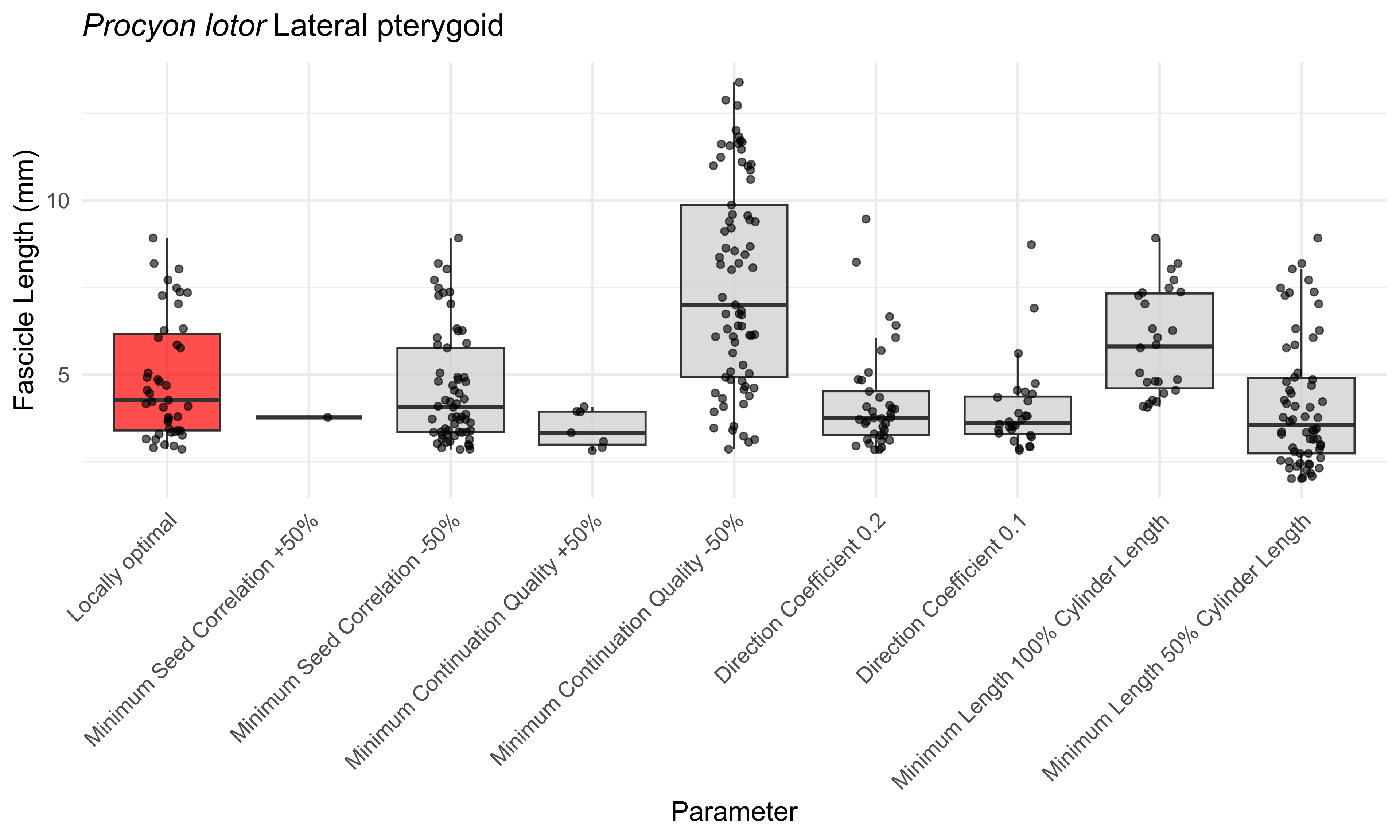

Procyon lotor Lateral pterygoid
Fascicle Length (mm)
10
5
Locally optimal
Minimum Seed Correlation +50%
Minimum Seed Correlation -50%
Minimum Continuation Quality +50%
Minimum Continuation Quality -50%
Direction Coefficient 0.2
Direction Coefficient 0.1
Minimum Length 100% Cylinder Length
Minimum Length 50% Cylinder Length
Parameter

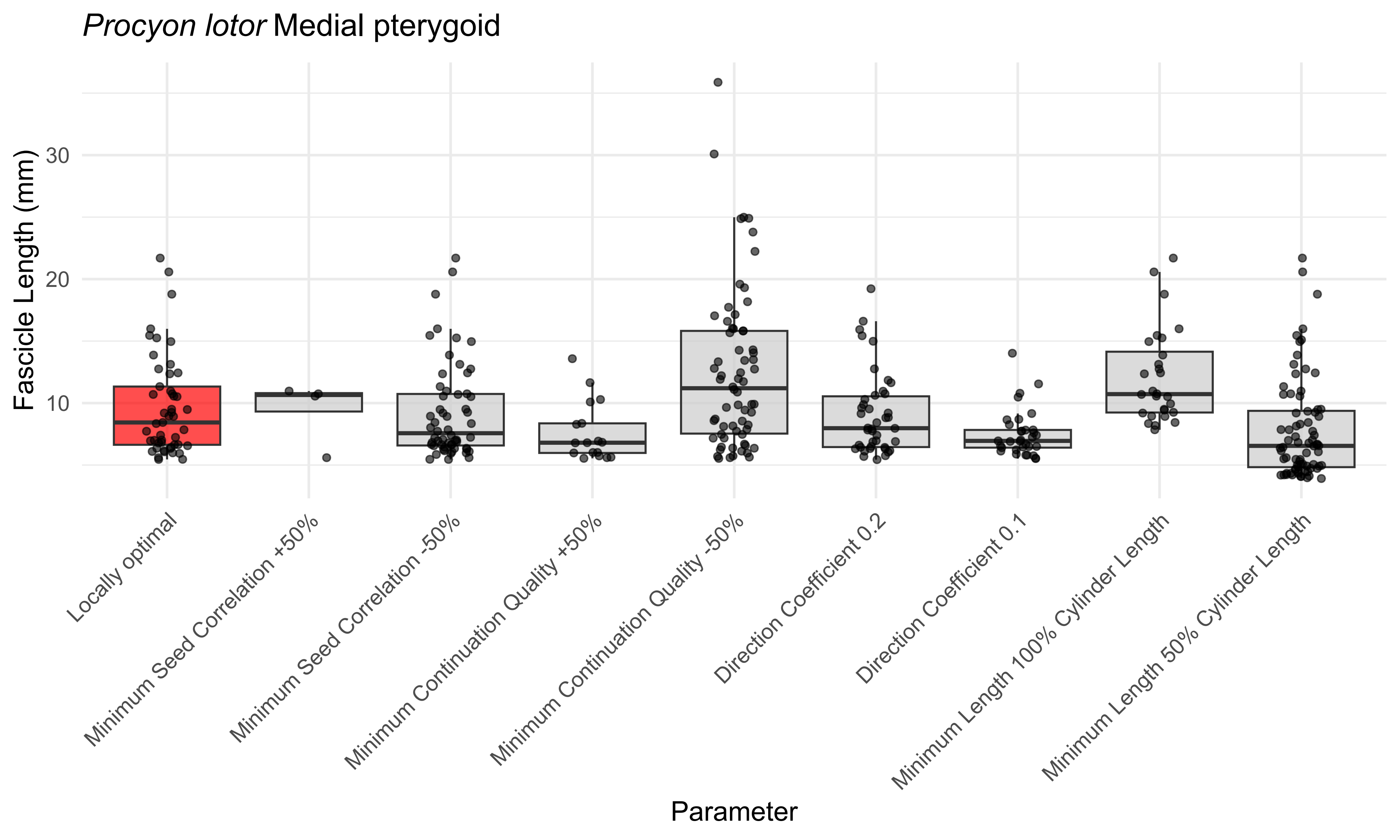

Procyon lotor Medial pterygoid
Fascicle Length (mm)
30
20
10
Locally optimal
Minimum Seed Correlation +50%
Minimum Seed Correlation -50%
Minimum Continuation Quality +50%
Minimum Continuation Quality -50%
Direction Coefficient 0.2
Direction Coefficient 0.1
Minimum Length 100% Cylinder Length
Minimum Length 50% Cylinder Length
Parameter

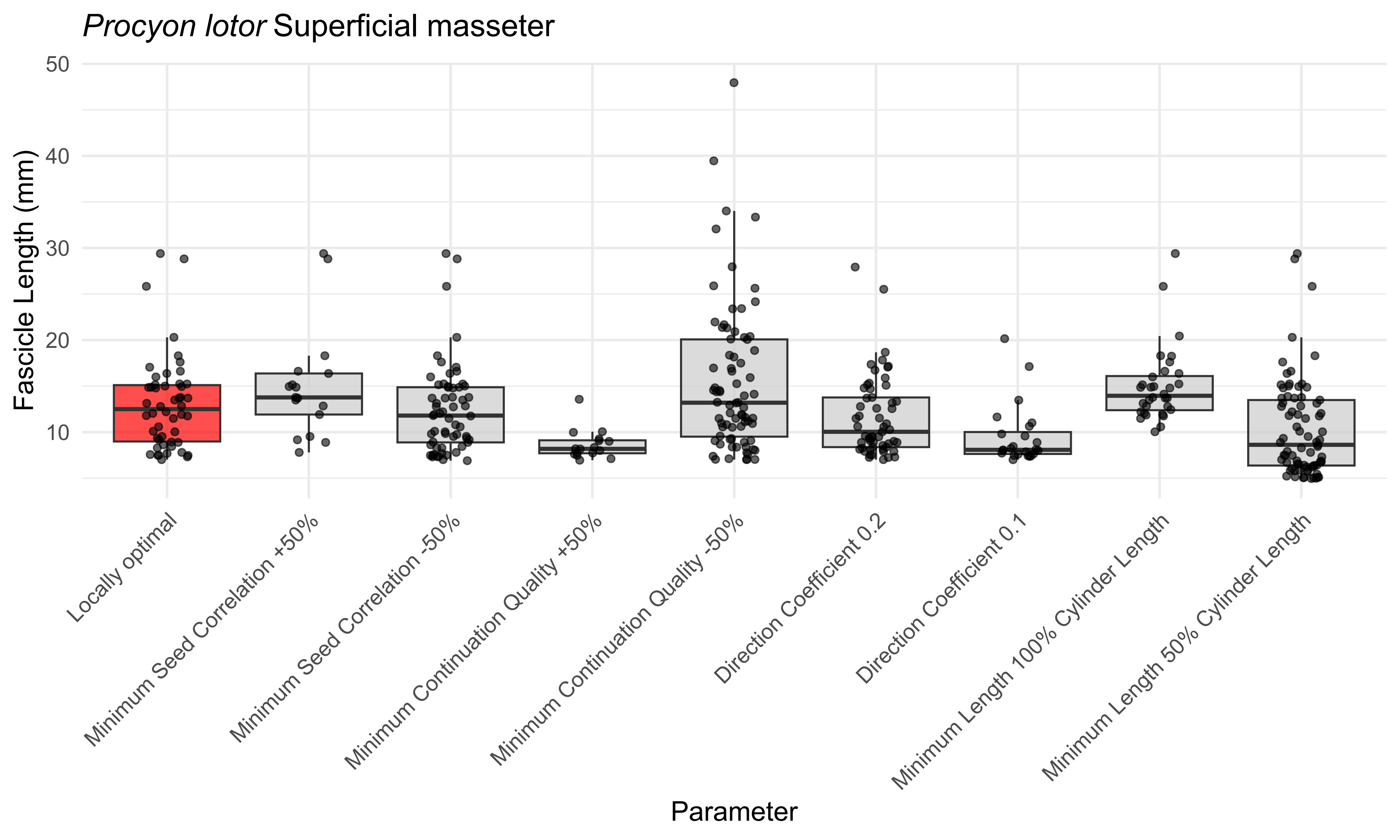

Procyon lotor Superficial masseter
Fascicle Length (mm)
50
40
30
20
10
Locally optimal
Minimum Seed Correlation +50%
Minimum Seed Correlation -50%
Minimum Continuation Quality +50%
Minimum Continuation Quality -50%
Direction Coefficient 0.2
Direction Coefficient 0.1
Minimum Length 100% Cylinder Length
Minimum Length 50% Cylinder Length
Parameter

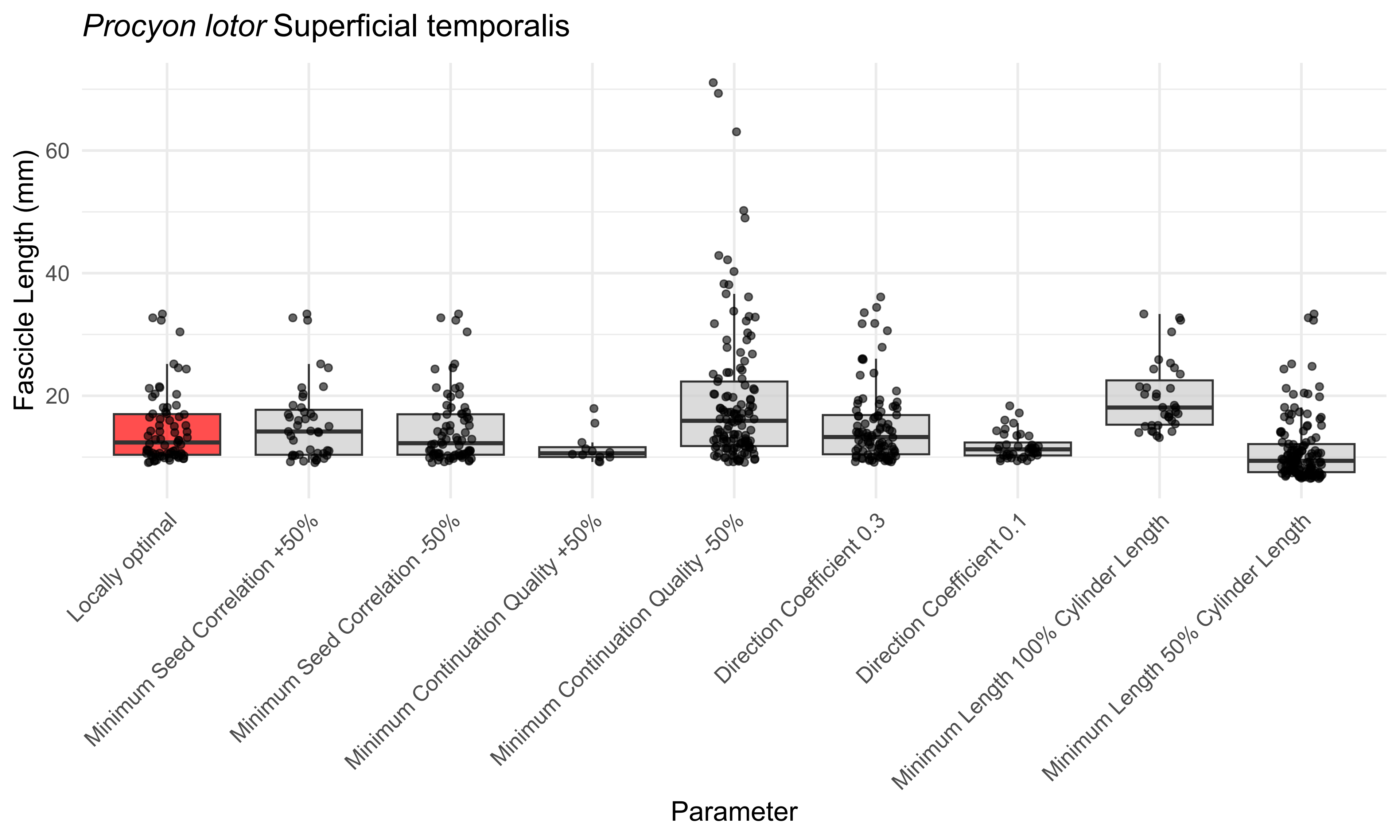
Procyon lotor Superficial temporalis
Fascicle Length (mm)
60
40
20
Locally optimal
Minimum Seed Correlation +50%
Minimum Seed Correlation -50%
Minimum Continuation Quality +50%
Minimum Continuation Quality -50%
Direction Coefficient 0.3
Direction Coefficient 0.1
Minimum Length 100% Cylinder Length
Minimum Length 50% Cylinder Length
Parameter

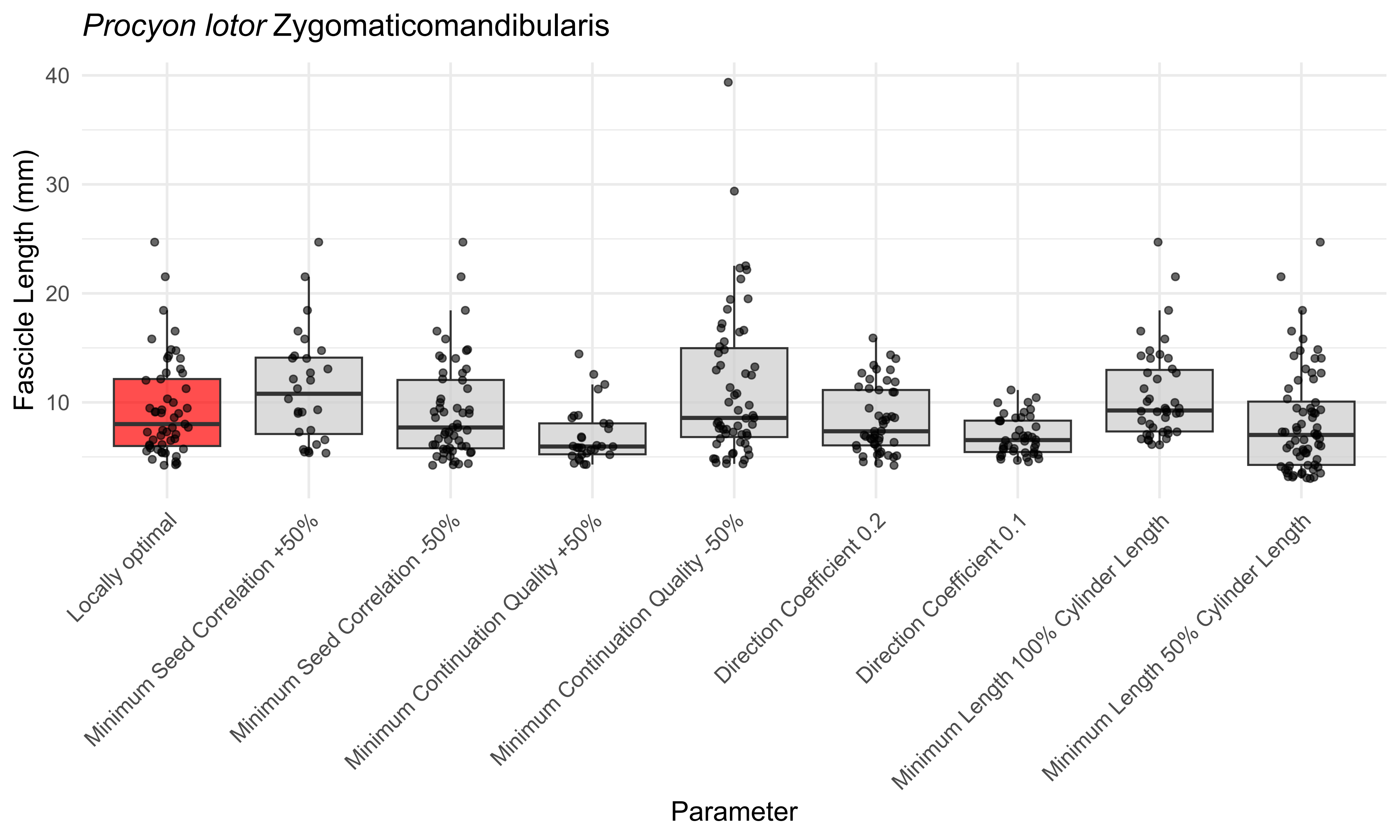

Procyon lotor Zygomaticomandibularis
Fascicle Length (mm)
40
30
20
10
Locally optimal
Minimum Seed Correlation +50%
Minimum Seed Correlation -50%
Minimum Continuation Quality +50%
Minimum Continuation Quality -50%
Direction Coefficient 0.2
Direction Coefficient 0.1
Minimum Length 100% Cylinder Length
Minimum Length 50% Cylinder Length
Parameter
