# Supplementary material for: Multi‐method analysis for the three‐dimensional reconstruction of muscle fascicles from DiceCT datasets
Source: Anat Rec (Hoboken). 2025 Dec 4;309(12):3491–510. doi: 10.1002/ar.70088 (PMC13749328; doi:10.1002/ar.70088)
Supplement: Supplementary file 2 [file AR-309-3491-s002.pdf]

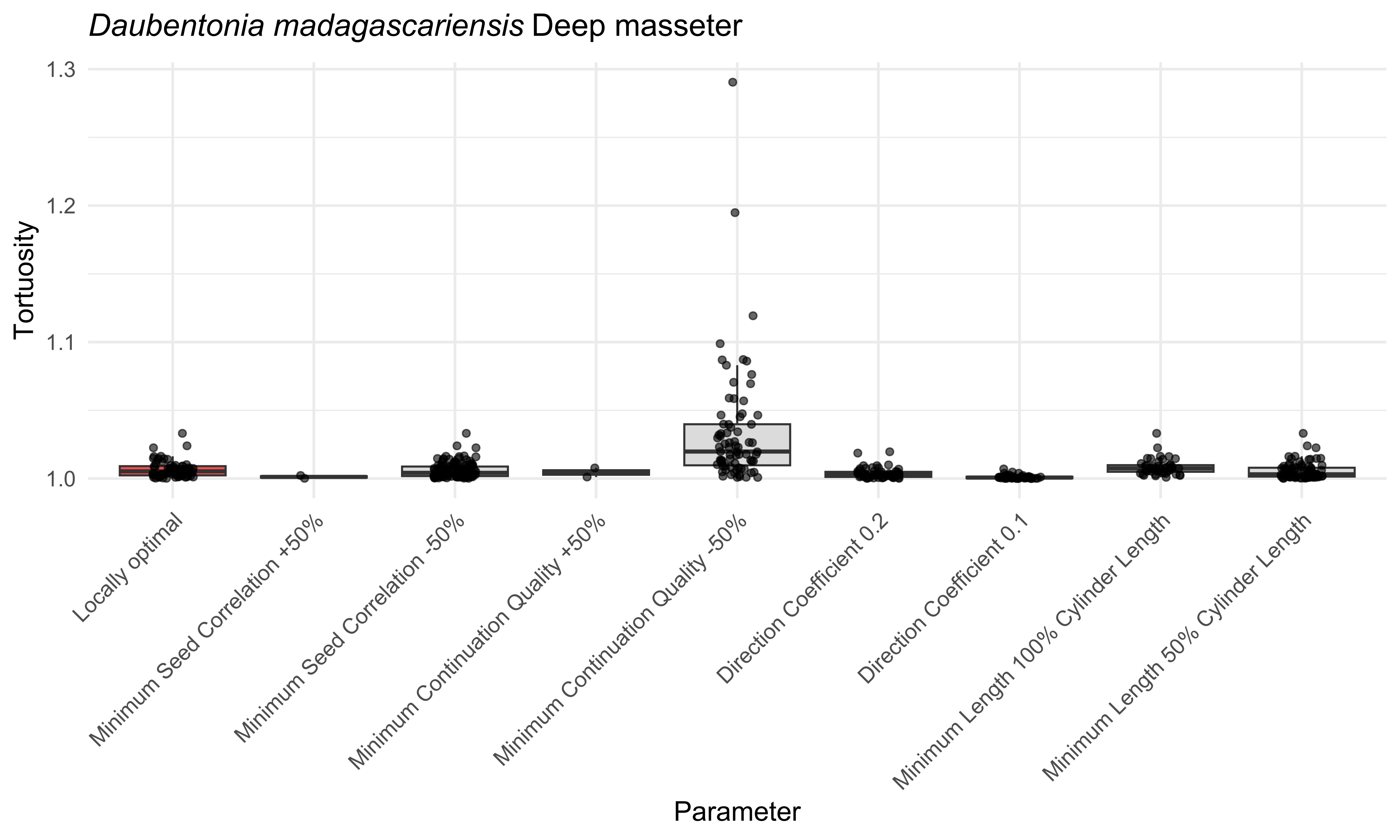

Daubentonia madagascariensis Deep masseter
Tortuosity
1.3
1.2
1.1
1.0
Locally optimal
Minimum Seed Correlation +50%
Minimum Seed Correlation -50%
Minimum Continuation Quality +50%
Minimum Continuation Quality -50%
Direction Coefficient 0.2
Direction Coefficient 0.1
Minimum Length 100% Cylinder Length
Minimum Length 50% Cylinder Length
Parameter

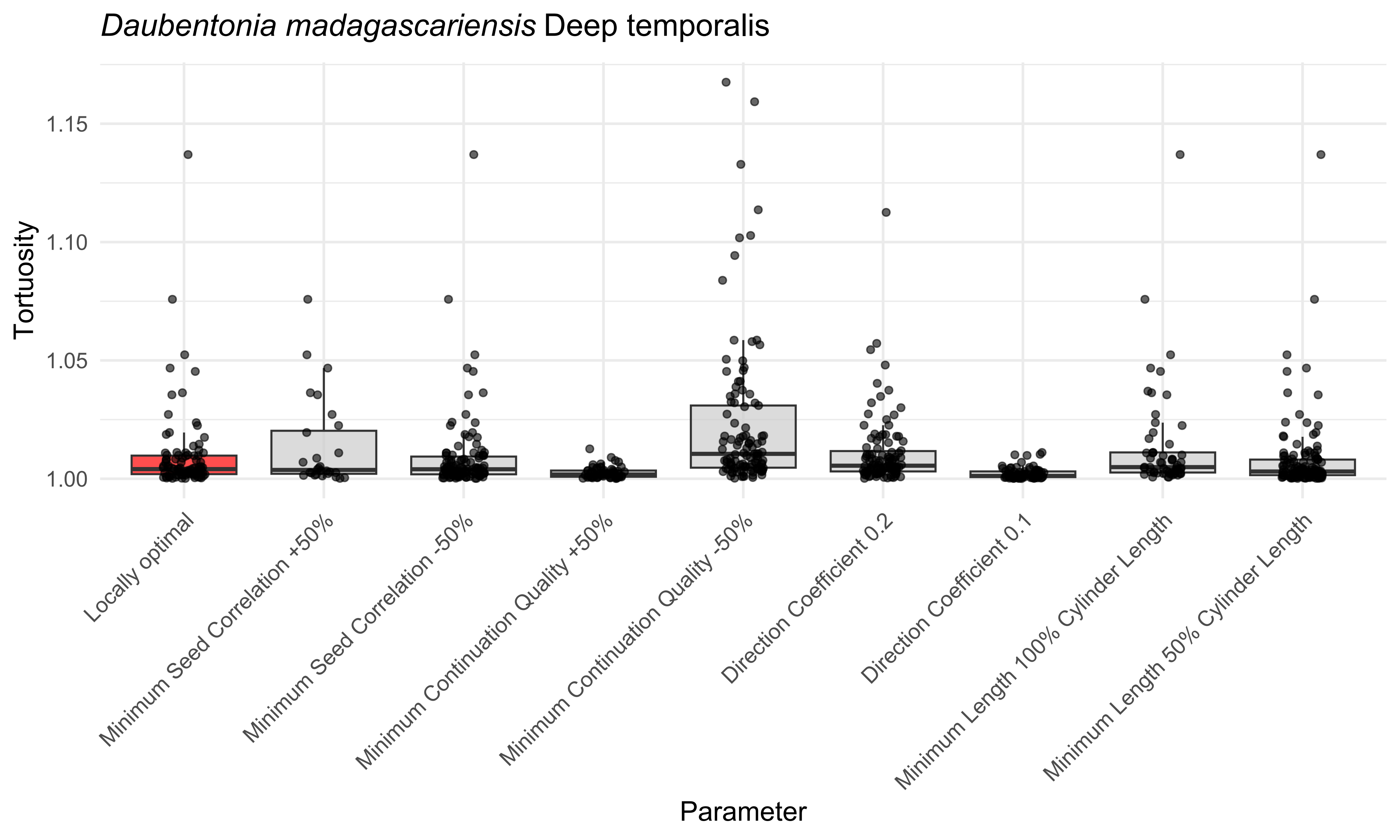

Daubentonia madagascariensis Deep temporalis
Tortuosity
1.15
1.10
1.05
1.00
Locally optimal
Minimum Seed Correlation +50%
Minimum Seed Correlation -50%
Minimum Continuation Quality +50%
Minimum Continuation Quality -50%
Direction Coefficient 0.2
Direction Coefficient 0.1
Minimum Length 100% Cylinder Length
Minimum Length 50% Cylinder Length
Parameter

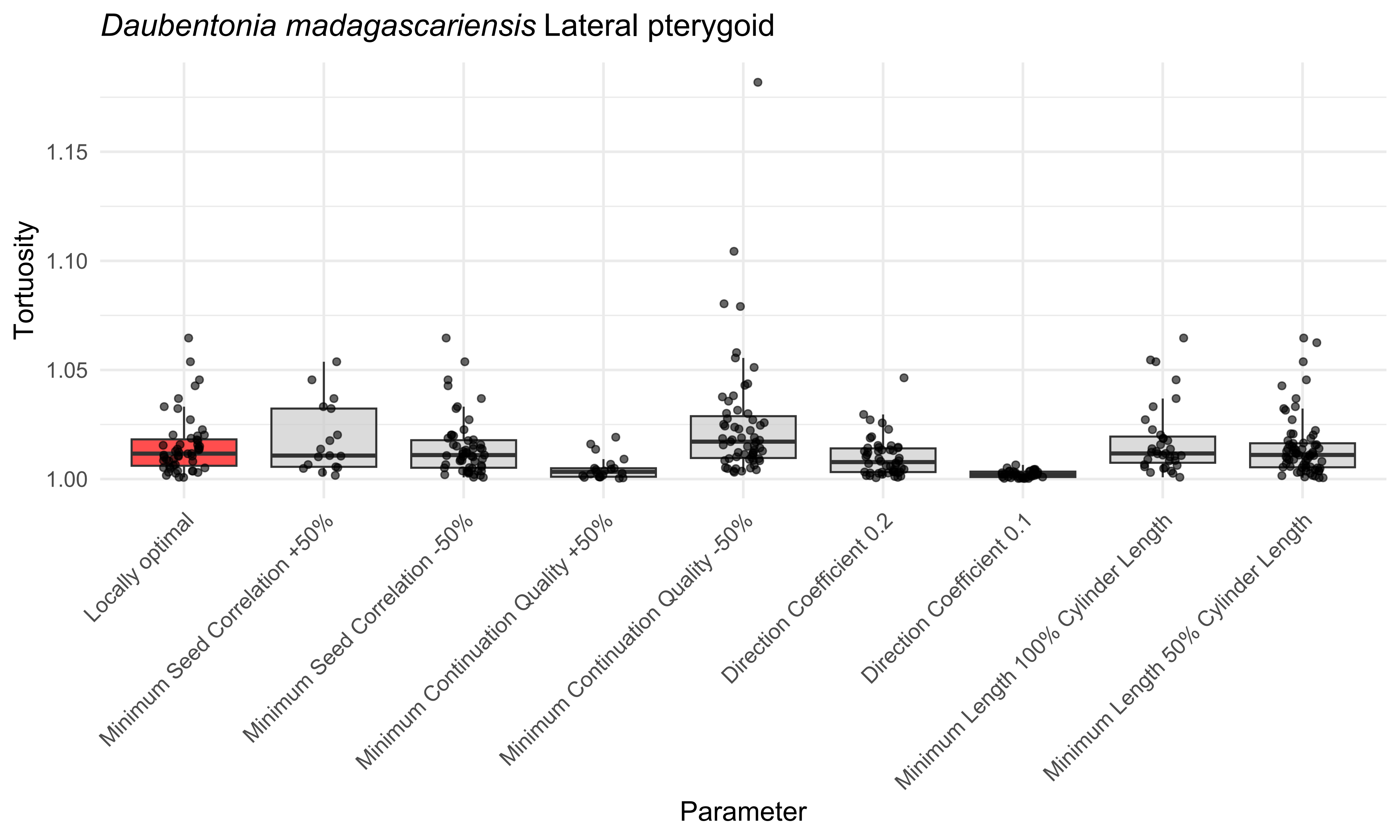
Daubentonia madagascariensis Lateral pterygoid
Tortuosity
1.15
1.10
1.05
1.00
Locally optimal
Minimum Seed Correlation +50%
Minimum Seed Correlation -50%
Minimum Continuation Quality +50%
Minimum Continuation Quality -50%
Direction Coefficient 0.2
Direction Coefficient 0.1
Minimum Length 100% Cylinder Length
Minimum Length 50% Cylinder Length
Parameter

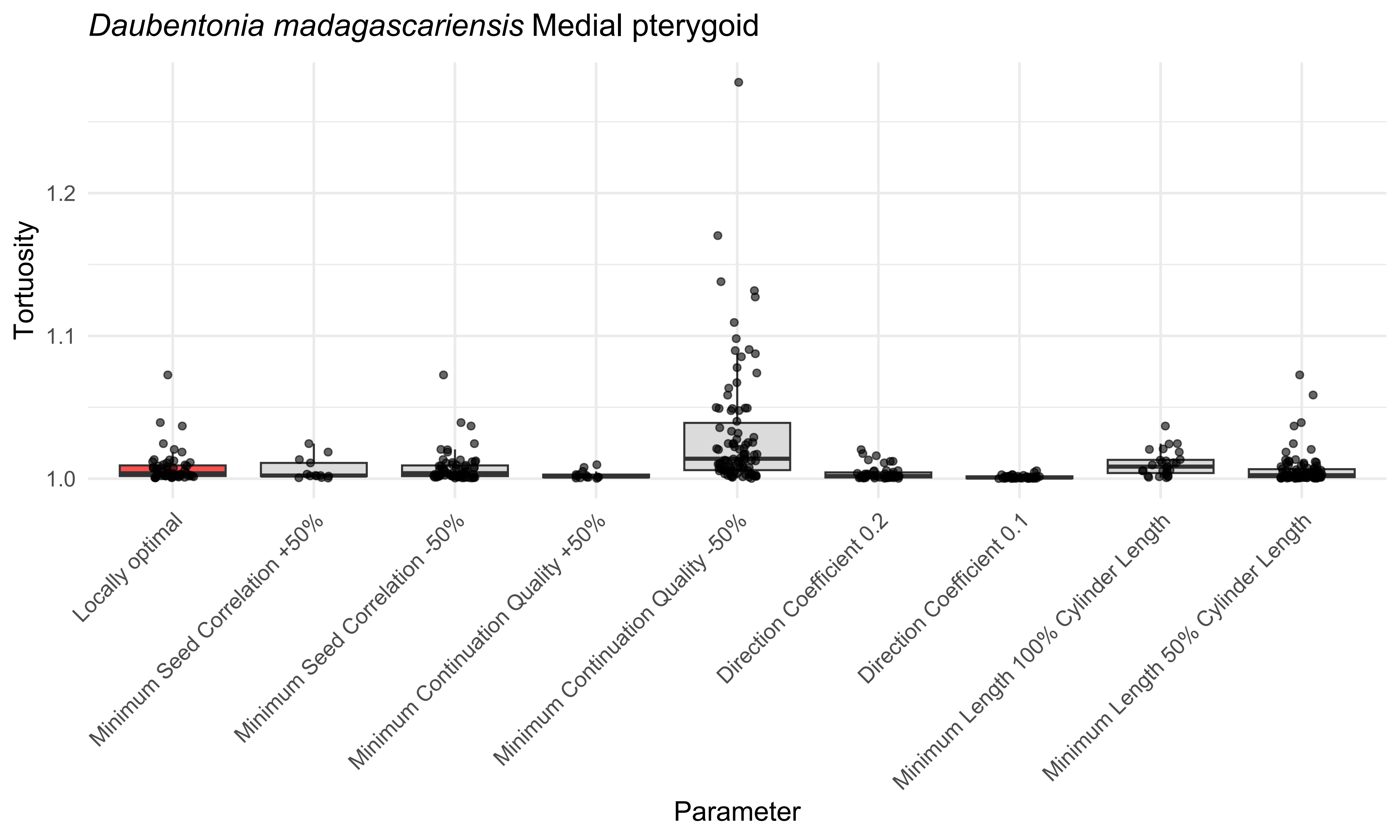

Daubentonia madagascariensis Medial pterygoid
Tortuosity
1.0
1.1
1.2
Locally optimal
Minimum Seed Correlation +50%
Minimum Seed Correlation -50%
Minimum Continuation Quality +50%
Minimum Continuation Quality -50%
Direction Coefficient 0.2
Direction Coefficient 0.1
Minimum Length 100% Cylinder Length
Minimum Length 50% Cylinder Length
Parameter

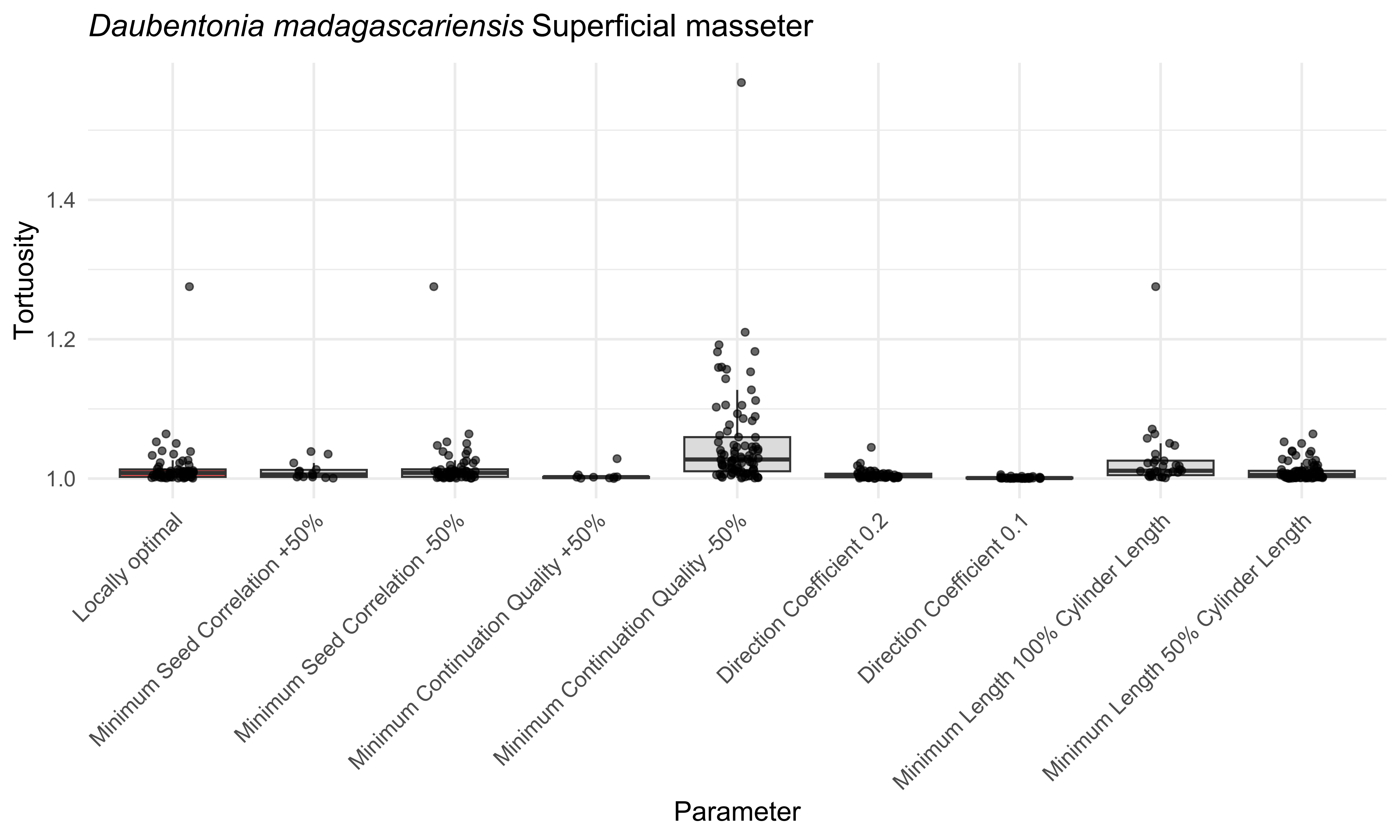

Daubentonia madagascariensis Superficial masseter
Tortuosity
1.4
1.2
1.0
Parameter
Locally optimal
Minimum Seed Correlation +50%
Minimum Seed Correlation -50%
Minimum Continuation Quality +50%
Minimum Continuation Quality -50%
Direction Coefficient 0.2
Direction Coefficient 0.1
Minimum Length 100% Cylinder Length
Minimum Length 50% Cylinder Length

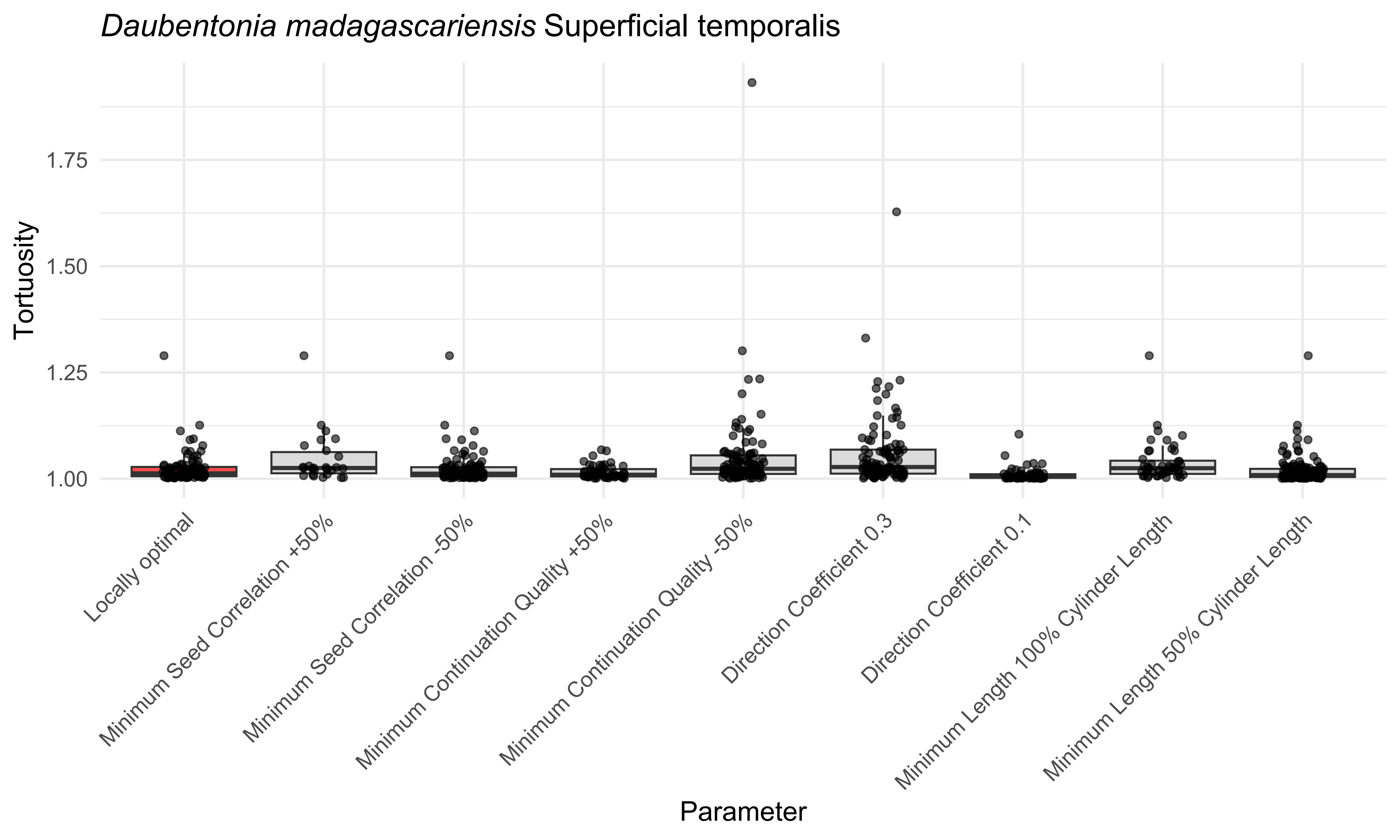

Daubentonia madagascariensis Superficial temporalis
Tortuosity
1.00
1.25
1.50
1.75
Parameter
Locally optimal
Minimum Seed Correlation +50%
Minimum Seed Correlation -50%
Minimum Continuation Quality +50%
Minimum Continuation Quality -50%
Direction Coefficient 0.3
Direction Coefficient 0.1
Minimum Length 100% Cylinder Length
Minimum Length 50% Cylinder Length

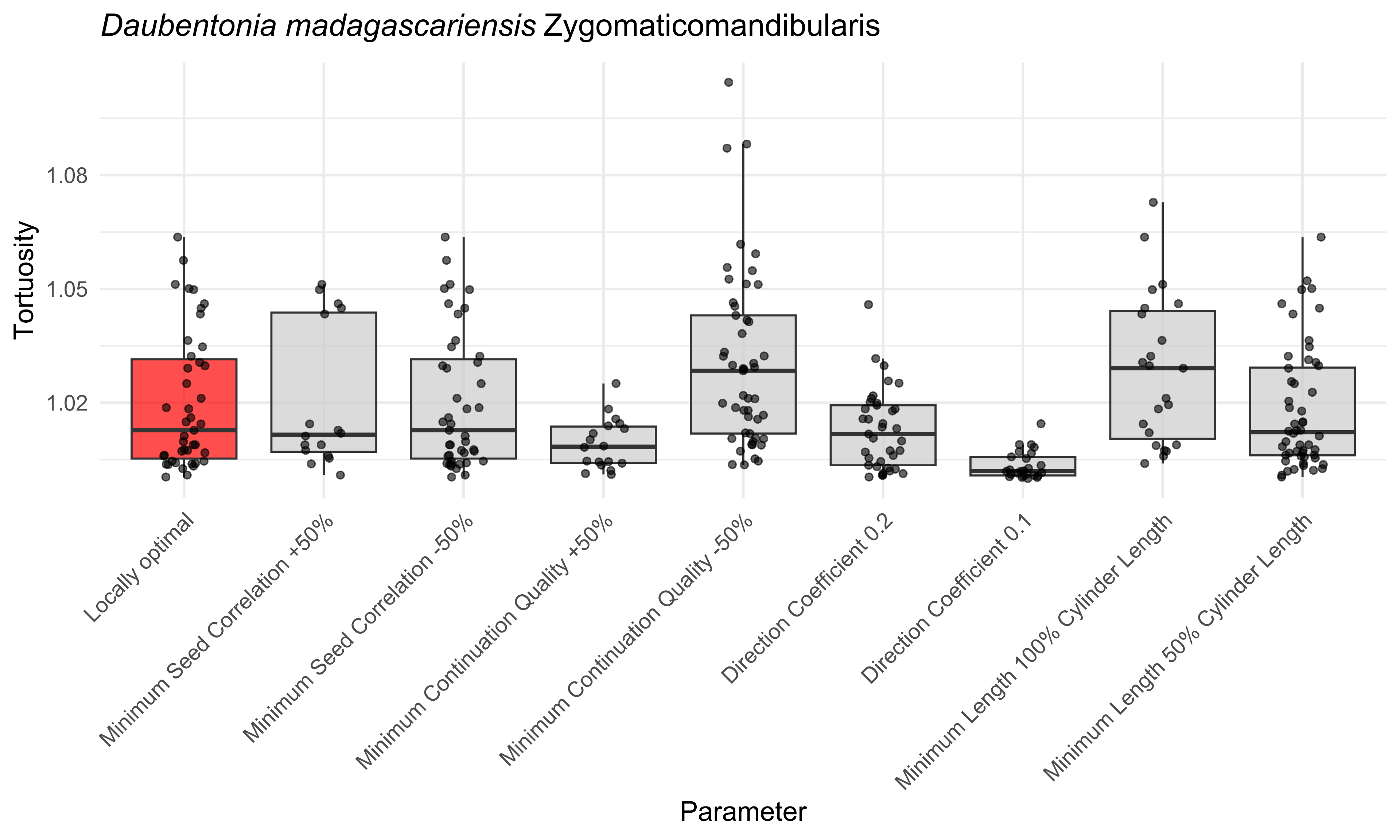

Daubentonia madagascariensis Zygomaticomandibularis
Tortuosity
1.08
1.05
1.02
Parameter
Locally optimal
Minimum Seed Correlation +50%
Minimum Seed Correlation -50%
Minimum Continuation Quality +50%
Minimum Continuation Quality -50%
Direction Coefficient 0.2
Direction Coefficient 0.1
Minimum Length 100% Cylinder Length
Minimum Length 50% Cylinder Length

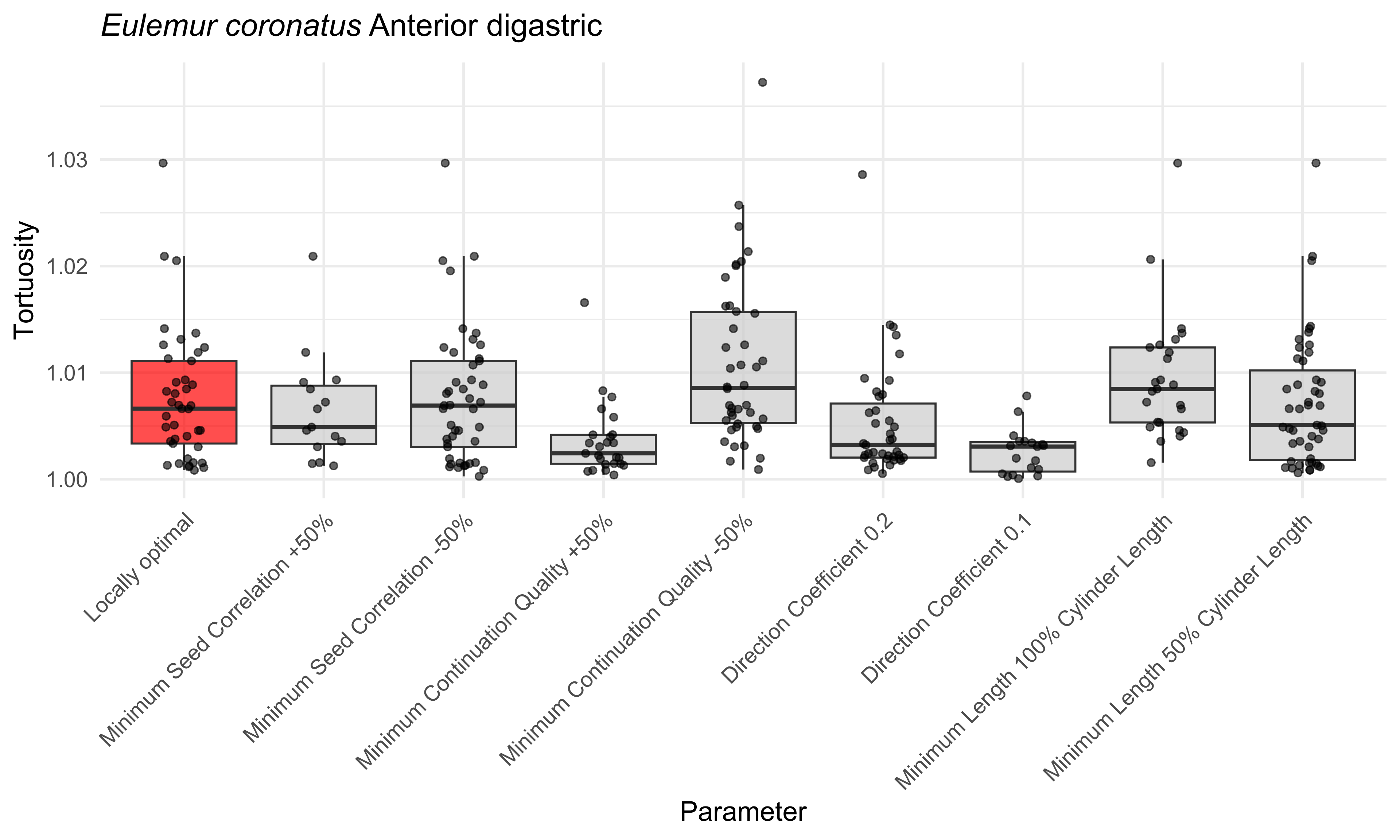

Eulemur coronatus Anterior digastric
Tortuosity
1.03
1.02
1.01
1.00
Locally optimal
Minimum Seed Correlation +50%
Minimum Seed Correlation -50%
Minimum Continuation Quality +50%
Minimum Continuation Quality -50%
Direction Coefficient 0.2
Direction Coefficient 0.1
Minimum Length 100% Cylinder Length
Minimum Length 50% Cylinder Length
Parameter

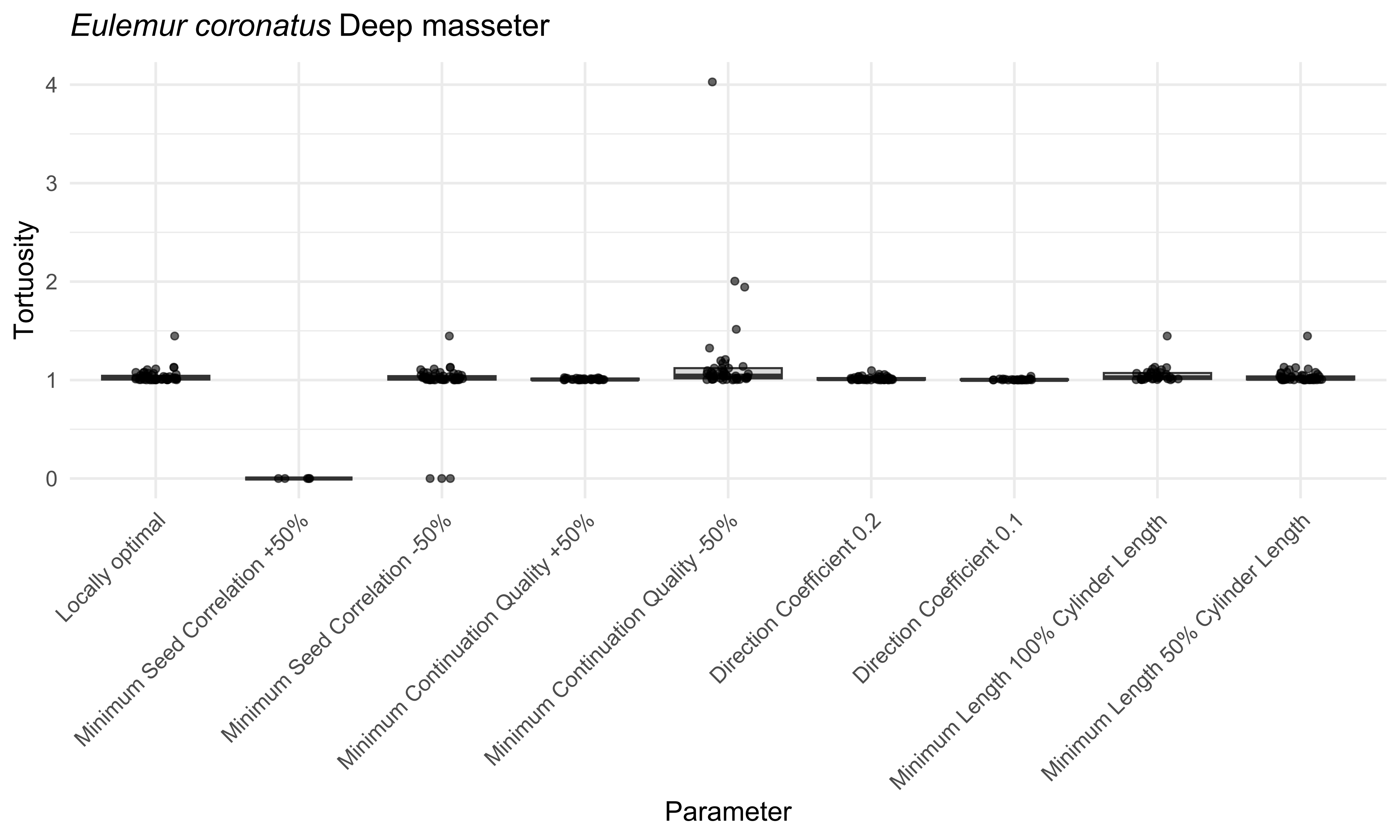

Eulemur coronatus Deep masseter
Tortuosity
4
3
2
1
0
Locally optimal
Minimum Seed Correlation +50%
Minimum Seed Correlation -50%
Minimum Continuation Quality +50%
Minimum Continuation Quality -50%
Direction Coefficient 0.2
Direction Coefficient 0.1
Minimum Length 100% Cylinder Length
Minimum Length 50% Cylinder Length
Parameter

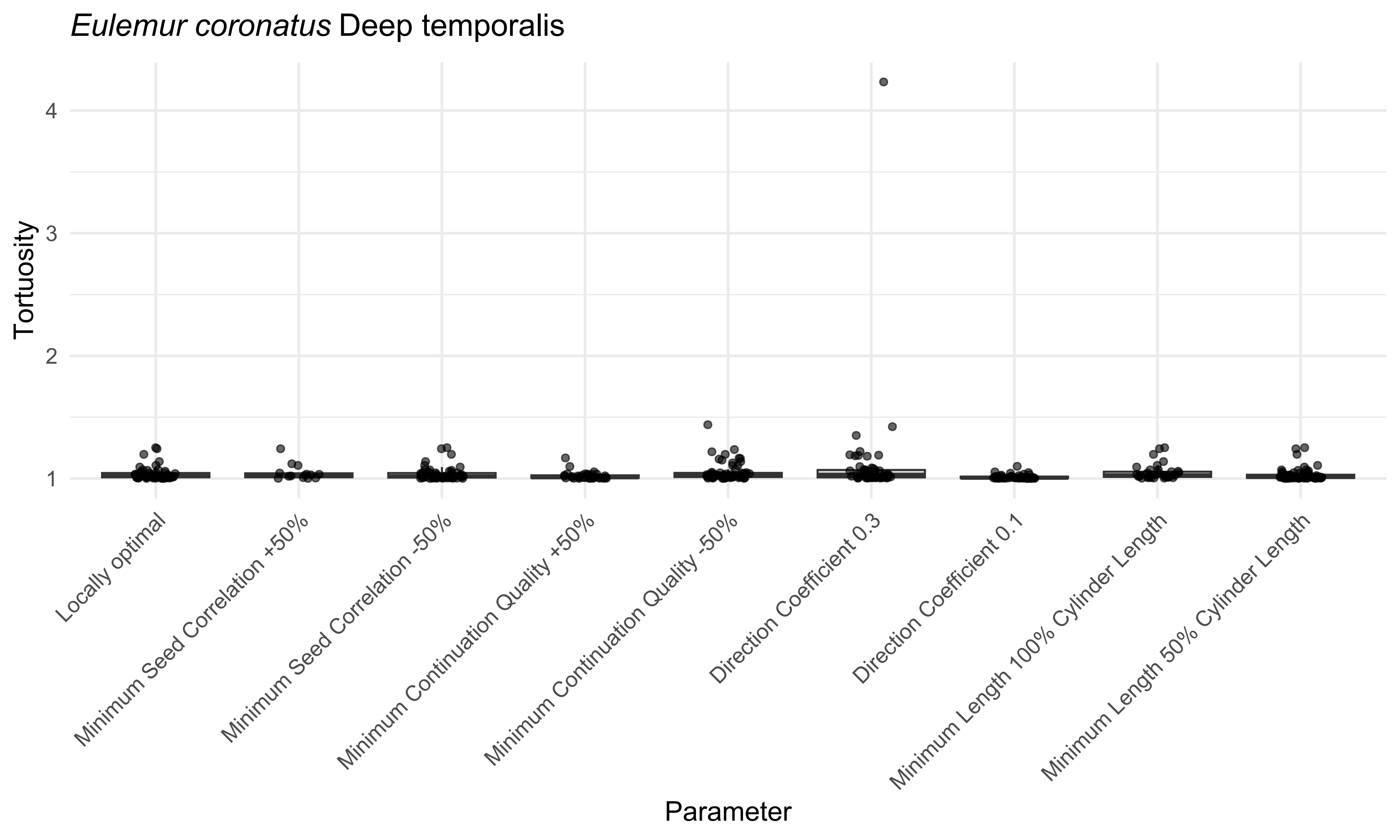

Eulemur coronatus Deep temporalis
Tortuosity
1
2
3
4
Locally optimal
Minimum Seed Correlation +50%
Minimum Seed Correlation -50%
Minimum Continuation Quality +50%
Minimum Continuation Quality -50%
Direction Coefficient 0.3
Direction Coefficient 0.1
Minimum Length 100% Cylinder Length
Minimum Length 50% Cylinder Length
Parameter

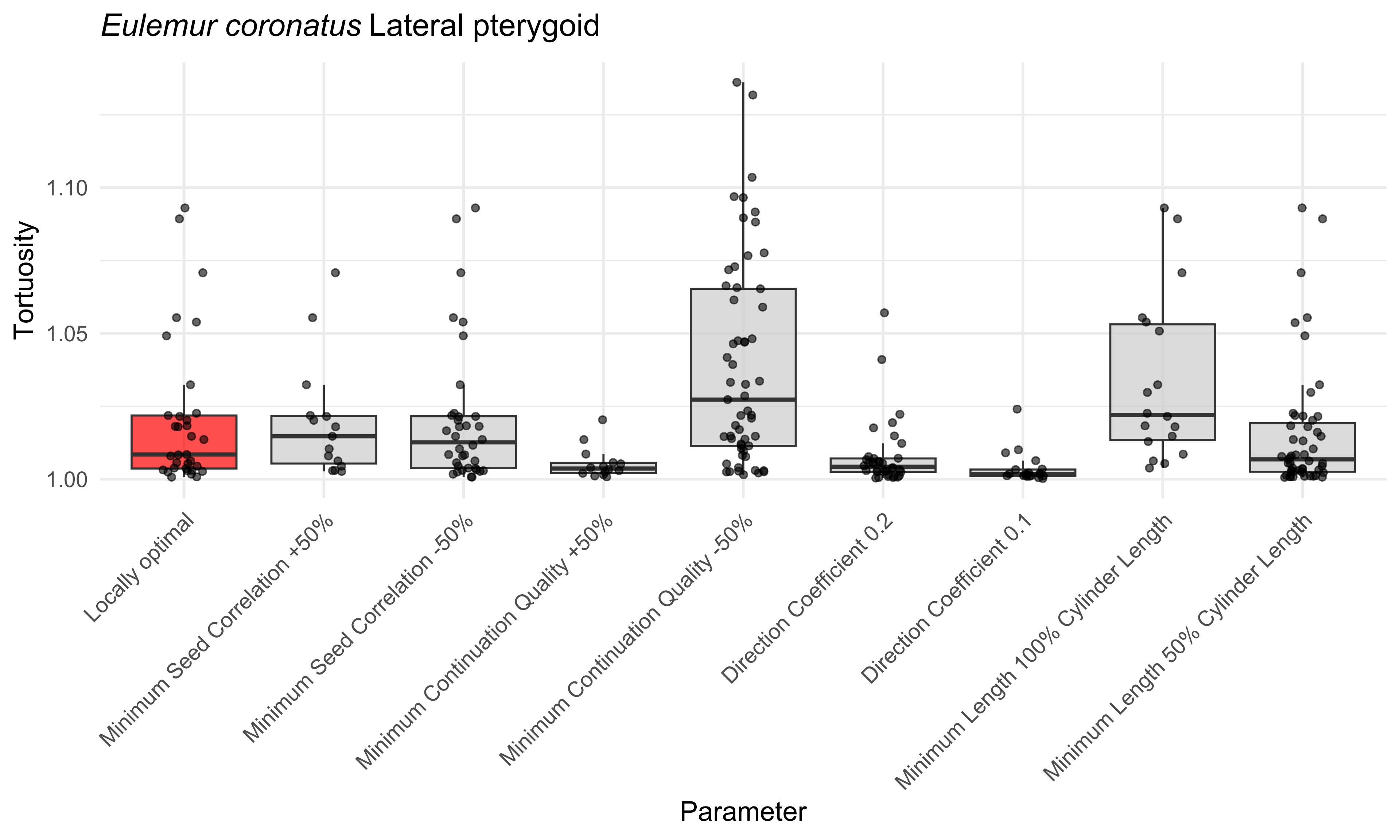

Eulemur coronatus Lateral pterygoid
Tortuosity
1.10
1.05
1.00
Parameter
Locally optimal
Minimum Seed Correlation +50%
Minimum Seed Correlation -50%
Minimum Continuation Quality +50%
Minimum Continuation Quality -50%
Direction Coefficient 0.2
Direction Coefficient 0.1
Minimum Length 100% Cylinder Length
Minimum Length 50% Cylinder Length

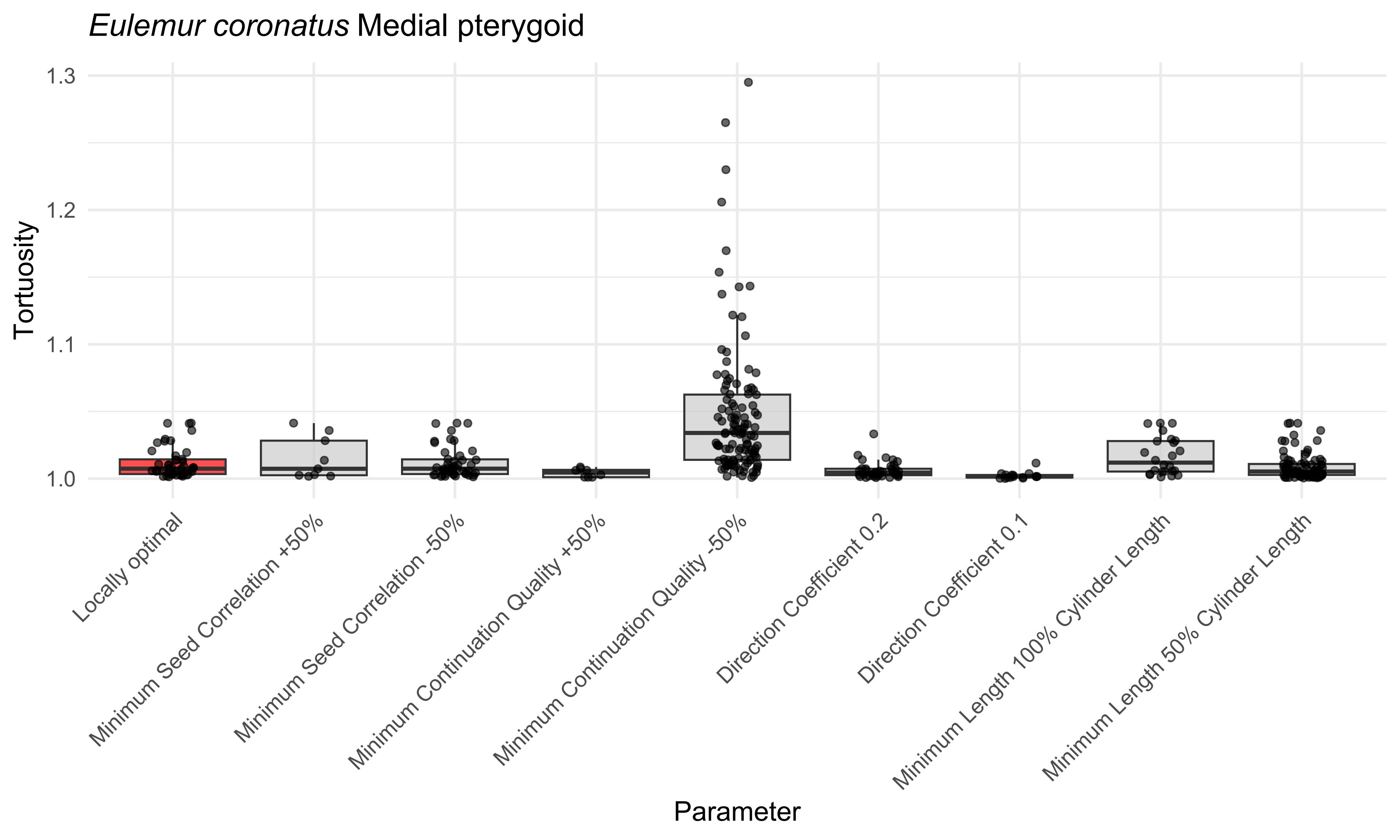

Eulemur coronatus Medial pterygoid
Tortuosity
1.3
1.2
1.1
1.0
Locally optimal
Minimum Seed Correlation +50%
Minimum Seed Correlation -50%
Minimum Continuation Quality +50%
Minimum Continuation Quality -50%
Direction Coefficient 0.2
Direction Coefficient 0.1
Minimum Length 100% Cylinder Length
Minimum Length 50% Cylinder Length
Parameter

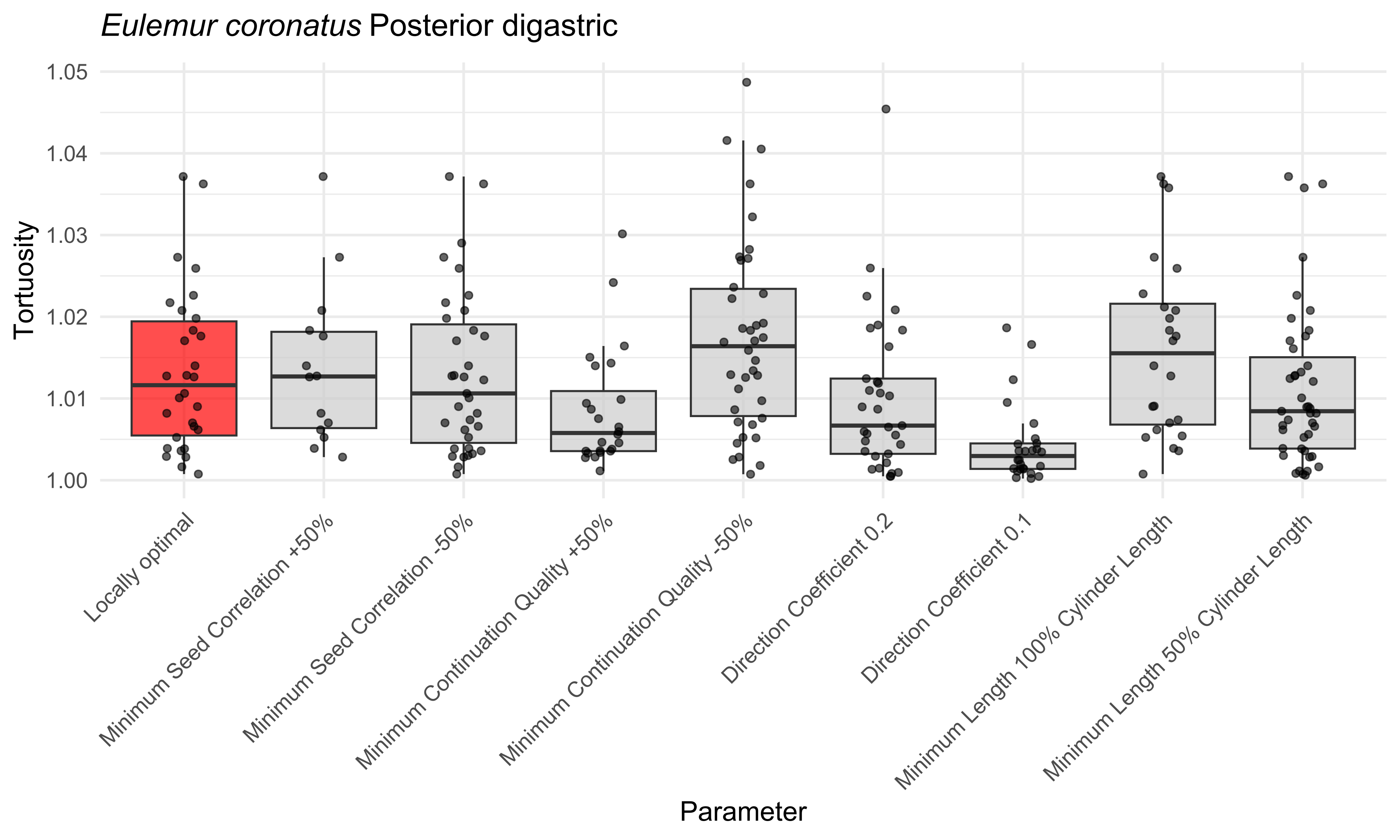

Eulemur coronatus Posterior digastric
Tortuosity
1.05
1.04
1.03
1.02
1.01
1.00
Locally optimal
Minimum Seed Correlation +50%
Minimum Seed Correlation -50%
Minimum Continuation Quality +50%
Minimum Continuation Quality -50%
Direction Coefficient 0.2
Direction Coefficient 0.1
Minimum Length 100% Cylinder Length
Minimum Length 50% Cylinder Length
Parameter

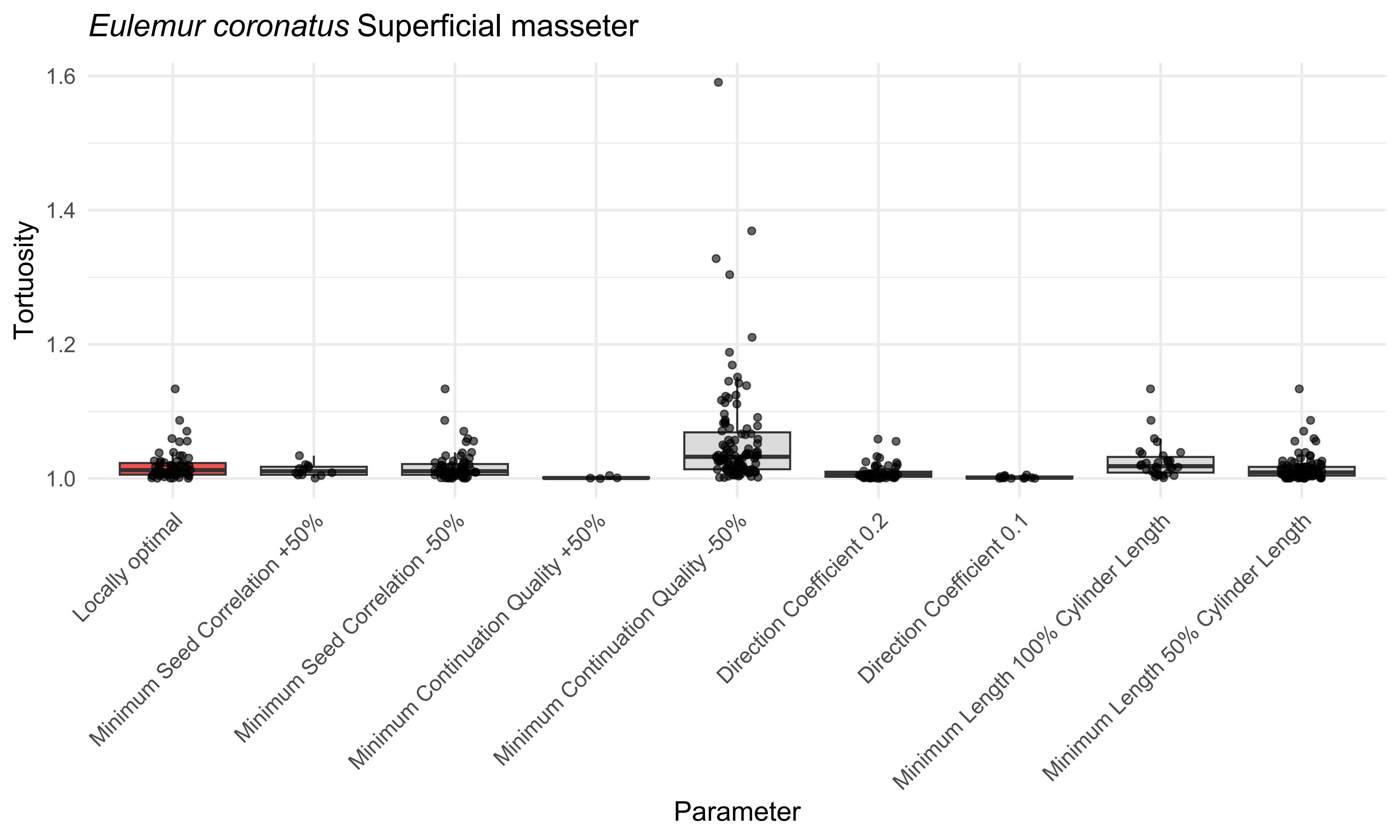
Eulemur coronatus Superficial masseter
Tortuosity
1.6
1.4
1.2
1.0
Locally optimal
Minimum Seed Correlation +50%
Minimum Seed Correlation -50%
Minimum Continuation Quality +50%
Minimum Continuation Quality -50%
Direction Coefficient 0.2
Direction Coefficient 0.1
Minimum Length 100% Cylinder Length
Minimum Length 50% Cylinder Length
Parameter

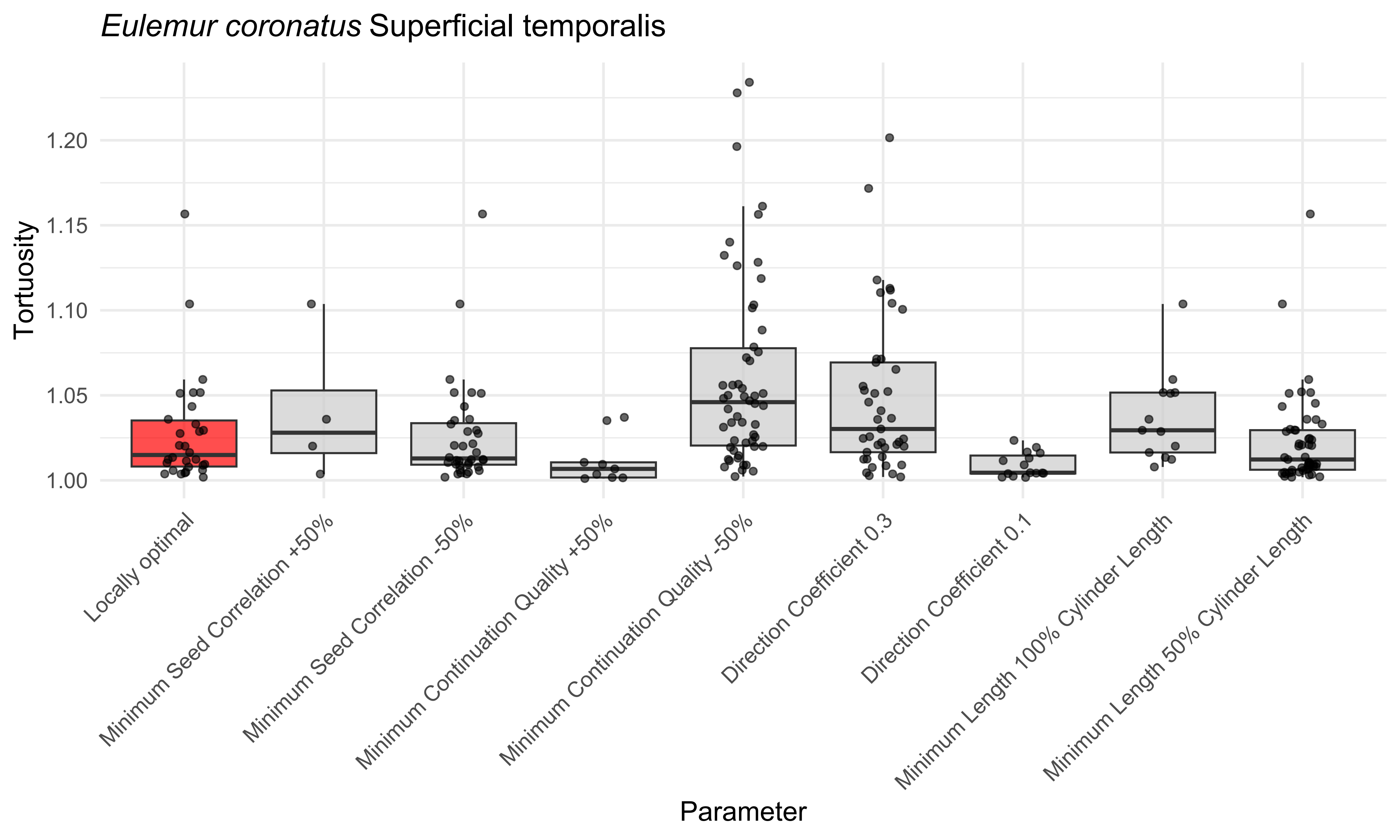

Eulemur coronatus Superficial temporalis
Tortuosity
1.00
1.05
1.10
1.15
1.20
Locally optimal
Minimum Seed Correlation +50%
Minimum Seed Correlation -50%
Minimum Continuation Quality +50%
Minimum Continuation Quality -50%
Direction Coefficient 0.3
Direction Coefficient 0.1
Minimum Length 100% Cylinder Length
Minimum Length 50% Cylinder Length
Parameter

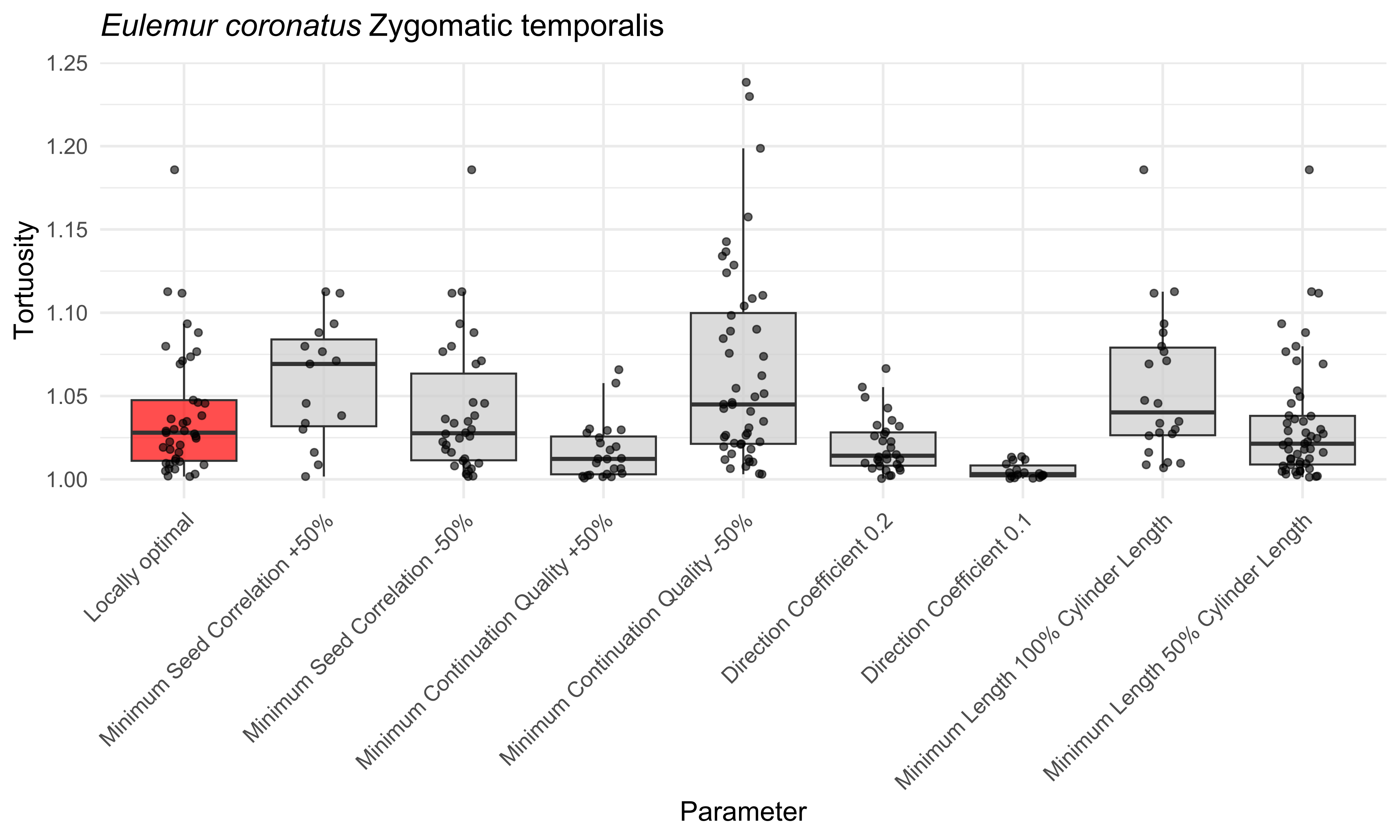

Eulemur coronatus Zygomatic temporalis
1.25
1.20
1.15
1.10
1.05
1.00
Tortuosity
Locally optimal
Minimum Seed Correlation +50%
Minimum Seed Correlation -50%
Minimum Continuation Quality +50%
Minimum Continuation Quality -50%
Direction Coefficient 0.2
Direction Coefficient 0.1
Minimum Length 100% Cylinder Length
Minimum Length 50% Cylinder Length
Parameter

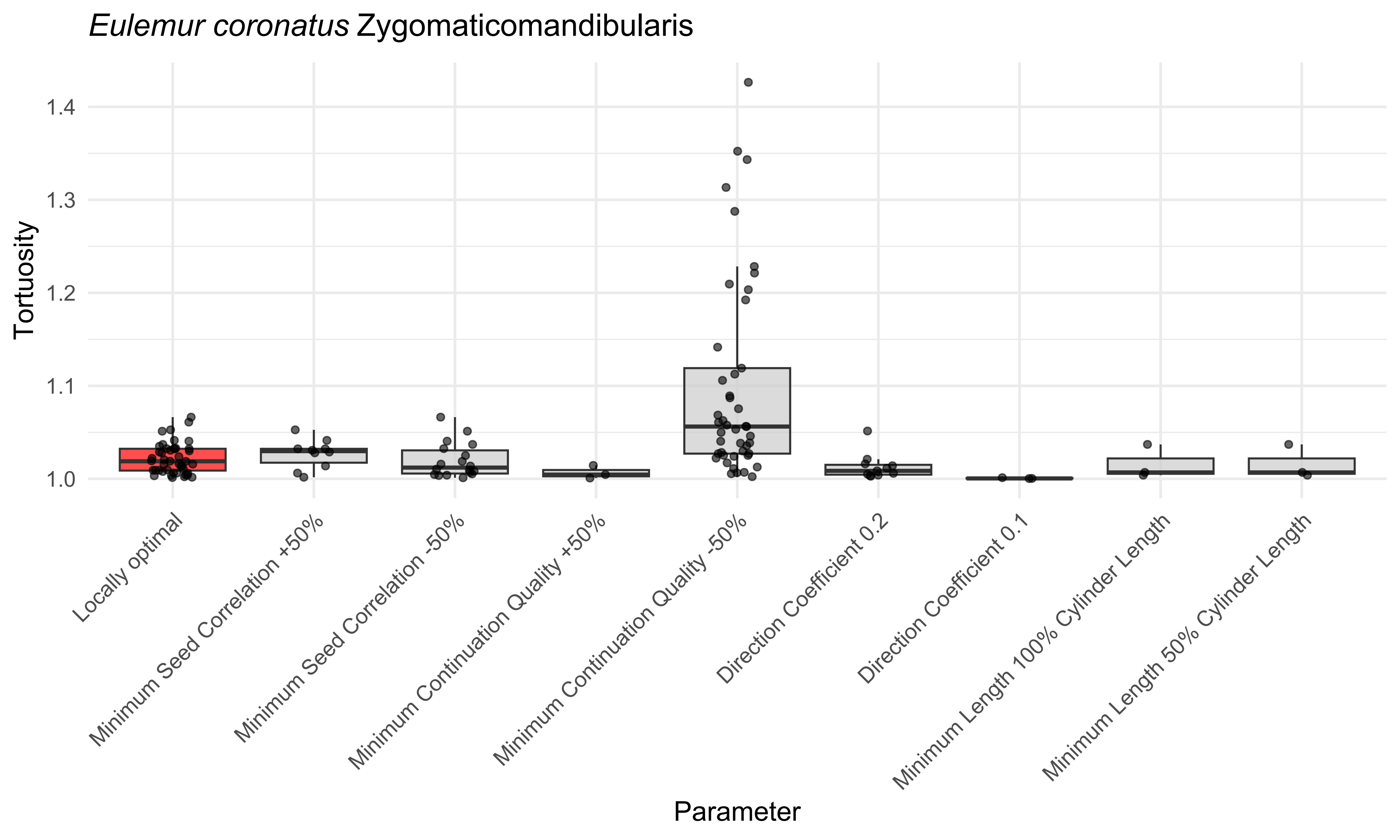

Eulemur coronatus Zygomaticomandibularis
Tortuosity
1.4
1.3
1.2
1.1
1.0
Locally optimal
Minimum Seed Correlation +50%
Minimum Seed Correlation -50%
Minimum Continuation Quality +50%
Minimum Continuation Quality -50%
Direction Coefficient 0.2
Direction Coefficient 0.1
Minimum Length 100% Cylinder Length
Minimum Length 50% Cylinder Length
Parameter

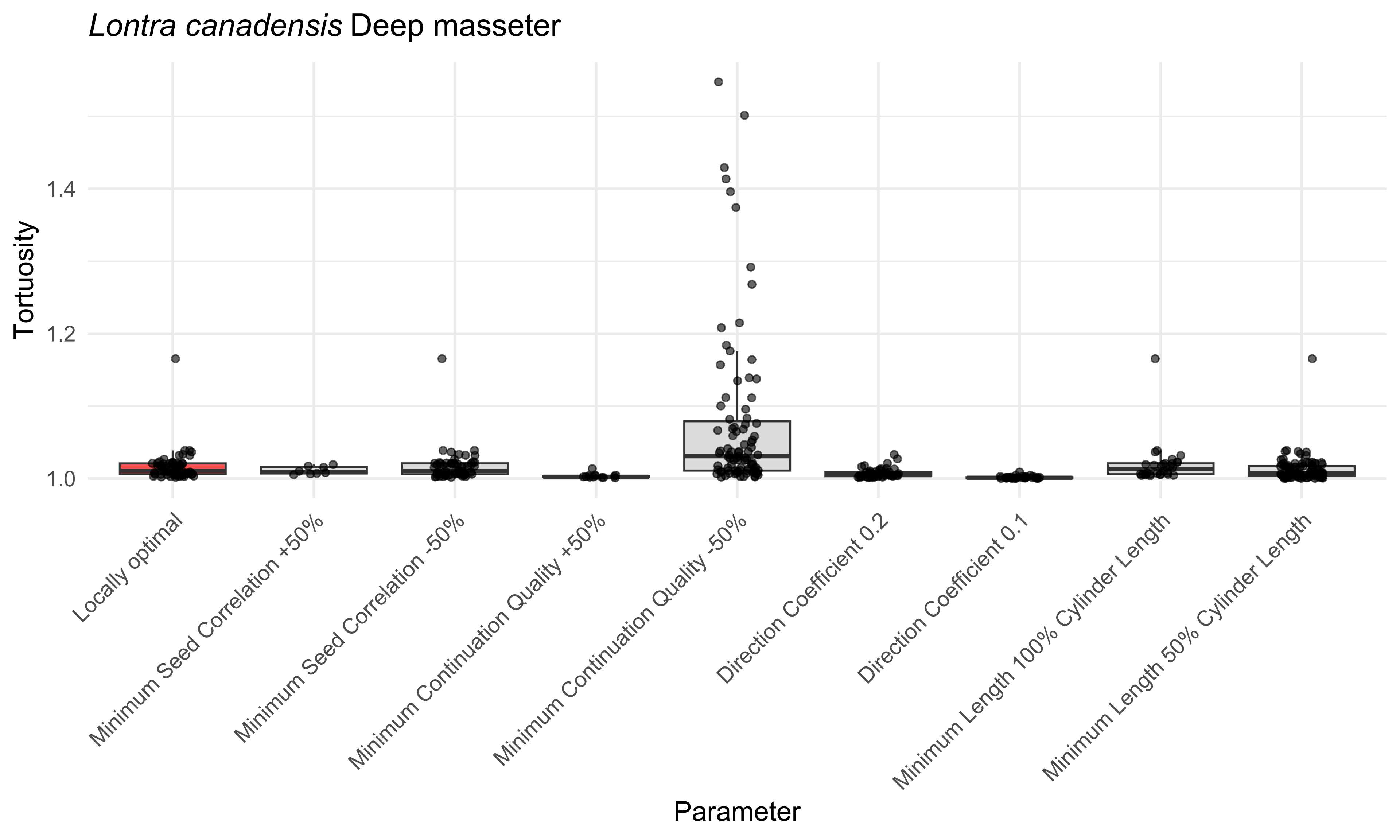

Lontra canadensis Deep masseter
Tortuosity
1.4
1.2
1.0
Locally optimal
Minimum Seed Correlation +50%
Minimum Seed Correlation -50%
Minimum Continuation Quality +50%
Minimum Continuation Quality -50%
Direction Coefficient 0.2
Direction Coefficient 0.1
Minimum Length 100% Cylinder Length
Minimum Length 50% Cylinder Length
Parameter

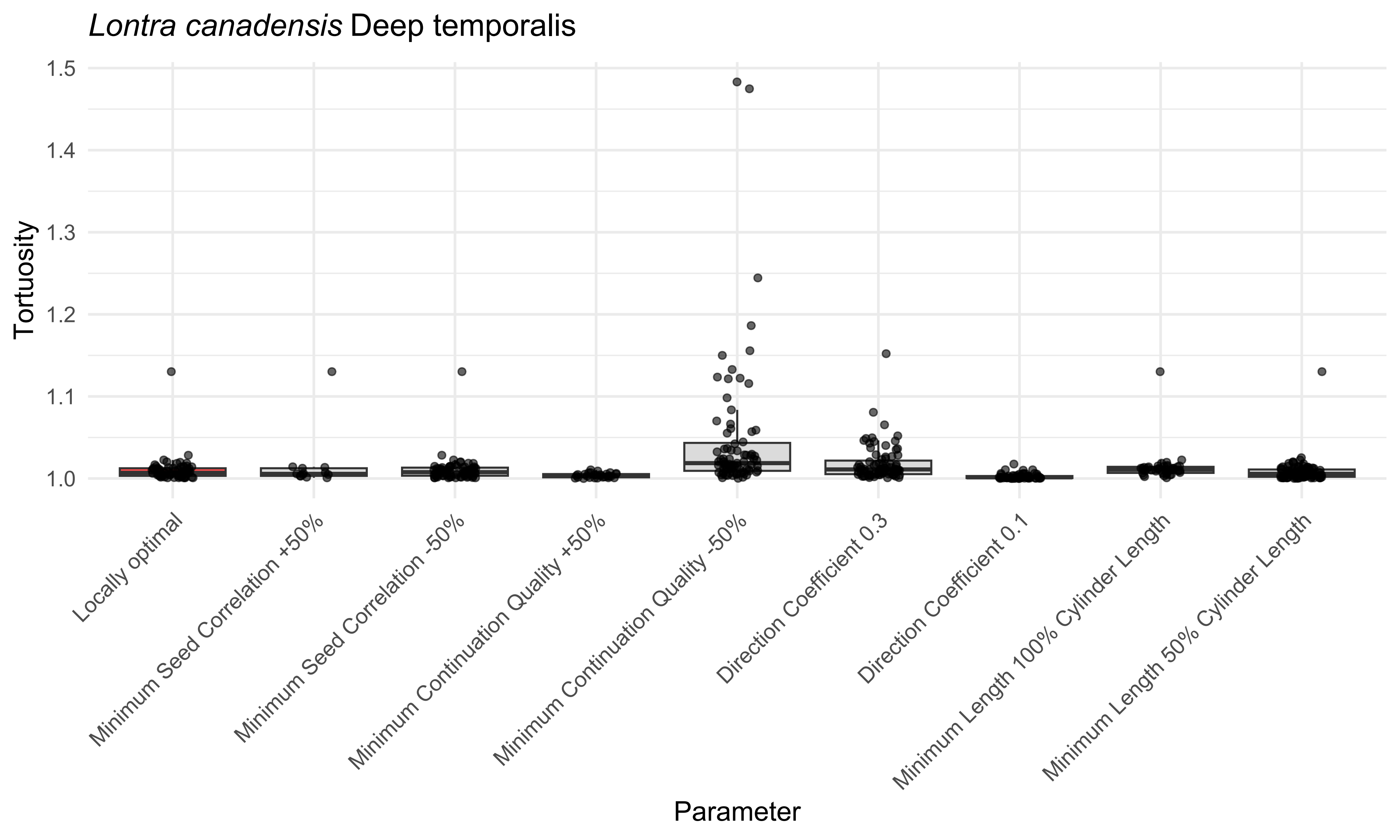

Lontra canadensis Deep temporalis
Tortuosity
1.5
1.4
1.3
1.2
1.1
1.0
Locally optimal
Minimum Seed Correlation +50%
Minimum Seed Correlation -50%
Minimum Continuation Quality +50%
Minimum Continuation Quality -50%
Direction Coefficient 0.3
Direction Coefficient 0.1
Minimum Length 100% Cylinder Length
Minimum Length 50% Cylinder Length
Parameter

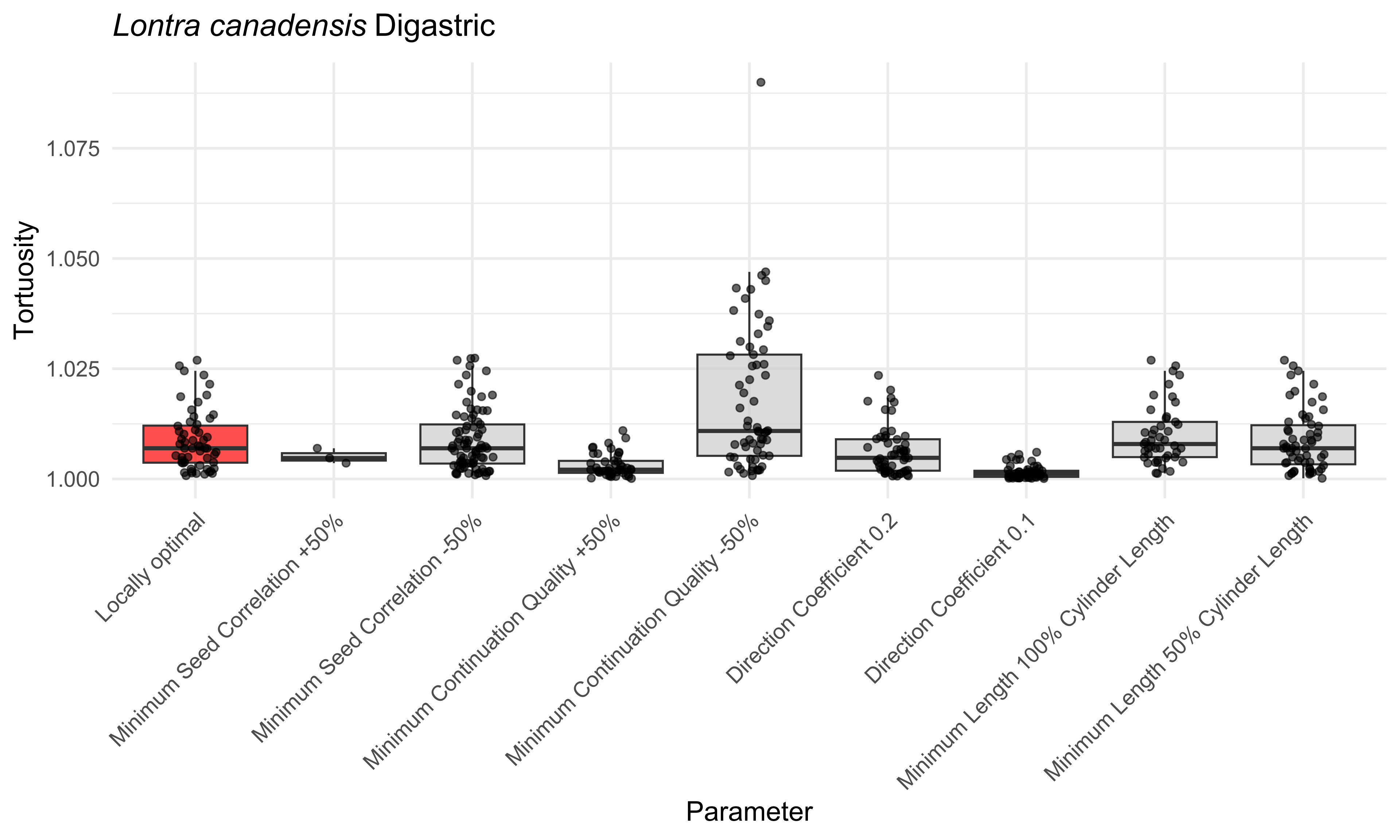

Lontra canadensis Digastric
Tortuosity
1.075
1.050
1.025
1.000
Locally optimal
Minimum Seed Correlation +50%
Minimum Seed Correlation -50%
Minimum Continuation Quality +50%
Minimum Continuation Quality -50%
Direction Coefficient 0.2
Direction Coefficient 0.1
Minimum Length 100% Cylinder Length
Minimum Length 50% Cylinder Length
Parameter

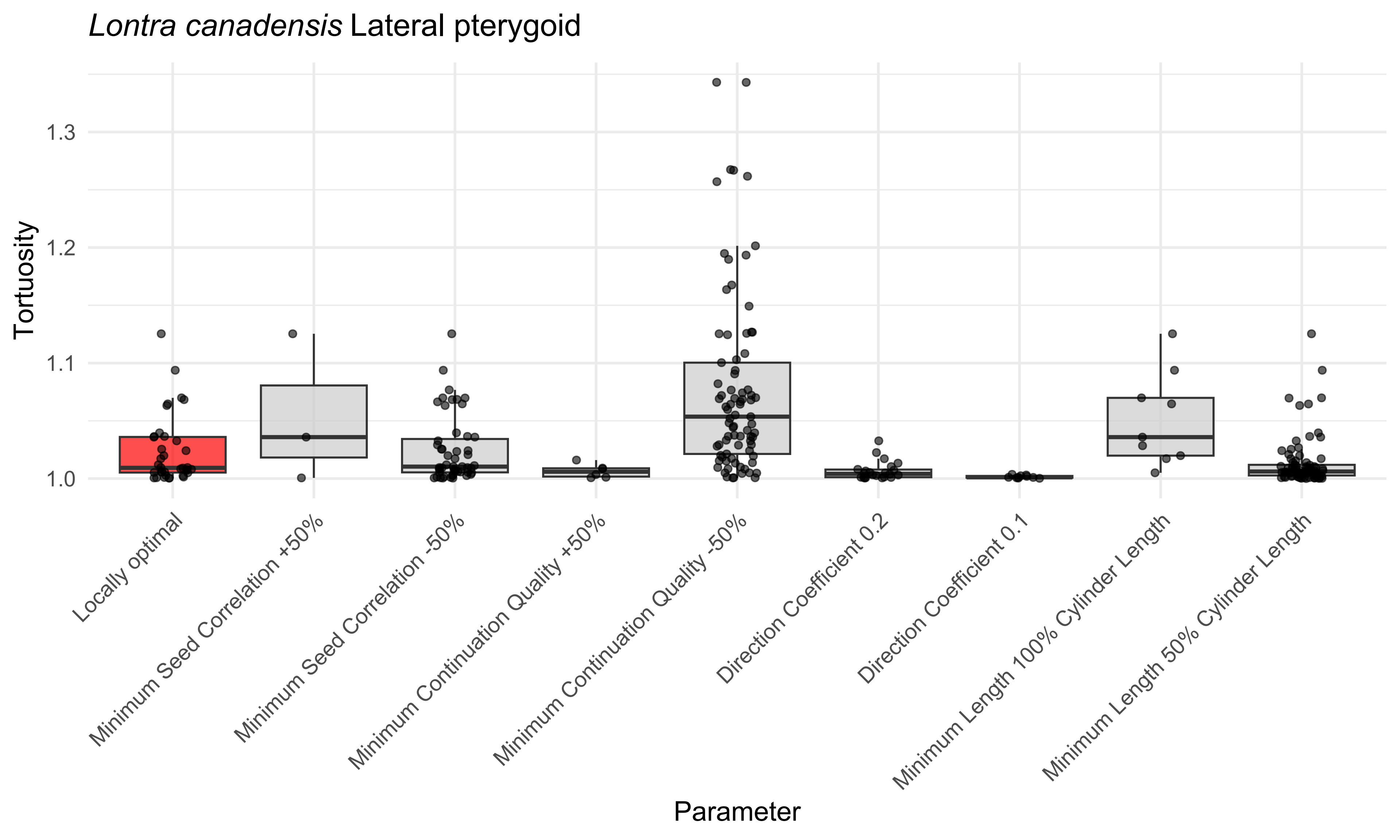

Lontra canadensis Lateral pterygoid
Tortuosity
1.3
1.2
1.1
1.0
Locally optimal
Minimum Seed Correlation +50%
Minimum Seed Correlation -50%
Minimum Continuation Quality +50%
Minimum Continuation Quality -50%
Direction Coefficient 0.2
Direction Coefficient 0.1
Minimum Length 100% Cylinder Length
Minimum Length 50% Cylinder Length
Parameter

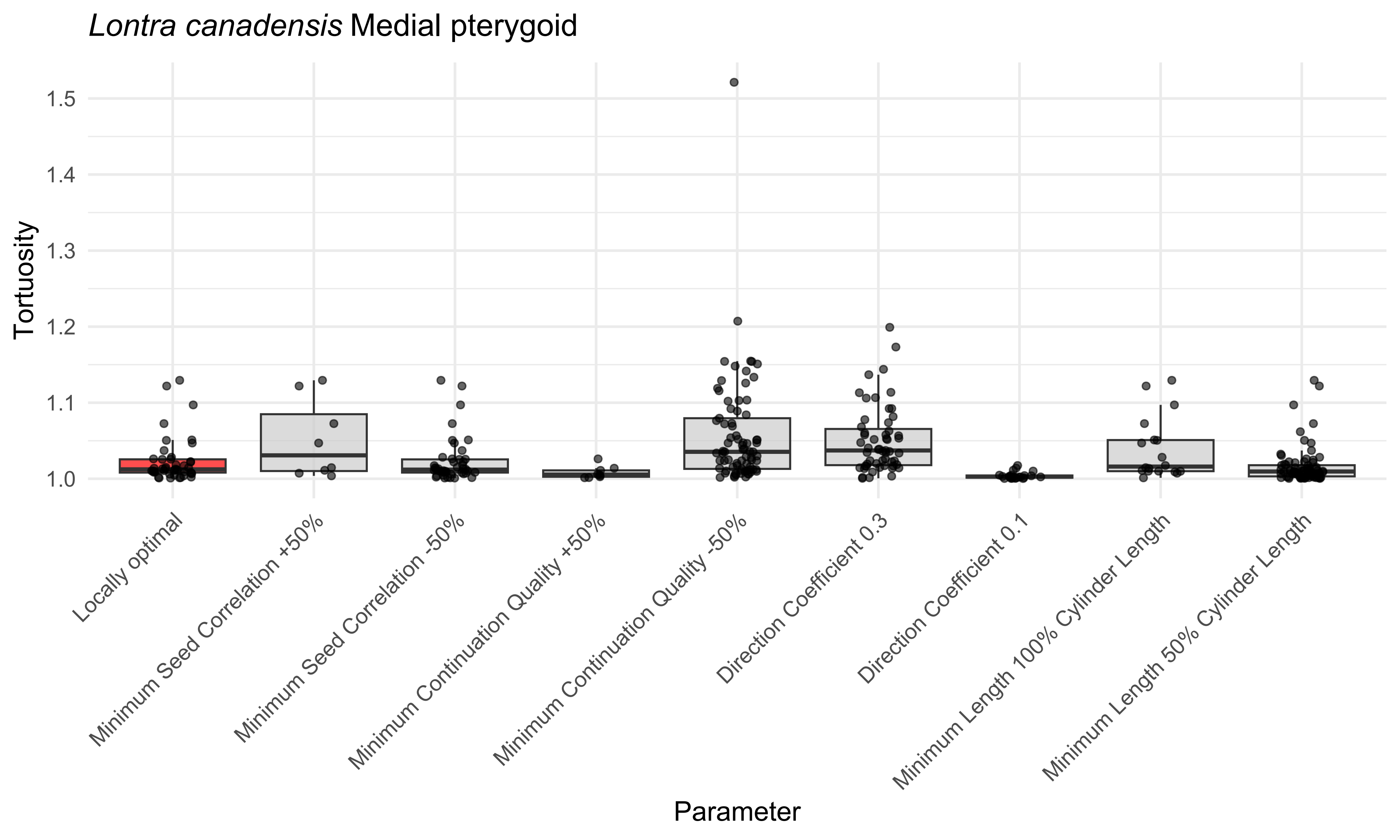

Lontra canadensis Medial pterygoid
Tortuosity
1.5
1.4
1.3
1.2
1.1
1.0
Locally optimal
Minimum Seed Correlation +50%
Minimum Seed Correlation -50%
Minimum Continuation Quality +50%
Minimum Continuation Quality -50%
Direction Coefficient 0.3
Direction Coefficient 0.1
Minimum Length 100% Cylinder Length
Minimum Length 50% Cylinder Length
Parameter

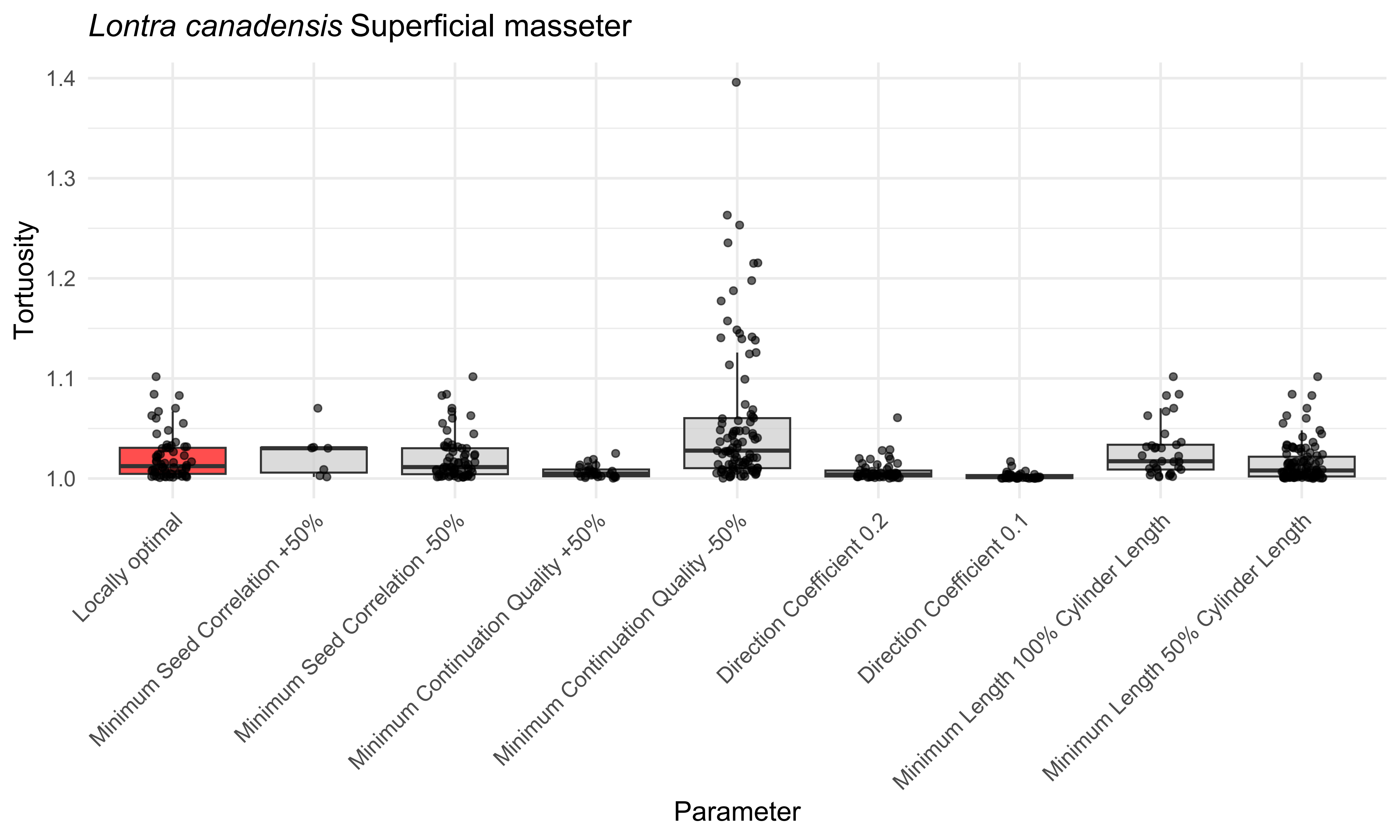

Lontra canadensis Superficial masseter
Tortuosity
1.4
1.3
1.2
1.1
1.0
Locally optimal
Minimum Seed Correlation +50%
Minimum Seed Correlation -50%
Minimum Continuation Quality +50%
Minimum Continuation Quality -50%
Direction Coefficient 0.2
Direction Coefficient 0.1
Minimum Length 100% Cylinder Length
Minimum Length 50% Cylinder Length
Parameter

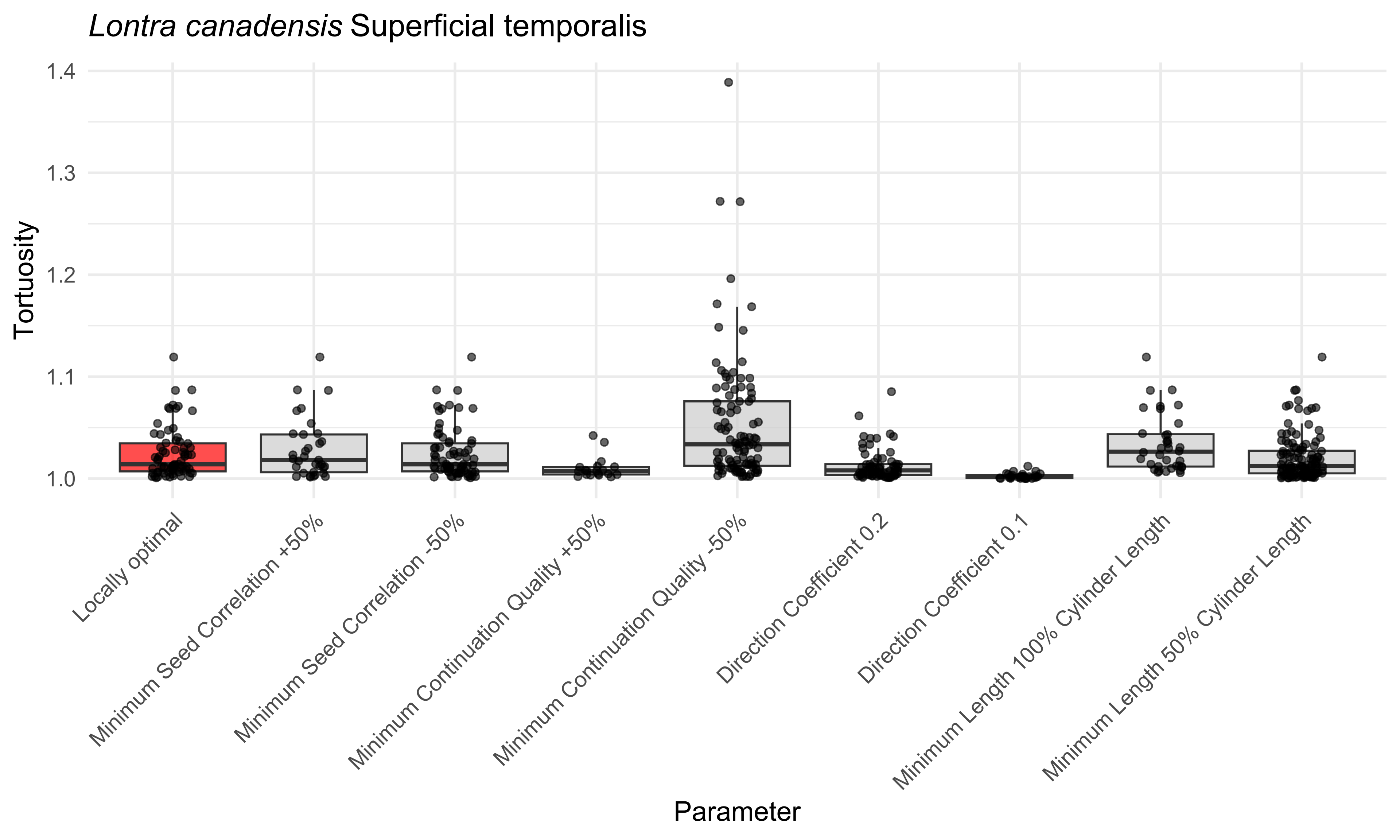

Lontra canadensis Superficial temporalis
Tortuosity
1.4
1.3
1.2
1.1
1.0
Locally optimal
Minimum Seed Correlation +50%
Minimum Seed Correlation -50%
Minimum Continuation Quality +50%
Minimum Continuation Quality -50%
Direction Coefficient 0.2
Direction Coefficient 0.1
Minimum Length 100% Cylinder Length
Minimum Length 50% Cylinder Length
Parameter

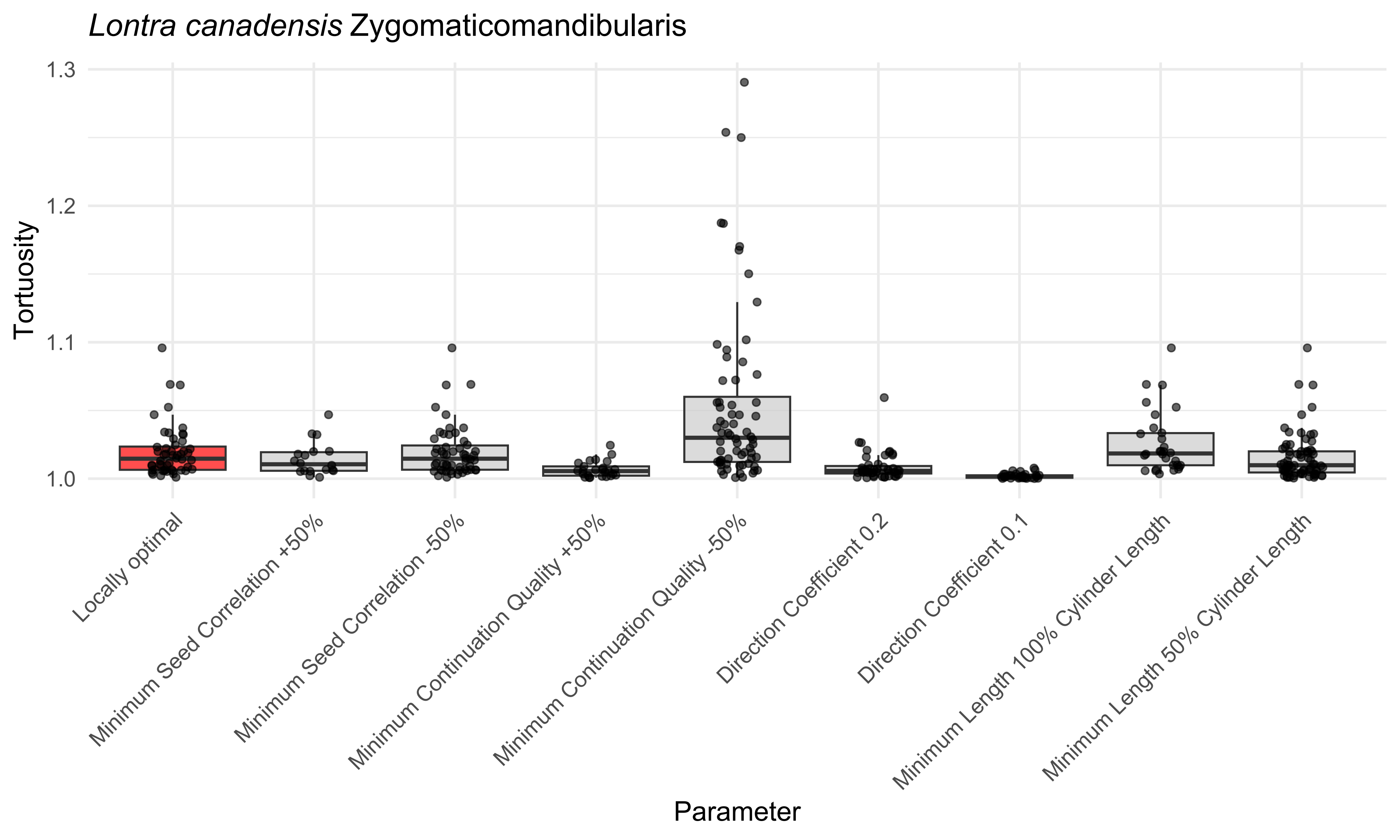

Lontra canadensis Zygomaticomandibularis
Tortuosity
1.0
1.1
1.2
1.3
Locally optimal
Minimum Seed Correlation +50%
Minimum Seed Correlation -50%
Minimum Continuation Quality +50%
Minimum Continuation Quality -50%
Direction Coefficient 0.2
Direction Coefficient 0.1
Minimum Length 100% Cylinder Length
Minimum Length 50% Cylinder Length
Parameter

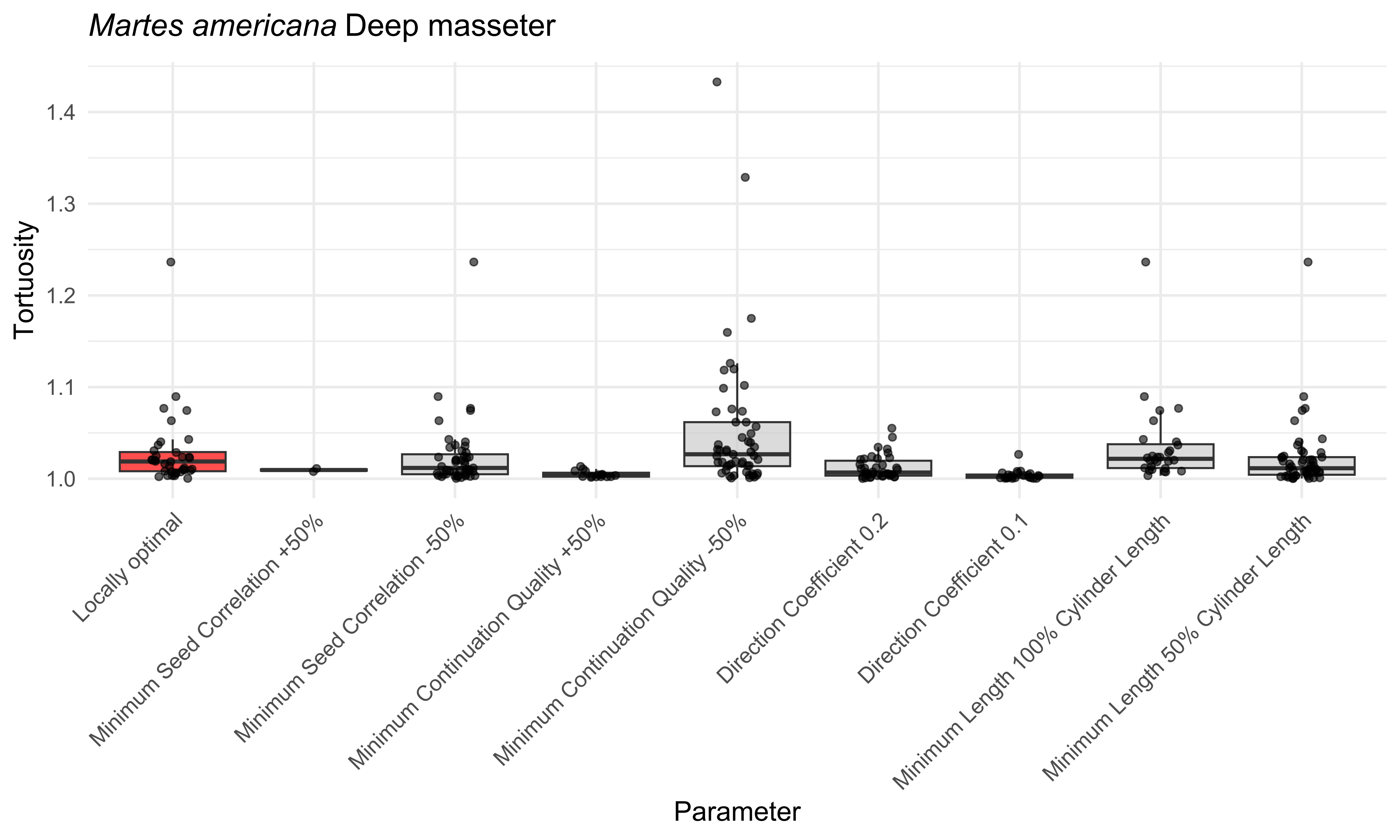

Martes americana Deep masseter
Tortuosity
1.0
1.1
1.2
1.3
1.4
Parameter
Locally optimal
Minimum Seed Correlation +50%
Minimum Seed Correlation -50%
Minimum Continuation Quality +50%
Minimum Continuation Quality -50%
Direction Coefficient 0.2
Direction Coefficient 0.1
Minimum Length 100% Cylinder Length
Minimum Length 50% Cylinder Length

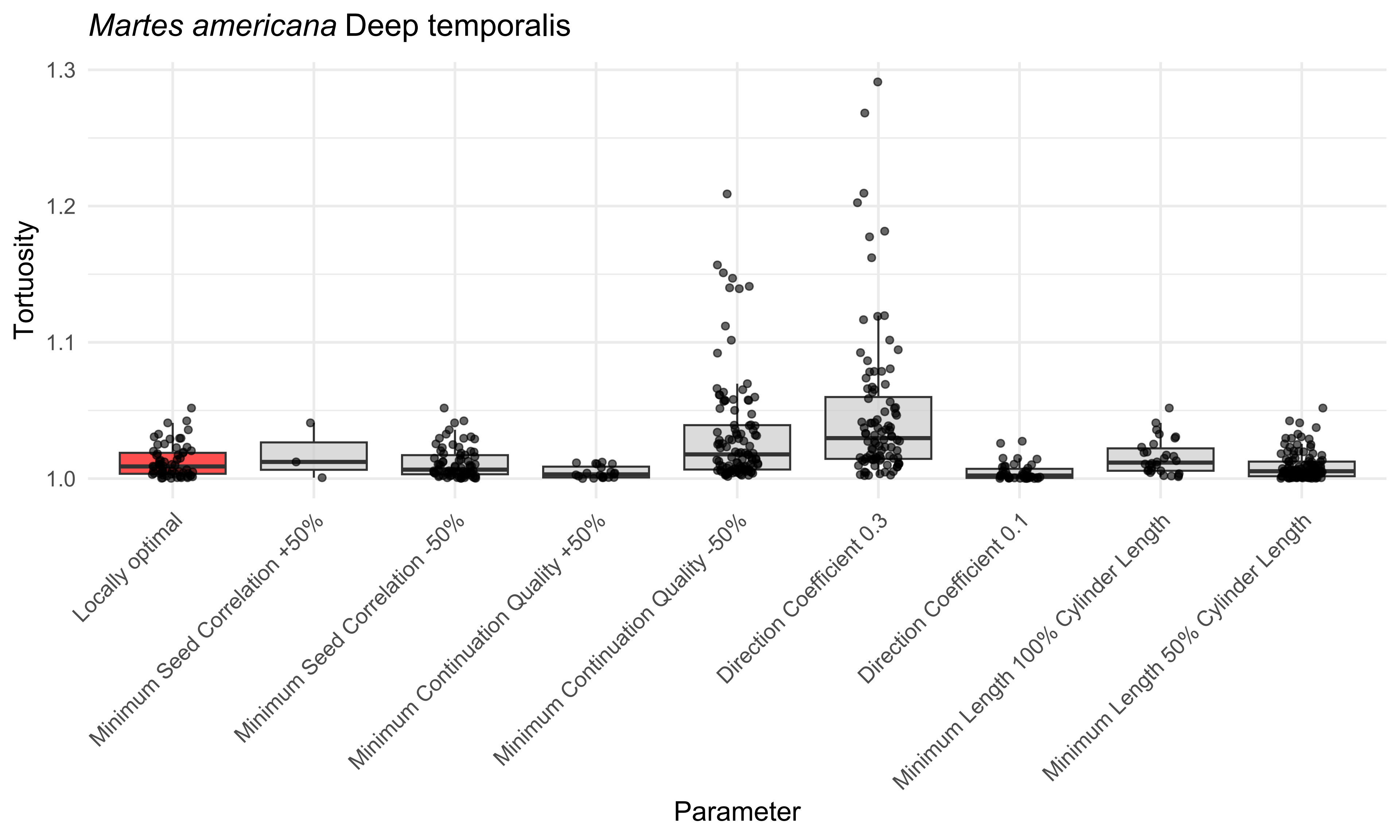

Martes americana Deep temporalis
1.3
1.2
1.1
1.0
Tortuosity
Locally optimal
Minimum Seed Correlation +50%
Minimum Seed Correlation -50%
Minimum Continuation Quality +50%
Minimum Continuation Quality -50%
Direction Coefficient 0.3
Direction Coefficient 0.1
Minimum Length 100% Cylinder Length
Minimum Length 50% Cylinder Length
Parameter

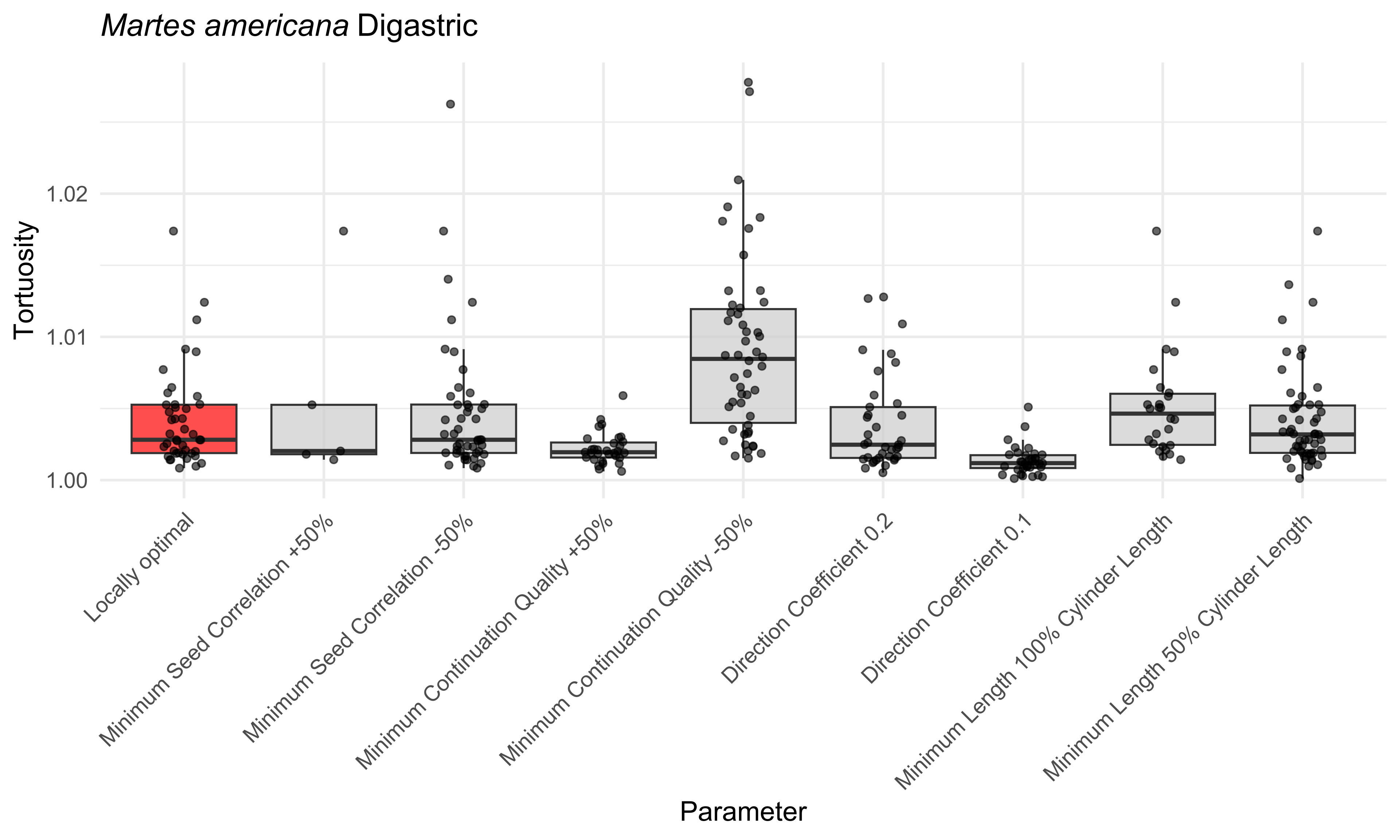

Martes americana Digastric
Tortuosity
1.00
1.01
1.02
Parameter
Locally optimal
Minimum Seed Correlation +50%
Minimum Seed Correlation -50%
Minimum Continuation Quality +50%
Minimum Continuation Quality -50%
Direction Coefficient 0.2
Direction Coefficient 0.1
Minimum Length 100% Cylinder Length
Minimum Length 50% Cylinder Length

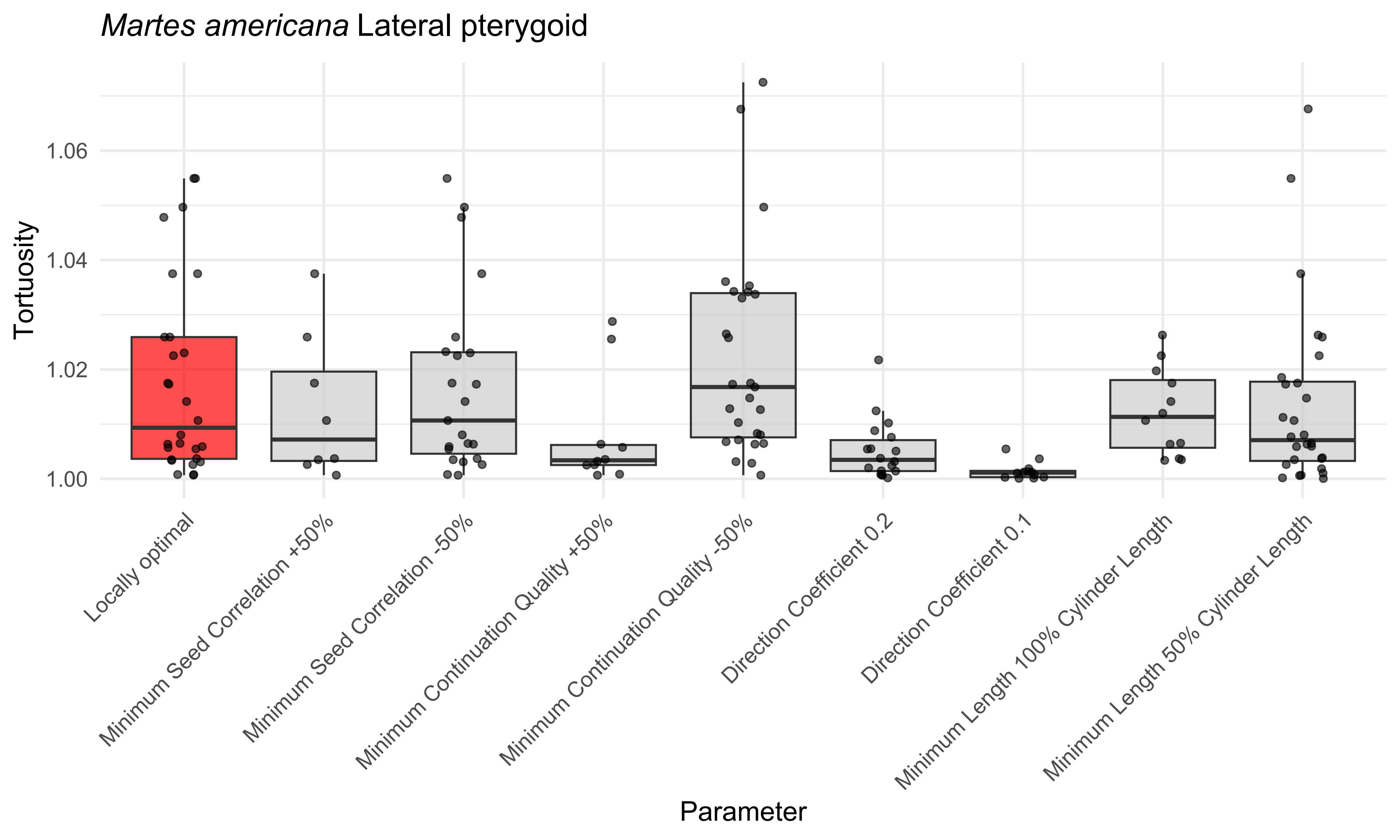
Martes americana Lateral pterygoid
Tortuosity
1.06
1.04
1.02
1.00
Locally optimal
Minimum Seed Correlation +50%
Minimum Seed Correlation -50%
Minimum Continuation Quality +50%
Minimum Continuation Quality -50%
Direction Coefficient 0.2
Direction Coefficient 0.1
Minimum Length 100% Cylinder Length
Minimum Length 50% Cylinder Length
Parameter

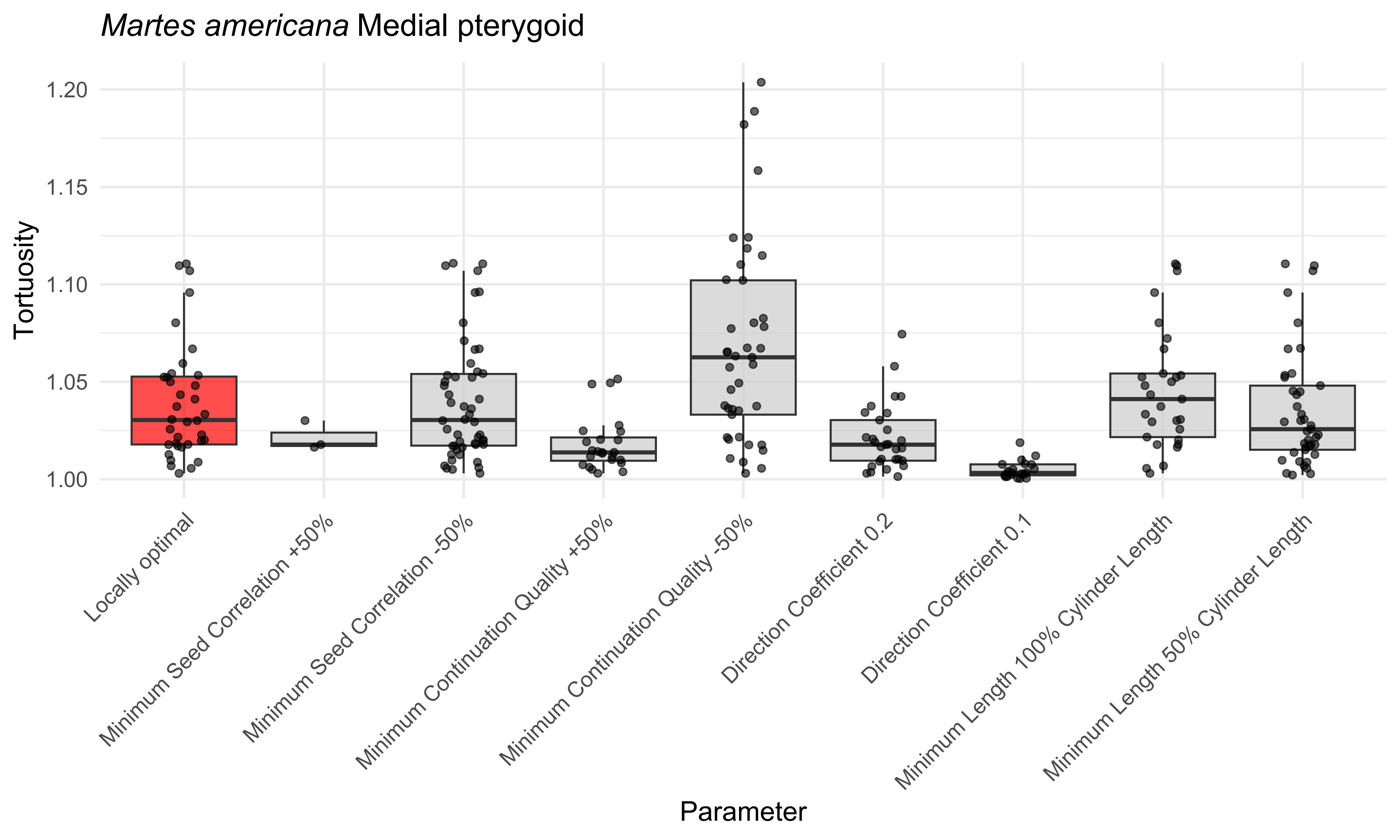
Martes americana Medial pterygoid
Tortuosity
1.20
1.15
1.10
1.05
1.00
Locally optimal
Minimum Seed Correlation +50%
Minimum Seed Correlation -50%
Minimum Continuation Quality +50%
Minimum Continuation Quality -50%
Direction Coefficient 0.2
Direction Coefficient 0.1
Minimum Length 100% Cylinder Length
Minimum Length 50% Cylinder Length
Parameter

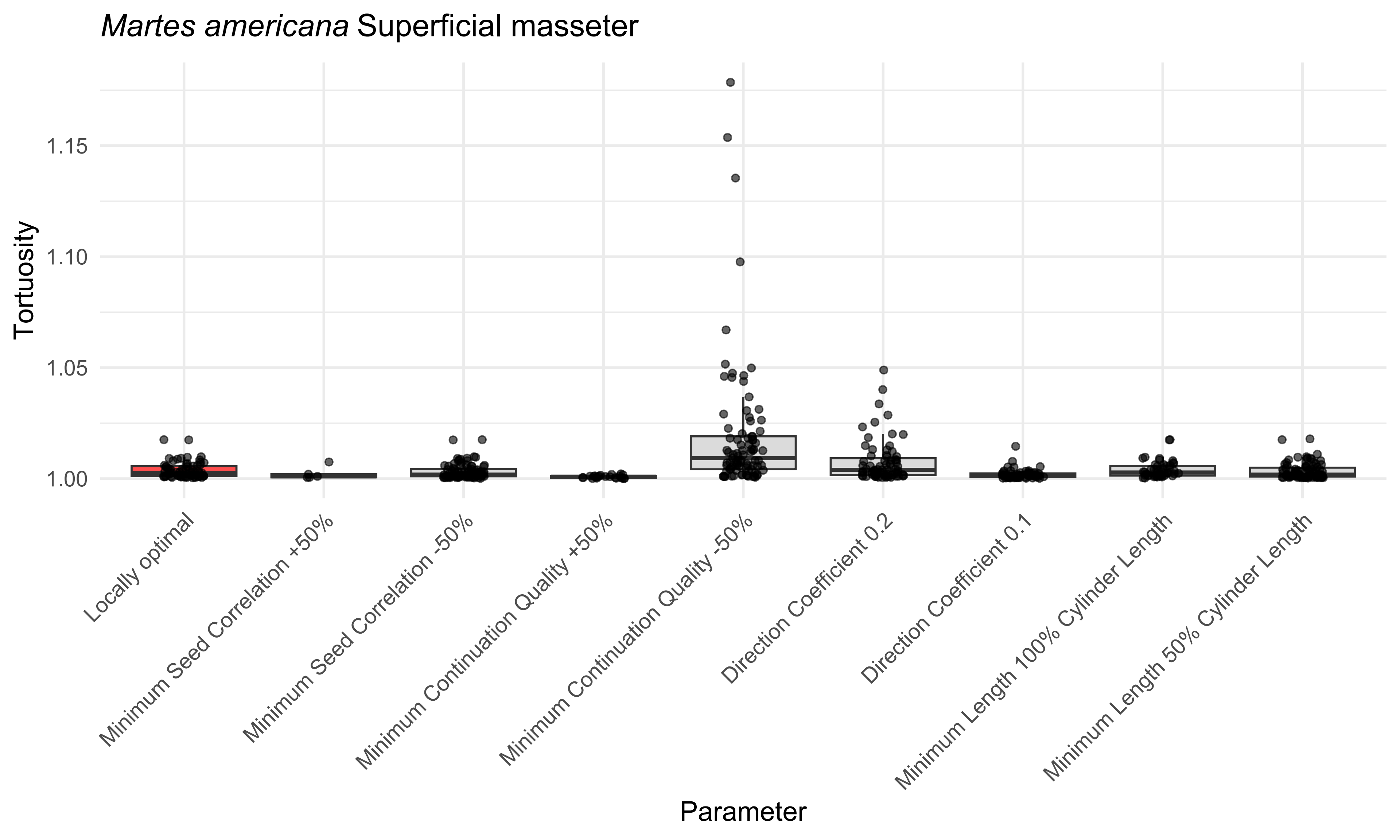
Martes americana Superficial masseter
Tortuosity
1.15
1.10
1.05
1.00
Locally optimal
Minimum Seed Correlation +50%
Minimum Seed Correlation -50%
Minimum Continuation Quality +50%
Minimum Continuation Quality -50%
Direction Coefficient 0.2
Direction Coefficient 0.1
Minimum Length 100% Cylinder Length
Minimum Length 50% Cylinder Length
Parameter

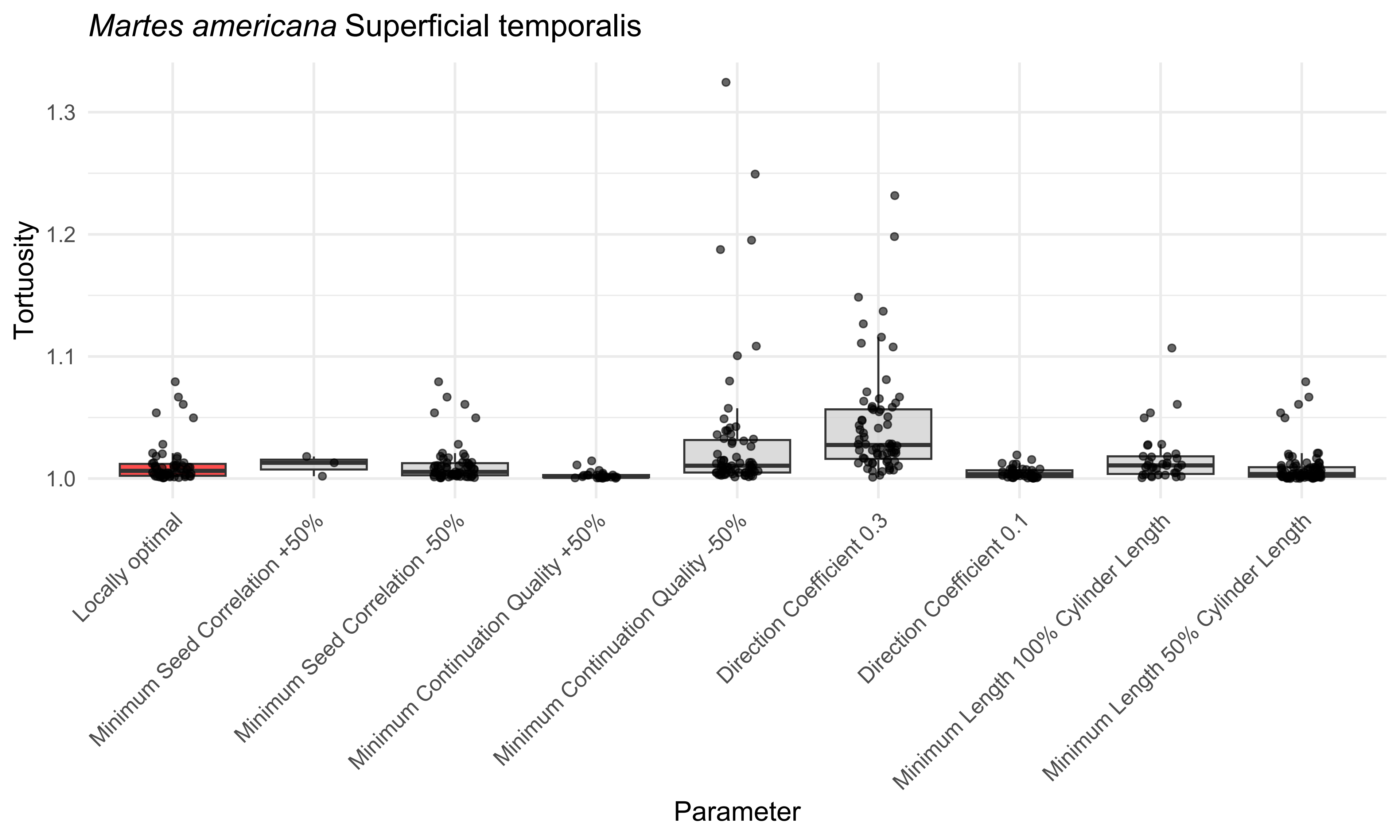

Martes americana Superficial temporalis
Tortuosity
1.3
1.2
1.1
1.0
Locally optimal
Minimum Seed Correlation +50%
Minimum Seed Correlation -50%
Minimum Continuation Quality +50%
Minimum Continuation Quality -50%
Direction Coefficient 0.3
Direction Coefficient 0.1
Minimum Length 100% Cylinder Length
Minimum Length 50% Cylinder Length
Parameter

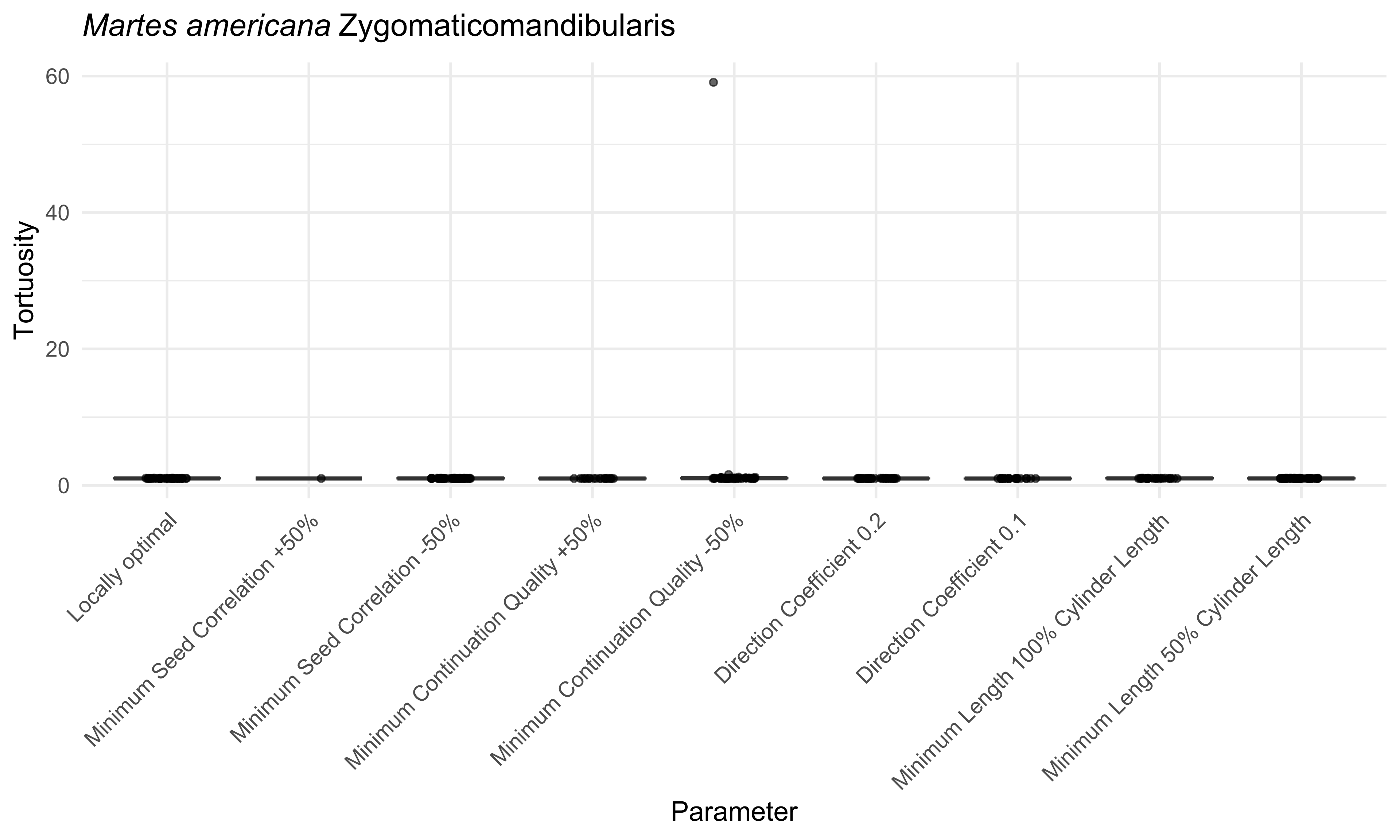

Martes americana Zygomaticomandibularis
Tortuosity
60
40
20
0
Locally optimal
Minimum Seed Correlation +50%
Minimum Seed Correlation -50%
Minimum Continuation Quality +50%
Minimum Continuation Quality -50%
Direction Coefficient 0.2
Direction Coefficient 0.1
Minimum Length 100% Cylinder Length
Minimum Length 50% Cylinder Length
Parameter

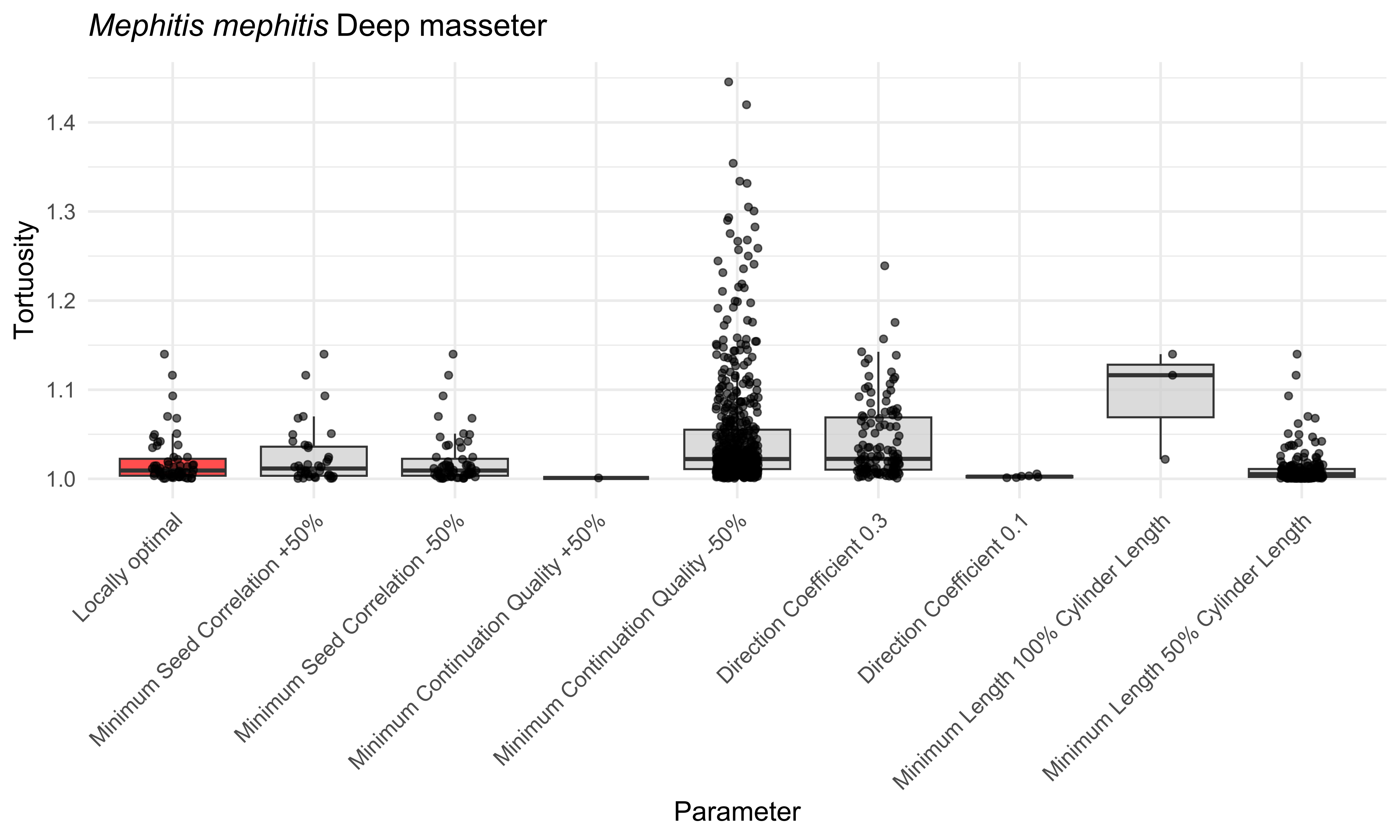

Mephitis mephitis Deep masseter
Tortuosity
1.0
1.1
1.2
1.3
1.4
Locally optimal
Minimum Seed Correlation +50%
Minimum Seed Correlation -50%
Minimum Continuation Quality +50%
Minimum Continuation Quality -50%
Direction Coefficient 0.3
Direction Coefficient 0.1
Minimum Length 100% Cylinder Length
Minimum Length 50% Cylinder Length
Parameter

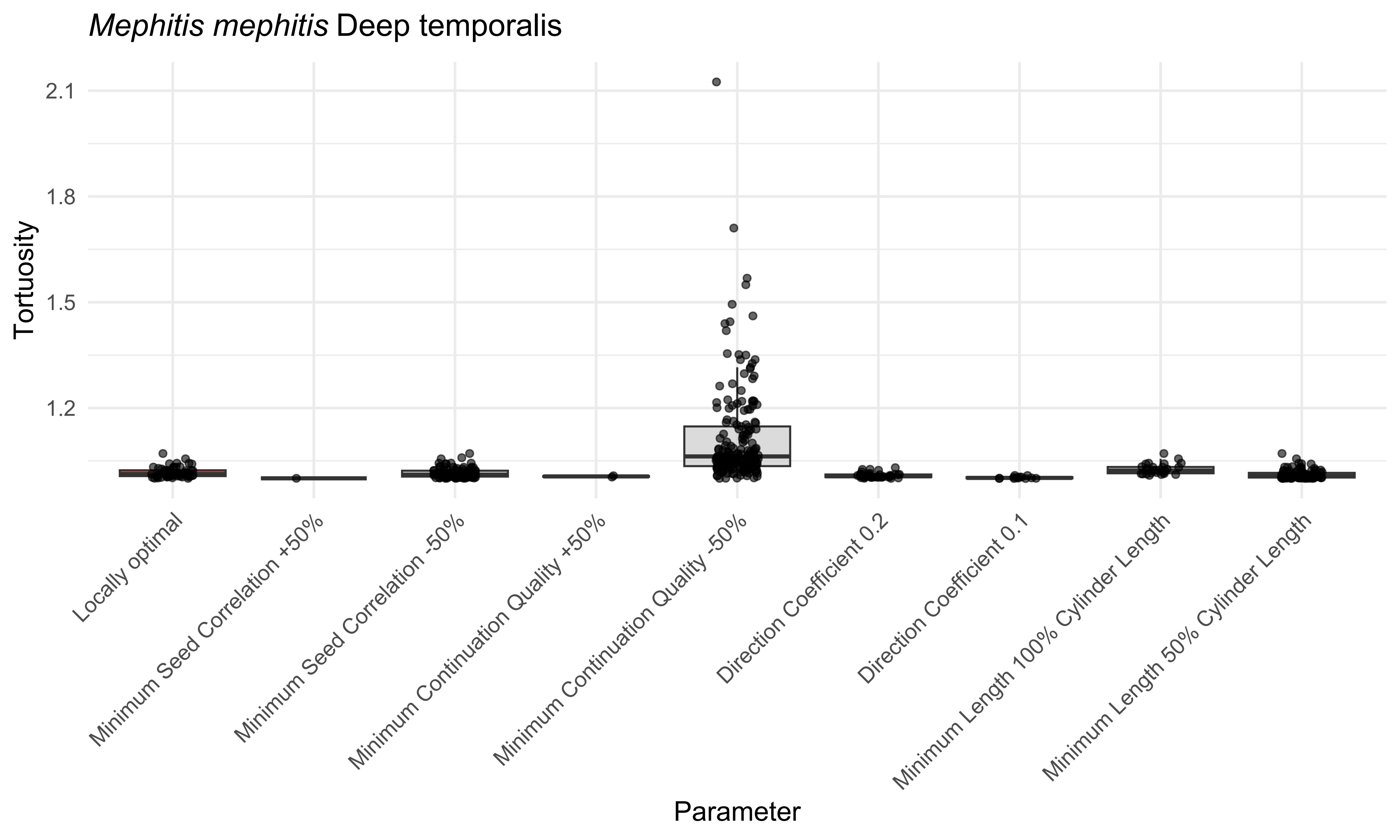

Mephitis mephitis Deep temporalis
Tortuosity
2.1
1.8
1.5
1.2
Locally optimal
Minimum Seed Correlation +50%
Minimum Seed Correlation -50%
Minimum Continuation Quality +50%
Minimum Continuation Quality -50%
Direction Coefficient 0.2
Direction Coefficient 0.1
Minimum Length 100% Cylinder Length
Minimum Length 50% Cylinder Length
Parameter

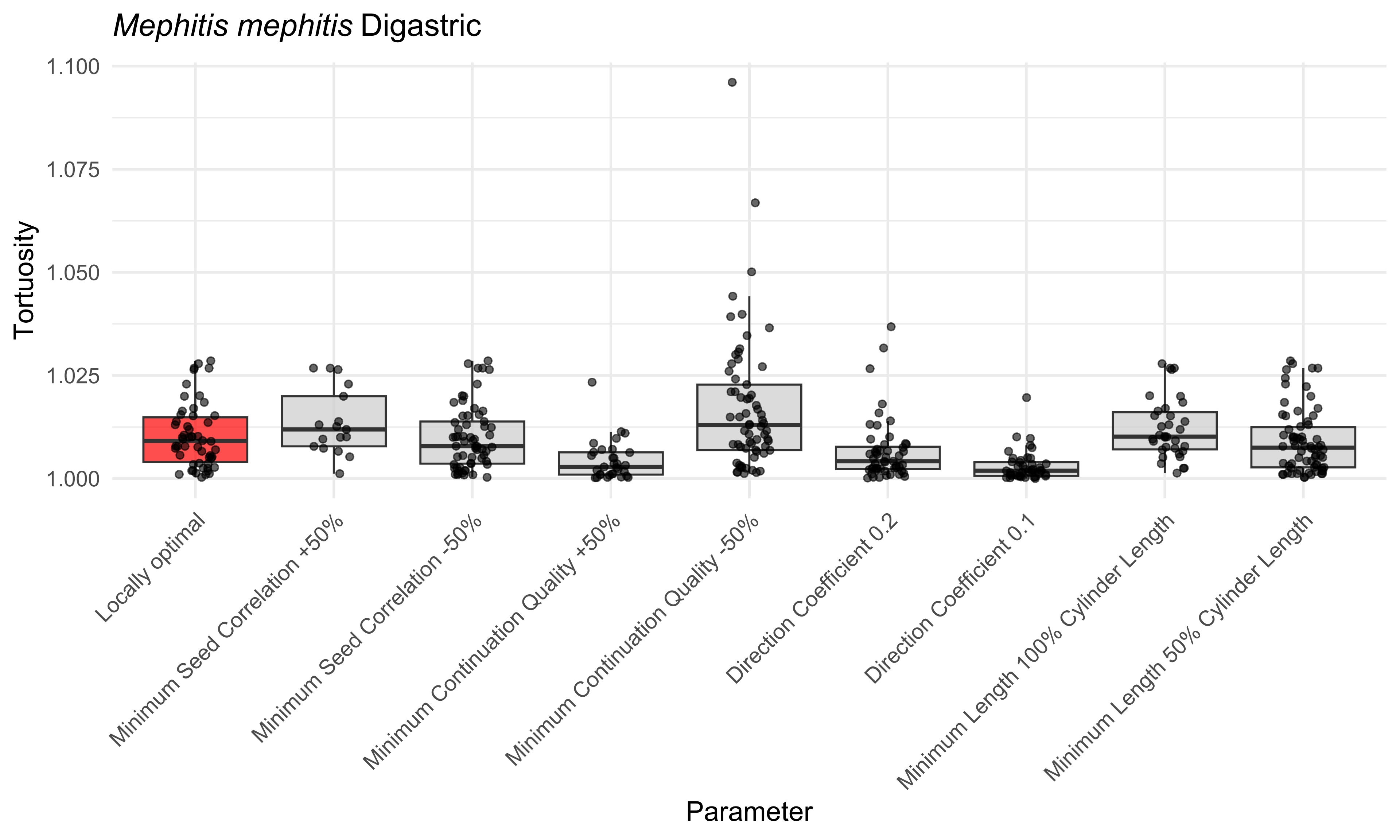

Mephitis mephitis Digastric
Tortuosity
1.100
1.075
1.050
1.025
1.000
Locally optimal
Minimum Seed Correlation +50%
Minimum Seed Correlation -50%
Minimum Continuation Quality +50%
Minimum Continuation Quality -50%
Direction Coefficient 0.2
Direction Coefficient 0.1
Minimum Length 100% Cylinder Length
Minimum Length 50% Cylinder Length
Parameter

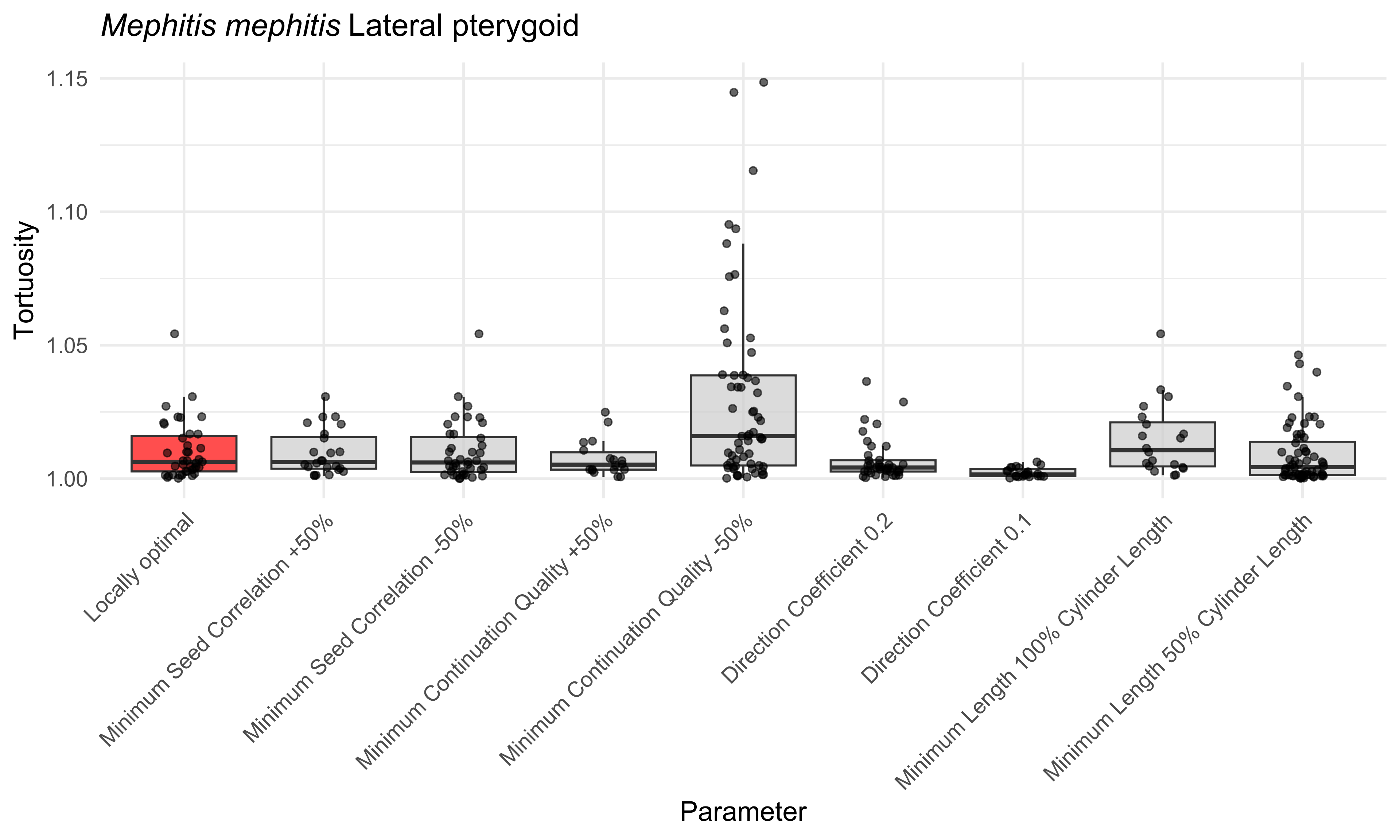

Mephitis mephitis Lateral pterygoid
Tortuosity
1.15
1.10
1.05
1.00
Locally optimal
Minimum Seed Correlation +50%
Minimum Seed Correlation -50%
Minimum Continuation Quality +50%
Minimum Continuation Quality -50%
Direction Coefficient 0.2
Direction Coefficient 0.1
Minimum Length 100% Cylinder Length
Minimum Length 50% Cylinder Length
Parameter

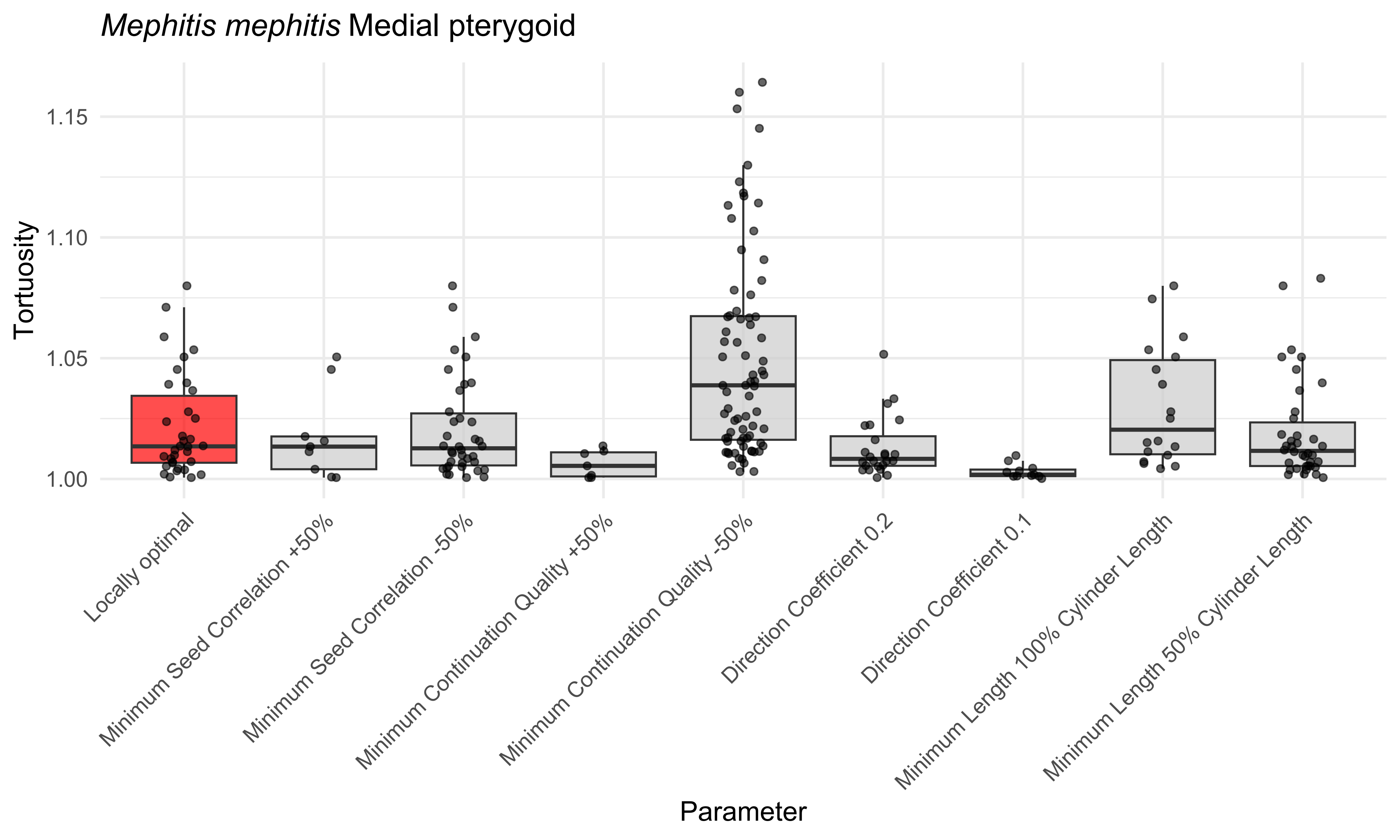

Mephitis mephitis Medial pterygoid
Tortuosity
1.15
1.10
1.05
1.00
Locally optimal
Minimum Seed Correlation +50%
Minimum Seed Correlation -50%
Minimum Continuation Quality +50%
Minimum Continuation Quality -50%
Direction Coefficient 0.2
Direction Coefficient 0.1
Minimum Length 100% Cylinder Length
Minimum Length 50% Cylinder Length
Parameter

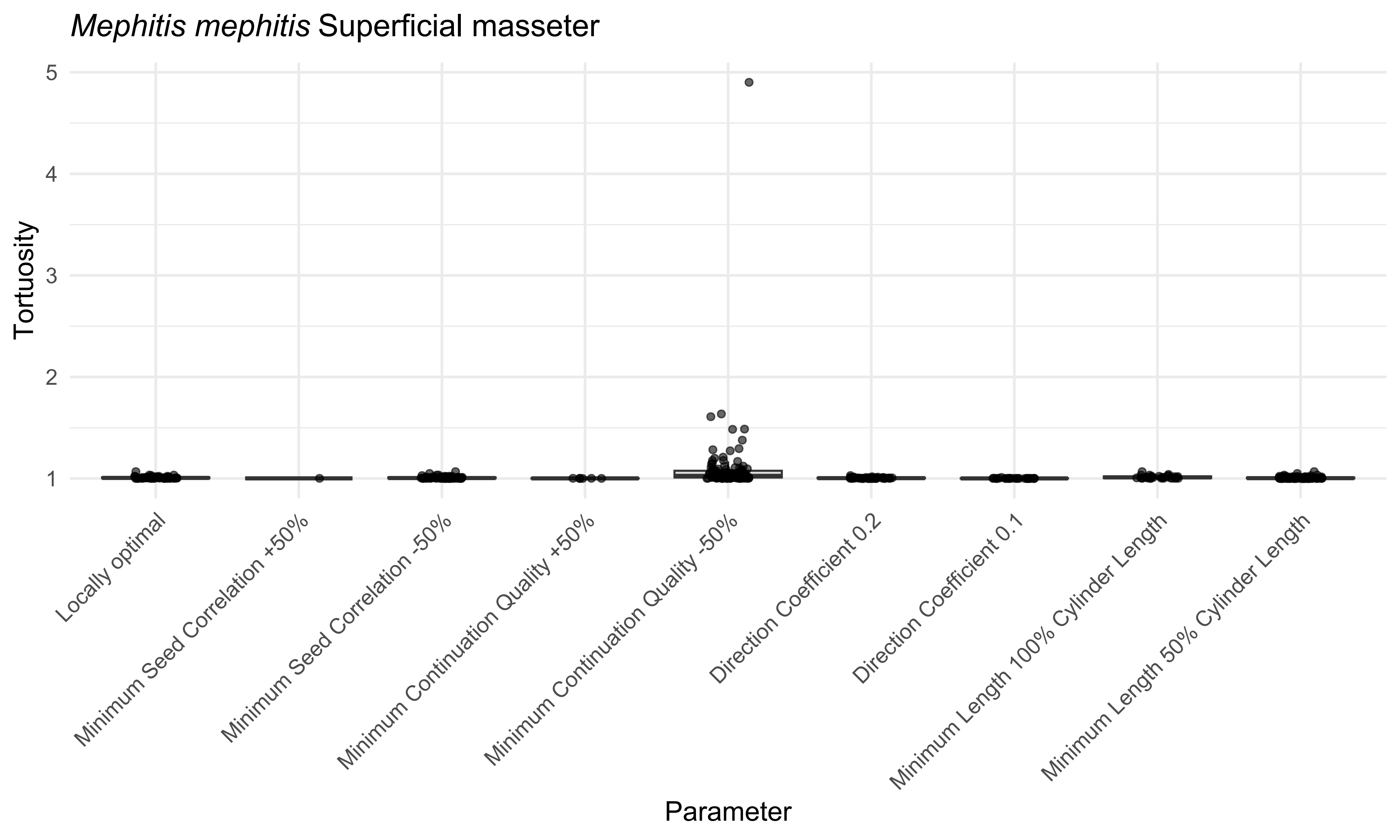

Mephitis mephitis Superficial masseter
Tortuosity
5
4
3
2
1
Locally optimal
Minimum Seed Correlation +50%
Minimum Seed Correlation -50%
Minimum Continuation Quality +50%
Minimum Continuation Quality -50%
Direction Coefficient 0.2
Direction Coefficient 0.1
Minimum Length 100% Cylinder Length
Minimum Length 50% Cylinder Length
Parameter

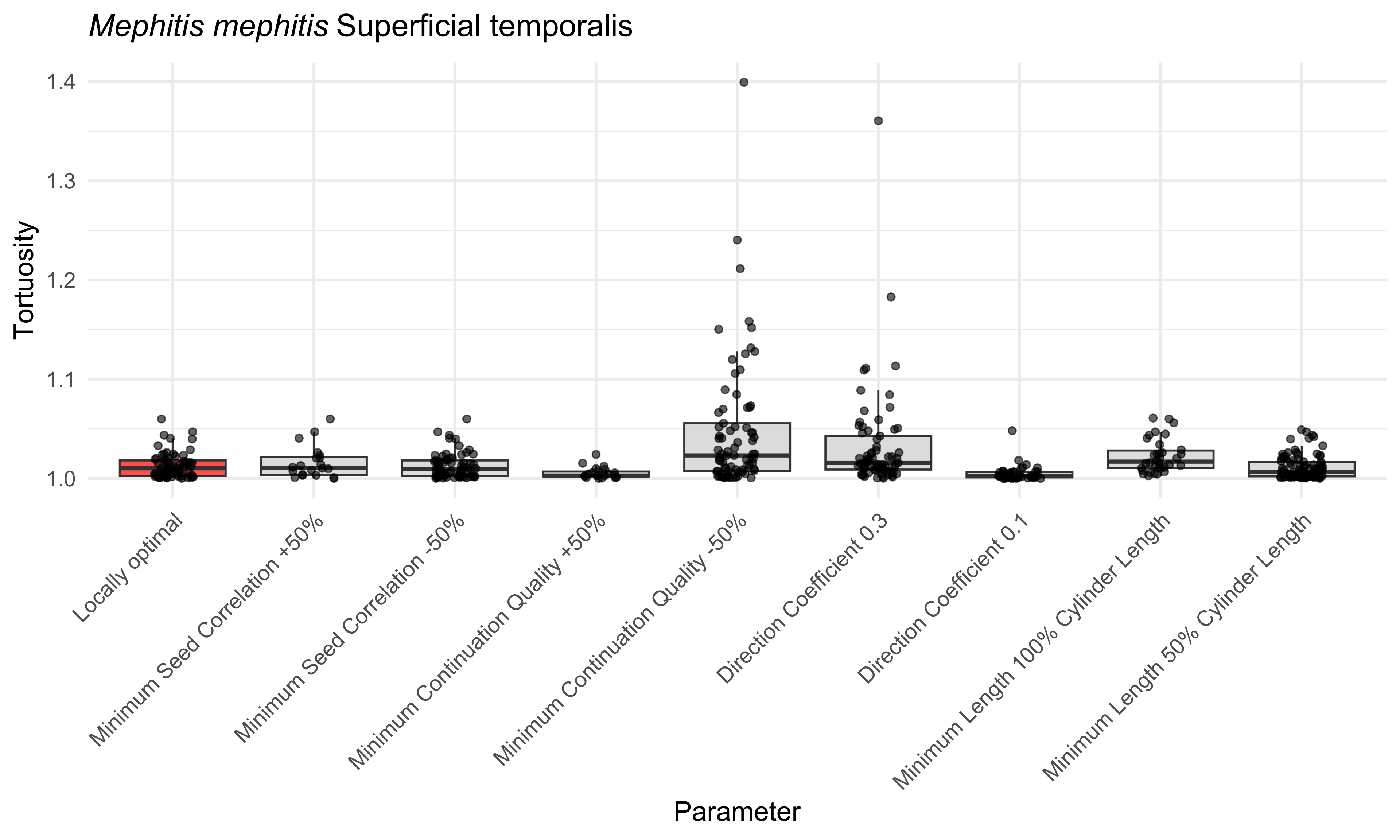

Mephitis mephitis Superficial temporalis
Tortuosity
1.4
1.3
1.2
1.1
1.0
Locally optimal
Minimum Seed Correlation +50%
Minimum Seed Correlation -50%
Minimum Continuation Quality +50%
Minimum Continuation Quality -50%
Direction Coefficient 0.3
Direction Coefficient 0.1
Minimum Length 100% Cylinder Length
Minimum Length 50% Cylinder Length
Parameter

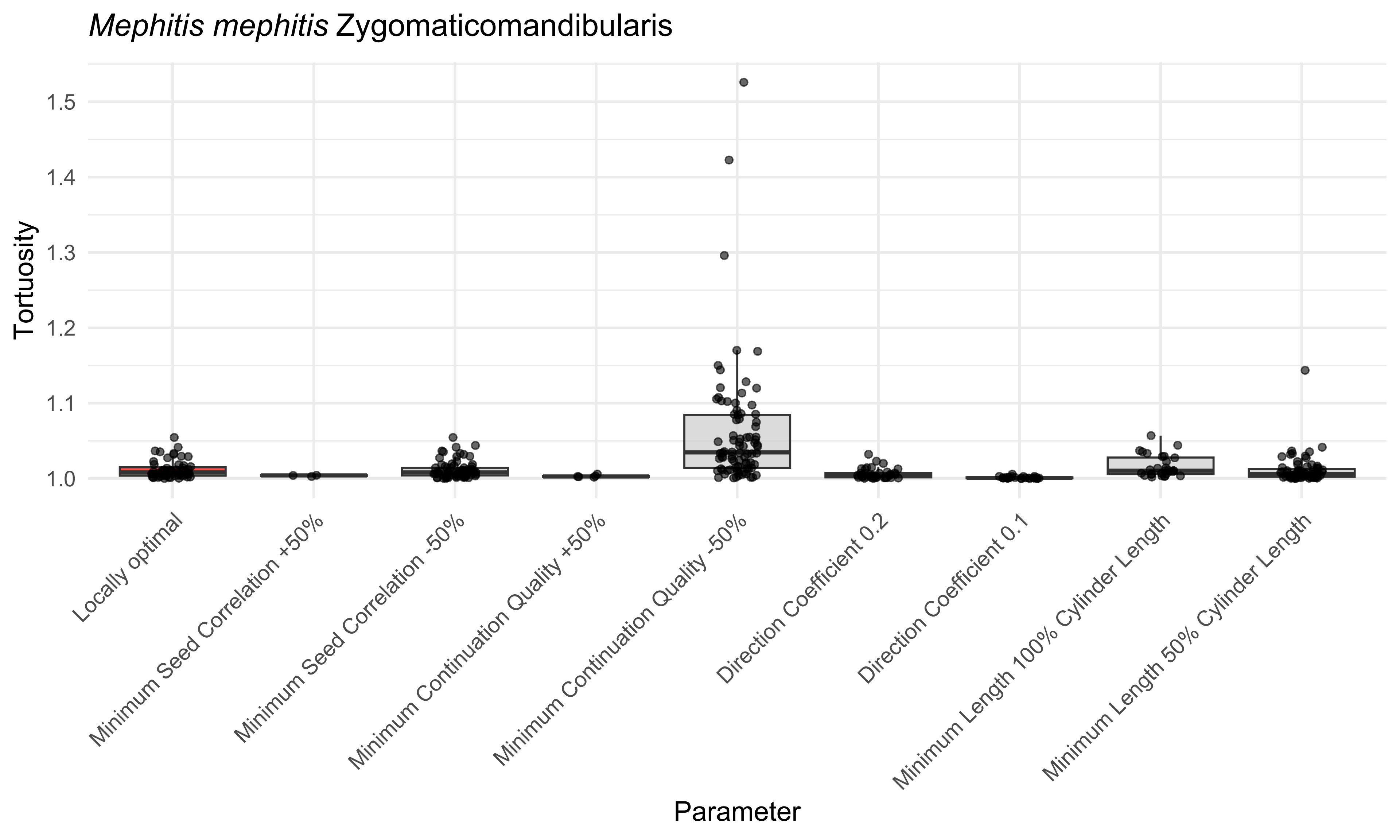
Mephitis mephitis Zygomaticomandibularis
Tortuosity
1.0
1.1
1.2
1.3
1.4
1.5
Locally optimal
Minimum Seed Correlation +50%
Minimum Seed Correlation -50%
Minimum Continuation Quality +50%
Minimum Continuation Quality -50%
Direction Coefficient 0.2
Direction Coefficient 0.1
Minimum Length 100% Cylinder Length
Minimum Length 50% Cylinder Length
Parameter

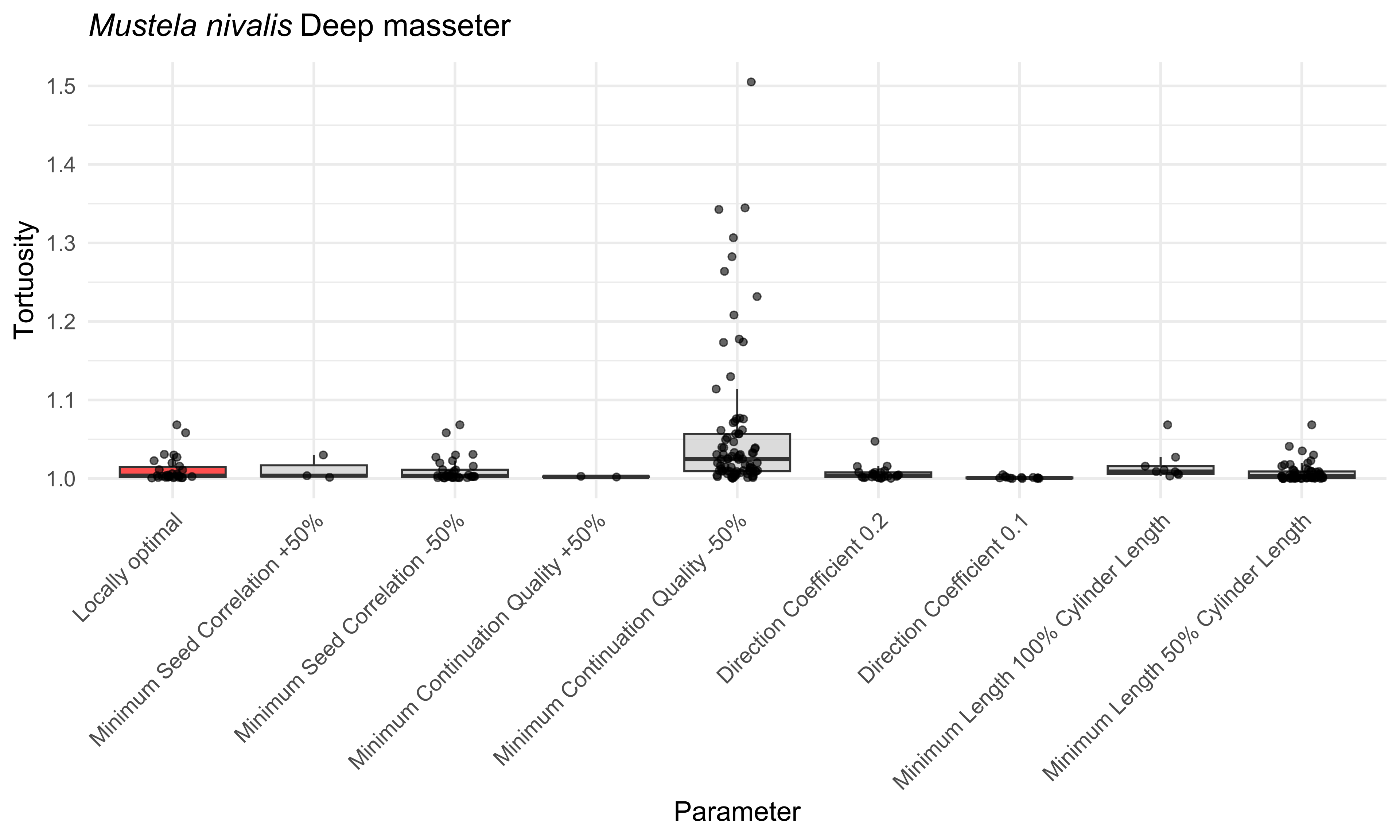

Mustela nivalis Deep masseter
Tortuosity
1.5
1.4
1.3
1.2
1.1
1.0
Locally optimal
Minimum Seed Correlation +50%
Minimum Seed Correlation -50%
Minimum Continuation Quality +50%
Minimum Continuation Quality -50%
Direction Coefficient 0.2
Direction Coefficient 0.1
Minimum Length 100% Cylinder Length
Minimum Length 50% Cylinder Length
Parameter

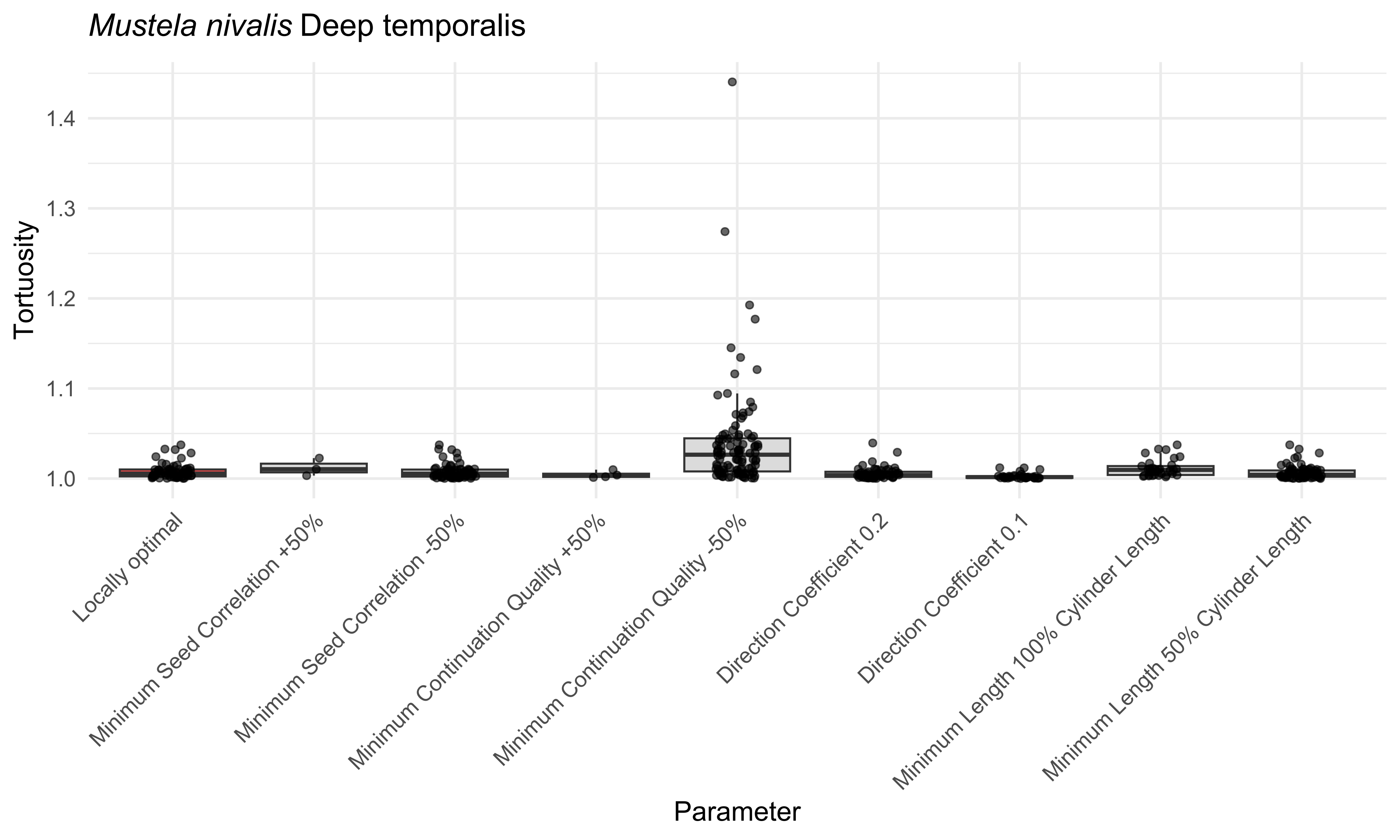

Mustela nivalis Deep temporalis
Tortuosity
1.4
1.3
1.2
1.1
1.0
Locally optimal
Minimum Seed Correlation +50%
Minimum Seed Correlation -50%
Minimum Continuation Quality +50%
Minimum Continuation Quality -50%
Direction Coefficient 0.2
Direction Coefficient 0.1
Minimum Length 100% Cylinder Length
Minimum Length 50% Cylinder Length
Parameter

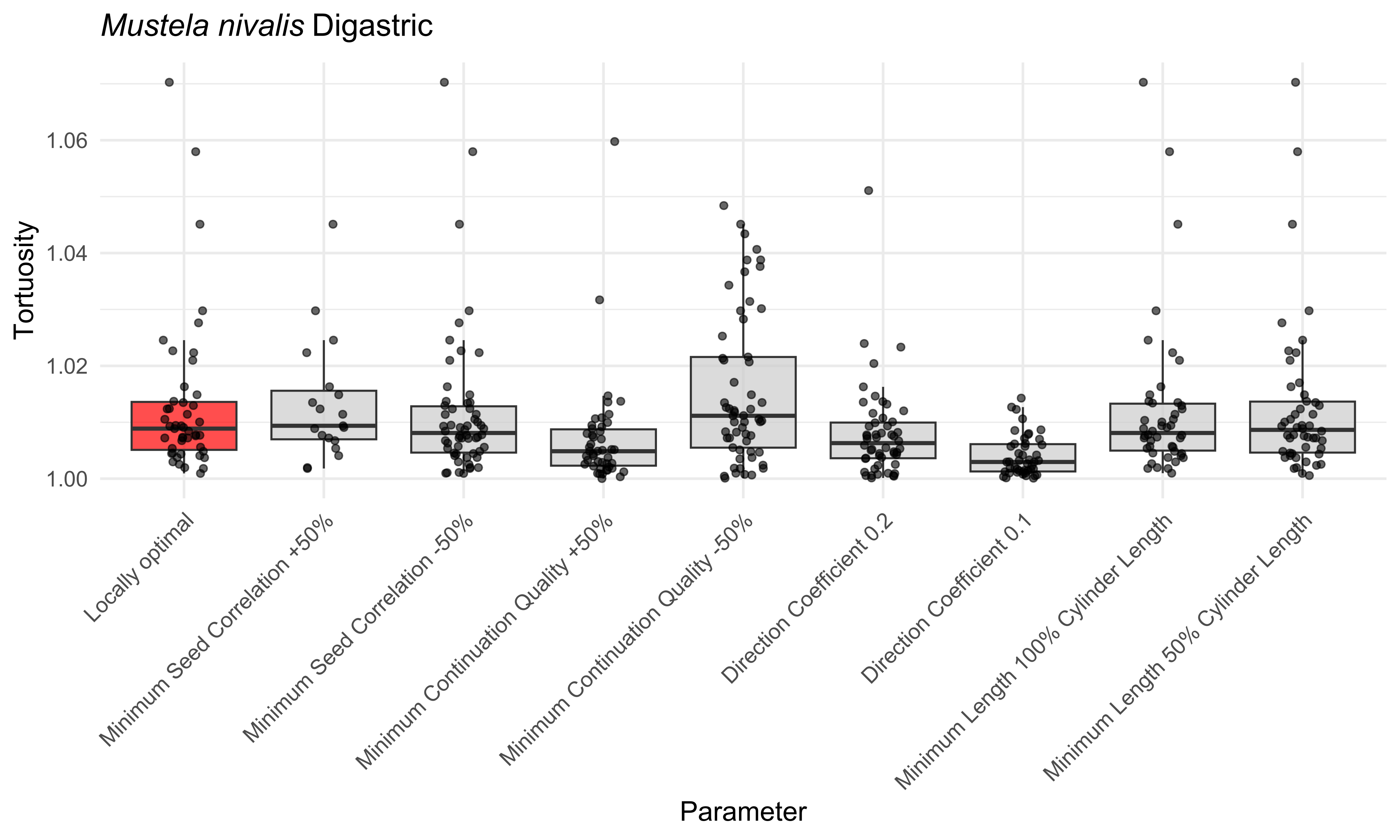
Mustela nivalis Digastric
Tortuosity
1.06
1.04
1.02
1.00
Parameter
Locally optimal
Minimum Seed Correlation +50%
Minimum Seed Correlation -50%
Minimum Continuation Quality +50%
Minimum Continuation Quality -50%
Direction Coefficient 0.2
Direction Coefficient 0.1
Minimum Length 100% Cylinder Length
Minimum Length 50% Cylinder Length

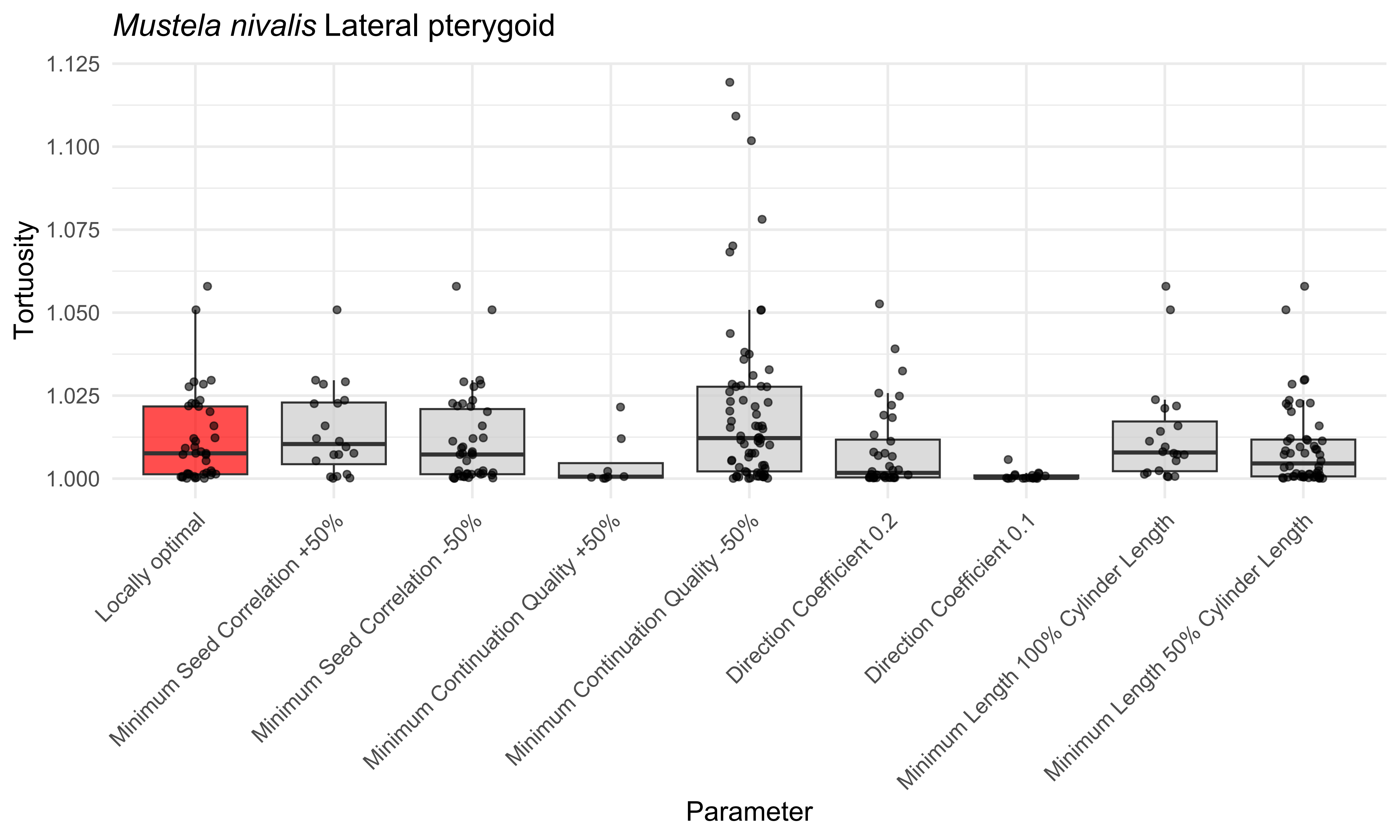

Mustela nivalis Lateral pterygoid
Tortuosity
1.125
1.100
1.075
1.050
1.025
1.000
Locally optimal
Minimum Seed Correlation +50%
Minimum Seed Correlation -50%
Minimum Continuation Quality +50%
Minimum Continuation Quality -50%
Direction Coefficient 0.2
Direction Coefficient 0.1
Minimum Length 100% Cylinder Length
Minimum Length 50% Cylinder Length
Parameter

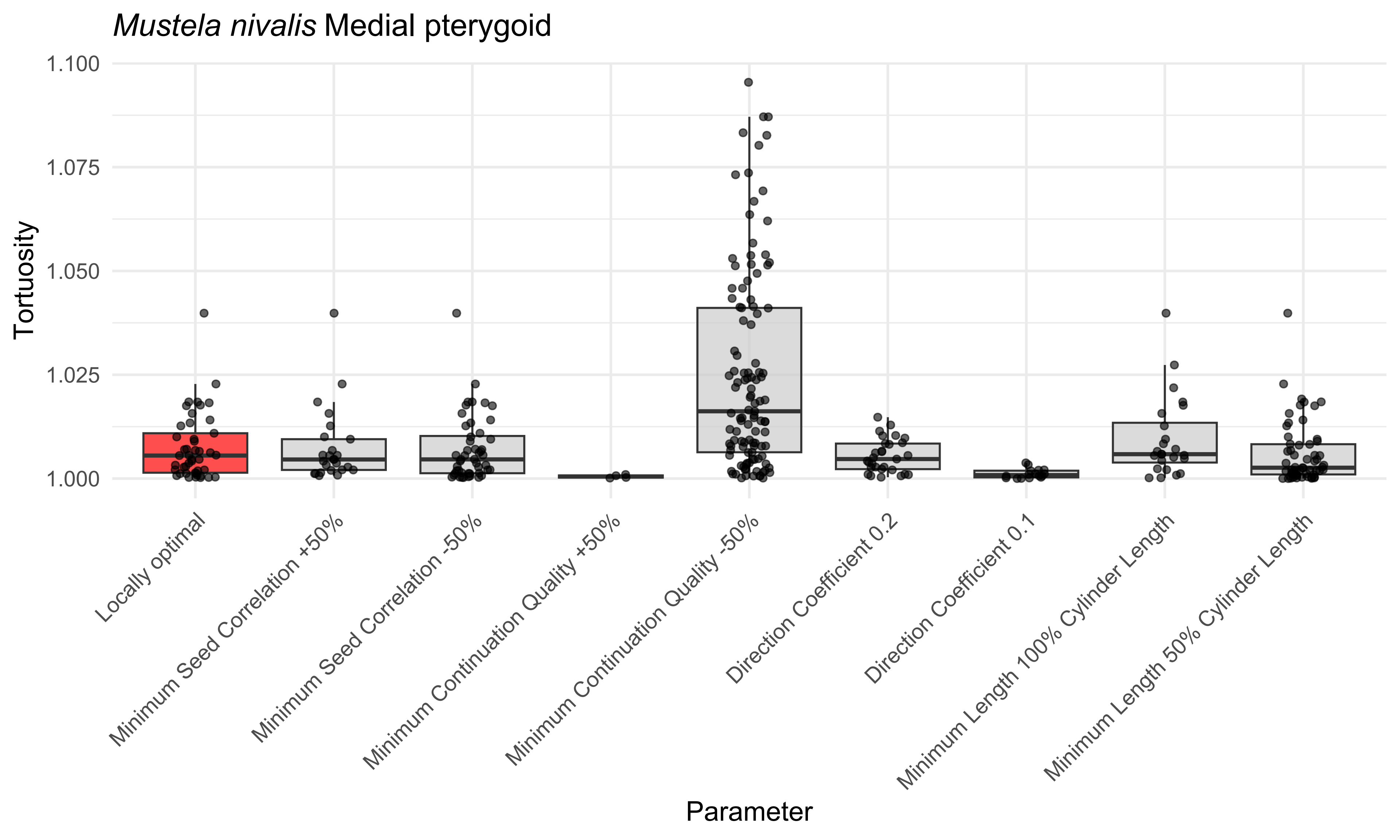
Mustela nivalis Medial pterygoid
Tortuosity
1.100
1.075
1.050
1.025
1.000
Locally optimal
Minimum Seed Correlation +50%
Minimum Seed Correlation -50%
Minimum Continuation Quality +50%
Minimum Continuation Quality -50%
Direction Coefficient 0.2
Direction Coefficient 0.1
Minimum Length 100% Cylinder Length
Minimum Length 50% Cylinder Length
Parameter

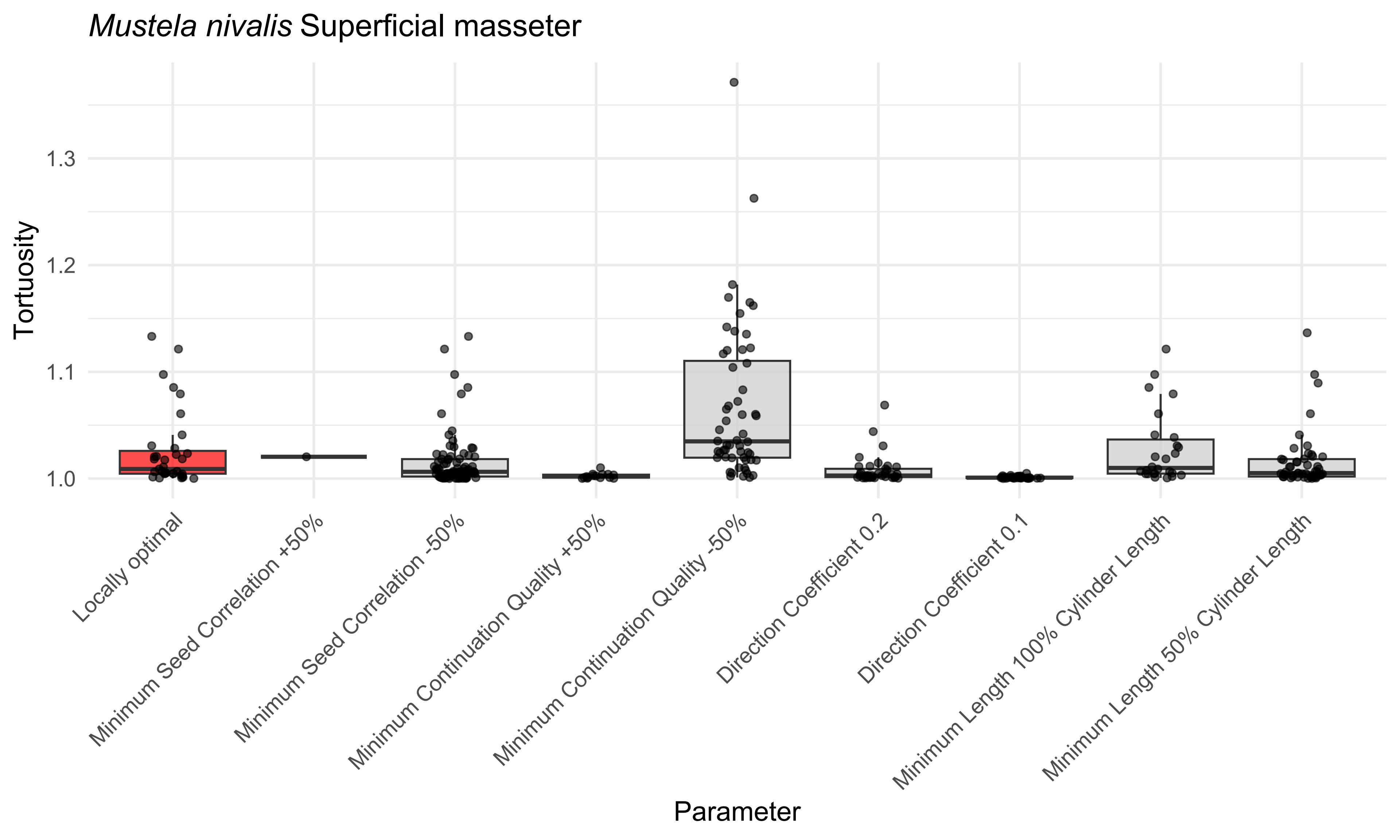

Mustela nivalis Superficial masseter
Tortuosity
1.3
1.2
1.1
1.0
Locally optimal
Minimum Seed Correlation +50%
Minimum Seed Correlation -50%
Minimum Continuation Quality +50%
Minimum Continuation Quality -50%
Direction Coefficient 0.2
Direction Coefficient 0.1
Minimum Length 100% Cylinder Length
Minimum Length 50% Cylinder Length
Parameter

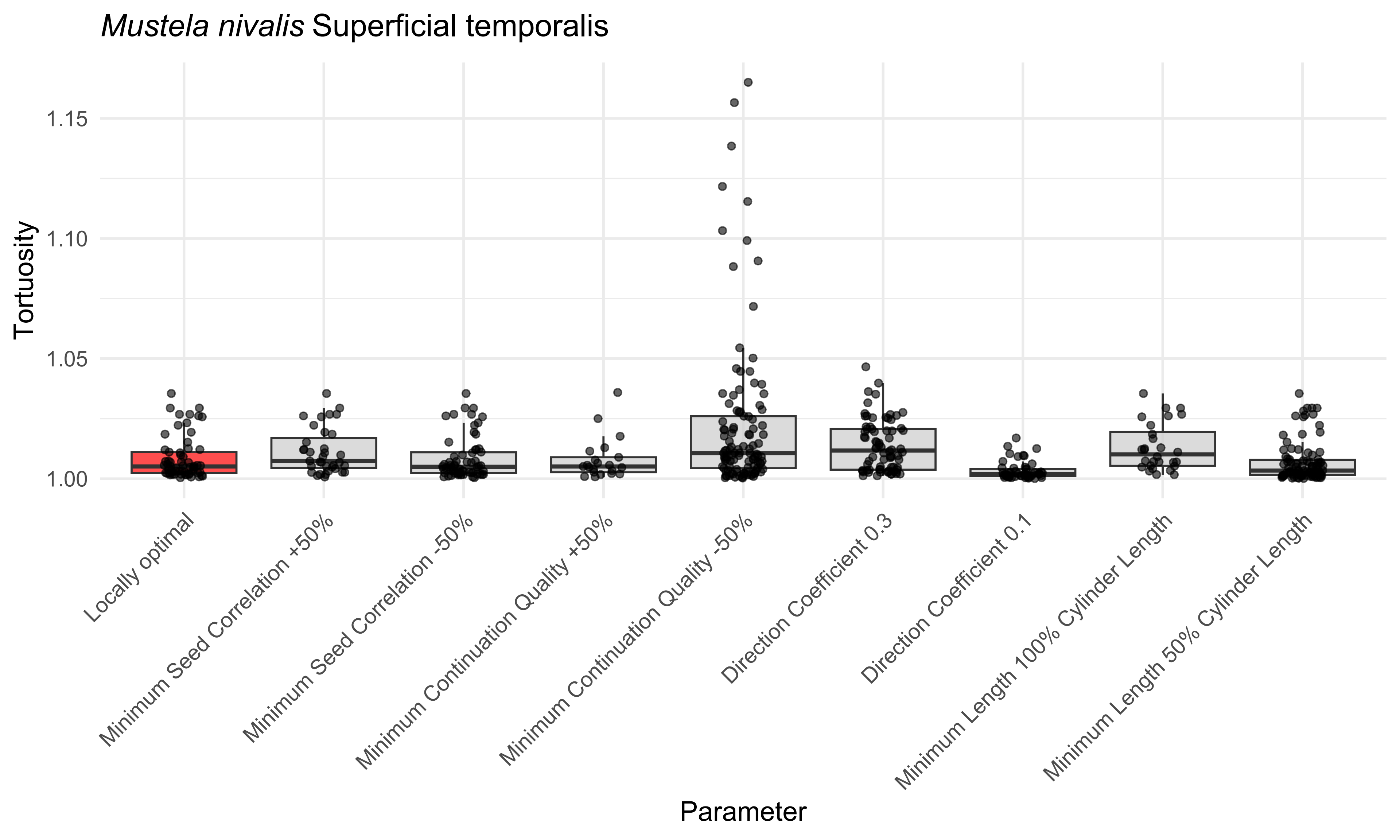

Mustela nivalis Superficial temporalis
Tortuosity
1.15
1.10
1.05
1.00
Locally optimal
Minimum Seed Correlation +50%
Minimum Seed Correlation -50%
Minimum Continuation Quality +50%
Minimum Continuation Quality -50%
Direction Coefficient 0.3
Direction Coefficient 0.1
Minimum Length 100% Cylinder Length
Minimum Length 50% Cylinder Length
Parameter

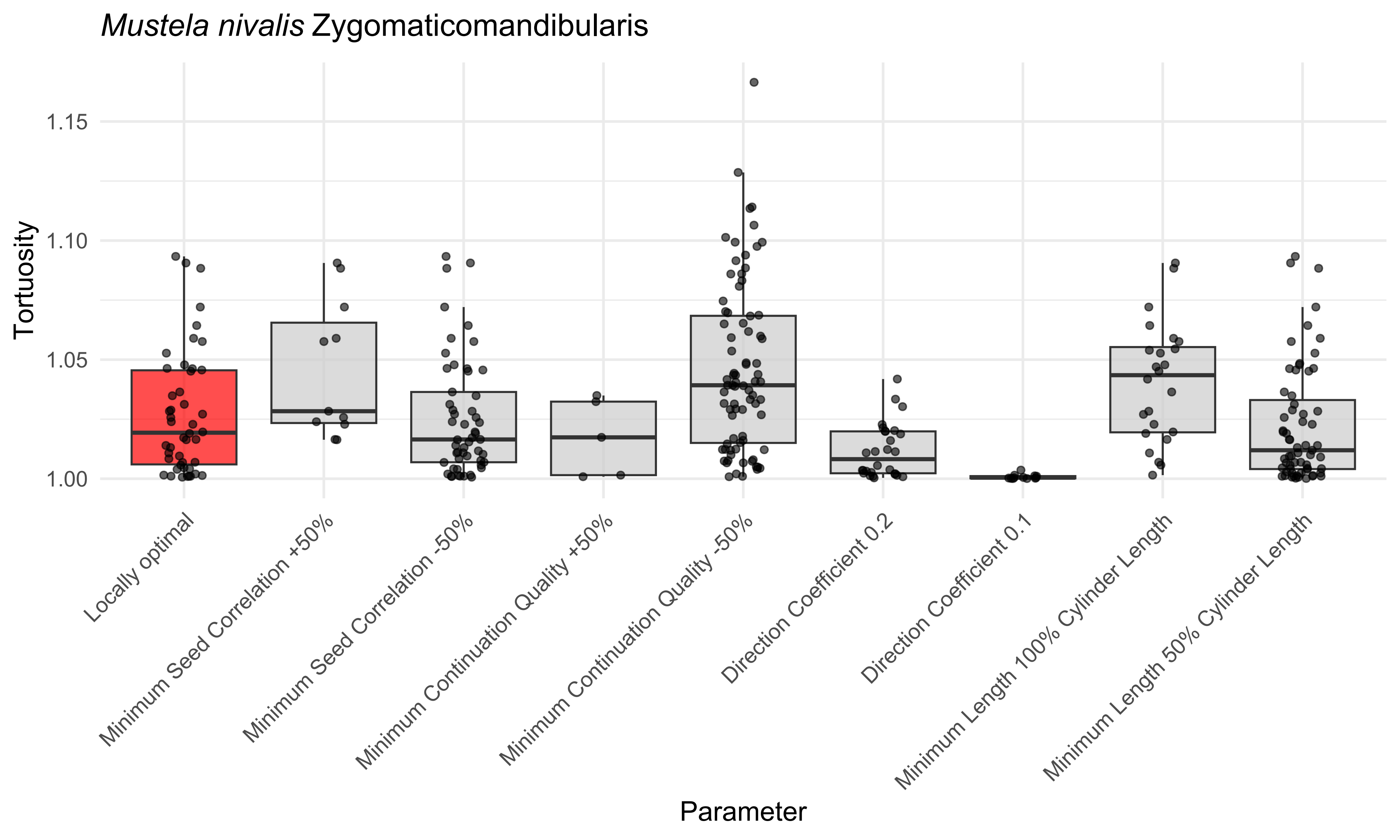
Mustela nivalis Zygomaticomandibularis
Tortuosity
1.15
1.10
1.05
1.00
Locally optimal
Minimum Seed Correlation +50%
Minimum Seed Correlation -50%
Minimum Continuation Quality +50%
Minimum Continuation Quality -50%
Direction Coefficient 0.2
Direction Coefficient 0.1
Minimum Length 100% Cylinder Length
Minimum Length 50% Cylinder Length
Parameter

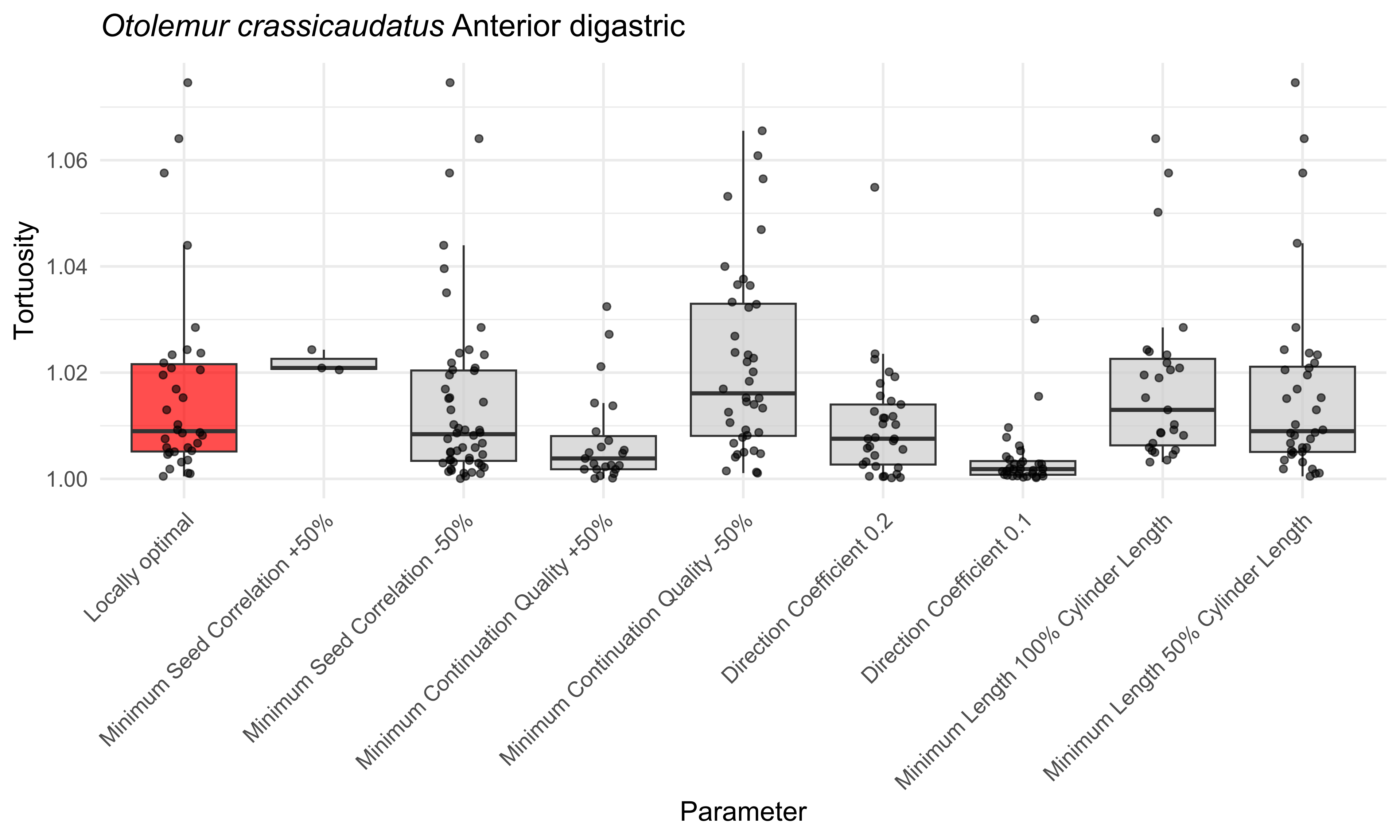

Otolemur crassicaudatus Anterior digastric
Tortuosity
1.00
1.02
1.04
1.06
Locally optimal
Minimum Seed Correlation +50%
Minimum Seed Correlation -50%
Minimum Continuation Quality +50%
Minimum Continuation Quality -50%
Direction Coefficient 0.2
Direction Coefficient 0.1
Minimum Length 100% Cylinder Length
Minimum Length 50% Cylinder Length
Parameter

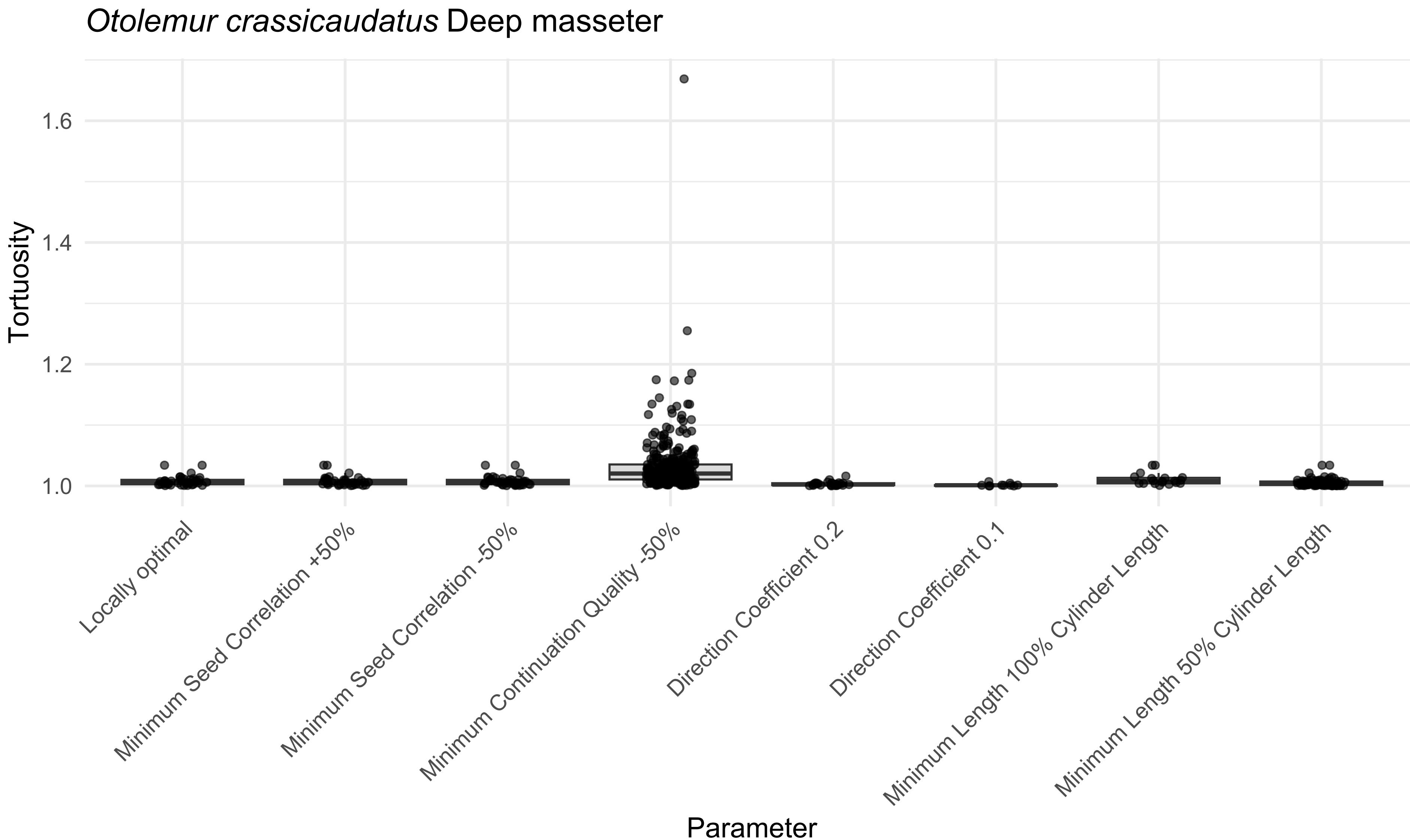

Otolemur crassicaudatus Deep masseter
Tortuosity
1.6
1.4
1.2
1.0
Locally optimal
Minimum Seed Correlation +50%
Minimum Seed Correlation -50%
Minimum Continuation Quality -50%
Direction Coefficient 0.2
Direction Coefficient 0.1
Minimum Length 100% Cylinder Length
Minimum Length 50% Cylinder Length
Parameter

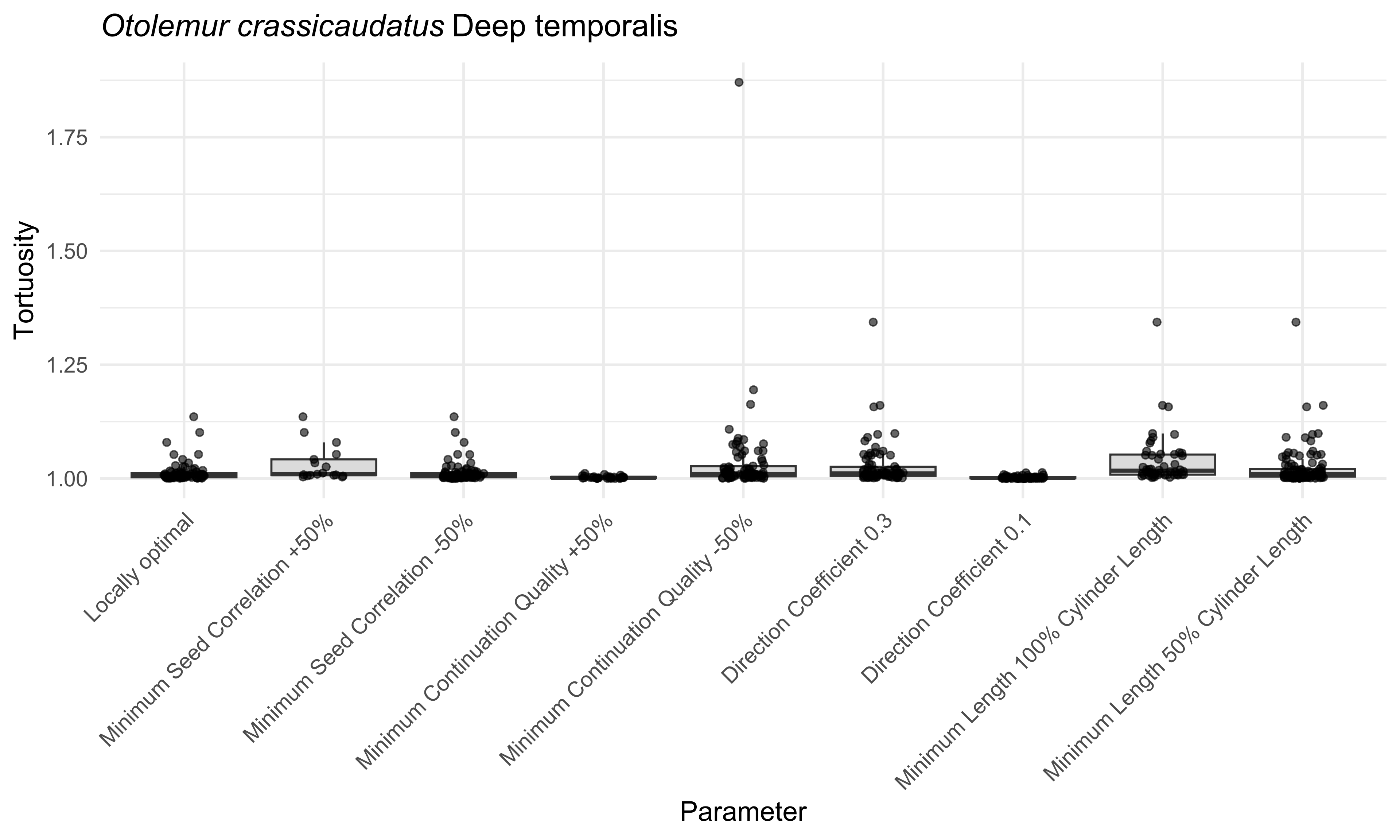

Otolemur crassicaudatus Deep temporalis
Tortuosity
1.75
1.50
1.25
1.00
Locally optimal
Minimum Seed Correlation +50%
Minimum Seed Correlation -50%
Minimum Continuation Quality +50%
Minimum Continuation Quality -50%
Direction Coefficient 0.3
Direction Coefficient 0.1
Minimum Length 100% Cylinder Length
Minimum Length 50% Cylinder Length
Parameter

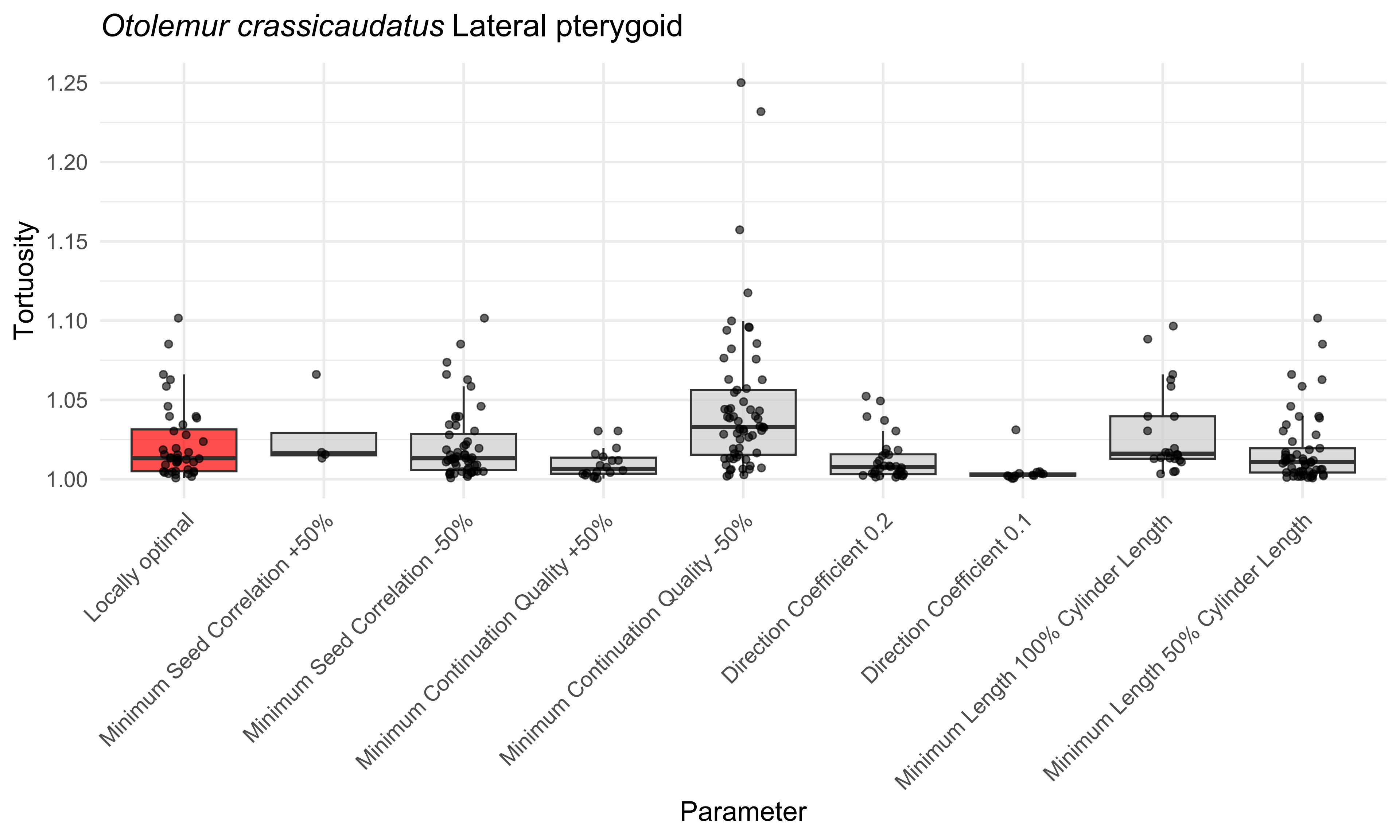
Otolemur crassicaudatus Lateral pterygoid
Tortuosity
1.25
1.20
1.15
1.10
1.05
1.00
Locally optimal
Minimum Seed Correlation +50%
Minimum Seed Correlation -50%
Minimum Continuation Quality +50%
Minimum Continuation Quality -50%
Direction Coefficient 0.2
Direction Coefficient 0.1
Minimum Length 100% Cylinder Length
Minimum Length 50% Cylinder Length
Parameter

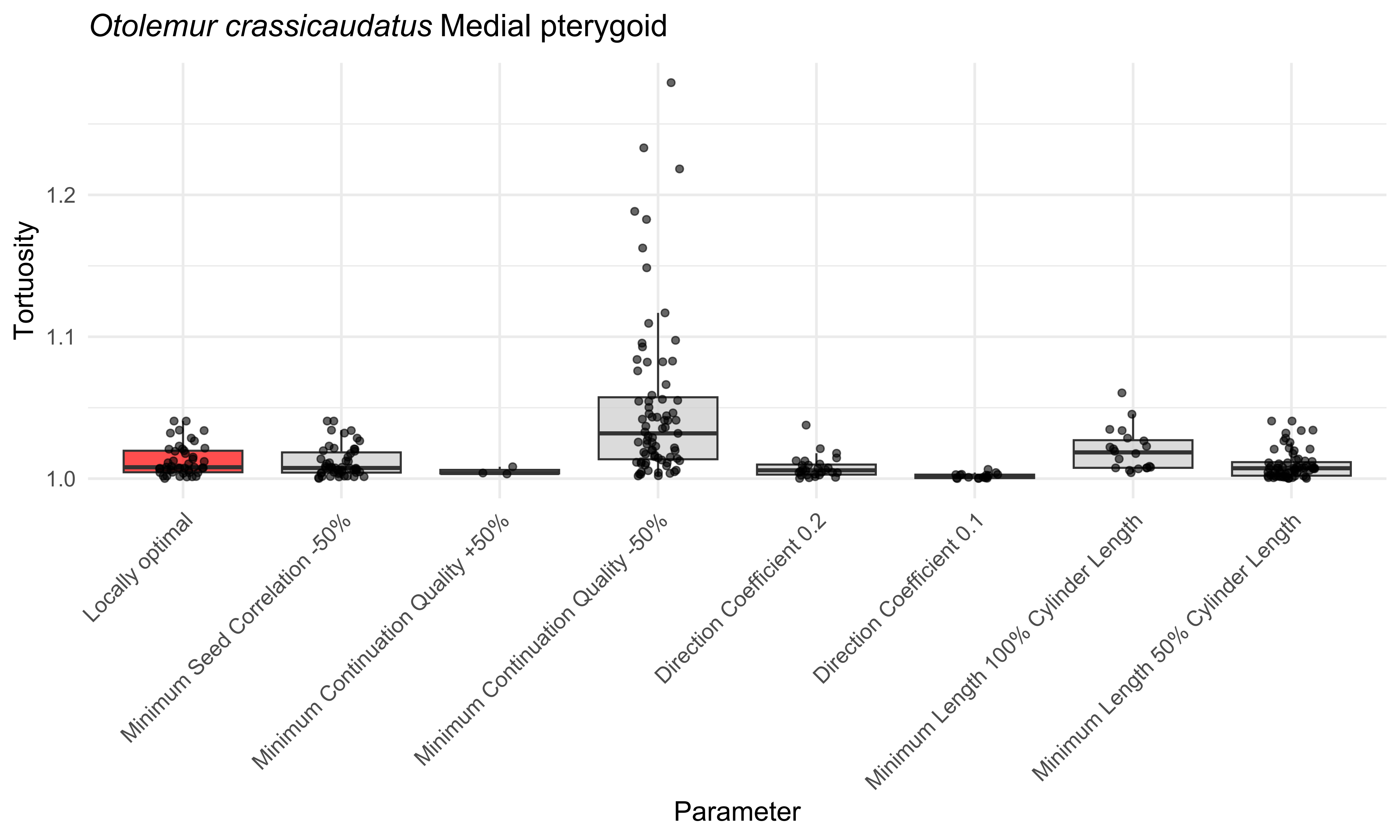
Otolemur crassicaudatus Medial pterygoid
Tortuosity
1.2
1.1
1.0
Locally optimal
Minimum Seed Correlation -50%
Minimum Continuation Quality +50%
Minimum Continuation Quality -50%
Direction Coefficient 0.2
Direction Coefficient 0.1
Minimum Length 100% Cylinder Length
Minimum Length 50% Cylinder Length
Parameter

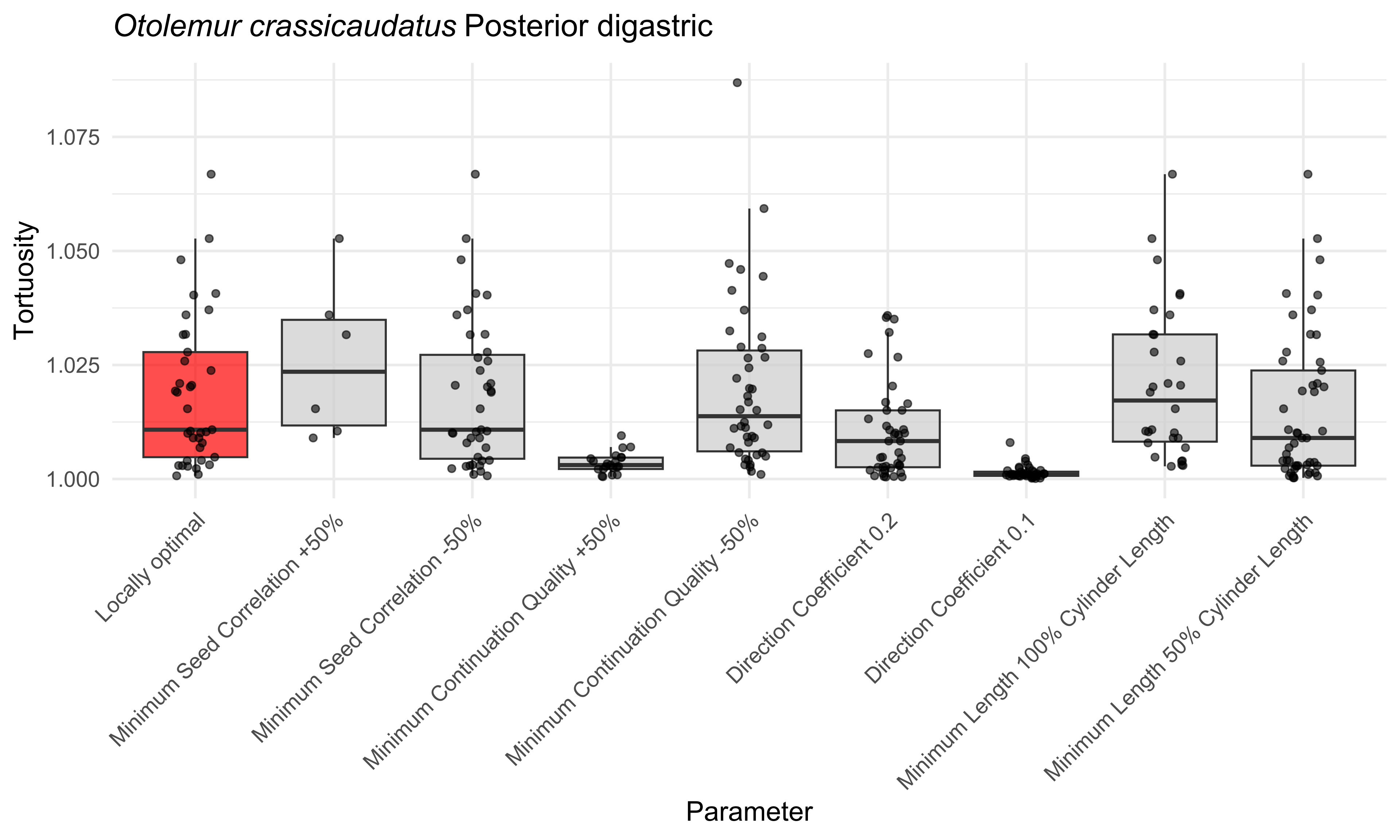

Otolemur crassicaudatus Posterior digastric
Tortuosity
1.075
1.050
1.025
1.000
Locally optimal
Minimum Seed Correlation +50%
Minimum Seed Correlation -50%
Minimum Continuation Quality +50%
Minimum Continuation Quality -50%
Direction Coefficient 0.2
Direction Coefficient 0.1
Minimum Length 100% Cylinder Length
Minimum Length 50% Cylinder Length
Parameter

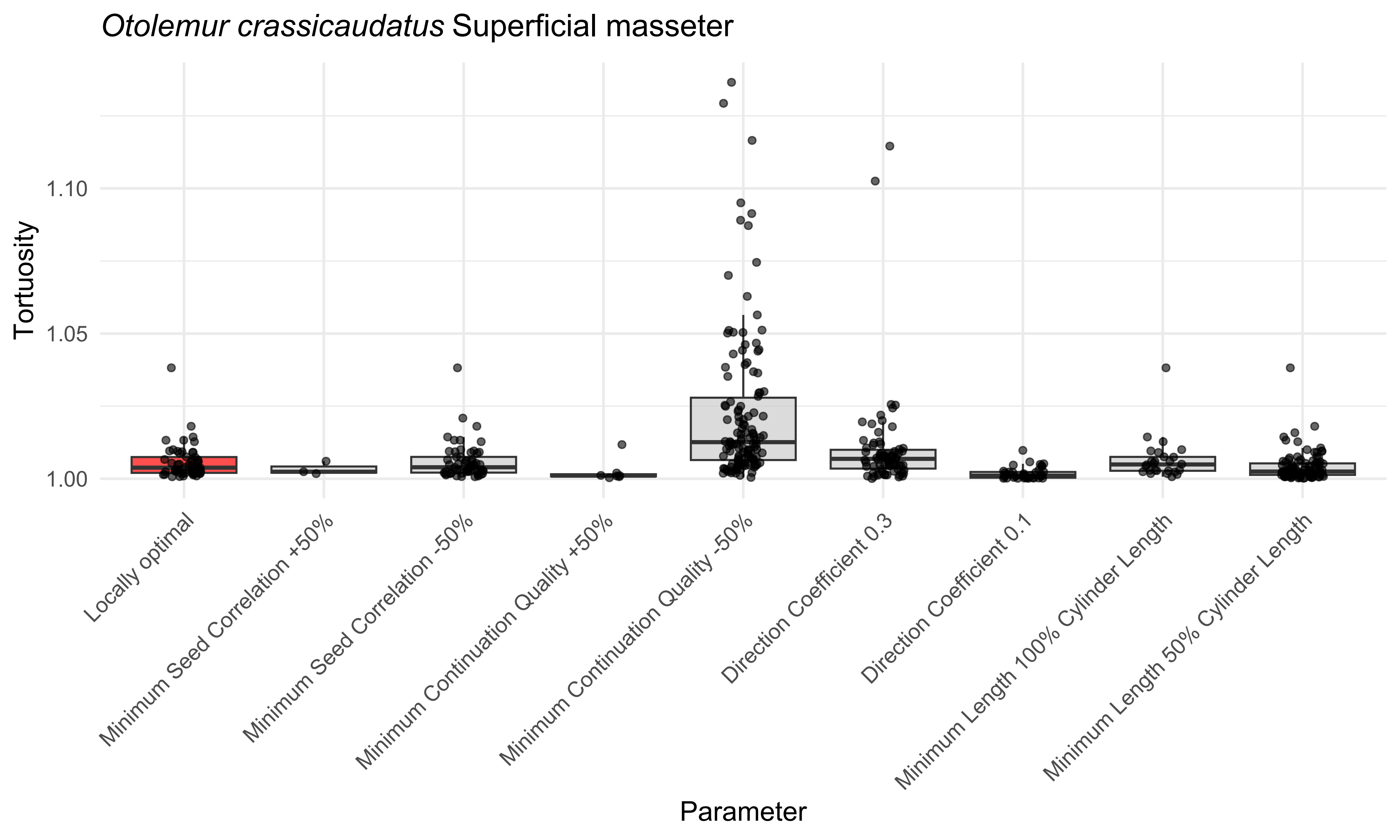
Otolemur crassicaudatus Superficial masseter
Tortuosity
1.10
1.05
1.00
Locally optimal
Minimum Seed Correlation +50%
Minimum Seed Correlation -50%
Minimum Continuation Quality +50%
Minimum Continuation Quality -50%
Direction Coefficient 0.3
Direction Coefficient 0.1
Minimum Length 100% Cylinder Length
Minimum Length 50% Cylinder Length
Parameter

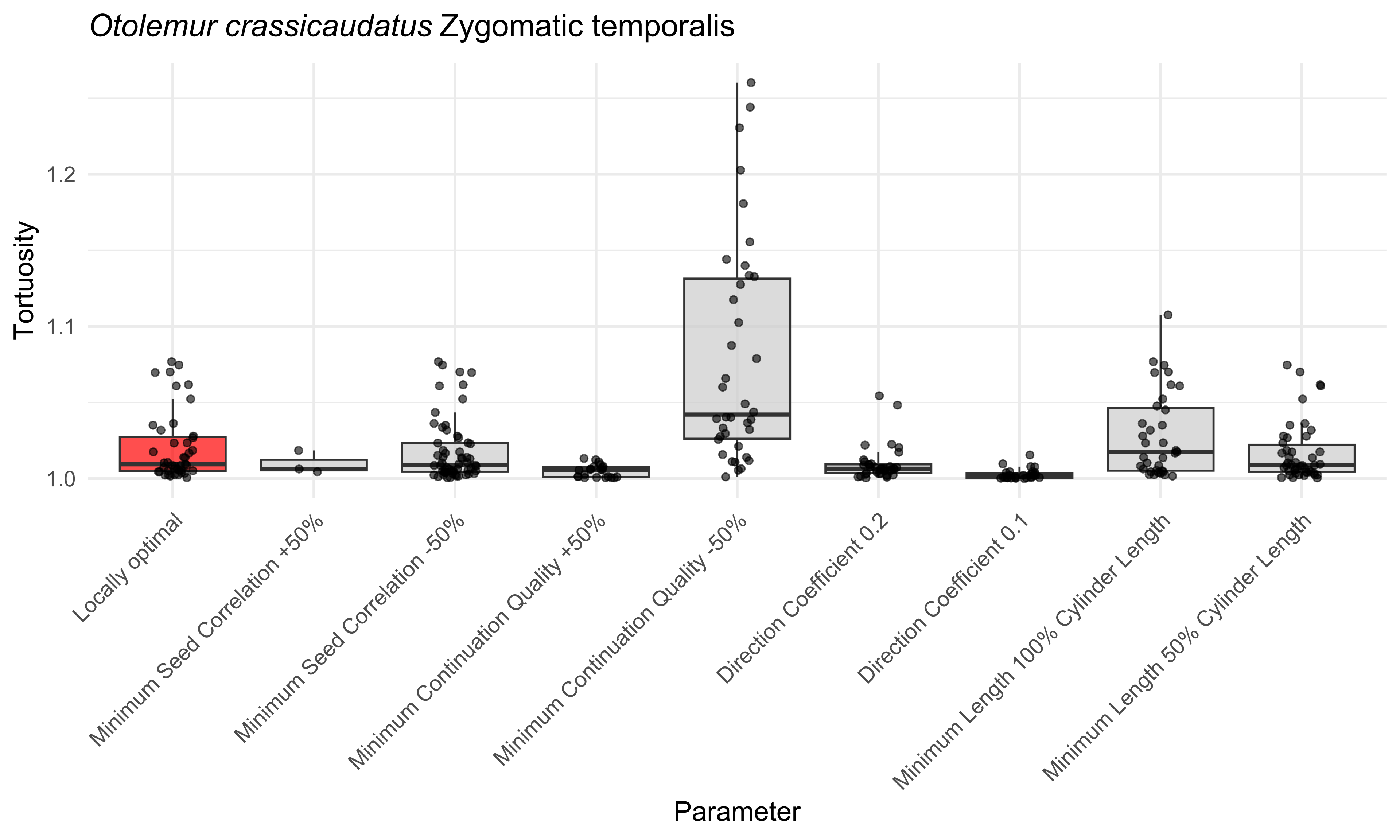

Otolemur crassicaudatus Zygomatic temporalis
Tortuosity
1.2
1.1
1.0
Locally optimal
Minimum Seed Correlation +50%
Minimum Seed Correlation -50%
Minimum Continuation Quality +50%
Minimum Continuation Quality -50%
Direction Coefficient 0.2
Direction Coefficient 0.1
Minimum Length 100% Cylinder Length
Minimum Length 50% Cylinder Length
Parameter

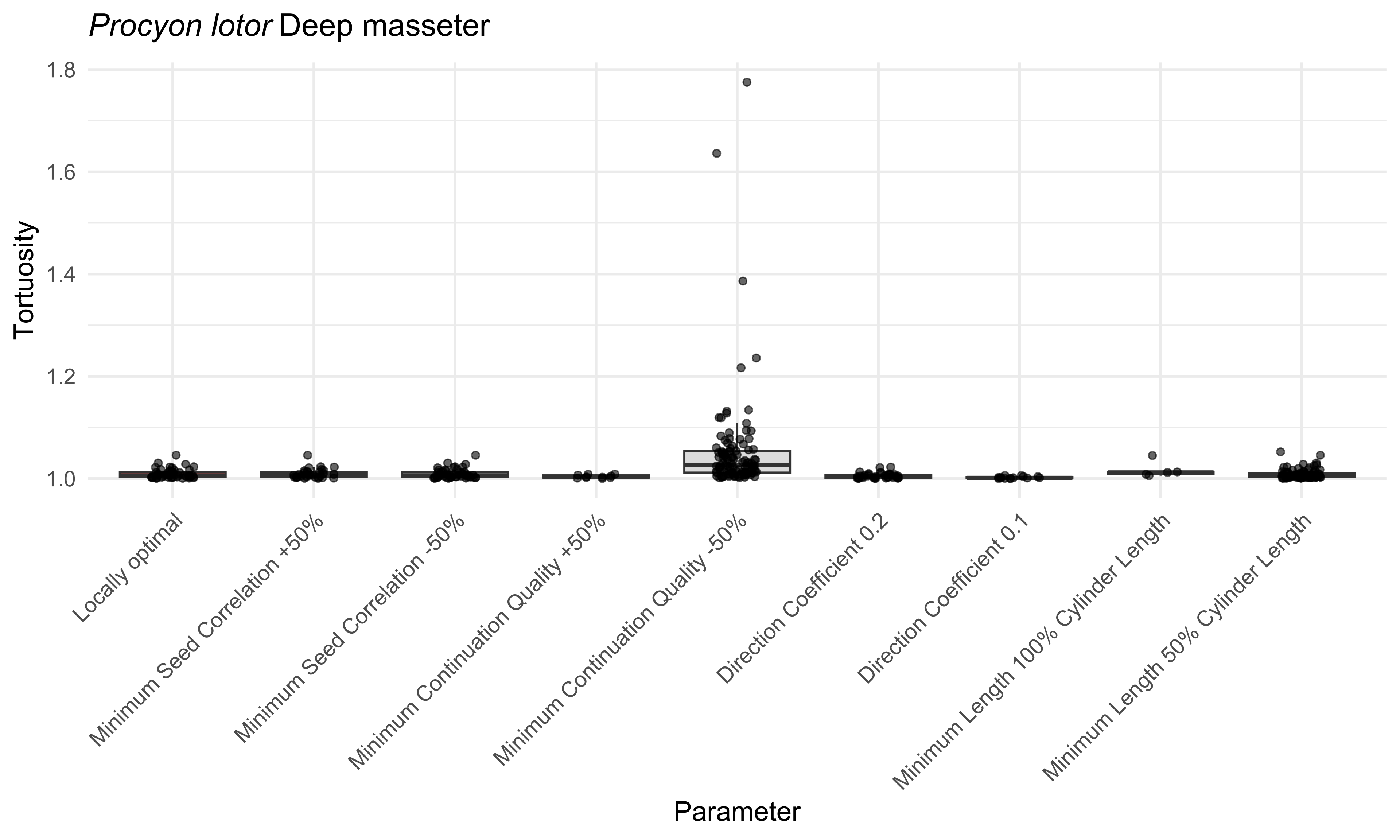

Procyon lotor Deep masseter
Tortuosity
1.8
1.6
1.4
1.2
1.0
Locally optimal
Minimum Seed Correlation +50%
Minimum Seed Correlation -50%
Minimum Continuation Quality +50%
Minimum Continuation Quality -50%
Direction Coefficient 0.2
Direction Coefficient 0.1
Minimum Length 100% Cylinder Length
Minimum Length 50% Cylinder Length
Parameter

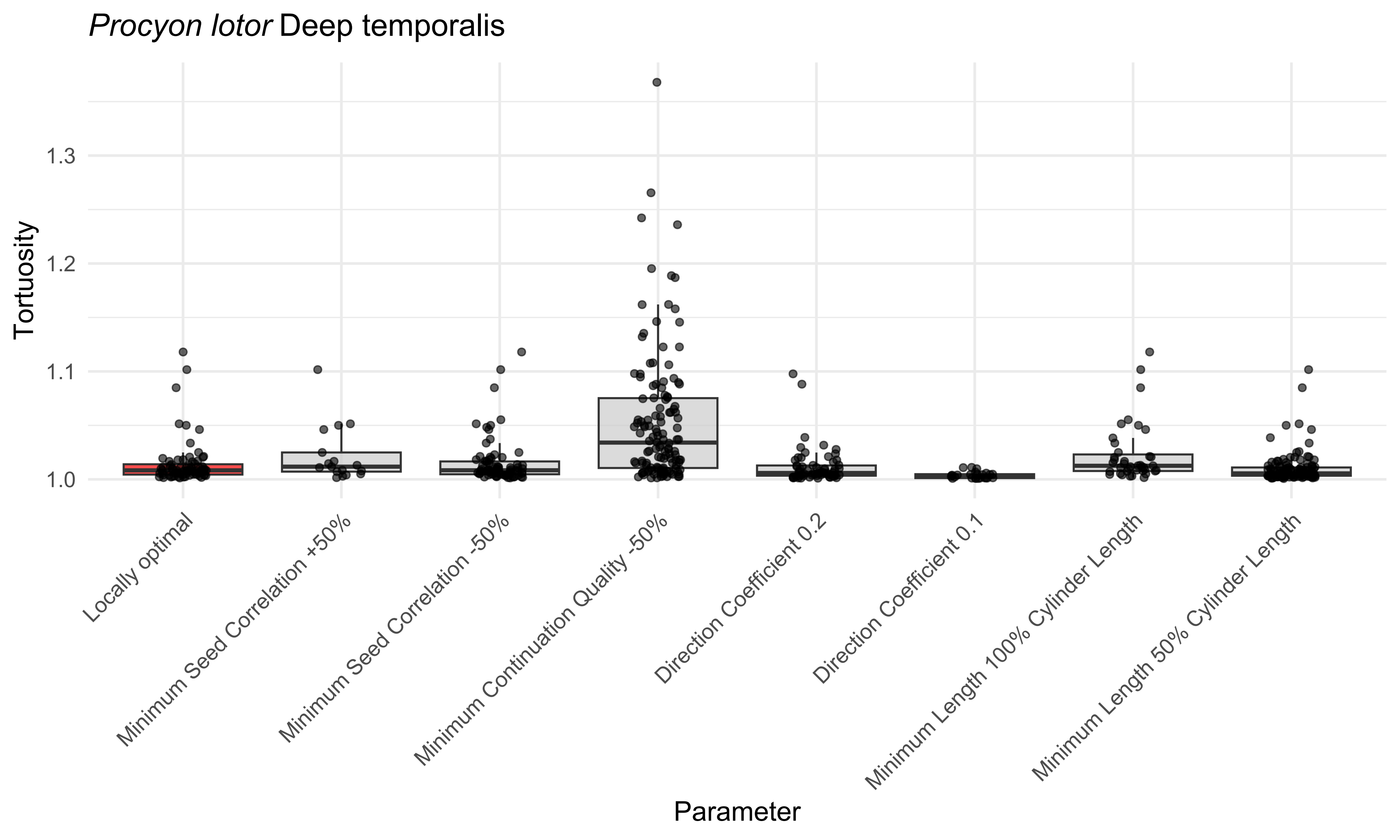

Procyon lotor Deep temporalis
Tortuosity
1.3
1.2
1.1
1.0
Locally optimal
Minimum Seed Correlation +50%
Minimum Seed Correlation -50%
Minimum Continuation Quality -50%
Direction Coefficient 0.2
Direction Coefficient 0.1
Minimum Length 100% Cylinder Length
Minimum Length 50% Cylinder Length
Parameter

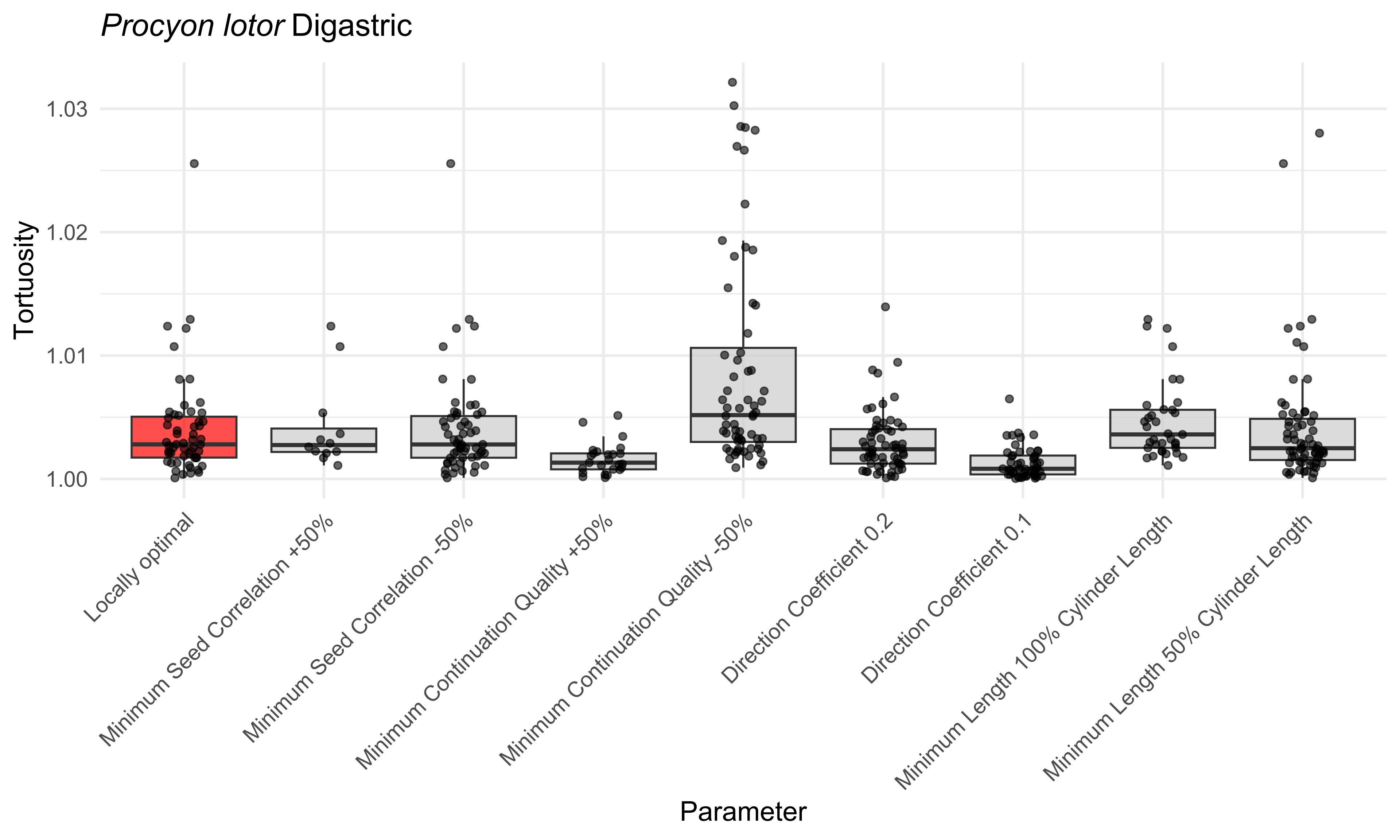
Procyon lotor Digastric
Tortuosity
1.03
1.02
1.01
1.00
Locally optimal
Minimum Seed Correlation +50%
Minimum Seed Correlation -50%
Minimum Continuation Quality +50%
Minimum Continuation Quality -50%
Direction Coefficient 0.2
Direction Coefficient 0.1
Minimum Length 100% Cylinder Length
Minimum Length 50% Cylinder Length
Parameter

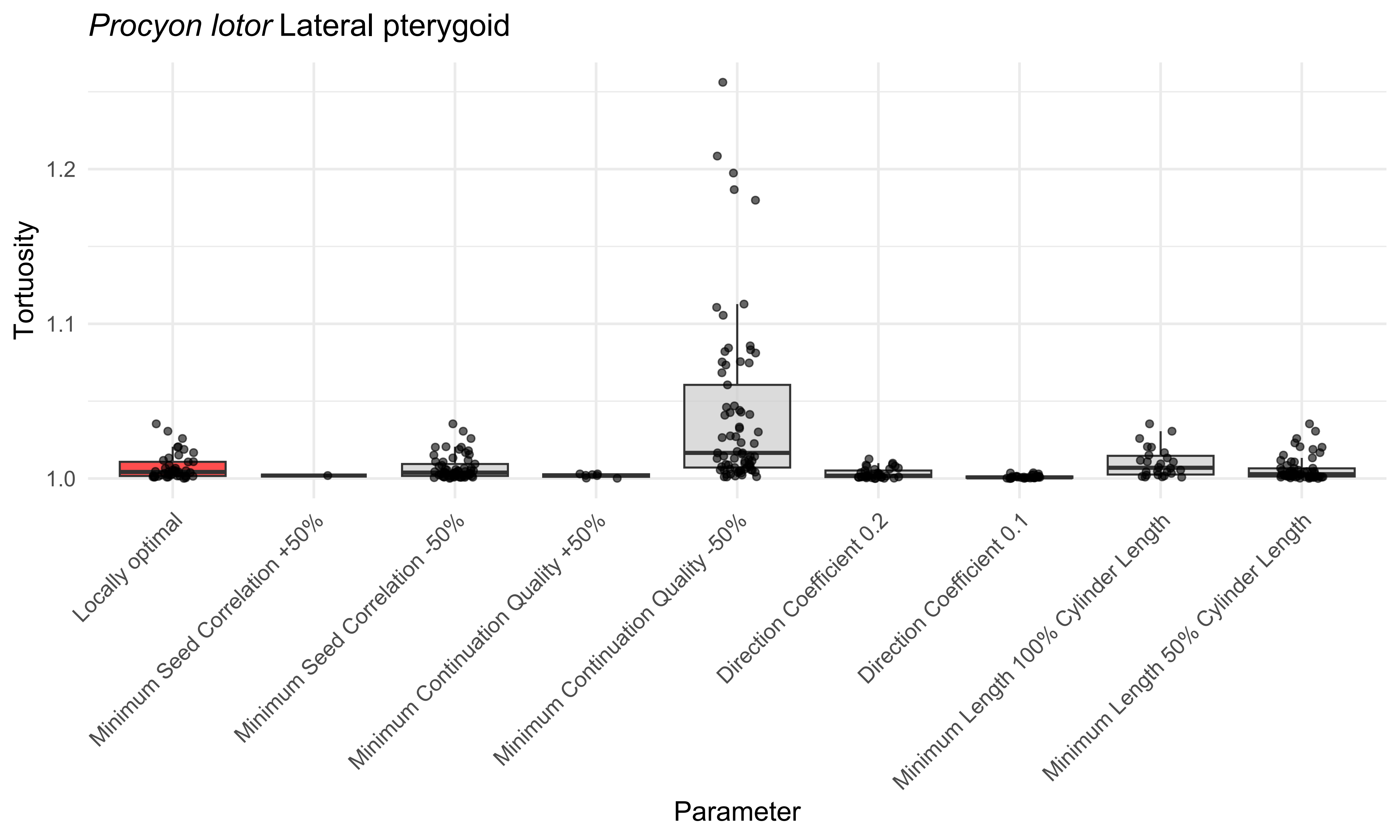

Procyon lotor Lateral pterygoid
Tortuosity
1.2
1.1
1.0
Locally optimal
Minimum Seed Correlation +50%
Minimum Seed Correlation -50%
Minimum Continuation Quality +50%
Minimum Continuation Quality -50%
Direction Coefficient 0.2
Direction Coefficient 0.1
Minimum Length 100% Cylinder Length
Minimum Length 50% Cylinder Length
Parameter

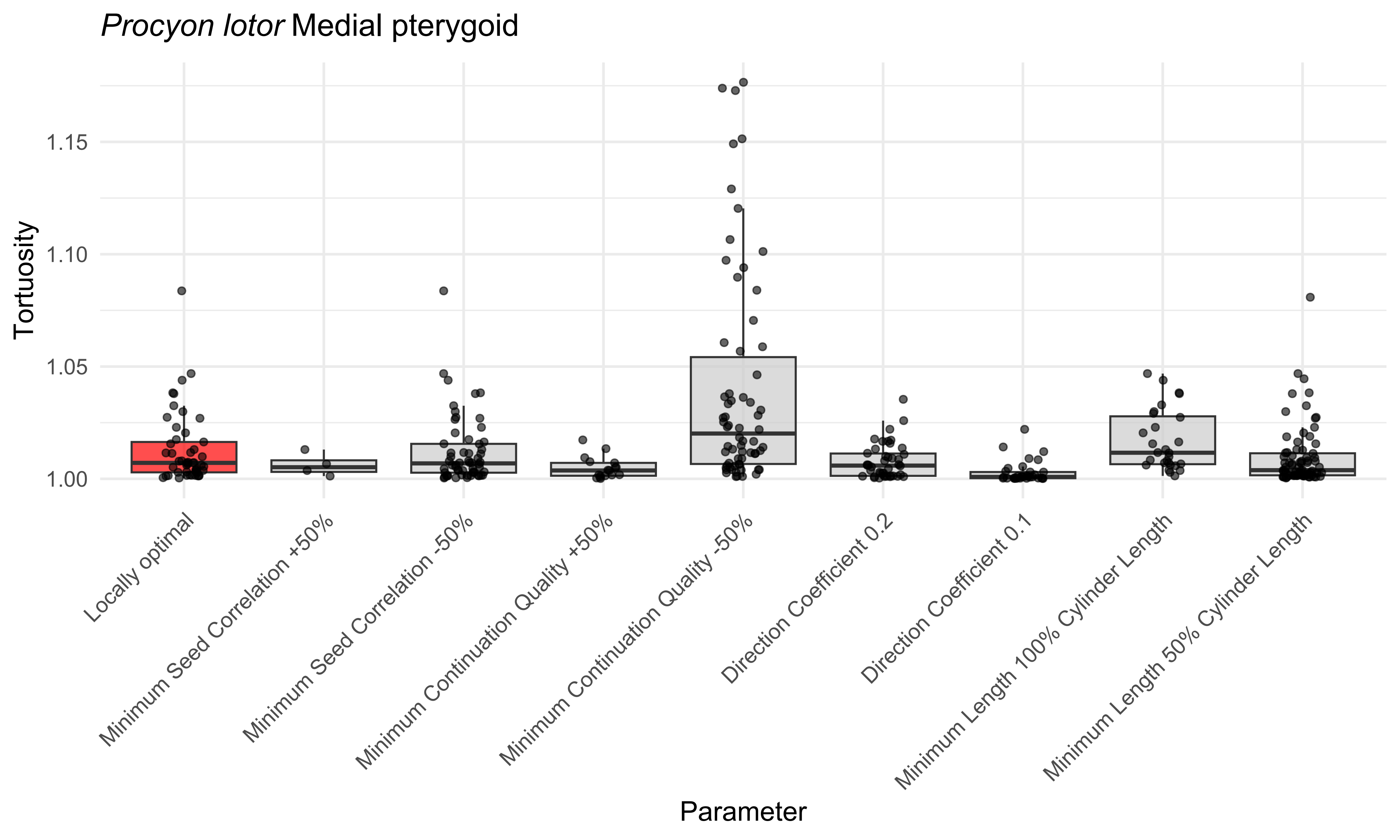

Procyon lotor Medial pterygoid
Tortuosity
1.15
1.10
1.05
1.00
Locally optimal
Minimum Seed Correlation +50%
Minimum Seed Correlation -50%
Minimum Continuation Quality +50%
Minimum Continuation Quality -50%
Direction Coefficient 0.2
Direction Coefficient 0.1
Minimum Length 100% Cylinder Length
Minimum Length 50% Cylinder Length
Parameter

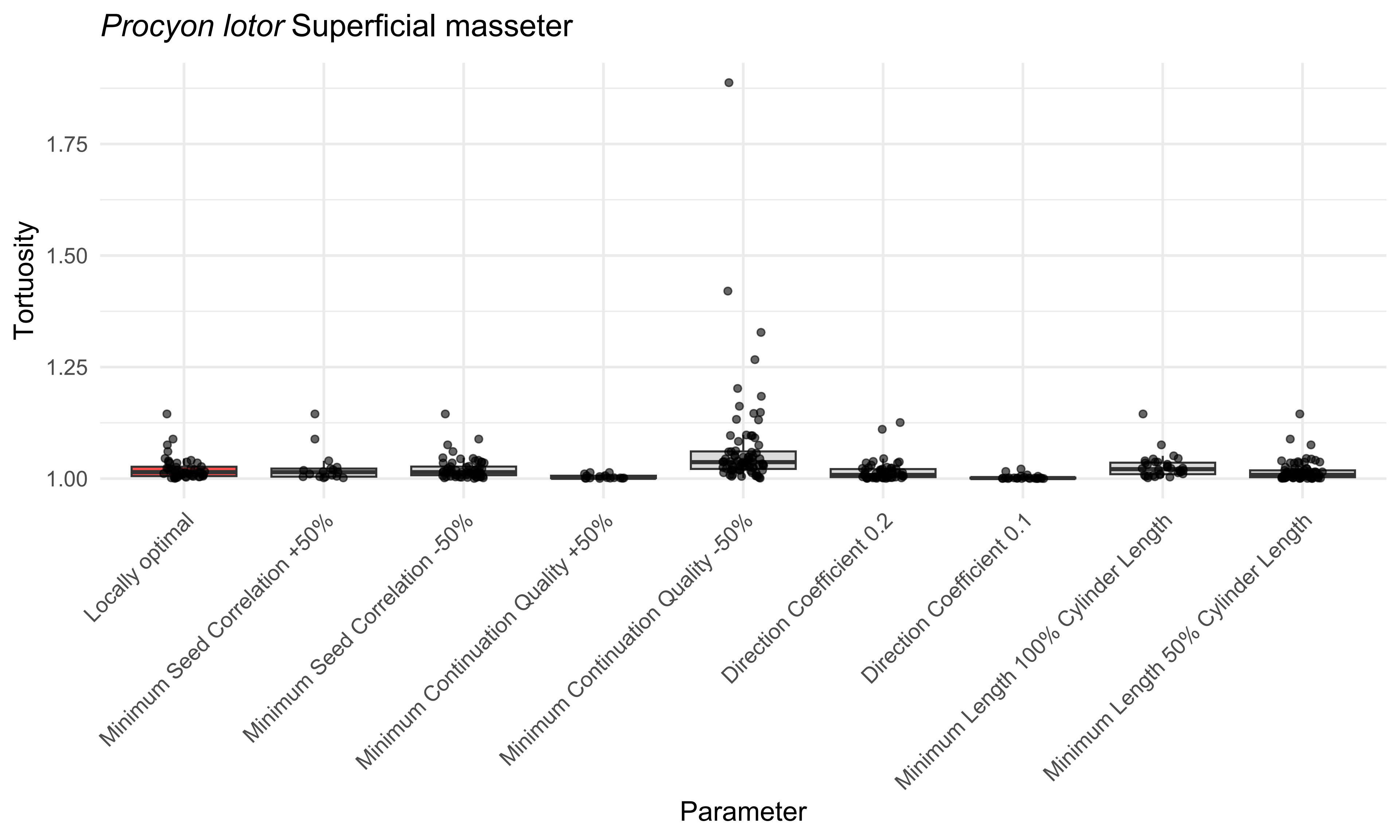

Procyon lotor Superficial masseter
Tortuosity
1.75
1.50
1.25
1.00
Locally optimal
Minimum Seed Correlation +50%
Minimum Seed Correlation -50%
Minimum Continuation Quality +50%
Minimum Continuation Quality -50%
Direction Coefficient 0.2
Direction Coefficient 0.1
Minimum Length 100% Cylinder Length
Minimum Length 50% Cylinder Length
Parameter

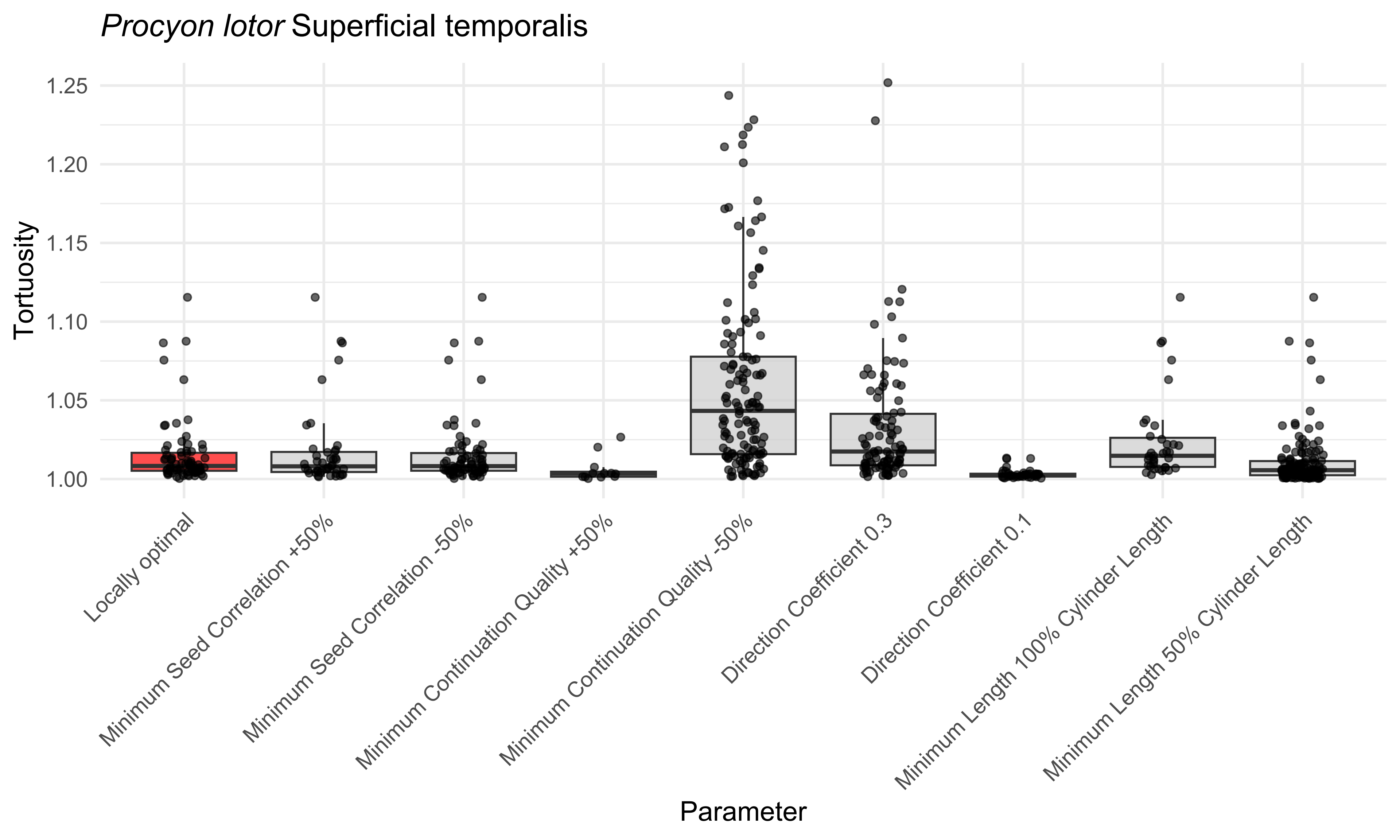
Procyon lotor Superficial temporalis
Tortuosity
1.25
1.20
1.15
1.10
1.05
1.00
Locally optimal
Minimum Seed Correlation +50%
Minimum Seed Correlation -50%
Minimum Continuation Quality +50%
Minimum Continuation Quality -50%
Direction Coefficient 0.3
Direction Coefficient 0.1
Minimum Length 100% Cylinder Length
Minimum Length 50% Cylinder Length
Parameter

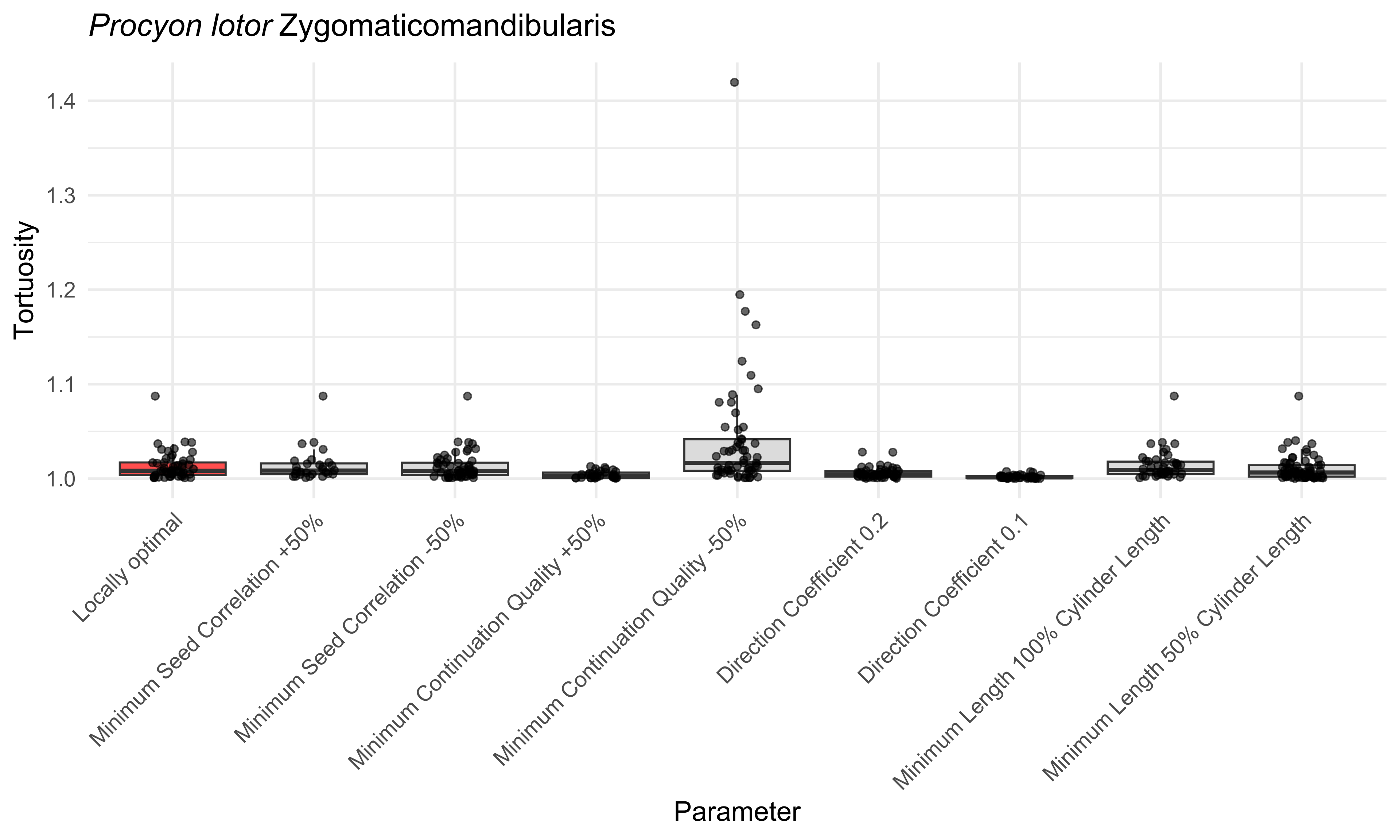

Procyon lotor Zygomaticomandibularis
Tortuosity
1.4
1.3
1.2
1.1
1.0
Locally optimal
Minimum Seed Correlation +50%
Minimum Seed Correlation -50%
Minimum Continuation Quality +50%
Minimum Continuation Quality -50%
Direction Coefficient 0.2
Direction Coefficient 0.1
Minimum Length 100% Cylinder Length
Minimum Length 50% Cylinder Length
Parameter
