# Supplementary material for: Multi‐method analysis for the three‐dimensional reconstruction of muscle fascicles from DiceCT datasets
Source: Anat Rec (Hoboken). 2025 Dec 4;309(12):3491–510. doi: 10.1002/ar.70088 (PMC13749328; doi:10.1002/ar.70088)
Supplement: Supplementary file 3 [file AR-309-3491-s004.pdf]

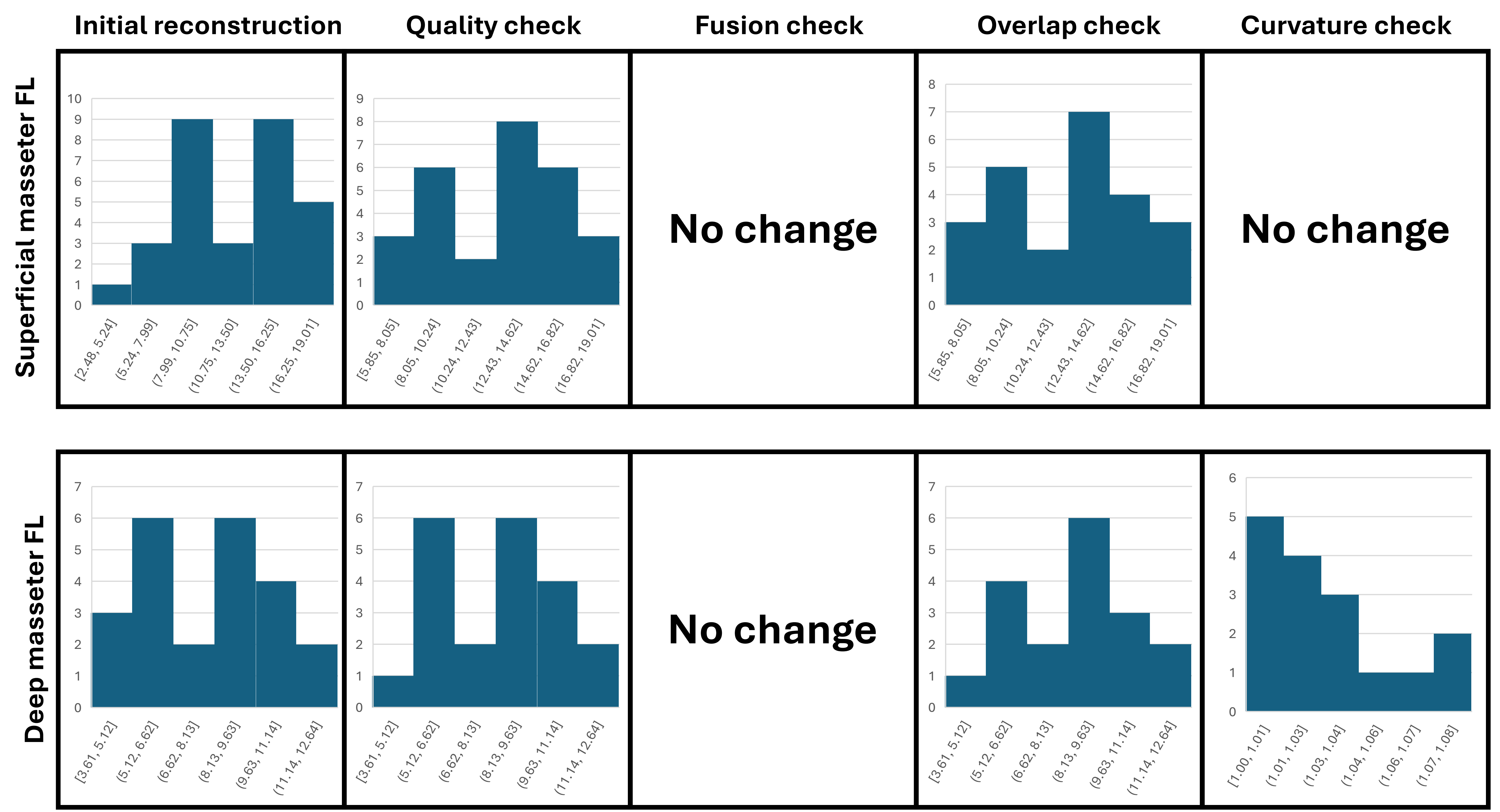
Initial reconstruction
Quality check
Fusion check
Overlap check
Curvature check
Superficial masseter FL
Deep masseter FL
No change
No change
No change
[2.48, 5.24]
(5.24, 7.99]
(7.99, 10.75]
(10.75, 13.50]
(13.50, 16.25]
(16.25, 19.01]
[5.85, 8.05]
(8.05, 10.24]
(10.24, 12.43]
(12.43, 14.62]
(14.62, 16.82]
(16.82, 19.01]
[3.61, 5.12]
(5.12, 6.62]
(6.62, 8.13]
(8.13, 9.63]
(9.63, 11.14]
(11.14, 12.64]
[1.00, 1.01]
(1.01, 1.03]
(1.03, 1.04]
(1.04, 1.06]
(1.06, 1.07]
(1.07, 1.08]

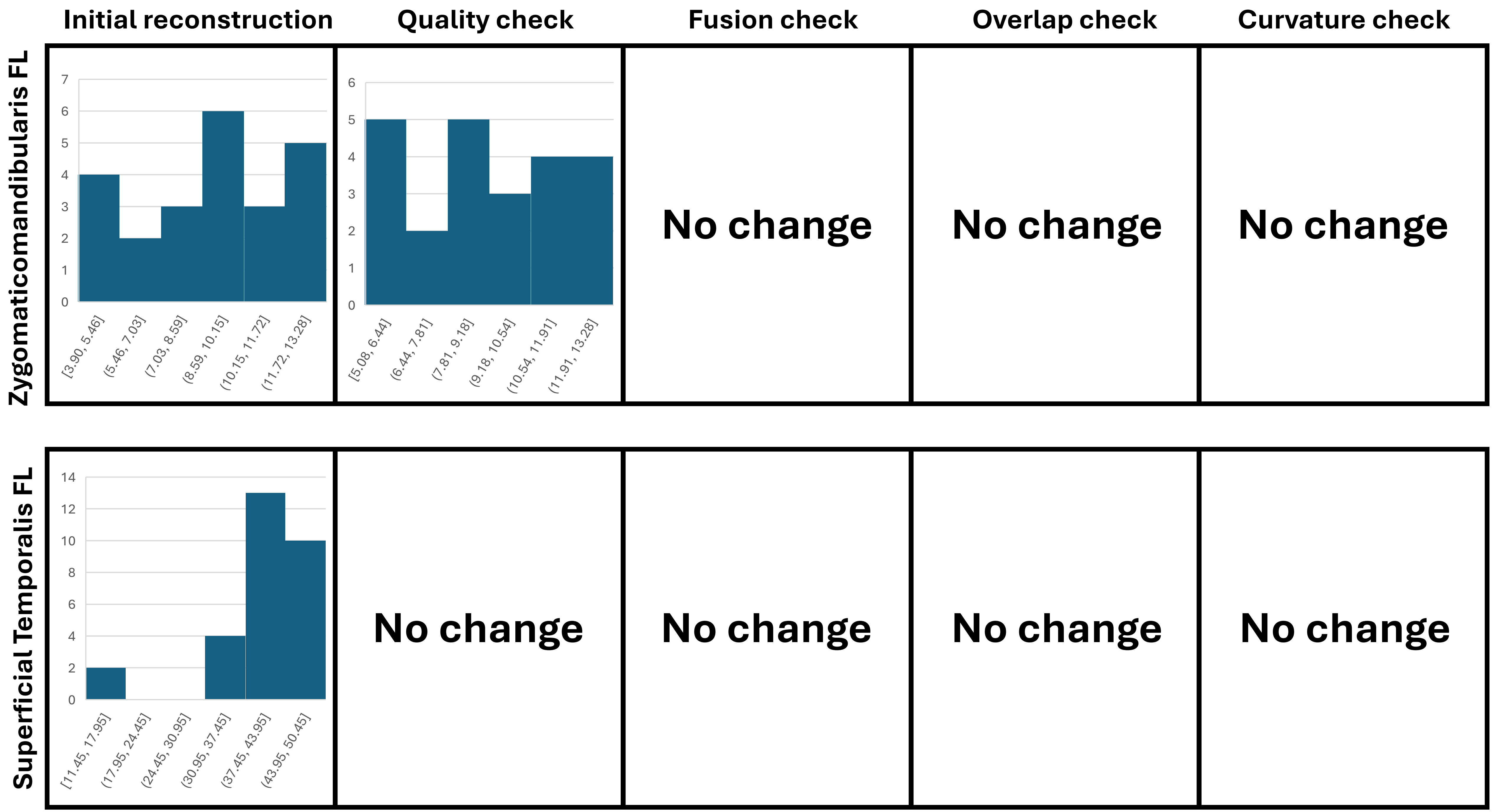

Initial reconstruction
Quality check
Fusion check
Overlap check
Curvature check
Zygomaticomandibularis FL
0
1
2
3
4
5
6
7
[3.90, 5.46]
(5.46, 7.03]
(7.03, 8.59]
(8.59, 10.15]
(10.15, 11.72]
(11.72, 13.28]
0
1
2
3
4
5
6
[5.08, 6.44]
(6.44, 7.81]
(7.81, 9.18]
(9.18, 10.54]
(10.54, 11.91]
(11.91, 13.28]
No change
No change
No change
Superficial Temporalis FL
0
2
4
6
8
10
12
14
[11.45, 17.95]
(17.95, 24.45]
(24.45, 30.95]
(30.95, 37.45]
(37.45, 43.95]
(43.95, 50.45]
No change
No change
No change
No change

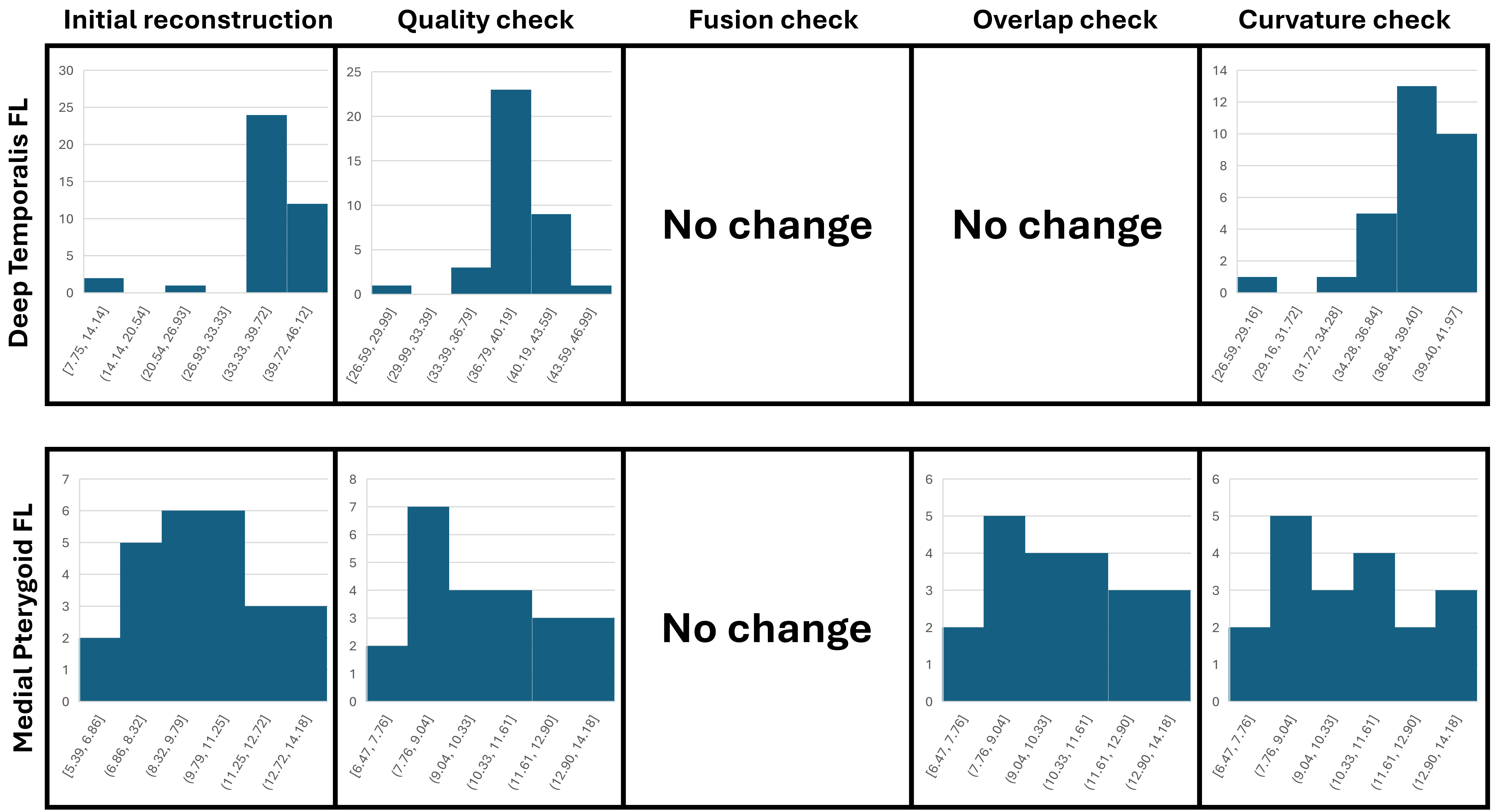

Initial reconstruction
Quality check
Fusion check
Overlap check
Curvature check
Deep Temporalis FL
Medial Pterygoid FL
No change
No change
No change
[7.75, 14.14]
(14.14, 20.54]
(20.54, 26.93]
(26.93, 33.33]
(33.33, 39.72]
(39.72, 46.12]
[26.59, 29.99]
(29.99, 33.39]
(33.39, 36.79]
(36.79, 40.19]
(40.19, 43.59]
(43.59, 46.99]
[26.59, 29.16]
(29.16, 31.72]
(31.72, 34.28]
(34.28, 36.84]
(36.84, 39.40]
(39.40, 41.97]
[5.39, 6.86]
(6.86, 8.32]
(8.32, 9.79]
(9.79, 11.25]
(11.25, 12.72]
(12.72, 14.18]
[6.47, 7.76]
(7.76, 9.04]
(9.04, 10.33]
(10.33, 11.61]
(11.61, 12.90]
(12.90, 14.18]

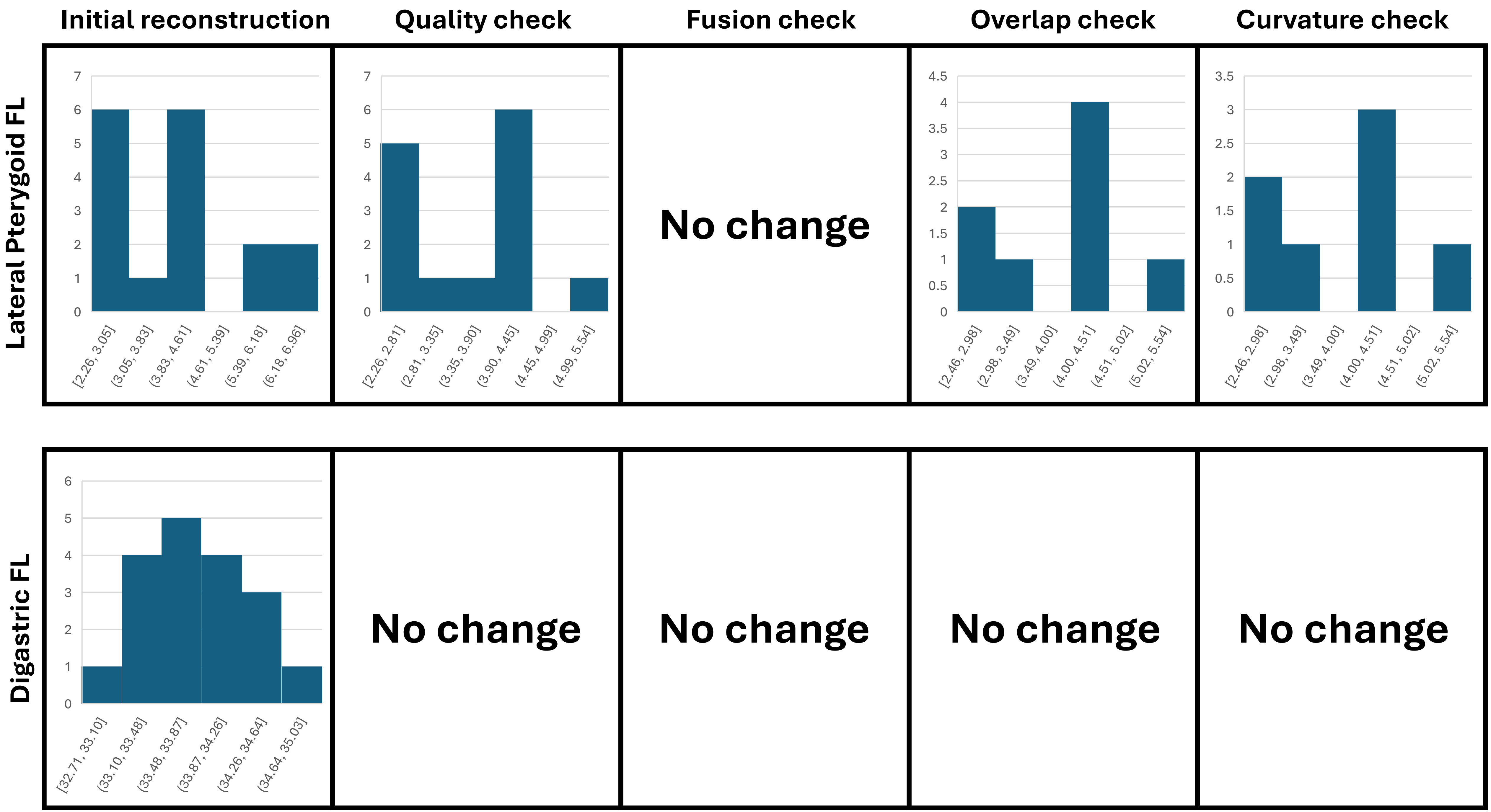

Initial reconstruction
Quality check
Fusion check
Overlap check
Curvature check
Lateral Pterygoid FL
Digastric FL
No change
No change
No change
No change
No change
[2.26, 3.05]
(3.05, 3.83]
(3.83, 4.61]
(4.61, 5.39]
(5.39, 6.18]
(6.18, 6.96]
[2.26, 2.81]
(2.81, 3.35]
(3.35, 3.90]
(3.90, 4.45]
(4.45, 4.99]
(4.99, 5.54]
[2.46, 2.98]
(2.98, 3.49]
(3.49, 4.00]
(4.00, 4.51]
(4.51, 5.02]
(5.02, 5.54]
[32.71, 33.10]
(33.10, 33.48]
(33.48, 33.87]
(33.87, 34.26]
(34.26, 34.64]
(34.64, 35.03]
