# Supplementary material for: Multi‐method analysis for the three‐dimensional reconstruction of muscle fascicles from DiceCT datasets
Source: Anat Rec (Hoboken). 2025 Dec 4;309(12):3491–510. doi: 10.1002/ar.70088 (PMC13749328; doi:10.1002/ar.70088)
Supplement: Supplementary file 4 [file AR-309-3491-s007.pdf]

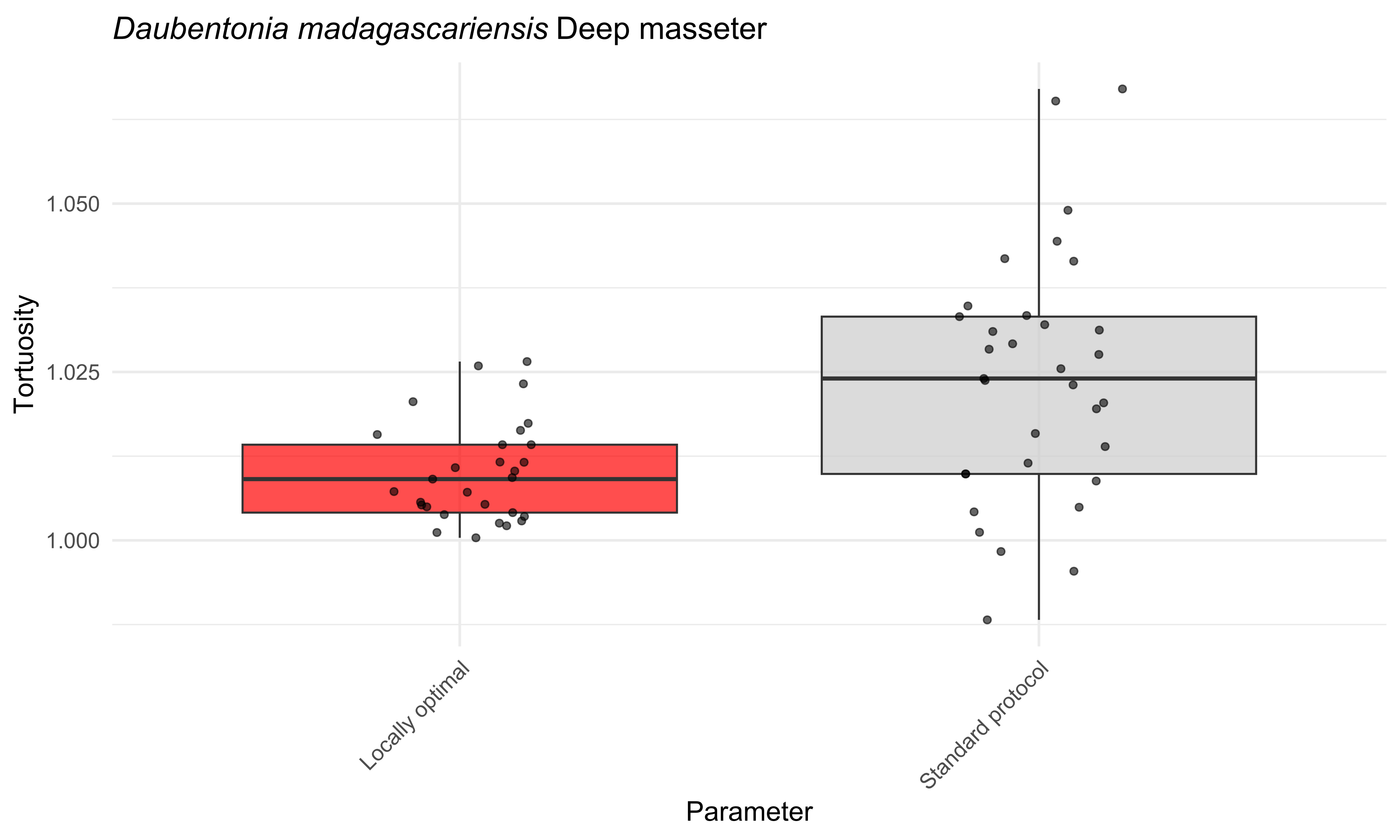

Daubentonia madagascariensis Deep masseter
Tortuosity
1.050
1.025
1.000
Locally optimal
Standard protocol
Parameter

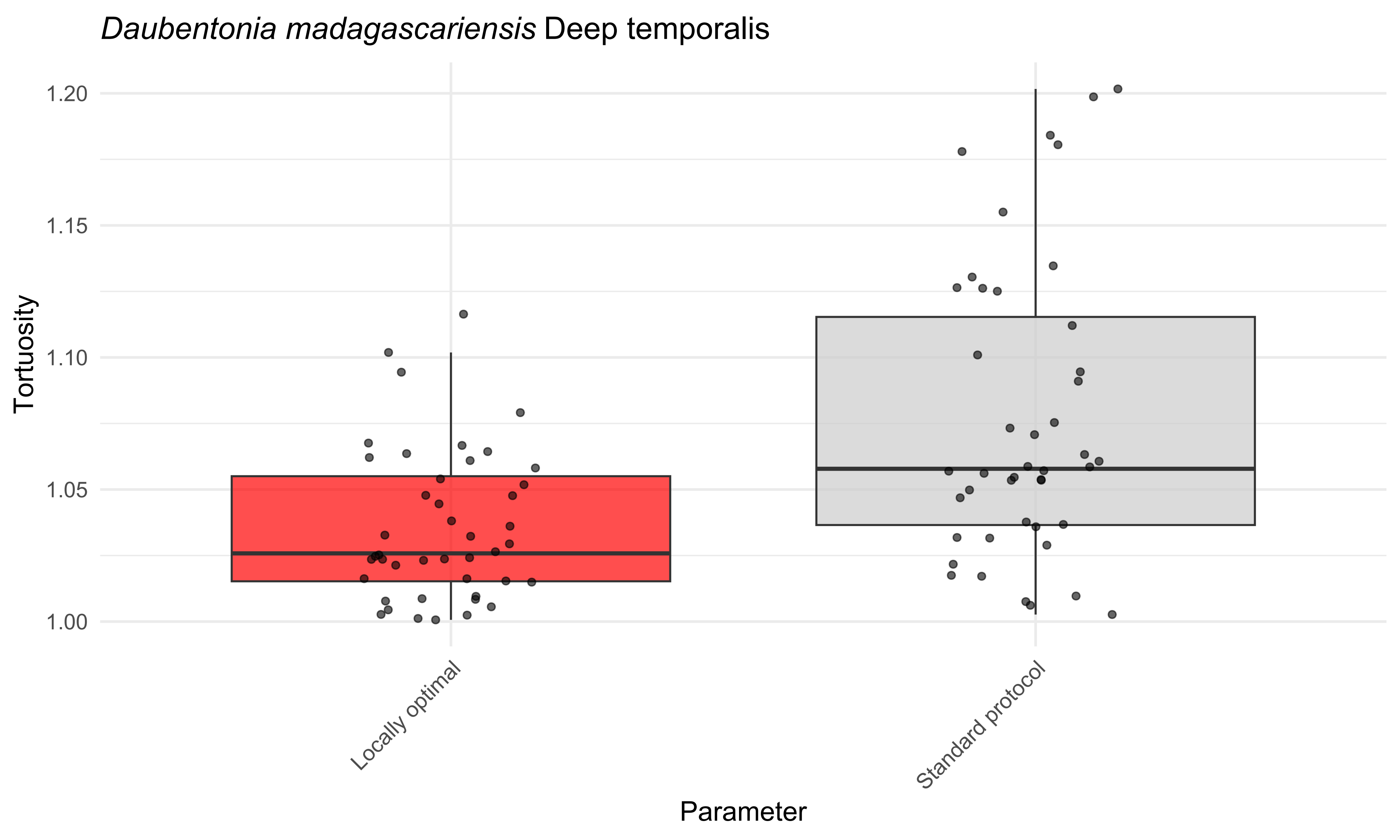

Daubentonia madagascariensis Deep temporalis
Tortuosity
1.20
1.15
1.10
1.05
1.00
Locally optimal
Standard protocol
Parameter

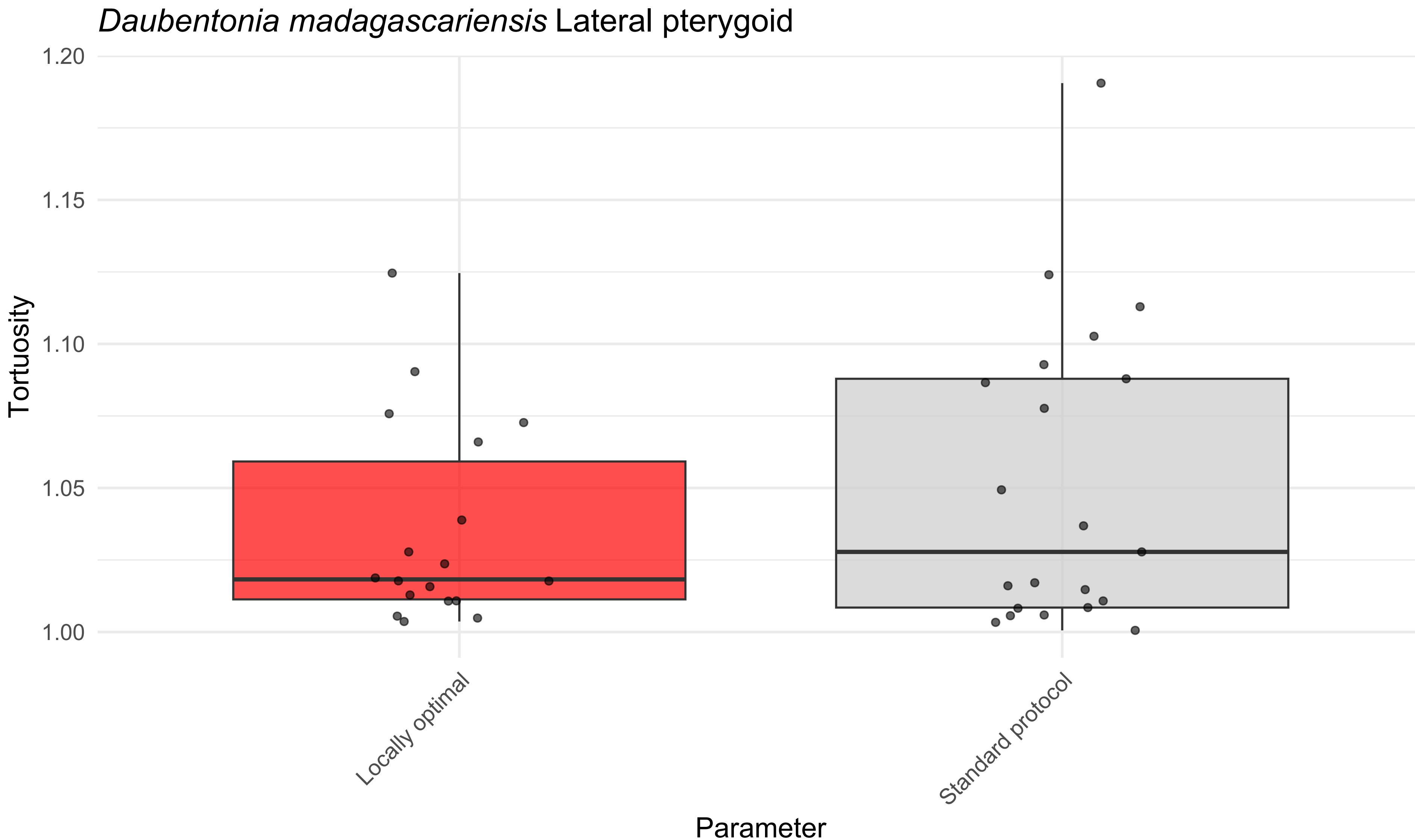

Daubentonia madagascariensis Lateral pterygoid
1.20
1.15
1.10
1.05
1.00
Tortuosity
Locally optimal
Standard protocol
Parameter

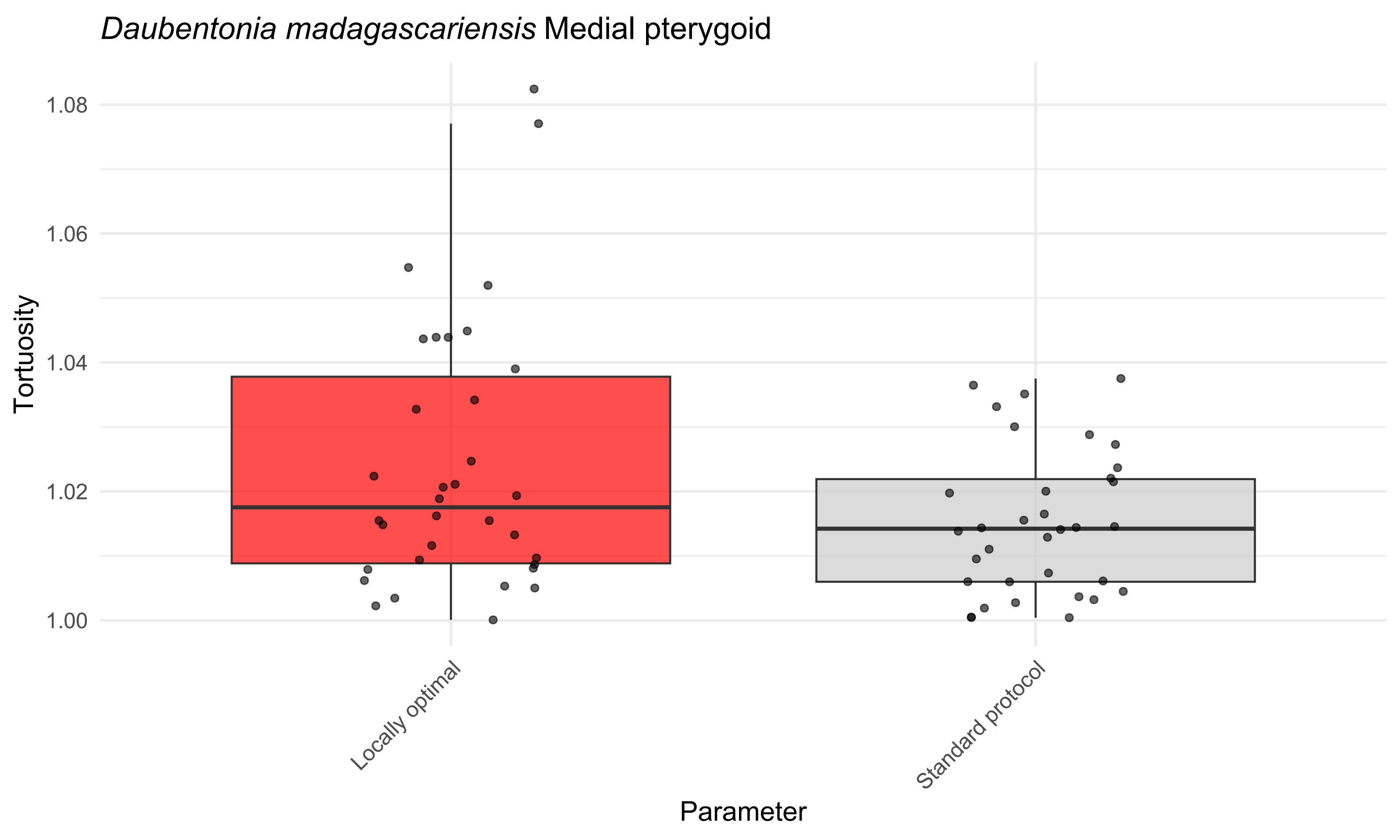
Daubentonia madagascariensis Medial pterygoid
Tortuosity
1.08
1.06
1.04
1.02
1.00
Locally optimal
Standard protocol
Parameter

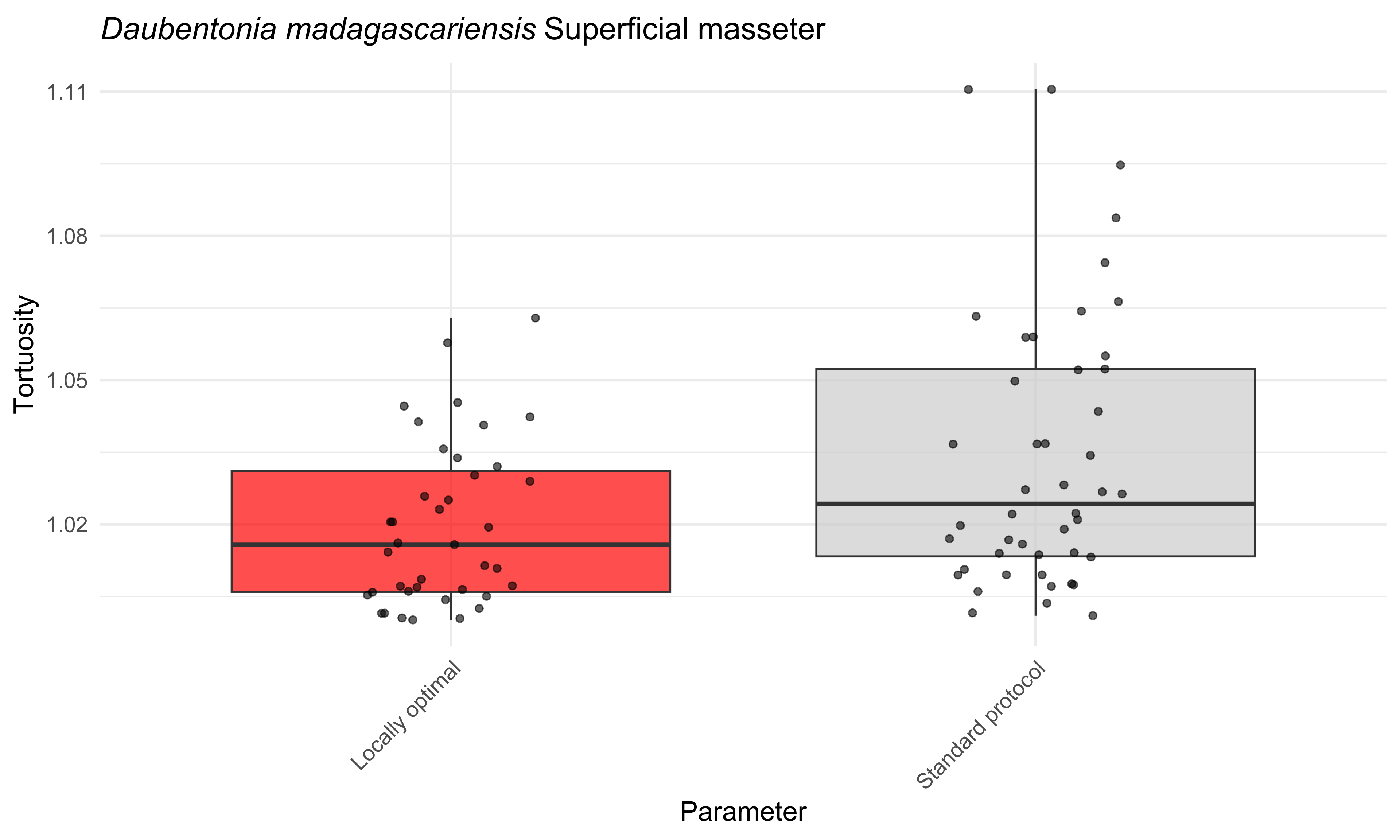

Daubentonia madagascariensis Superficial masseter
Tortuosity
1.11
1.08
1.05
1.02
Locally optimal
Standard protocol
Parameter

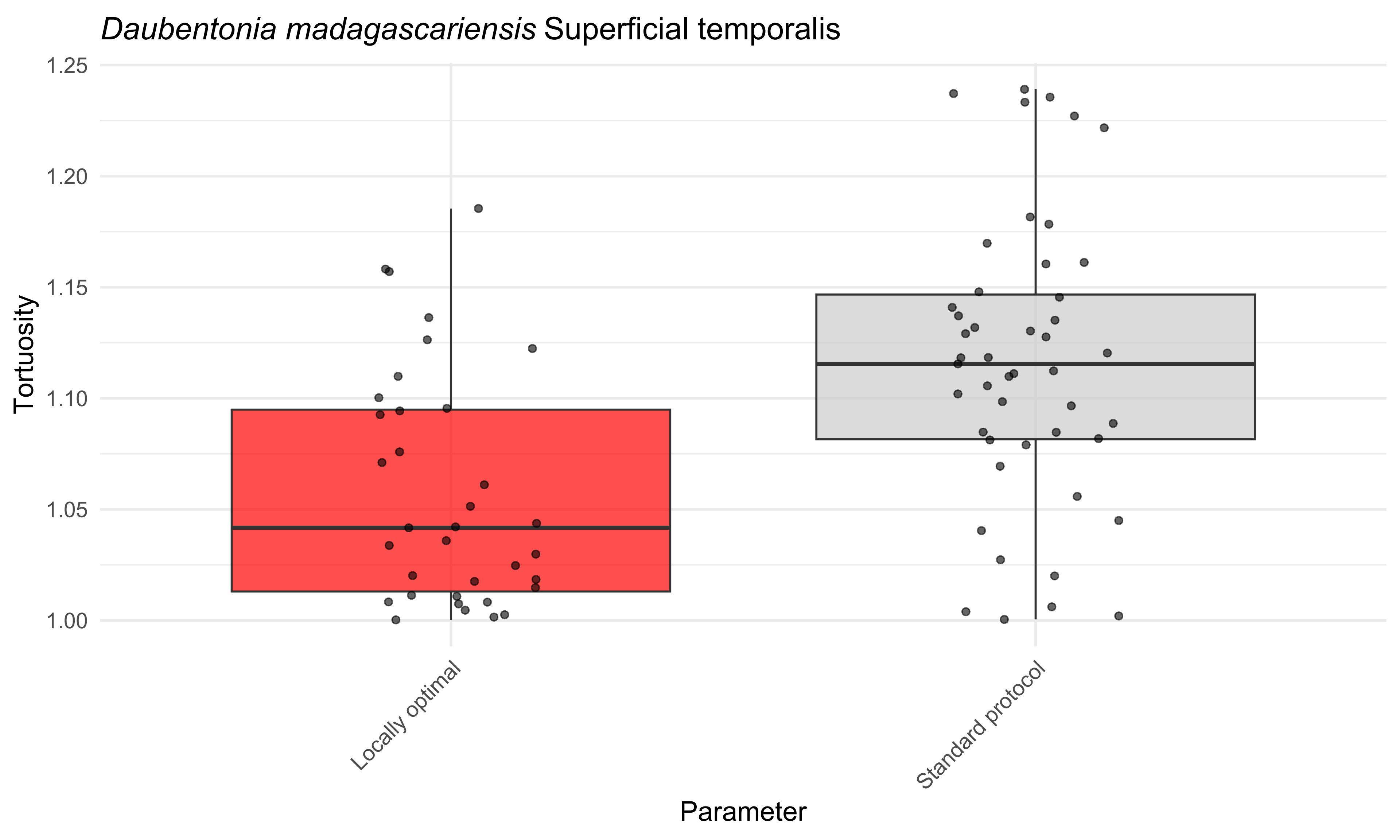

Daubentonia madagascariensis Superficial temporalis
1.25
1.20
1.15
1.10
1.05
1.00
Tortuosity
Locally optimal
Standard protocol
Parameter

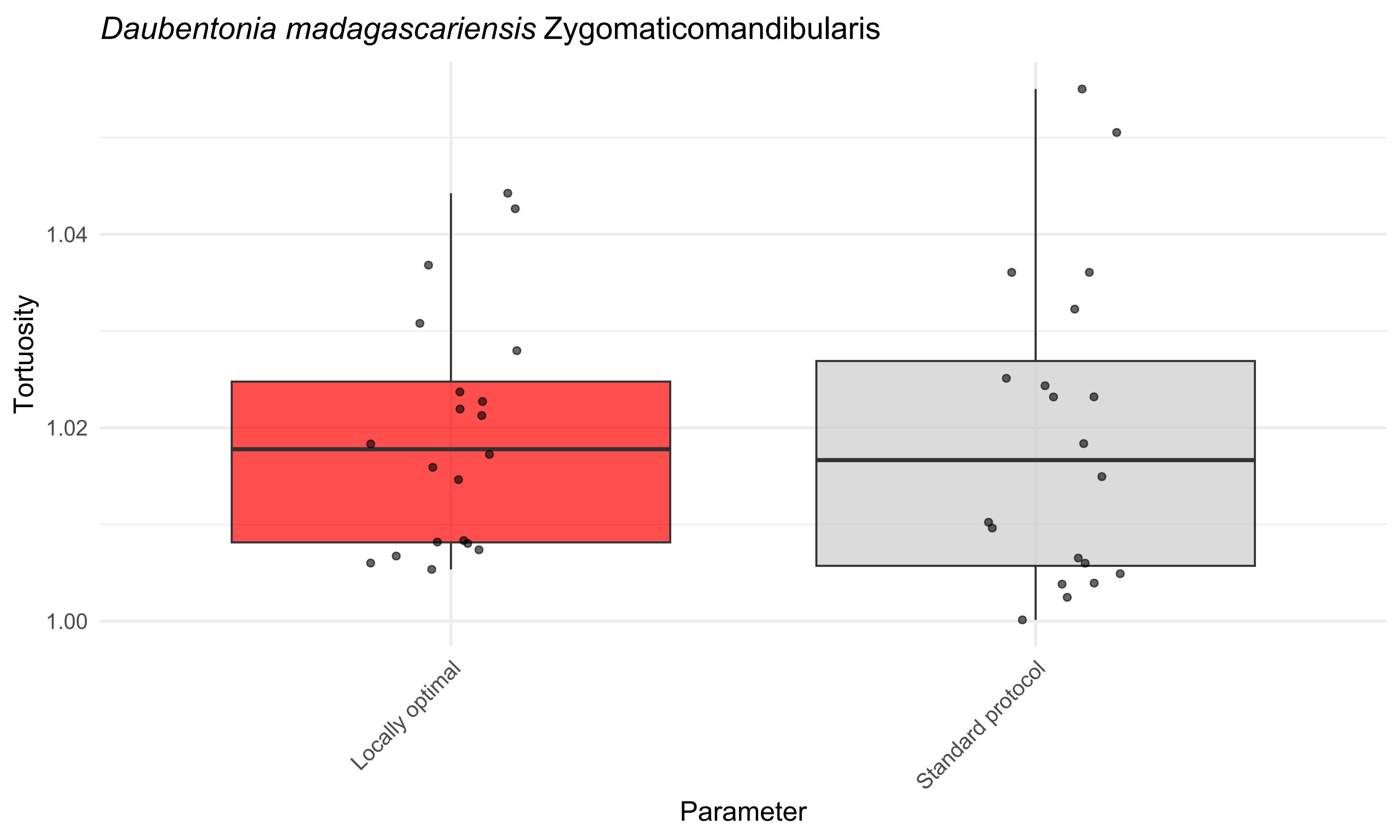
Daubentonia madagascariensis Zygomaticomandibularis
Tortuosity
1.04
1.02
1.00
Locally optimal
Standard protocol
Parameter

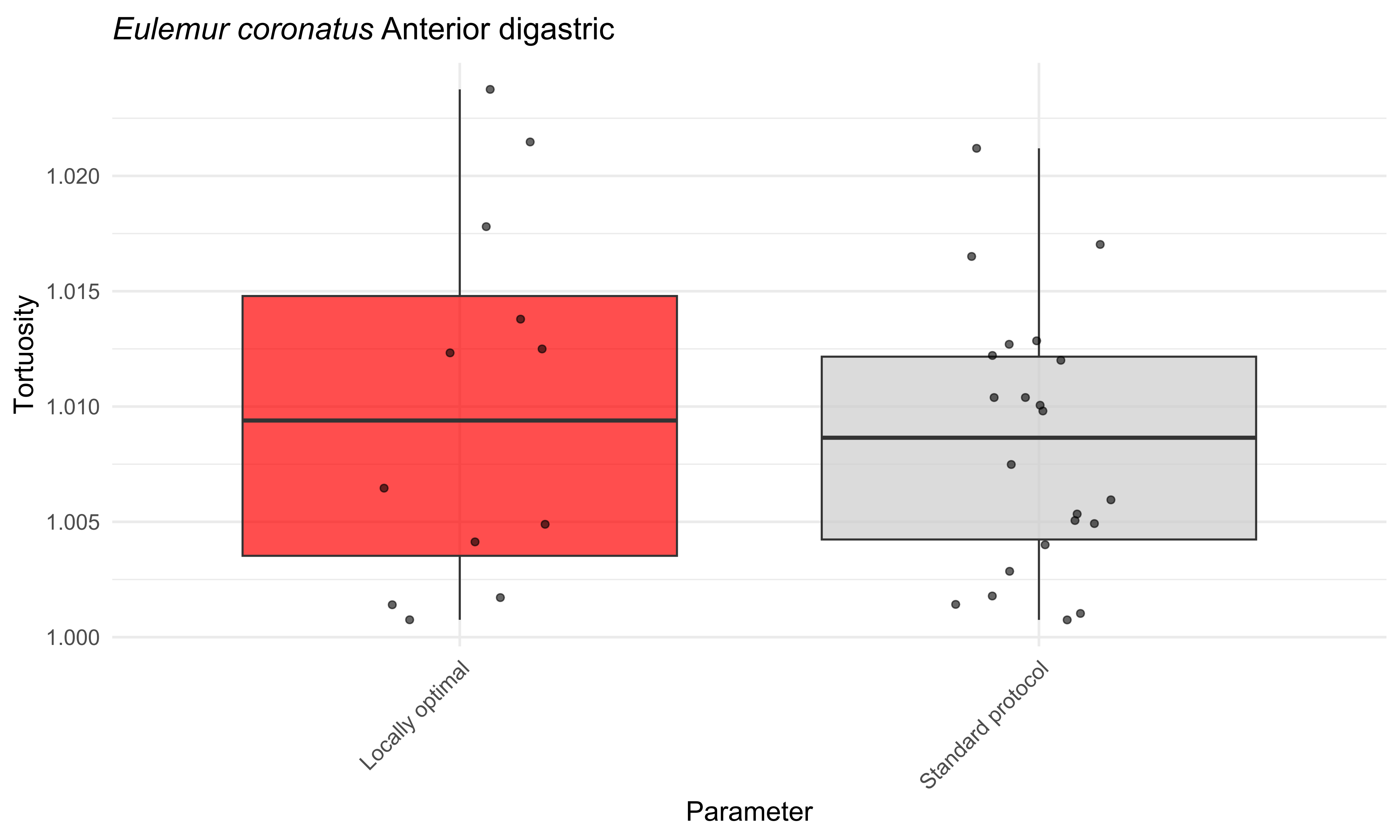

Eulemur coronatus Anterior digastric
Tortuosity
1.020
1.015
1.010
1.005
1.000
Locally optimal
Standard protocol
Parameter

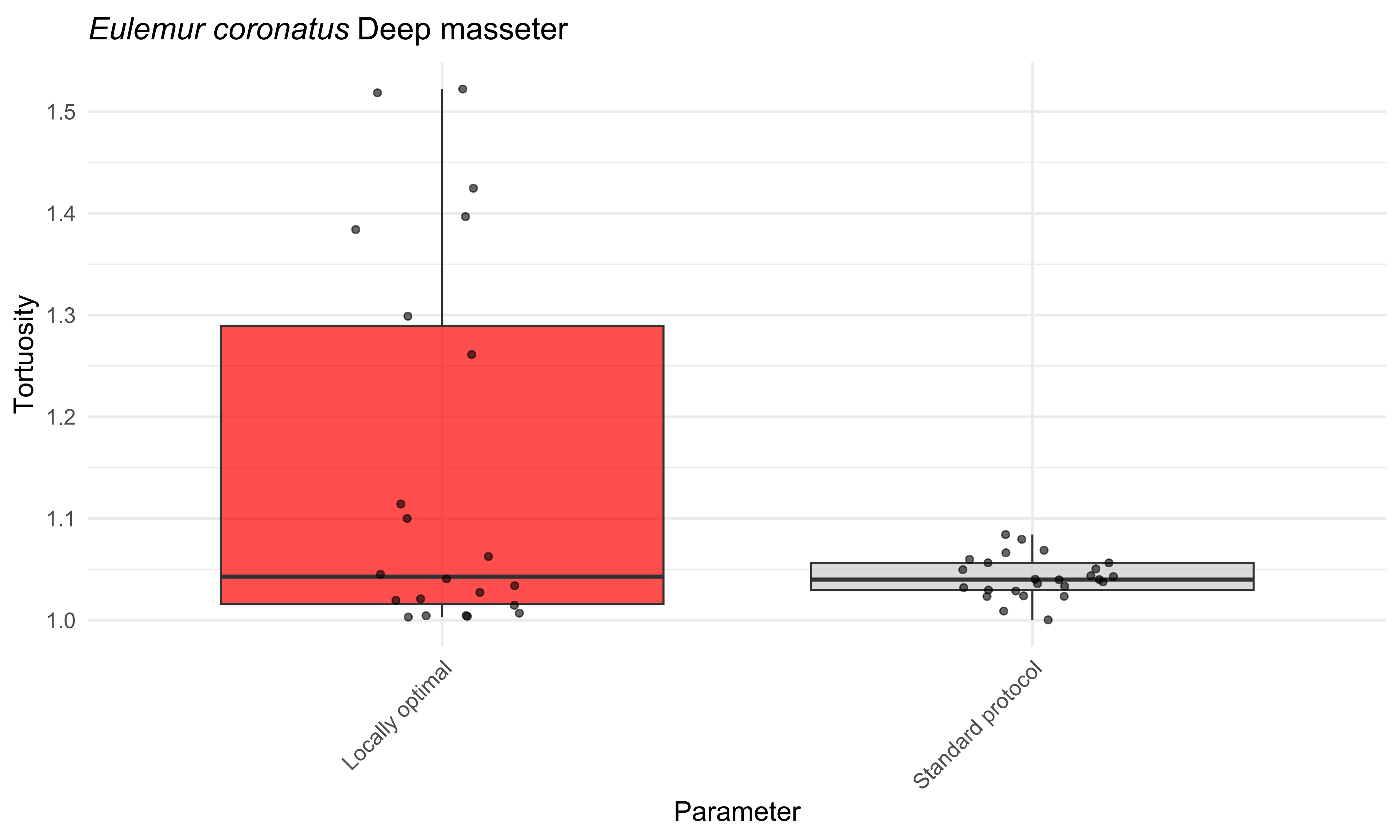

Eulemur coronatus Deep masseter
Tortuosity
1.5
1.4
1.3
1.2
1.1
1.0
Locally optimal
Standard protocol
Parameter

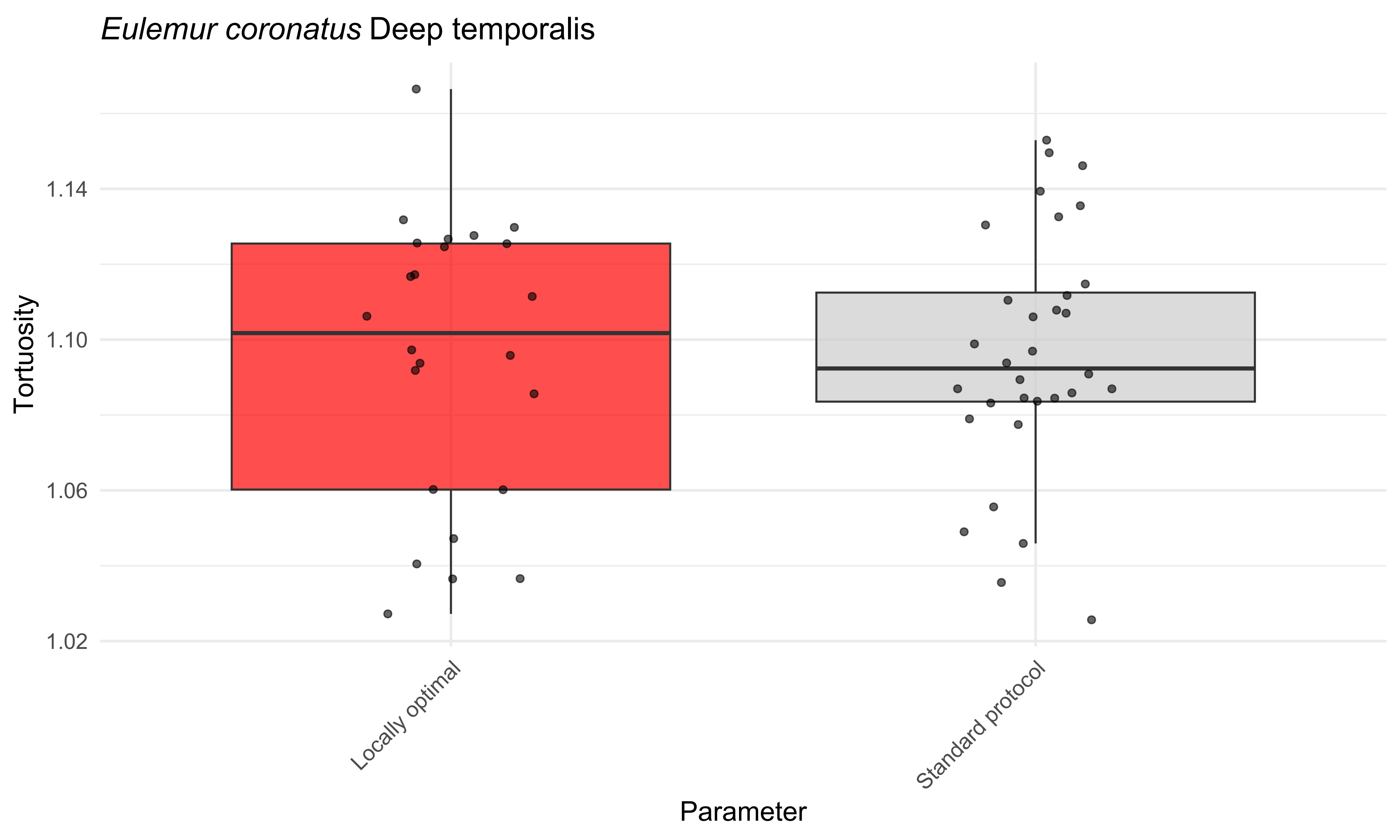

Eulemur coronatus Deep temporalis
Tortuosity
1.14
1.10
1.06
1.02
Locally optimal
Standard protocol
Parameter

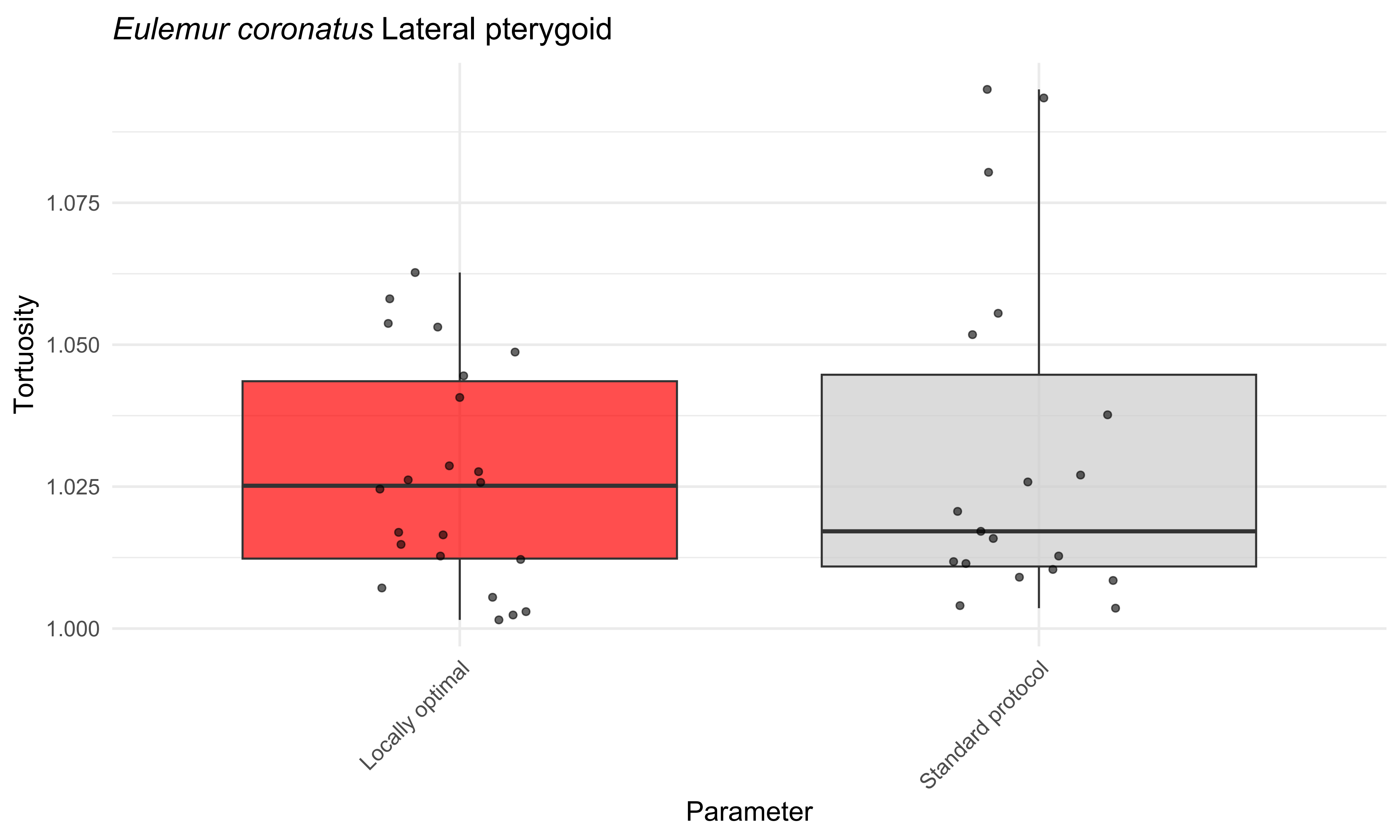

Eulemur coronatus Lateral pterygoid
Tortuosity
1.075
1.050
1.025
1.000
Locally optimal
Standard protocol
Parameter

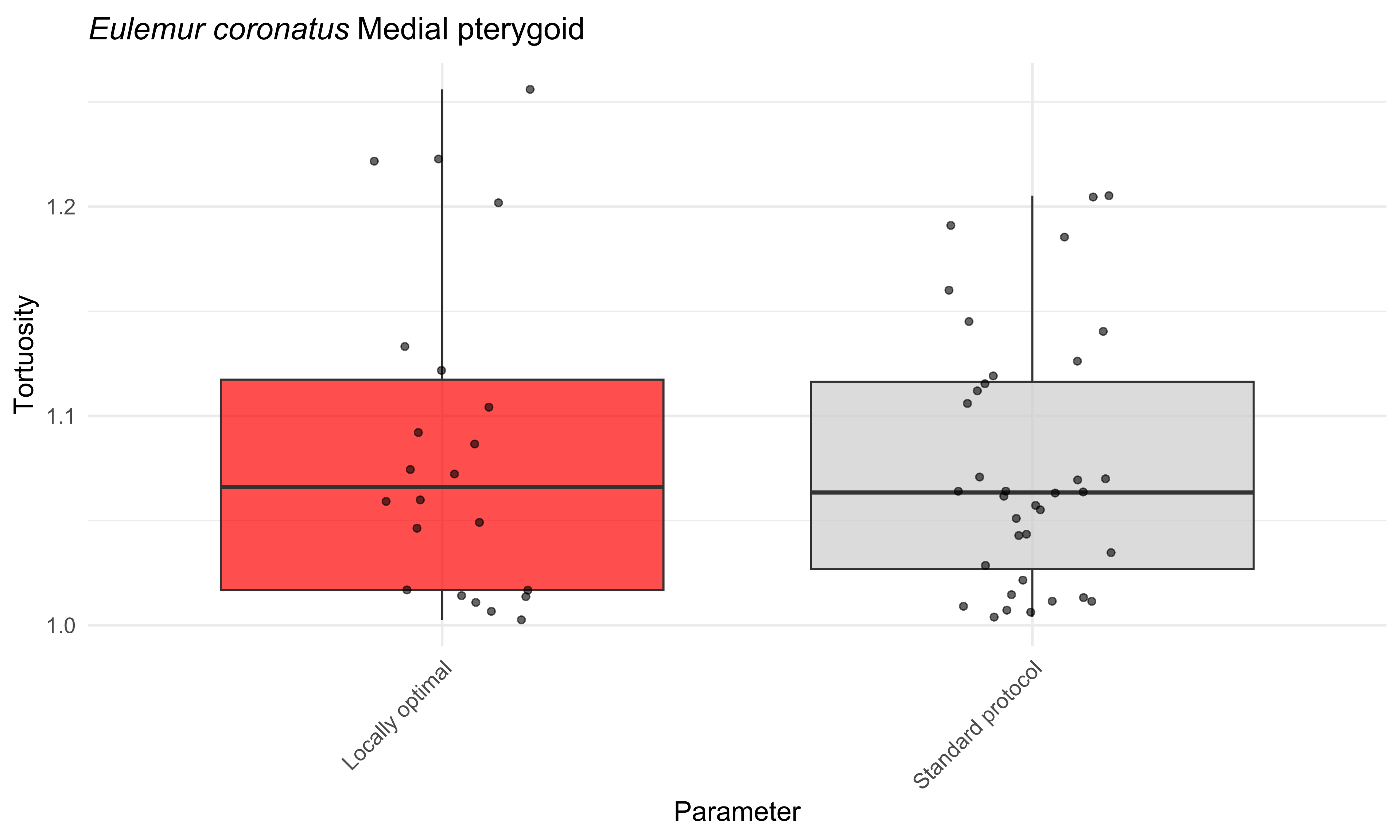

Eulemur coronatus Medial pterygoid
Tortuosity
1.2
1.1
1.0
Locally optimal
Standard protocol
Parameter

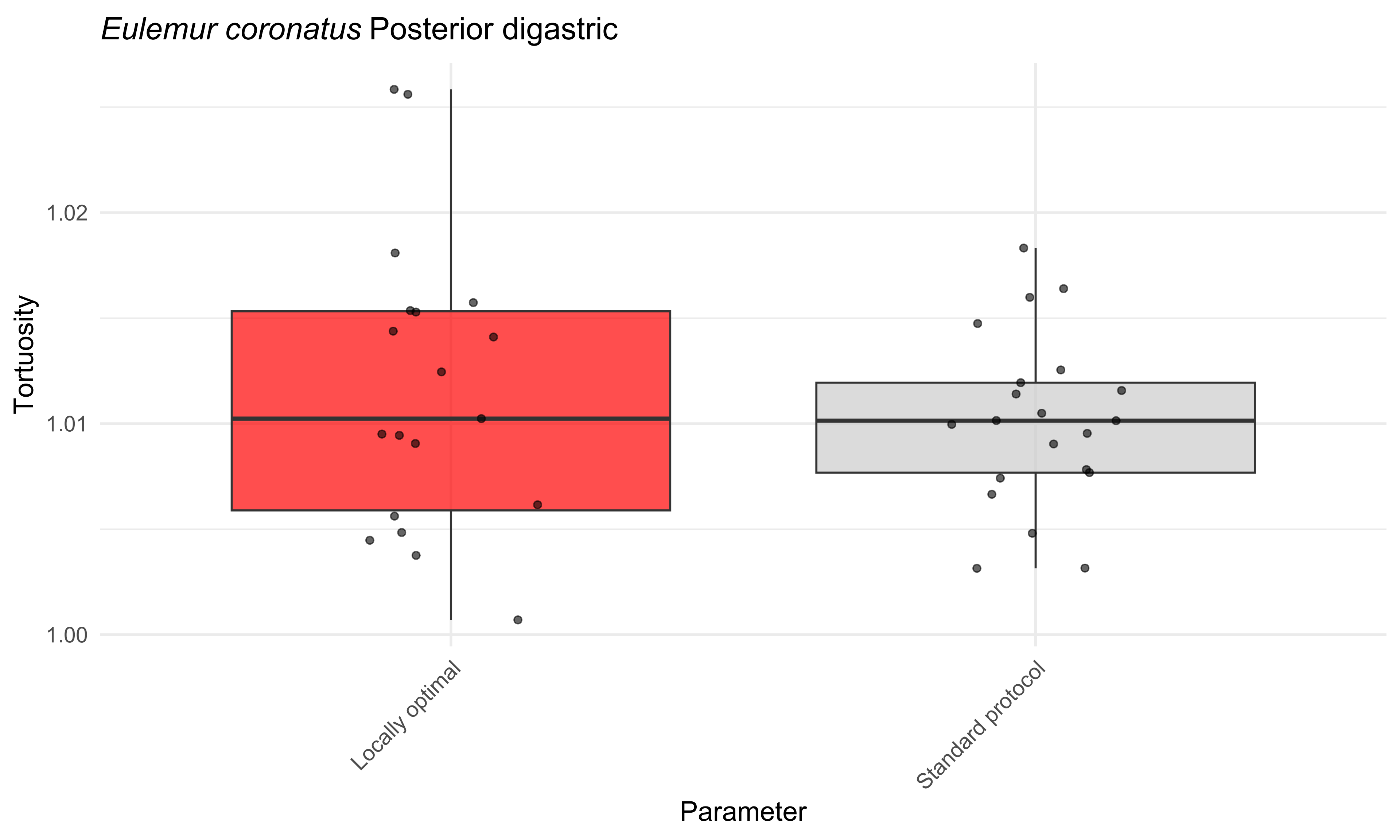

Eulemur coronatus Posterior digastric
Tortuosity
1.02
1.01
1.00
Locally optimal
Standard protocol
Parameter

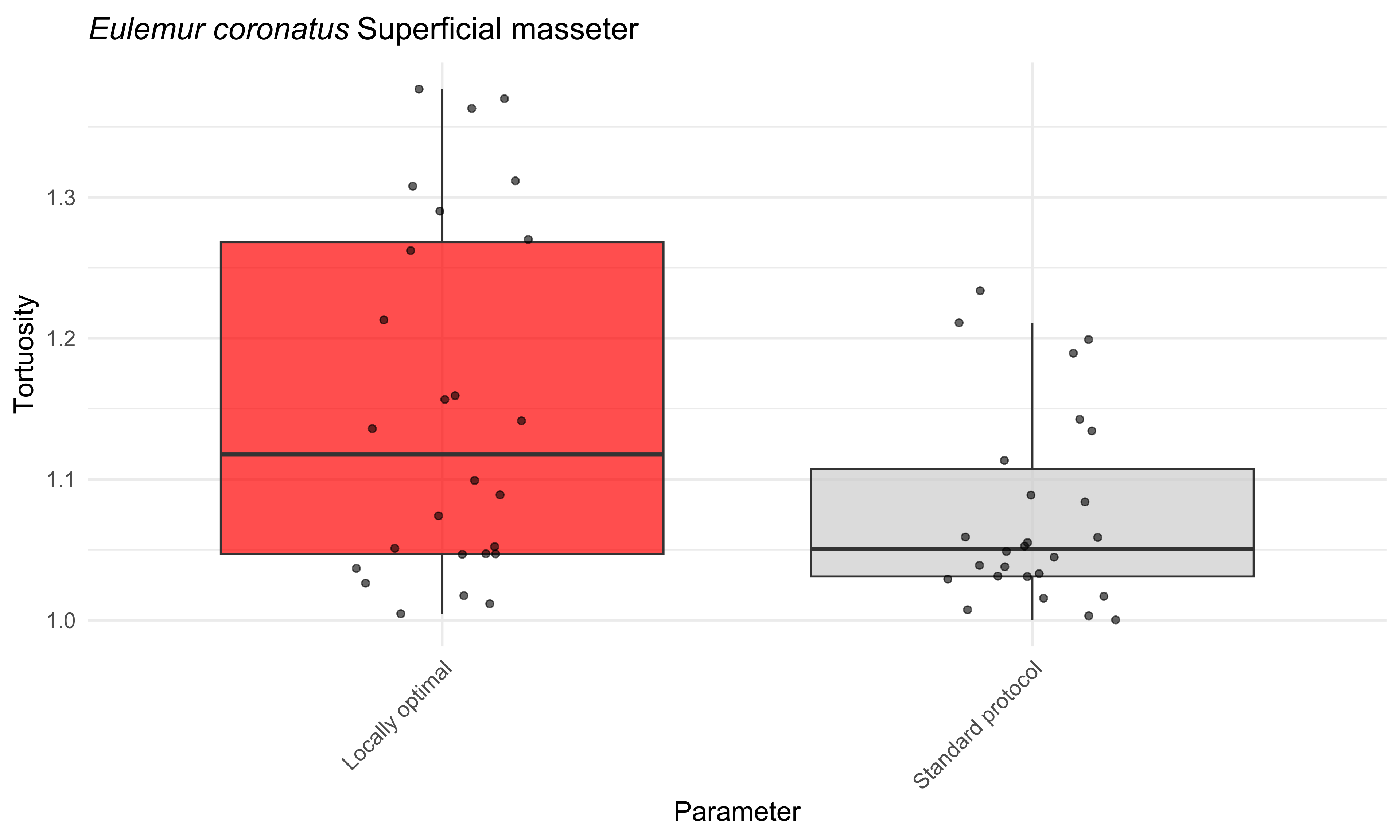
Eulemur coronatus Superficial masseter
Tortuosity
1.0
1.1
1.2
1.3
Locally optimal
Standard protocol
Parameter

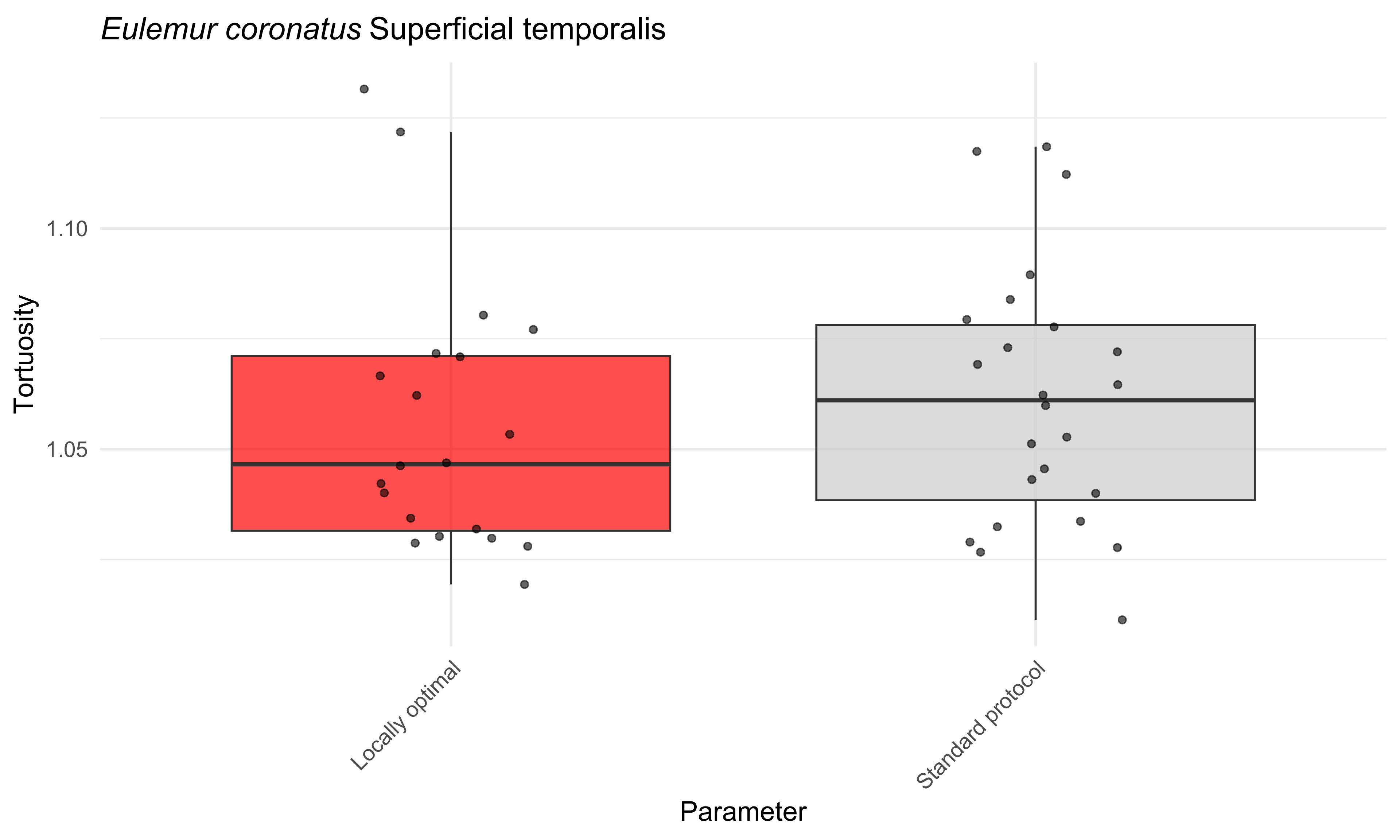
Eulemur coronatus Superficial temporalis
Tortuosity
1.10
1.05
Locally optimal
Standard protocol
Parameter

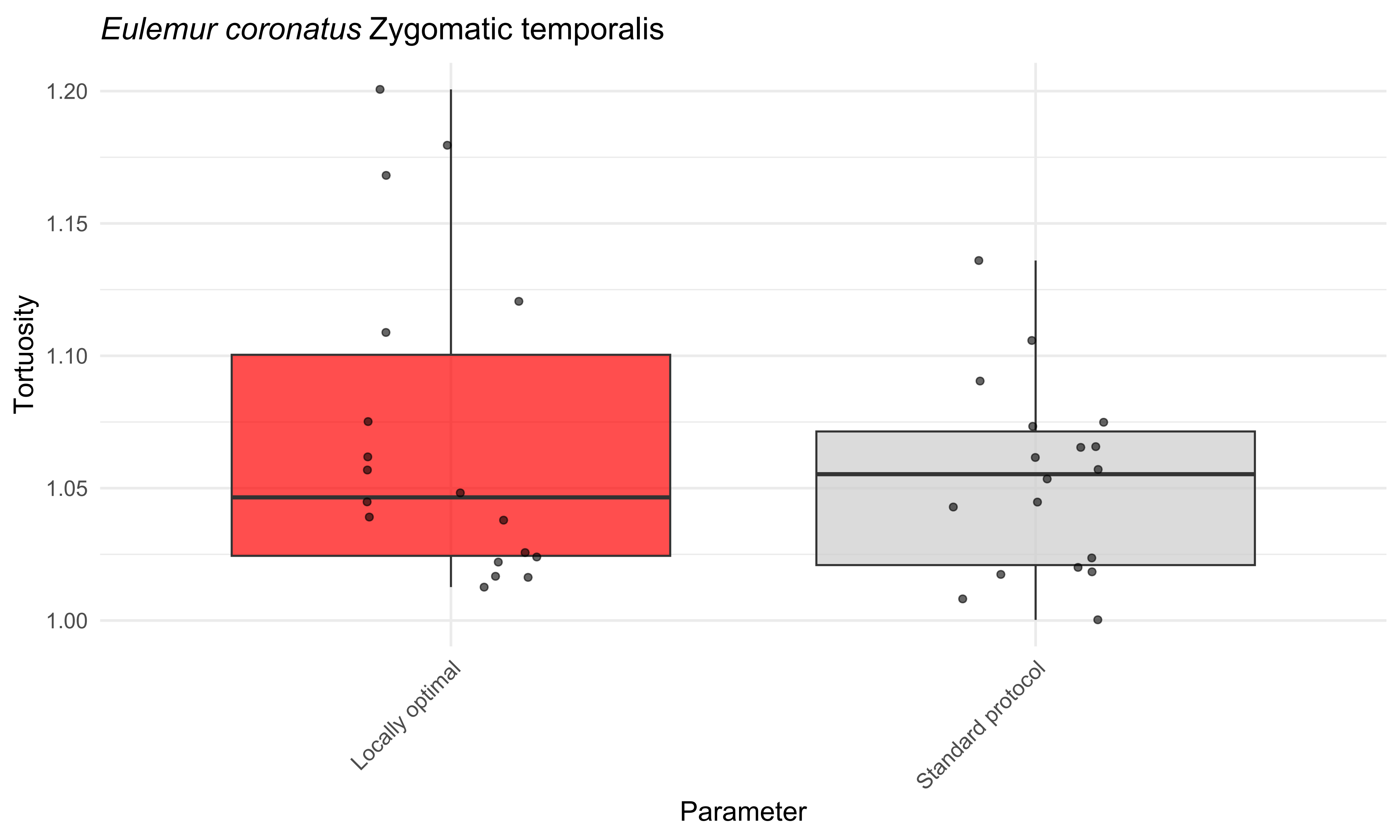

Eulemur coronatus Zygomatic temporalis
Tortuosity
1.20
1.15
1.10
1.05
1.00
Locally optimal
Standard protocol
Parameter

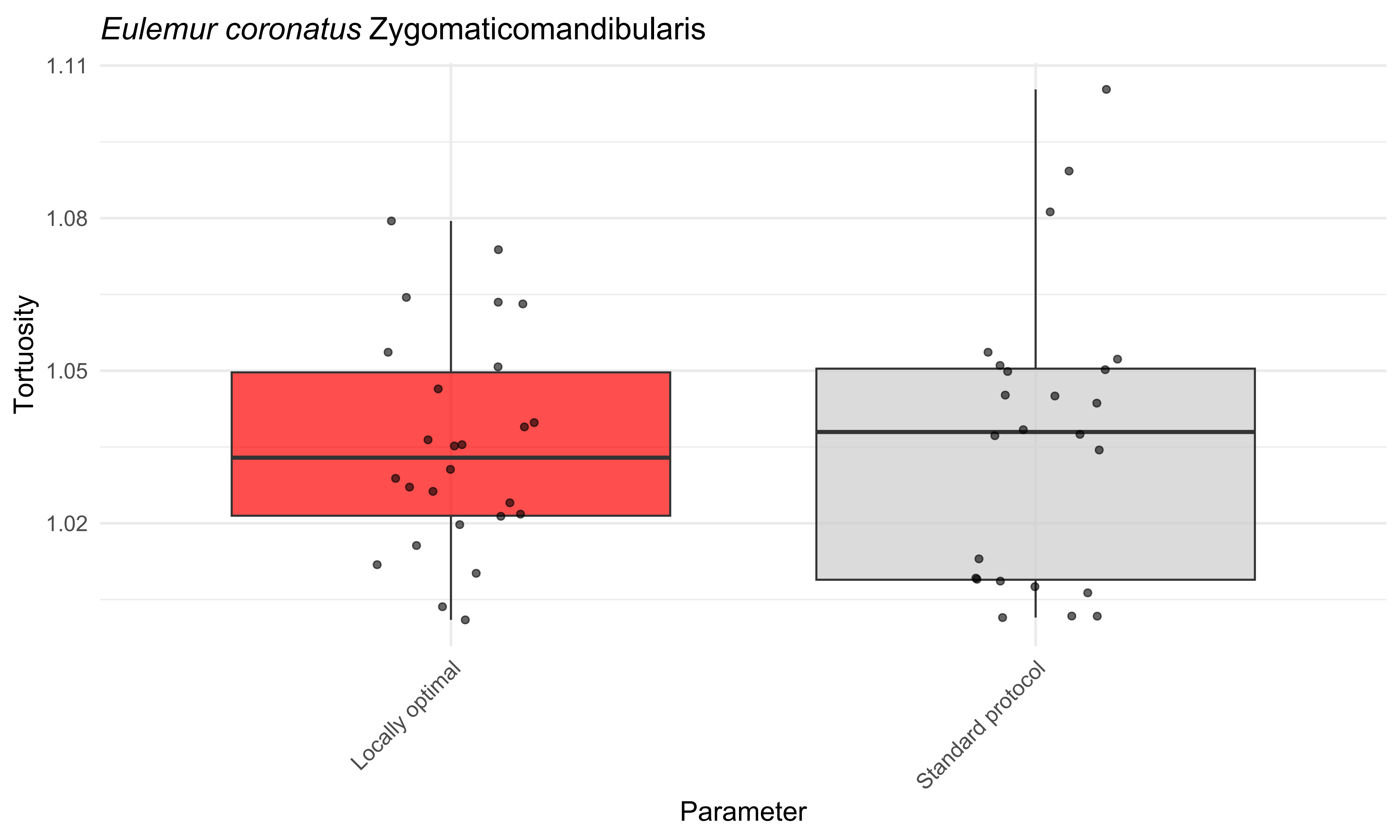

Eulemur coronatus Zygomaticomandibularis
1.11
1.08
1.05
1.02
Tortuosity
Locally optimal
Standard protocol
Parameter

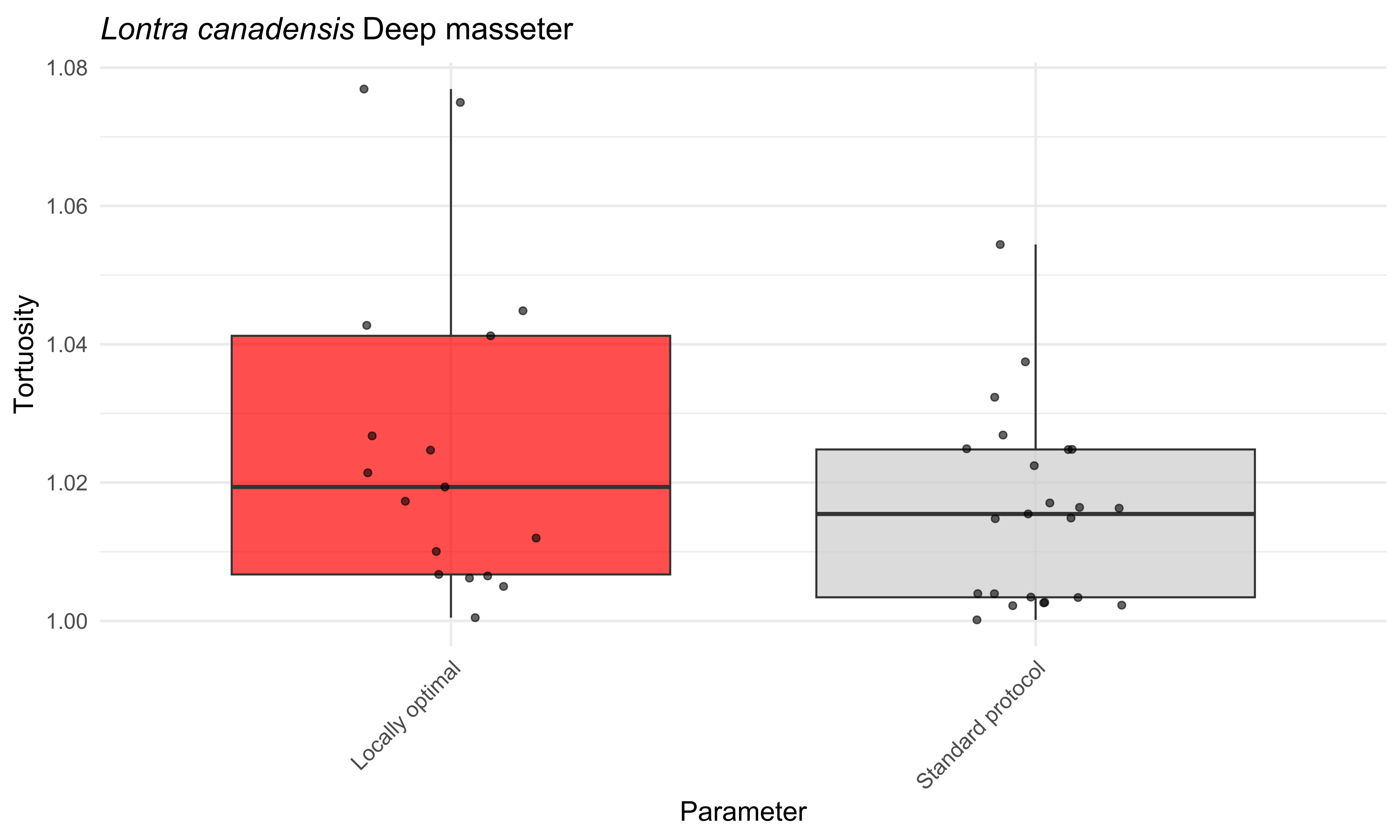

Lontra canadensis Deep masseter
Tortuosity
1.08
1.06
1.04
1.02
1.00
Locally optimal
Standard protocol
Parameter

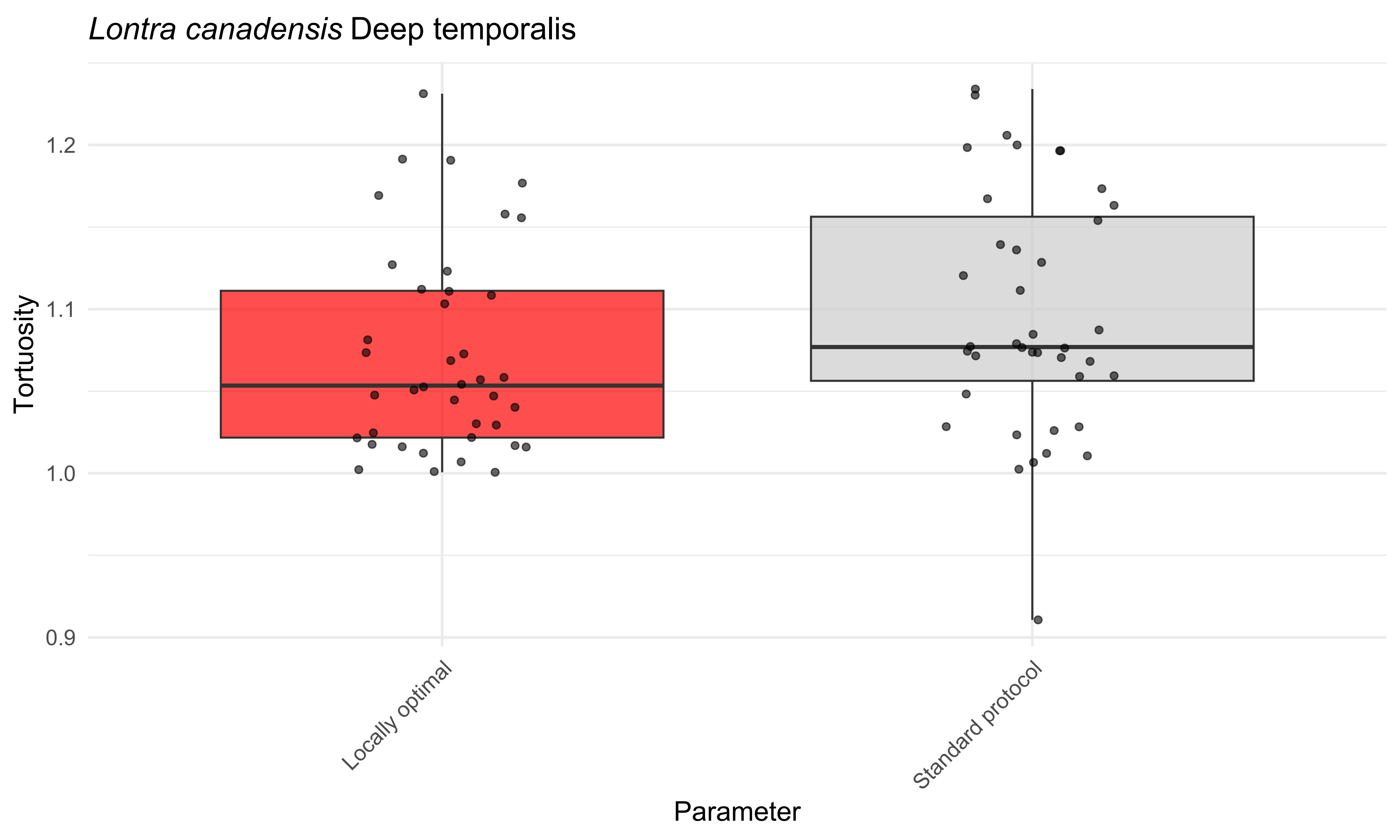

Lontra canadensis Deep temporalis
Tortuosity
1.2
1.1
1.0
0.9
Locally optimal
Standard protocol
Parameter

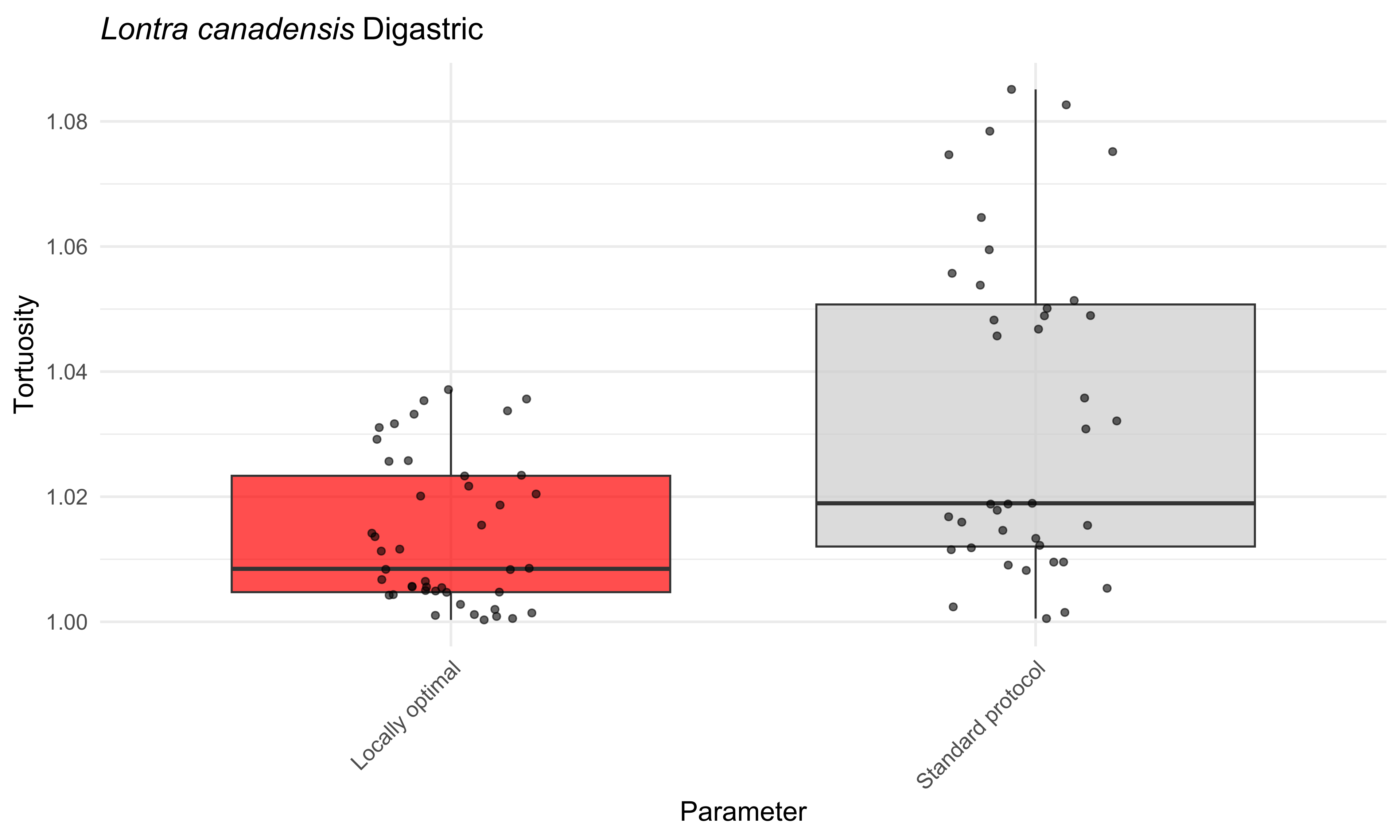

Lontra canadensis Digastric
Tortuosity
1.08
1.06
1.04
1.02
1.00
Locally optimal
Standard protocol
Parameter

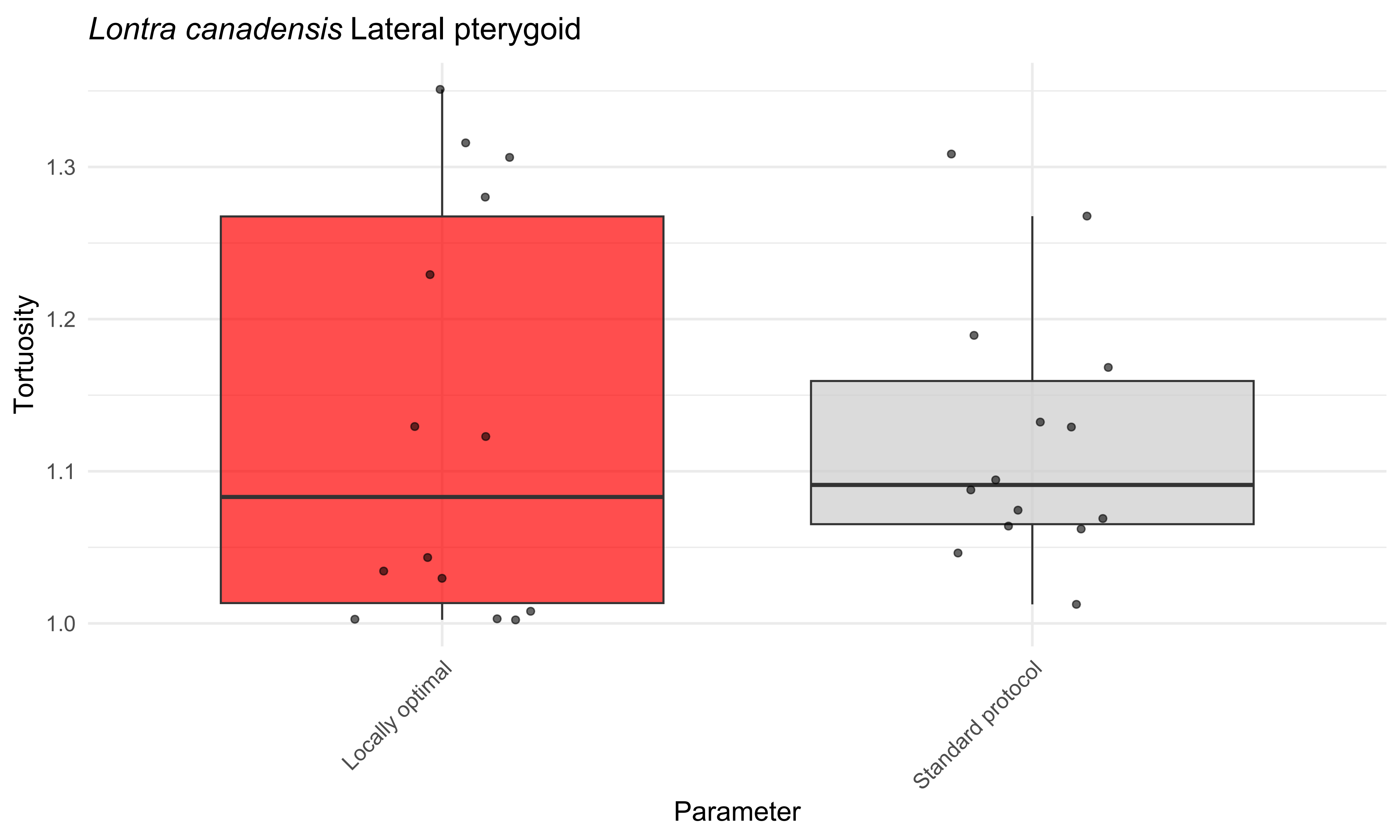

Lontra canadensis Lateral pterygoid
Tortuosity
1.3
1.2
1.1
1.0
Locally optimal
Standard protocol
Parameter

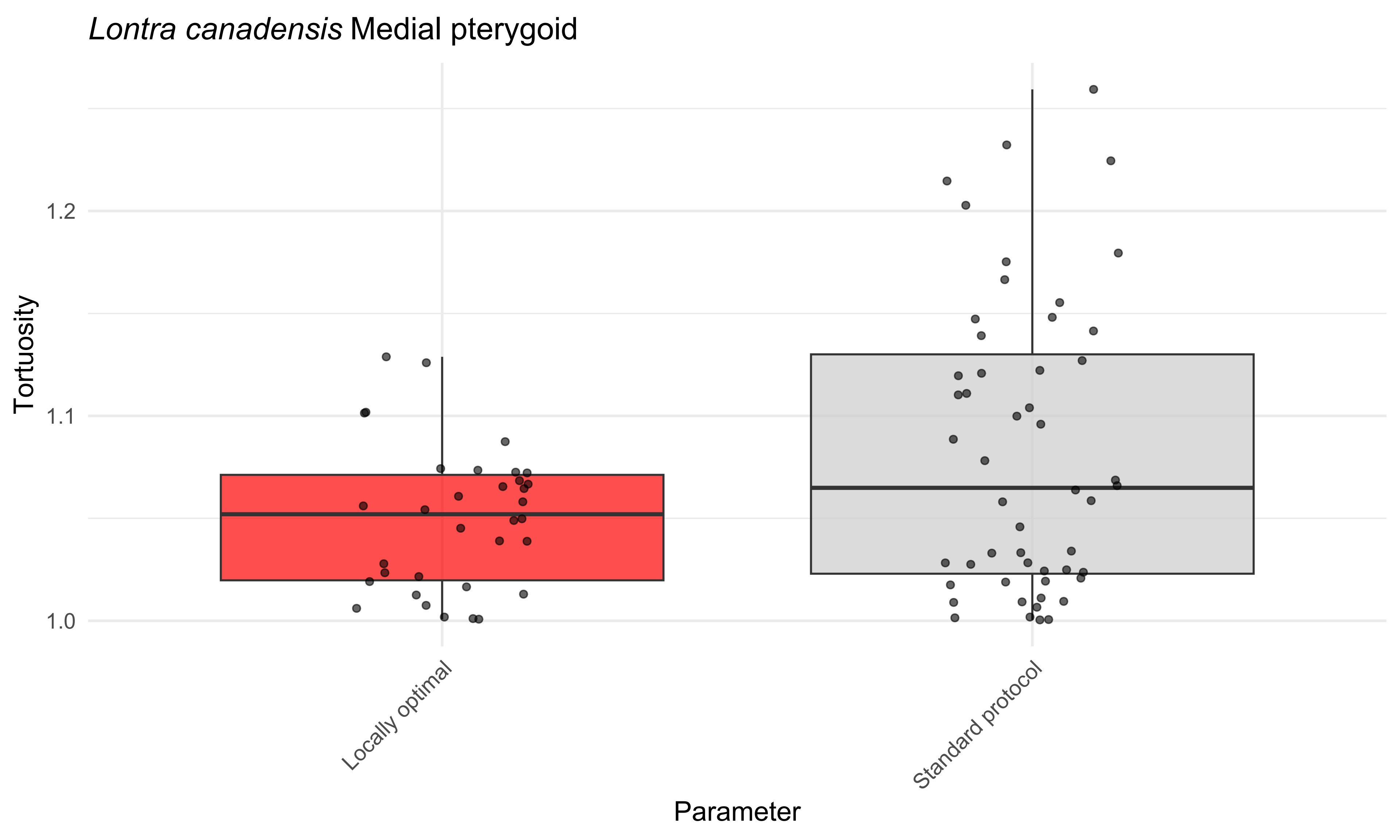

Lontra canadensis Medial pterygoid
Tortuosity
1.2
1.1
1.0
Locally optimal
Standard protocol
Parameter

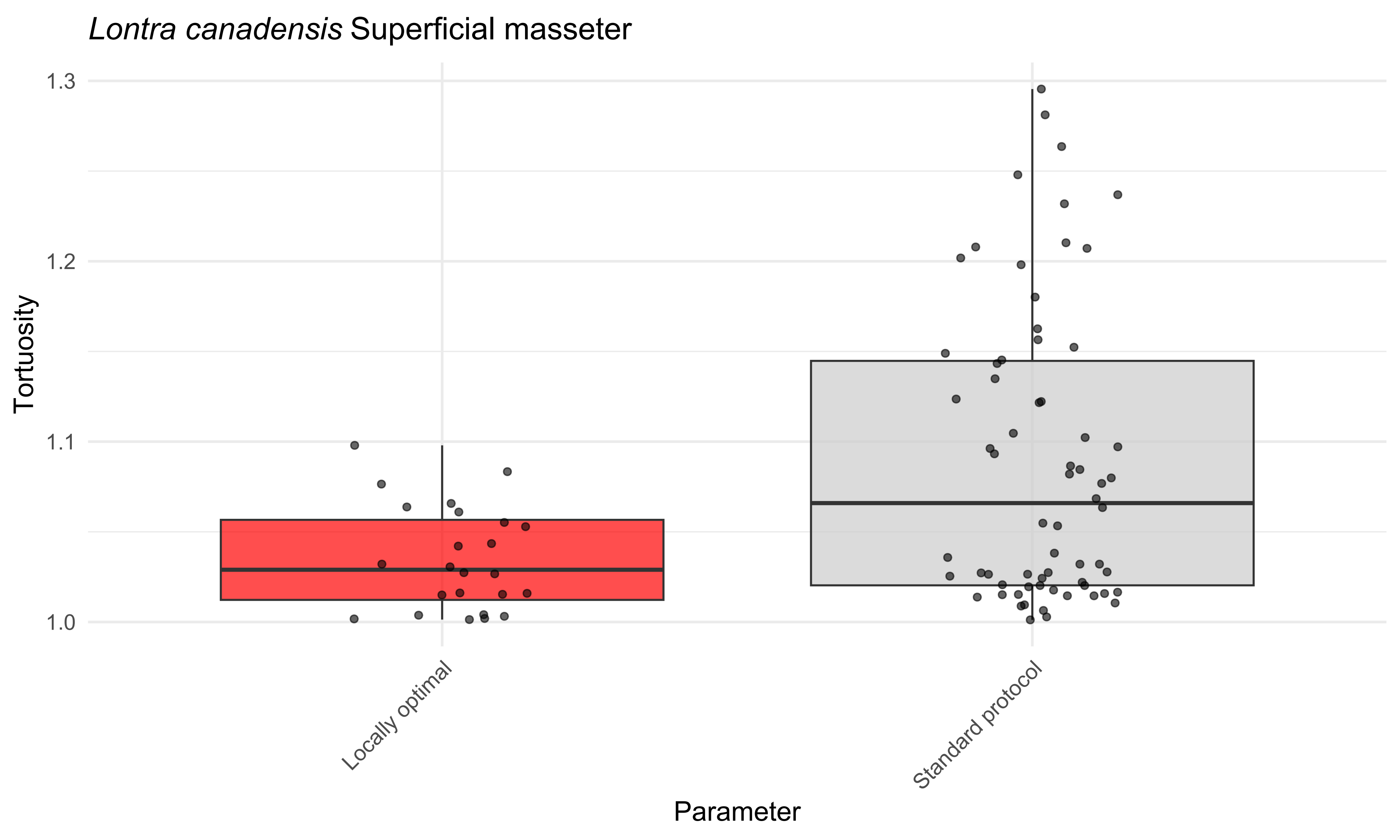

Lontra canadensis Superficial masseter
1.3
1.2
1.1
1.0
Tortuosity
Locally optimal
Standard protocol
Parameter

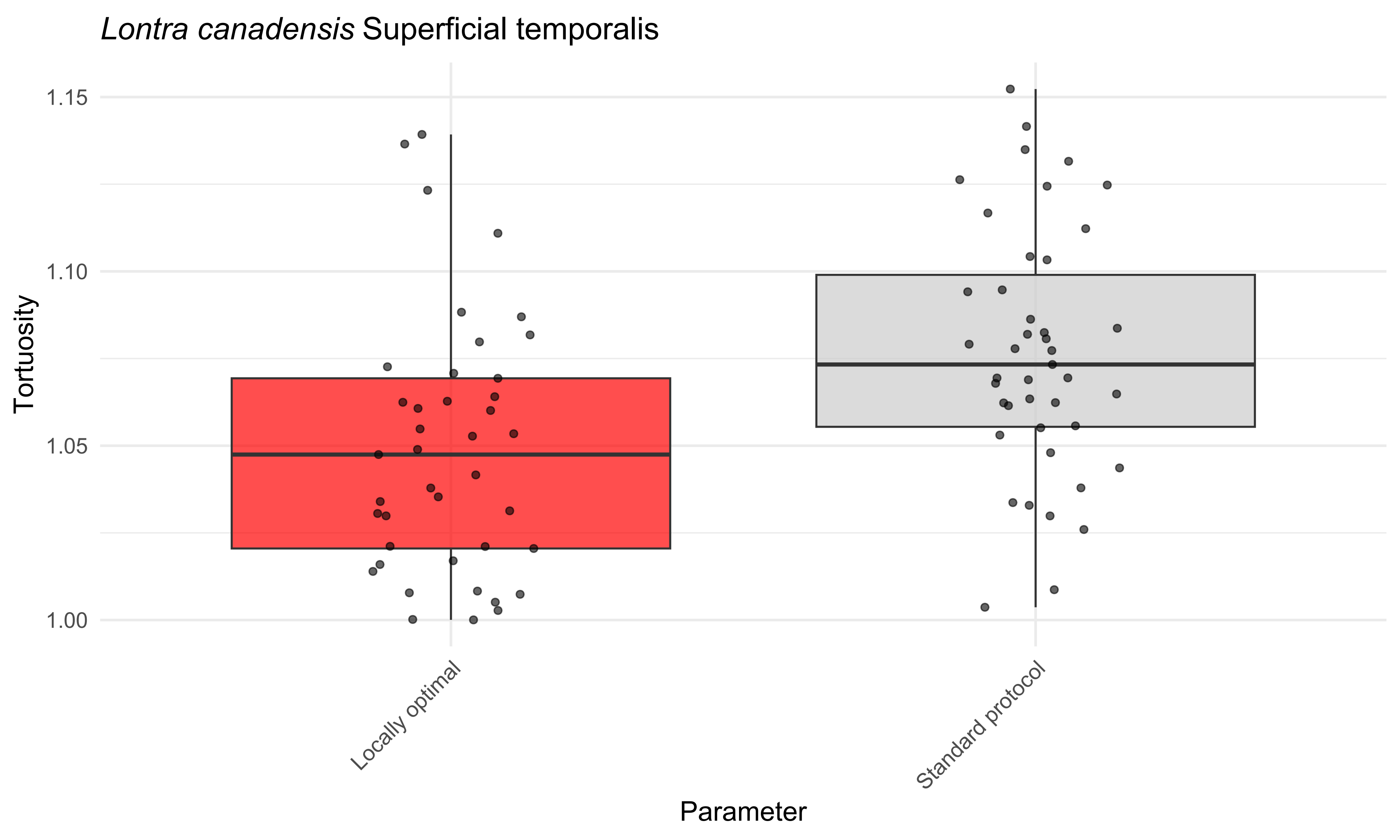

Lontra canadensis Superficial temporalis
Tortuosity
1.15
1.10
1.05
1.00
Locally optimal
Standard protocol
Parameter

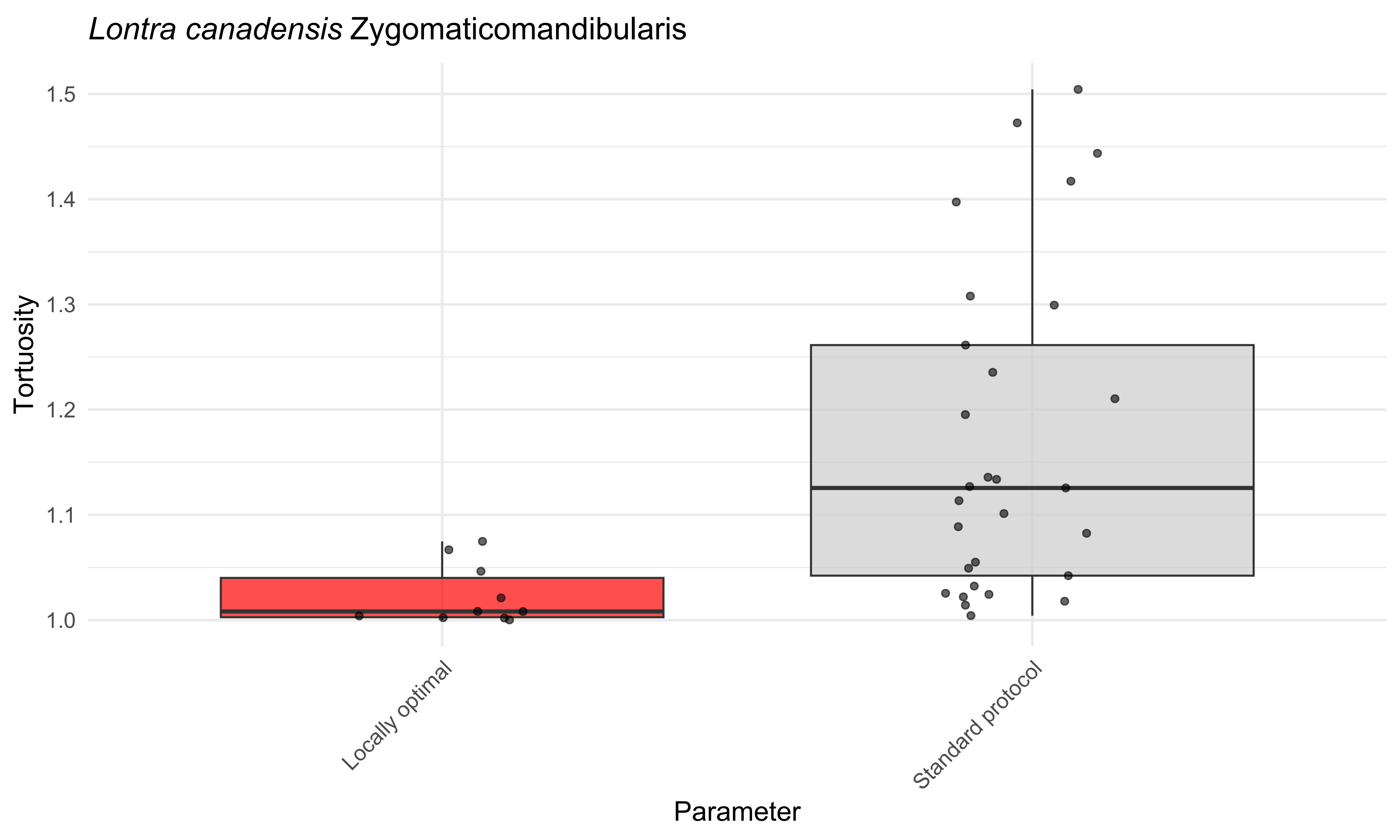

Lontra canadensis Zygomaticomandibularis
Tortuosity
1.5
1.4
1.3
1.2
1.1
1.0
Locally optimal
Standard protocol
Parameter

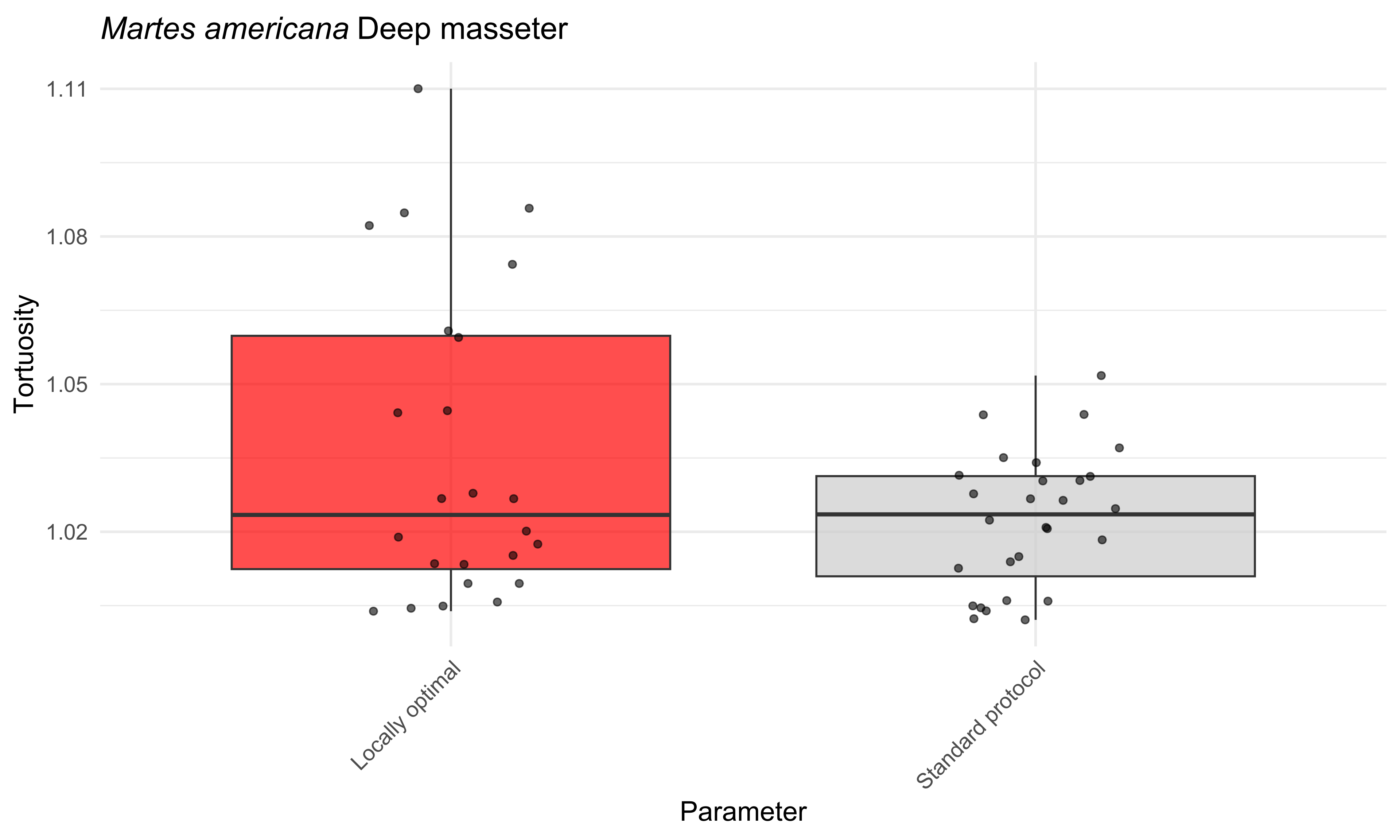

Martes americana Deep masseter
1.11
1.08
1.05
1.02
Tortuosity
Locally optimal
Standard protocol
Parameter

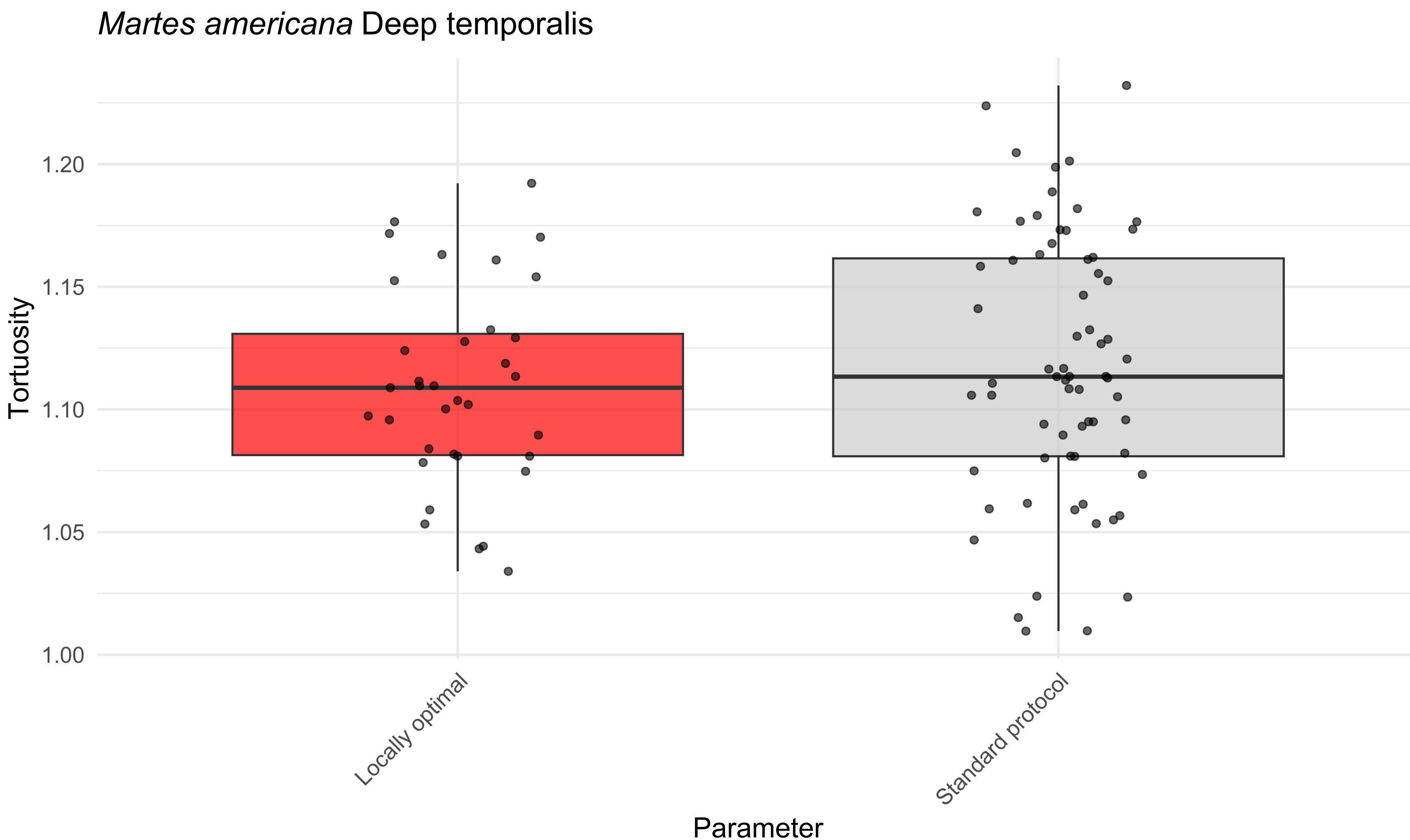

Martes americana Deep temporalis
Tortuosity
1.20
1.15
1.10
1.05
1.00
Locally optimal
Standard protocol
Parameter

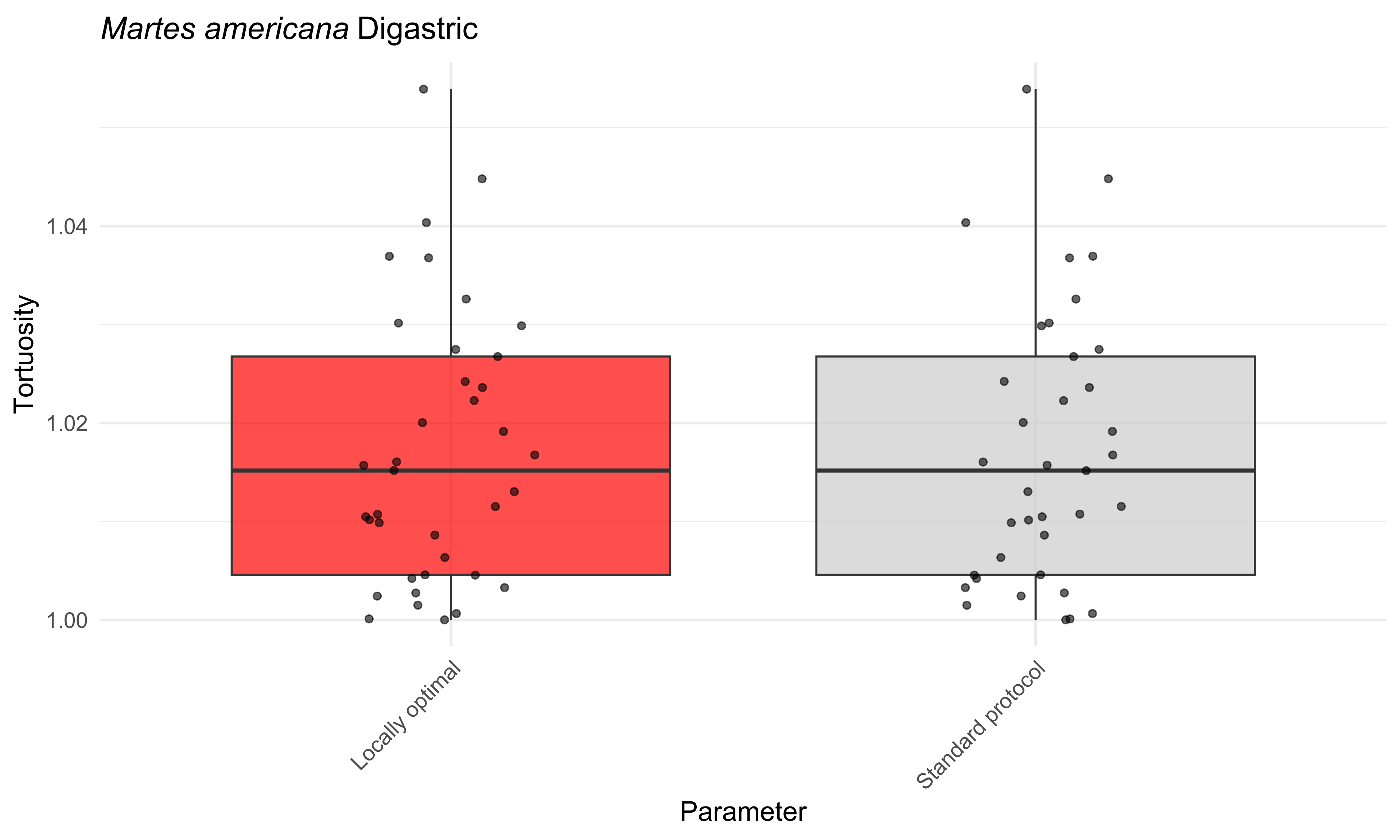

Martes americana Digastric
Tortuosity
1.04
1.02
1.00
Locally optimal
Standard protocol
Parameter

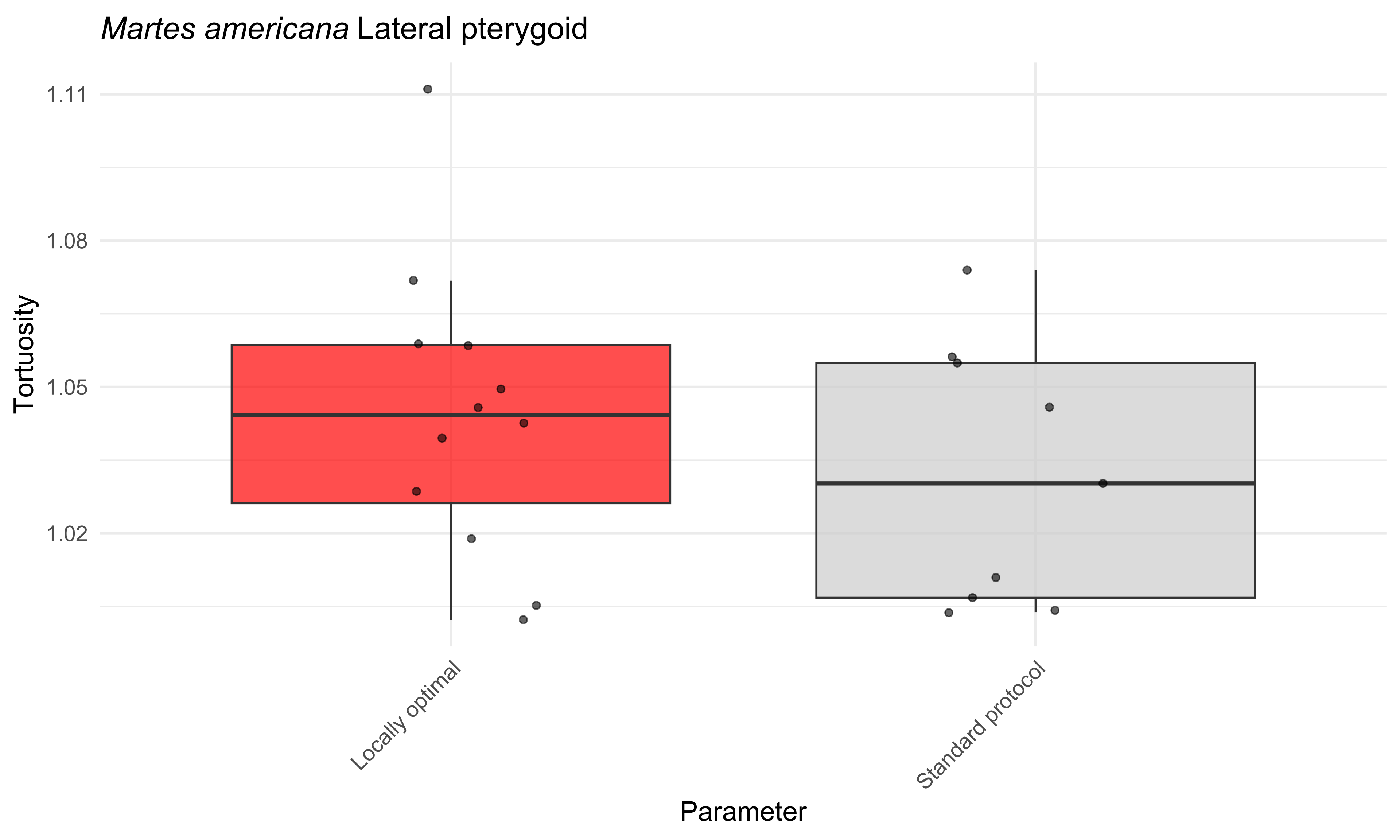
Martes americana Lateral pterygoid
Tortuosity
1.11
1.08
1.05
1.02
Locally optimal
Standard protocol
Parameter

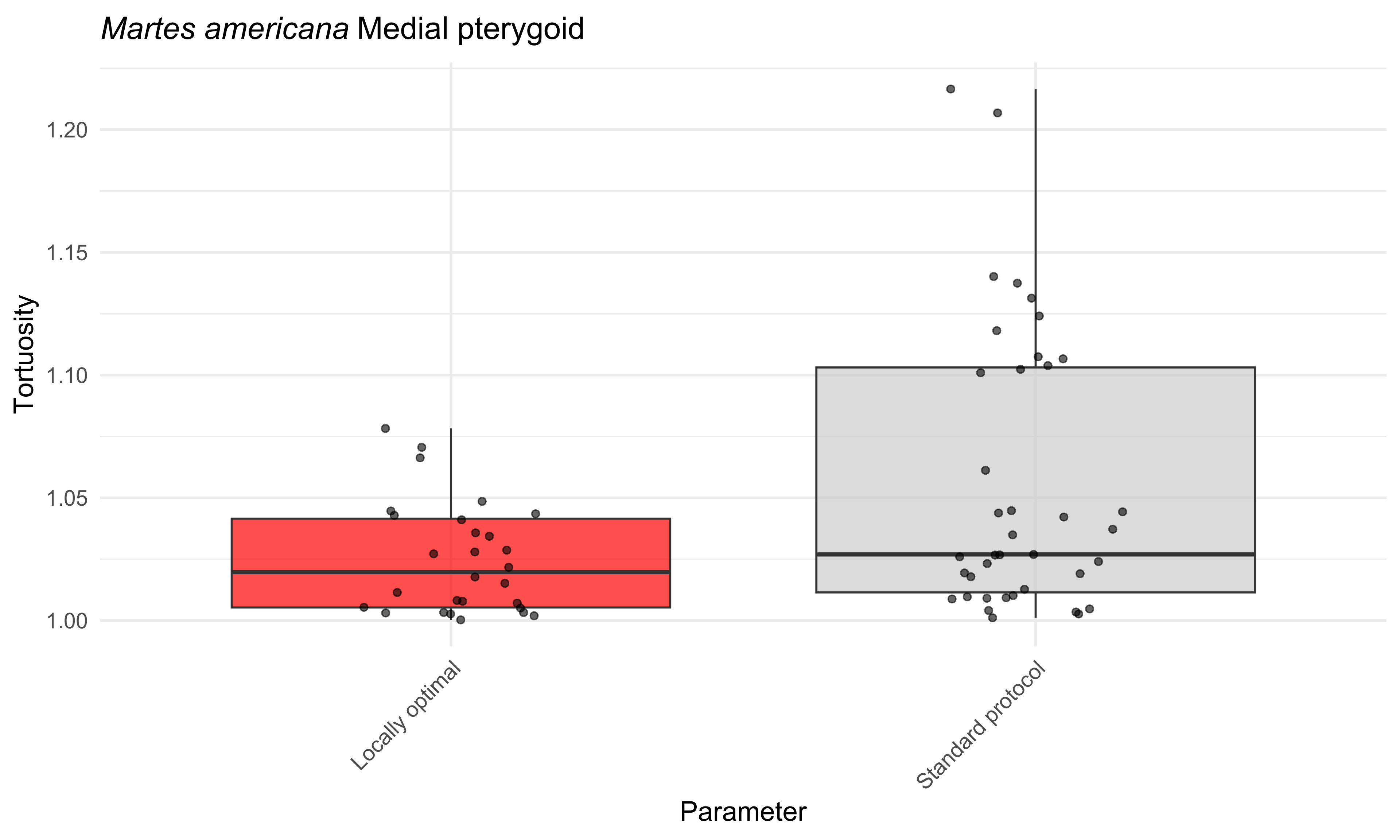

Martes americana Medial pterygoid
Tortuosity
1.20
1.15
1.10
1.05
1.00
Locally optimal
Standard protocol
Parameter

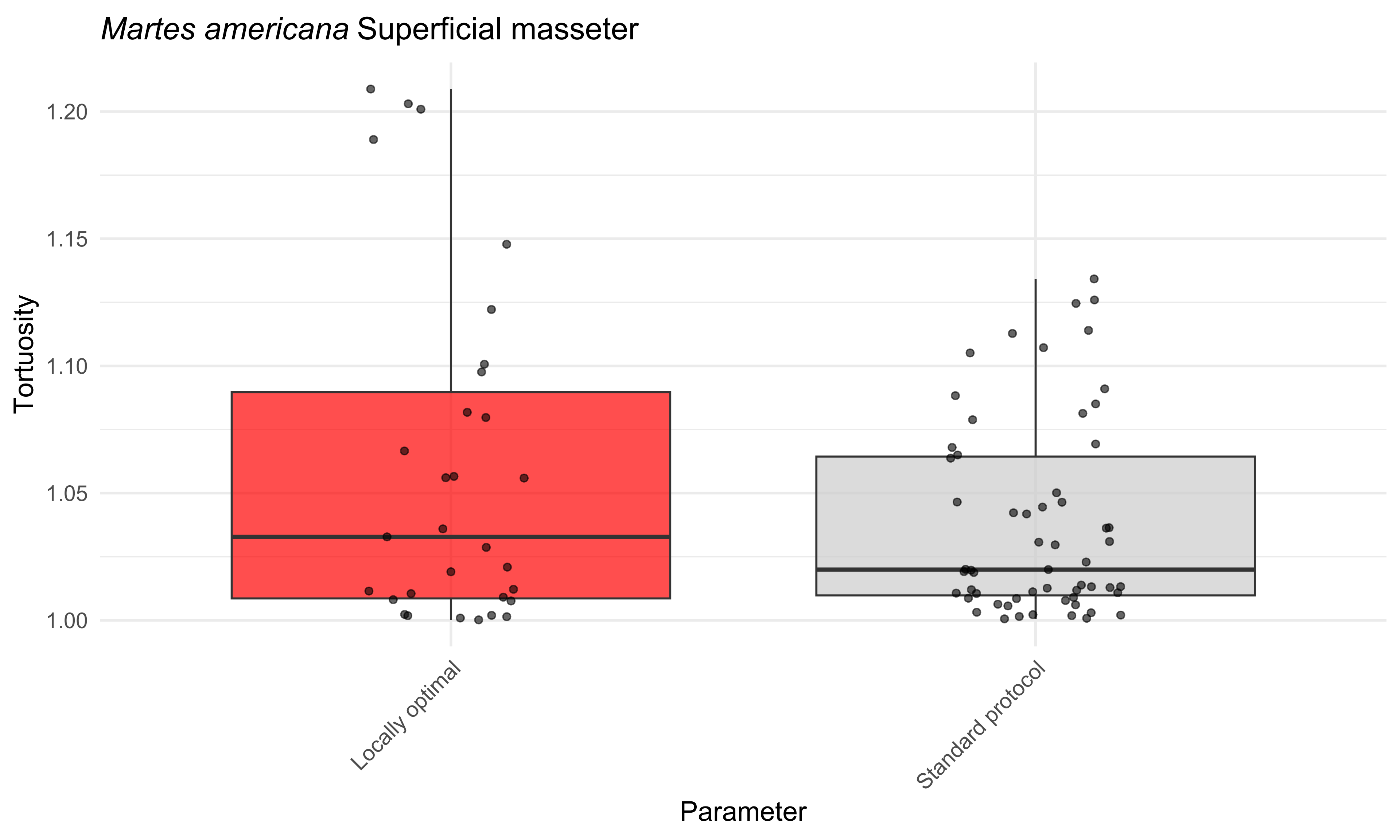

Martes americana Superficial masseter
Tortuosity
1.20
1.15
1.10
1.05
1.00
Locally optimal
Standard protocol
Parameter

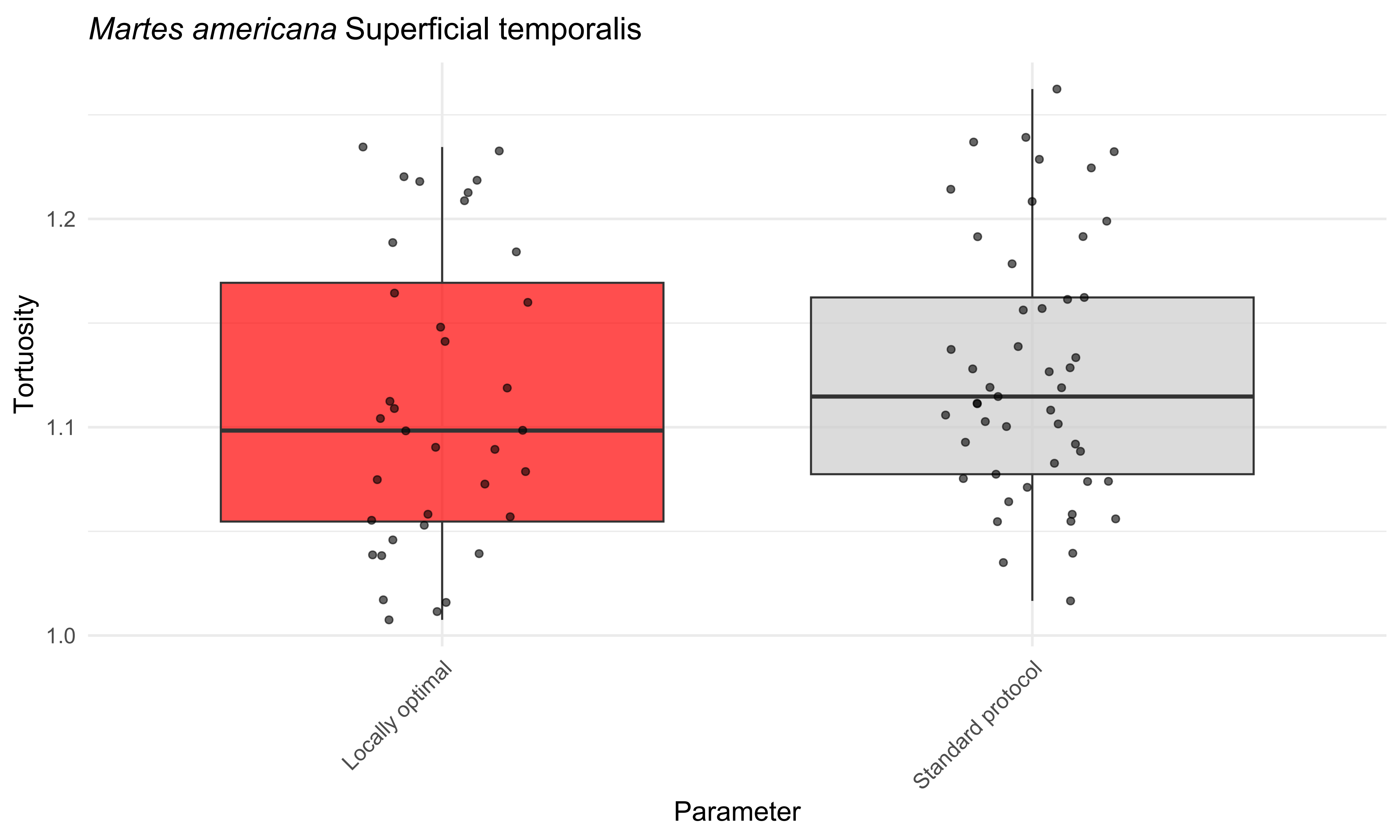
Martes americana Superficial temporalis
Tortuosity
1.2
1.1
1.0
Locally optimal
Standard protocol
Parameter

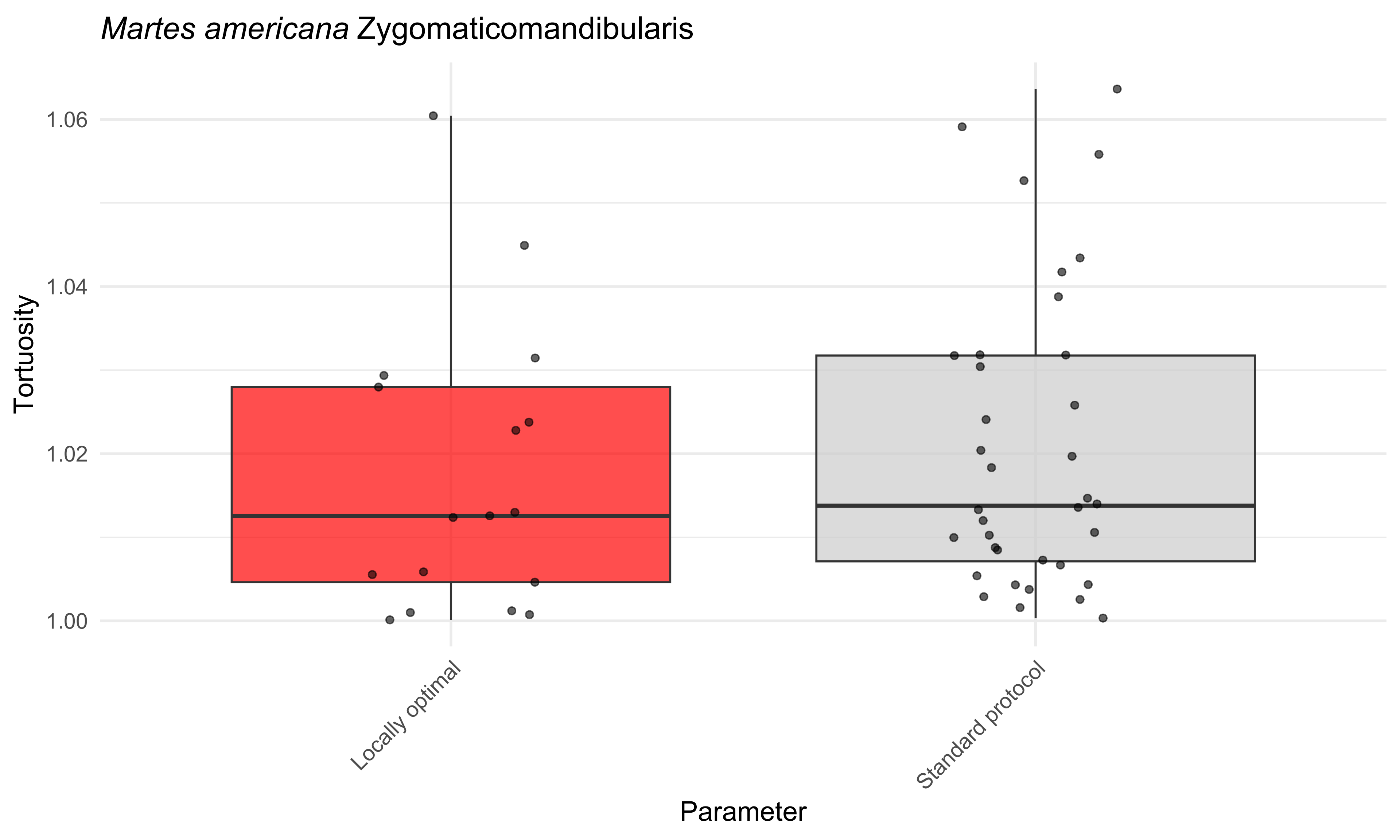
Martes americana Zygomaticomandibularis
Tortuosity
1.06
1.04
1.02
1.00
Locally optimal
Standard protocol
Parameter

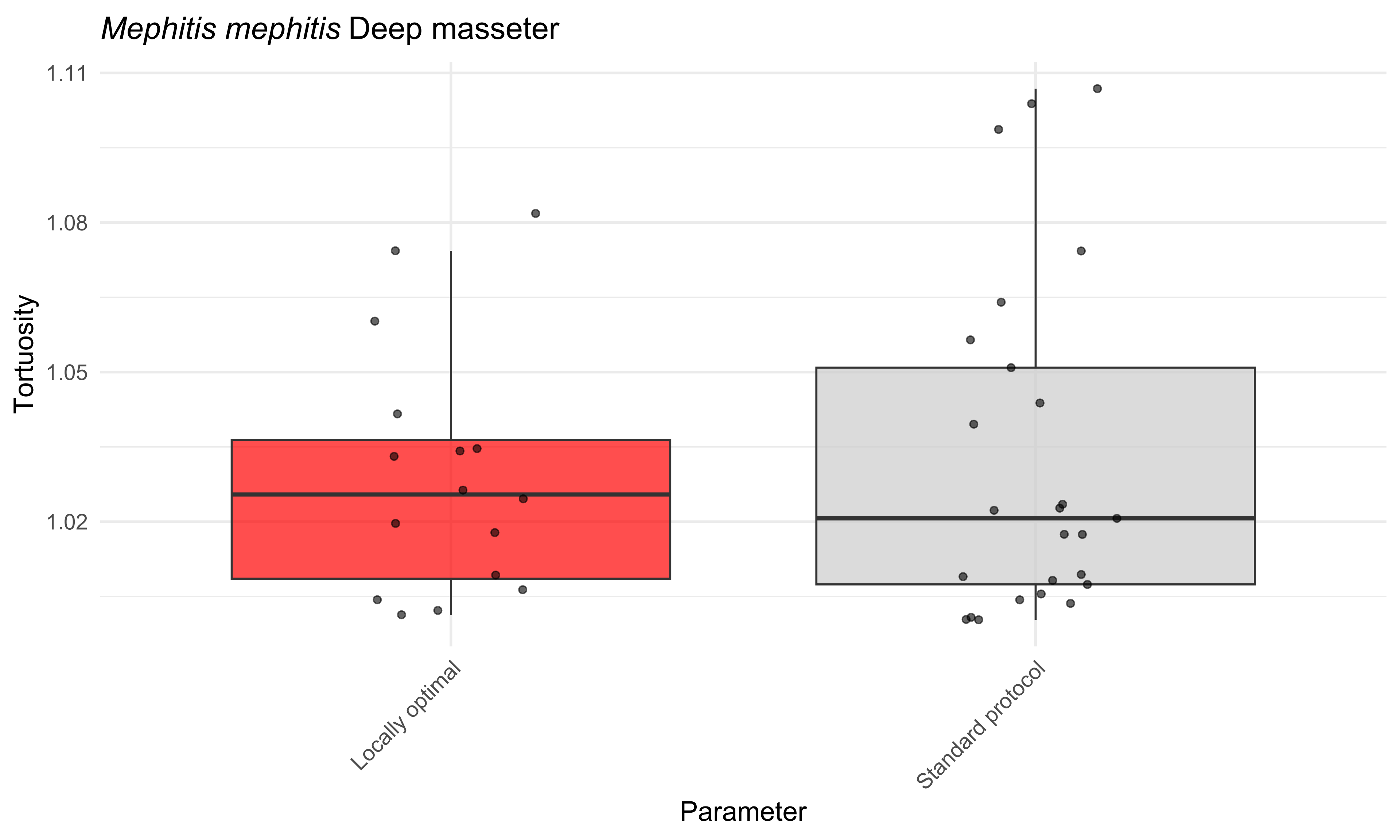
Mephitis mephitis Deep masseter
1.11
1.08
1.05
1.02
Tortuosity
Locally optimal
Standard protocol
Parameter

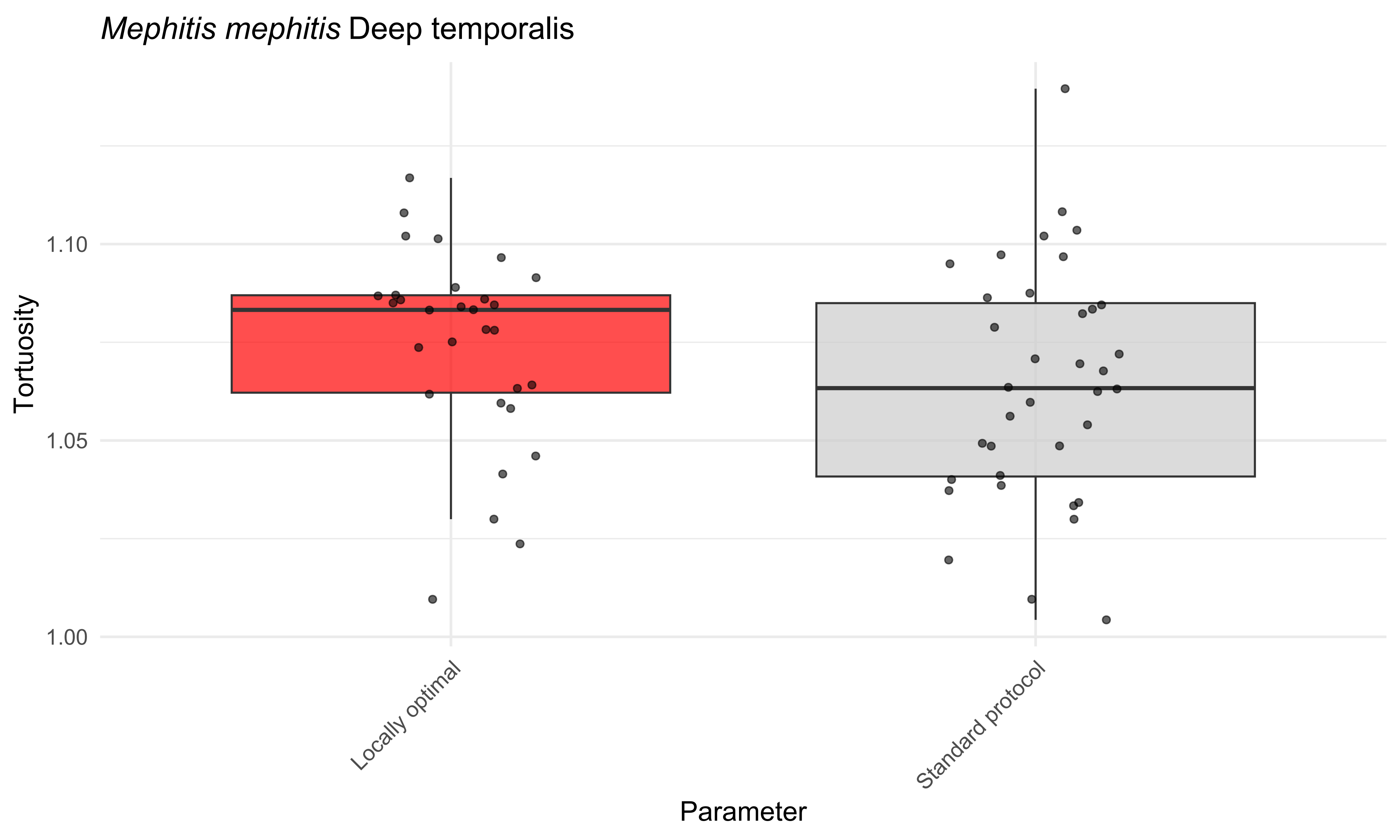

Mephitis mephitis Deep temporalis
Tortuosity
1.10
1.05
1.00
Locally optimal
Standard protocol
Parameter

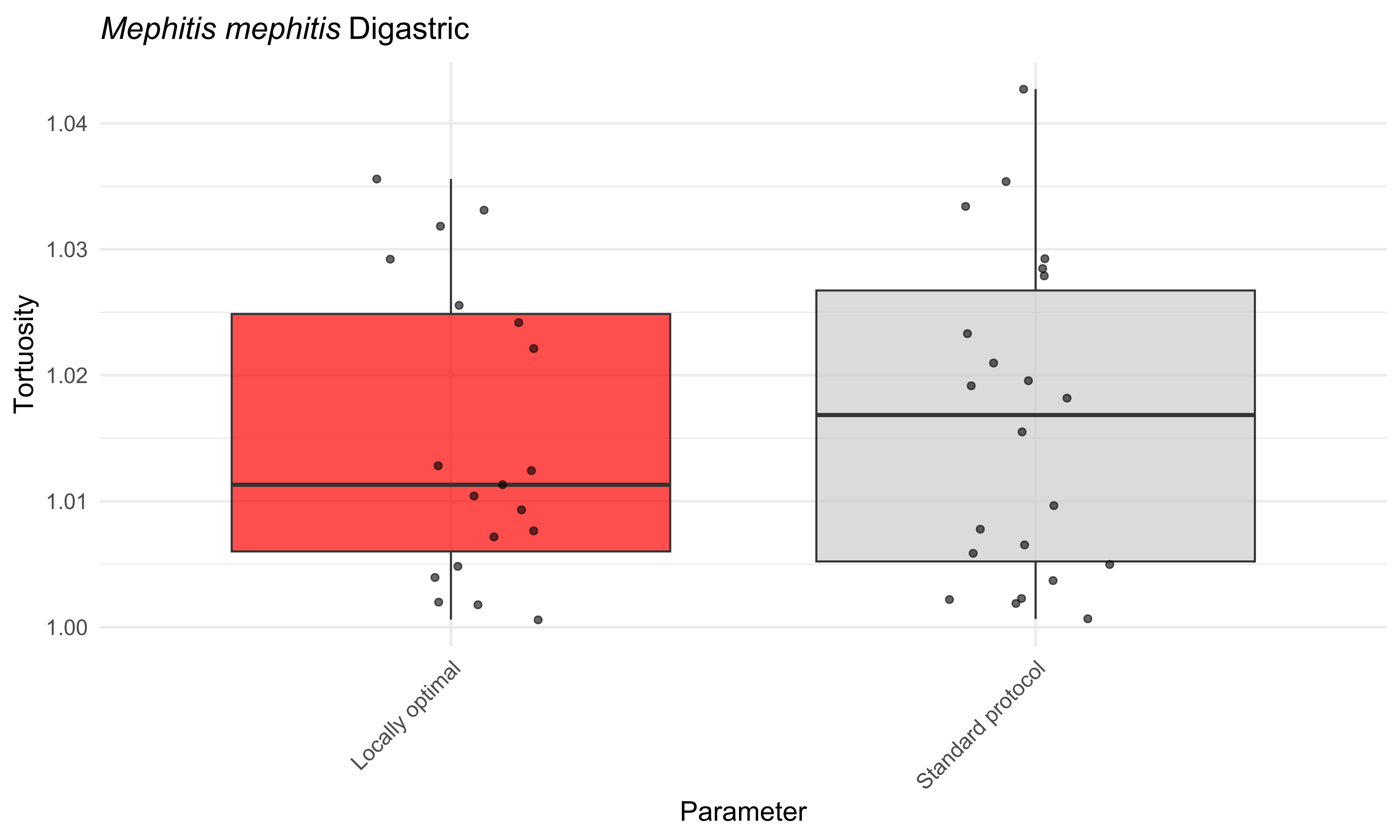

Mephitis mephitis Digastric
Tortuosity
1.04
1.03
1.02
1.01
1.00
Locally optimal
Standard protocol
Parameter

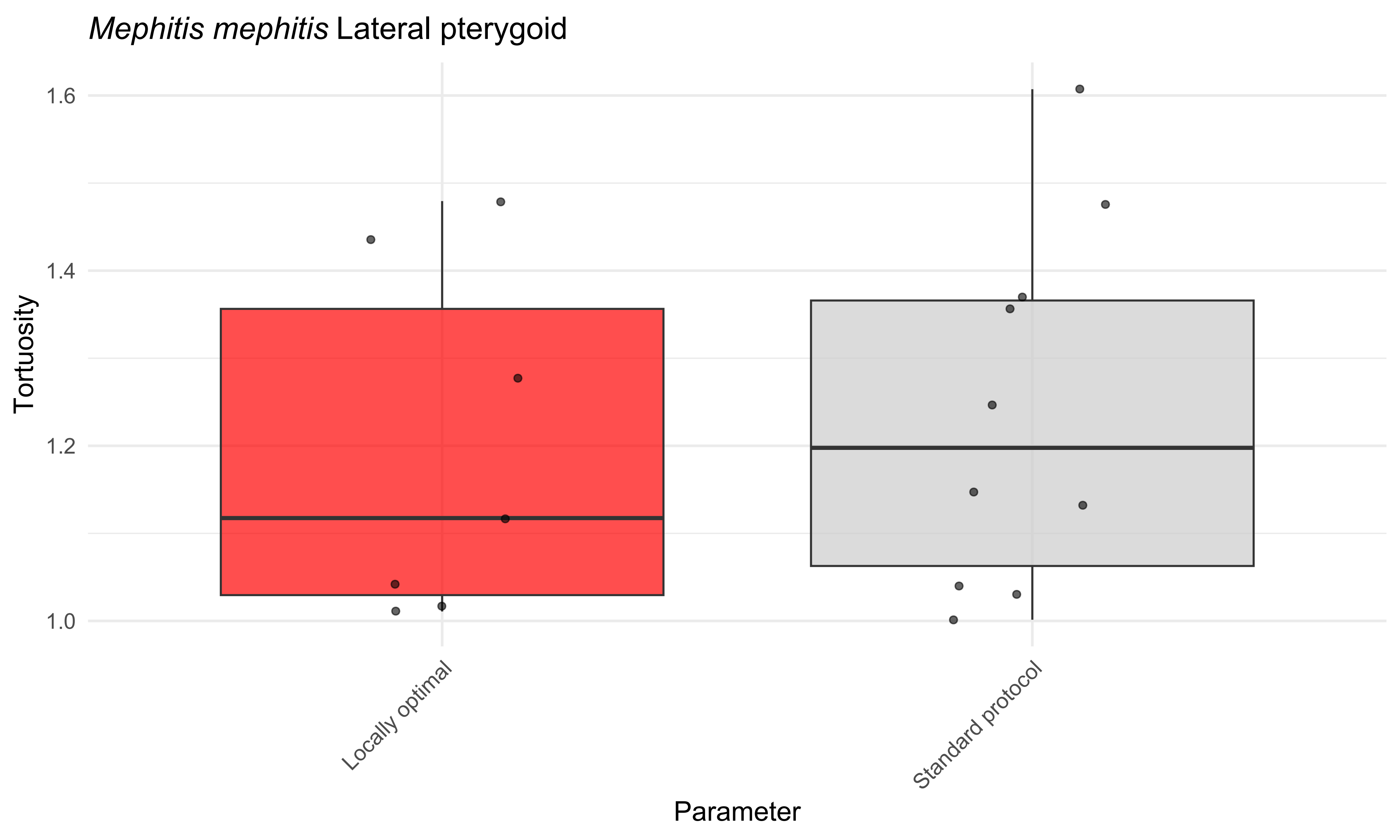

Mephitis mephitis Lateral pterygoid
1.6
1.4
1.2
1.0
Tortuosity
Locally optimal
Standard protocol
Parameter

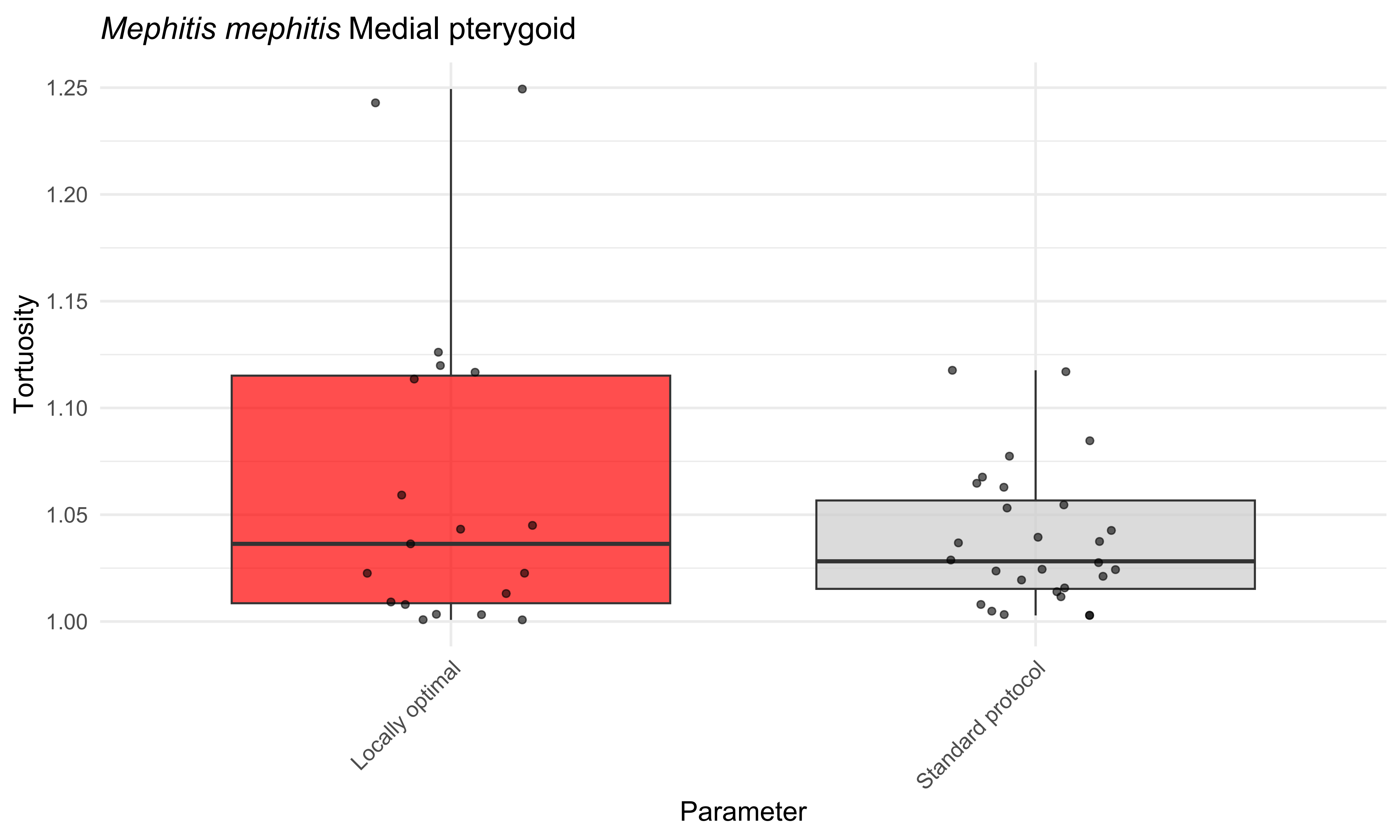
Mephitis mephitis Medial pterygoid
1.25
1.20
1.15
1.10
1.05
1.00
Tortuosity
Locally optimal
Standard protocol
Parameter

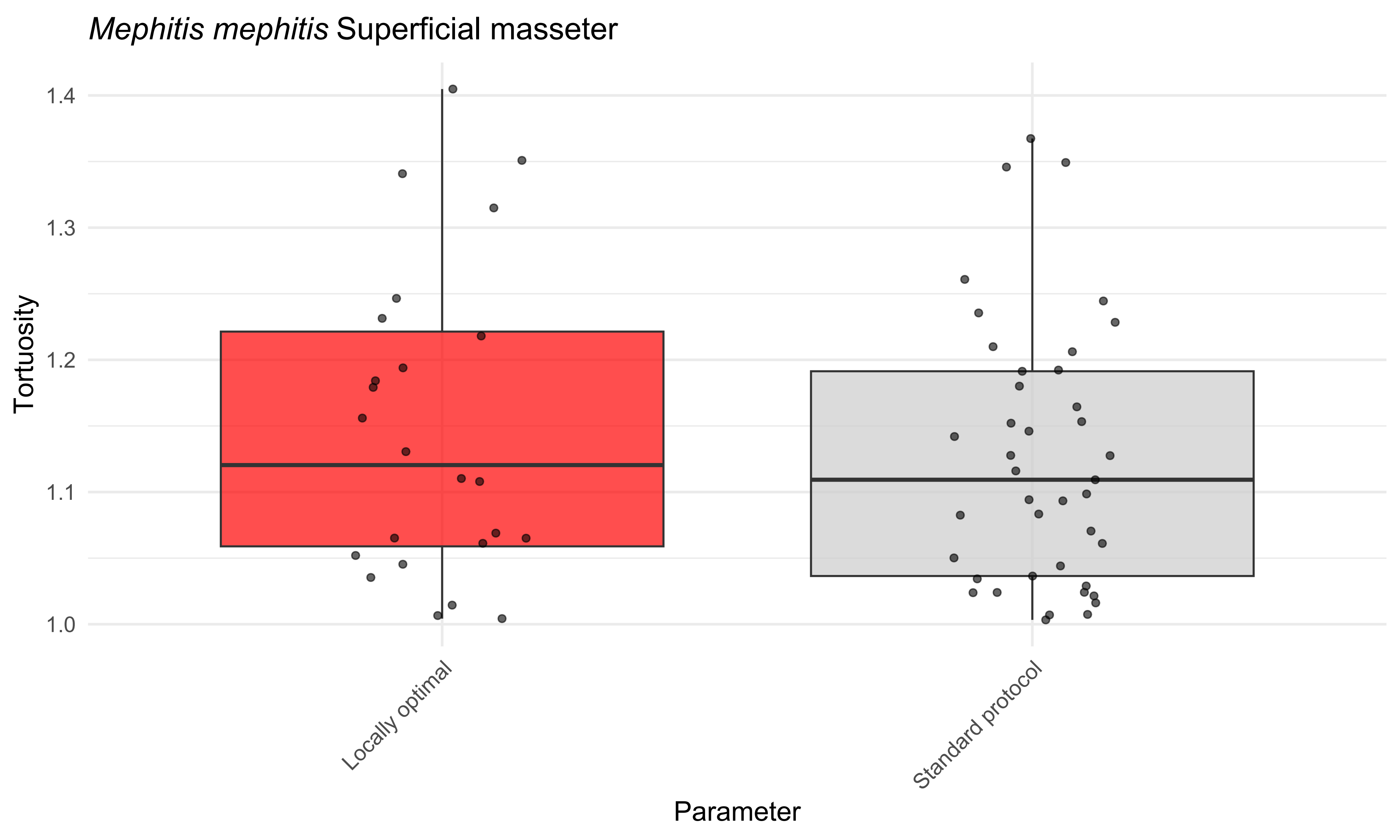

Mephitis mephitis Superficial masseter
Tortuosity
1.4
1.3
1.2
1.1
1.0
Locally optimal
Standard protocol
Parameter

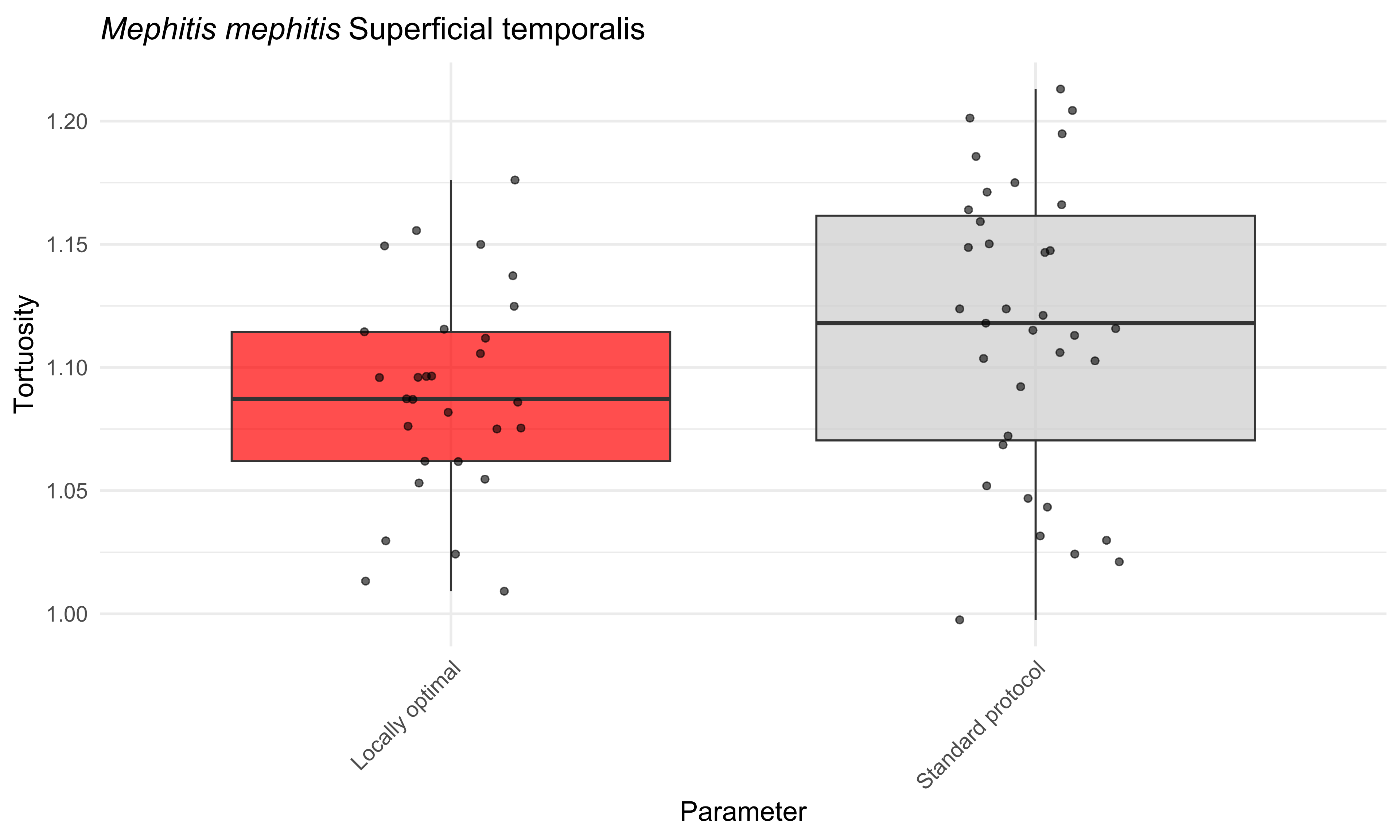

Mephitis mephitis Superficial temporalis
Tortuosity
1.20
1.15
1.10
1.05
1.00
Locally optimal
Standard protocol
Parameter

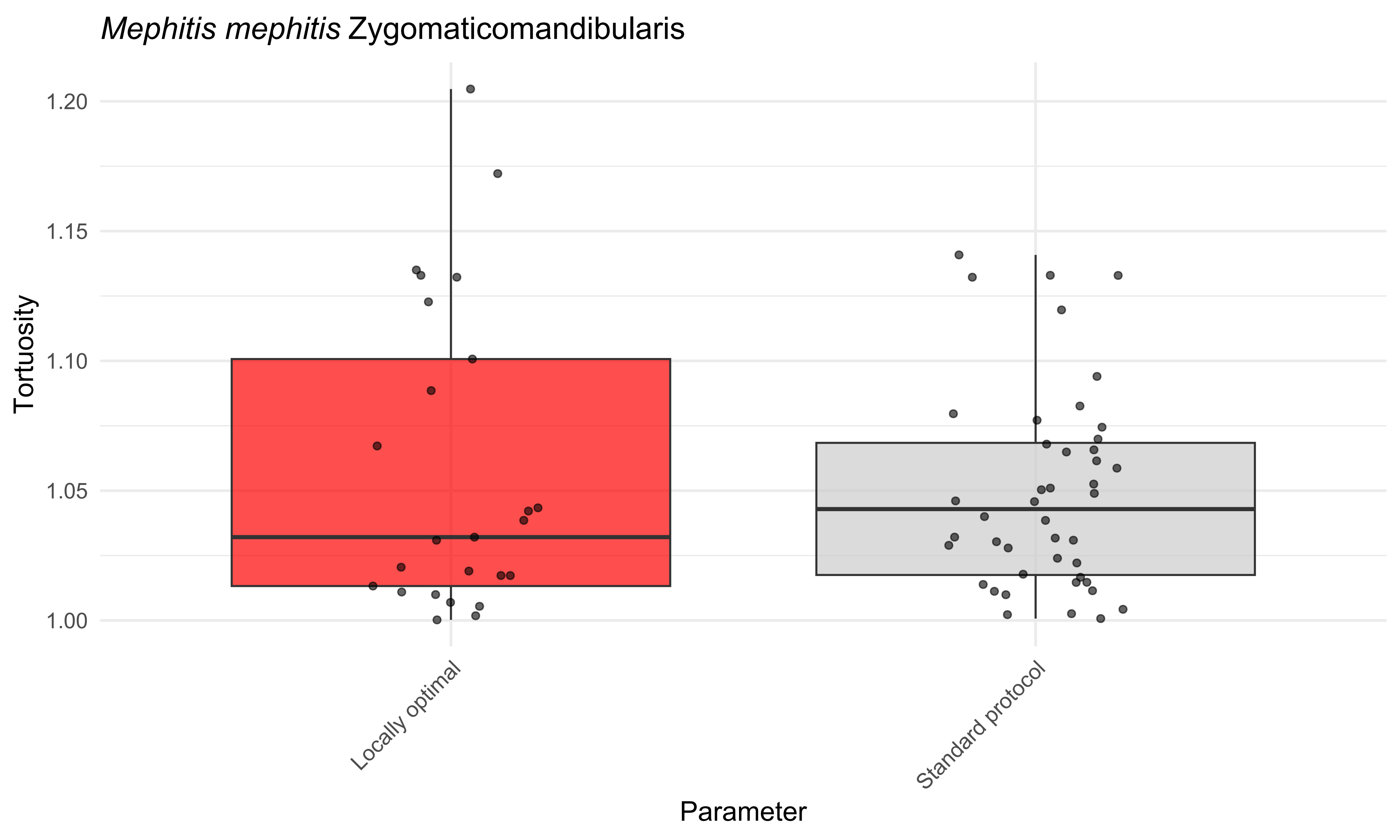

Mephitis mephitis Zygomaticomandibularis
Tortuosity
1.20
1.15
1.10
1.05
1.00
Locally optimal
Standard protocol
Parameter

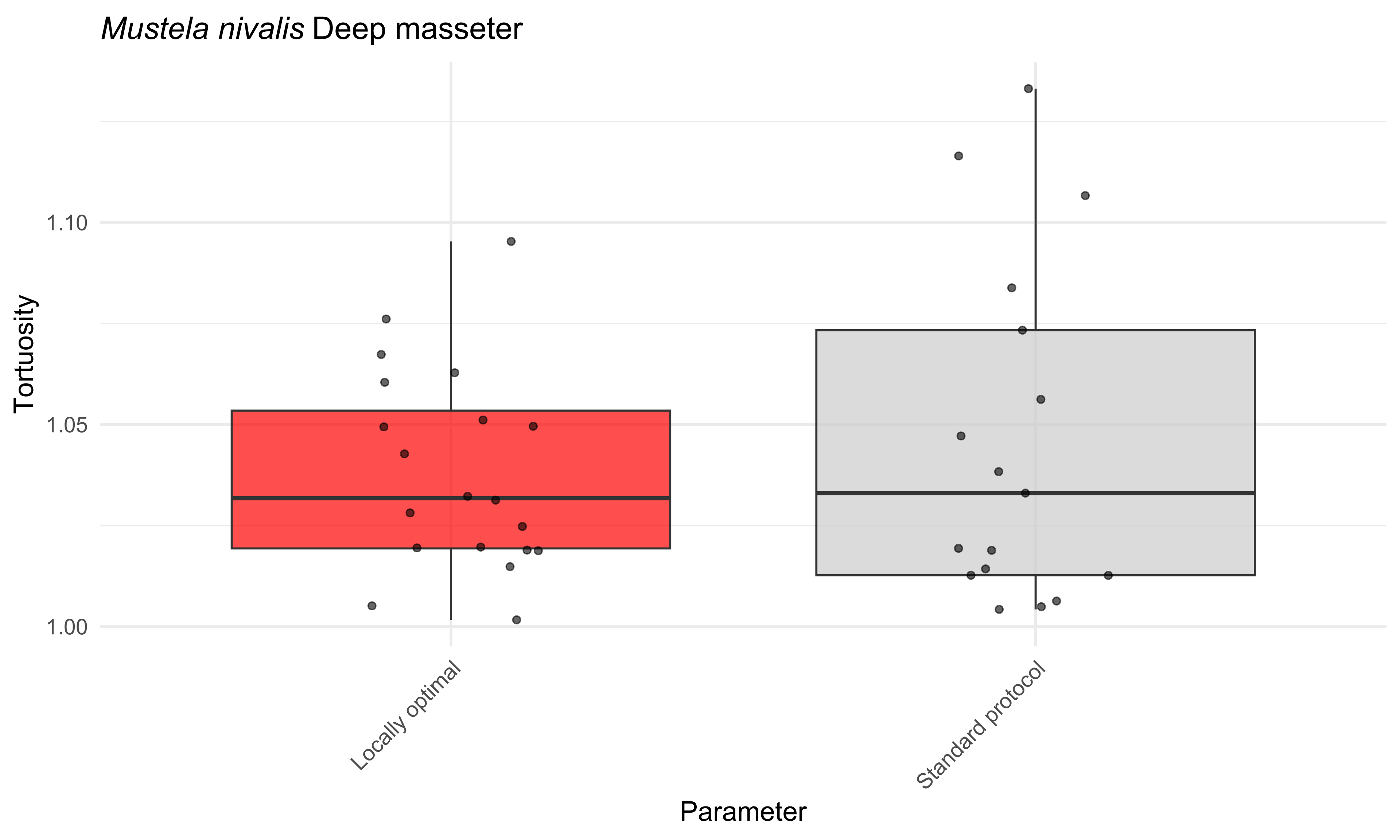

Mustela nivalis Deep masseter
Tortuosity
1.10
1.05
1.00
Locally optimal
Standard protocol
Parameter

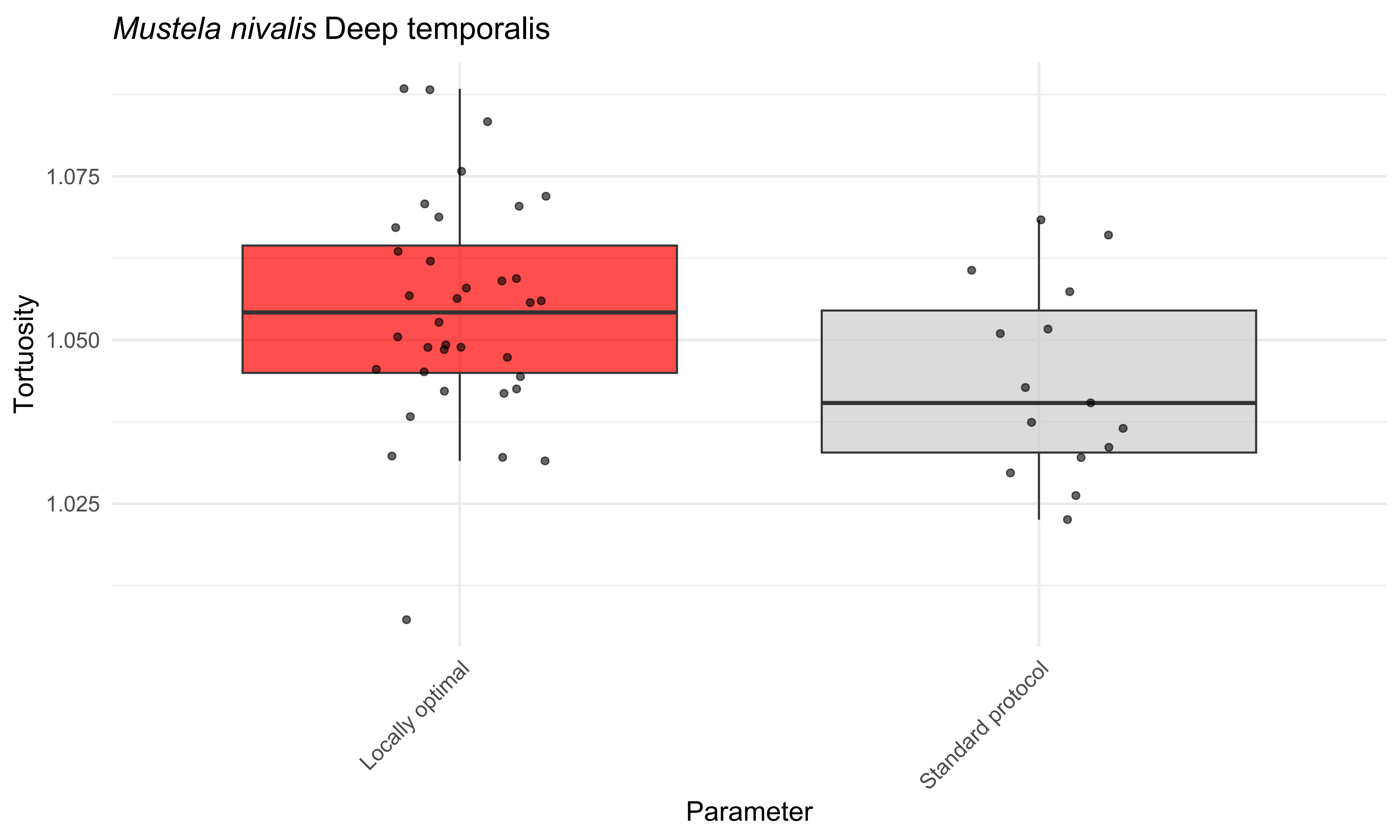

Mustela nivalis Deep temporalis
Tortuosity
1.075
1.050
1.025
Locally optimal
Standard protocol
Parameter

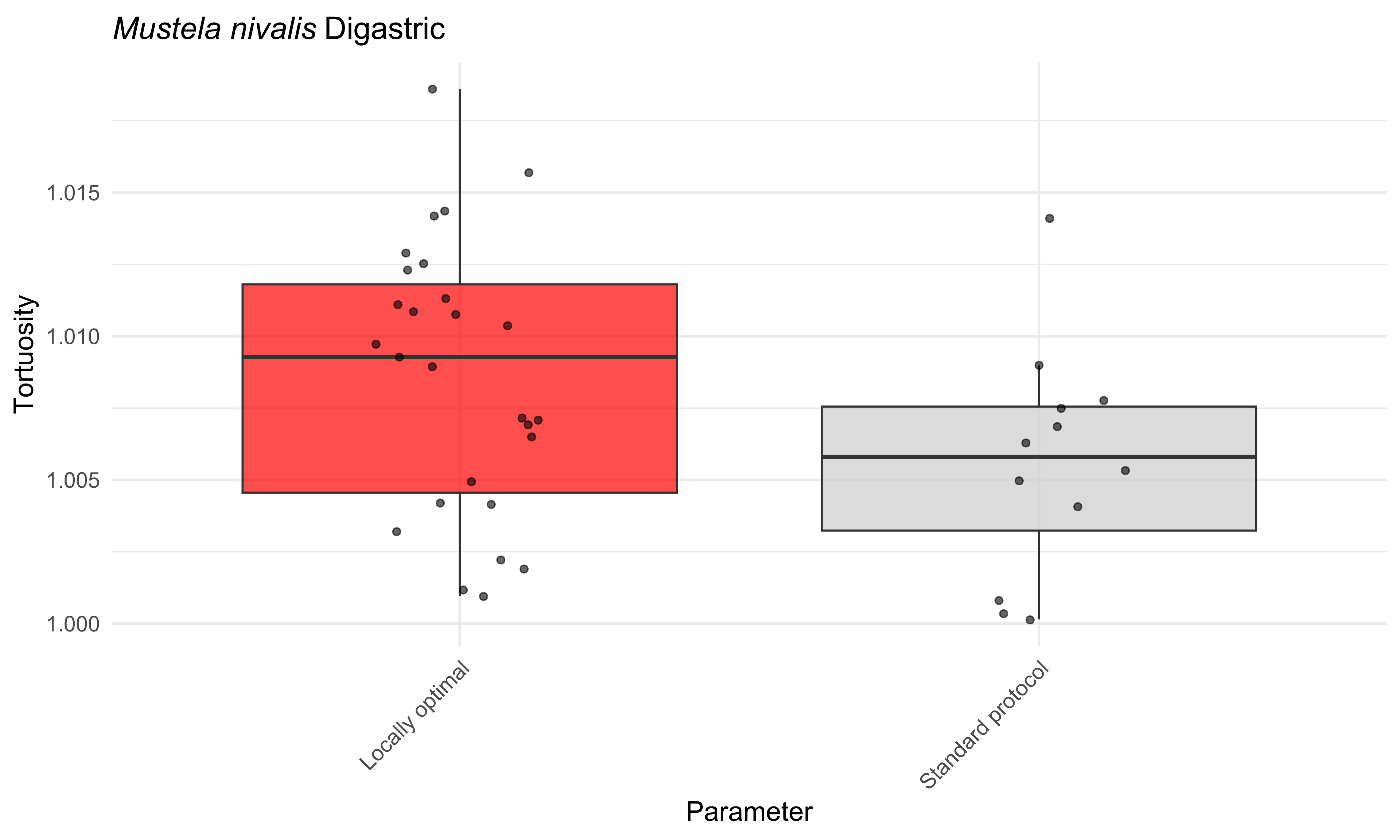

Mustela nivalis Digastric
Tortuosity
1.015
1.010
1.005
1.000
Locally optimal
Standard protocol
Parameter

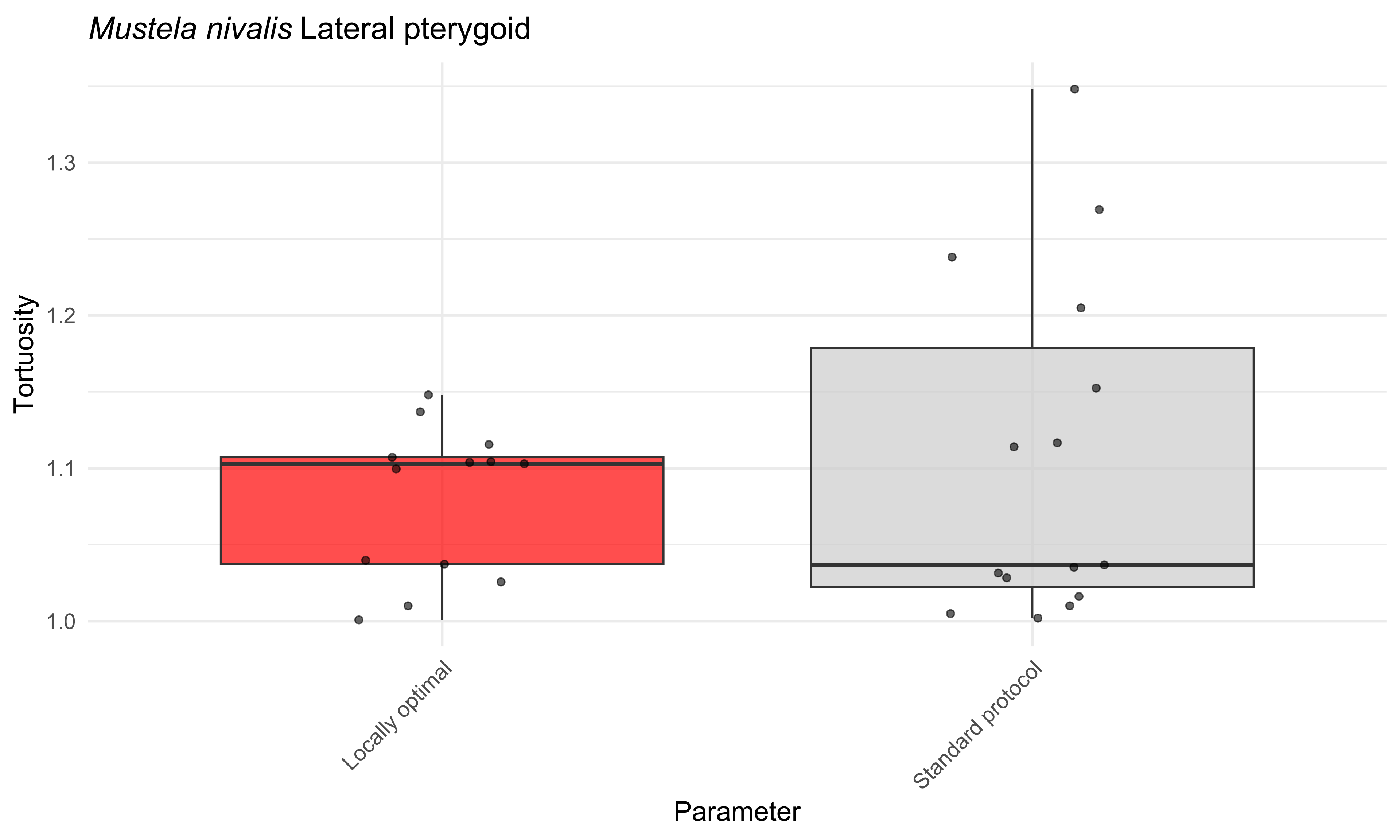

Mustela nivalis Lateral pterygoid
Tortuosity
1.3
1.2
1.1
1.0
Locally optimal
Standard protocol
Parameter

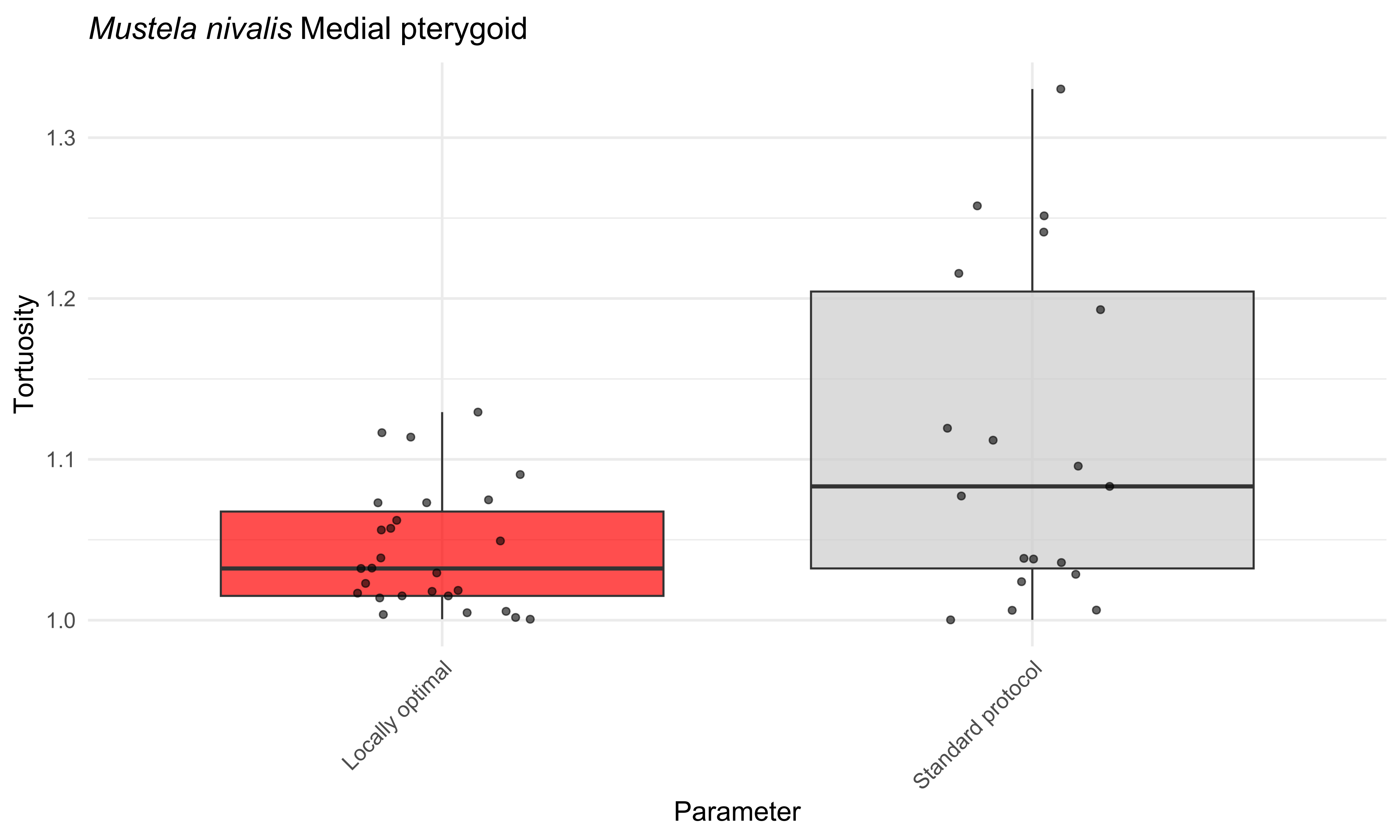

Mustela nivalis Medial pterygoid
Tortuosity
1.3
1.2
1.1
1.0
Locally optimal
Standard protocol
Parameter

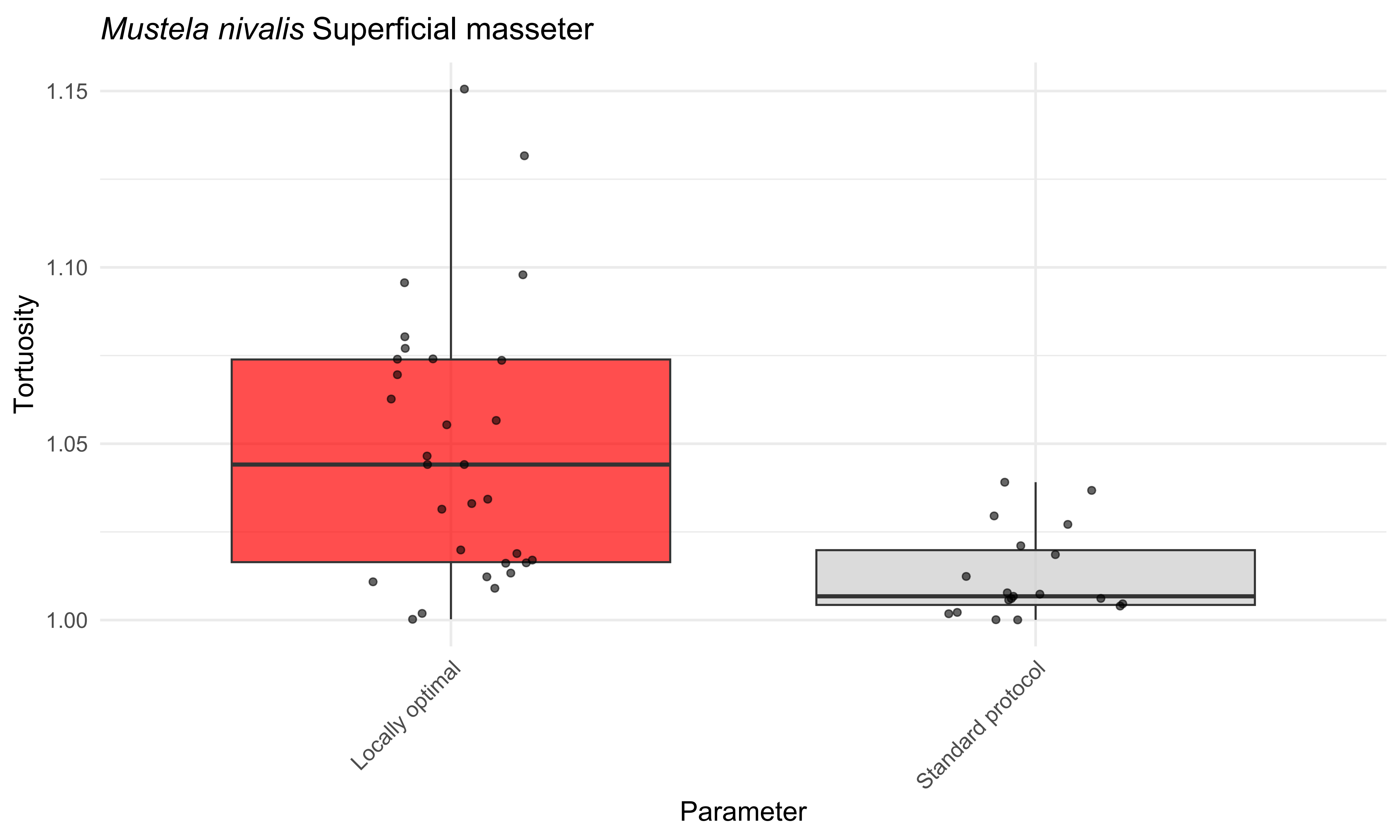

Mustela nivalis Superficial masseter
1.15
1.10
1.05
1.00
Tortuosity
Locally optimal
Standard protocol
Parameter

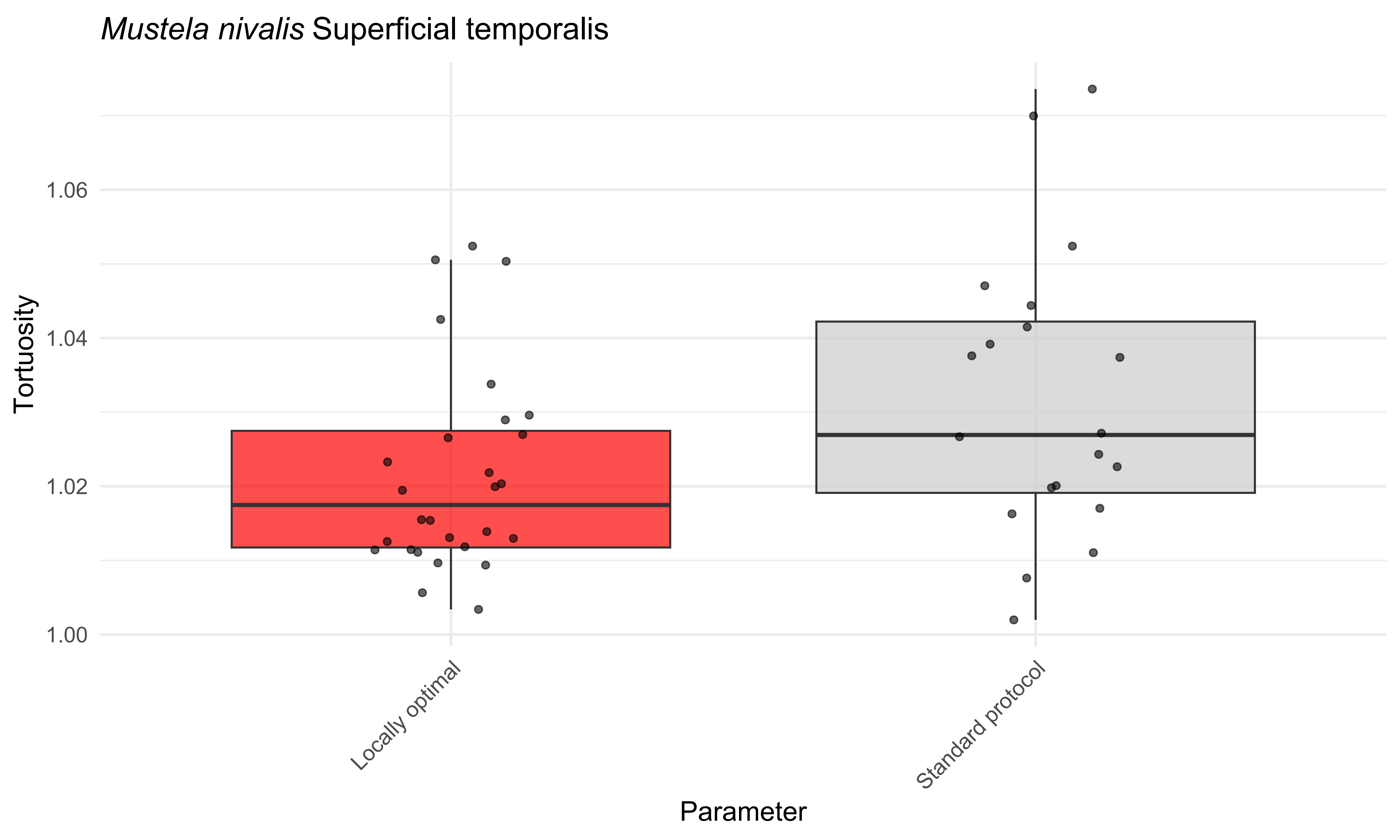
Mustela nivalis Superficial temporalis
Tortuosity
1.06
1.04
1.02
1.00
Locally optimal
Standard protocol
Parameter

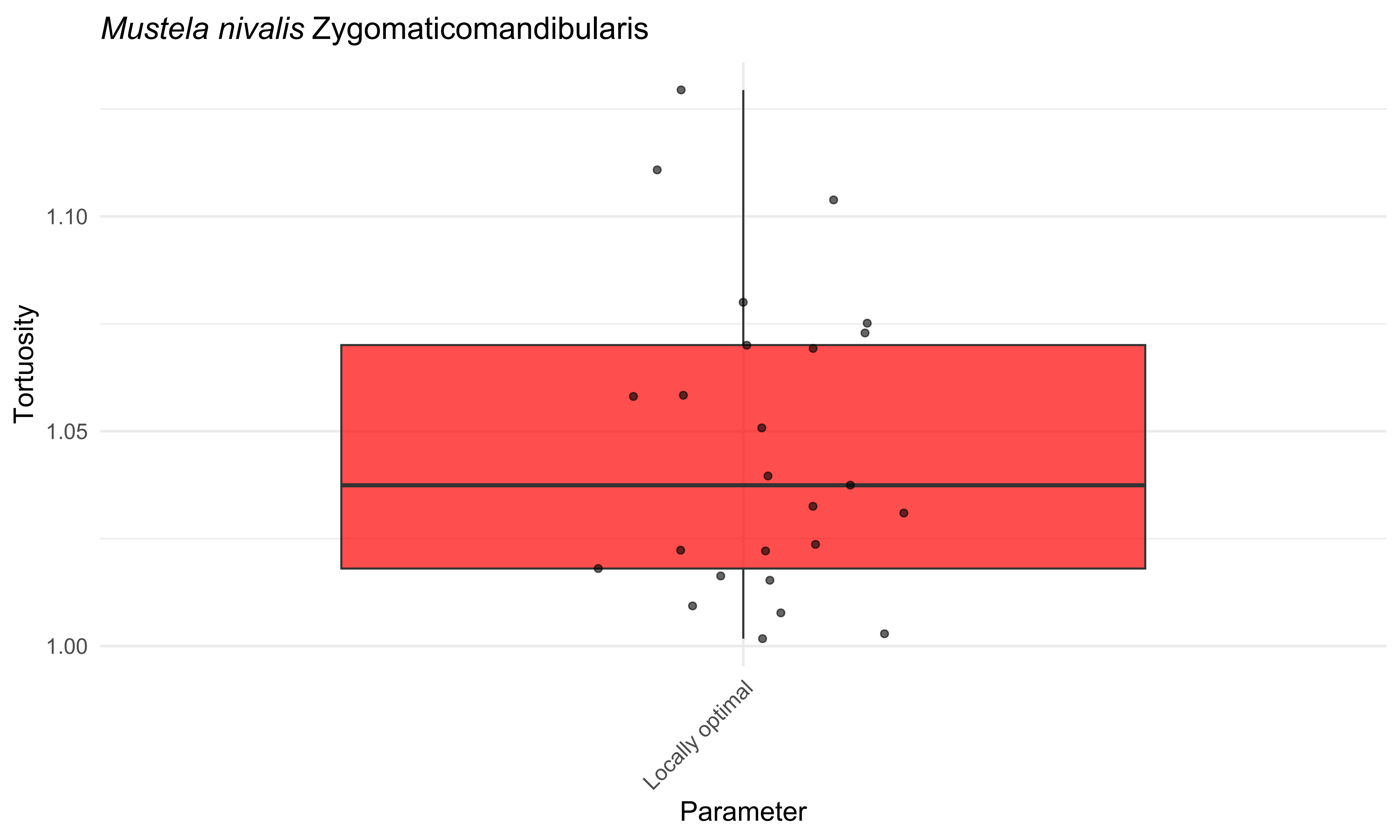

Mustela nivalis Zygomaticomandibularis
Tortuosity
1.10
1.05
1.00
Locally optimal
Parameter

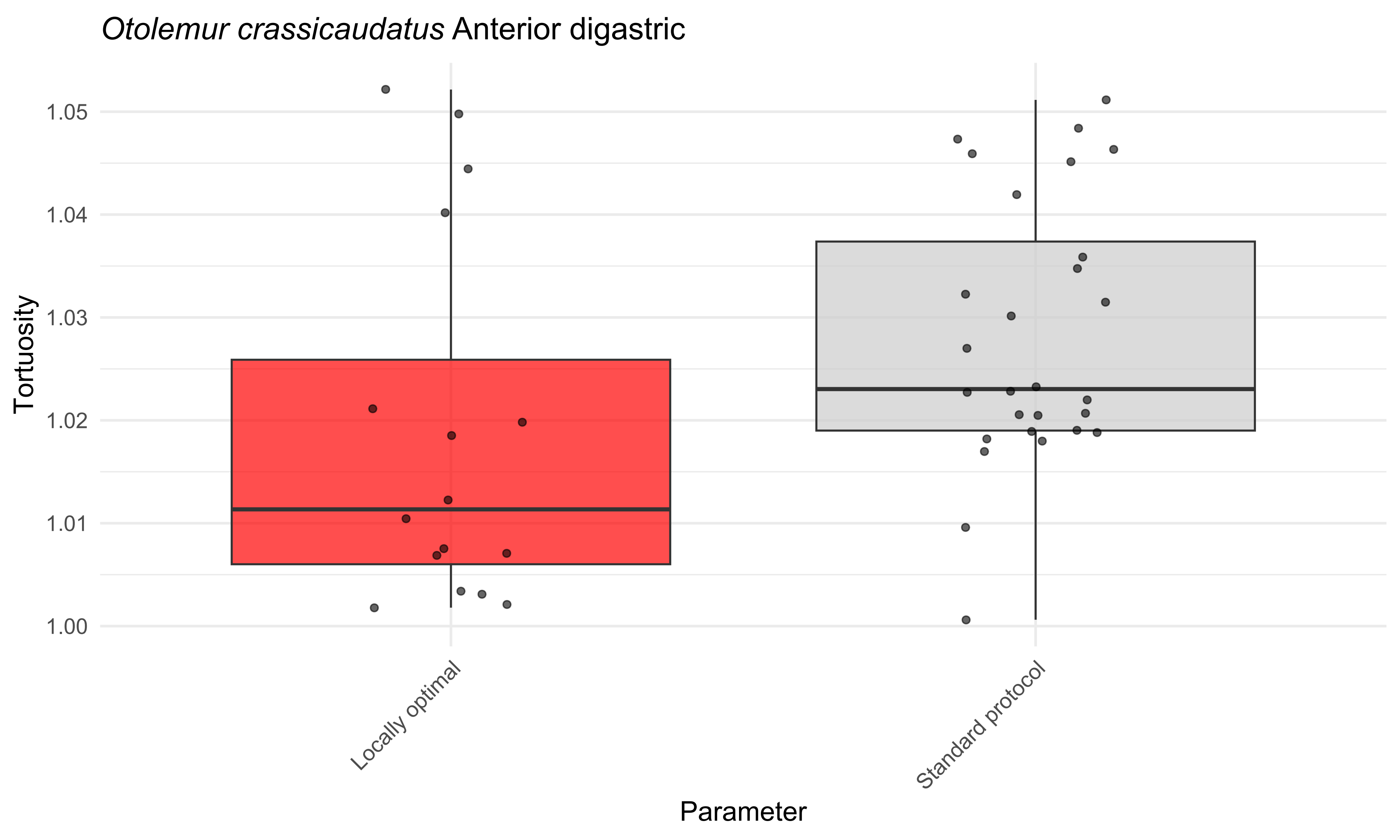

Otolemur crassicaudatus Anterior digastric
Tortuosity
1.05
1.04
1.03
1.02
1.01
1.00
Locally optimal
Standard protocol
Parameter

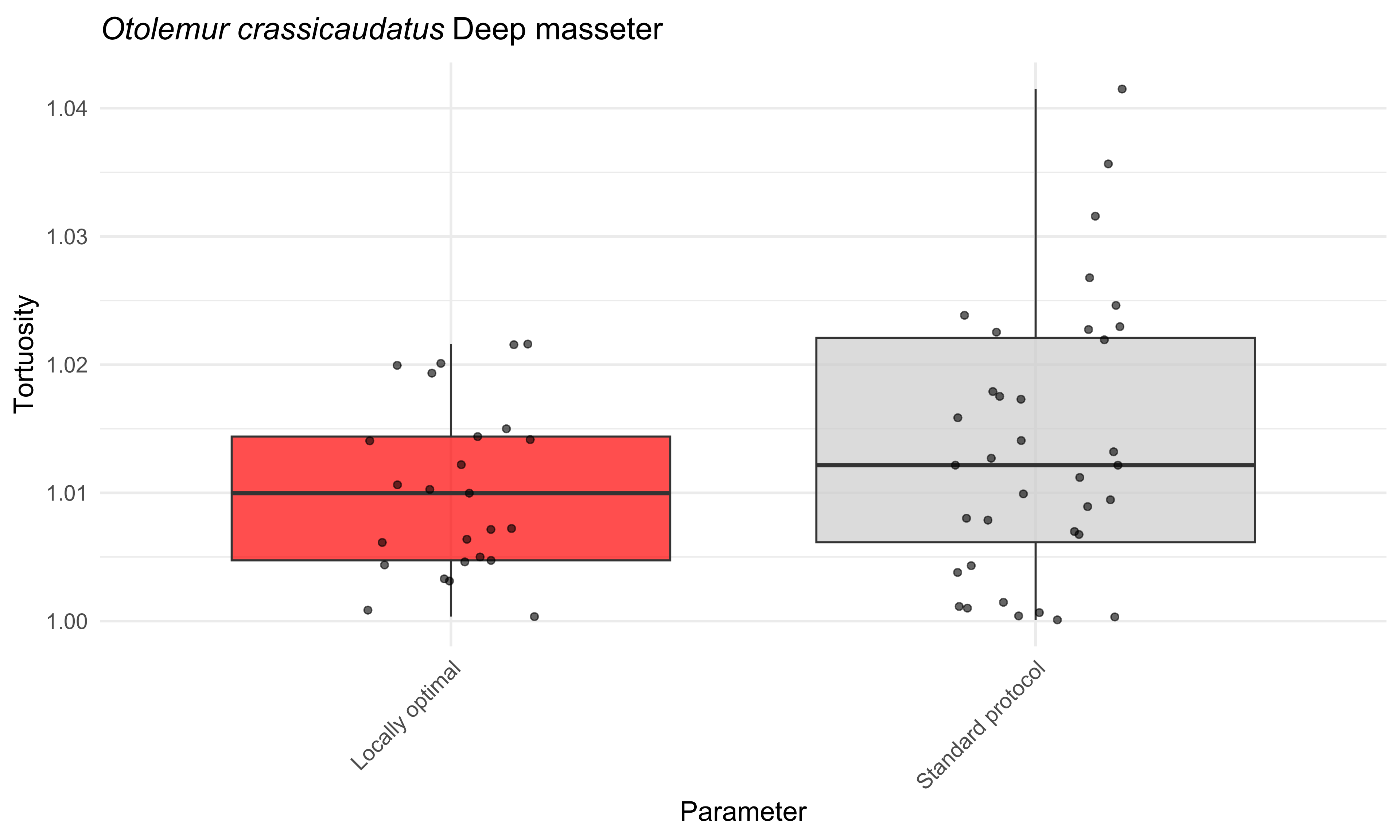

Otolemur crassicaudatus Deep masseter
Tortuosity
1.04
1.03
1.02
1.01
1.00
Locally optimal
Standard protocol
Parameter

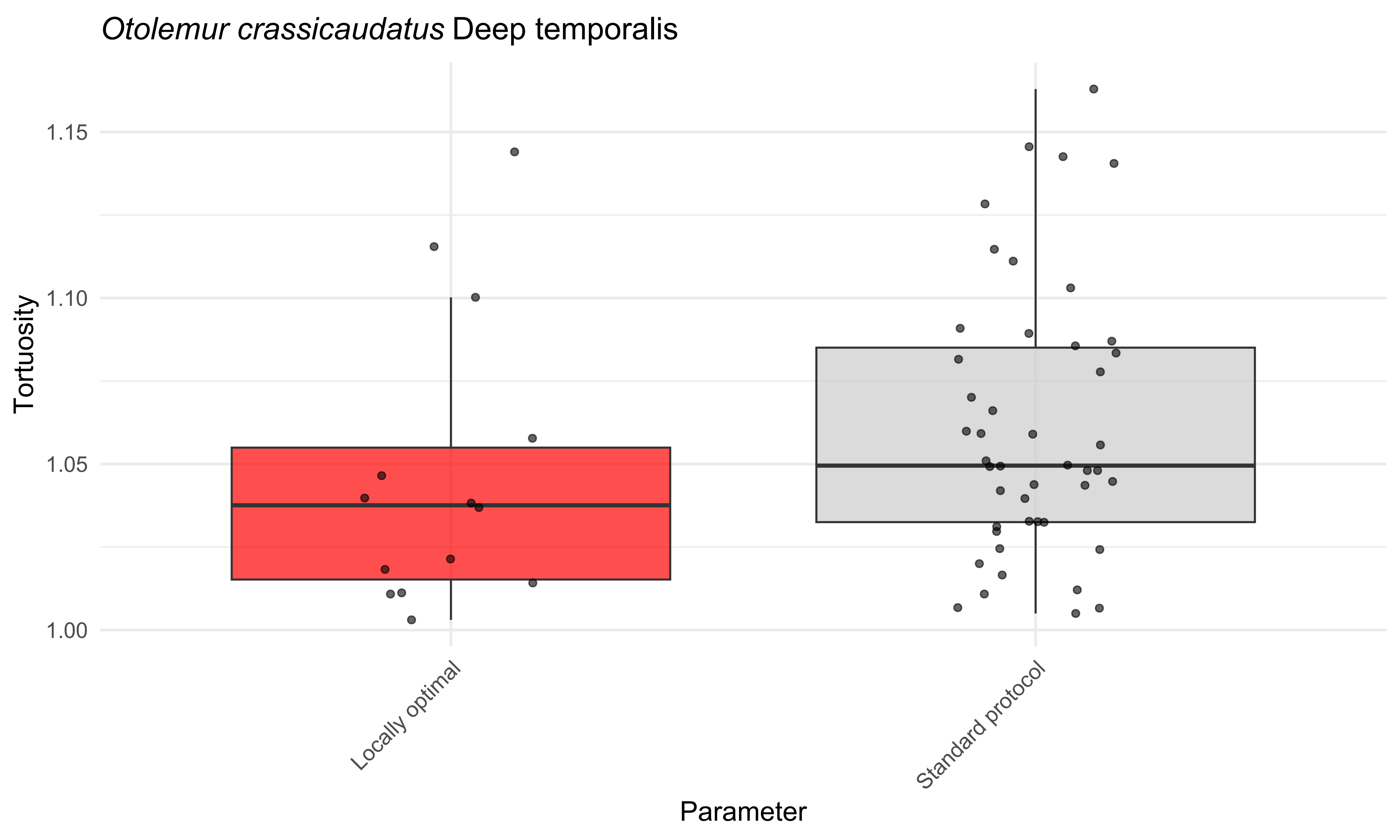

Otolemur crassicaudatus Deep temporalis
Tortuosity
1.15
1.10
1.05
1.00
Locally optimal
Standard protocol
Parameter

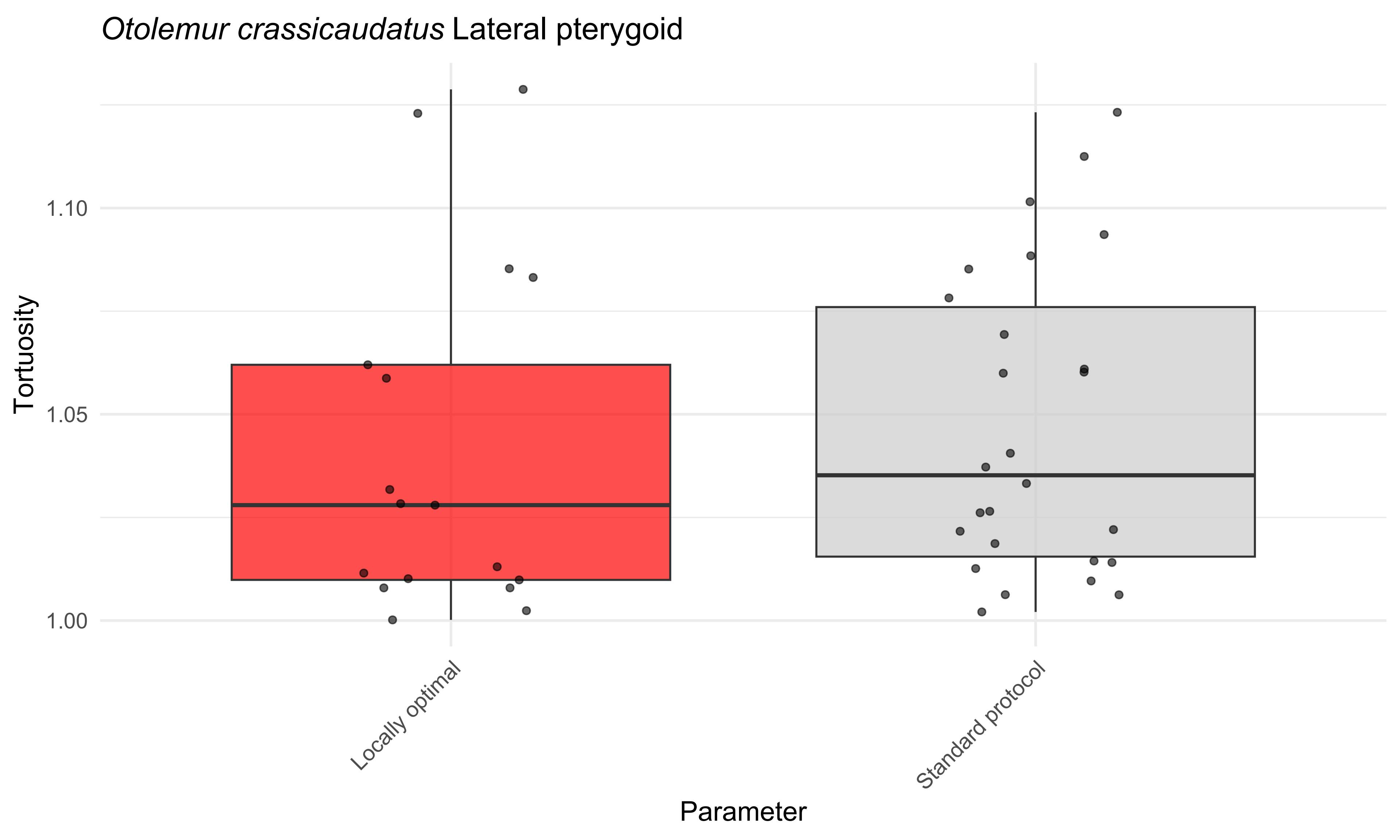
Otolemur crassicaudatus Lateral pterygoid
Tortuosity
1.10
1.05
1.00
Locally optimal
Standard protocol
Parameter

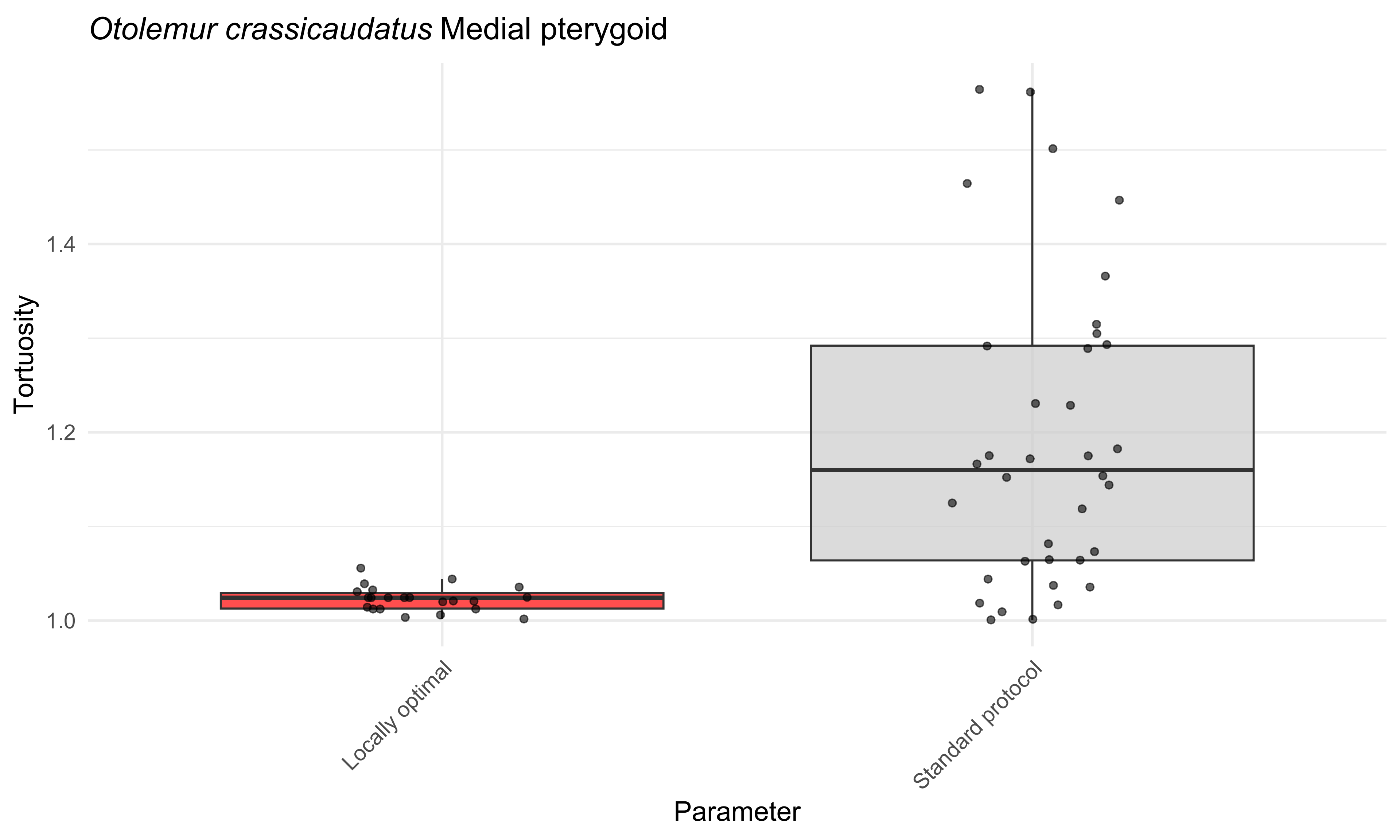

Otolemur crassicaudatus Medial pterygoid
Tortuosity
1.4
1.2
1.0
Locally optimal
Standard protocol
Parameter

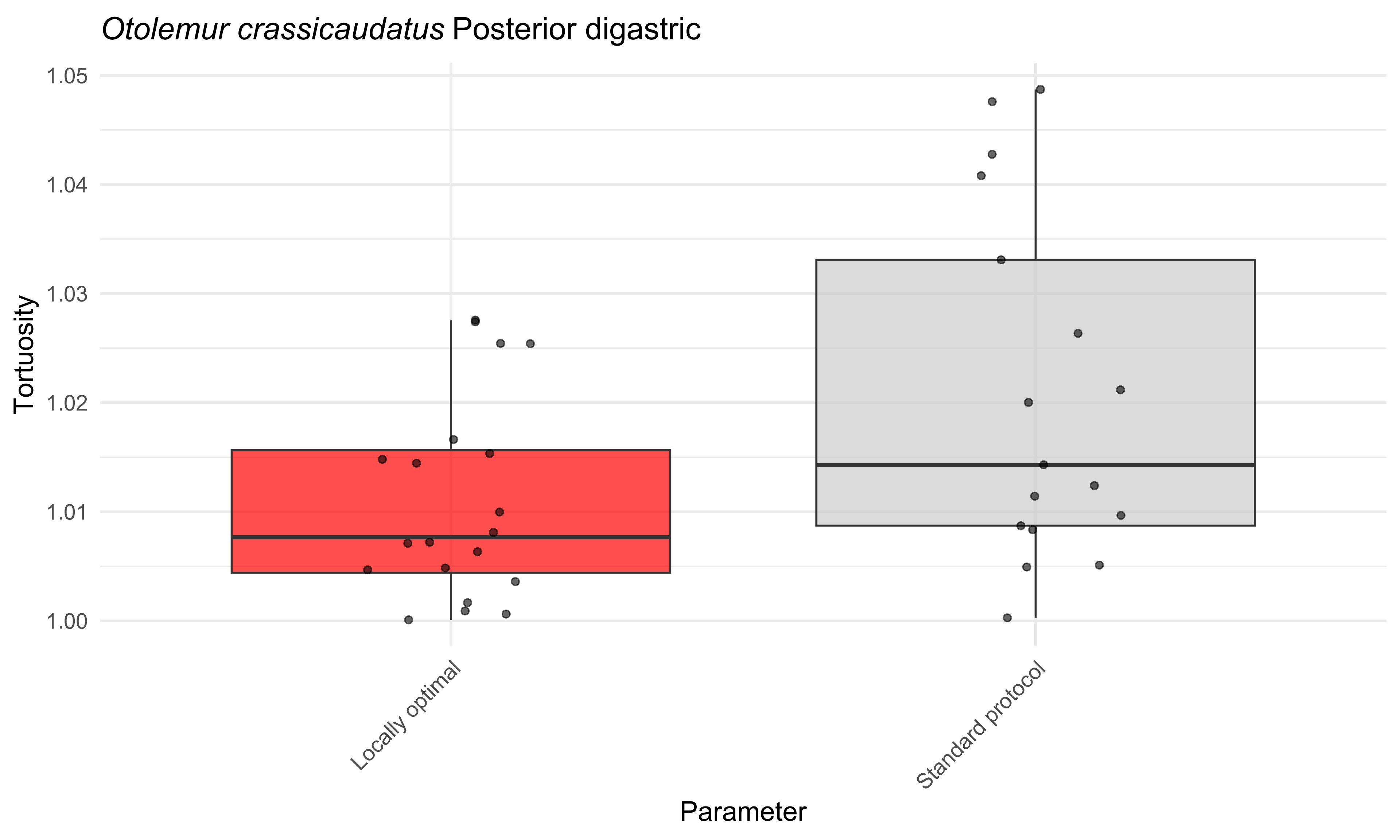

Otolemur crassicaudatus Posterior digastric
Tortuosity
1.05
1.04
1.03
1.02
1.01
1.00
Locally optimal
Standard protocol
Parameter

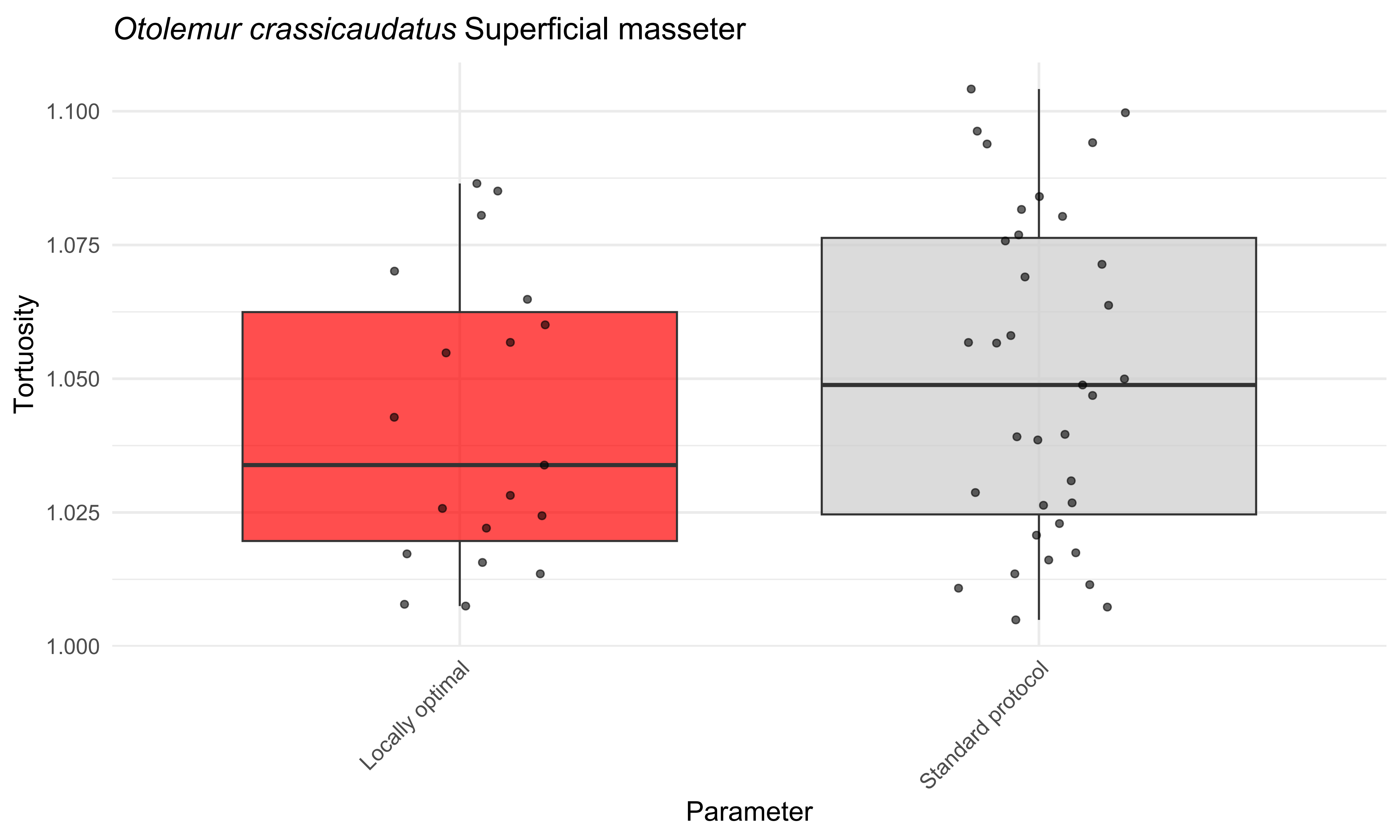

Otolemur crassicaudatus Superficial masseter
1.100
1.075
1.050
1.025
1.000
Tortuosity
Locally optimal
Standard protocol
Parameter

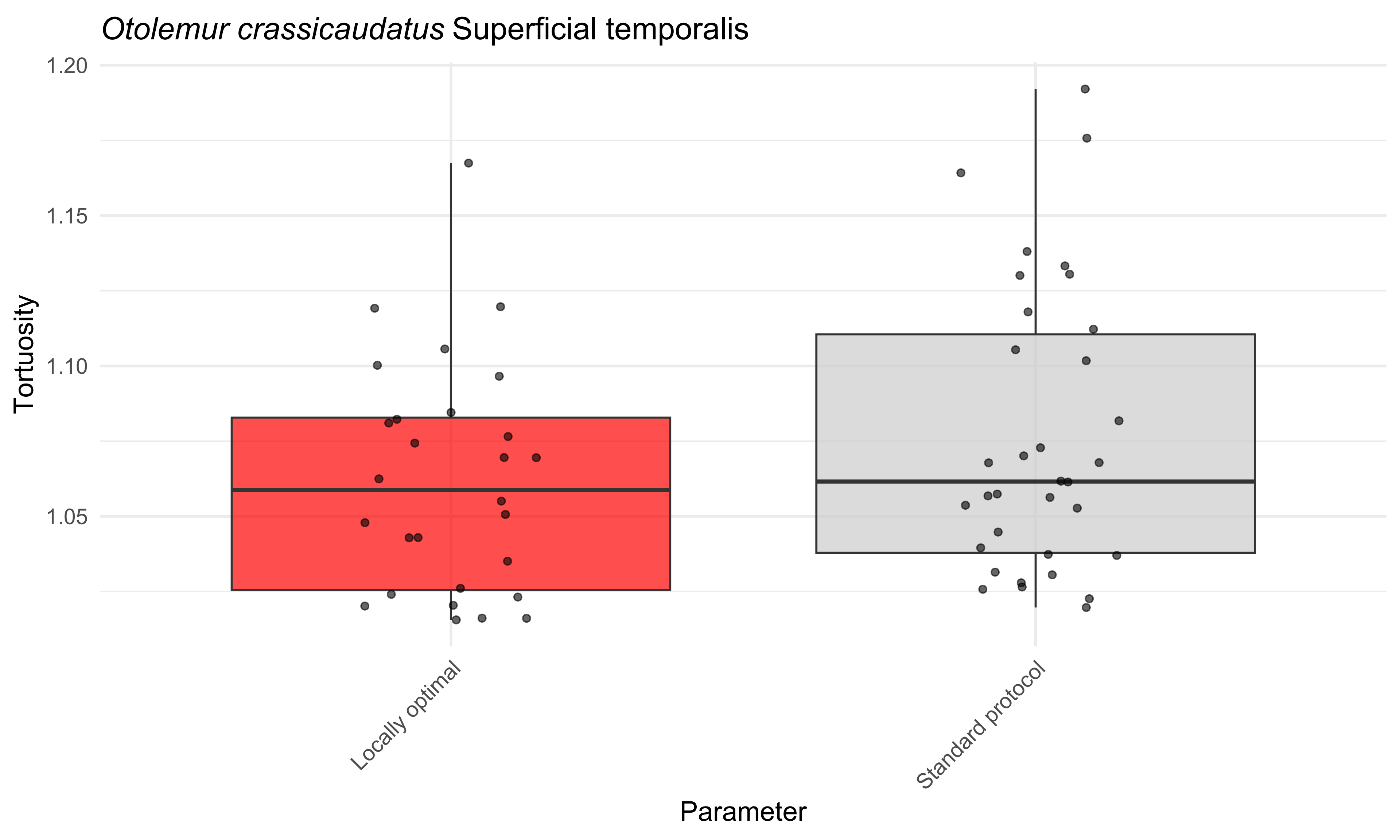
Otolemur crassicaudatus Superficial temporalis
1.20
1.15
1.10
1.05
Tortuosity
Locally optimal
Standard protocol
Parameter

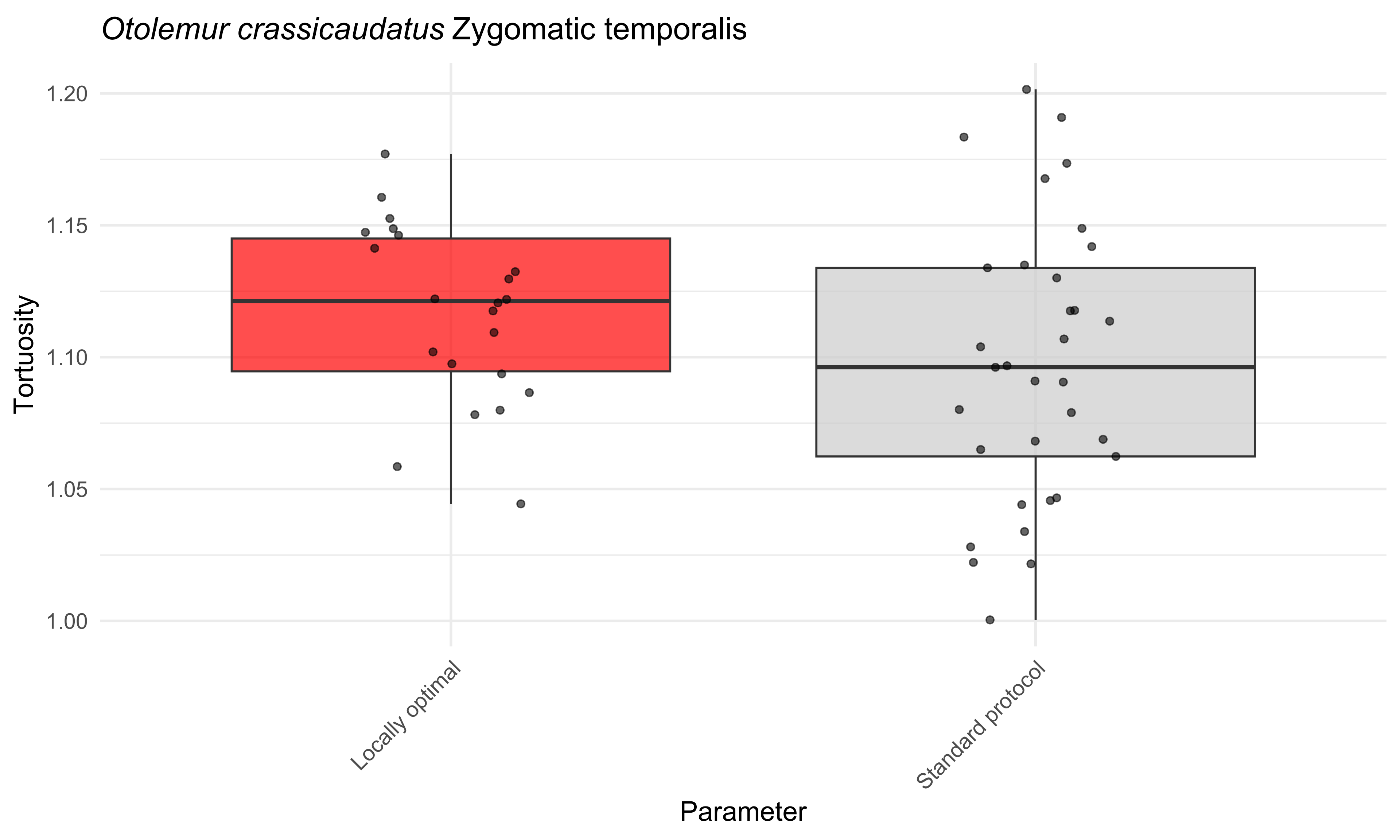

Otolemur crassicaudatus Zygomatic temporalis
Tortuosity
1.20
1.15
1.10
1.05
1.00
Locally optimal
Standard protocol
Parameter

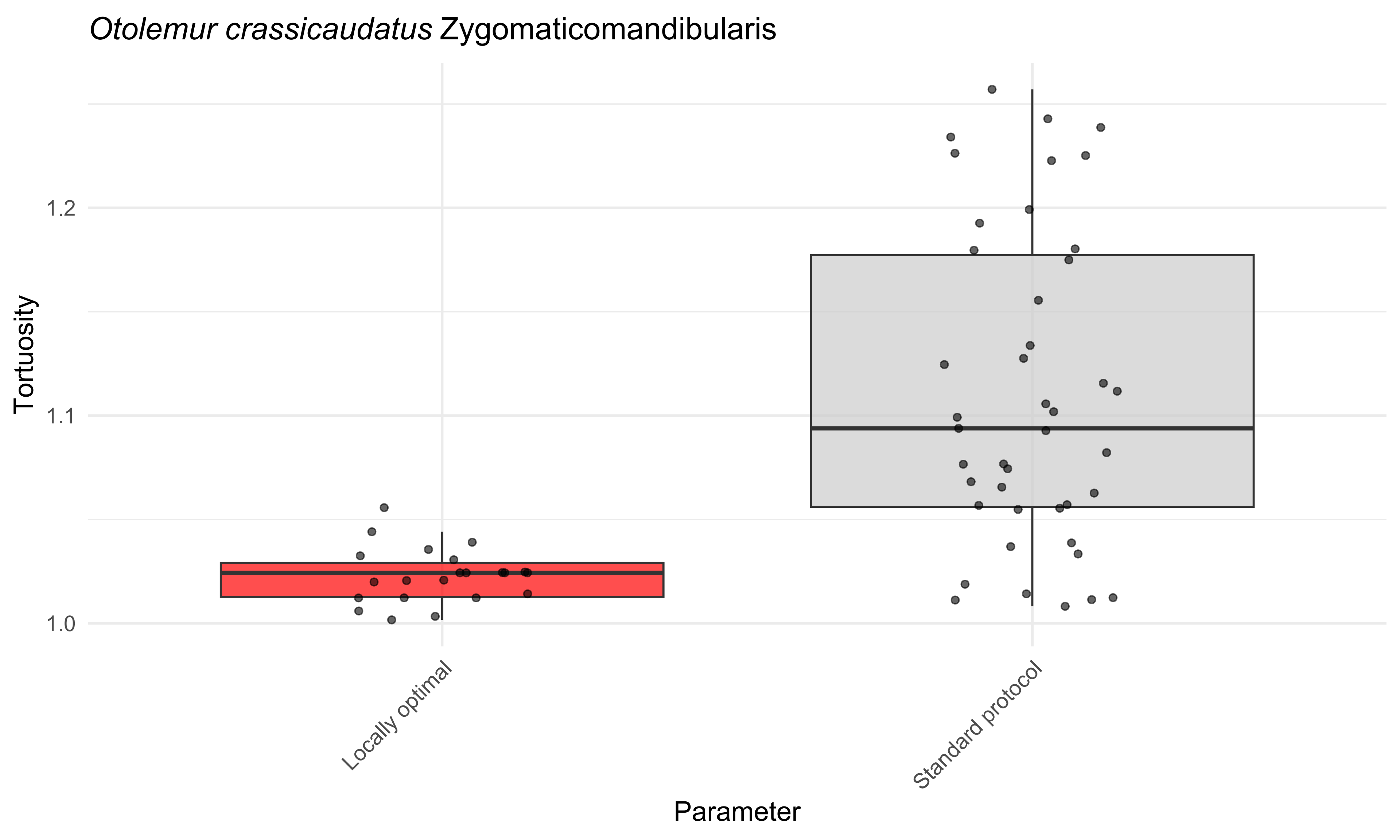

Otolemur crassicaudatus Zygomaticomandibularis
Tortuosity
1.2
1.1
1.0
Locally optimal
Standard protocol
Parameter

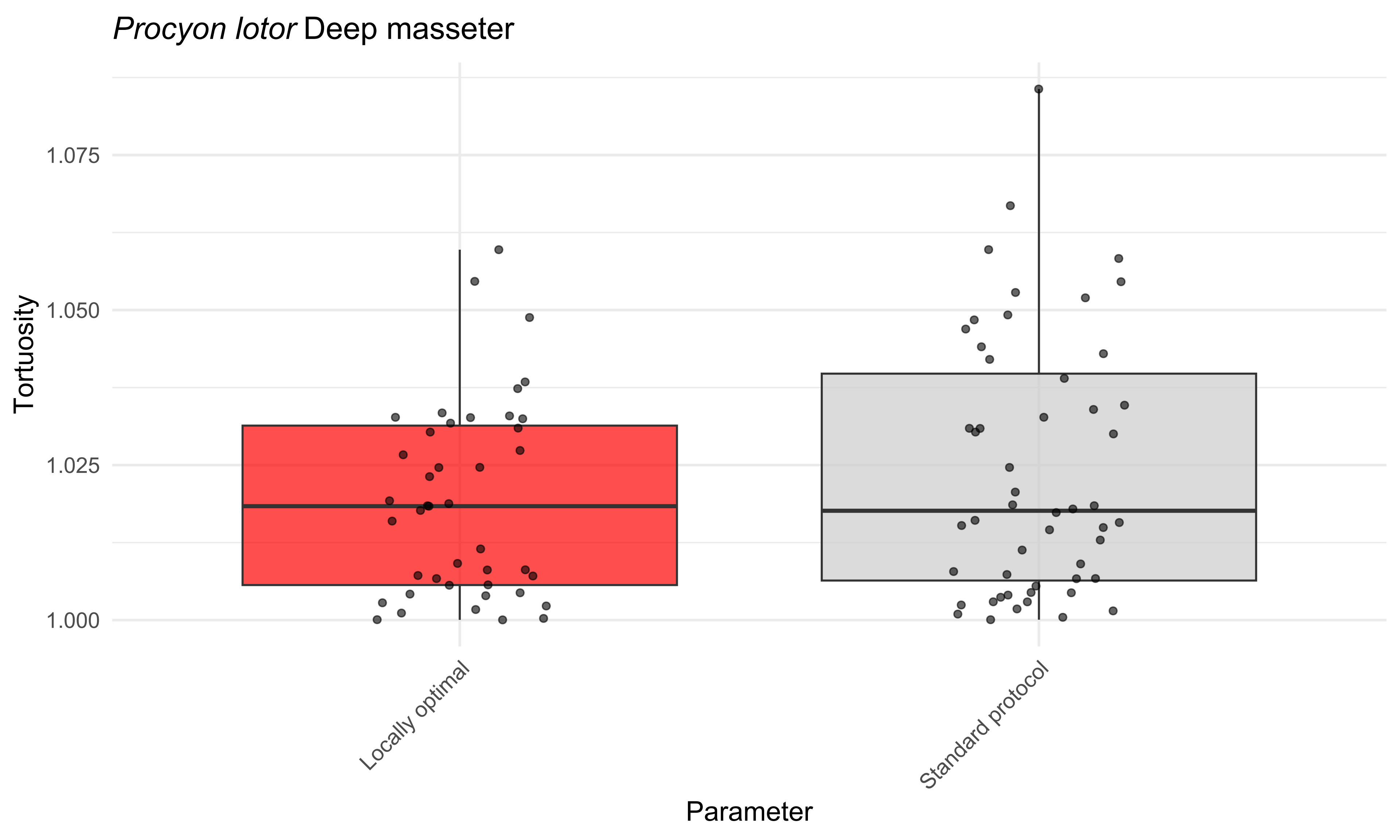

Procyon lotor Deep masseter
Tortuosity
1.075
1.050
1.025
1.000
Locally optimal
Standard protocol
Parameter

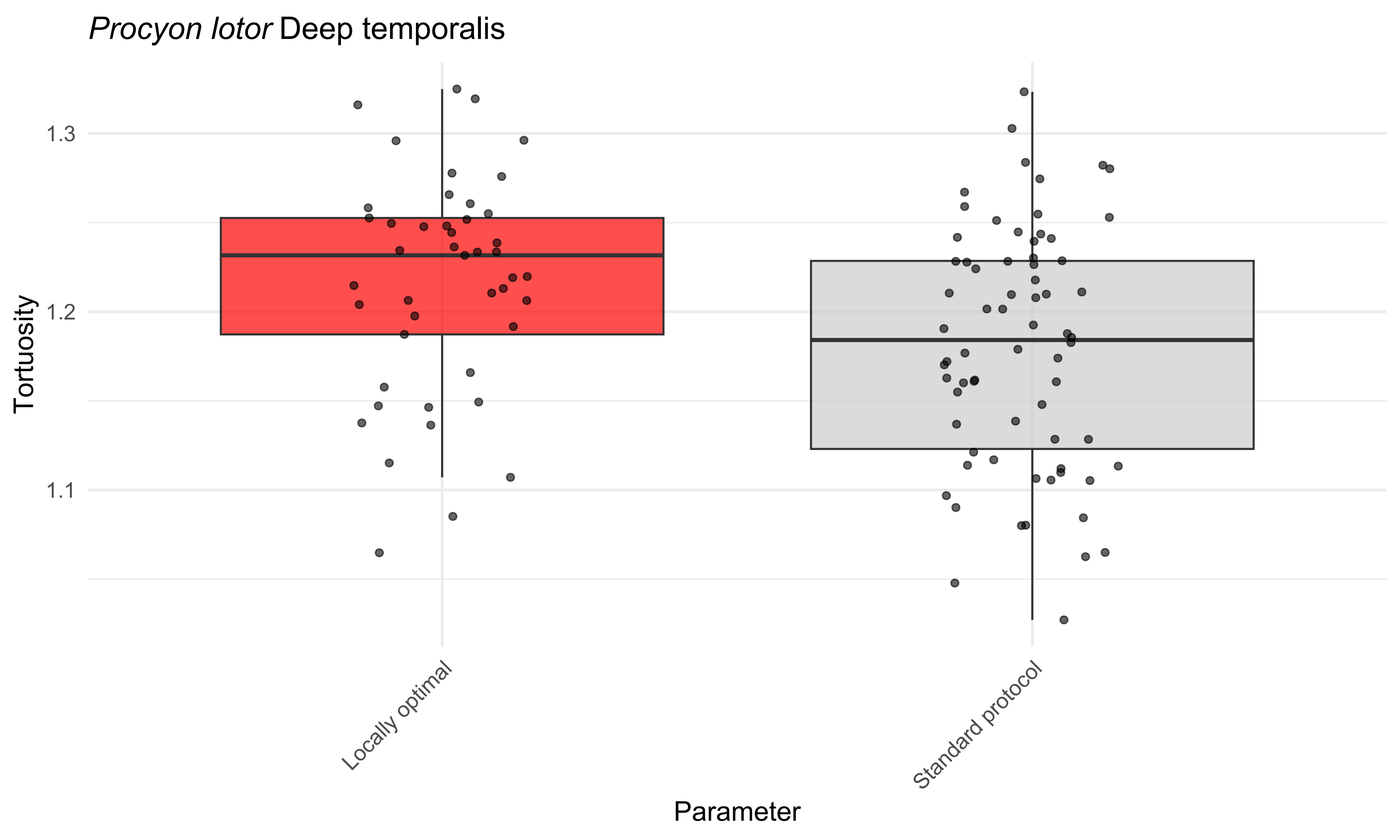

Procyon lotor Deep temporalis
Tortuosity
1.3
1.2
1.1
Locally optimal
Standard protocol
Parameter

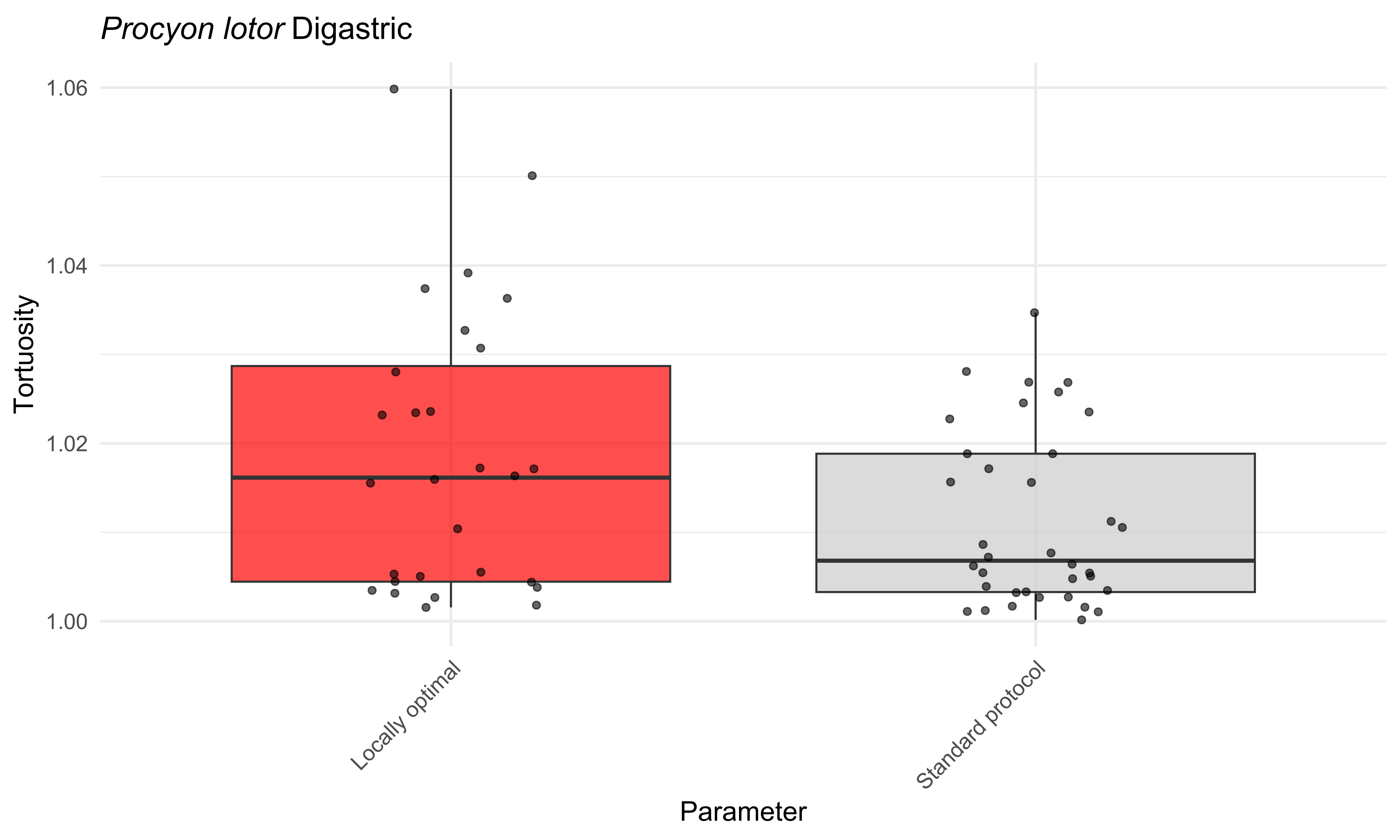

Procyon lotor Digastric
1.06
1.04
1.02
1.00
Tortuosity
Locally optimal
Standard protocol
Parameter

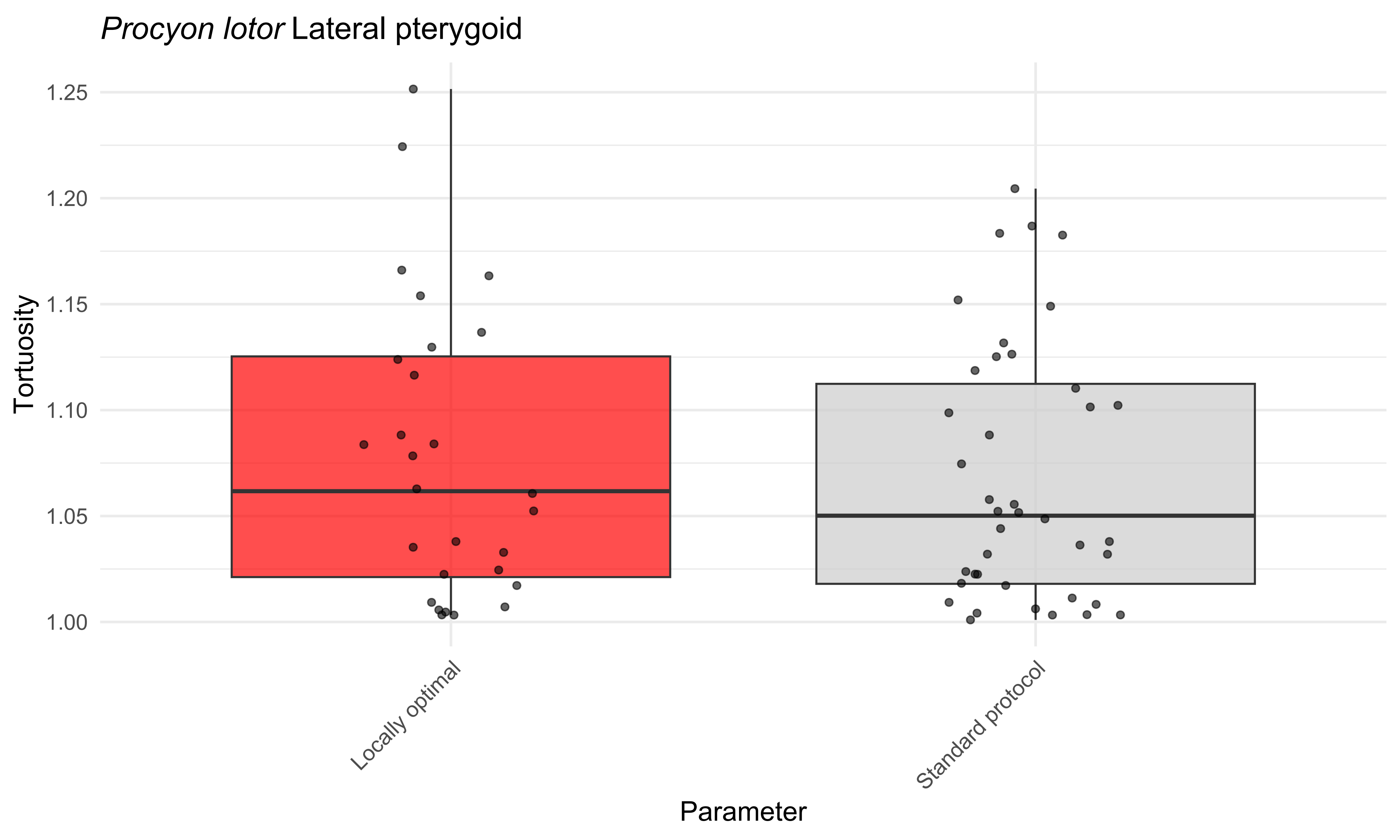

Procyon lotor Lateral pterygoid
Tortuosity
1.25
1.20
1.15
1.10
1.05
1.00
Locally optimal
Standard protocol
Parameter

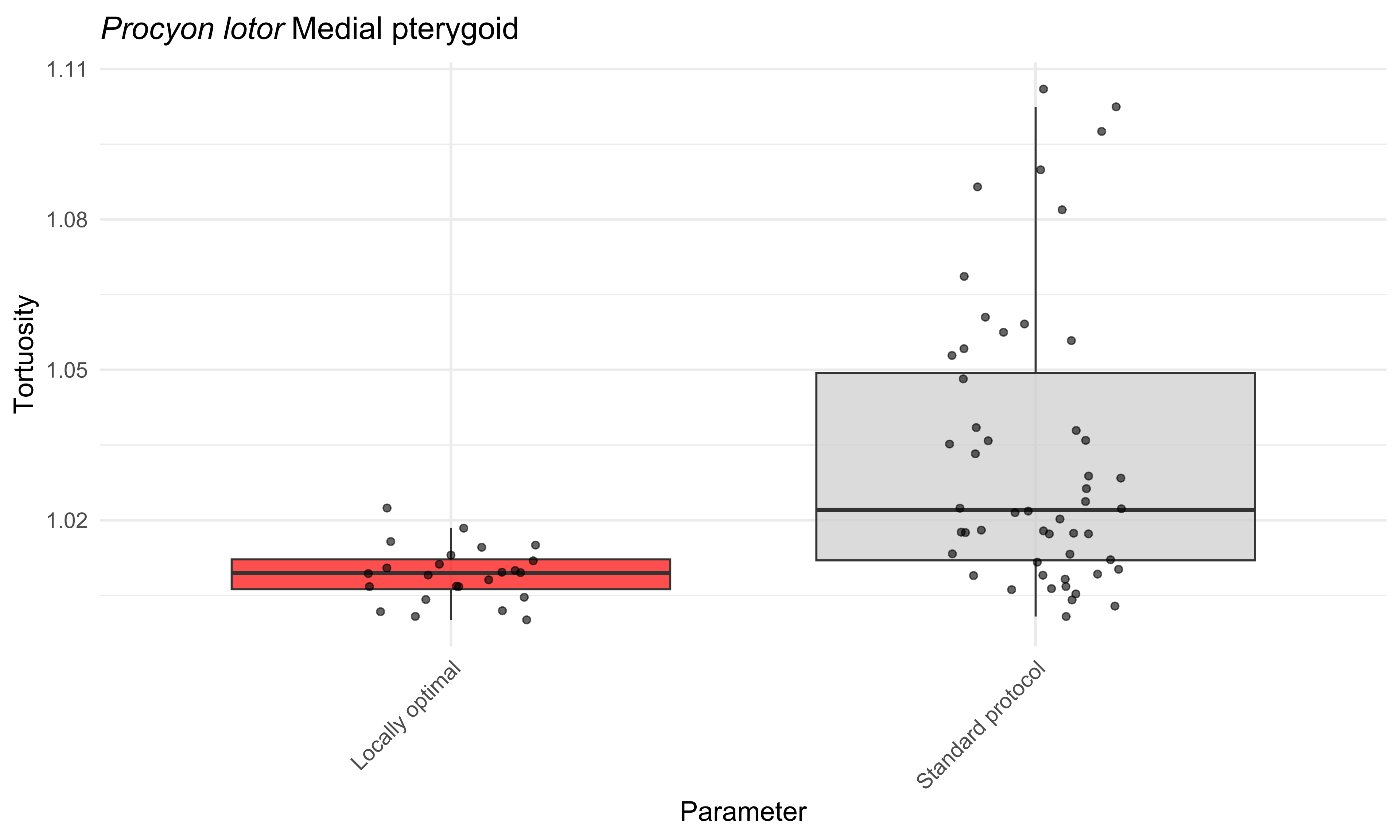

Procyon lotor Medial pterygoid
1.11
1.08
1.05
1.02
Tortuosity
Locally optimal
Standard protocol
Parameter

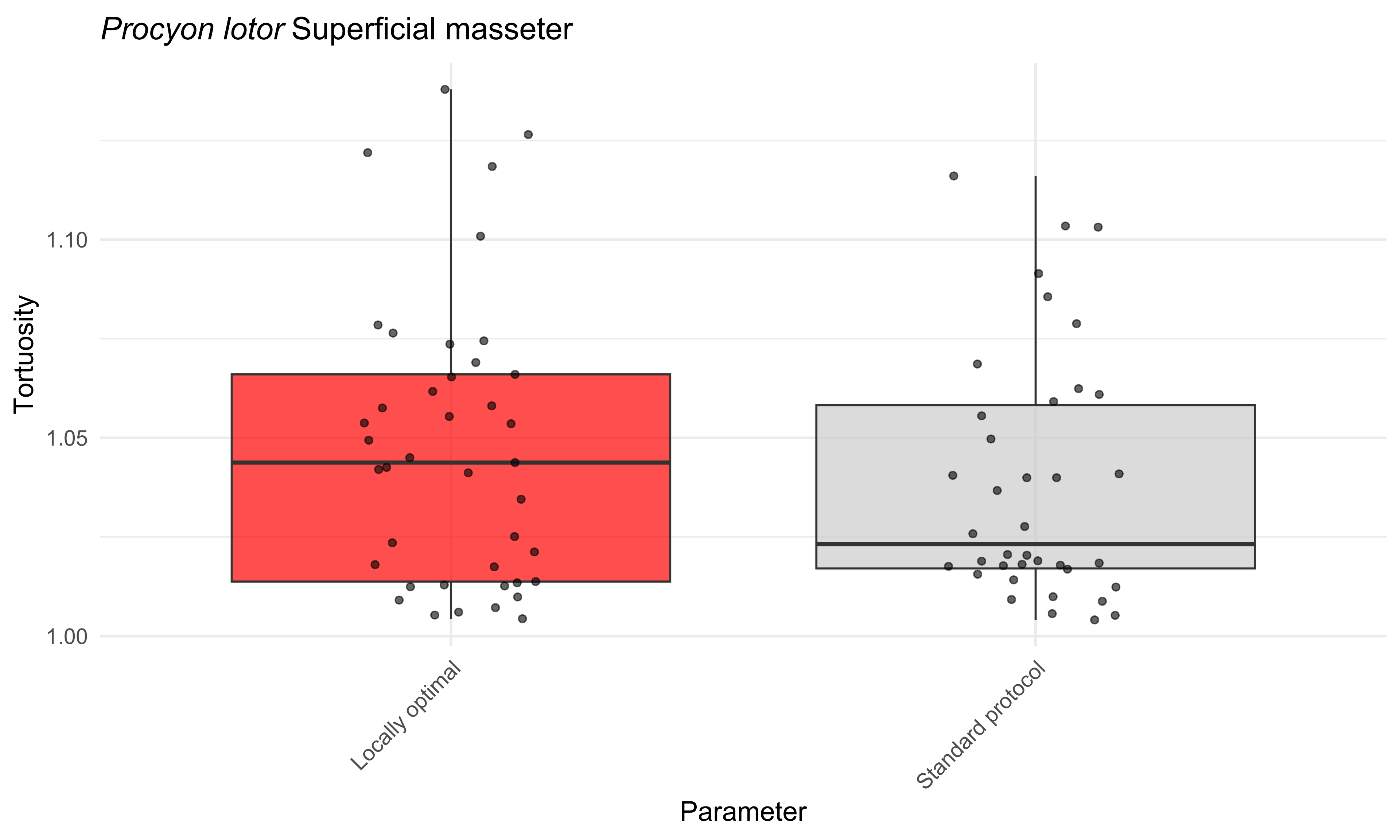

Procyon lotor Superficial masseter
Tortuosity
1.10
1.05
1.00
Locally optimal
Standard protocol
Parameter

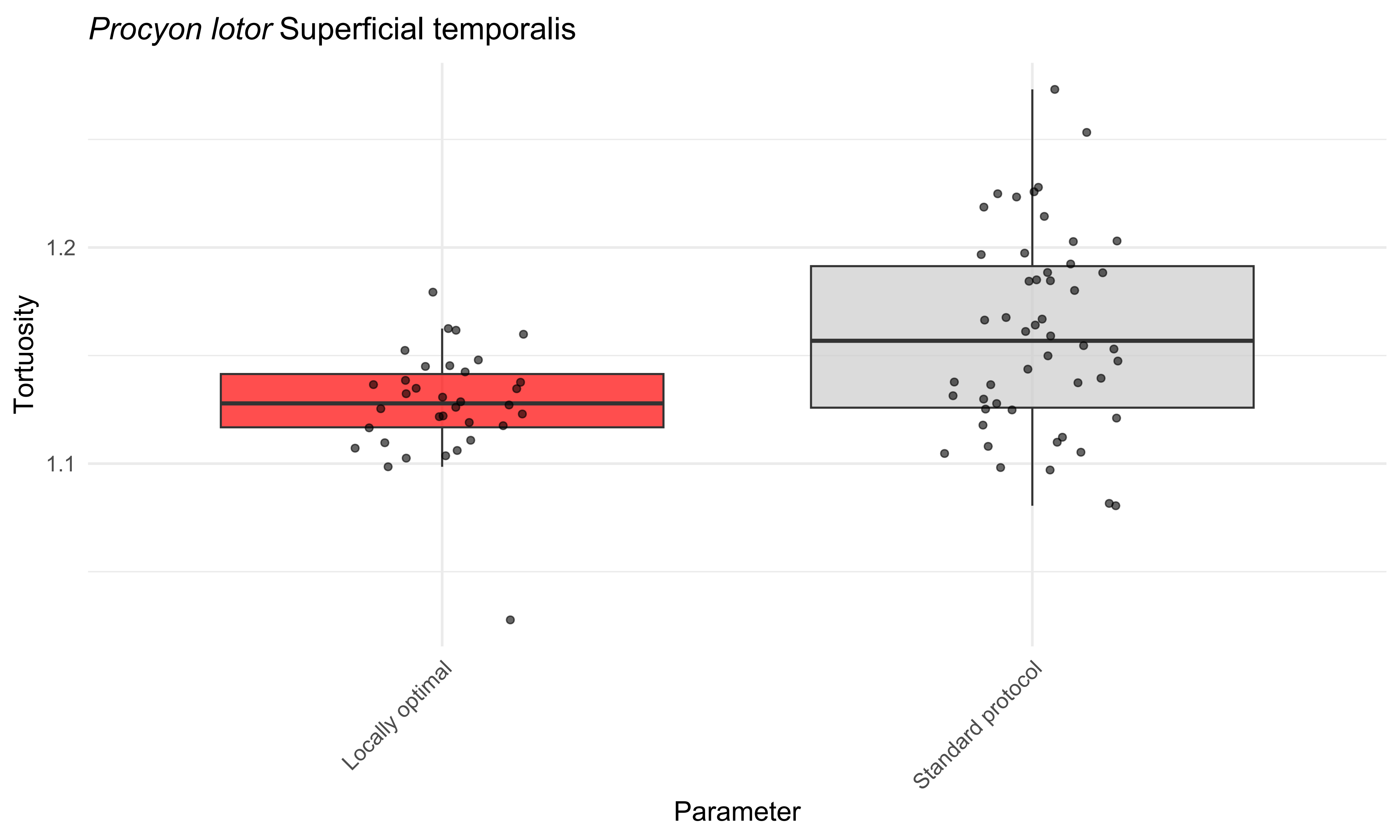

Procyon lotor Superficial temporalis
Tortuosity
1.2
1.1
Locally optimal
Standard protocol
Parameter

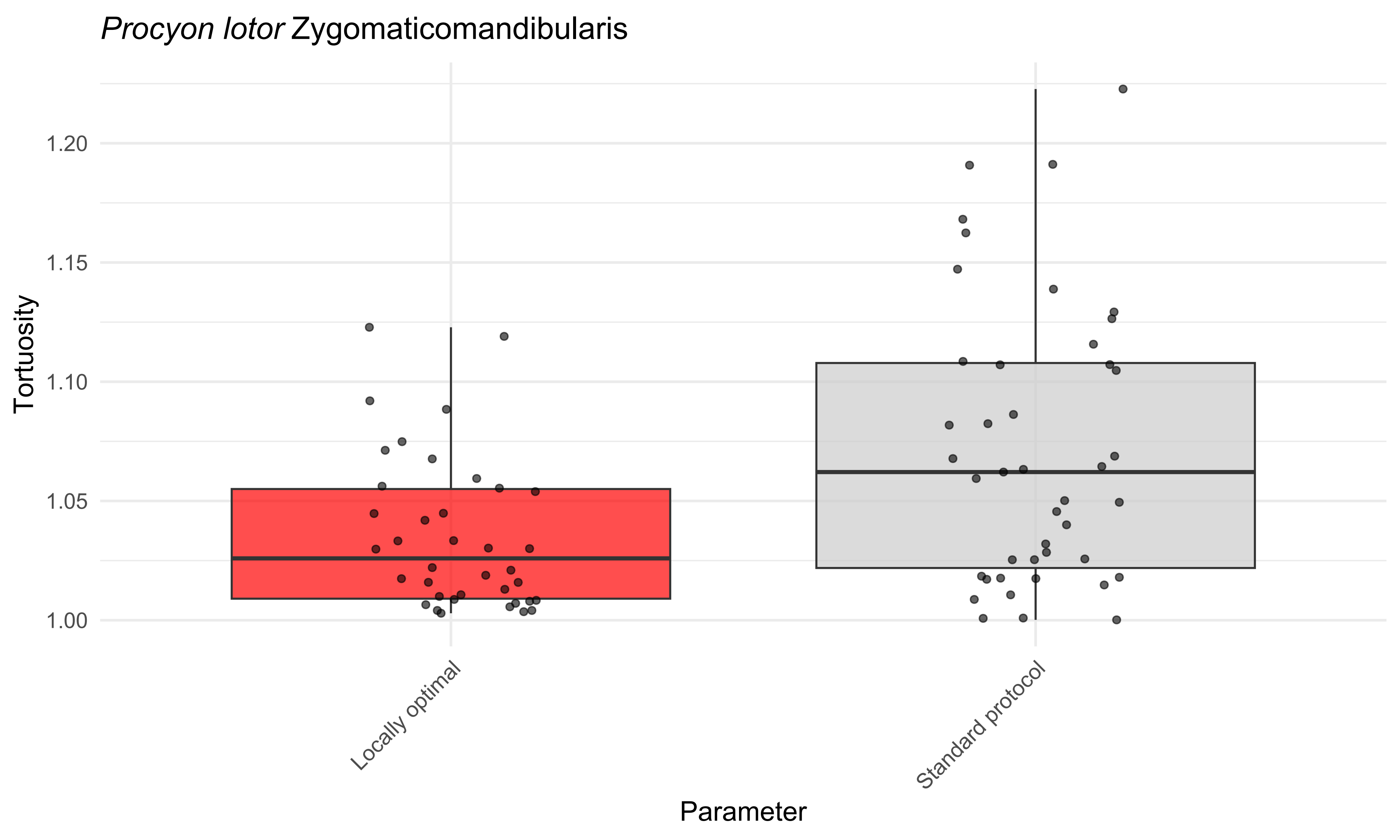

Procyon lotor Zygomaticomandibularis
Tortuosity
1.20
1.15
1.10
1.05
1.00
Locally optimal
Standard protocol
Parameter
