# Supplementary material for: Multi‐method analysis for the three‐dimensional reconstruction of muscle fascicles from DiceCT datasets
Source: Anat Rec (Hoboken). 2025 Dec 4;309(12):3491–510. doi: 10.1002/ar.70088 (PMC13749328; doi:10.1002/ar.70088)
Supplement: Supplementary file 5 [file AR-309-3491-s008.pdf]

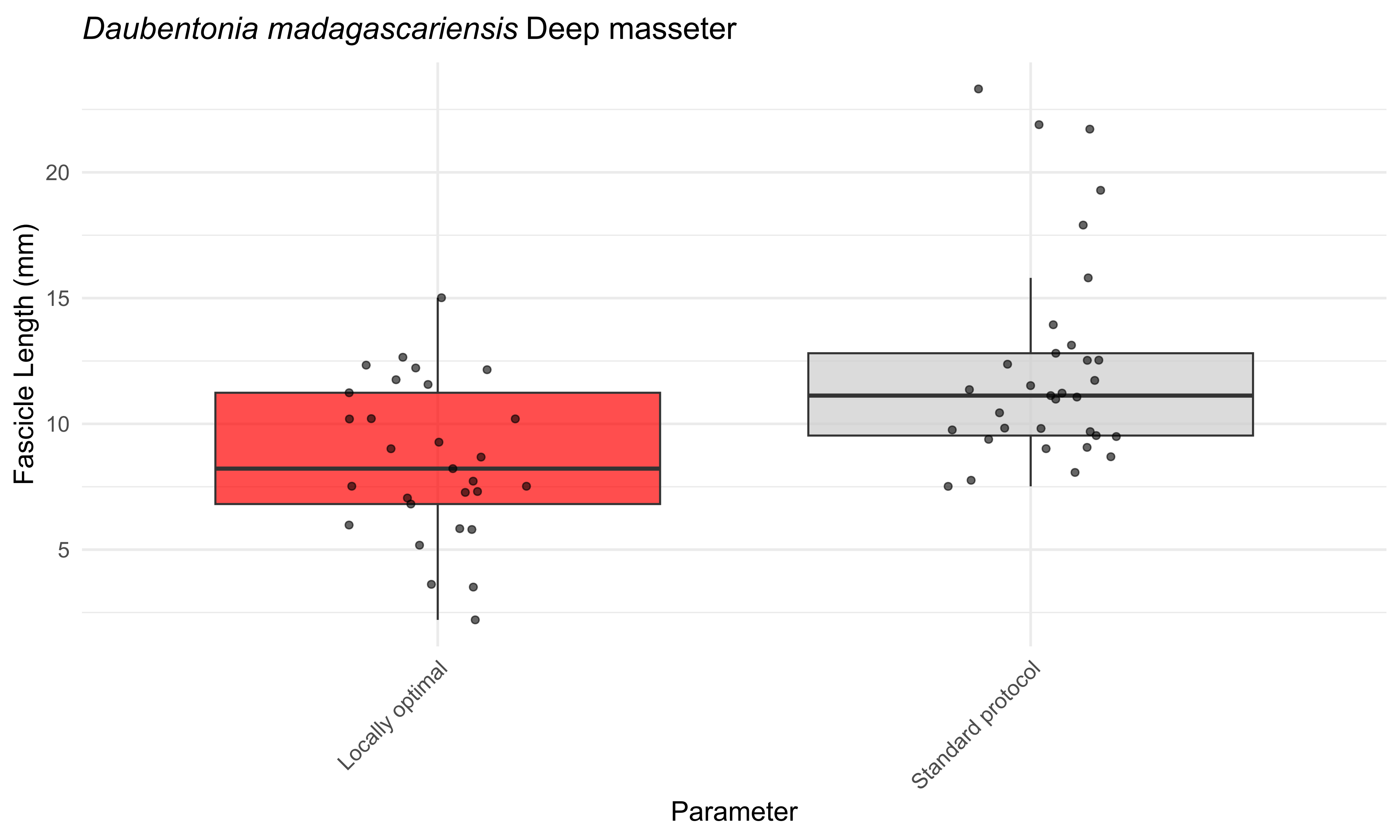

Daubentonia madagascariensis Deep masseter
Fascicle Length (mm)
20
15
10
5
Locally optimal
Standard protocol
Parameter

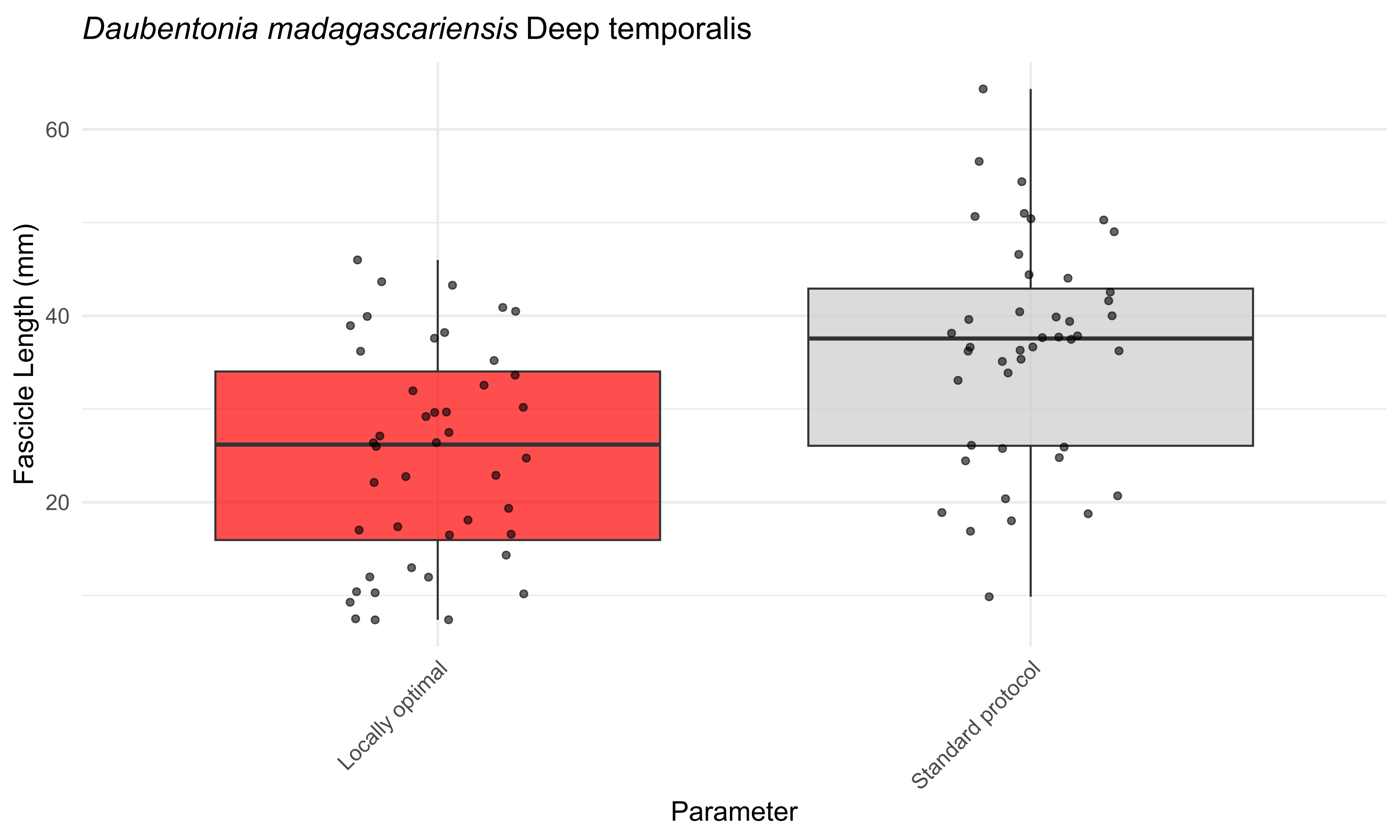
Daubentonia madagascariensis Deep temporalis
Fascicle Length (mm)
60
40
20
Locally optimal
Standard protocol
Parameter

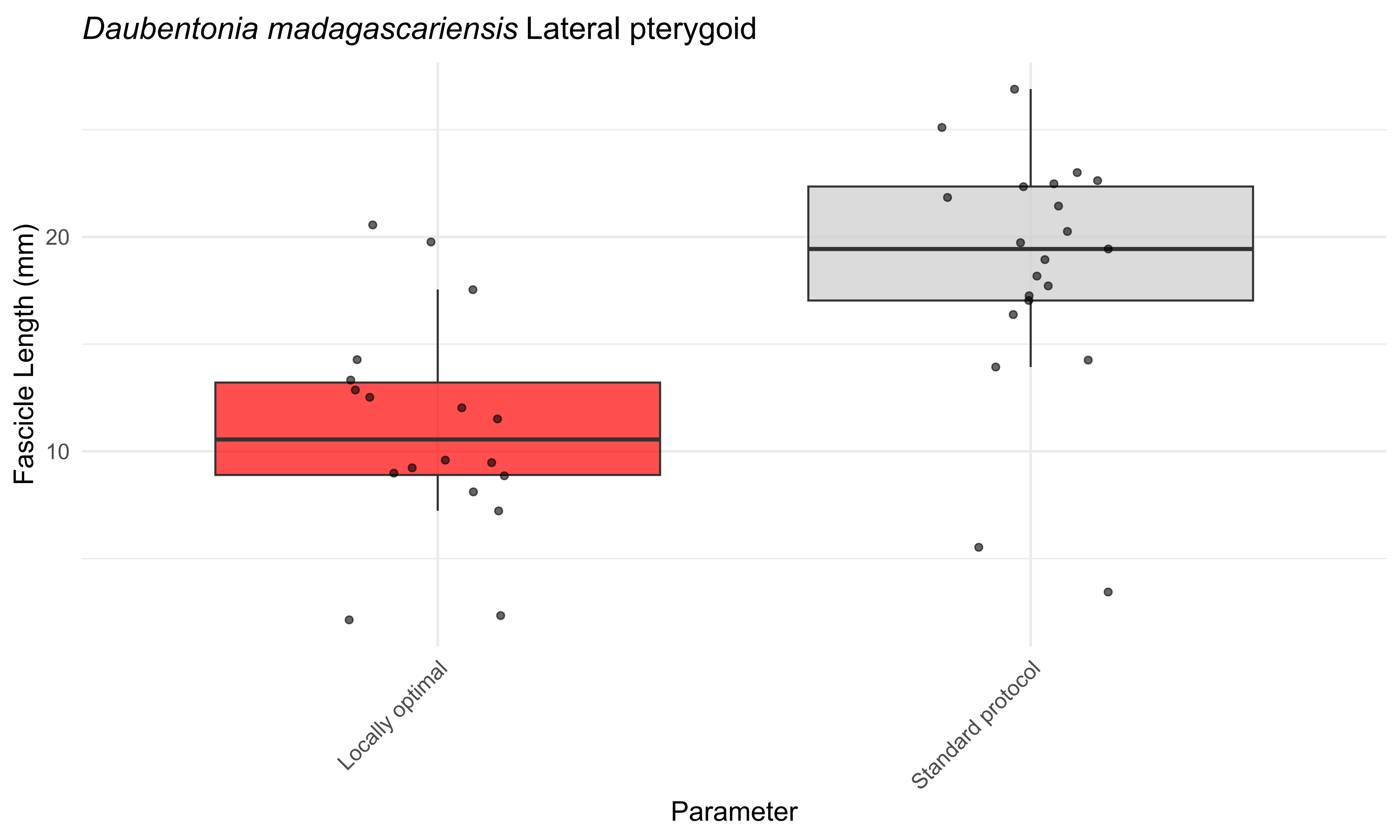

Daubentonia madagascariensis Lateral pterygoid
Fascicle Length (mm)
20
10
Locally optimal
Standard protocol
Parameter

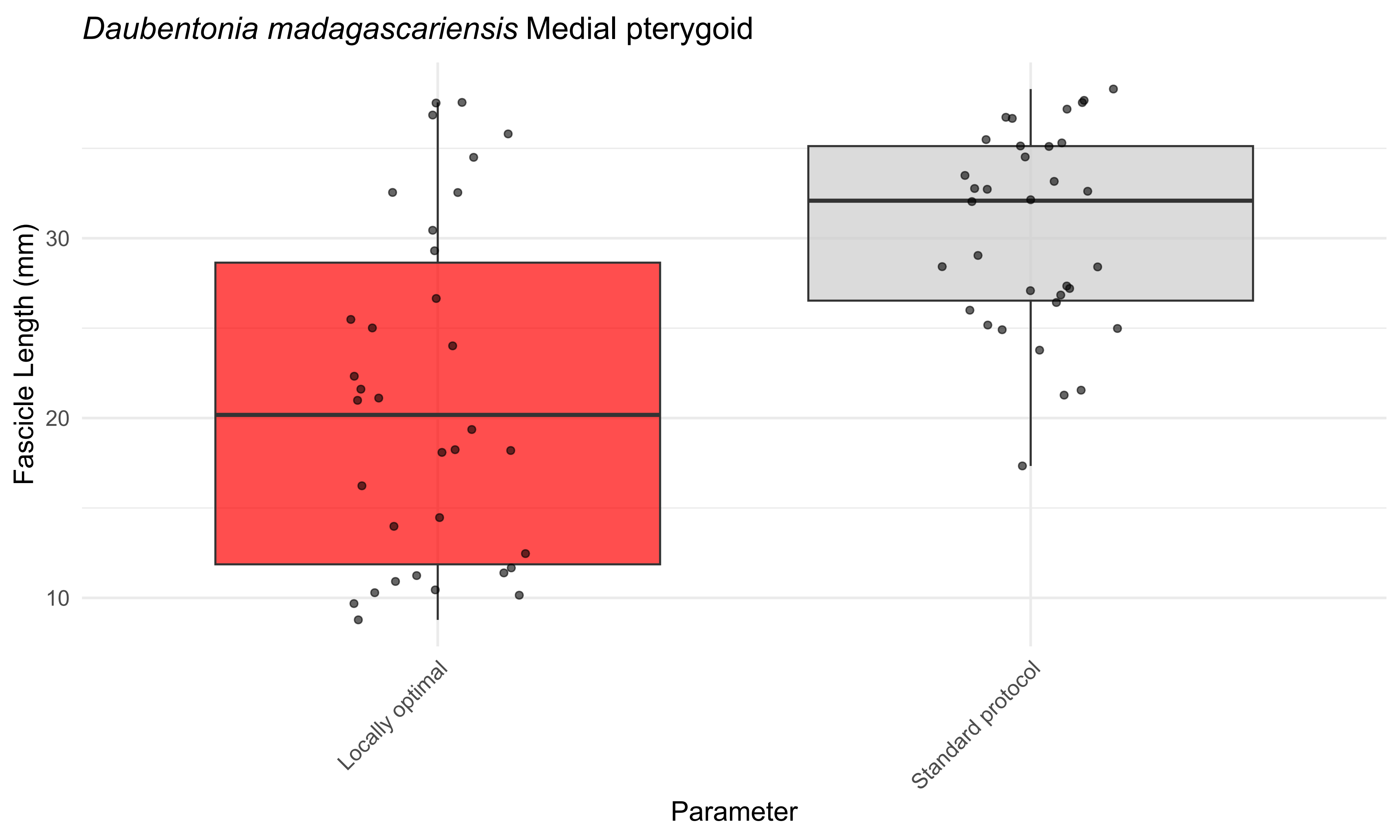

Daubentonia madagascariensis Medial pterygoid
Fascicle Length (mm)
30
20
10
Locally optimal
Standard protocol
Parameter

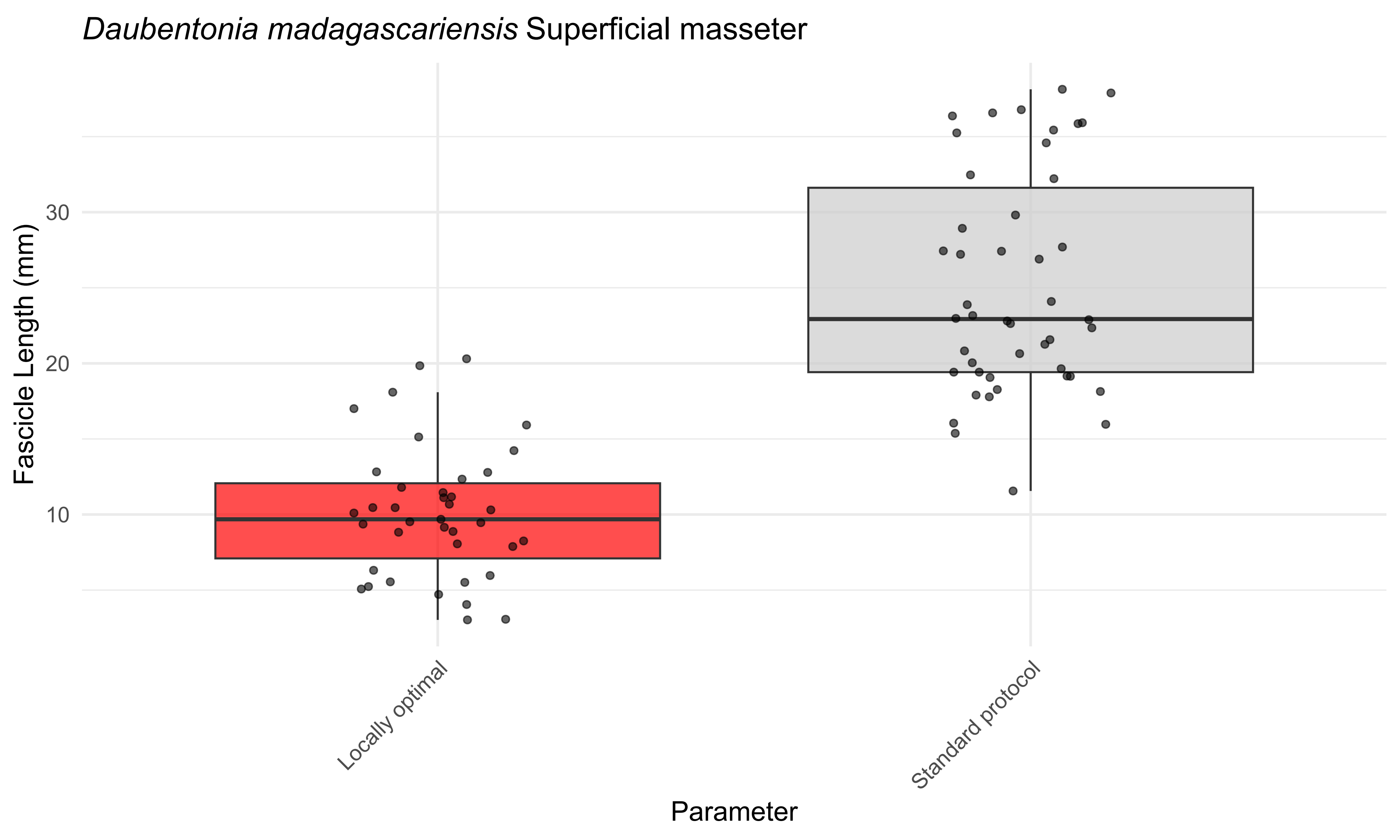

Daubentonia madagascariensis Superficial masseter
Fascicle Length (mm)
30
20
10
Locally optimal
Standard protocol
Parameter

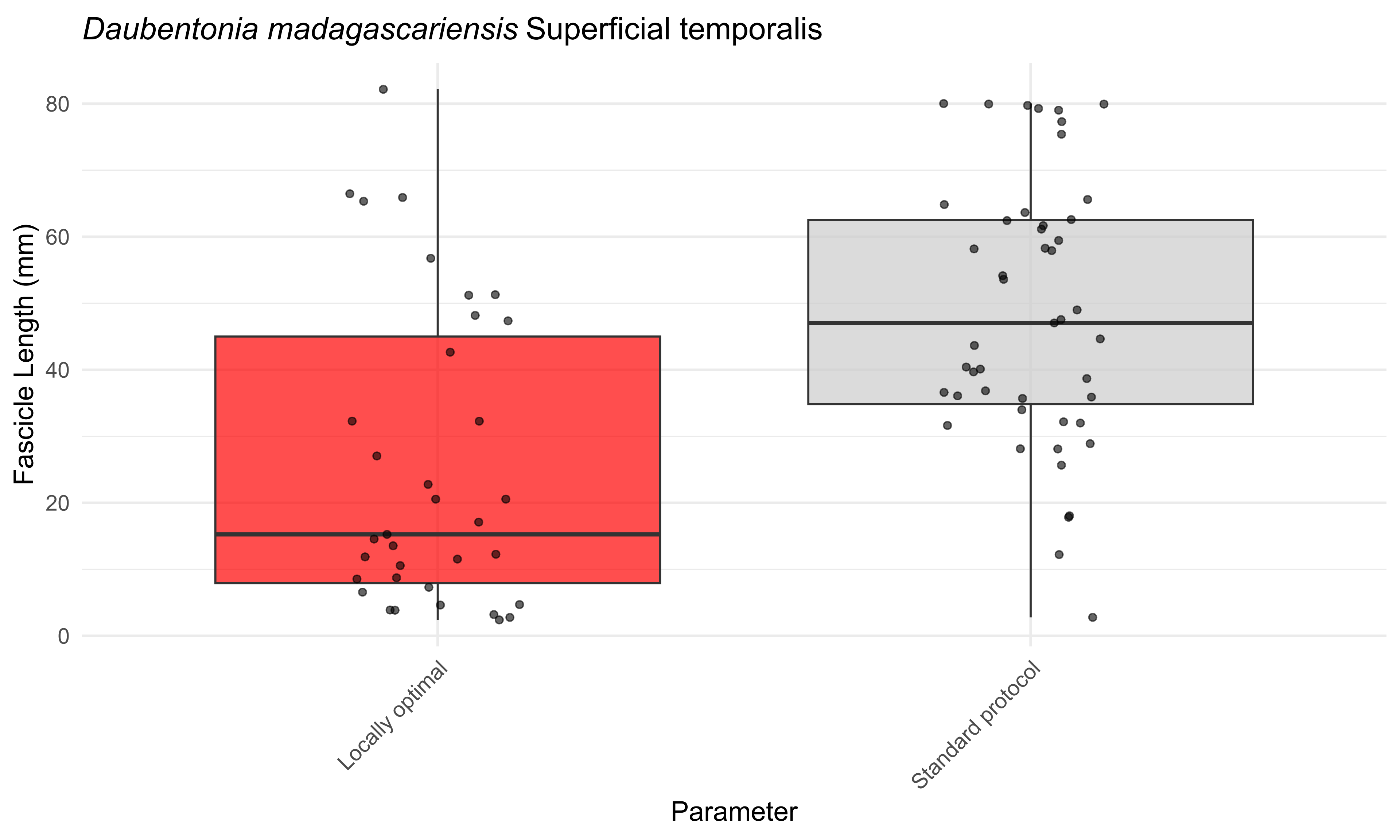

Daubentonia madagascariensis Superficial temporalis
Fascicle Length (mm)
0
20
40
60
80
Locally optimal
Standard protocol
Parameter

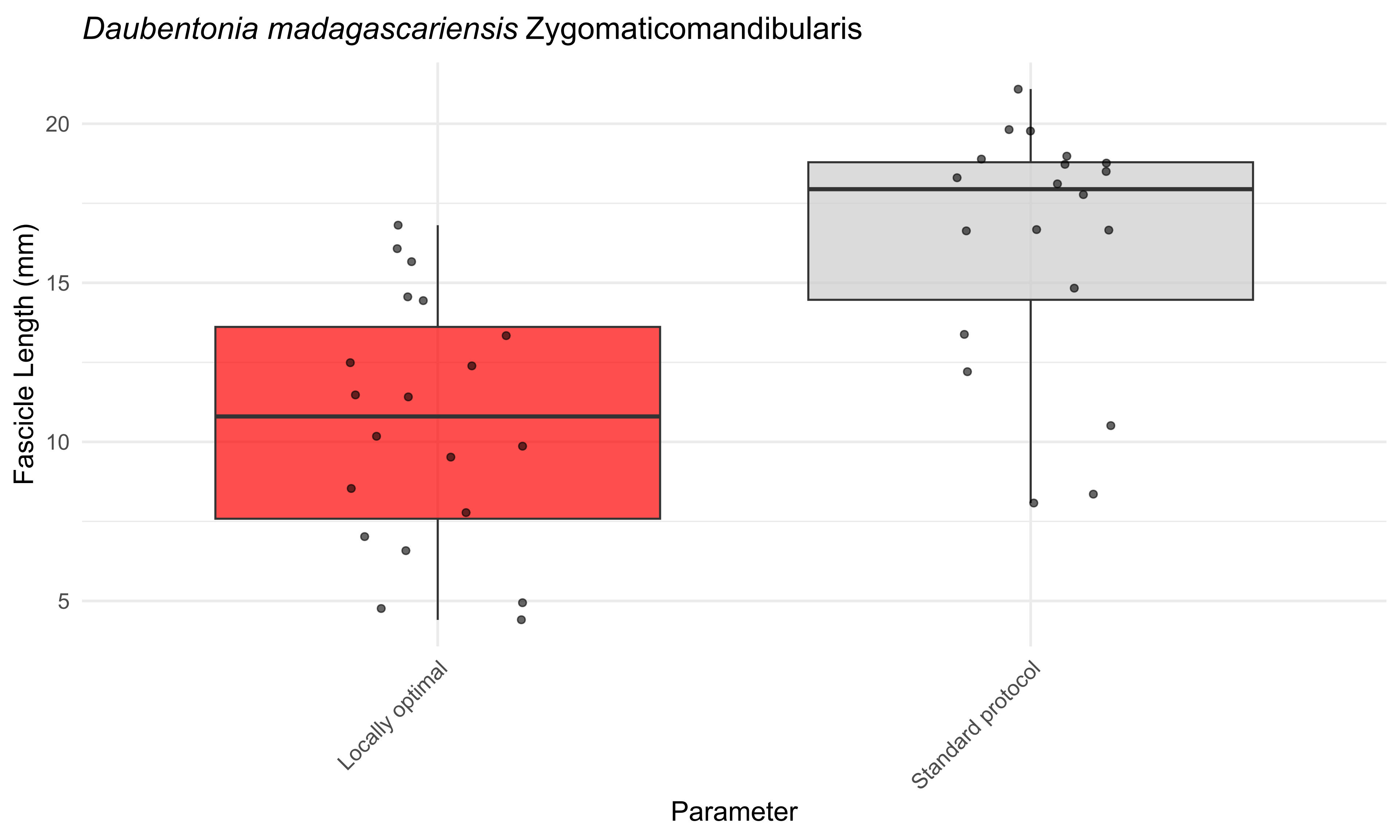

Daubentonia madagascariensis Zygomaticomandibularis
Fascicle Length (mm)
20
15
10
5
Locally optimal
Standard protocol
Parameter

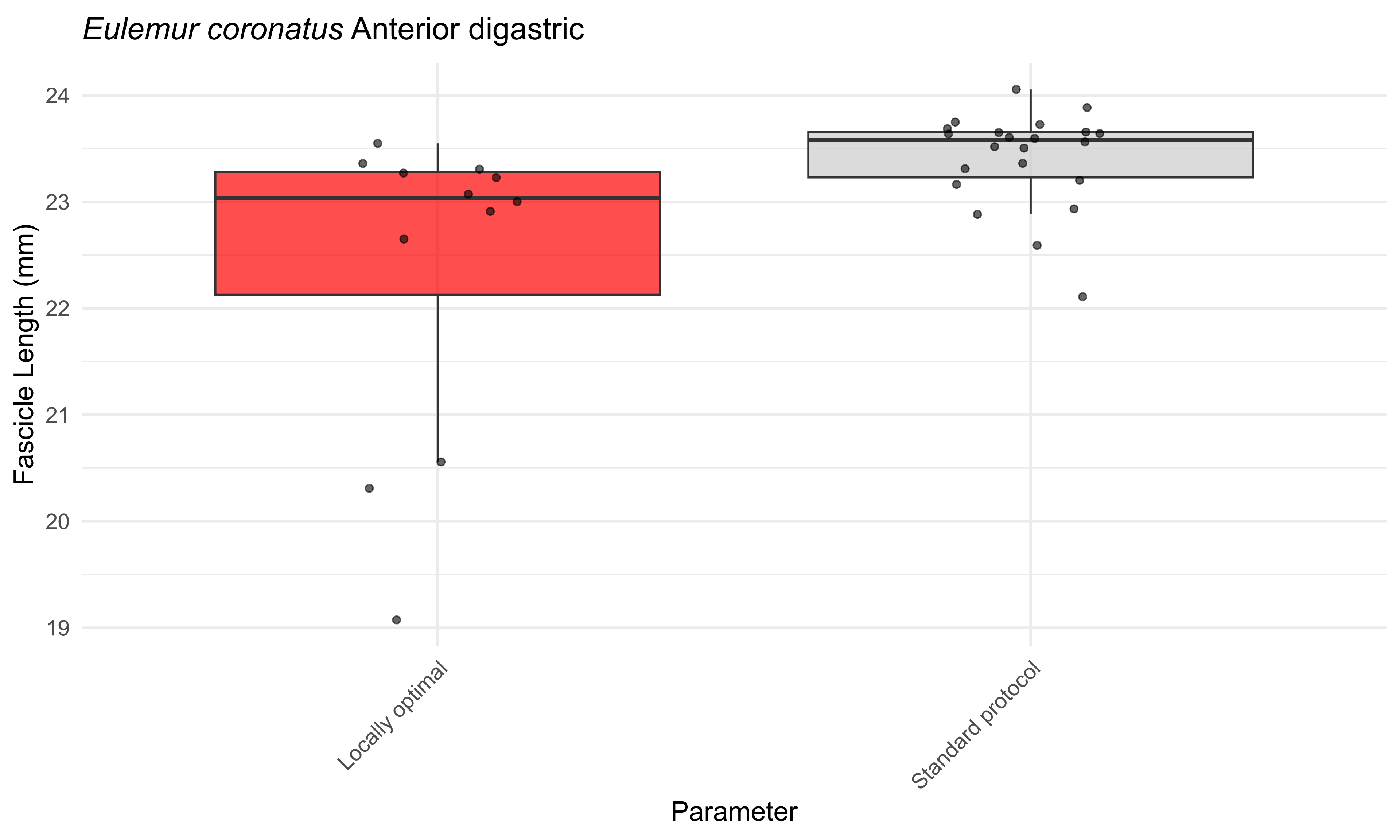
Eulemur coronatus Anterior digastric
Fascicle Length (mm)
24
23
22
21
20
19
Locally optimal
Standard protocol
Parameter

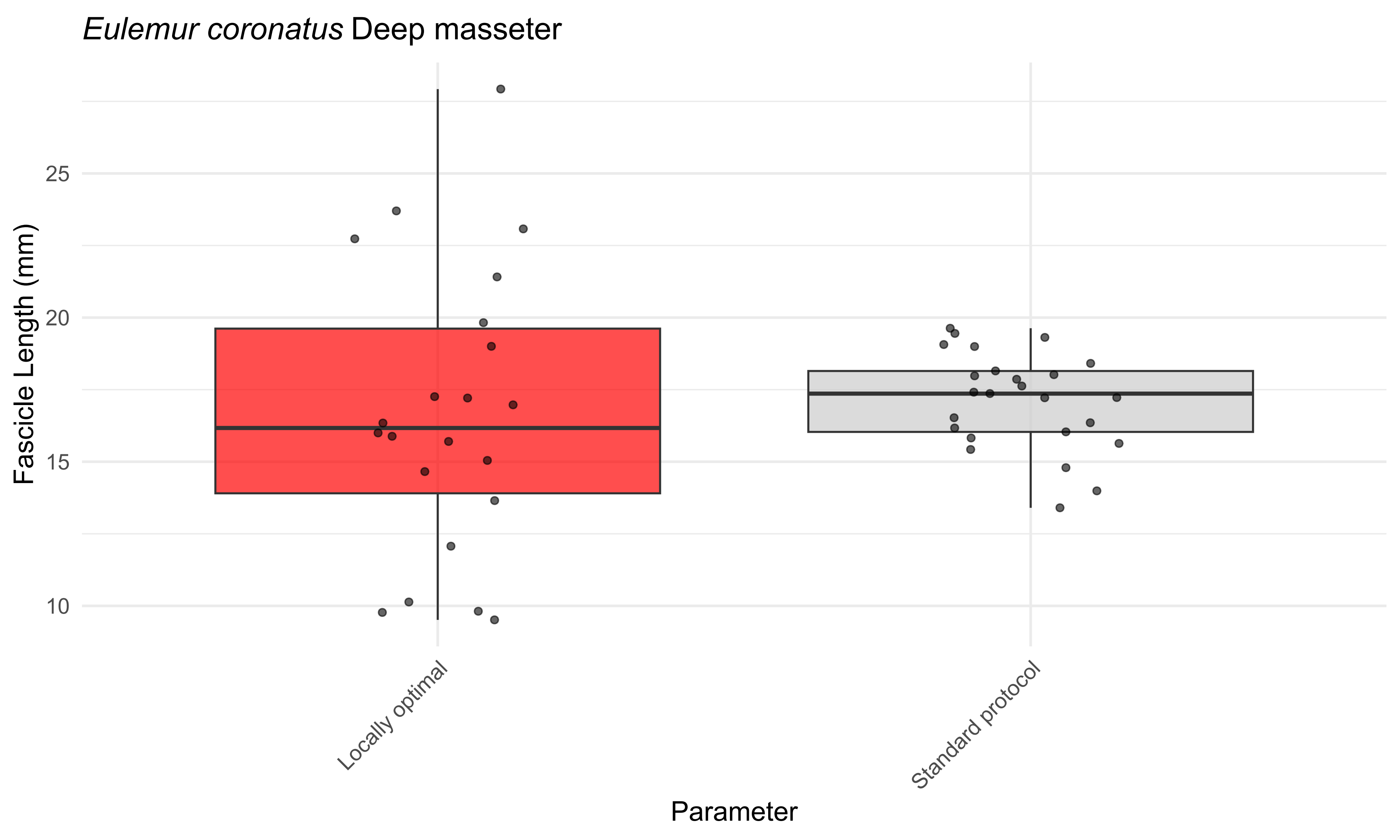
Eulemur coronatus Deep masseter
Fascicle Length (mm)
25
20
15
10
Locally optimal
Standard protocol
Parameter

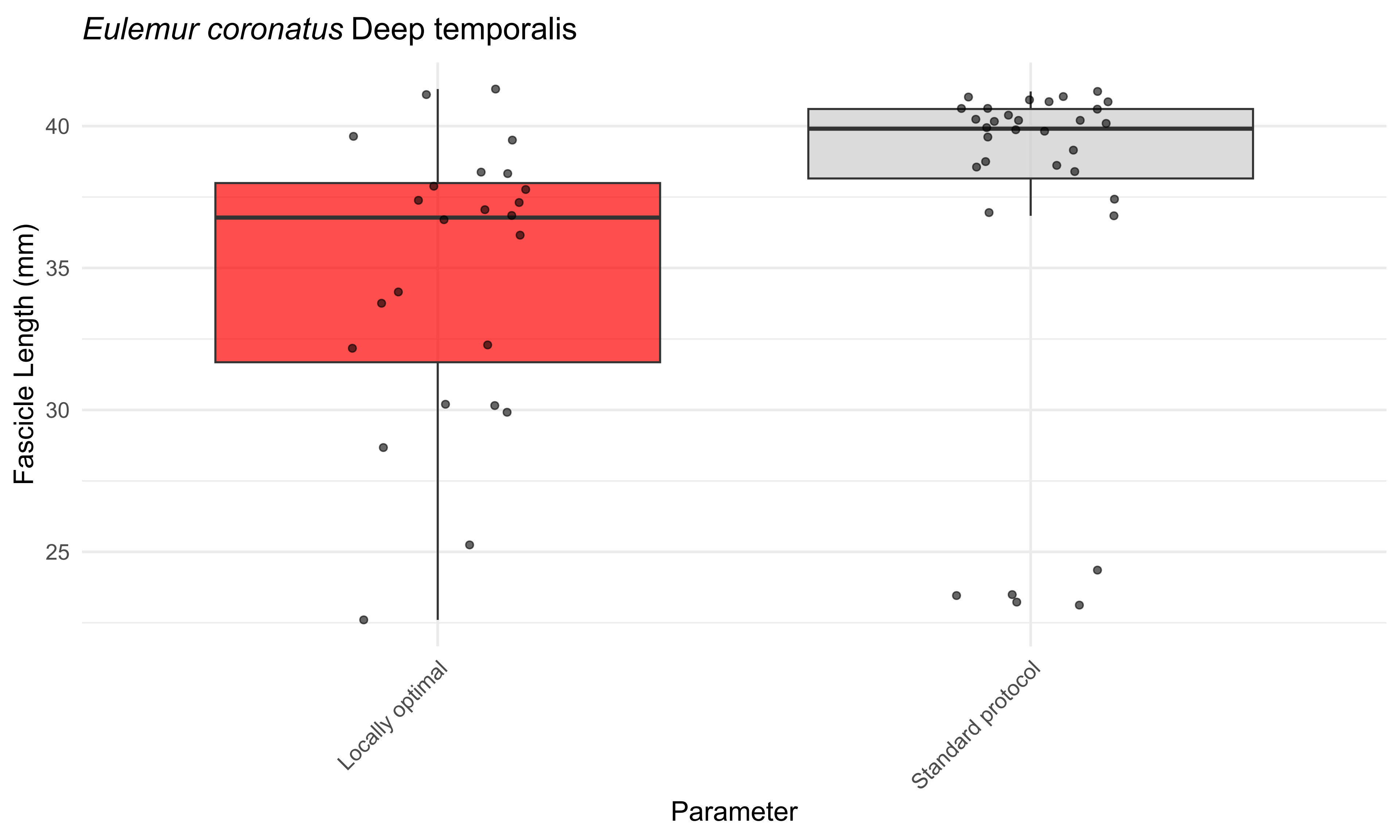

Eulemur coronatus Deep temporalis
Fascicle Length (mm)
40
35
30
25
Locally optimal
Standard protocol
Parameter

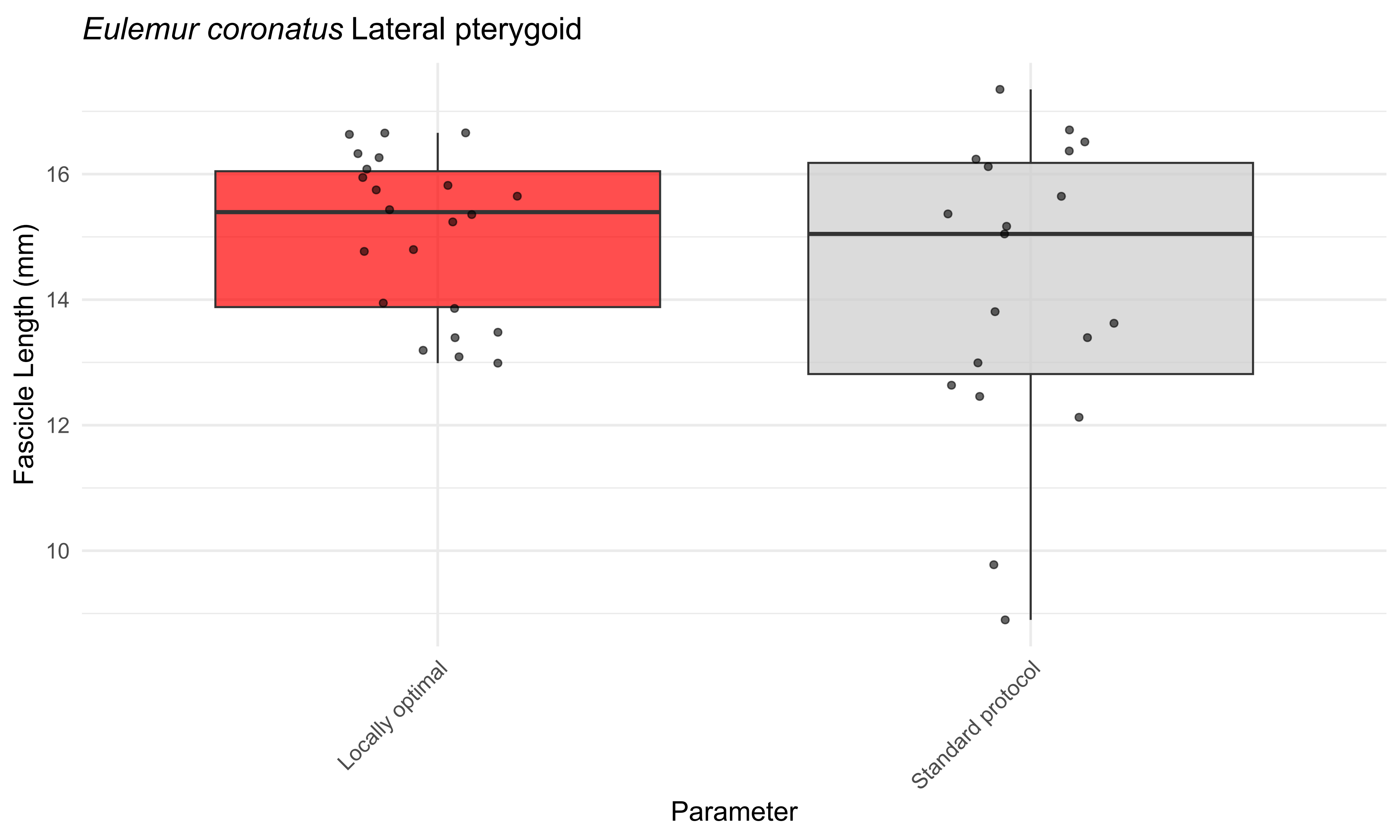

Eulemur coronatus Lateral pterygoid
Fascicle Length (mm)
16
14
12
10
Locally optimal
Standard protocol
Parameter

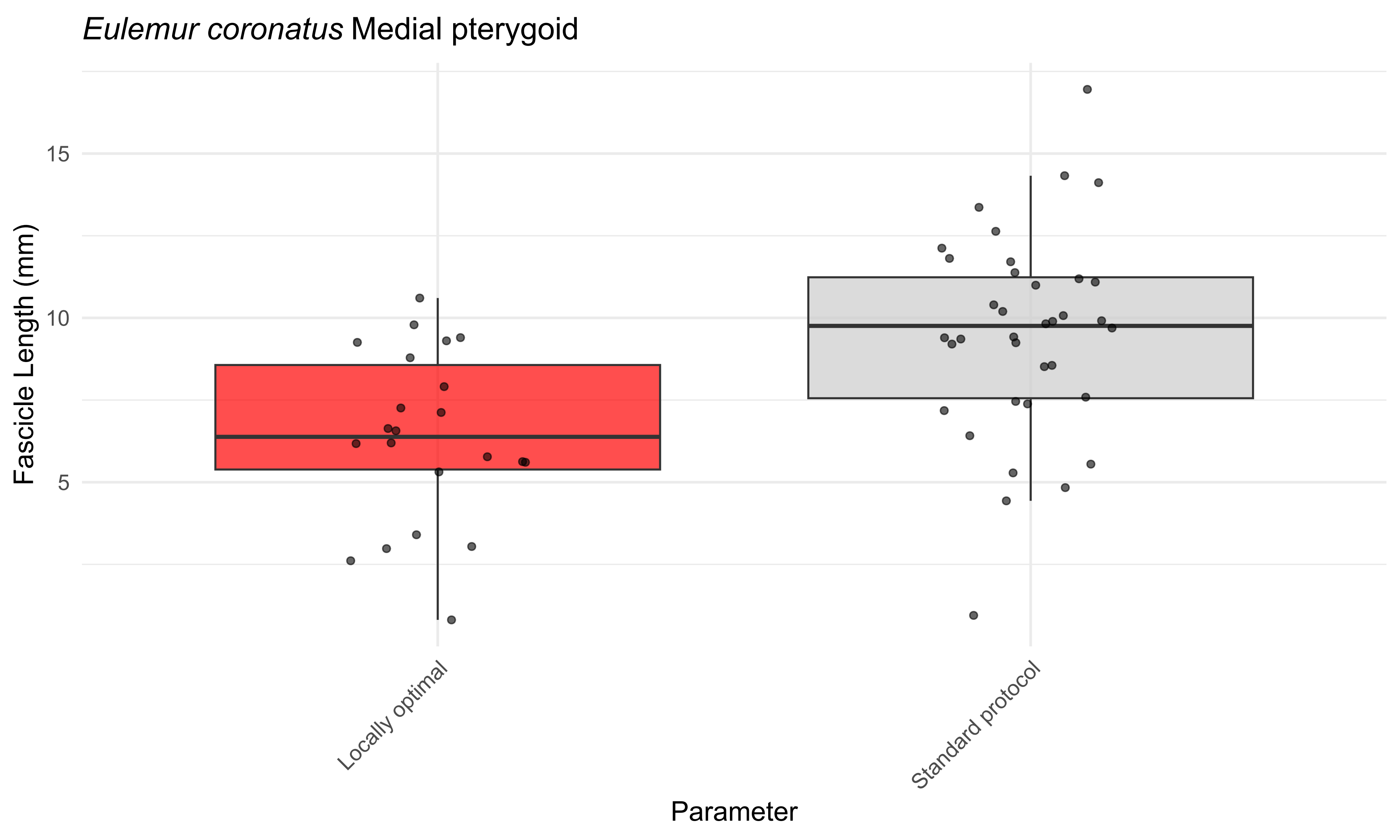

Eulemur coronatus Medial pterygoid
Fascicle Length (mm)
15
10
5
Locally optimal
Standard protocol
Parameter

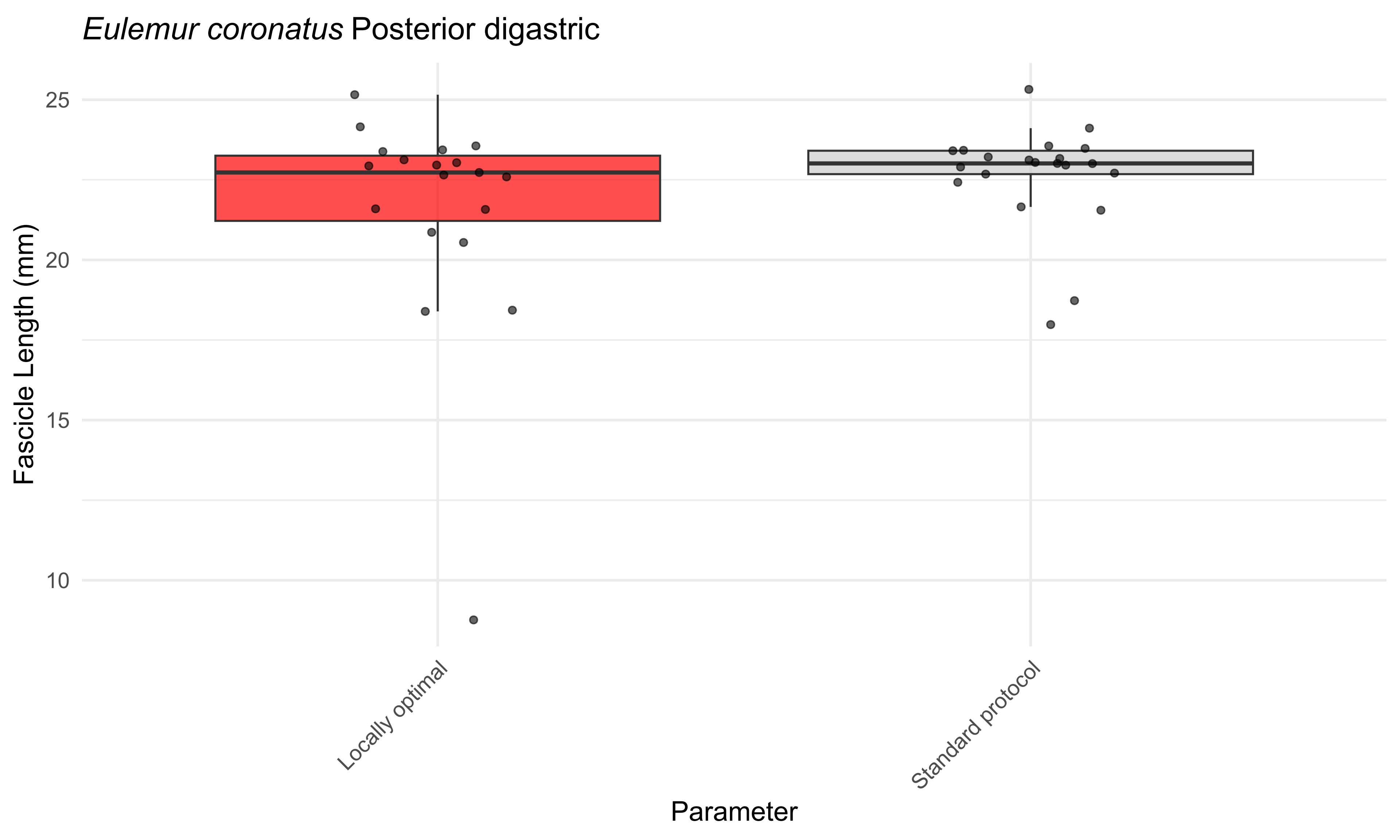
Eulemur coronatus Posterior digastric
Fascicle Length (mm)
25
20
15
10
Locally optimal
Standard protocol
Parameter

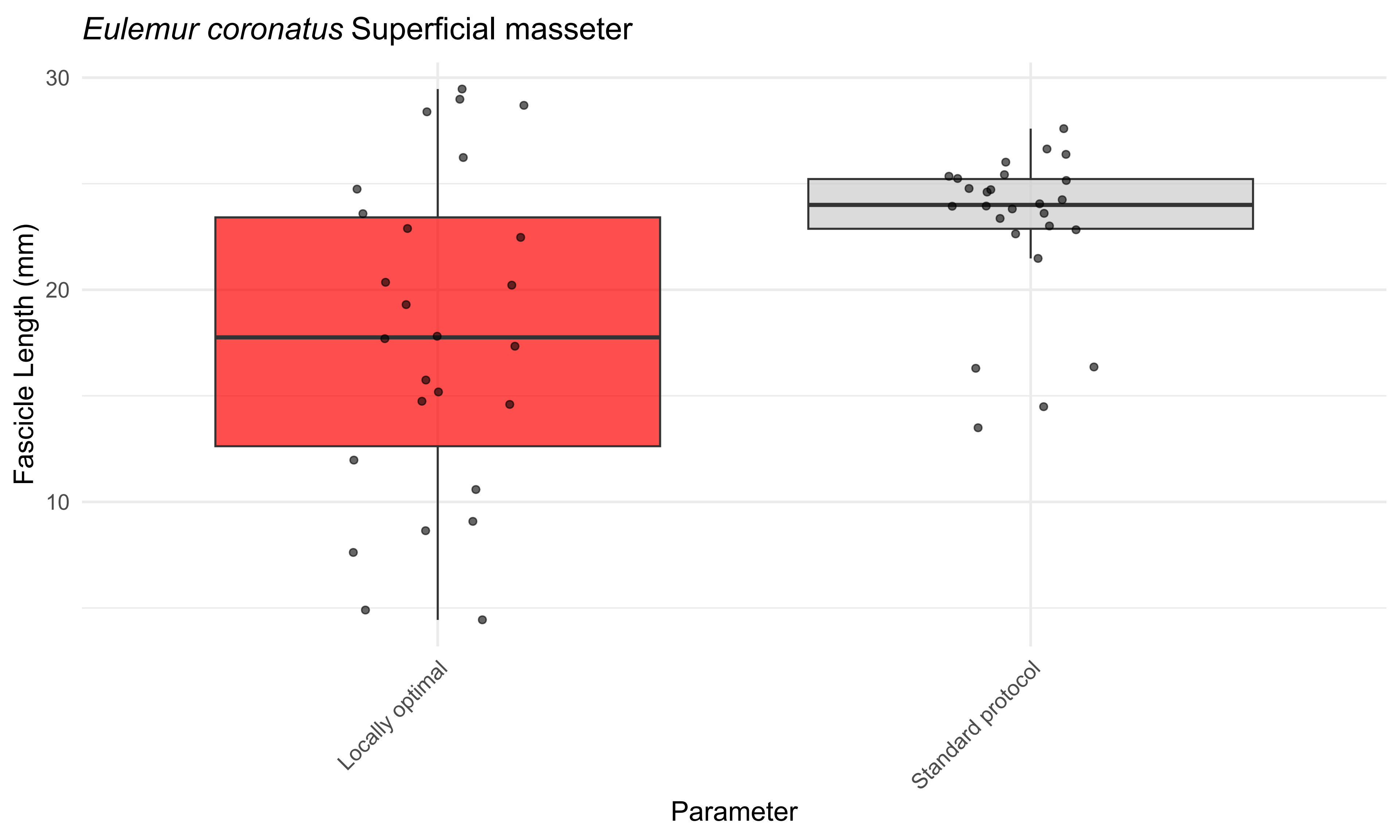
Eulemur coronatus Superficial masseter
Fascicle Length (mm)
30
20
10
Locally optimal
Standard protocol
Parameter

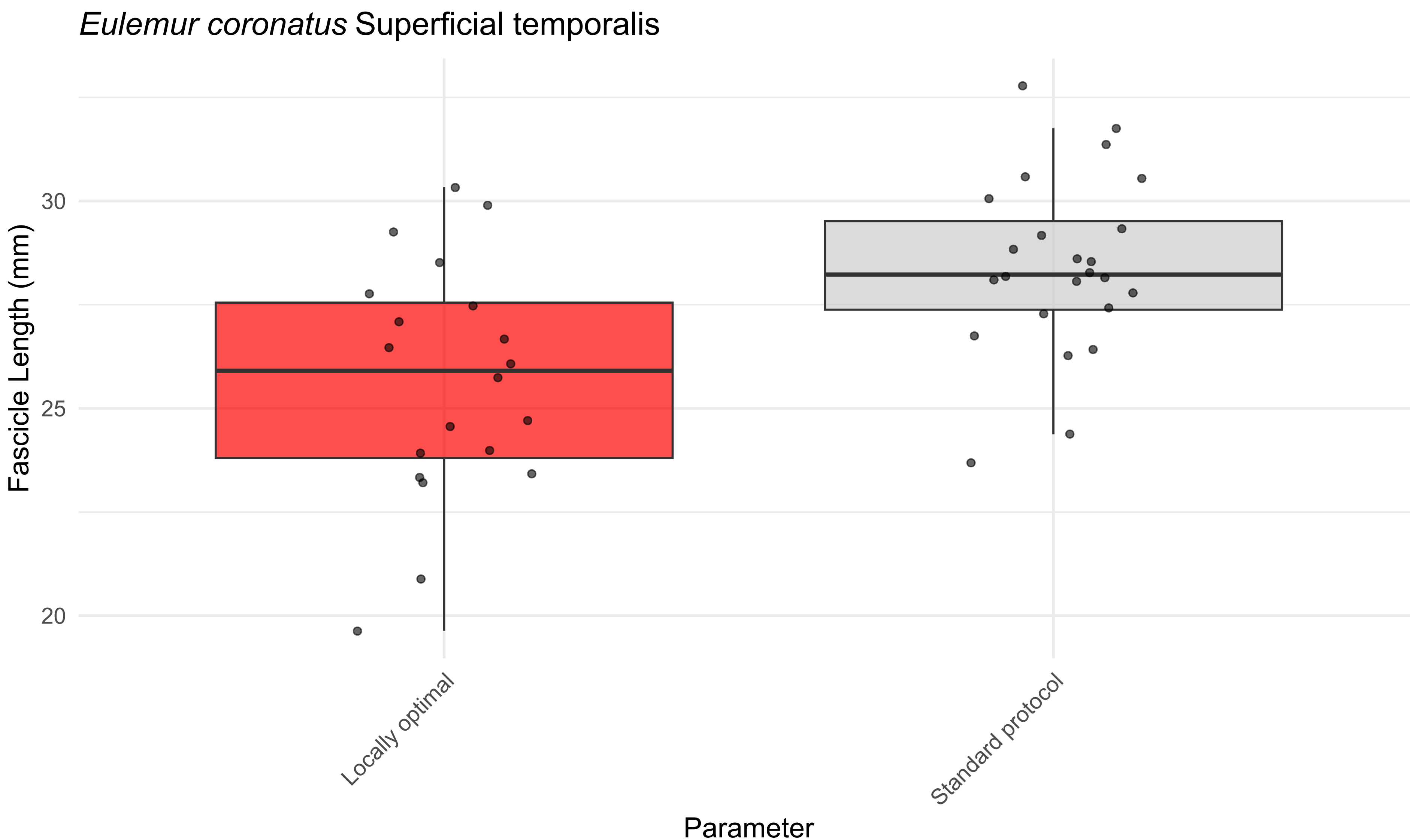

Eulemur coronatus Superficial temporalis
Fascicle Length (mm)
30
25
20
Locally optimal
Standard protocol
Parameter

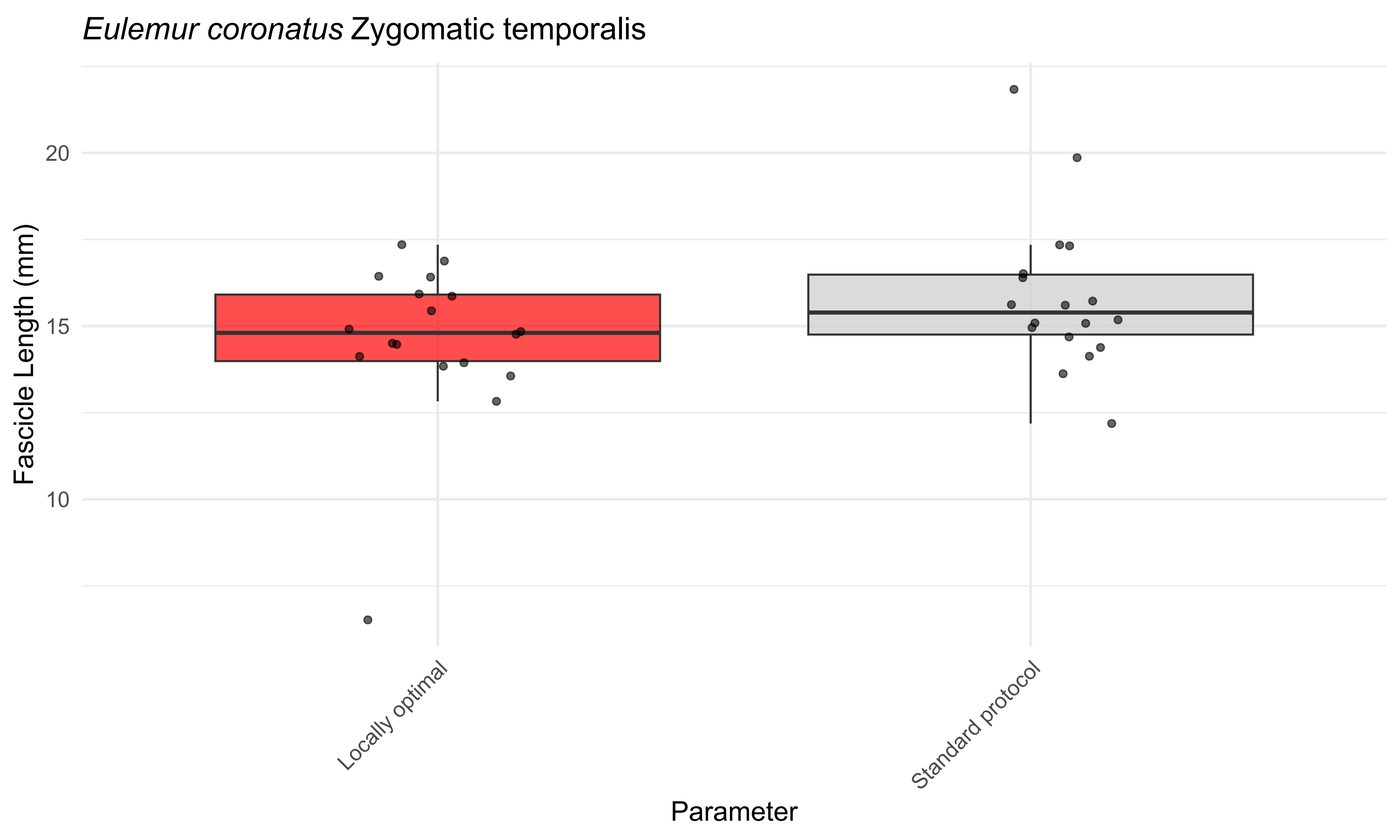

Eulemur coronatus Zygomatic temporalis
Fascicle Length (mm)
20
15
10
Locally optimal
Standard protocol
Parameter

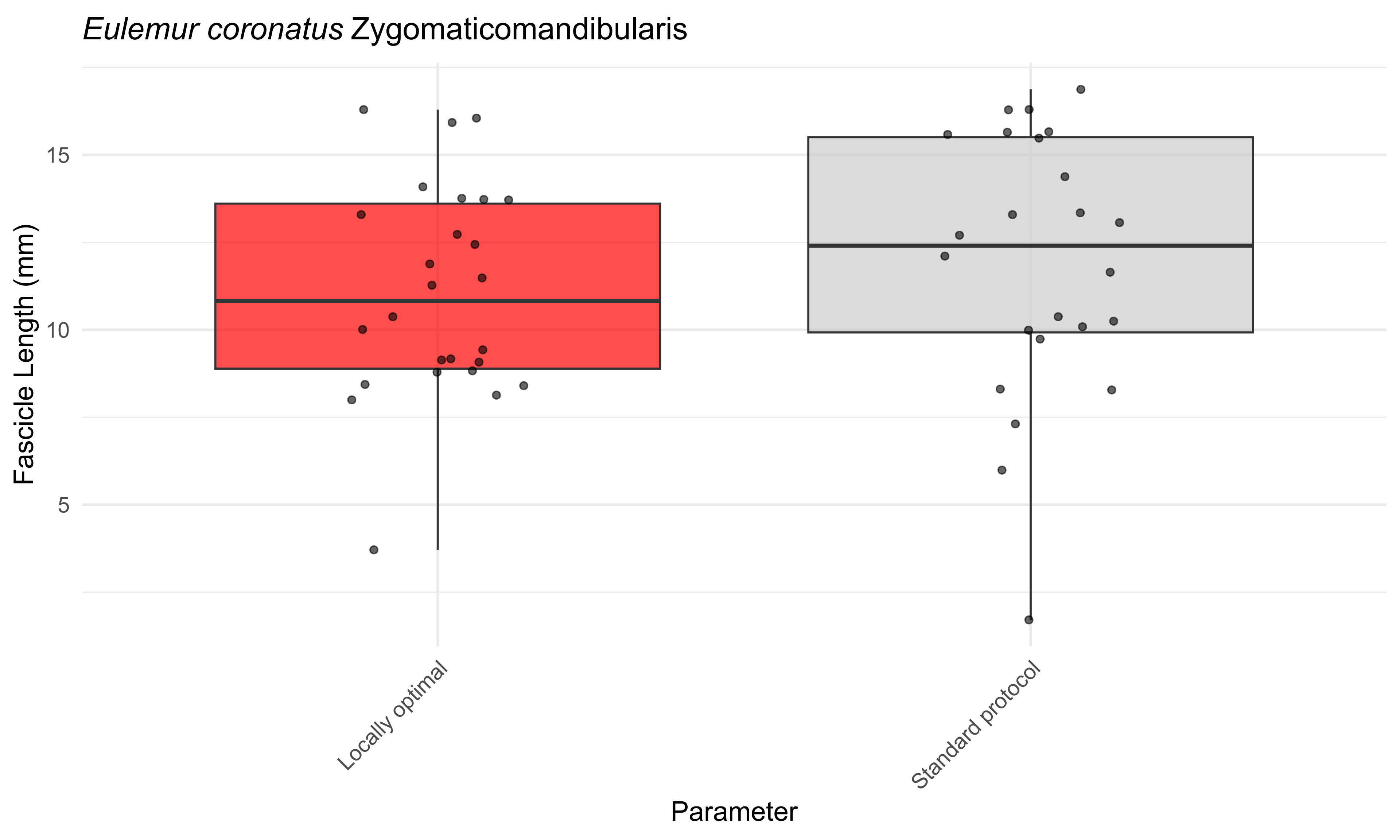

Eulemur coronatus Zygomaticomandibularis
Fascicle Length (mm)
15
10
5
Locally optimal
Standard protocol
Parameter

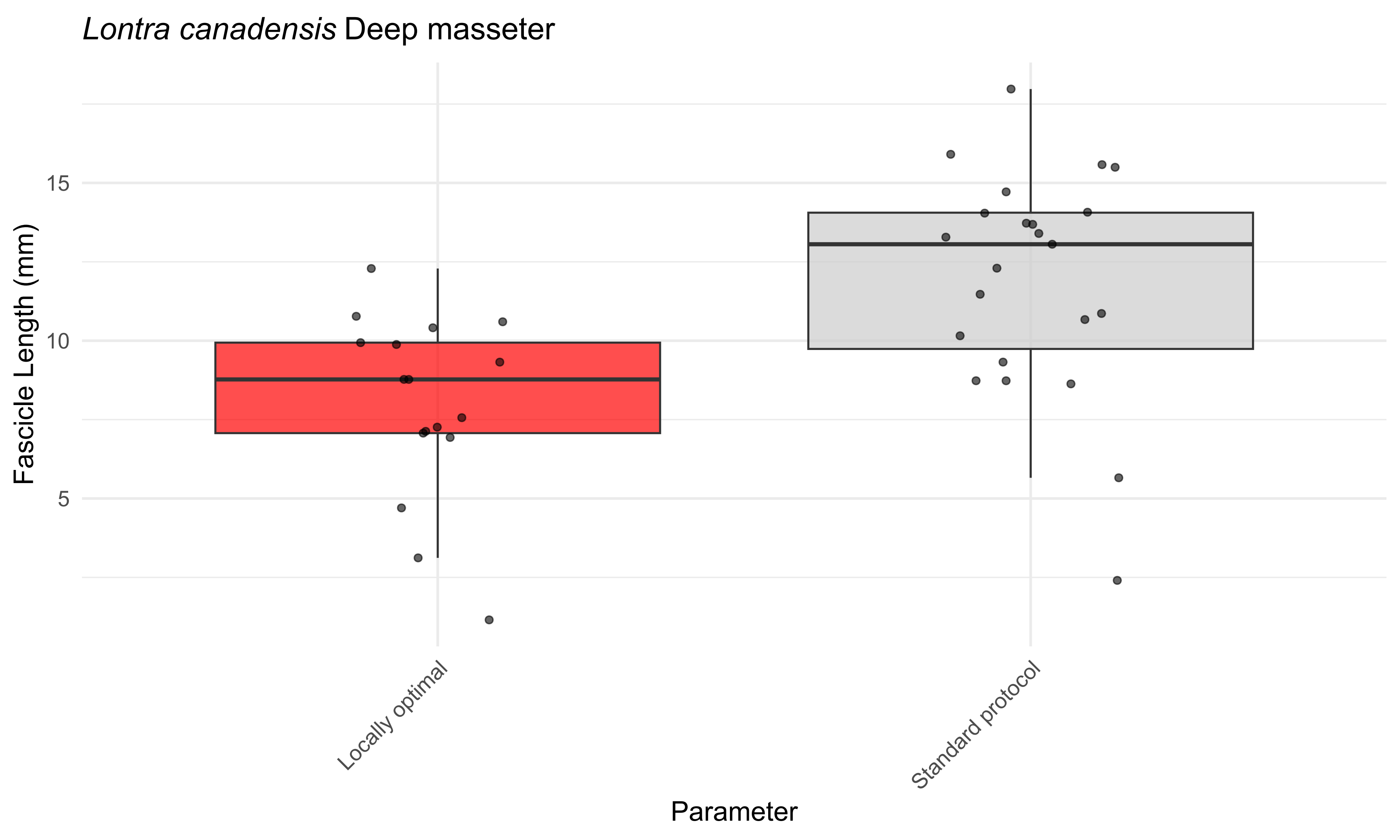

Lontra canadensis Deep masseter
Fascicle Length (mm)
15
10
5
Locally optimal
Standard protocol
Parameter

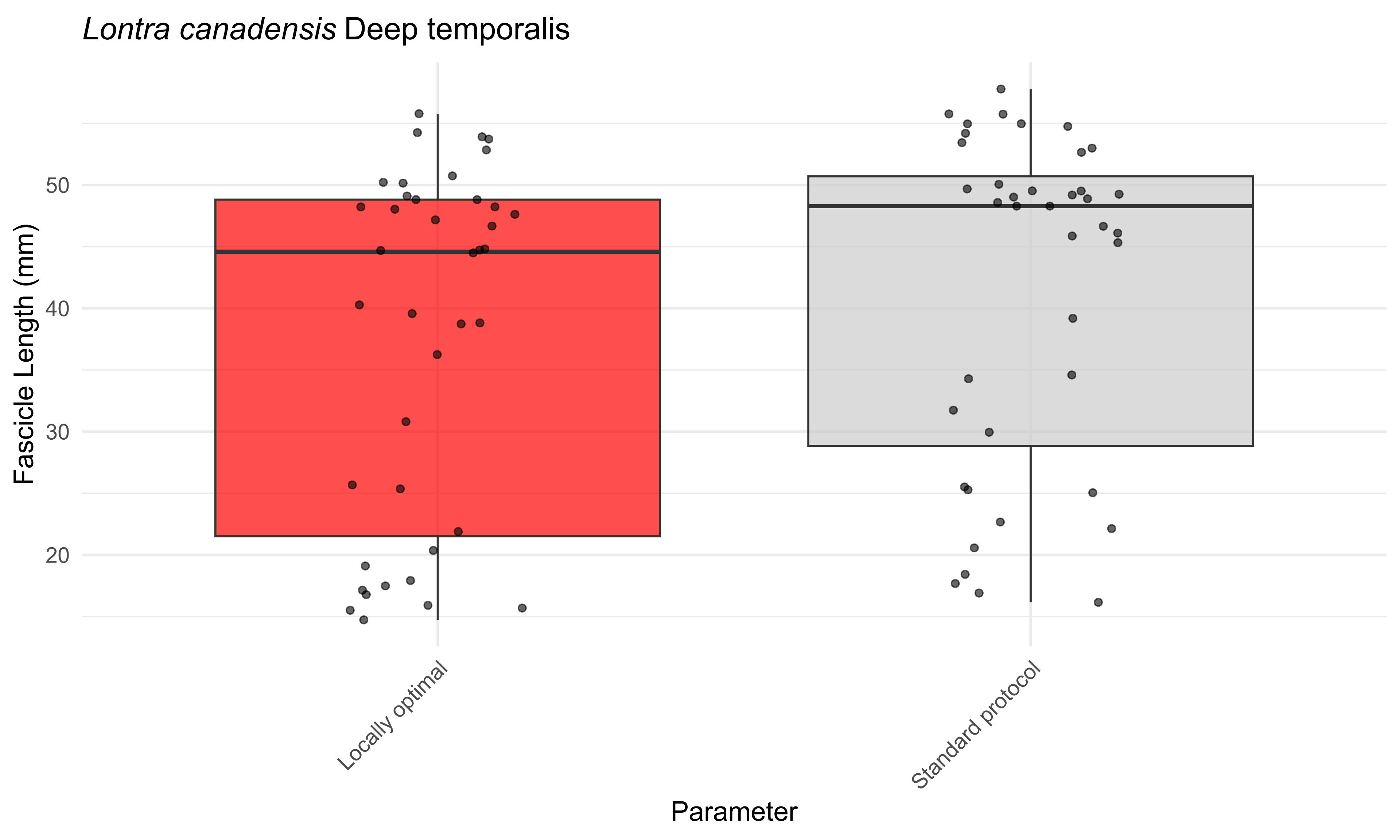
Lontra canadensis Deep temporalis
Fascicle Length (mm)
50
40
30
20
Locally optimal
Standard protocol
Parameter

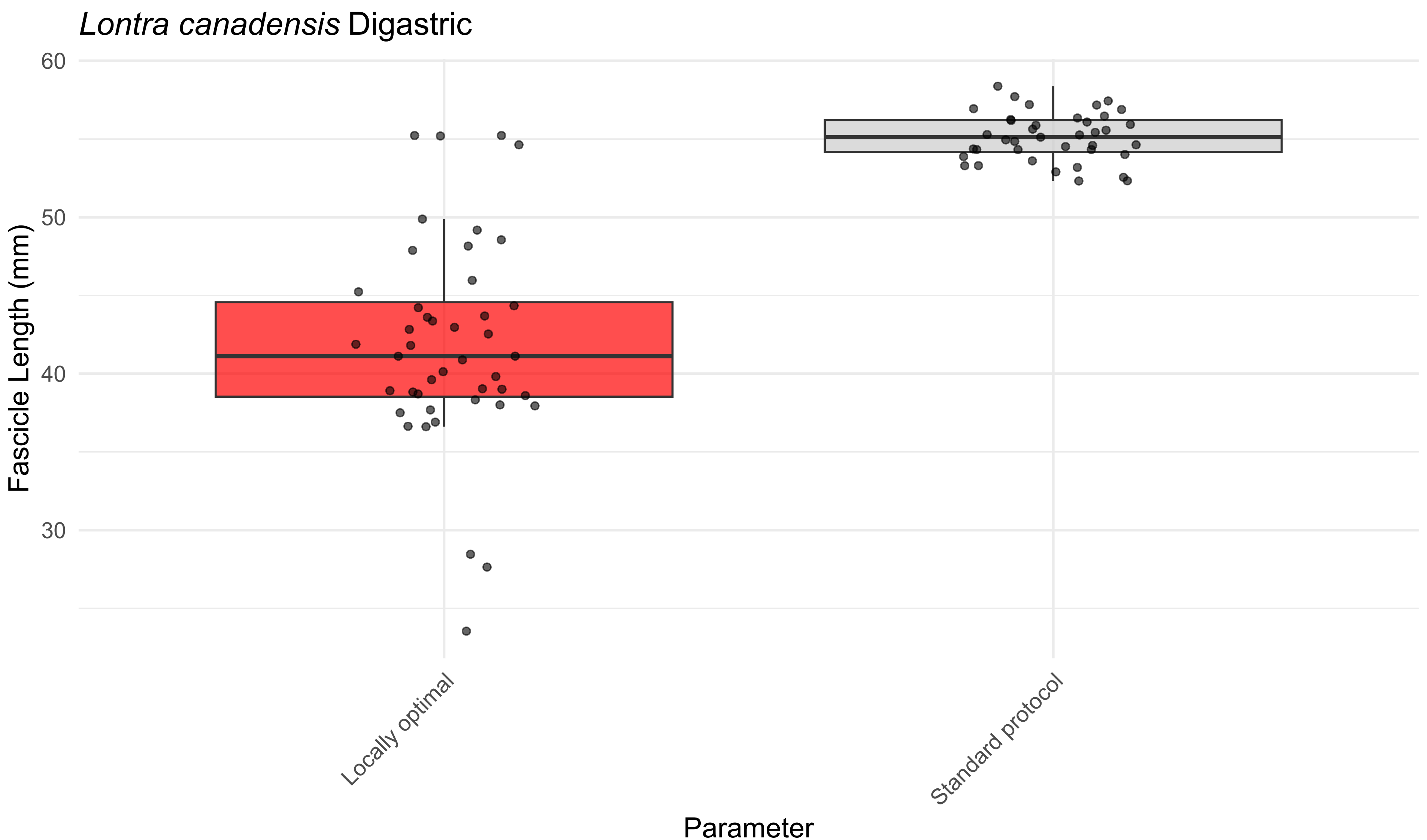

Lontra canadensis Digastric
60
50
40
30
Fascicle Length (mm)
Locally optimal
Standard protocol
Parameter

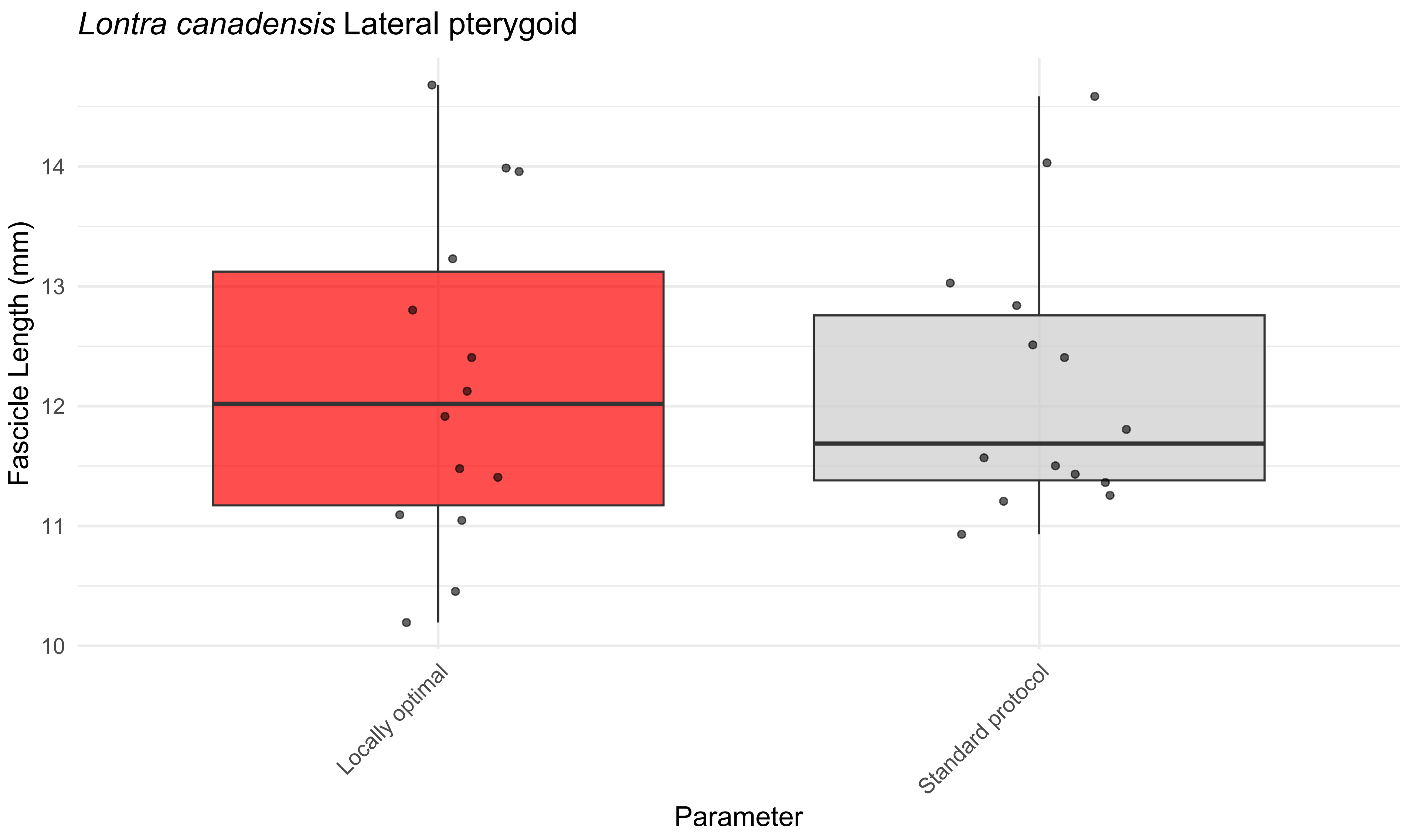

Lontra canadensis Lateral pterygoid
Fascicle Length (mm)
14
13
12
11
10
Locally optimal
Standard protocol
Parameter

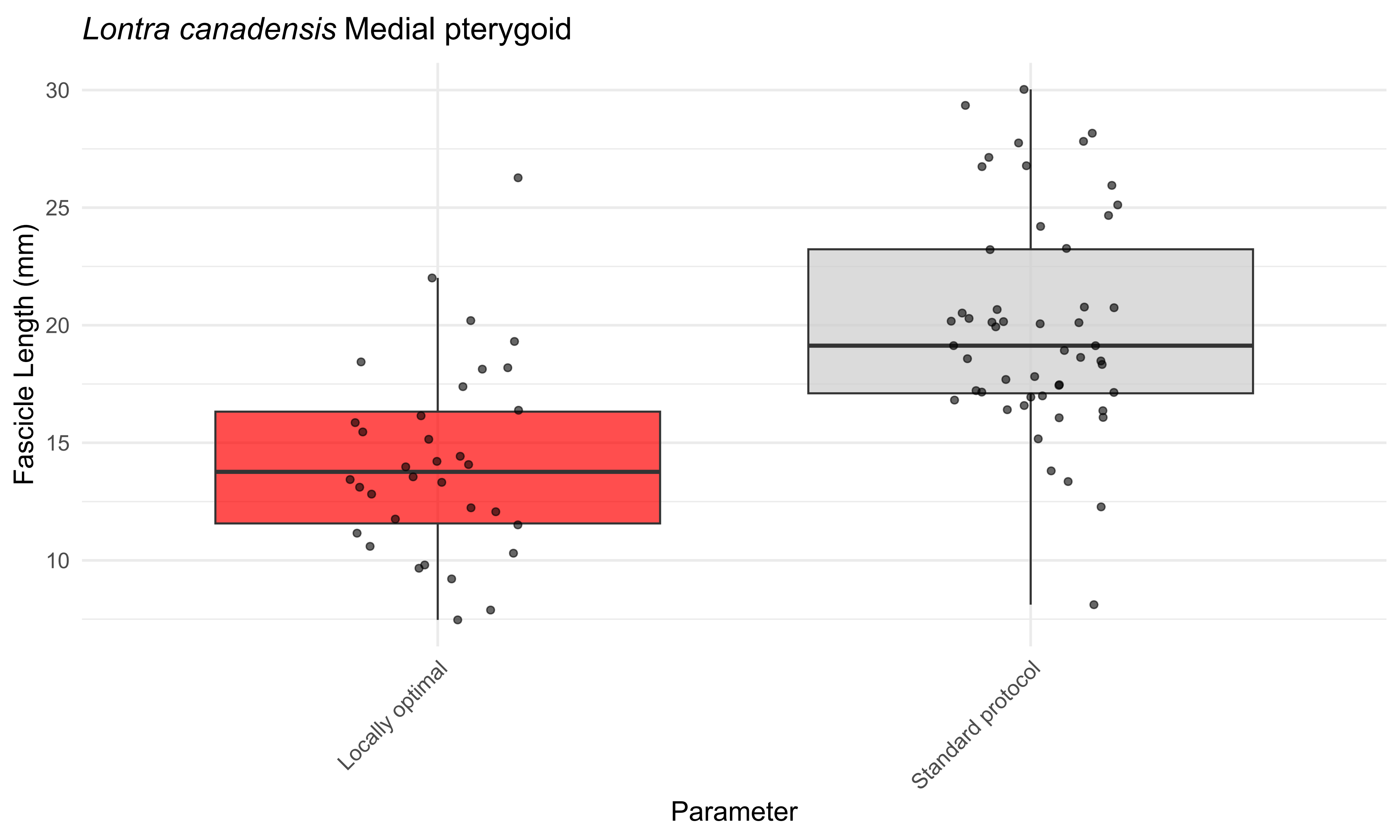

Lontra canadensis Medial pterygoid
30
25
20
15
10
Fascicle Length (mm)
Locally optimal
Standard protocol
Parameter

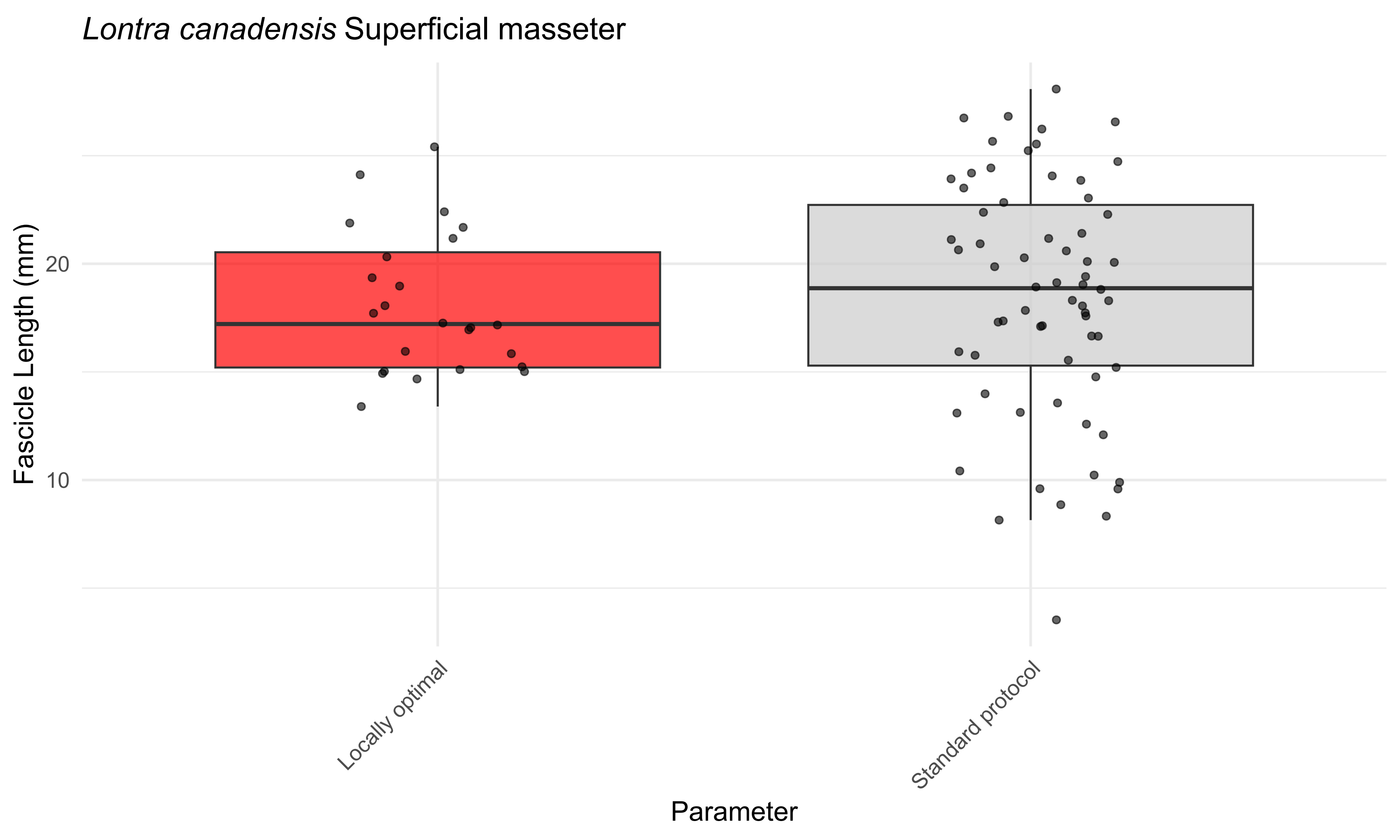

Lontra canadensis Superficial masseter
Fascicle Length (mm)
20
10
Locally optimal
Standard protocol
Parameter

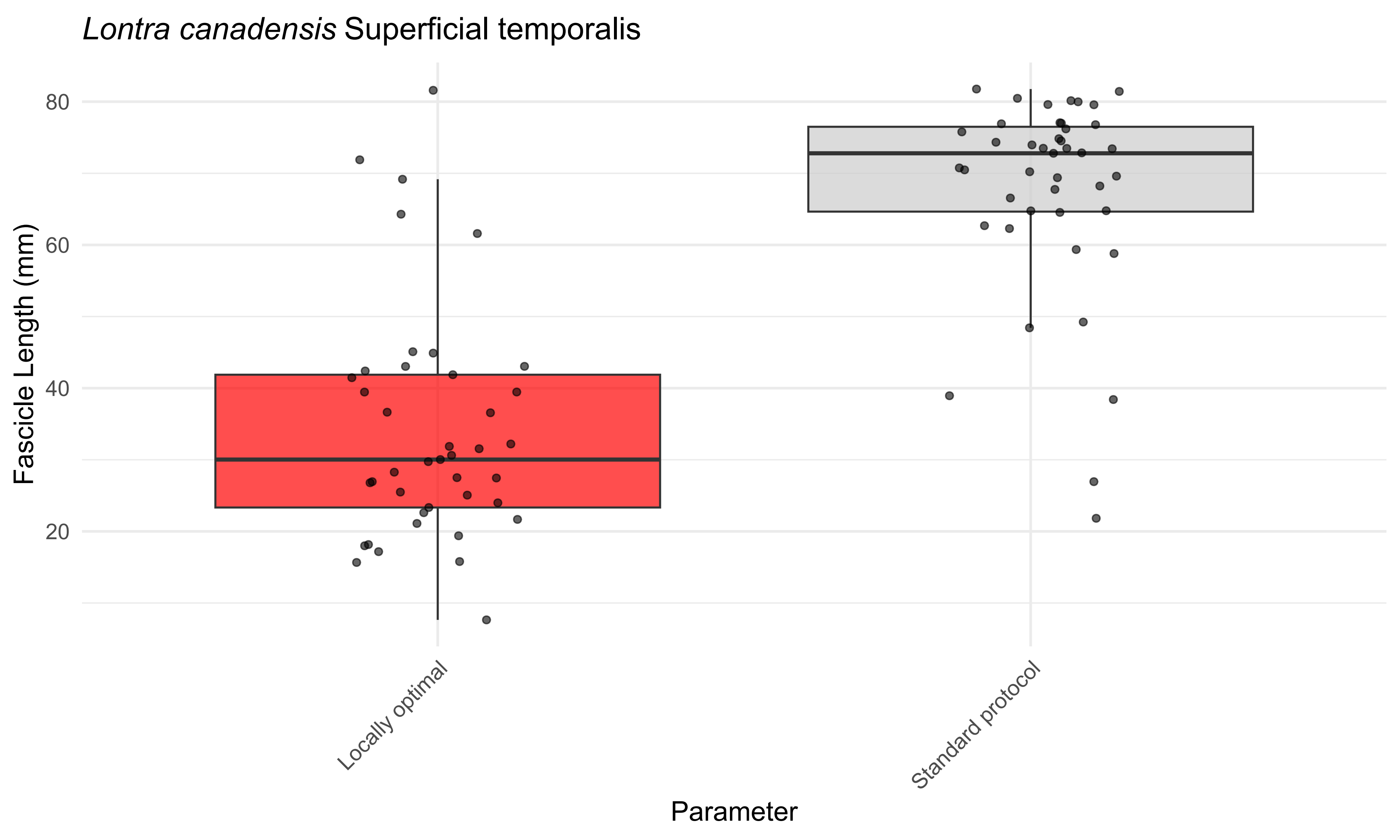
Lontra canadensis Superficial temporalis
Fascicle Length (mm)
80
60
40
20
Locally optimal
Standard protocol
Parameter

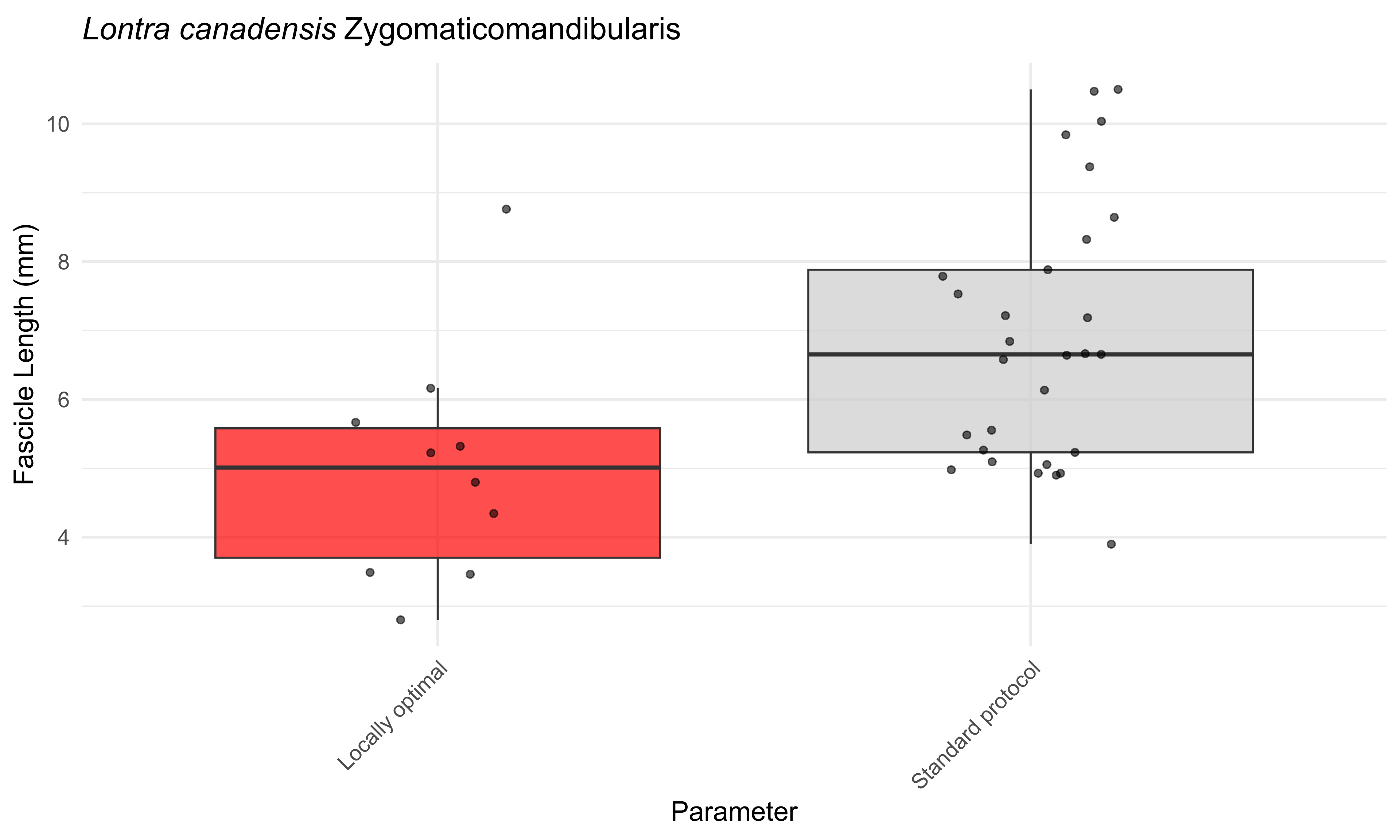

Lontra canadensis Zygomaticomandibularis
Fascicle Length (mm)
10
8
6
4
Locally optimal
Standard protocol
Parameter

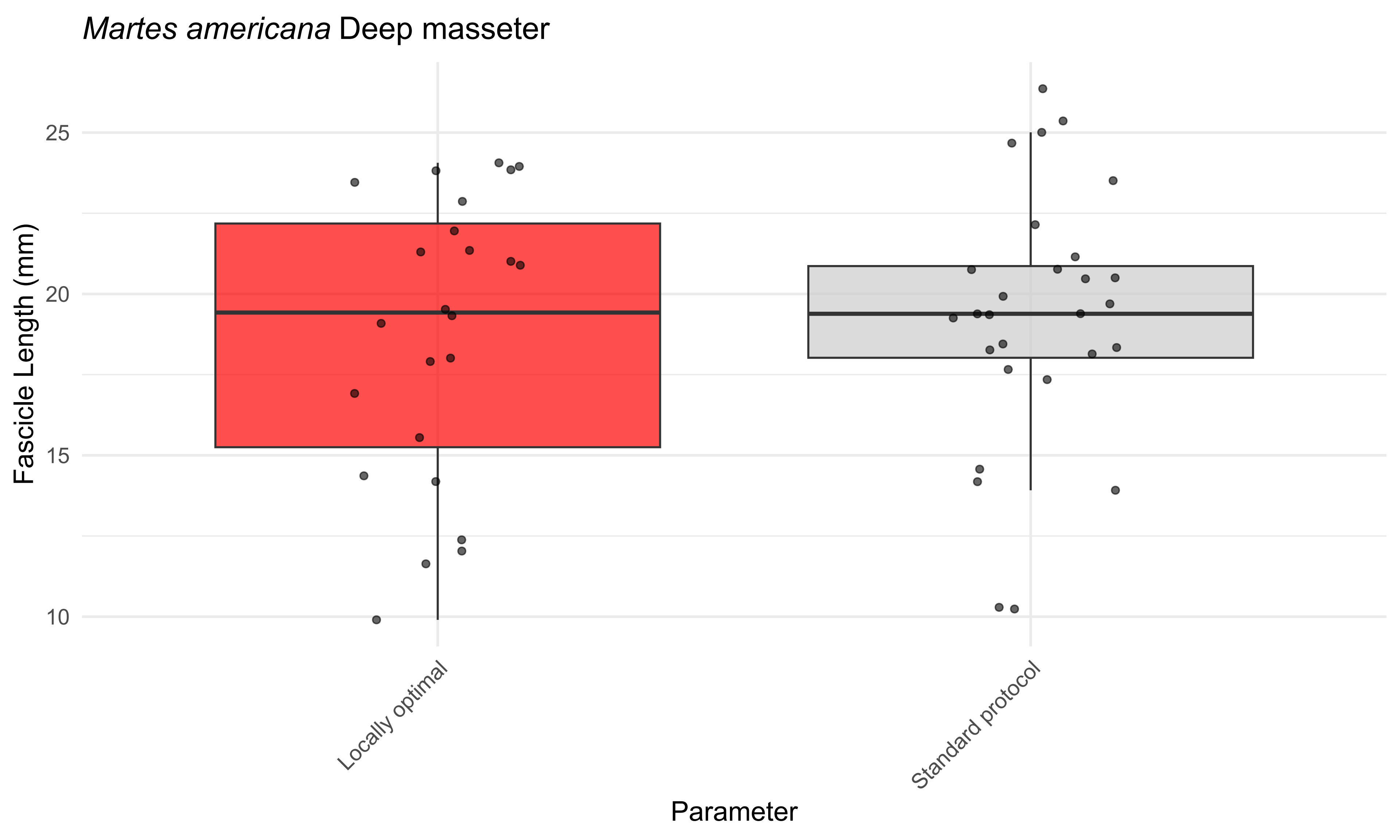
Martes americana Deep masseter
Fascicle Length (mm)
25
20
15
10
Locally optimal
Standard protocol
Parameter

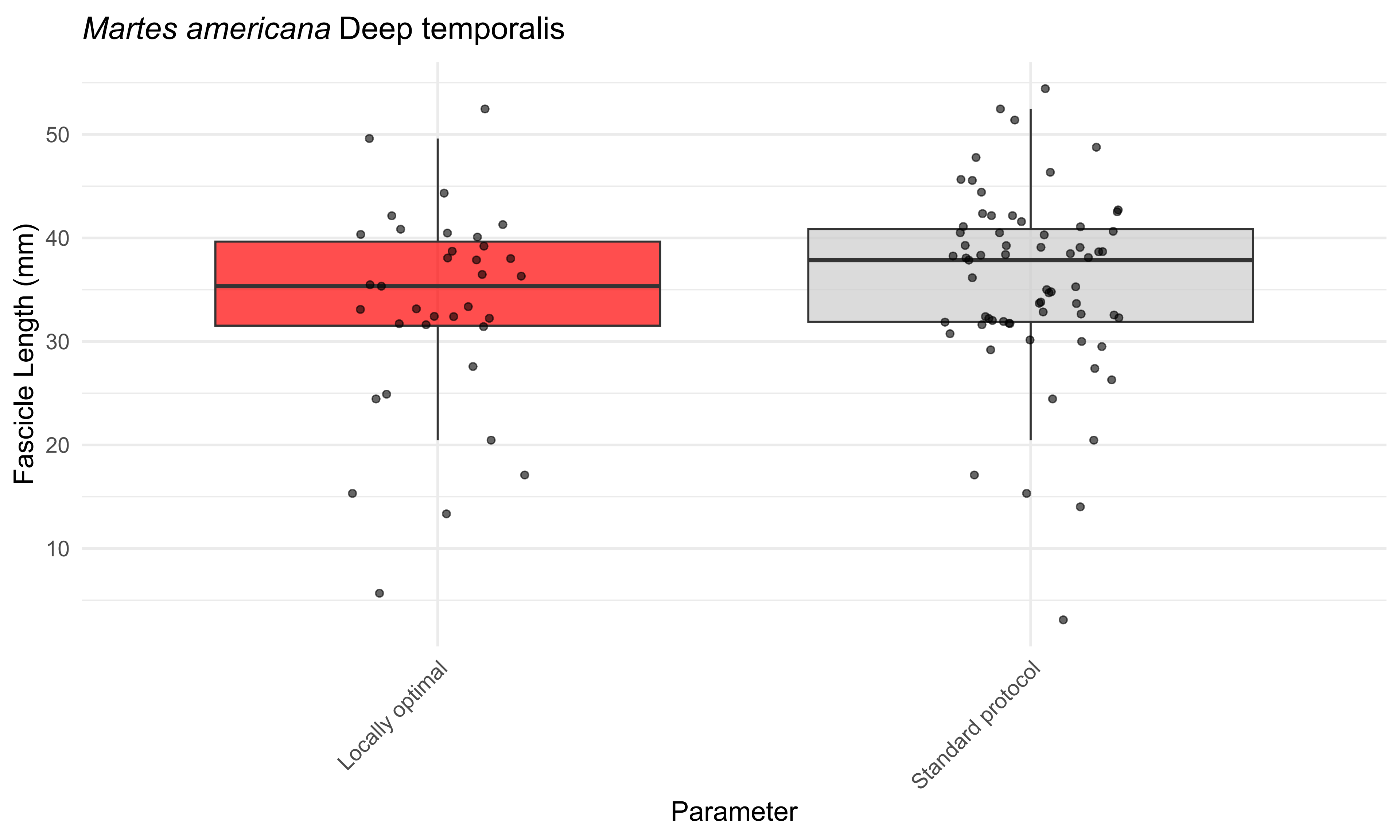

Martes americana Deep temporalis
Fascicle Length (mm)
50
40
30
20
10
Locally optimal
Standard protocol
Parameter

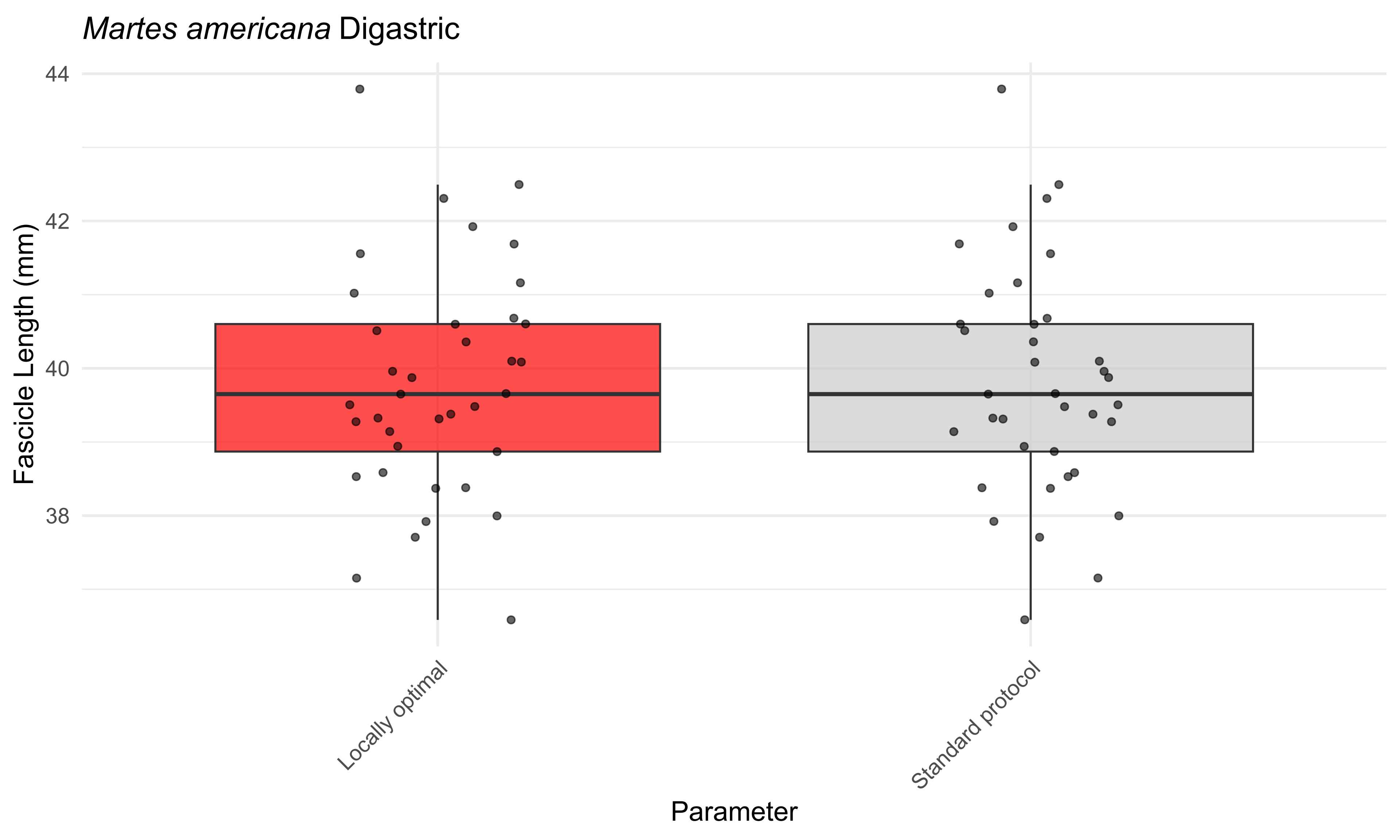

Martes americana Digastric
Fascicle Length (mm)
44
42
40
38
Locally optimal
Standard protocol
Parameter

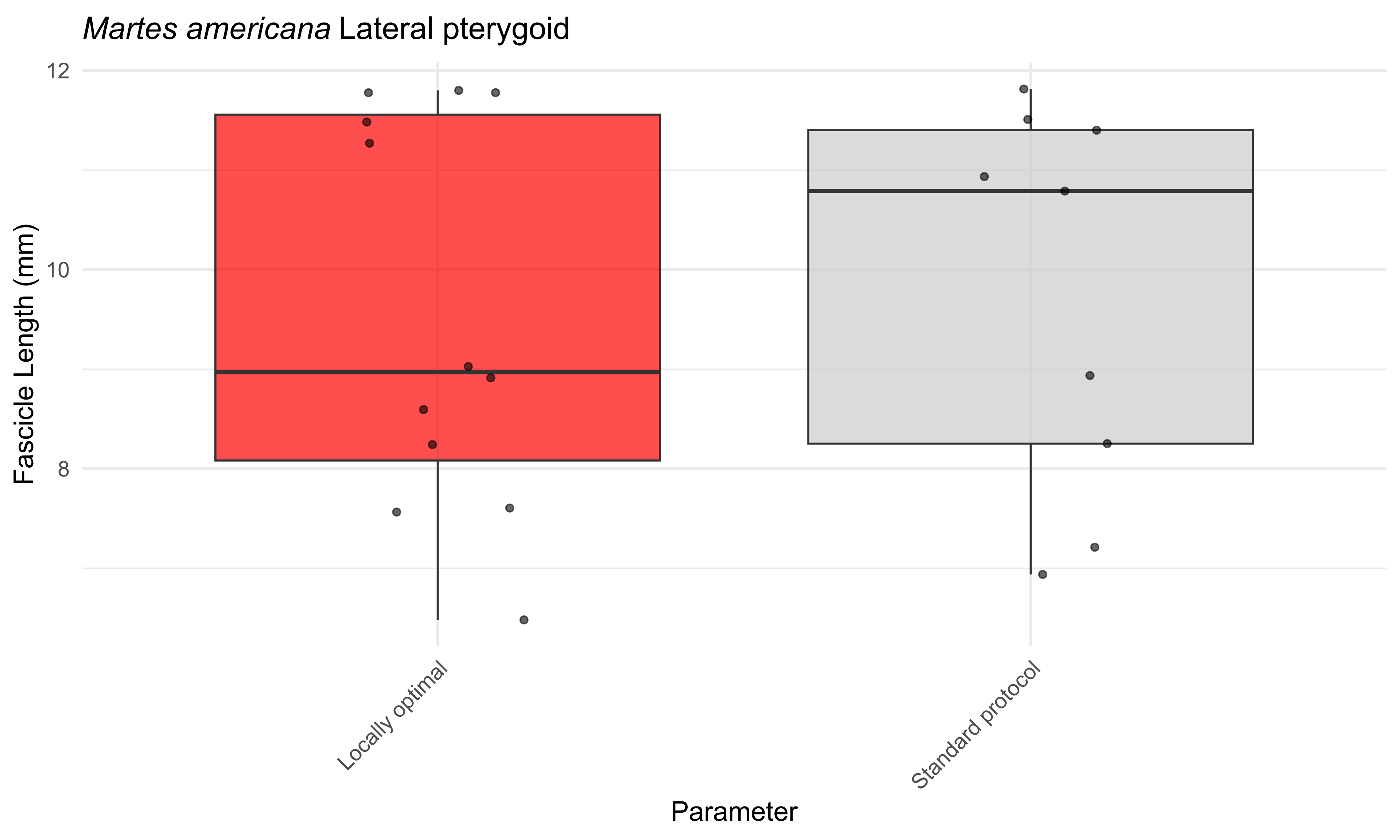

Martes americana Lateral pterygoid
Fascicle Length (mm)
12
10
8
Locally optimal
Standard protocol
Parameter

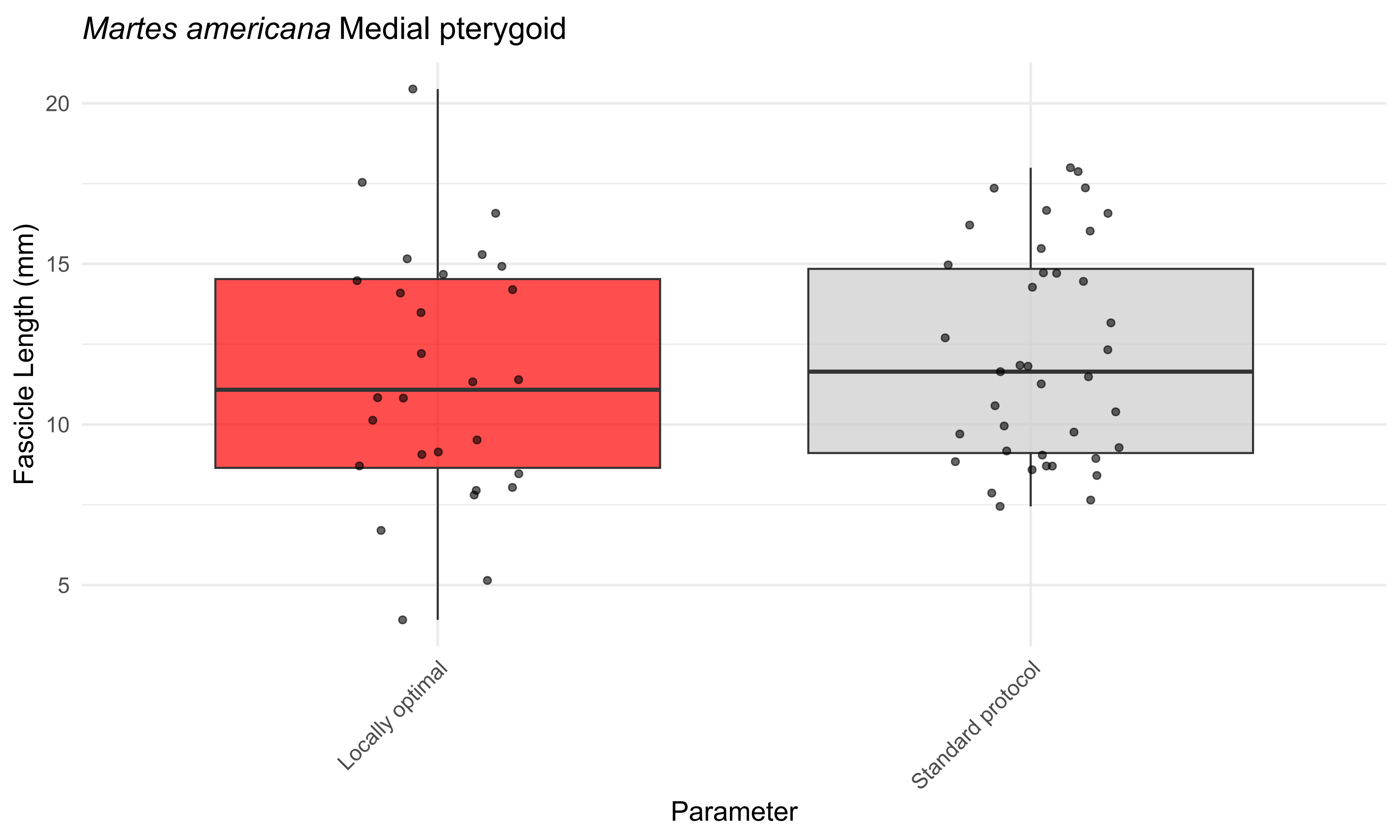
Martes americana Medial pterygoid
Fascicle Length (mm)
20
15
10
5
Locally optimal
Standard protocol
Parameter

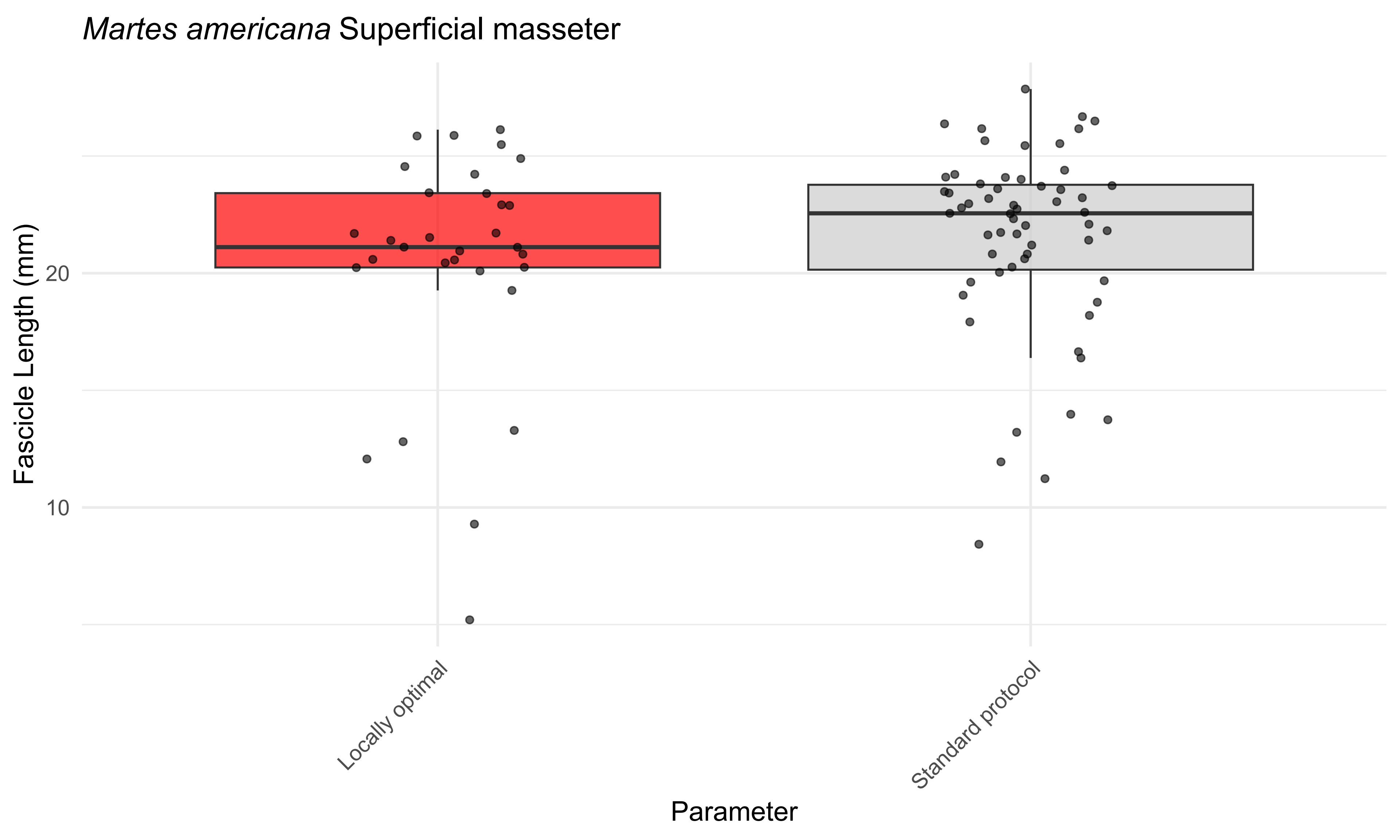
Martes americana Superficial masseter
Fascicle Length (mm)
20
10
Locally optimal
Standard protocol
Parameter

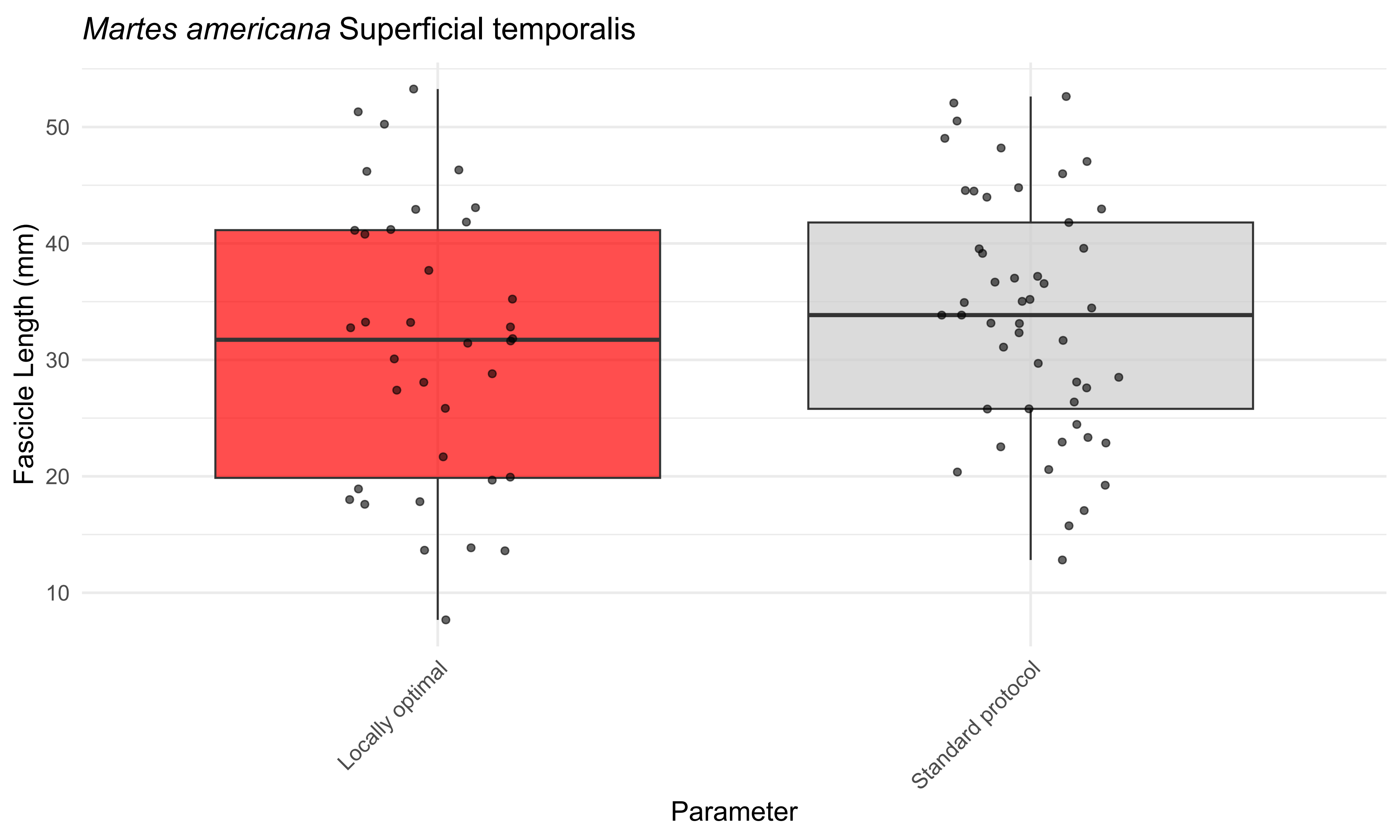

Martes americana Superficial temporalis
Fascicle Length (mm)
50
40
30
20
10
Locally optimal
Standard protocol
Parameter

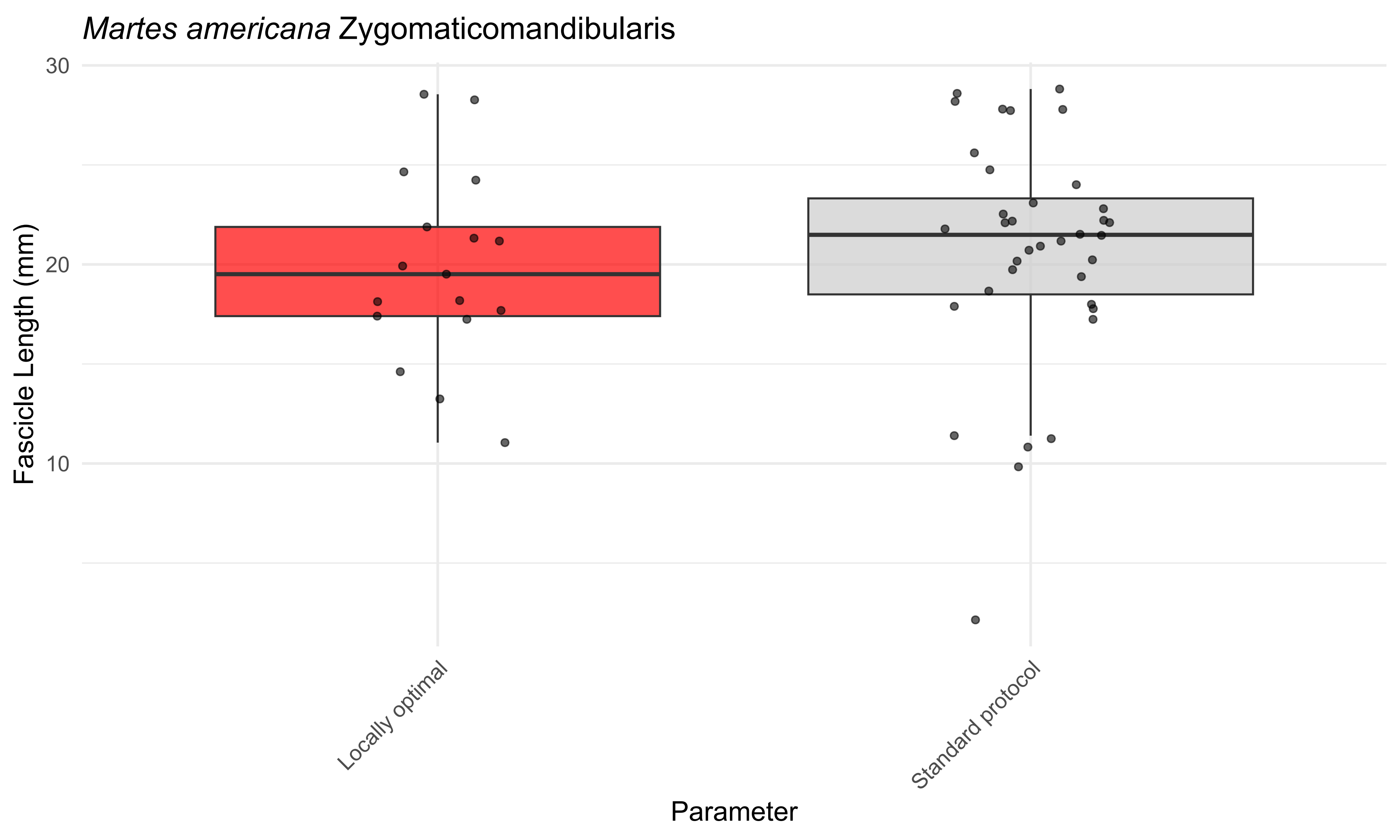

Martes americana Zygomaticomandibularis
30
20
10
Fascicle Length (mm)
Locally optimal
Standard protocol
Parameter

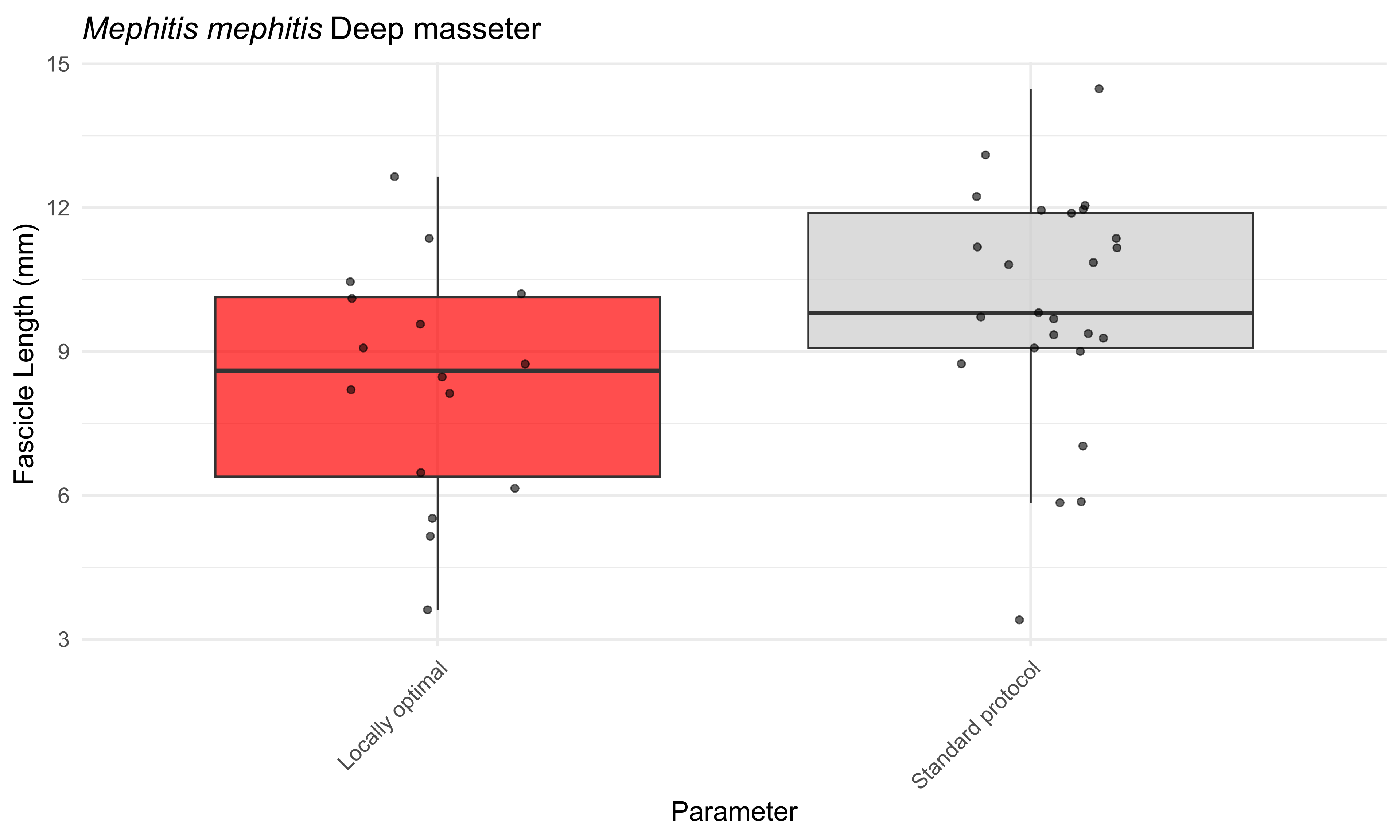

Mephitis mephitis Deep masseter
15
12
9
6
3
Fascicle Length (mm)
Locally optimal
Standard protocol
Parameter

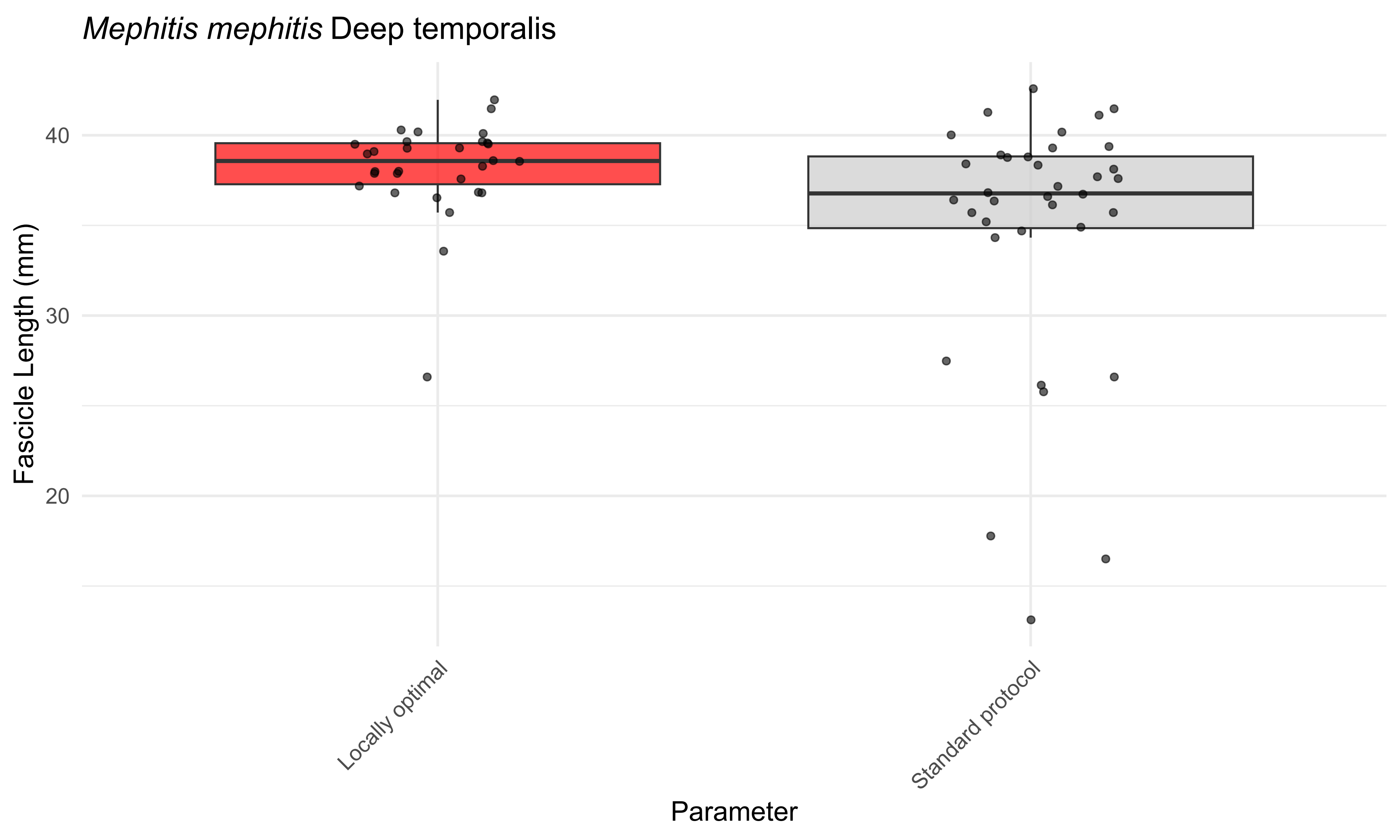
Mephitis mephitis Deep temporalis
Fascicle Length (mm)
40
30
20
Locally optimal
Standard protocol
Parameter

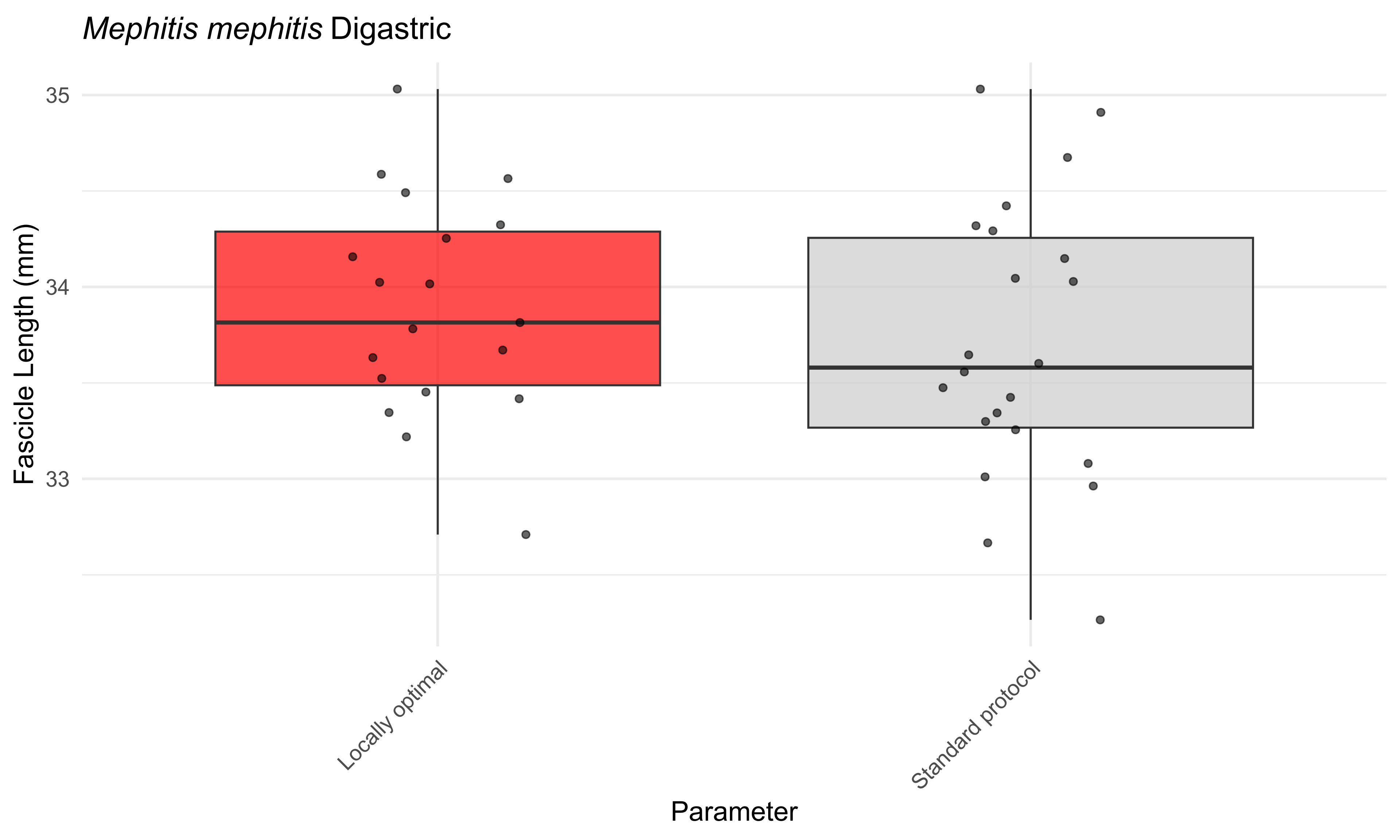

Mephitis mephitis Digastric
Fascicle Length (mm)
35
34
33
Locally optimal
Standard protocol
Parameter

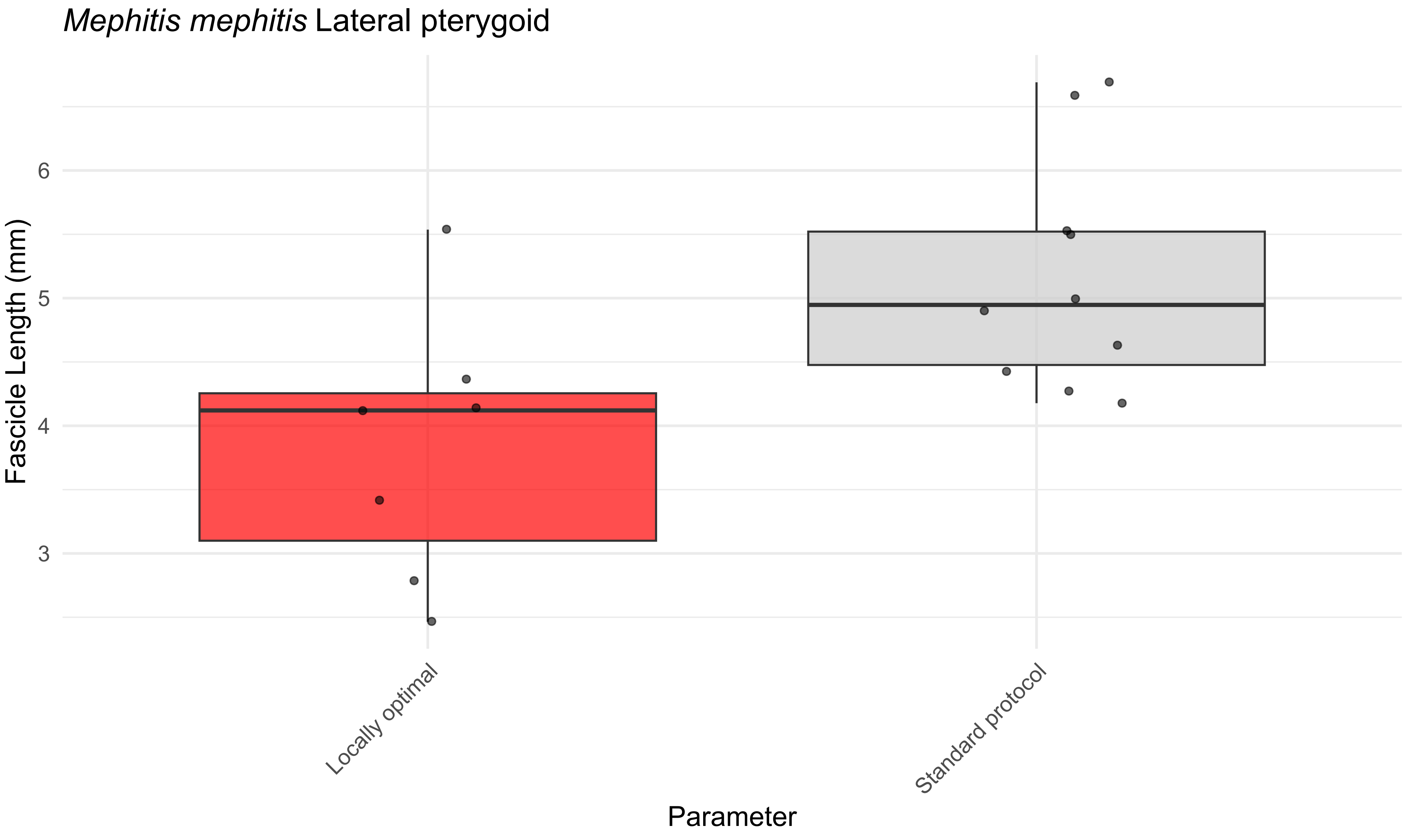
Mephitis mephitis Lateral pterygoid
Fascicle Length (mm)
6
5
4
3
Locally optimal
Standard protocol
Parameter

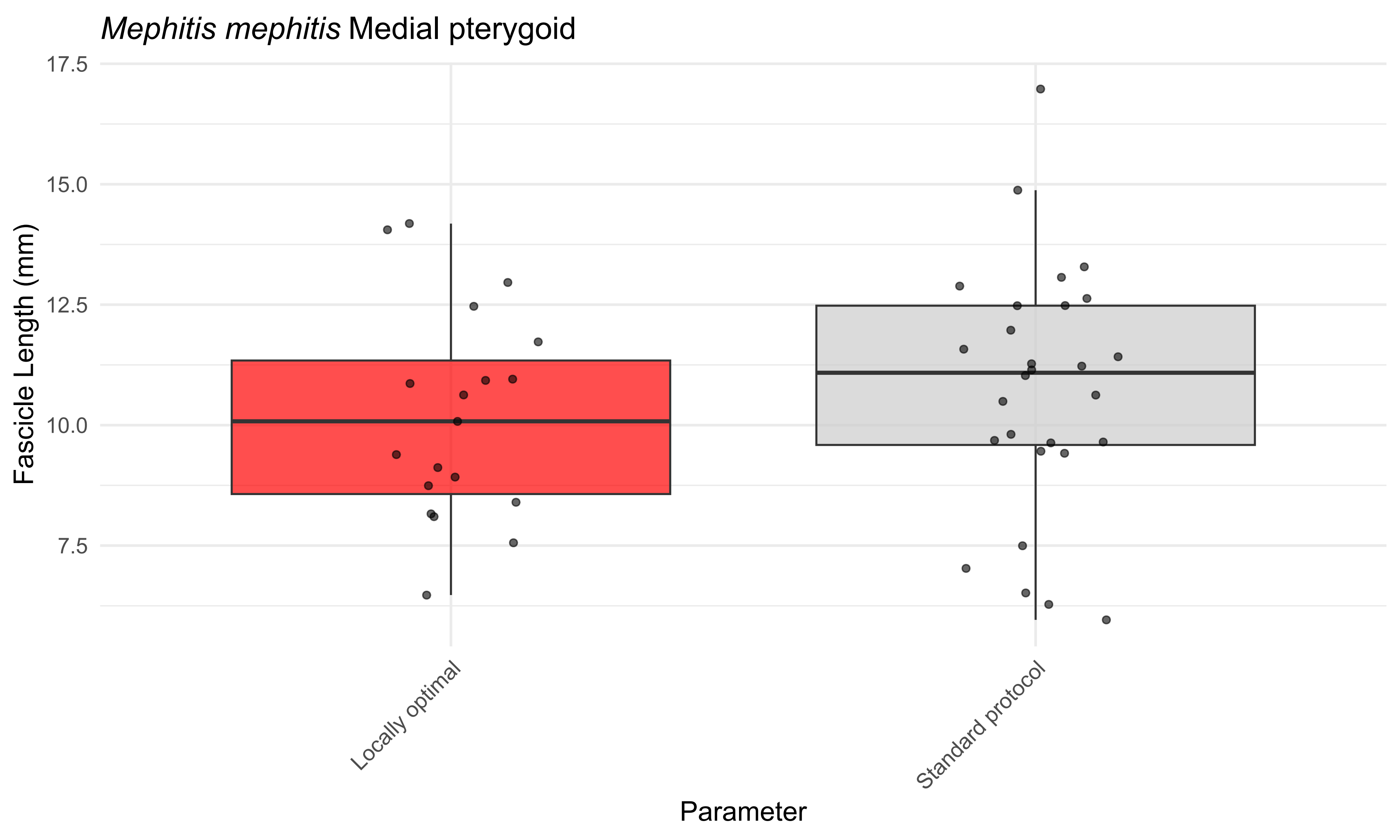

Mephitis mephitis Medial pterygoid
17.5
15.0
12.5
10.0
7.5
Fascicle Length (mm)
Locally optimal
Standard protocol
Parameter

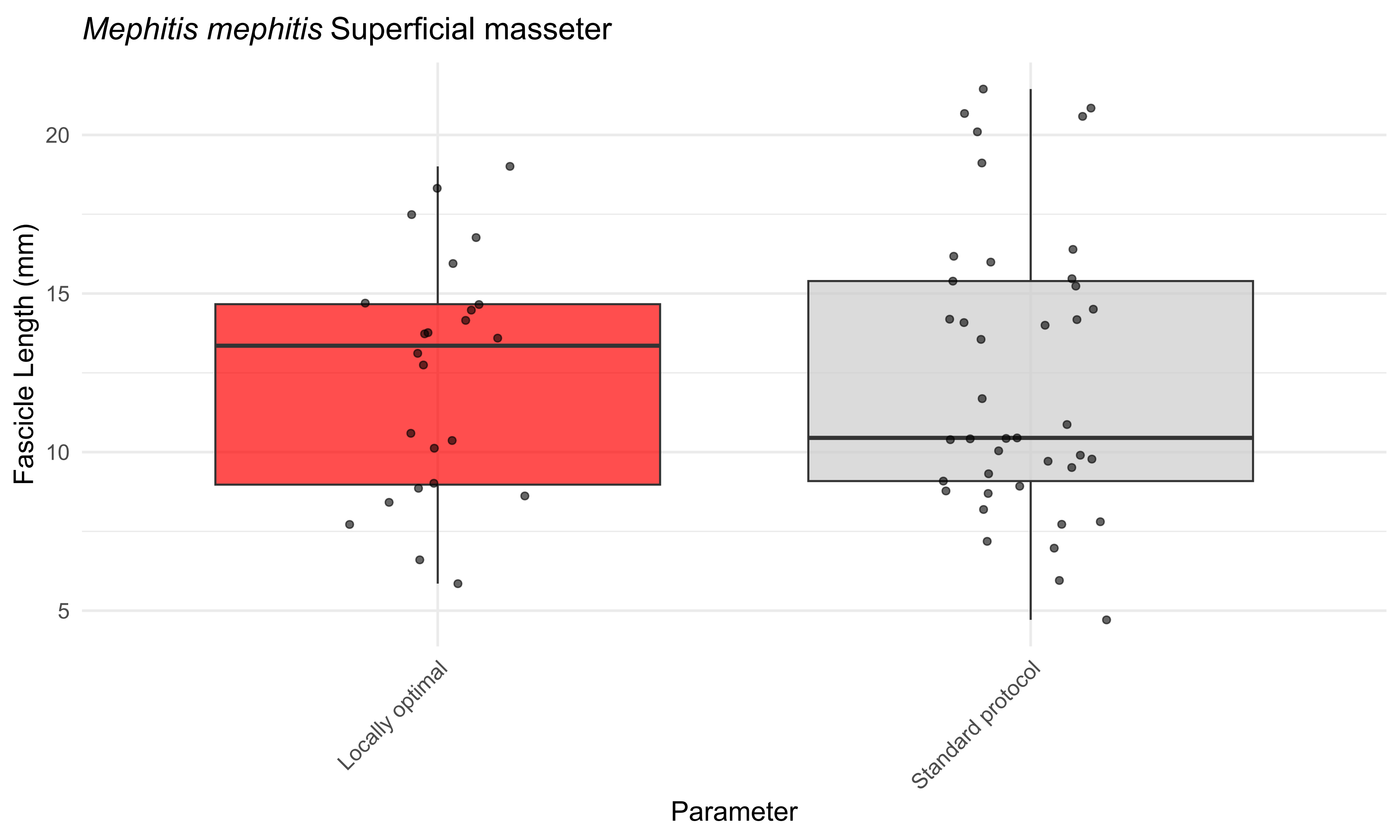

Mephitis mephitis Superficial masseter
Fascicle Length (mm)
20
15
10
5
Locally optimal
Standard protocol
Parameter

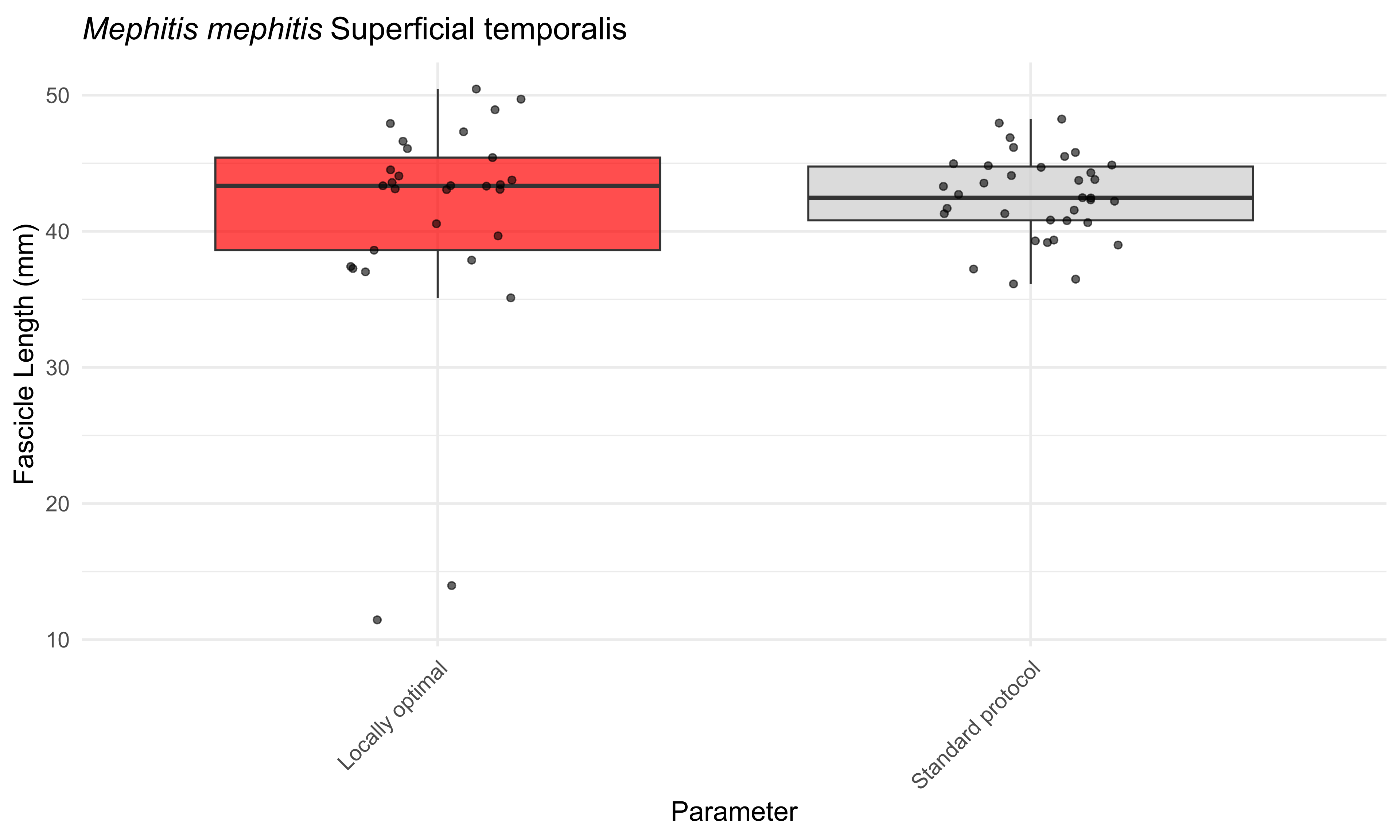

Mephitis mephitis Superficial temporalis
Fascicle Length (mm)
50
40
30
20
10
Locally optimal
Standard protocol
Parameter

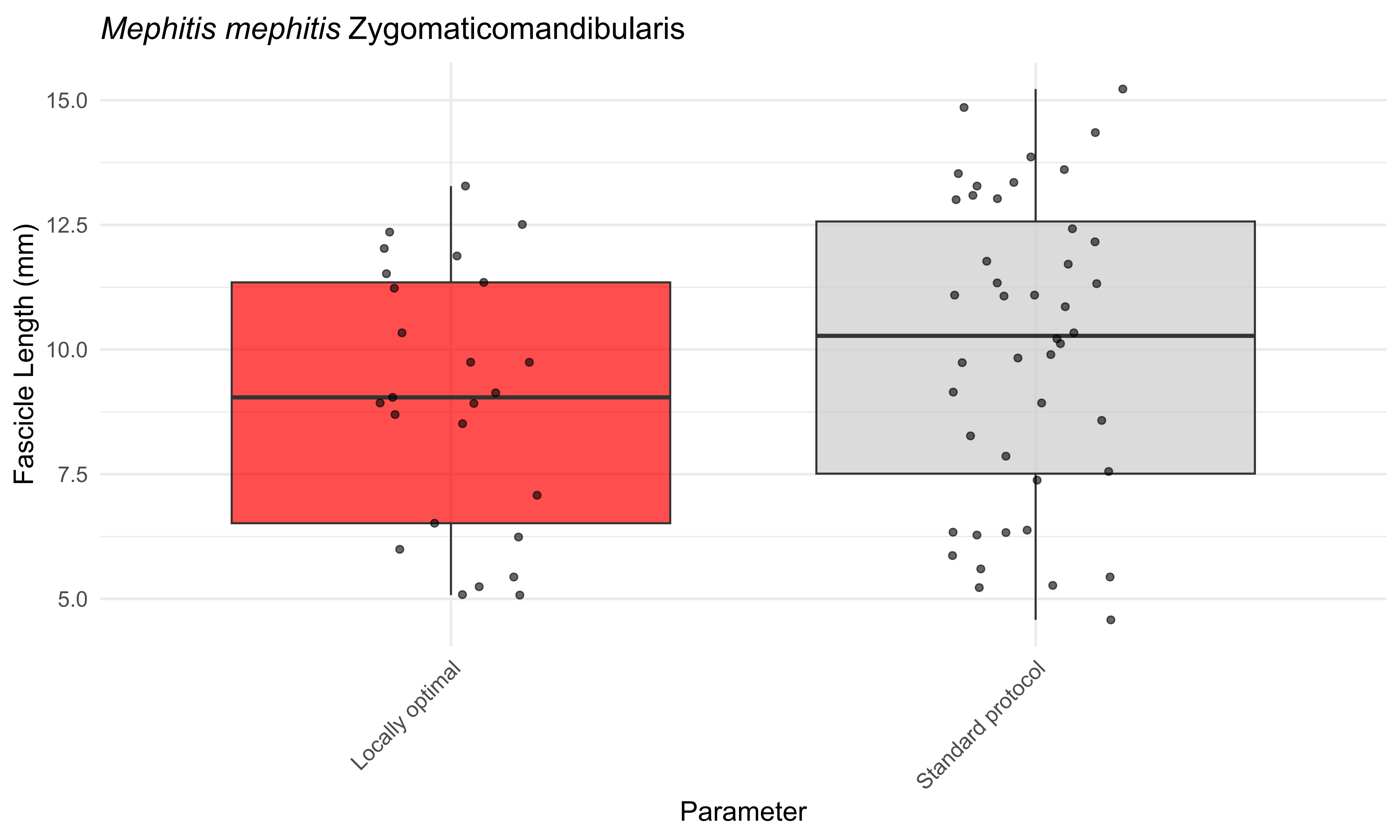

Mephitis mephitis Zygomaticomandibularis
15.0
12.5
10.0
7.5
5.0
Fascicle Length (mm)
Locally optimal
Standard protocol
Parameter

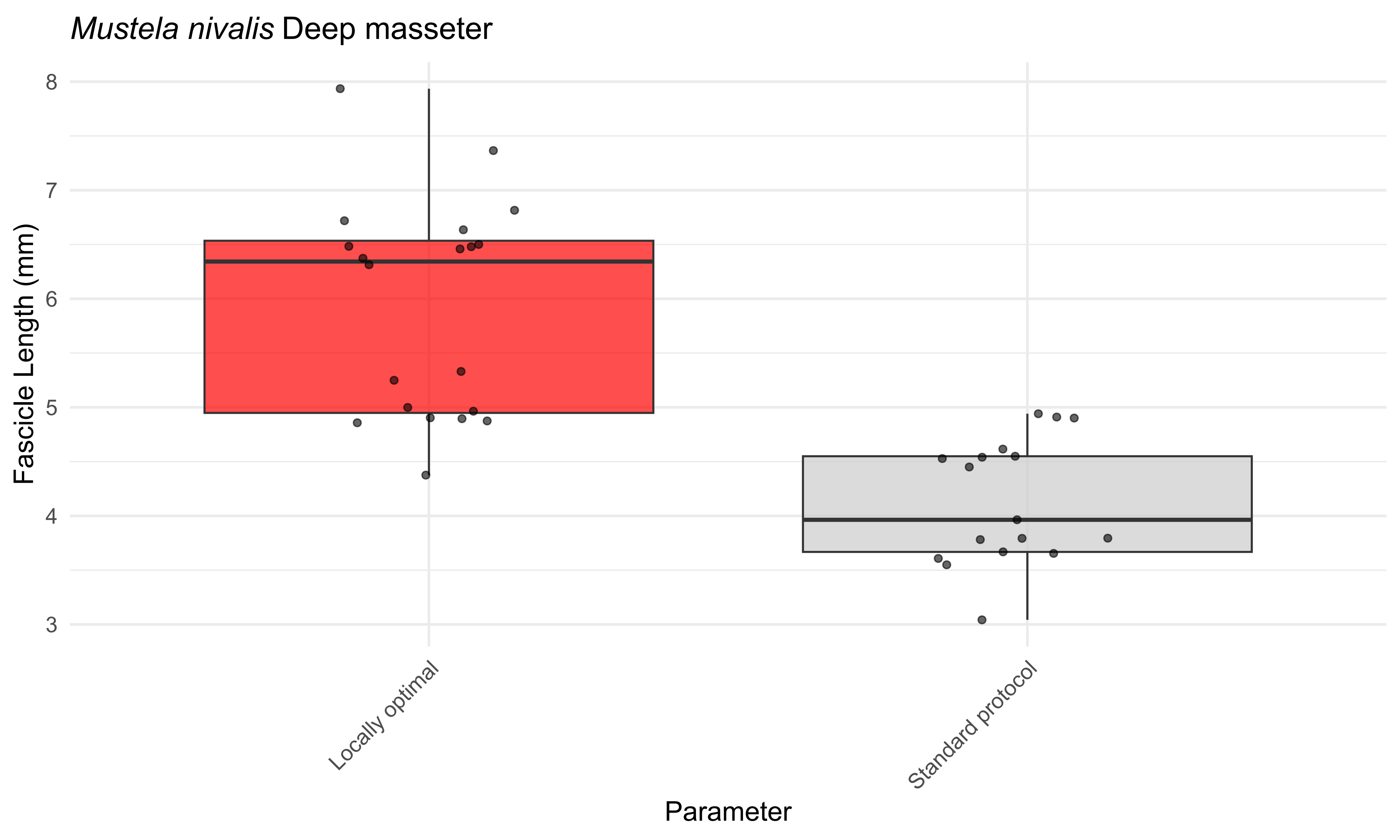

Mustela nivalis Deep masseter
Fascicle Length (mm)
8
7
6
5
4
3
Locally optimal
Standard protocol
Parameter

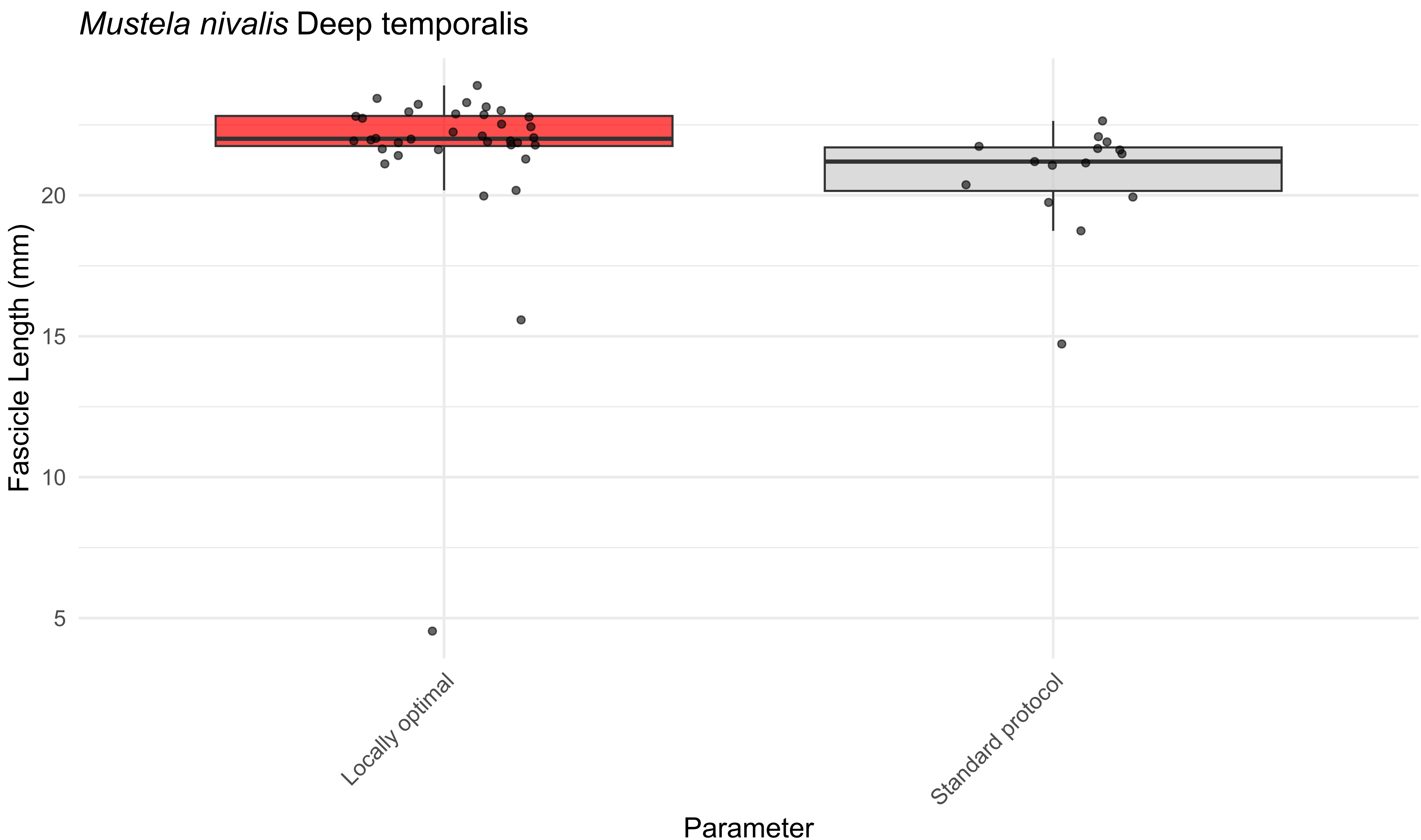

Mustela nivalis Deep temporalis
Fascicle Length (mm)
20
15
10
5
Locally optimal
Standard protocol
Parameter

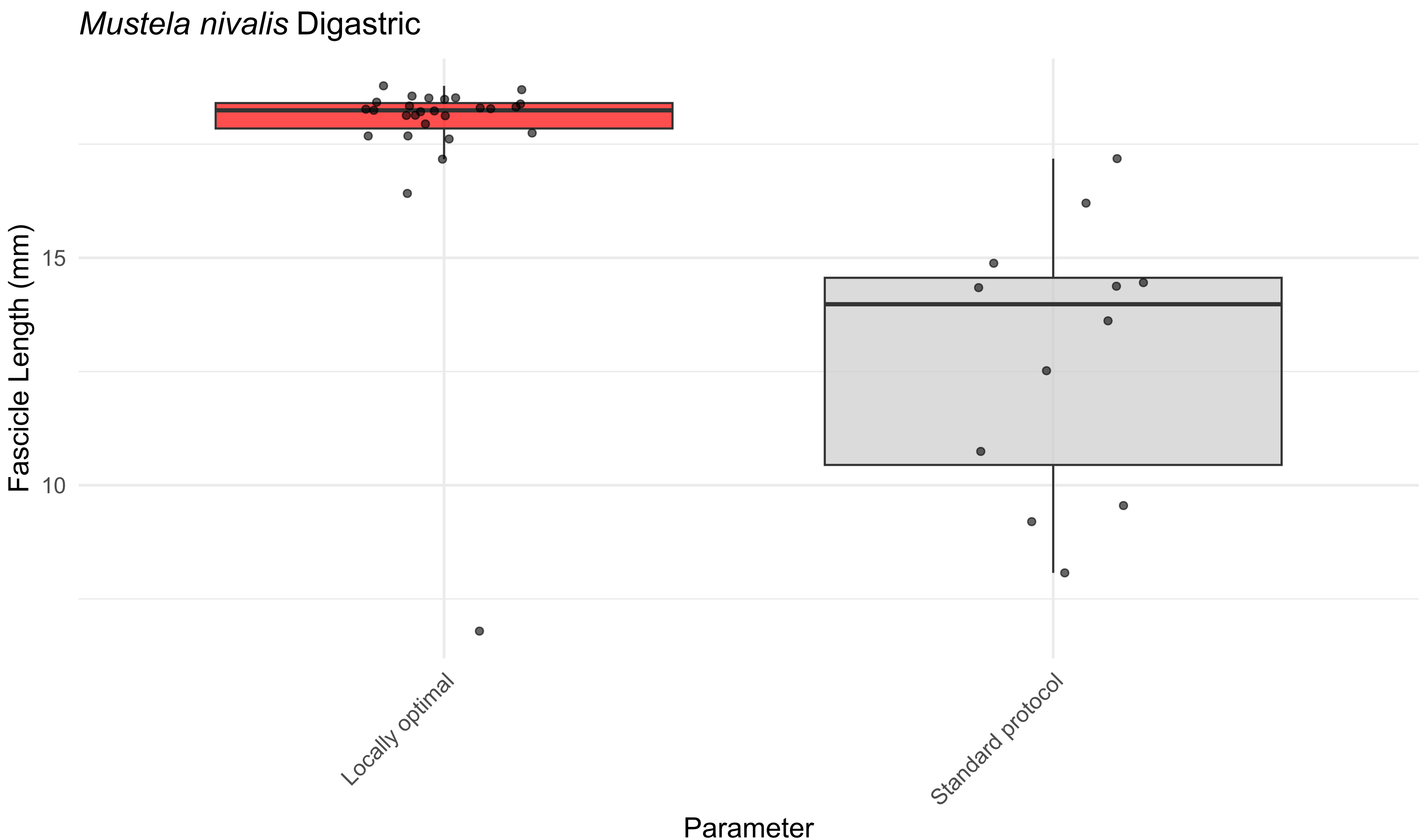

Mustela nivalis Digastric
Fascicle Length (mm)
15
10
Locally optimal
Standard protocol
Parameter

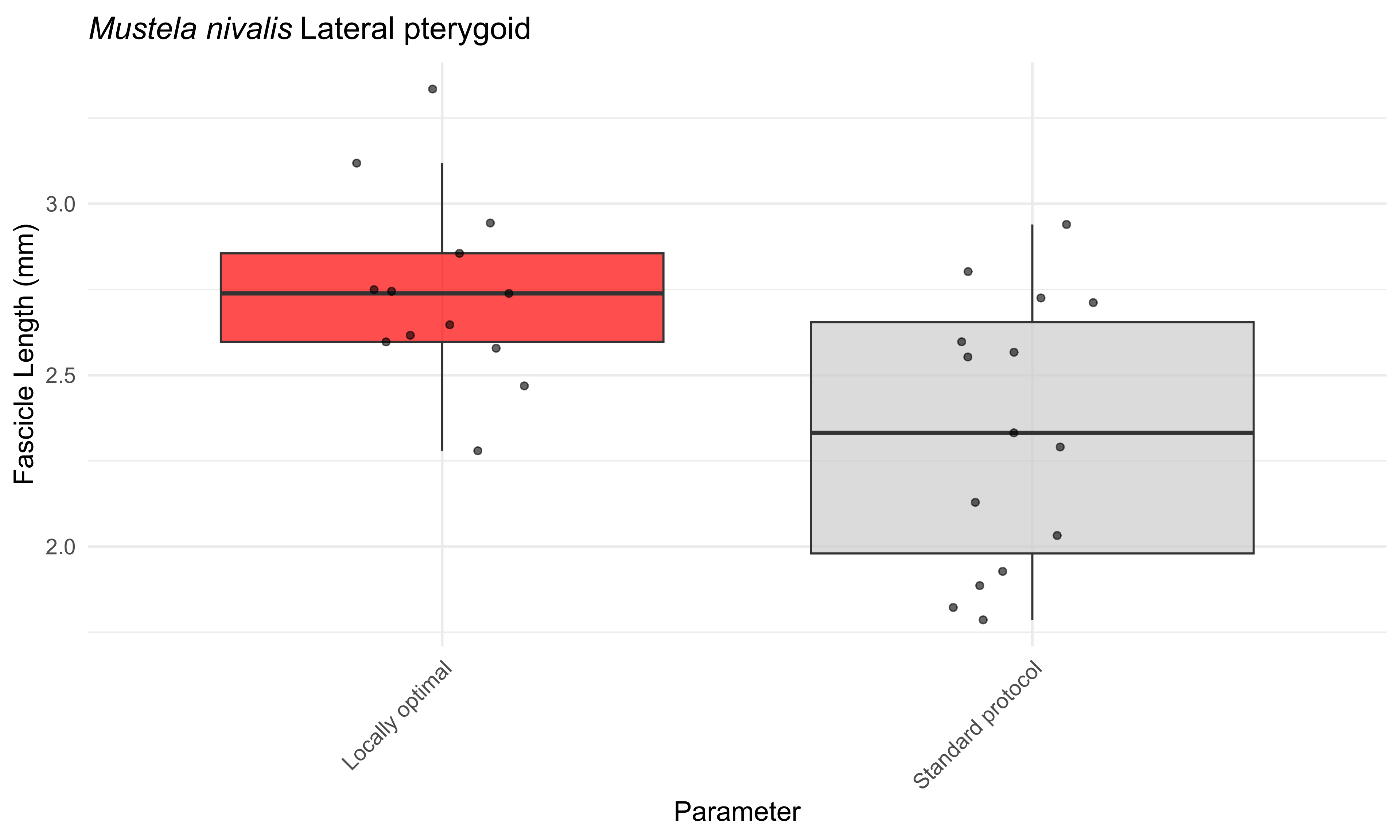
Mustela nivalis Lateral pterygoid
Fascicle Length (mm)
3.0
2.5
2.0
Locally optimal
Standard protocol
Parameter

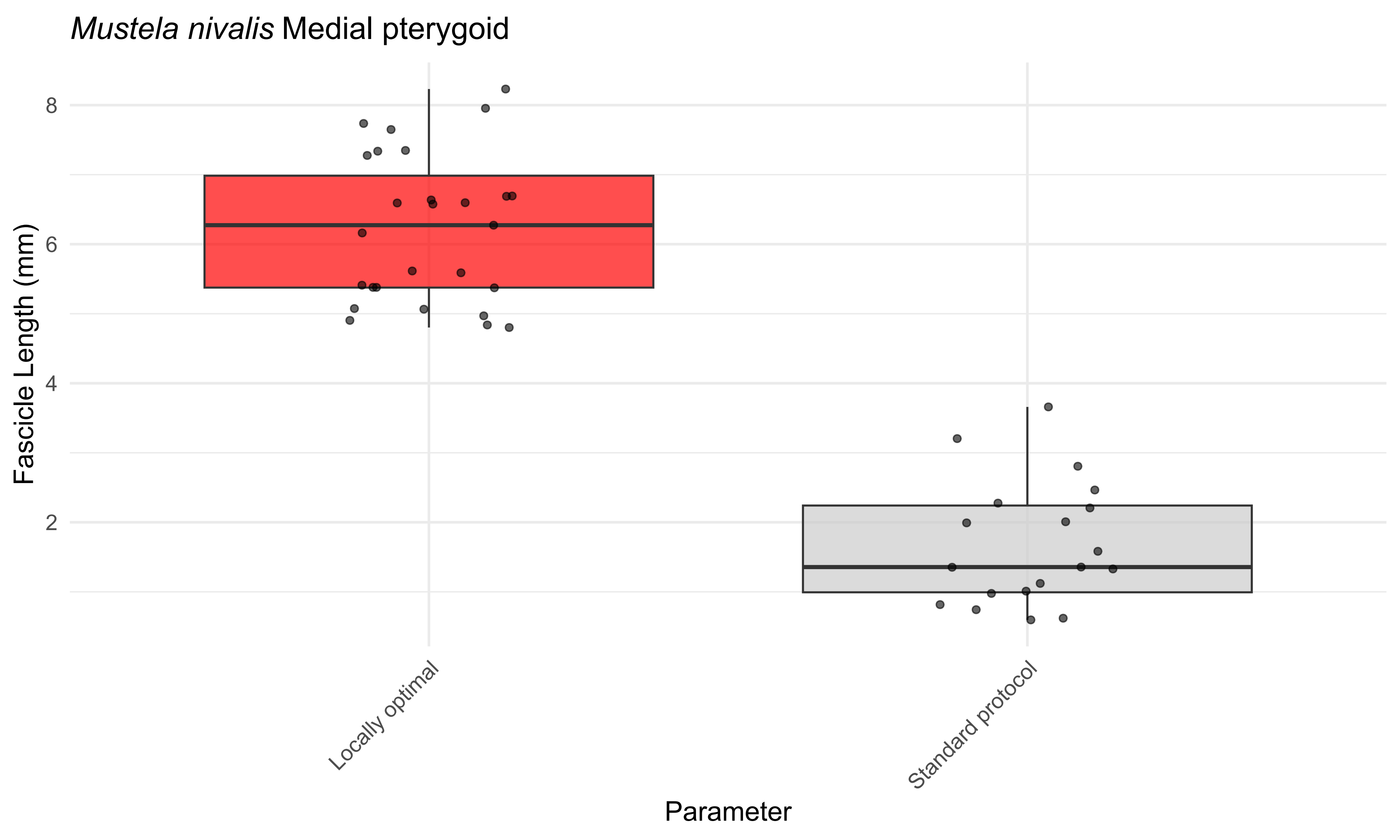

Mustela nivalis Medial pterygoid
Fascicle Length (mm)
8
6
4
2
Locally optimal
Standard protocol
Parameter

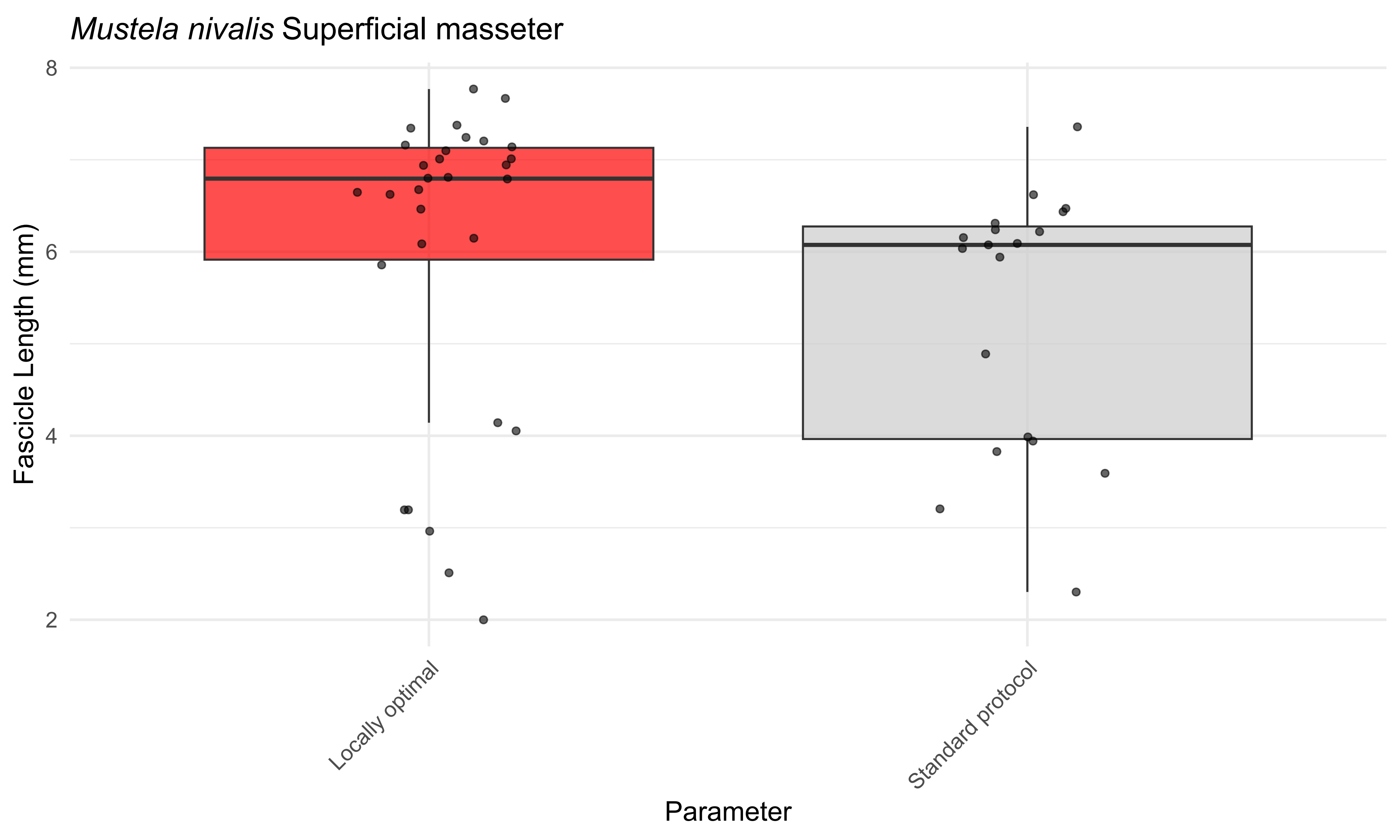
Mustela nivalis Superficial masseter
Fascicle Length (mm)
8
6
4
2
Locally optimal
Standard protocol
Parameter

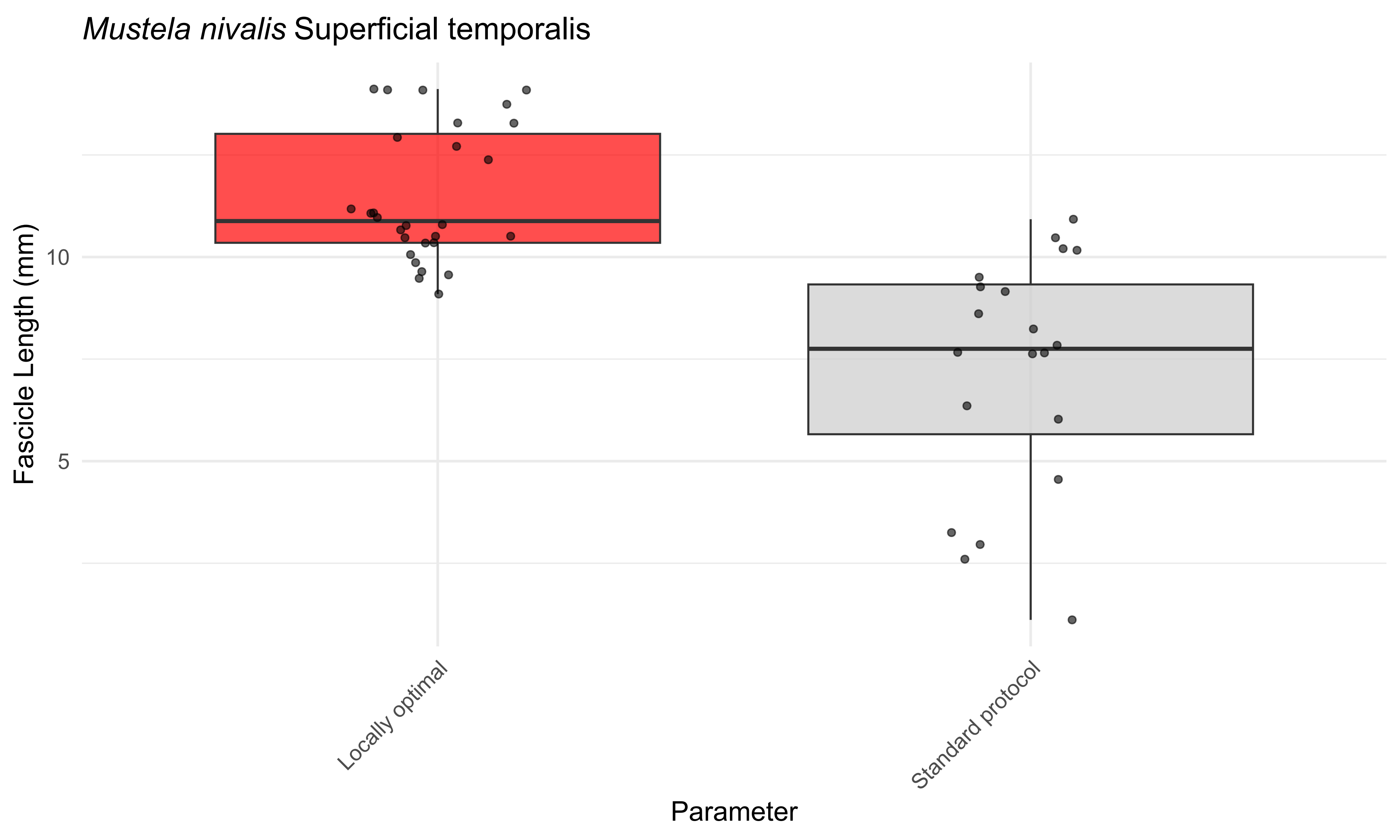

Mustela nivalis Superficial temporalis
Fascicle Length (mm)
10
5
Locally optimal
Standard protocol
Parameter

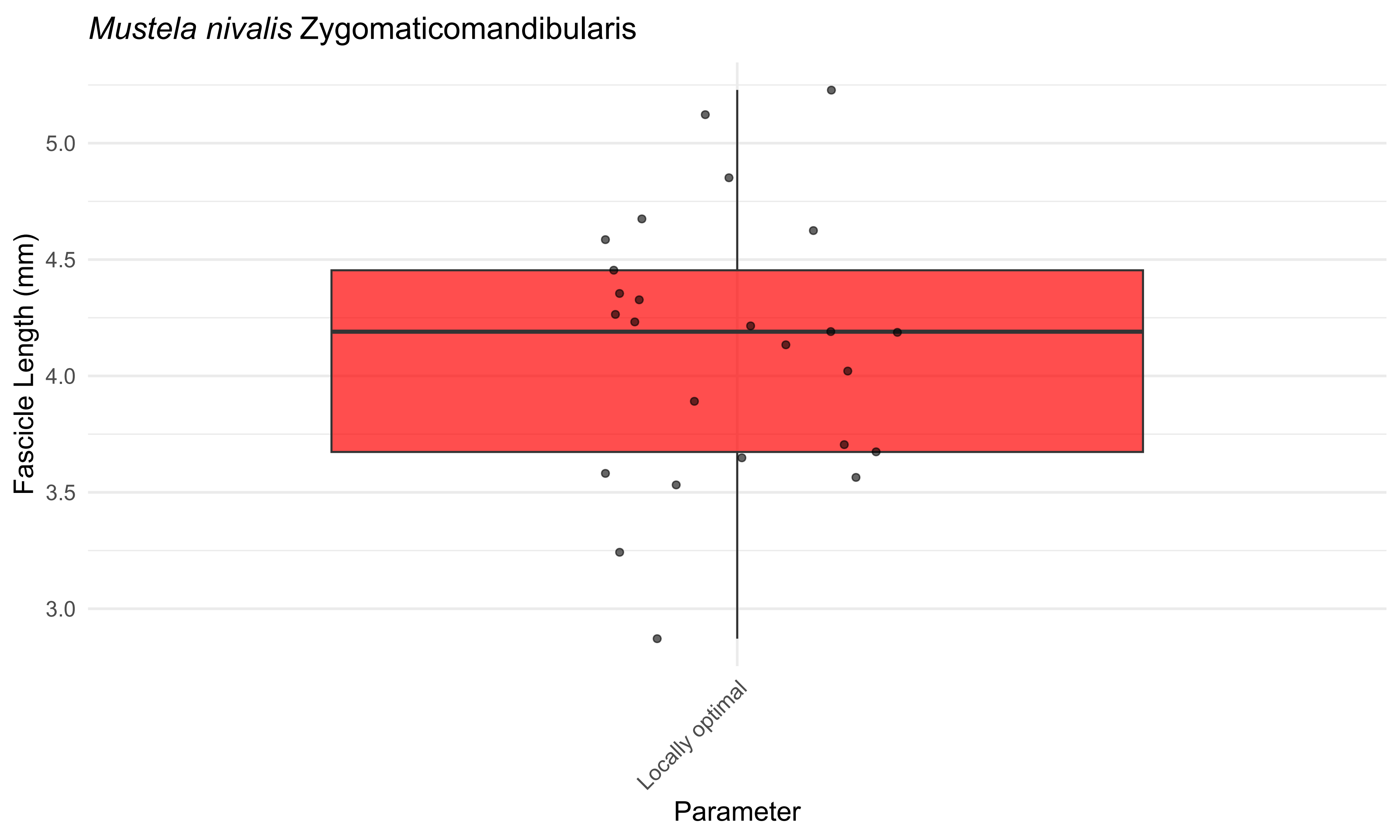
Mustela nivalis Zygomaticomandibularis
Fascicle Length (mm)
5.0
4.5
4.0
3.5
3.0
Locally optimal
Parameter

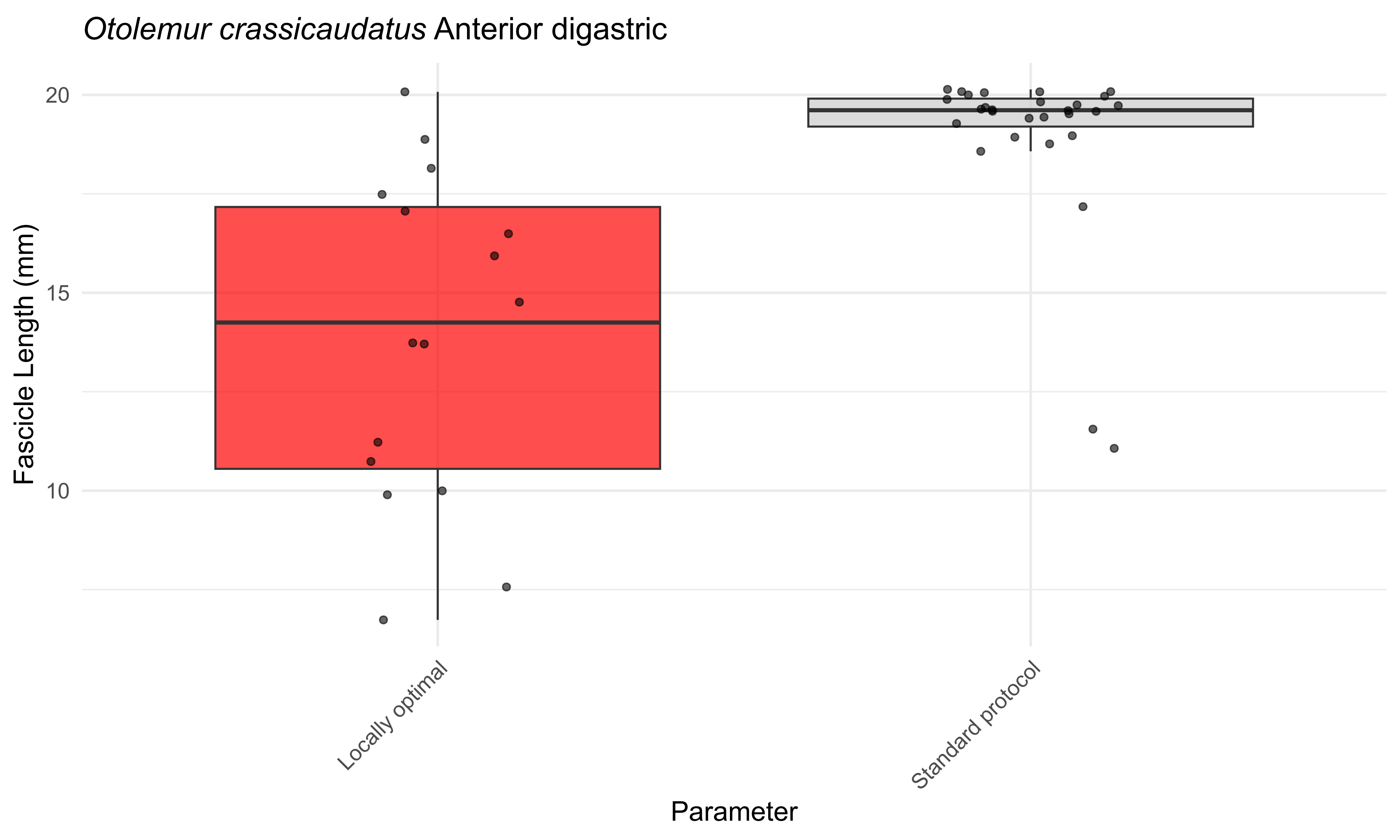

Otolemur crassicaudatus Anterior digastric
Fascicle Length (mm)
20
15
10
Locally optimal
Standard protocol
Parameter

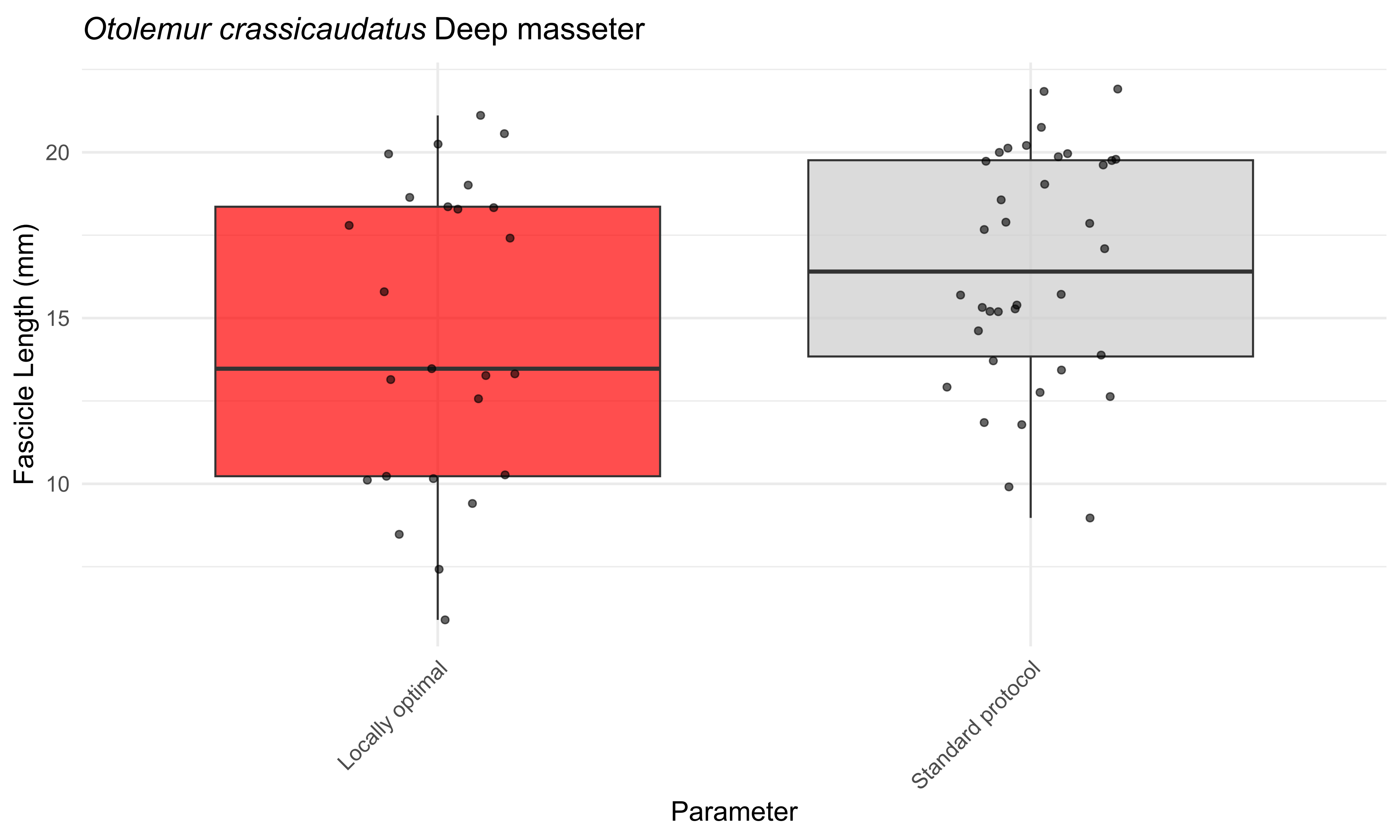

Otolemur crassicaudatus Deep masseter
Fascicle Length (mm)
20
15
10
Locally optimal
Standard protocol
Parameter

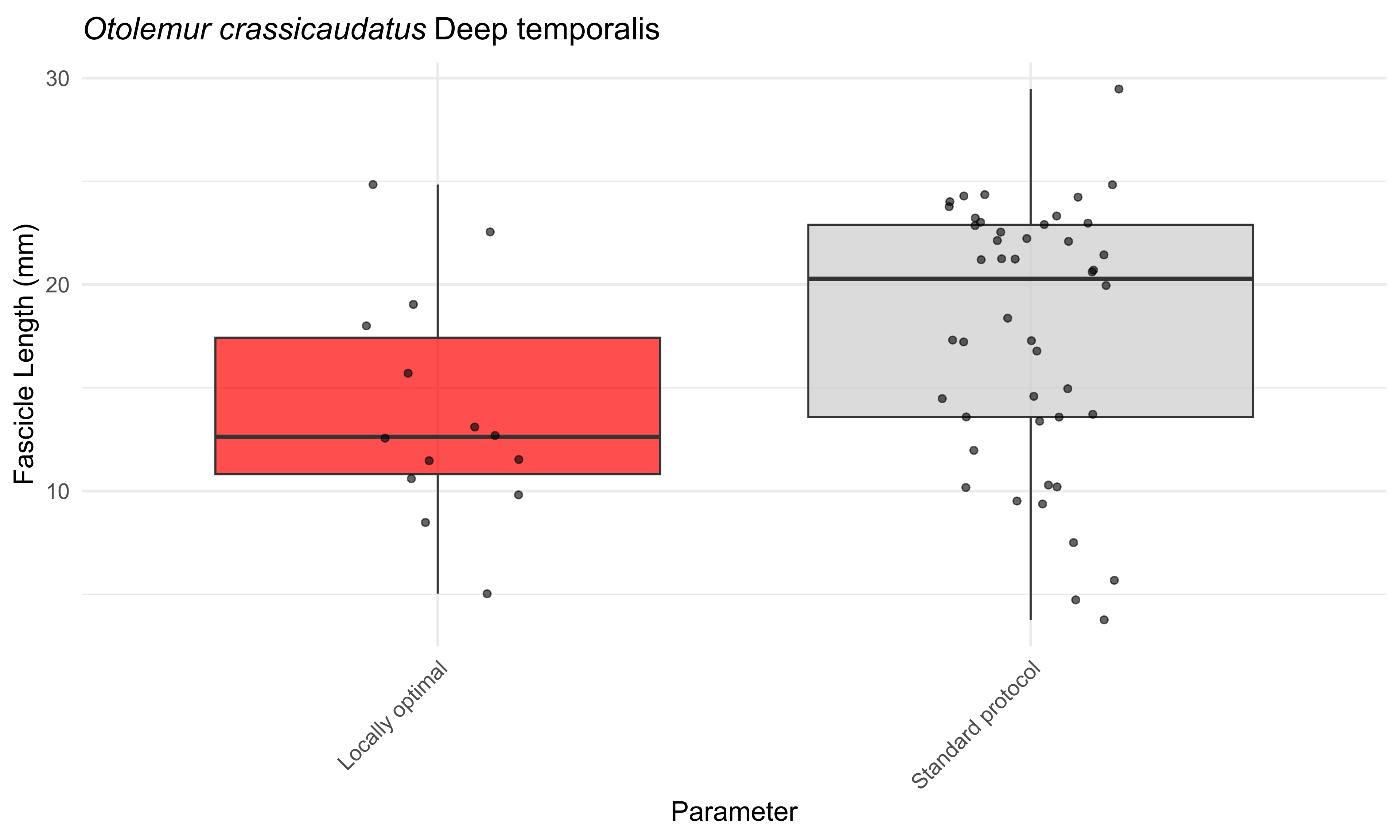
Otolemur crassicaudatus Deep temporalis
Fascicle Length (mm)
30
20
10
Locally optimal
Standard protocol
Parameter

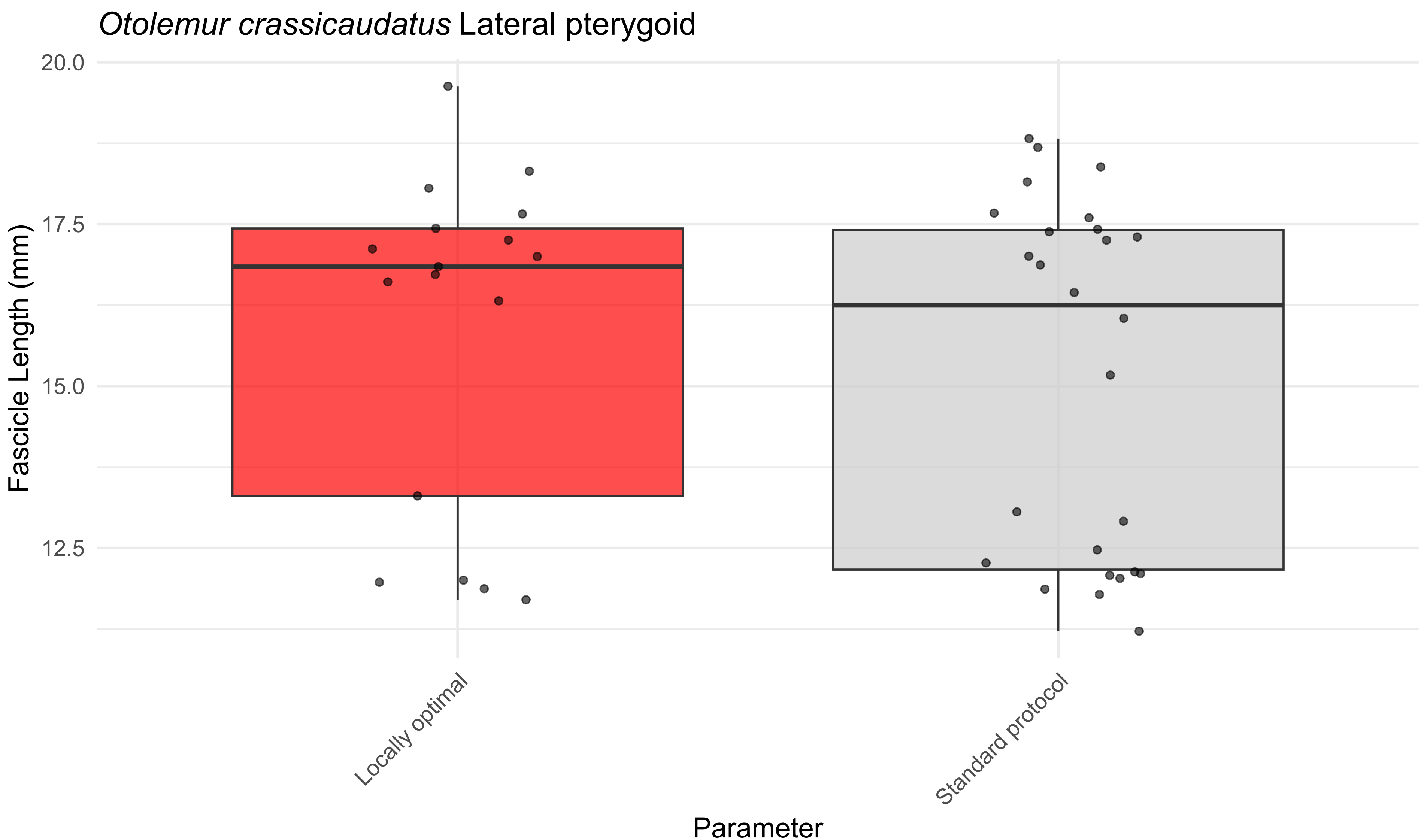

Otolemur crassicaudatus Lateral pterygoid
20.0
17.5
15.0
12.5
Fascicle Length (mm)
Locally optimal
Standard protocol
Parameter

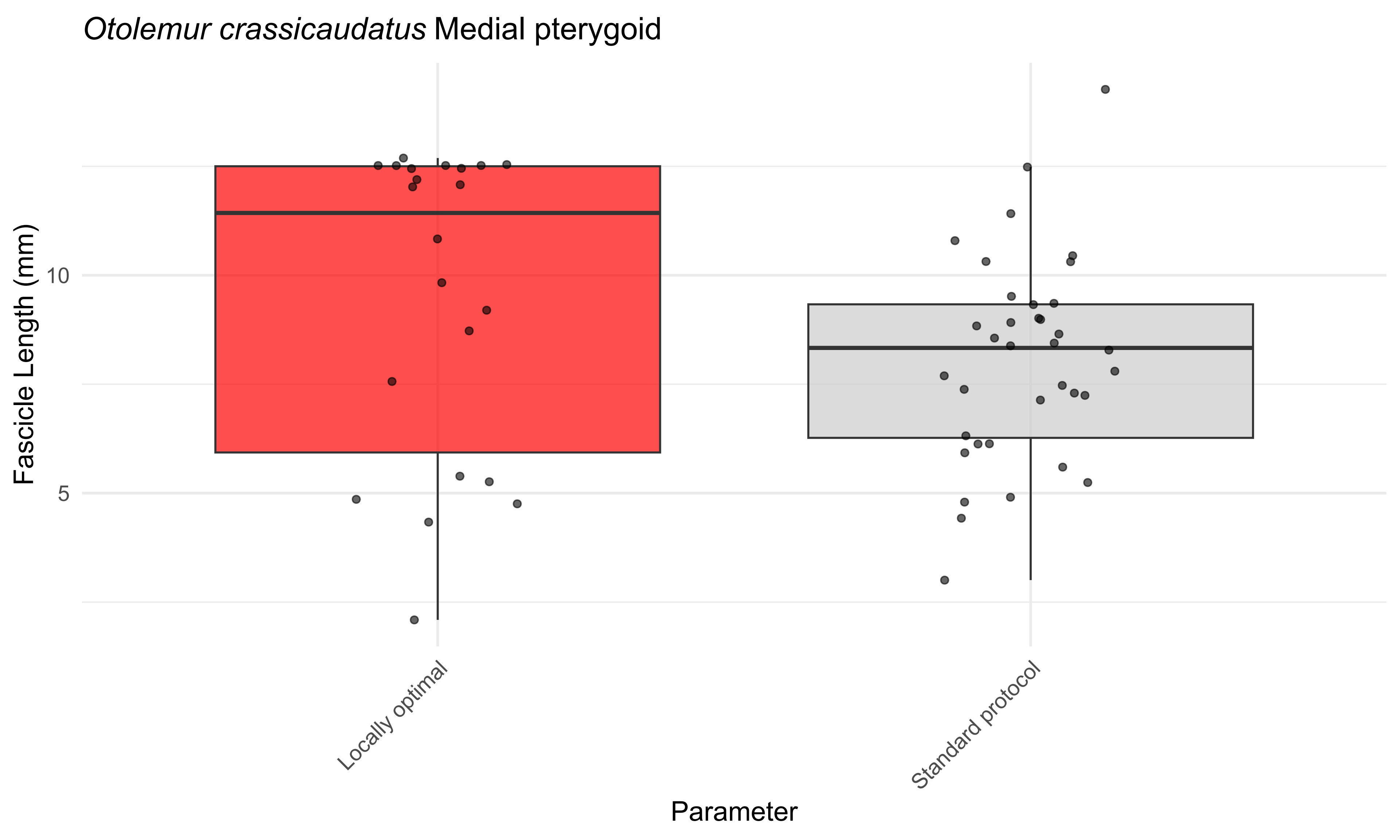

Otolemur crassicaudatus Medial pterygoid
Fascicle Length (mm)
10
5
Locally optimal
Standard protocol
Parameter

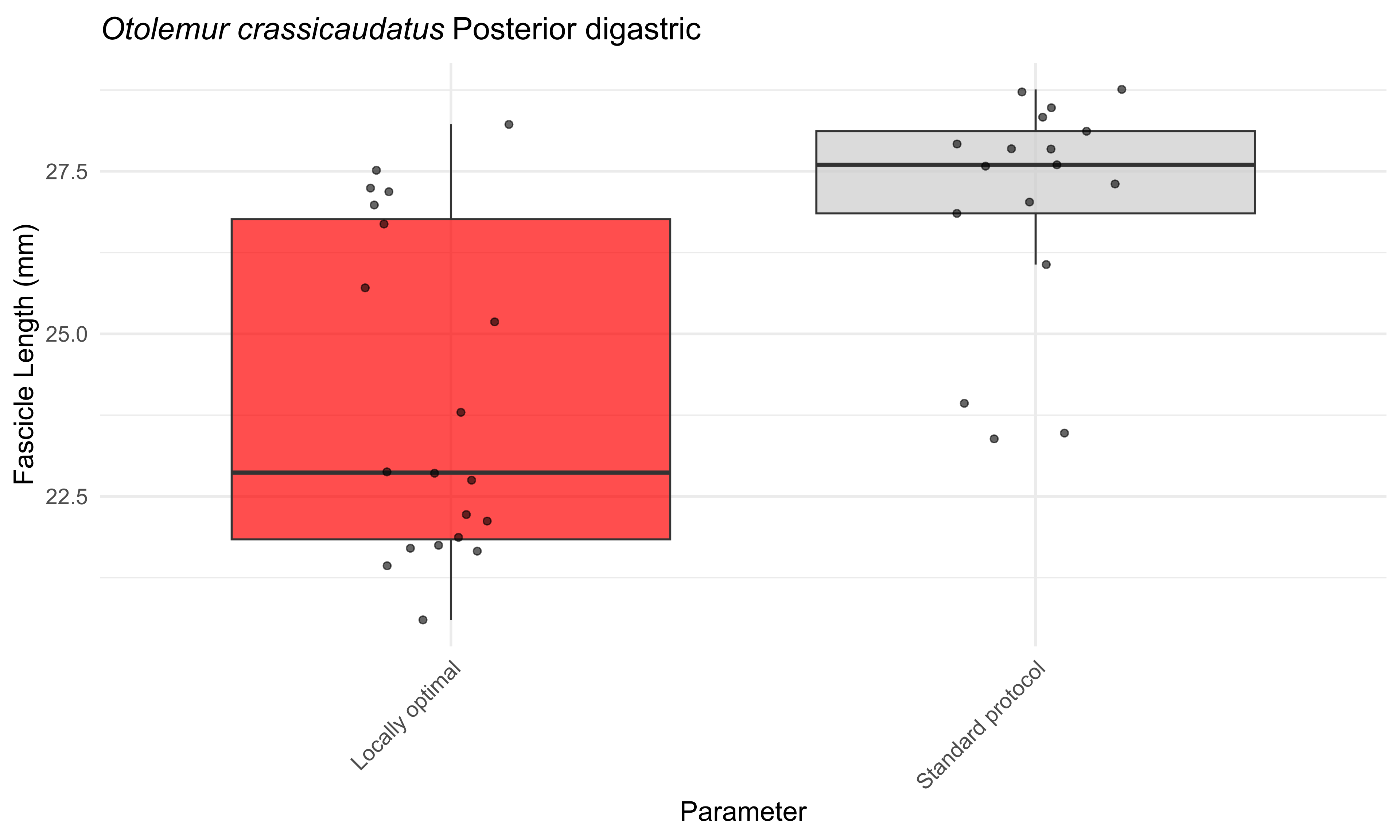

Otolemur crassicaudatus Posterior digastric
Fascicle Length (mm)
27.5
25.0
22.5
Locally optimal
Standard protocol
Parameter

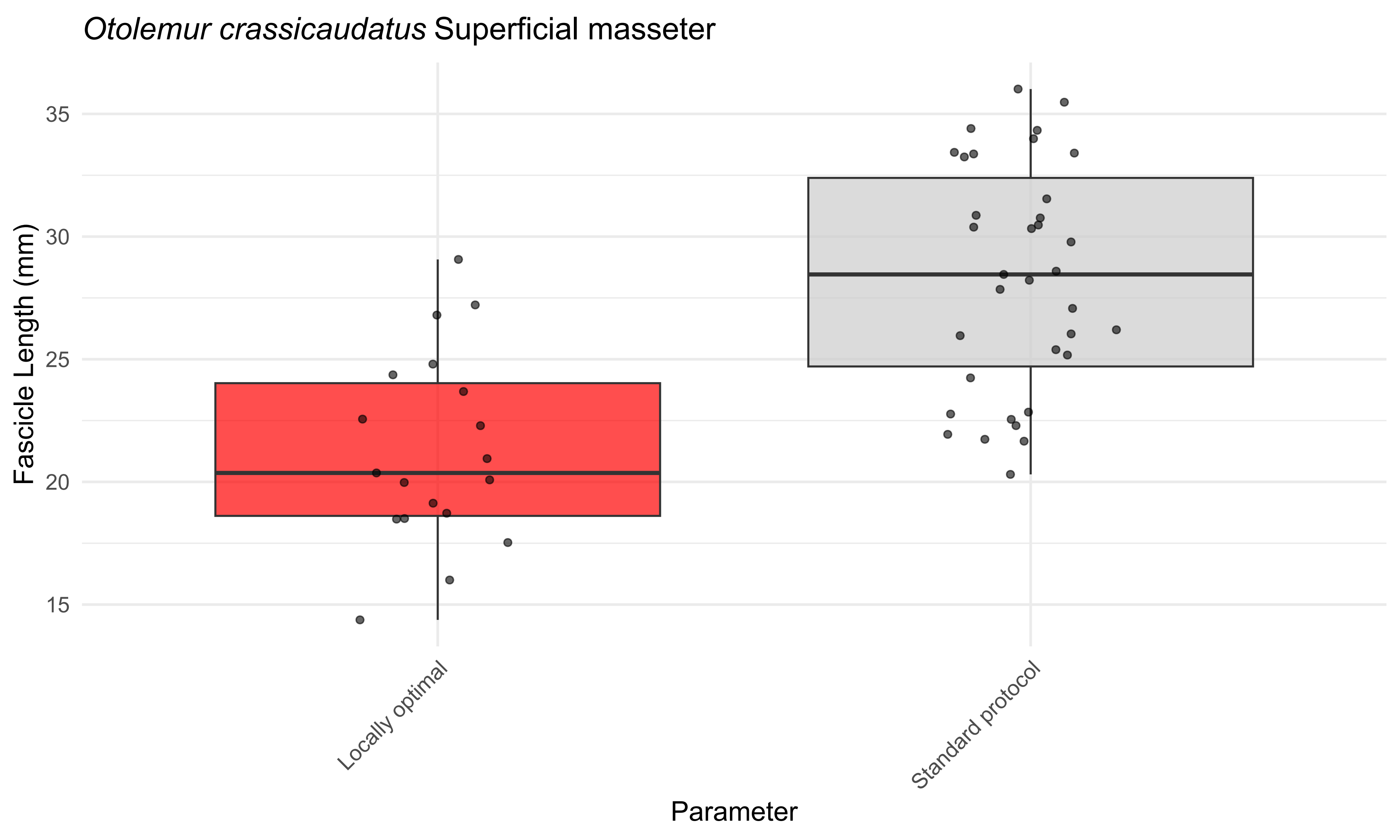

Otolemur crassicaudatus Superficial masseter
Fascicle Length (mm)
35
30
25
20
15
Locally optimal
Standard protocol
Parameter

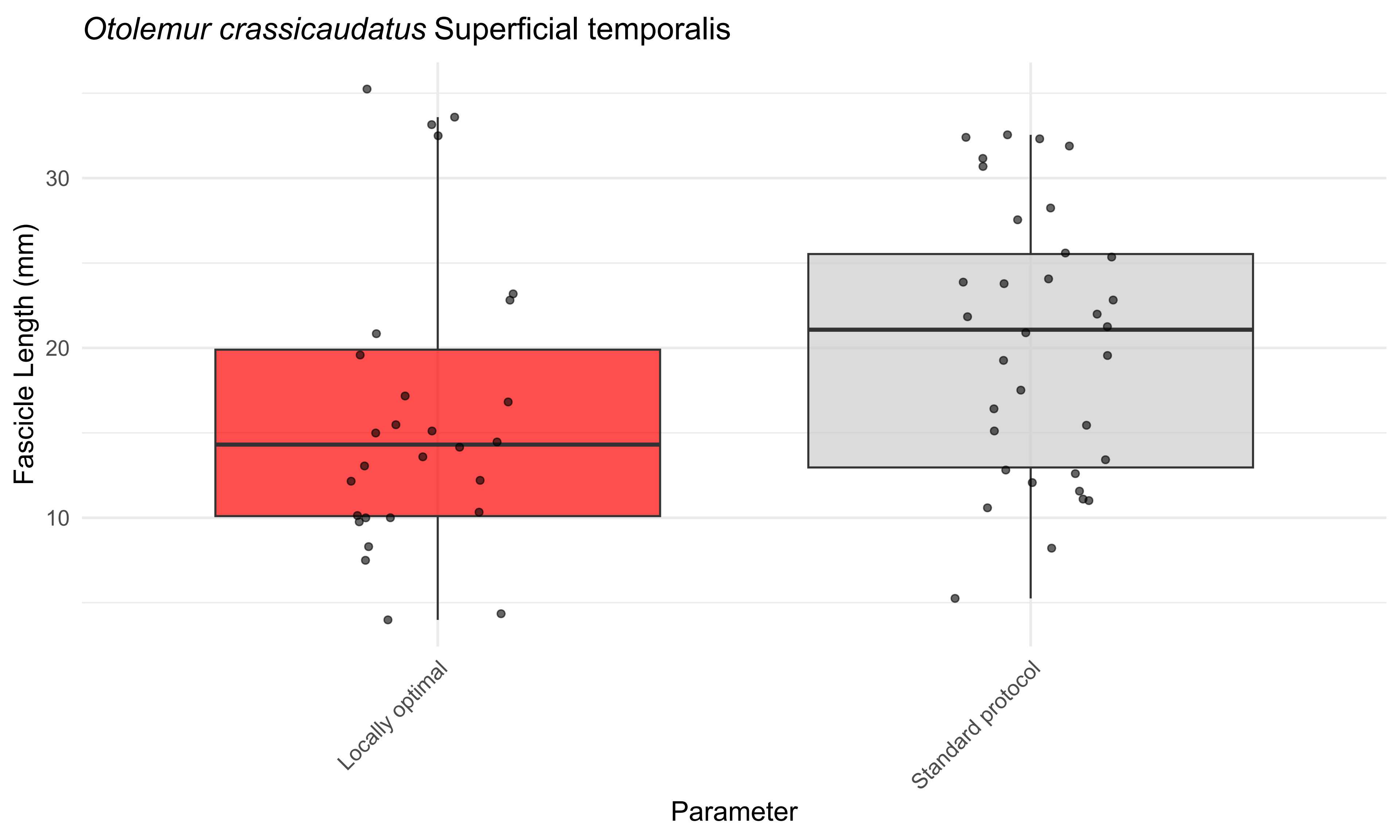
Otolemur crassicaudatus Superficial temporalis
Fascicle Length (mm)
30
20
10
Locally optimal
Standard protocol
Parameter

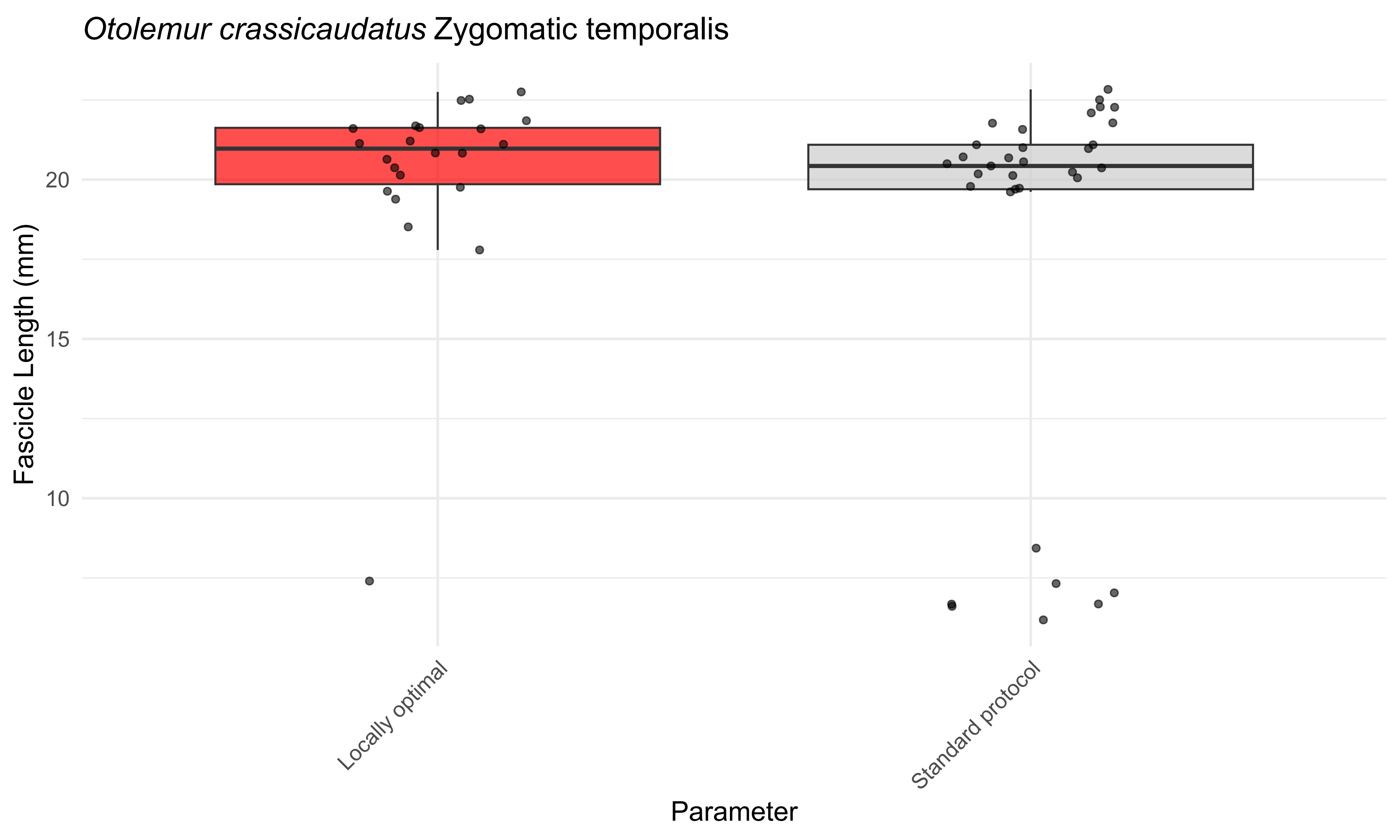

Otolemur crassicaudatus Zygomatic temporalis
Fascicle Length (mm)
20
15
10
Locally optimal
Standard protocol
Parameter

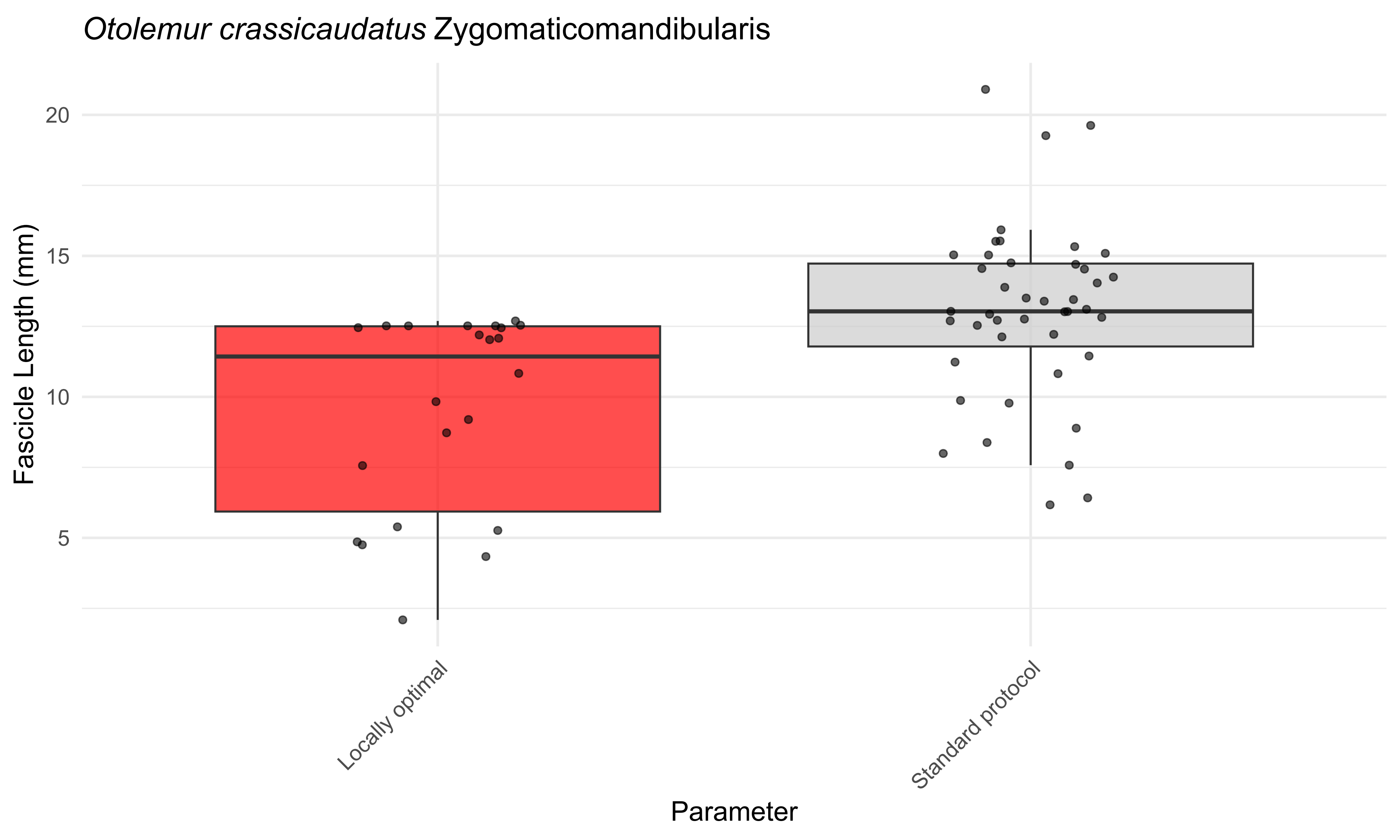

Otolemur crassicaudatus Zygomaticomandibularis
Fascicle Length (mm)
20
15
10
5
Locally optimal
Standard protocol
Parameter

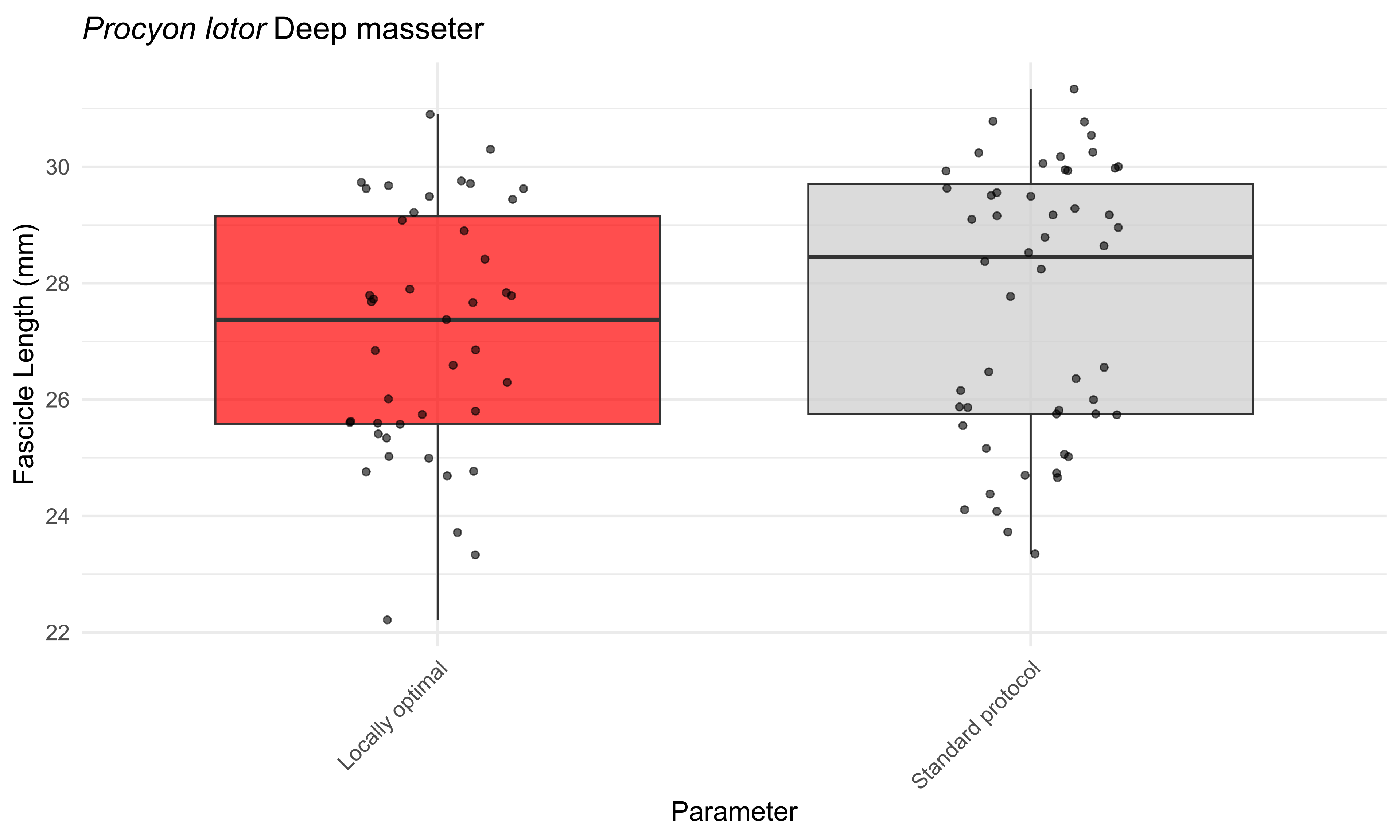

Procyon lotor Deep masseter
Fascicle Length (mm)
30
28
26
24
22
Locally optimal
Standard protocol
Parameter

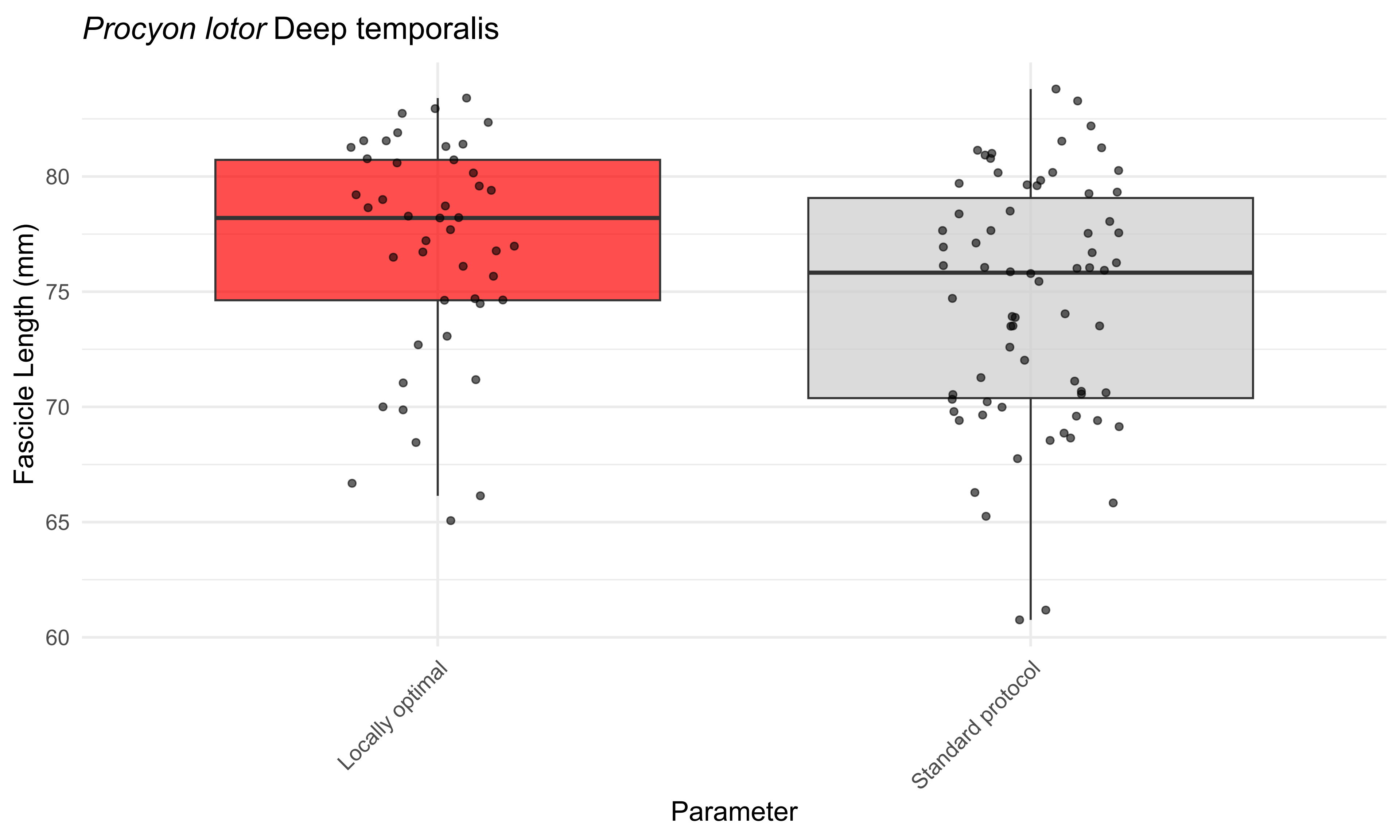
Procyon lotor Deep temporalis
Fascicle Length (mm)
60
65
70
75
80
Locally optimal
Standard protocol
Parameter

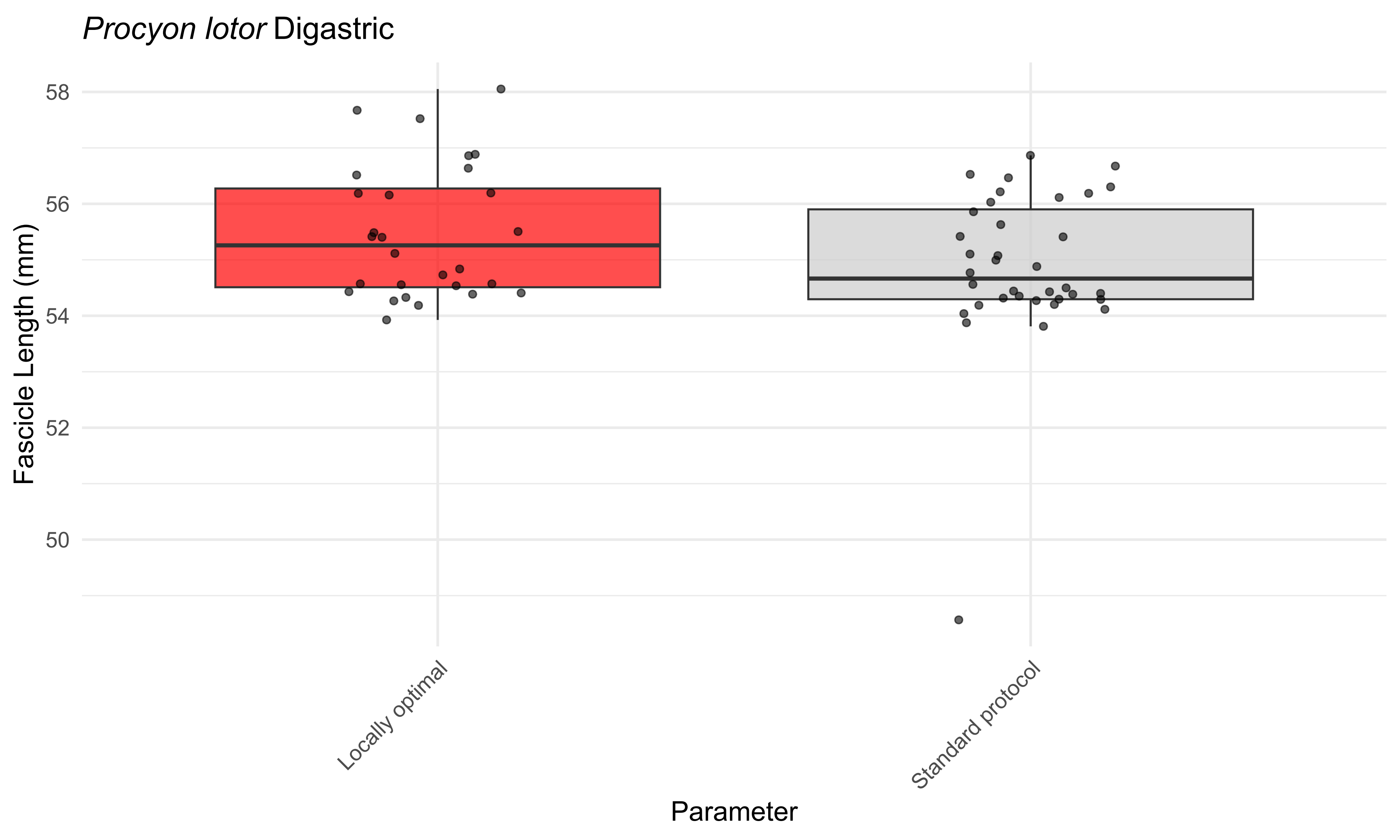

Procyon lotor Digastric
Fascicle Length (mm)
58
56
54
52
50
Locally optimal
Standard protocol
Parameter

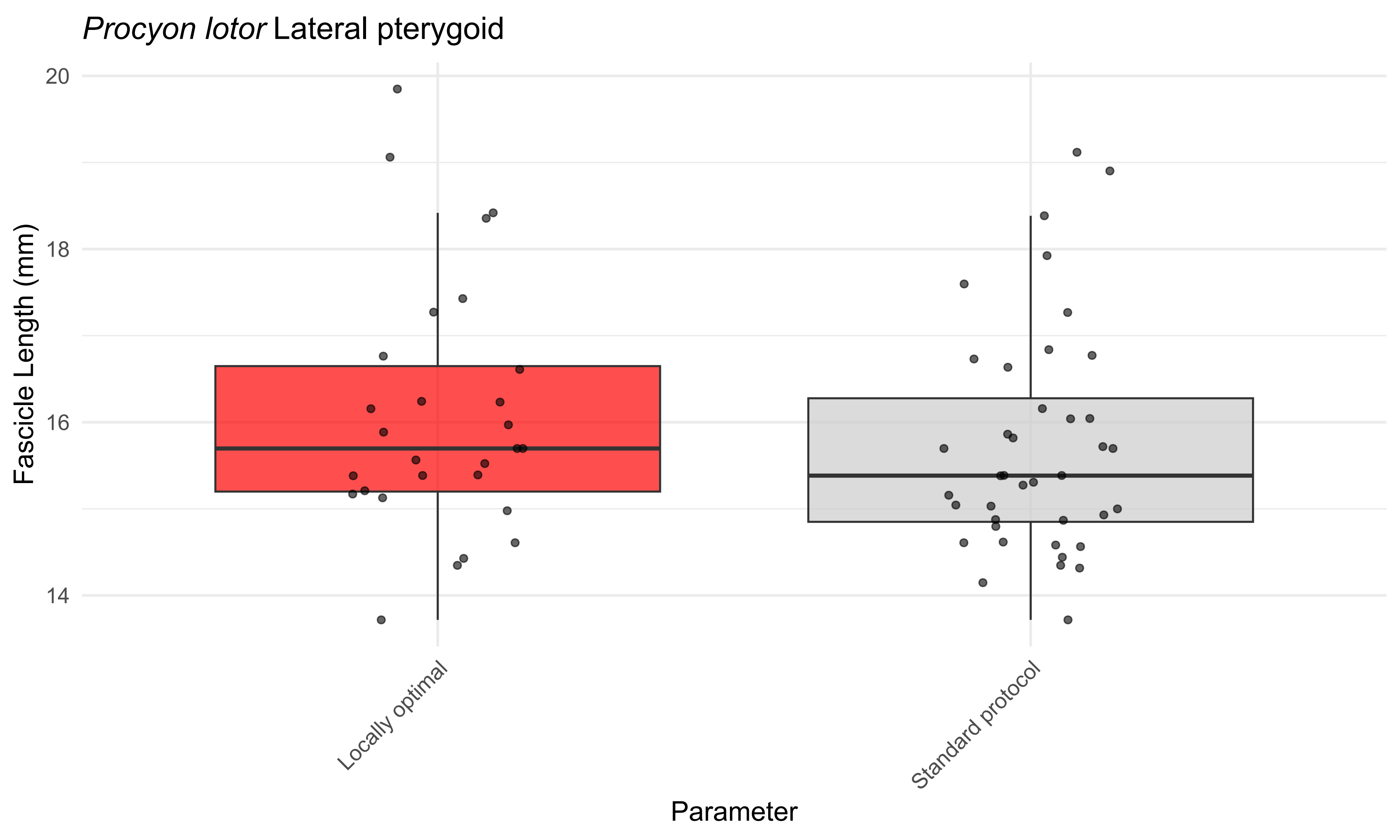

Procyon lotor Lateral pterygoid
Fascicle Length (mm)
20
18
16
14
Locally optimal
Standard protocol
Parameter

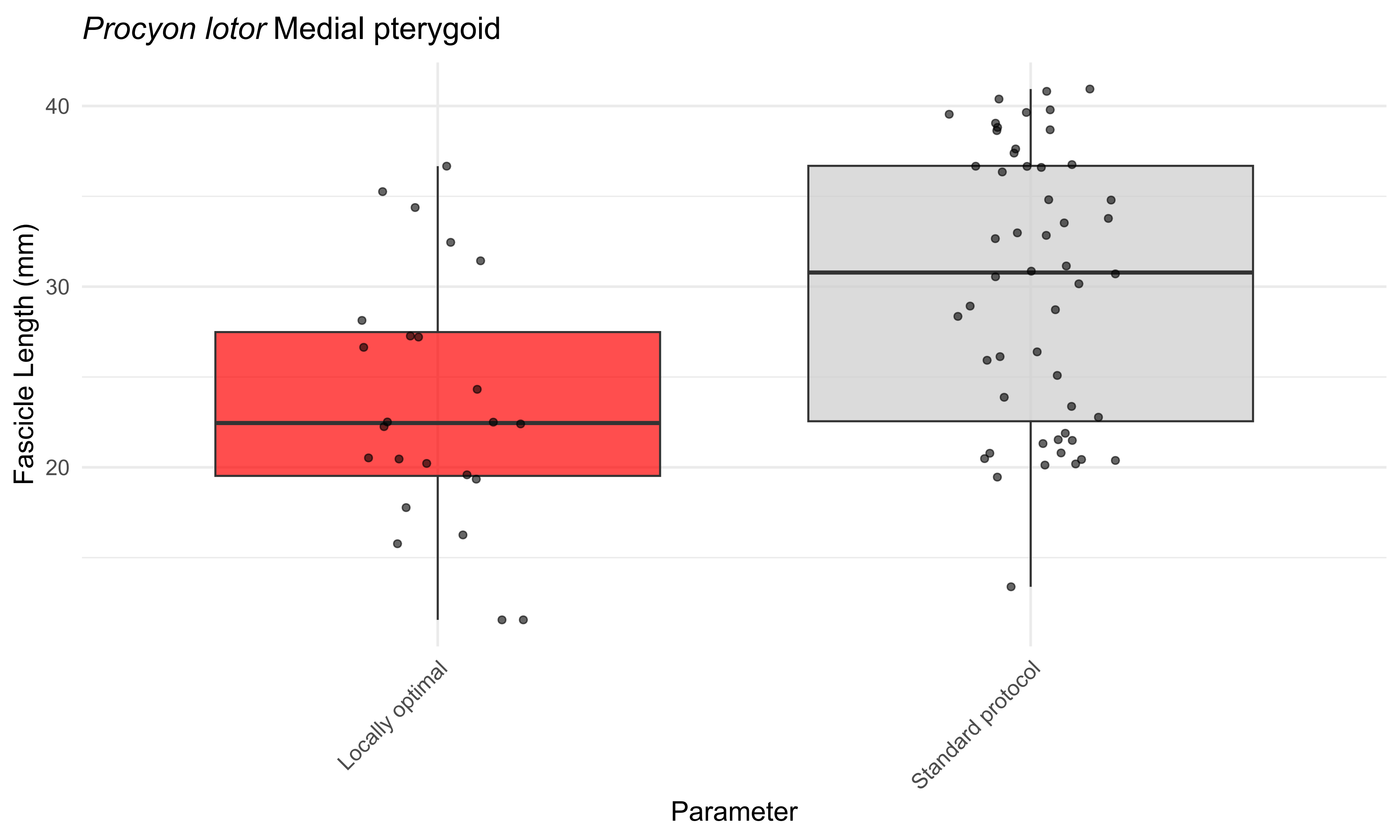

Procyon lotor Medial pterygoid
Fascicle Length (mm)
40
30
20
Locally optimal
Standard protocol
Parameter

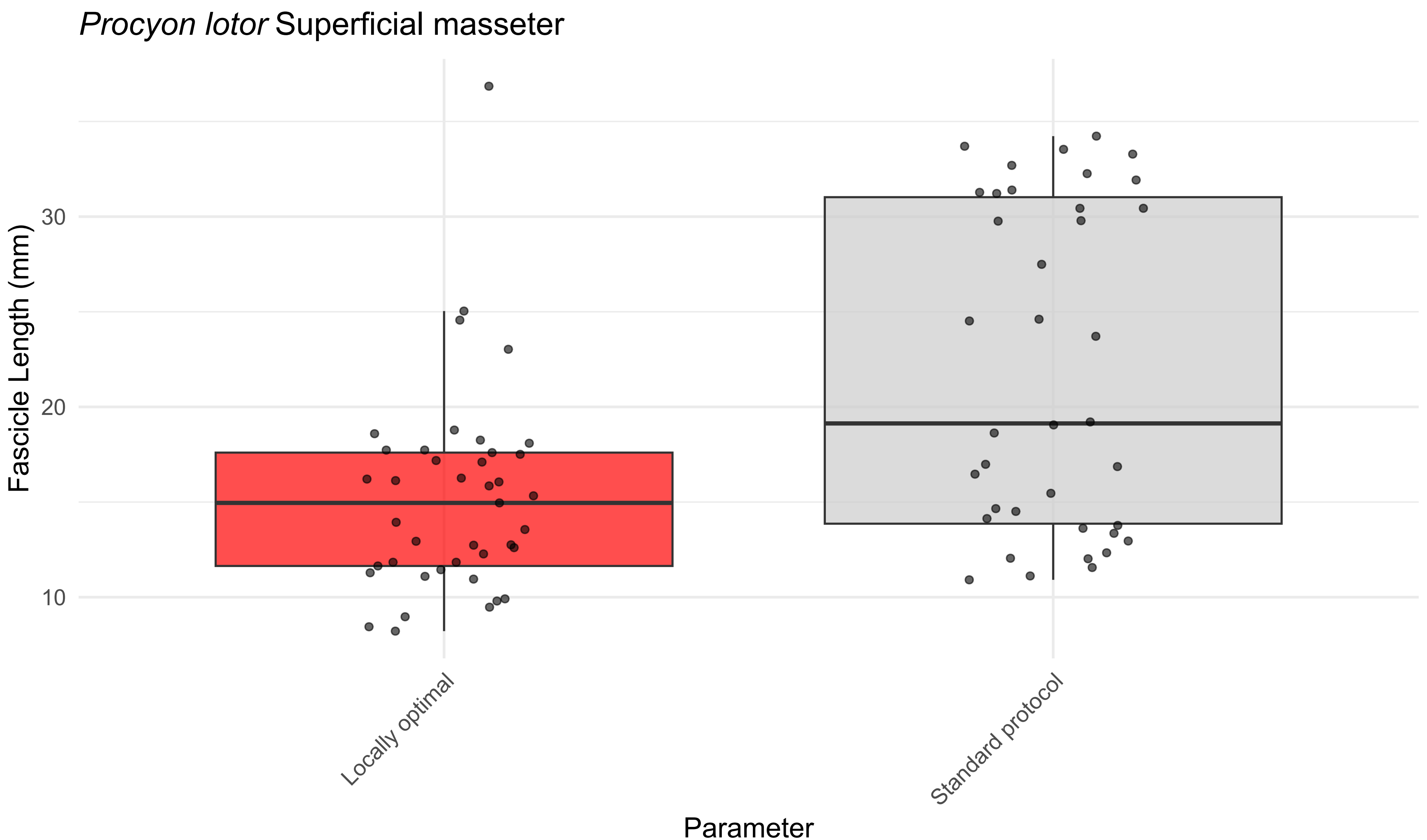
Procyon lotor Superficial masseter
Fascicle Length (mm)
30
20
10
Locally optimal
Standard protocol
Parameter

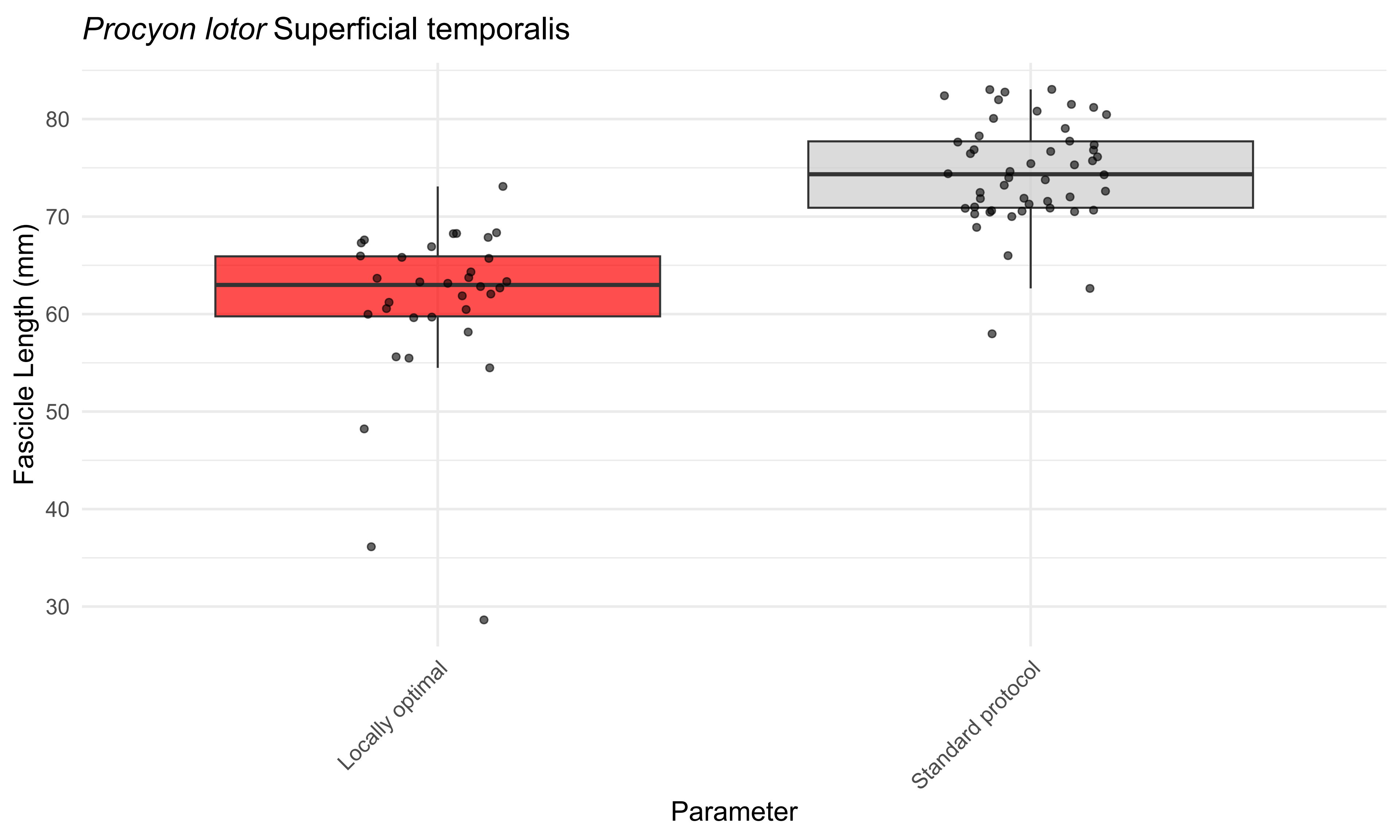

Procyon lotor Superficial temporalis
Fascicle Length (mm)
80
70
60
50
40
30
Locally optimal
Standard protocol
Parameter

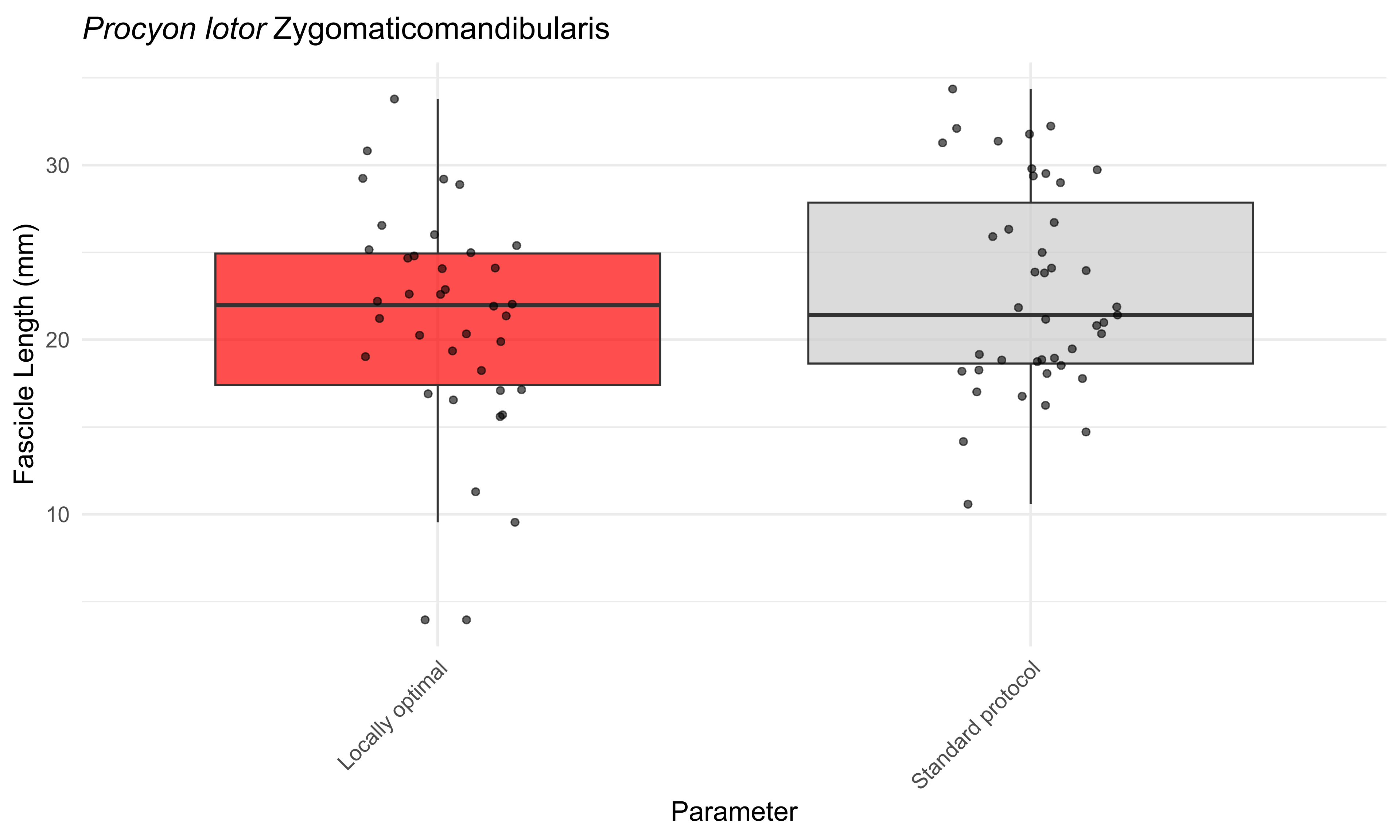
Procyon lotor Zygomaticomandibularis
Fascicle Length (mm)
30
20
10
Locally optimal
Standard protocol
Parameter
