# Supplementary material for: Multi‐method analysis for the three‐dimensional reconstruction of muscle fascicles from DiceCT datasets
Source: Anat Rec (Hoboken). 2025 Dec 4;309(12):3491–510. doi: 10.1002/ar.70088 (PMC13749328; doi:10.1002/ar.70088)
Supplement: Supplementary file 6 [file AR-309-3491-s009.pdf]

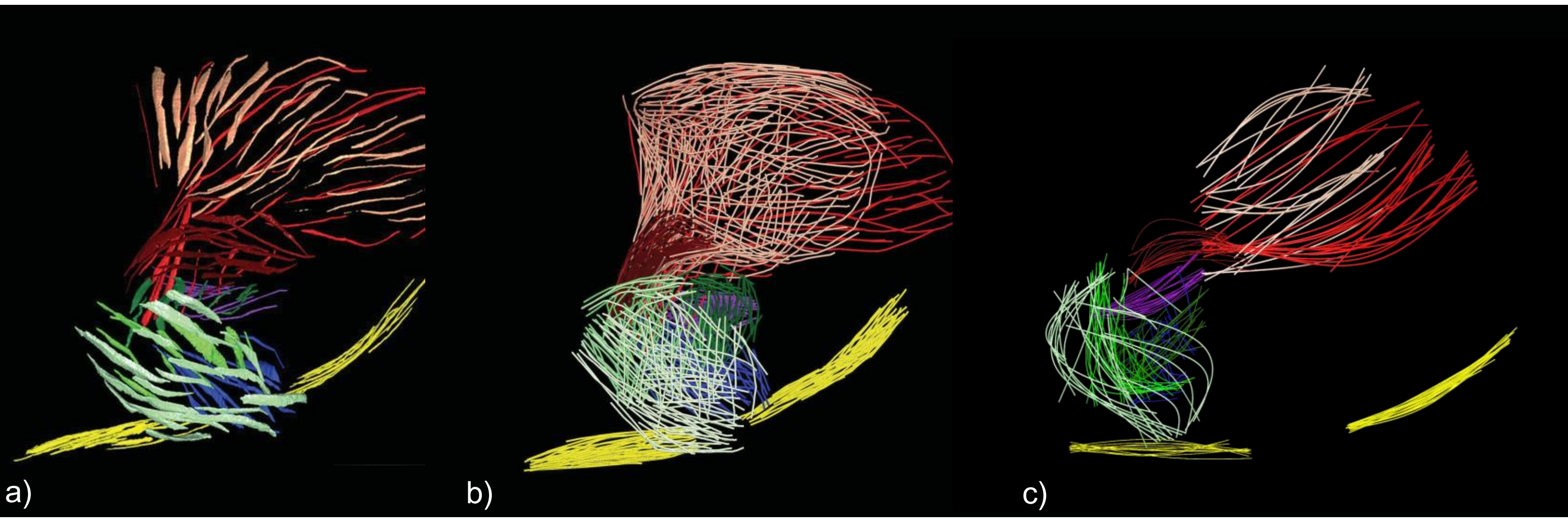
a)
b)
c)
