# Supplementary material for: Multi‐method analysis for the three‐dimensional reconstruction of muscle fascicles from DiceCT datasets
Source: Anat Rec (Hoboken). 2025 Dec 4;309(12):3491–510. doi: 10.1002/ar.70088 (PMC13749328; doi:10.1002/ar.70088)
Supplement: Supplementary file 7 [file AR-309-3491-s005.docx]

**SUPPLEMENTAL TABLES**

**Supplemental Table 1. Statistical results derived from comparisons of fiber length and tortuosity generated from the various XFiber parameters.** Each box provides the *t*-value; degrees of freedom; *p*-value; and confidence interval (minimum – maximum). Any missing comparisons mean that fascicles that were not generated. The abbreviations represent the following: Min. Seed Corr. +50% - Minimum Seed Correlation 50% greater than optimal; Min. Seed Corr. −50% - Minimum Seed Correlation 50% lower than optimal; Min. Cont. Qual. +50% - Minimum Continuation Quality 50% greater than optimal; Min. Cont. Qual. −50% - Minimum Continuation Quality 50% lower than optimal; Dir. Coeff. 0.3 - Direction Coefficient set to 0.3; Dir. Coeff. 0.2 - Direction Coefficient set to 0.2; Dir. Coeff. 0.1 - Direction Coefficient set to 0.1; 100% Cyl. Length - 100% of Cylinder Length value used; 50% Cyl. Length - 50% of Cylinder Length value used.

| Species | Muscle | Measure | Parameter_Compared | N_LocallyOptimal | N_Compared | Mean_LocallyOptimal | Mean_Compared | t_statistic | df | p_value |
| --- | --- | --- | --- | --- | --- | --- | --- | --- | --- | --- |
| Eulemur coronatus | Anterior digastric | Fascicle Length (mm) | Minimum Seed Correlation +50% | 41 | 15 | 10.47 | 13.45 | -2.17 | 25 | 0.040 |
| Eulemur coronatus | Anterior digastric | Fascicle Length (mm) | Minimum Seed Correlation -50% | 41 | 45 | 10.47 | 9.99 | 0.49 | 83.4 | 0.625 |
| Eulemur coronatus | Anterior digastric | Fascicle Length (mm) | Minimum Continuation Quality +50% | 41 | 25 | 10.47 | 8.41 | 2.11 | 61.8 | 0.039 |
| Eulemur coronatus | Anterior digastric | Fascicle Length (mm) | Minimum Continuation Quality -50% | 41 | 42 | 10.47 | 12.79 | -2.14 | 79.7 | 0.036 |
| Eulemur coronatus | Anterior digastric | Fascicle Length (mm) | Direction Coefficient 0.2 | 41 | 39 | 10.47 | 10.05 | 0.43 | 77.9 | 0.672 |
| Eulemur coronatus | Anterior digastric | Fascicle Length (mm) | Direction Coefficient 0.1 | 41 | 19 | 10.47 | 9.29 | 0.96 | 36.5 | 0.344 |
| Eulemur coronatus | Anterior digastric | Fascicle Length (mm) | Minimum Length 100% Cylinder Length | 41 | 25 | 10.47 | 13.06 | -2.51 | 58.1 | 0.015 |
| Eulemur coronatus | Anterior digastric | Fascicle Length (mm) | Minimum Length 50% Cylinder Length | 41 | 47 | 10.47 | 9.76 | 0.73 | 84.9 | 0.470 |
| Eulemur coronatus | Deep masseter | Fascicle Length (mm) | Minimum Seed Correlation +50% | 52 | 5 | 8.26 | 11.26 | -1.80 | 4.5 | 0.137 |
| Eulemur coronatus | Deep masseter | Fascicle Length (mm) | Minimum Seed Correlation -50% | 52 | 55 | 8.26 | 7.67 | 1.03 | 104.9 | 0.305 |
| Eulemur coronatus | Deep masseter | Fascicle Length (mm) | Minimum Continuation Quality +50% | 52 | 30 | 8.26 | 6.27 | 3.93 | 80 | 0.000 |
| Eulemur coronatus | Deep masseter | Fascicle Length (mm) | Minimum Continuation Quality -50% | 52 | 45 | 8.26 | 11.25 | -2.96 | 60 | 0.004 |
| Eulemur coronatus | Deep masseter | Fascicle Length (mm) | Direction Coefficient 0.2 | 52 | 49 | 8.26 | 7.13 | 2.22 | 94.9 | 0.029 |
| Eulemur coronatus | Deep masseter | Fascicle Length (mm) | Direction Coefficient 0.1 | 52 | 26 | 8.26 | 6.58 | 2.97 | 67.2 | 0.004 |
| Eulemur coronatus | Deep masseter | Fascicle Length (mm) | Minimum Length 100% Cylinder Length | 52 | 35 | 8.26 | 9.06 | -1.31 | 74.7 | 0.196 |
| Eulemur coronatus | Deep masseter | Fascicle Length (mm) | Minimum Length 50% Cylinder Length | 52 | 56 | 8.26 | 7.25 | 1.71 | 105.9 | 0.091 |
| Eulemur coronatus | Deep temporalis | Fascicle Length (mm) | Minimum Seed Correlation +50% | 58 | 14 | 13.52 | 17.30 | -1.32 | 16.9 | 0.205 |
| Eulemur coronatus | Deep temporalis | Fascicle Length (mm) | Minimum Seed Correlation -50% | 58 | 61 | 13.52 | 13.27 | 0.18 | 116.5 | 0.859 |
| Eulemur coronatus | Deep temporalis | Fascicle Length (mm) | Minimum Continuation Quality +50% | 58 | 39 | 13.52 | 11.03 | 2.03 | 92 | 0.045 |
| Eulemur coronatus | Deep temporalis | Fascicle Length (mm) | Minimum Continuation Quality -50% | 58 | 62 | 13.52 | 14.56 | -0.65 | 115.3 | 0.516 |
| Eulemur coronatus | Deep temporalis | Fascicle Length (mm) | Direction Coefficient 0.3 | 58 | 57 | 13.52 | 14.59 | -0.63 | 104.1 | 0.532 |
| Eulemur coronatus | Deep temporalis | Fascicle Length (mm) | Direction Coefficient 0.1 | 58 | 50 | 13.52 | 10.69 | 2.31 | 97.1 | 0.023 |
| Eulemur coronatus | Deep temporalis | Fascicle Length (mm) | Minimum Length 100% Cylinder Length | 58 | 37 | 13.52 | 16.11 | -1.57 | 75.3 | 0.122 |
| Eulemur coronatus | Deep temporalis | Fascicle Length (mm) | Minimum Length 50% Cylinder Length | 58 | 74 | 13.52 | 11.62 | 1.43 | 119.5 | 0.155 |
| Eulemur coronatus | Lateral pterygoid | Fascicle Length (mm) | Minimum Seed Correlation +50% | 33 | 15 | 7.50 | 7.83 | -0.51 | 27.3 | 0.617 |
| Eulemur coronatus | Lateral pterygoid | Fascicle Length (mm) | Minimum Seed Correlation -50% | 33 | 36 | 7.50 | 7.33 | 0.33 | 66.4 | 0.745 |
| Eulemur coronatus | Lateral pterygoid | Fascicle Length (mm) | Minimum Continuation Quality +50% | 33 | 14 | 7.50 | 6.25 | 2.69 | 43.6 | 0.010 |
| Eulemur coronatus | Lateral pterygoid | Fascicle Length (mm) | Minimum Continuation Quality -50% | 33 | 57 | 7.50 | 9.27 | -3.21 | 85.6 | 0.002 |
| Eulemur coronatus | Lateral pterygoid | Fascicle Length (mm) | Direction Coefficient 0.2 | 33 | 36 | 7.50 | 6.39 | 2.46 | 59.2 | 0.017 |
| Eulemur coronatus | Lateral pterygoid | Fascicle Length (mm) | Direction Coefficient 0.1 | 33 | 20 | 7.50 | 6.32 | 2.33 | 48.8 | 0.024 |
| Eulemur coronatus | Lateral pterygoid | Fascicle Length (mm) | Minimum Length 100% Cylinder Length | 33 | 18 | 7.50 | 9.16 | -3.23 | 44.9 | 0.002 |
| Eulemur coronatus | Lateral pterygoid | Fascicle Length (mm) | Minimum Length 50% Cylinder Length | 33 | 51 | 7.50 | 6.32 | 2.37 | 74.5 | 0.020 |
| Eulemur coronatus | Medial pterygoid | Fascicle Length (mm) | Minimum Seed Correlation +50% | 46 | 9 | 9.08 | 10.61 | -1.17 | 9.5 | 0.272 |
| Eulemur coronatus | Medial pterygoid | Fascicle Length (mm) | Minimum Seed Correlation -50% | 46 | 46 | 9.08 | 9.08 | 0.00 | 90 | 1.000 |
| Eulemur coronatus | Medial pterygoid | Fascicle Length (mm) | Minimum Continuation Quality +50% | 46 | 8 | 9.08 | 7.35 | 3.47 | 28.9 | 0.002 |
| Eulemur coronatus | Medial pterygoid | Fascicle Length (mm) | Minimum Continuation Quality -50% | 46 | 116 | 9.08 | 11.41 | -4.22 | 139.3 | 0.000 |
| Eulemur coronatus | Medial pterygoid | Fascicle Length (mm) | Direction Coefficient 0.2 | 46 | 38 | 9.08 | 8.78 | 0.55 | 80.5 | 0.583 |
| Eulemur coronatus | Medial pterygoid | Fascicle Length (mm) | Direction Coefficient 0.1 | 46 | 16 | 9.08 | 7.73 | 2.43 | 40.5 | 0.020 |
| Eulemur coronatus | Medial pterygoid | Fascicle Length (mm) | Minimum Length 100% Cylinder Length | 46 | 24 | 9.08 | 10.73 | -2.67 | 48.6 | 0.010 |
| Eulemur coronatus | Medial pterygoid | Fascicle Length (mm) | Minimum Length 50% Cylinder Length | 46 | 85 | 9.08 | 7.21 | 3.91 | 99 | 0.000 |
| Eulemur coronatus | Posterior digastric | Fascicle Length (mm) | Minimum Seed Correlation +50% | 30 | 14 | 9.80 | 11.25 | -1.04 | 23.2 | 0.308 |
| Eulemur coronatus | Posterior digastric | Fascicle Length (mm) | Minimum Seed Correlation -50% | 30 | 35 | 9.80 | 9.47 | 0.34 | 60.8 | 0.738 |
| Eulemur coronatus | Posterior digastric | Fascicle Length (mm) | Minimum Continuation Quality +50% | 30 | 24 | 9.80 | 7.86 | 2.16 | 50.5 | 0.036 |
| Eulemur coronatus | Posterior digastric | Fascicle Length (mm) | Minimum Continuation Quality -50% | 30 | 38 | 9.80 | 10.47 | -0.61 | 66 | 0.545 |
| Eulemur coronatus | Posterior digastric | Fascicle Length (mm) | Direction Coefficient 0.2 | 30 | 33 | 9.80 | 8.89 | 0.91 | 60.4 | 0.365 |
| Eulemur coronatus | Posterior digastric | Fascicle Length (mm) | Direction Coefficient 0.1 | 30 | 26 | 9.80 | 7.92 | 2.05 | 52.5 | 0.046 |
| Eulemur coronatus | Posterior digastric | Fascicle Length (mm) | Minimum Length 100% Cylinder Length | 30 | 24 | 9.80 | 11.16 | -1.33 | 51.5 | 0.189 |
| Eulemur coronatus | Posterior digastric | Fascicle Length (mm) | Minimum Length 50% Cylinder Length | 30 | 43 | 9.80 | 8.29 | 1.58 | 63.5 | 0.119 |
| Eulemur coronatus | Superficial masseter | Fascicle Length (mm) | Minimum Seed Correlation +50% | 62 | 12 | 9.36 | 10.54 | -1.23 | 15.9 | 0.237 |
| Eulemur coronatus | Superficial masseter | Fascicle Length (mm) | Minimum Seed Correlation -50% | 62 | 66 | 9.36 | 9.21 | 0.28 | 125.3 | 0.779 |
| Eulemur coronatus | Superficial masseter | Fascicle Length (mm) | Minimum Continuation Quality +50% | 62 | 4 | 9.36 | 7.63 | 3.87 | 40.3 | 0.000 |
| Eulemur coronatus | Superficial masseter | Fascicle Length (mm) | Minimum Continuation Quality -50% | 62 | 107 | 9.36 | 12.34 | -4.59 | 166.9 | 0.000 |
| Eulemur coronatus | Superficial masseter | Fascicle Length (mm) | Direction Coefficient 0.2 | 62 | 55 | 9.36 | 8.28 | 2.38 | 95.2 | 0.020 |
| Eulemur coronatus | Superficial masseter | Fascicle Length (mm) | Direction Coefficient 0.1 | 62 | 12 | 9.36 | 8.53 | 1.29 | 27 | 0.208 |
| Eulemur coronatus | Superficial masseter | Fascicle Length (mm) | Minimum Length 100% Cylinder Length | 62 | 30 | 9.36 | 11.73 | -3.44 | 58.2 | 0.001 |
| Eulemur coronatus | Superficial masseter | Fascicle Length (mm) | Minimum Length 50% Cylinder Length | 62 | 96 | 9.36 | 7.90 | 2.85 | 131.9 | 0.005 |
| Eulemur coronatus | Superficial temporalis | Fascicle Length (mm) | Minimum Seed Correlation +50% | 30 | 4 | 11.83 | 14.00 | -0.68 | 3.4 | 0.542 |
| Eulemur coronatus | Superficial temporalis | Fascicle Length (mm) | Minimum Seed Correlation -50% | 30 | 36 | 11.83 | 11.53 | 0.29 | 60.9 | 0.773 |
| Eulemur coronatus | Superficial temporalis | Fascicle Length (mm) | Minimum Continuation Quality +50% | 30 | 9 | 11.83 | 8.95 | 3.60 | 33.4 | 0.001 |
| Eulemur coronatus | Superficial temporalis | Fascicle Length (mm) | Minimum Continuation Quality -50% | 30 | 54 | 11.83 | 15.82 | -3.28 | 81.3 | 0.002 |
| Eulemur coronatus | Superficial temporalis | Fascicle Length (mm) | Direction Coefficient 0.3 | 30 | 41 | 11.83 | 12.36 | -0.51 | 64.9 | 0.612 |
| Eulemur coronatus | Superficial temporalis | Fascicle Length (mm) | Direction Coefficient 0.1 | 30 | 15 | 11.83 | 9.99 | 2.14 | 40.3 | 0.039 |
| Eulemur coronatus | Superficial temporalis | Fascicle Length (mm) | Minimum Length 100% Cylinder Length | 30 | 13 | 11.83 | 14.31 | -2.09 | 29.2 | 0.045 |
| Eulemur coronatus | Superficial temporalis | Fascicle Length (mm) | Minimum Length 50% Cylinder Length | 30 | 51 | 11.83 | 9.57 | 2.37 | 59 | 0.021 |
| Eulemur coronatus | Zygomaticomandibularis | Fascicle Length (mm) | Minimum Seed Correlation +50% | 44 | 10 | 6.35 | 7.62 | -1.64 | 12.1 | 0.126 |
| Eulemur coronatus | Zygomaticomandibularis | Fascicle Length (mm) | Minimum Seed Correlation -50% | 44 | 18 | 6.35 | 5.11 | 3.59 | 60 | 0.001 |
| Eulemur coronatus | Zygomaticomandibularis | Fascicle Length (mm) | Minimum Continuation Quality +50% | 44 | 3 | 6.35 | 6.19 | 0.38 | 7.4 | 0.712 |
| Eulemur coronatus | Zygomaticomandibularis | Fascicle Length (mm) | Minimum Continuation Quality -50% | 44 | 45 | 6.35 | 7.37 | -2.07 | 80.3 | 0.041 |
| Eulemur coronatus | Zygomaticomandibularis | Fascicle Length (mm) | Direction Coefficient 0.2 | 44 | 11 | 6.35 | 4.94 | 3.72 | 40.4 | 0.001 |
| Eulemur coronatus | Zygomaticomandibularis | Fascicle Length (mm) | Direction Coefficient 0.1 | 44 | 3 | 6.35 | 5.07 | 2.74 | 5.3 | 0.039 |
| Eulemur coronatus | Zygomaticomandibularis | Fascicle Length (mm) | Minimum Length 100% Cylinder Length | 44 | 3 | 6.35 | 6.49 | -0.35 | 9.4 | 0.733 |
| Eulemur coronatus | Zygomaticomandibularis | Fascicle Length (mm) | Minimum Length 50% Cylinder Length | 44 | 3 | 6.35 | 6.49 | -0.35 | 9.4 | 0.733 |
| Eulemur coronatus | Zygomatic temporalis | Fascicle Length (mm) | Minimum Seed Correlation +50% | 41 | 15 | 9.40 | 11.29 | -1.99 | 25.4 | 0.057 |
| Eulemur coronatus | Zygomatic temporalis | Fascicle Length (mm) | Minimum Seed Correlation -50% | 41 | 34 | 9.40 | 9.35 | 0.07 | 69.1 | 0.944 |
| Eulemur coronatus | Zygomatic temporalis | Fascicle Length (mm) | Minimum Continuation Quality +50% | 41 | 24 | 9.40 | 6.91 | 3.75 | 61.6 | 0.000 |
| Eulemur coronatus | Zygomatic temporalis | Fascicle Length (mm) | Minimum Continuation Quality -50% | 41 | 48 | 9.40 | 11.01 | -1.87 | 82 | 0.065 |
| Eulemur coronatus | Zygomatic temporalis | Fascicle Length (mm) | Direction Coefficient 0.2 | 41 | 30 | 9.40 | 7.77 | 2.56 | 68.8 | 0.013 |
| Eulemur coronatus | Zygomatic temporalis | Fascicle Length (mm) | Direction Coefficient 0.1 | 41 | 18 | 9.40 | 6.65 | 4.82 | 55.9 | 0.000 |
| Eulemur coronatus | Zygomatic temporalis | Fascicle Length (mm) | Minimum Length 100% Cylinder Length | 41 | 22 | 9.40 | 11.16 | -2.28 | 48.8 | 0.027 |
| Eulemur coronatus | Zygomatic temporalis | Fascicle Length (mm) | Minimum Length 50% Cylinder Length | 41 | 51 | 9.40 | 7.74 | 2.34 | 89.2 | 0.022 |
| Otolemur crassicaudatus | Anterior digastric | Fascicle Length (mm) | Minimum Seed Correlation +50% | 34 | 3 | 7.65 | 14.70 | -4.65 | 2.9 | 0.021 |
| Otolemur crassicaudatus | Anterior digastric | Fascicle Length (mm) | Minimum Seed Correlation -50% | 34 | 52 | 7.65 | 7.08 | 0.72 | 69 | 0.475 |
| Otolemur crassicaudatus | Anterior digastric | Fascicle Length (mm) | Minimum Continuation Quality +50% | 34 | 23 | 7.65 | 6.32 | 1.44 | 50.8 | 0.155 |
| Otolemur crassicaudatus | Anterior digastric | Fascicle Length (mm) | Minimum Continuation Quality -50% | 34 | 40 | 7.65 | 9.19 | -1.72 | 71.9 | 0.091 |
| Otolemur crassicaudatus | Anterior digastric | Fascicle Length (mm) | Direction Coefficient 0.2 | 34 | 33 | 7.65 | 7.43 | 0.26 | 64.7 | 0.799 |
| Otolemur crassicaudatus | Anterior digastric | Fascicle Length (mm) | Direction Coefficient 0.1 | 34 | 32 | 7.65 | 6.11 | 2.02 | 58.6 | 0.048 |
| Otolemur crassicaudatus | Anterior digastric | Fascicle Length (mm) | Minimum Length 100% Cylinder Length | 34 | 27 | 7.65 | 8.51 | -0.94 | 56.7 | 0.349 |
| Otolemur crassicaudatus | Anterior digastric | Fascicle Length (mm) | Minimum Length 50% Cylinder Length | 34 | 36 | 7.65 | 7.74 | -0.10 | 68 | 0.920 |
| Otolemur crassicaudatus | Deep masseter | Fascicle Length (mm) | Minimum Seed Correlation +50% | 43 | 37 | 7.97 | 8.07 | -0.23 | 75.6 | 0.817 |
| Otolemur crassicaudatus | Deep masseter | Fascicle Length (mm) | Minimum Seed Correlation -50% | 43 | 43 | 7.97 | 7.97 | 0.00 | 84 | 1.000 |
| Otolemur crassicaudatus | Deep masseter | Fascicle Length (mm) | Minimum Continuation Quality -50% | 43 | 355 | 7.97 | 9.67 | -4.97 | 70.8 | 0.000 |
| Otolemur crassicaudatus | Deep masseter | Fascicle Length (mm) | Direction Coefficient 0.2 | 43 | 29 | 7.97 | 7.47 | 1.14 | 65.3 | 0.257 |
| Otolemur crassicaudatus | Deep masseter | Fascicle Length (mm) | Direction Coefficient 0.1 | 43 | 10 | 7.97 | 7.06 | 1.75 | 19.3 | 0.095 |
| Otolemur crassicaudatus | Deep masseter | Fascicle Length (mm) | Minimum Length 100% Cylinder Length | 43 | 19 | 7.97 | 9.69 | -3.80 | 45.2 | 0.000 |
| Otolemur crassicaudatus | Deep masseter | Fascicle Length (mm) | Minimum Length 50% Cylinder Length | 43 | 85 | 7.97 | 6.28 | 4.37 | 95.2 | 0.000 |
| Otolemur crassicaudatus | Deep temporalis | Fascicle Length (mm) | Minimum Seed Correlation +50% | 74 | 17 | 9.21 | 14.44 | -2.59 | 19.8 | 0.018 |
| Otolemur crassicaudatus | Deep temporalis | Fascicle Length (mm) | Minimum Seed Correlation -50% | 74 | 77 | 9.21 | 9.07 | 0.16 | 148.6 | 0.876 |
| Otolemur crassicaudatus | Deep temporalis | Fascicle Length (mm) | Minimum Continuation Quality +50% | 74 | 33 | 9.21 | 5.90 | 4.65 | 96.7 | 0.000 |
| Otolemur crassicaudatus | Deep temporalis | Fascicle Length (mm) | Minimum Continuation Quality -50% | 74 | 82 | 9.21 | 11.42 | -1.93 | 140.7 | 0.056 |
| Otolemur crassicaudatus | Deep temporalis | Fascicle Length (mm) | Direction Coefficient 0.3 | 74 | 78 | 9.21 | 10.07 | -0.82 | 143.3 | 0.417 |
| Otolemur crassicaudatus | Deep temporalis | Fascicle Length (mm) | Direction Coefficient 0.1 | 74 | 65 | 9.21 | 7.09 | 2.91 | 106.7 | 0.004 |
| Otolemur crassicaudatus | Deep temporalis | Fascicle Length (mm) | Minimum Length 100% Cylinder Length | 74 | 50 | 9.21 | 13.00 | -2.97 | 82.3 | 0.004 |
| Otolemur crassicaudatus | Deep temporalis | Fascicle Length (mm) | Minimum Length 50% Cylinder Length | 74 | 101 | 9.21 | 8.60 | 0.64 | 171.6 | 0.525 |
| Otolemur crassicaudatus | Lateral pterygoid | Fascicle Length (mm) | Minimum Seed Correlation +50% | 40 | 4 | 7.10 | 8.70 | -1.22 | 3.7 | 0.296 |
| Otolemur crassicaudatus | Lateral pterygoid | Fascicle Length (mm) | Minimum Seed Correlation -50% | 40 | 52 | 7.10 | 6.90 | 0.37 | 81.4 | 0.710 |
| Otolemur crassicaudatus | Lateral pterygoid | Fascicle Length (mm) | Minimum Continuation Quality +50% | 40 | 18 | 7.10 | 6.28 | 1.35 | 43.5 | 0.184 |
| Otolemur crassicaudatus | Lateral pterygoid | Fascicle Length (mm) | Minimum Continuation Quality -50% | 40 | 61 | 7.10 | 8.68 | -2.78 | 92.8 | 0.007 |
| Otolemur crassicaudatus | Lateral pterygoid | Fascicle Length (mm) | Direction Coefficient 0.2 | 40 | 32 | 7.10 | 6.60 | 0.96 | 69.3 | 0.341 |
| Otolemur crassicaudatus | Lateral pterygoid | Fascicle Length (mm) | Direction Coefficient 0.1 | 40 | 13 | 7.10 | 5.77 | 2.40 | 40.3 | 0.021 |
| Otolemur crassicaudatus | Lateral pterygoid | Fascicle Length (mm) | Minimum Length 100% Cylinder Length | 40 | 22 | 7.10 | 8.74 | -2.54 | 47.3 | 0.015 |
| Otolemur crassicaudatus | Lateral pterygoid | Fascicle Length (mm) | Minimum Length 50% Cylinder Length | 40 | 53 | 7.10 | 6.24 | 1.55 | 85.9 | 0.124 |
| Otolemur crassicaudatus | Medial pterygoid | Fascicle Length (mm) | Minimum Seed Correlation -50% | 45 | 51 | 7.62 | 7.39 | 0.45 | 91.8 | 0.655 |
| Otolemur crassicaudatus | Medial pterygoid | Fascicle Length (mm) | Minimum Continuation Quality +50% | 45 | 3 | 7.62 | 5.53 | 4.02 | 8.3 | 0.004 |
| Otolemur crassicaudatus | Medial pterygoid | Fascicle Length (mm) | Minimum Continuation Quality -50% | 45 | 75 | 7.62 | 11.30 | -5.08 | 112.7 | 0.000 |
| Otolemur crassicaudatus | Medial pterygoid | Fascicle Length (mm) | Direction Coefficient 0.2 | 45 | 27 | 7.62 | 7.43 | 0.32 | 56.4 | 0.747 |
| Otolemur crassicaudatus | Medial pterygoid | Fascicle Length (mm) | Direction Coefficient 0.1 | 45 | 17 | 7.62 | 6.24 | 2.86 | 55.5 | 0.006 |
| Otolemur crassicaudatus | Medial pterygoid | Fascicle Length (mm) | Minimum Length 100% Cylinder Length | 45 | 20 | 7.62 | 9.72 | -3.33 | 40.6 | 0.002 |
| Otolemur crassicaudatus | Medial pterygoid | Fascicle Length (mm) | Minimum Length 50% Cylinder Length | 45 | 67 | 7.62 | 6.31 | 2.63 | 98.4 | 0.010 |
| Otolemur crassicaudatus | Posterior digastric | Fascicle Length (mm) | Minimum Seed Correlation +50% | 37 | 6 | 8.90 | 13.77 | -2.28 | 6.8 | 0.058 |
| Otolemur crassicaudatus | Posterior digastric | Fascicle Length (mm) | Minimum Seed Correlation -50% | 37 | 39 | 8.90 | 8.66 | 0.22 | 73.8 | 0.829 |
| Otolemur crassicaudatus | Posterior digastric | Fascicle Length (mm) | Minimum Continuation Quality +50% | 37 | 21 | 8.90 | 4.73 | 4.86 | 44.1 | 0.000 |
| Otolemur crassicaudatus | Posterior digastric | Fascicle Length (mm) | Minimum Continuation Quality -50% | 37 | 42 | 8.90 | 10.47 | -1.15 | 73.1 | 0.254 |
| Otolemur crassicaudatus | Posterior digastric | Fascicle Length (mm) | Direction Coefficient 0.2 | 37 | 41 | 8.90 | 7.98 | 0.95 | 63.2 | 0.345 |
| Otolemur crassicaudatus | Posterior digastric | Fascicle Length (mm) | Direction Coefficient 0.1 | 37 | 40 | 8.90 | 5.92 | 3.21 | 56.8 | 0.002 |
| Otolemur crassicaudatus | Posterior digastric | Fascicle Length (mm) | Minimum Length 100% Cylinder Length | 37 | 30 | 8.90 | 10.11 | -1.02 | 63.1 | 0.312 |
| Otolemur crassicaudatus | Posterior digastric | Fascicle Length (mm) | Minimum Length 50% Cylinder Length | 37 | 49 | 8.90 | 7.60 | 1.21 | 77.6 | 0.229 |
| Otolemur crassicaudatus | Superficial masseter | Fascicle Length (mm) | Minimum Seed Correlation +50% | 56 | 3 | 9.33 | 10.90 | -1.37 | 2.5 | 0.282 |
| Otolemur crassicaudatus | Superficial masseter | Fascicle Length (mm) | Minimum Seed Correlation -50% | 56 | 58 | 9.33 | 9.24 | 0.18 | 111.8 | 0.859 |
| Otolemur crassicaudatus | Superficial masseter | Fascicle Length (mm) | Minimum Continuation Quality +50% | 56 | 7 | 9.33 | 7.55 | 2.13 | 9.2 | 0.061 |
| Otolemur crassicaudatus | Superficial masseter | Fascicle Length (mm) | Minimum Continuation Quality -50% | 56 | 126 | 9.33 | 12.45 | -5.62 | 163.9 | 0.000 |
| Otolemur crassicaudatus | Superficial masseter | Fascicle Length (mm) | Direction Coefficient 0.3 | 56 | 74 | 9.33 | 9.40 | -0.13 | 117 | 0.901 |
| Otolemur crassicaudatus | Superficial masseter | Fascicle Length (mm) | Direction Coefficient 0.1 | 56 | 39 | 9.33 | 8.82 | 0.97 | 89.5 | 0.335 |
| Otolemur crassicaudatus | Superficial masseter | Fascicle Length (mm) | Minimum Length 100% Cylinder Length | 56 | 26 | 9.33 | 11.86 | -4.53 | 63 | 0.000 |
| Otolemur crassicaudatus | Superficial masseter | Fascicle Length (mm) | Minimum Length 50% Cylinder Length | 56 | 98 | 9.33 | 7.49 | 3.84 | 121.9 | 0.000 |
| Otolemur crassicaudatus | Zygomatic temporalis | Fascicle Length (mm) | Minimum Seed Correlation +50% | 43 | 3 | 7.06 | 9.86 | -2.37 | 2.7 | 0.109 |
| Otolemur crassicaudatus | Zygomatic temporalis | Fascicle Length (mm) | Minimum Seed Correlation -50% | 43 | 59 | 7.06 | 6.47 | 1.07 | 87.3 | 0.290 |
| Otolemur crassicaudatus | Zygomatic temporalis | Fascicle Length (mm) | Minimum Continuation Quality +50% | 43 | 22 | 7.06 | 5.17 | 3.43 | 62.3 | 0.001 |
| Otolemur crassicaudatus | Zygomatic temporalis | Fascicle Length (mm) | Minimum Continuation Quality -50% | 43 | 38 | 7.06 | 11.34 | -3.89 | 50.2 | 0.000 |
| Otolemur crassicaudatus | Zygomatic temporalis | Fascicle Length (mm) | Direction Coefficient 0.2 | 43 | 37 | 7.06 | 6.45 | 1.06 | 77.8 | 0.293 |
| Otolemur crassicaudatus | Zygomatic temporalis | Fascicle Length (mm) | Direction Coefficient 0.1 | 43 | 28 | 7.06 | 5.83 | 2.27 | 68.8 | 0.027 |
| Otolemur crassicaudatus | Zygomatic temporalis | Fascicle Length (mm) | Minimum Length 100% Cylinder Length | 43 | 35 | 7.06 | 7.99 | -1.52 | 75 | 0.132 |
| Otolemur crassicaudatus | Zygomatic temporalis | Fascicle Length (mm) | Minimum Length 50% Cylinder Length | 43 | 46 | 7.06 | 6.44 | 1.05 | 86 | 0.295 |
| Lontra canadensis | Digastric | Fascicle Length (mm) | Minimum Seed Correlation +50% | 56 | 3 | 24.04 | 27.91 | -2.73 | 5.3 | 0.039 |
| Lontra canadensis | Digastric | Fascicle Length (mm) | Minimum Seed Correlation -50% | 56 | 81 | 24.04 | 21.95 | 1.76 | 123.6 | 0.082 |
| Lontra canadensis | Digastric | Fascicle Length (mm) | Minimum Continuation Quality +50% | 56 | 46 | 24.04 | 16.84 | 6.45 | 97.4 | 0.000 |
| Lontra canadensis | Digastric | Fascicle Length (mm) | Minimum Continuation Quality -50% | 56 | 61 | 24.04 | 29.84 | -3.94 | 109.1 | 0.000 |
| Lontra canadensis | Digastric | Fascicle Length (mm) | Direction Coefficient 0.2 | 56 | 56 | 24.04 | 22.72 | 1.09 | 109.5 | 0.279 |
| Lontra canadensis | Digastric | Fascicle Length (mm) | Direction Coefficient 0.1 | 56 | 55 | 24.04 | 19.24 | 4.09 | 107.1 | 0.000 |
| Lontra canadensis | Digastric | Fascicle Length (mm) | Minimum Length 100% Cylinder Length | 56 | 45 | 24.04 | 26.24 | -1.96 | 97.3 | 0.053 |
| Lontra canadensis | Digastric | Fascicle Length (mm) | Minimum Length 50% Cylinder Length | 56 | 59 | 24.04 | 23.63 | 0.32 | 113 | 0.750 |
| Lontra canadensis | Deep masseter | Fascicle Length (mm) | Minimum Seed Correlation +50% | 60 | 8 | 9.06 | 11.12 | -2.43 | 11.5 | 0.032 |
| Lontra canadensis | Deep masseter | Fascicle Length (mm) | Minimum Seed Correlation -50% | 60 | 62 | 9.06 | 8.99 | 0.12 | 119.8 | 0.902 |
| Lontra canadensis | Deep masseter | Fascicle Length (mm) | Minimum Continuation Quality +50% | 60 | 13 | 9.06 | 7.08 | 3.23 | 32.7 | 0.003 |
| Lontra canadensis | Deep masseter | Fascicle Length (mm) | Minimum Continuation Quality -50% | 60 | 91 | 9.06 | 11.47 | -3.29 | 143.3 | 0.001 |
| Lontra canadensis | Deep masseter | Fascicle Length (mm) | Direction Coefficient 0.2 | 60 | 52 | 9.06 | 8.75 | 0.64 | 101.8 | 0.521 |
| Lontra canadensis | Deep masseter | Fascicle Length (mm) | Direction Coefficient 0.1 | 60 | 32 | 9.06 | 7.72 | 2.51 | 86.5 | 0.014 |
| Lontra canadensis | Deep masseter | Fascicle Length (mm) | Minimum Length 100% Cylinder Length | 60 | 30 | 9.06 | 11.55 | -3.99 | 67.4 | 0.000 |
| Lontra canadensis | Deep masseter | Fascicle Length (mm) | Minimum Length 50% Cylinder Length | 60 | 97 | 9.06 | 7.32 | 3.36 | 129.9 | 0.001 |
| Lontra canadensis | Deep temporalis | Fascicle Length (mm) | Minimum Seed Correlation +50% | 66 | 13 | 17.75 | 20.54 | -1.50 | 16.1 | 0.152 |
| Lontra canadensis | Deep temporalis | Fascicle Length (mm) | Minimum Seed Correlation -50% | 66 | 70 | 17.75 | 17.59 | 0.17 | 133.2 | 0.865 |
| Lontra canadensis | Deep temporalis | Fascicle Length (mm) | Minimum Continuation Quality +50% | 66 | 29 | 17.75 | 15.20 | 2.72 | 83.3 | 0.008 |
| Lontra canadensis | Deep temporalis | Fascicle Length (mm) | Minimum Continuation Quality -50% | 66 | 79 | 17.75 | 23.17 | -3.69 | 117.3 | 0.000 |
| Lontra canadensis | Deep temporalis | Fascicle Length (mm) | Direction Coefficient 0.3 | 66 | 79 | 17.75 | 18.23 | -0.46 | 142.6 | 0.649 |
| Lontra canadensis | Deep temporalis | Fascicle Length (mm) | Direction Coefficient 0.1 | 66 | 57 | 17.75 | 15.49 | 2.54 | 119 | 0.013 |
| Lontra canadensis | Deep temporalis | Fascicle Length (mm) | Minimum Length 100% Cylinder Length | 66 | 39 | 17.75 | 21.66 | -3.92 | 93.5 | 0.000 |
| Lontra canadensis | Deep temporalis | Fascicle Length (mm) | Minimum Length 50% Cylinder Length | 66 | 96 | 17.75 | 15.48 | 2.46 | 145.5 | 0.015 |
| Lontra canadensis | Lateral pterygoid | Fascicle Length (mm) | Minimum Seed Correlation +50% | 36 | 3 | 5.45 | 7.11 | -1.42 | 2.1 | 0.287 |
| Lontra canadensis | Lateral pterygoid | Fascicle Length (mm) | Minimum Seed Correlation -50% | 36 | 51 | 5.45 | 5.34 | 0.45 | 73.1 | 0.651 |
| Lontra canadensis | Lateral pterygoid | Fascicle Length (mm) | Minimum Continuation Quality +50% | 36 | 6 | 5.45 | 4.71 | 3.58 | 39.3 | 0.001 |
| Lontra canadensis | Lateral pterygoid | Fascicle Length (mm) | Minimum Continuation Quality -50% | 36 | 81 | 5.45 | 6.50 | -4.02 | 97.6 | 0.000 |
| Lontra canadensis | Lateral pterygoid | Fascicle Length (mm) | Direction Coefficient 0.2 | 36 | 22 | 5.45 | 4.90 | 2.57 | 51.2 | 0.013 |
| Lontra canadensis | Lateral pterygoid | Fascicle Length (mm) | Direction Coefficient 0.1 | 36 | 10 | 5.45 | 4.59 | 3.88 | 42.2 | 0.000 |
| Lontra canadensis | Lateral pterygoid | Fascicle Length (mm) | Minimum Length 100% Cylinder Length | 36 | 9 | 5.45 | 7.21 | -5.63 | 17.9 | 0.000 |
| Lontra canadensis | Lateral pterygoid | Fascicle Length (mm) | Minimum Length 50% Cylinder Length | 36 | 81 | 5.45 | 4.32 | 4.87 | 74.8 | 0.000 |
| Lontra canadensis | Medial pterygoid | Fascicle Length (mm) | Minimum Seed Correlation +50% | 44 | 8 | 11.56 | 16.38 | -1.93 | 8 | 0.090 |
| Lontra canadensis | Medial pterygoid | Fascicle Length (mm) | Minimum Seed Correlation -50% | 44 | 45 | 11.56 | 11.49 | 0.08 | 86.9 | 0.938 |
| Lontra canadensis | Medial pterygoid | Fascicle Length (mm) | Minimum Continuation Quality +50% | 44 | 9 | 11.56 | 10.00 | 1.64 | 24.1 | 0.114 |
| Lontra canadensis | Medial pterygoid | Fascicle Length (mm) | Minimum Continuation Quality -50% | 44 | 77 | 11.56 | 14.62 | -3.20 | 114.9 | 0.002 |
| Lontra canadensis | Medial pterygoid | Fascicle Length (mm) | Direction Coefficient 0.3 | 44 | 55 | 11.56 | 13.54 | -2.05 | 97 | 0.043 |
| Lontra canadensis | Medial pterygoid | Fascicle Length (mm) | Direction Coefficient 0.1 | 44 | 24 | 11.56 | 9.54 | 2.70 | 63.8 | 0.009 |
| Lontra canadensis | Medial pterygoid | Fascicle Length (mm) | Minimum Length 100% Cylinder Length | 44 | 18 | 11.56 | 14.90 | -2.51 | 28 | 0.018 |
| Lontra canadensis | Medial pterygoid | Fascicle Length (mm) | Minimum Length 50% Cylinder Length | 44 | 76 | 11.56 | 9.54 | 2.53 | 87.8 | 0.013 |
| Lontra canadensis | Superficial masseter | Fascicle Length (mm) | Minimum Seed Correlation +50% | 64 | 7 | 12.01 | 15.59 | -2.16 | 7.5 | 0.065 |
| Lontra canadensis | Superficial masseter | Fascicle Length (mm) | Minimum Seed Correlation -50% | 64 | 69 | 12.01 | 11.80 | 0.28 | 129.9 | 0.783 |
| Lontra canadensis | Superficial masseter | Fascicle Length (mm) | Minimum Continuation Quality +50% | 64 | 29 | 12.01 | 9.64 | 3.52 | 90.6 | 0.001 |
| Lontra canadensis | Superficial masseter | Fascicle Length (mm) | Minimum Continuation Quality -50% | 64 | 97 | 12.01 | 13.81 | -1.92 | 156.6 | 0.057 |
| Lontra canadensis | Superficial masseter | Fascicle Length (mm) | Direction Coefficient 0.2 | 64 | 54 | 12.01 | 10.87 | 1.64 | 113.1 | 0.103 |
| Lontra canadensis | Superficial masseter | Fascicle Length (mm) | Direction Coefficient 0.1 | 64 | 39 | 12.01 | 9.85 | 3.26 | 99.4 | 0.002 |
| Lontra canadensis | Superficial masseter | Fascicle Length (mm) | Minimum Length 100% Cylinder Length | 64 | 33 | 12.01 | 14.92 | -3.24 | 68.8 | 0.002 |
| Lontra canadensis | Superficial masseter | Fascicle Length (mm) | Minimum Length 50% Cylinder Length | 64 | 105 | 12.01 | 9.39 | 3.76 | 133.5 | 0.000 |
| Lontra canadensis | Superficial temporalis | Fascicle Length (mm) | Minimum Seed Correlation +50% | 72 | 33 | 17.64 | 20.35 | -1.54 | 52.5 | 0.129 |
| Lontra canadensis | Superficial temporalis | Fascicle Length (mm) | Minimum Seed Correlation -50% | 72 | 72 | 17.64 | 17.64 | 0.00 | 142 | 1.000 |
| Lontra canadensis | Superficial temporalis | Fascicle Length (mm) | Minimum Continuation Quality +50% | 72 | 19 | 17.64 | 13.39 | 4.24 | 86 | 0.000 |
| Lontra canadensis | Superficial temporalis | Fascicle Length (mm) | Minimum Continuation Quality -50% | 72 | 102 | 17.64 | 19.56 | -1.44 | 172 | 0.152 |
| Lontra canadensis | Superficial temporalis | Fascicle Length (mm) | Direction Coefficient 0.2 | 72 | 69 | 17.64 | 16.76 | 0.82 | 130.3 | 0.414 |
| Lontra canadensis | Superficial temporalis | Fascicle Length (mm) | Direction Coefficient 0.1 | 72 | 30 | 17.64 | 15.13 | 2.24 | 92.7 | 0.027 |
| Lontra canadensis | Superficial temporalis | Fascicle Length (mm) | Minimum Length 100% Cylinder Length | 72 | 36 | 17.64 | 22.89 | -3.63 | 72 | 0.001 |
| Lontra canadensis | Superficial temporalis | Fascicle Length (mm) | Minimum Length 50% Cylinder Length | 72 | 119 | 17.64 | 14.48 | 2.97 | 144.6 | 0.004 |
| Lontra canadensis | Zygomaticomandibularis | Fascicle Length (mm) | Minimum Seed Correlation +50% | 52 | 18 | 10.12 | 10.51 | -0.46 | 27.7 | 0.650 |
| Lontra canadensis | Zygomaticomandibularis | Fascicle Length (mm) | Minimum Seed Correlation -50% | 52 | 54 | 10.12 | 9.96 | 0.28 | 104 | 0.783 |
| Lontra canadensis | Zygomaticomandibularis | Fascicle Length (mm) | Minimum Continuation Quality +50% | 52 | 25 | 10.12 | 7.89 | 4.43 | 74.8 | 0.000 |
| Lontra canadensis | Zygomaticomandibularis | Fascicle Length (mm) | Minimum Continuation Quality -50% | 52 | 68 | 10.12 | 12.02 | -2.64 | 112.1 | 0.009 |
| Lontra canadensis | Zygomaticomandibularis | Fascicle Length (mm) | Direction Coefficient 0.2 | 52 | 47 | 10.12 | 8.83 | 2.43 | 95.7 | 0.017 |
| Lontra canadensis | Zygomaticomandibularis | Fascicle Length (mm) | Direction Coefficient 0.1 | 52 | 33 | 10.12 | 7.82 | 4.50 | 82.9 | 0.000 |
| Lontra canadensis | Zygomaticomandibularis | Fascicle Length (mm) | Minimum Length 100% Cylinder Length | 52 | 30 | 10.12 | 11.68 | -2.55 | 68.5 | 0.013 |
| Lontra canadensis | Zygomaticomandibularis | Fascicle Length (mm) | Minimum Length 50% Cylinder Length | 52 | 74 | 10.12 | 8.61 | 2.65 | 119.4 | 0.009 |
| Mustela nivalis | Digastric | Fascicle Length (mm) | Minimum Seed Correlation +50% | 47 | 19 | 8.74 | 12.40 | -3.48 | 39.3 | 0.001 |
| Mustela nivalis | Digastric | Fascicle Length (mm) | Minimum Seed Correlation -50% | 47 | 58 | 8.74 | 7.93 | 0.93 | 100.1 | 0.352 |
| Mustela nivalis | Digastric | Fascicle Length (mm) | Minimum Continuation Quality +50% | 47 | 46 | 8.74 | 7.77 | 1.14 | 90.1 | 0.257 |
| Mustela nivalis | Digastric | Fascicle Length (mm) | Minimum Continuation Quality -50% | 47 | 57 | 8.74 | 8.95 | -0.23 | 101.5 | 0.815 |
| Mustela nivalis | Digastric | Fascicle Length (mm) | Direction Coefficient 0.2 | 47 | 52 | 8.74 | 8.22 | 0.61 | 94.6 | 0.544 |
| Mustela nivalis | Digastric | Fascicle Length (mm) | Direction Coefficient 0.1 | 47 | 46 | 8.74 | 7.82 | 1.05 | 91 | 0.298 |
| Mustela nivalis | Digastric | Fascicle Length (mm) | Minimum Length 100% Cylinder Length | 47 | 46 | 8.74 | 9.16 | -0.48 | 90.9 | 0.636 |
| Mustela nivalis | Digastric | Fascicle Length (mm) | Minimum Length 50% Cylinder Length | 47 | 50 | 8.74 | 8.32 | 0.46 | 95 | 0.645 |
| Mustela nivalis | Deep masseter | Fascicle Length (mm) | Minimum Seed Correlation +50% | 30 | 3 | 3.40 | 3.04 | 2.60 | 23.7 | 0.016 |
| Mustela nivalis | Deep masseter | Fascicle Length (mm) | Minimum Seed Correlation -50% | 30 | 34 | 3.40 | 3.40 | -0.03 | 61 | 0.980 |
| Mustela nivalis | Deep masseter | Fascicle Length (mm) | Minimum Continuation Quality +50% | 30 | 2 | 3.40 | 3.05 | 0.87 | 1.2 | 0.522 |
| Mustela nivalis | Deep masseter | Fascicle Length (mm) | Minimum Continuation Quality -50% | 30 | 85 | 3.40 | 5.25 | -7.91 | 112.7 | 0.000 |
| Mustela nivalis | Deep masseter | Fascicle Length (mm) | Direction Coefficient 0.2 | 30 | 24 | 3.40 | 3.38 | 0.10 | 41.9 | 0.924 |
| Mustela nivalis | Deep masseter | Fascicle Length (mm) | Direction Coefficient 0.1 | 30 | 13 | 3.40 | 2.88 | 3.50 | 41 | 0.001 |
| Mustela nivalis | Deep masseter | Fascicle Length (mm) | Minimum Length 100% Cylinder Length | 30 | 9 | 3.40 | 4.31 | -4.21 | 16.5 | 0.001 |
| Mustela nivalis | Deep masseter | Fascicle Length (mm) | Minimum Length 50% Cylinder Length | 30 | 58 | 3.40 | 2.86 | 3.24 | 70.4 | 0.002 |
| Mustela nivalis | Deep temporalis | Fascicle Length (mm) | Minimum Seed Correlation +50% | 62 | 3 | 6.74 | 6.87 | -0.25 | 8.2 | 0.808 |
| Mustela nivalis | Deep temporalis | Fascicle Length (mm) | Minimum Seed Correlation -50% | 62 | 68 | 6.74 | 6.56 | 0.35 | 126.3 | 0.728 |
| Mustela nivalis | Deep temporalis | Fascicle Length (mm) | Minimum Continuation Quality +50% | 62 | 4 | 6.74 | 4.46 | 4.34 | 11.7 | 0.001 |
| Mustela nivalis | Deep temporalis | Fascicle Length (mm) | Minimum Continuation Quality -50% | 62 | 107 | 6.74 | 9.62 | -4.70 | 167 | 0.000 |
| Mustela nivalis | Deep temporalis | Fascicle Length (mm) | Direction Coefficient 0.2 | 62 | 60 | 6.74 | 6.47 | 0.55 | 118.7 | 0.581 |
| Mustela nivalis | Deep temporalis | Fascicle Length (mm) | Direction Coefficient 0.1 | 62 | 34 | 6.74 | 5.47 | 2.72 | 94 | 0.008 |
| Mustela nivalis | Deep temporalis | Fascicle Length (mm) | Minimum Length 100% Cylinder Length | 62 | 33 | 6.74 | 8.82 | -3.55 | 73.4 | 0.001 |
| Mustela nivalis | Deep temporalis | Fascicle Length (mm) | Minimum Length 50% Cylinder Length | 62 | 78 | 6.74 | 5.96 | 1.55 | 132.4 | 0.125 |
| Mustela nivalis | Lateral pterygoid | Fascicle Length (mm) | Minimum Seed Correlation +50% | 41 | 20 | 2.14 | 2.30 | -1.14 | 36.4 | 0.263 |
| Mustela nivalis | Lateral pterygoid | Fascicle Length (mm) | Minimum Seed Correlation -50% | 41 | 43 | 2.14 | 2.12 | 0.17 | 81.7 | 0.867 |
| Mustela nivalis | Lateral pterygoid | Fascicle Length (mm) | Minimum Continuation Quality +50% | 41 | 8 | 2.14 | 1.70 | 3.65 | 21.3 | 0.001 |
| Mustela nivalis | Lateral pterygoid | Fascicle Length (mm) | Minimum Continuation Quality -50% | 41 | 69 | 2.14 | 2.45 | -2.91 | 92.9 | 0.005 |
| Mustela nivalis | Lateral pterygoid | Fascicle Length (mm) | Direction Coefficient 0.2 | 41 | 36 | 2.14 | 2.00 | 1.24 | 74.9 | 0.220 |
| Mustela nivalis | Lateral pterygoid | Fascicle Length (mm) | Direction Coefficient 0.1 | 41 | 19 | 2.14 | 1.96 | 1.53 | 47.2 | 0.133 |
| Mustela nivalis | Lateral pterygoid | Fascicle Length (mm) | Minimum Length 100% Cylinder Length | 41 | 20 | 2.14 | 2.47 | -2.70 | 46.6 | 0.010 |
| Mustela nivalis | Lateral pterygoid | Fascicle Length (mm) | Minimum Length 50% Cylinder Length | 41 | 54 | 2.14 | 1.87 | 2.28 | 92 | 0.025 |
| Mustela nivalis | Medial pterygoid | Fascicle Length (mm) | Minimum Seed Correlation +50% | 45 | 25 | 3.56 | 3.57 | -0.03 | 47 | 0.973 |
| Mustela nivalis | Medial pterygoid | Fascicle Length (mm) | Minimum Seed Correlation -50% | 45 | 48 | 3.56 | 3.52 | 0.19 | 90.4 | 0.853 |
| Mustela nivalis | Medial pterygoid | Fascicle Length (mm) | Minimum Continuation Quality +50% | 45 | 4 | 3.56 | 2.50 | 4.55 | 15.8 | 0.000 |
| Mustela nivalis | Medial pterygoid | Fascicle Length (mm) | Minimum Continuation Quality -50% | 45 | 113 | 3.56 | 4.44 | -3.64 | 115.6 | 0.000 |
| Mustela nivalis | Medial pterygoid | Fascicle Length (mm) | Direction Coefficient 0.2 | 45 | 29 | 3.56 | 3.62 | -0.22 | 64.1 | 0.828 |
| Mustela nivalis | Medial pterygoid | Fascicle Length (mm) | Direction Coefficient 0.1 | 45 | 16 | 3.56 | 3.05 | 1.77 | 35 | 0.085 |
| Mustela nivalis | Medial pterygoid | Fascicle Length (mm) | Minimum Length 100% Cylinder Length | 45 | 24 | 3.56 | 4.32 | -2.76 | 54.2 | 0.008 |
| Mustela nivalis | Medial pterygoid | Fascicle Length (mm) | Minimum Length 50% Cylinder Length | 45 | 59 | 3.56 | 3.06 | 2.09 | 96.2 | 0.039 |
| Mustela nivalis | Superficial masseter | Fascicle Length (mm) | Minimum Seed Correlation -50% | 35 | 85 | 4.16 | 3.57 | 2.39 | 55.7 | 0.020 |
| Mustela nivalis | Superficial masseter | Fascicle Length (mm) | Minimum Continuation Quality +50% | 35 | 15 | 4.16 | 2.85 | 4.74 | 46.1 | 0.000 |
| Mustela nivalis | Superficial masseter | Fascicle Length (mm) | Minimum Continuation Quality -50% | 35 | 56 | 4.16 | 5.80 | -4.95 | 88.2 | 0.000 |
| Mustela nivalis | Superficial masseter | Fascicle Length (mm) | Direction Coefficient 0.2 | 35 | 37 | 4.16 | 3.52 | 2.41 | 61.6 | 0.019 |
| Mustela nivalis | Superficial masseter | Fascicle Length (mm) | Direction Coefficient 0.1 | 35 | 30 | 4.16 | 3.07 | 4.42 | 51.9 | 0.000 |
| Mustela nivalis | Superficial masseter | Fascicle Length (mm) | Minimum Length 100% Cylinder Length | 35 | 26 | 4.16 | 4.50 | -1.30 | 55.7 | 0.199 |
| Mustela nivalis | Superficial masseter | Fascicle Length (mm) | Minimum Length 50% Cylinder Length | 35 | 49 | 4.16 | 3.56 | 2.02 | 77.4 | 0.047 |
| Mustela nivalis | Superficial temporalis | Fascicle Length (mm) | Minimum Seed Correlation +50% | 61 | 35 | 5.92 | 6.94 | -2.39 | 72.1 | 0.019 |
| Mustela nivalis | Superficial temporalis | Fascicle Length (mm) | Minimum Seed Correlation -50% | 61 | 62 | 5.92 | 5.89 | 0.09 | 121 | 0.926 |
| Mustela nivalis | Superficial temporalis | Fascicle Length (mm) | Minimum Continuation Quality +50% | 61 | 21 | 5.92 | 5.32 | 1.55 | 54.9 | 0.127 |
| Mustela nivalis | Superficial temporalis | Fascicle Length (mm) | Minimum Continuation Quality -50% | 61 | 109 | 5.92 | 6.46 | -1.53 | 147.2 | 0.128 |
| Mustela nivalis | Superficial temporalis | Fascicle Length (mm) | Direction Coefficient 0.3 | 61 | 74 | 5.92 | 5.92 | 0.00 | 126.7 | 0.999 |
| Mustela nivalis | Superficial temporalis | Fascicle Length (mm) | Direction Coefficient 0.1 | 61 | 50 | 5.92 | 5.51 | 1.14 | 108.8 | 0.256 |
| Mustela nivalis | Superficial temporalis | Fascicle Length (mm) | Minimum Length 100% Cylinder Length | 61 | 28 | 5.92 | 7.65 | -4.38 | 66.4 | 0.000 |
| Mustela nivalis | Superficial temporalis | Fascicle Length (mm) | Minimum Length 50% Cylinder Length | 61 | 94 | 5.92 | 4.86 | 3.08 | 134.7 | 0.003 |
| Mustela nivalis | Zygomaticomandibularis | Fascicle Length (mm) | Minimum Seed Correlation +50% | 46 | 11 | 3.77 | 4.29 | -1.48 | 14.5 | 0.160 |
| Mustela nivalis | Zygomaticomandibularis | Fascicle Length (mm) | Minimum Seed Correlation -50% | 46 | 53 | 3.77 | 3.66 | 0.53 | 94.5 | 0.595 |
| Mustela nivalis | Zygomaticomandibularis | Fascicle Length (mm) | Minimum Continuation Quality +50% | 46 | 5 | 3.77 | 2.93 | 2.71 | 6.7 | 0.032 |
| Mustela nivalis | Zygomaticomandibularis | Fascicle Length (mm) | Minimum Continuation Quality -50% | 46 | 80 | 3.77 | 5.13 | -7.10 | 102.4 | 0.000 |
| Mustela nivalis | Zygomaticomandibularis | Fascicle Length (mm) | Direction Coefficient 0.2 | 46 | 28 | 3.77 | 3.37 | 2.07 | 71.3 | 0.042 |
| Mustela nivalis | Zygomaticomandibularis | Fascicle Length (mm) | Direction Coefficient 0.1 | 46 | 12 | 3.77 | 2.75 | 6.03 | 55.2 | 0.000 |
| Mustela nivalis | Zygomaticomandibularis | Fascicle Length (mm) | Minimum Length 100% Cylinder Length | 46 | 24 | 3.77 | 4.52 | -3.66 | 61.3 | 0.001 |
| Mustela nivalis | Zygomaticomandibularis | Fascicle Length (mm) | Minimum Length 50% Cylinder Length | 46 | 63 | 3.77 | 3.30 | 2.27 | 103.2 | 0.025 |
| Procyon lotor | Digastric | Fascicle Length (mm) | Minimum Seed Correlation +50% | 55 | 12 | 16.12 | 18.52 | -1.21 | 18.7 | 0.241 |
| Procyon lotor | Digastric | Fascicle Length (mm) | Minimum Seed Correlation -50% | 55 | 58 | 16.12 | 15.73 | 0.28 | 110.7 | 0.778 |
| Procyon lotor | Digastric | Fascicle Length (mm) | Minimum Continuation Quality +50% | 55 | 25 | 16.12 | 11.62 | 3.76 | 77.5 | 0.000 |
| Procyon lotor | Digastric | Fascicle Length (mm) | Minimum Continuation Quality -50% | 55 | 64 | 16.12 | 20.23 | -2.61 | 113.9 | 0.010 |
| Procyon lotor | Digastric | Fascicle Length (mm) | Direction Coefficient 0.2 | 55 | 60 | 16.12 | 15.02 | 0.85 | 109.1 | 0.396 |
| Procyon lotor | Digastric | Fascicle Length (mm) | Direction Coefficient 0.1 | 55 | 51 | 16.12 | 12.53 | 3.05 | 93.8 | 0.003 |
| Procyon lotor | Digastric | Fascicle Length (mm) | Minimum Length 100% Cylinder Length | 55 | 35 | 16.12 | 20.21 | -2.88 | 80.5 | 0.005 |
| Procyon lotor | Digastric | Fascicle Length (mm) | Minimum Length 50% Cylinder Length | 55 | 66 | 16.12 | 14.49 | 1.21 | 116.8 | 0.229 |
| Procyon lotor | Deep masseter | Fascicle Length (mm) | Minimum Seed Correlation +50% | 48 | 32 | 12.50 | 12.59 | -0.20 | 61.1 | 0.842 |
| Procyon lotor | Deep masseter | Fascicle Length (mm) | Minimum Seed Correlation -50% | 48 | 48 | 12.50 | 12.50 | 0.00 | 94 | 1.000 |
| Procyon lotor | Deep masseter | Fascicle Length (mm) | Minimum Continuation Quality +50% | 48 | 10 | 12.50 | 11.86 | 1.03 | 12.8 | 0.321 |
| Procyon lotor | Deep masseter | Fascicle Length (mm) | Minimum Continuation Quality -50% | 48 | 108 | 12.50 | 15.95 | -6.74 | 151.2 | 0.000 |
| Procyon lotor | Deep masseter | Fascicle Length (mm) | Direction Coefficient 0.2 | 48 | 37 | 12.50 | 12.16 | 0.88 | 76.1 | 0.384 |
| Procyon lotor | Deep masseter | Fascicle Length (mm) | Direction Coefficient 0.1 | 48 | 14 | 12.50 | 11.87 | 1.62 | 32.9 | 0.116 |
| Procyon lotor | Deep masseter | Fascicle Length (mm) | Minimum Length 100% Cylinder Length | 48 | 5 | 12.50 | 16.01 | -4.45 | 5 | 0.007 |
| Procyon lotor | Deep masseter | Fascicle Length (mm) | Minimum Length 50% Cylinder Length | 48 | 90 | 12.50 | 10.63 | 5.14 | 126.3 | 0.000 |
| Procyon lotor | Deep temporalis | Fascicle Length (mm) | Minimum Seed Correlation +50% | 72 | 17 | 19.21 | 23.11 | -2.15 | 21.4 | 0.043 |
| Procyon lotor | Deep temporalis | Fascicle Length (mm) | Minimum Seed Correlation -50% | 72 | 78 | 19.21 | 19.23 | -0.02 | 147.9 | 0.983 |
| Procyon lotor | Deep temporalis | Fascicle Length (mm) | Minimum Continuation Quality -50% | 72 | 130 | 19.21 | 25.29 | -4.79 | 195.3 | 0.000 |
| Procyon lotor | Deep temporalis | Fascicle Length (mm) | Direction Coefficient 0.2 | 72 | 64 | 19.21 | 19.20 | 0.01 | 133.5 | 0.991 |
| Procyon lotor | Deep temporalis | Fascicle Length (mm) | Direction Coefficient 0.1 | 72 | 25 | 19.21 | 16.68 | 3.11 | 93 | 0.002 |
| Procyon lotor | Deep temporalis | Fascicle Length (mm) | Minimum Length 100% Cylinder Length | 72 | 39 | 19.21 | 23.54 | -3.64 | 73.4 | 0.000 |
| Procyon lotor | Deep temporalis | Fascicle Length (mm) | Minimum Length 50% Cylinder Length | 72 | 120 | 19.21 | 16.00 | 3.68 | 156.5 | 0.000 |
| Procyon lotor | Lateral pterygoid | Fascicle Length (mm) | Minimum Seed Correlation -50% | 43 | 61 | 4.89 | 4.60 | 0.87 | 86.1 | 0.387 |
| Procyon lotor | Lateral pterygoid | Fascicle Length (mm) | Minimum Continuation Quality +50% | 43 | 7 | 4.89 | 3.44 | 4.33 | 31.3 | 0.000 |
| Procyon lotor | Lateral pterygoid | Fascicle Length (mm) | Minimum Continuation Quality -50% | 43 | 73 | 4.89 | 7.59 | -6.20 | 114 | 0.000 |
| Procyon lotor | Lateral pterygoid | Fascicle Length (mm) | Direction Coefficient 0.2 | 43 | 37 | 4.89 | 4.24 | 1.81 | 78 | 0.075 |
| Procyon lotor | Lateral pterygoid | Fascicle Length (mm) | Direction Coefficient 0.1 | 43 | 28 | 4.89 | 4.01 | 2.44 | 67.9 | 0.017 |
| Procyon lotor | Lateral pterygoid | Fascicle Length (mm) | Minimum Length 100% Cylinder Length | 43 | 26 | 4.89 | 5.91 | -2.58 | 58.8 | 0.013 |
| Procyon lotor | Lateral pterygoid | Fascicle Length (mm) | Minimum Length 50% Cylinder Length | 43 | 62 | 4.89 | 4.14 | 2.11 | 93.9 | 0.038 |
| Procyon lotor | Medial pterygoid | Fascicle Length (mm) | Minimum Seed Correlation +50% | 49 | 4 | 9.69 | 9.47 | 0.16 | 4.3 | 0.884 |
| Procyon lotor | Medial pterygoid | Fascicle Length (mm) | Minimum Seed Correlation -50% | 49 | 58 | 9.69 | 9.23 | 0.59 | 100.4 | 0.556 |
| Procyon lotor | Medial pterygoid | Fascicle Length (mm) | Minimum Continuation Quality +50% | 49 | 17 | 9.69 | 7.66 | 2.48 | 47.5 | 0.017 |
| Procyon lotor | Medial pterygoid | Fascicle Length (mm) | Minimum Continuation Quality -50% | 49 | 66 | 9.69 | 12.51 | -2.89 | 110.3 | 0.005 |
| Procyon lotor | Medial pterygoid | Fascicle Length (mm) | Direction Coefficient 0.2 | 49 | 42 | 9.69 | 9.10 | 0.76 | 88.9 | 0.449 |
| Procyon lotor | Medial pterygoid | Fascicle Length (mm) | Direction Coefficient 0.1 | 49 | 33 | 9.69 | 7.53 | 3.25 | 72.9 | 0.002 |
| Procyon lotor | Medial pterygoid | Fascicle Length (mm) | Minimum Length 100% Cylinder Length | 49 | 28 | 9.69 | 12.10 | -2.63 | 59.5 | 0.011 |
| Procyon lotor | Medial pterygoid | Fascicle Length (mm) | Minimum Length 50% Cylinder Length | 49 | 76 | 9.69 | 7.82 | 2.52 | 103.3 | 0.013 |
| Procyon lotor | Superficial masseter | Fascicle Length (mm) | Minimum Seed Correlation +50% | 50 | 17 | 13.06 | 15.04 | -1.21 | 24.2 | 0.238 |
| Procyon lotor | Superficial masseter | Fascicle Length (mm) | Minimum Seed Correlation -50% | 50 | 61 | 13.06 | 12.45 | 0.64 | 102.8 | 0.523 |
| Procyon lotor | Superficial masseter | Fascicle Length (mm) | Minimum Continuation Quality +50% | 50 | 16 | 13.06 | 8.65 | 5.33 | 64 | 0.000 |
| Procyon lotor | Superficial masseter | Fascicle Length (mm) | Minimum Continuation Quality -50% | 50 | 76 | 13.06 | 15.50 | -2.09 | 123.9 | 0.039 |
| Procyon lotor | Superficial masseter | Fascicle Length (mm) | Direction Coefficient 0.2 | 50 | 57 | 13.06 | 11.65 | 1.52 | 97.1 | 0.132 |
| Procyon lotor | Superficial masseter | Fascicle Length (mm) | Direction Coefficient 0.1 | 50 | 24 | 13.06 | 9.54 | 3.59 | 66.2 | 0.001 |
| Procyon lotor | Superficial masseter | Fascicle Length (mm) | Minimum Length 100% Cylinder Length | 50 | 32 | 13.06 | 15.02 | -1.92 | 76 | 0.059 |
| Procyon lotor | Superficial masseter | Fascicle Length (mm) | Minimum Length 50% Cylinder Length | 50 | 77 | 13.06 | 10.34 | 2.88 | 107.7 | 0.005 |
| Procyon lotor | Superficial temporalis | Fascicle Length (mm) | Minimum Seed Correlation +50% | 75 | 43 | 14.56 | 15.57 | -0.85 | 81.5 | 0.399 |
| Procyon lotor | Superficial temporalis | Fascicle Length (mm) | Minimum Seed Correlation -50% | 75 | 76 | 14.56 | 14.53 | 0.04 | 148.9 | 0.973 |
| Procyon lotor | Superficial temporalis | Fascicle Length (mm) | Minimum Continuation Quality +50% | 75 | 12 | 14.56 | 11.53 | 2.98 | 32.6 | 0.005 |
| Procyon lotor | Superficial temporalis | Fascicle Length (mm) | Minimum Continuation Quality -50% | 75 | 129 | 14.56 | 19.38 | -3.93 | 198.9 | 0.000 |
| Procyon lotor | Superficial temporalis | Fascicle Length (mm) | Direction Coefficient 0.3 | 75 | 98 | 14.56 | 15.10 | -0.58 | 163.9 | 0.565 |
| Procyon lotor | Superficial temporalis | Fascicle Length (mm) | Direction Coefficient 0.1 | 75 | 40 | 14.56 | 11.77 | 3.66 | 104.3 | 0.000 |
| Procyon lotor | Superficial temporalis | Fascicle Length (mm) | Minimum Length 100% Cylinder Length | 75 | 35 | 14.56 | 19.80 | -4.42 | 67.9 | 0.000 |
| Procyon lotor | Superficial temporalis | Fascicle Length (mm) | Minimum Length 50% Cylinder Length | 75 | 138 | 14.56 | 11.12 | 4.22 | 139 | 0.000 |
| Procyon lotor | Zygomaticomandibularis | Fascicle Length (mm) | Minimum Seed Correlation +50% | 53 | 28 | 9.43 | 11.32 | -1.65 | 50 | 0.105 |
| Procyon lotor | Zygomaticomandibularis | Fascicle Length (mm) | Minimum Seed Correlation -50% | 53 | 56 | 9.43 | 9.20 | 0.27 | 106.6 | 0.790 |
| Procyon lotor | Zygomaticomandibularis | Fascicle Length (mm) | Minimum Continuation Quality +50% | 53 | 30 | 9.43 | 6.98 | 3.14 | 81 | 0.002 |
| Procyon lotor | Zygomaticomandibularis | Fascicle Length (mm) | Minimum Continuation Quality -50% | 53 | 59 | 9.43 | 11.38 | -1.79 | 101.5 | 0.076 |
| Procyon lotor | Zygomaticomandibularis | Fascicle Length (mm) | Direction Coefficient 0.2 | 53 | 49 | 9.43 | 8.40 | 1.35 | 92.6 | 0.181 |
| Procyon lotor | Zygomaticomandibularis | Fascicle Length (mm) | Direction Coefficient 0.1 | 53 | 42 | 9.43 | 6.93 | 3.68 | 70.8 | 0.000 |
| Procyon lotor | Zygomaticomandibularis | Fascicle Length (mm) | Minimum Length 100% Cylinder Length | 53 | 42 | 9.43 | 10.69 | -1.39 | 90.3 | 0.168 |
| Procyon lotor | Zygomaticomandibularis | Fascicle Length (mm) | Minimum Length 50% Cylinder Length | 53 | 68 | 9.43 | 8.16 | 1.51 | 113.6 | 0.134 |
| Martes americana | Digastric | Fascicle Length (mm) | Minimum Seed Correlation +50% | 43 | 5 | 17.95 | 20.27 | -0.89 | 4.9 | 0.414 |
| Martes americana | Digastric | Fascicle Length (mm) | Minimum Seed Correlation -50% | 43 | 48 | 17.95 | 17.45 | 0.43 | 88.3 | 0.666 |
| Martes americana | Digastric | Fascicle Length (mm) | Minimum Continuation Quality +50% | 43 | 31 | 17.95 | 14.85 | 2.95 | 71.4 | 0.004 |
| Martes americana | Digastric | Fascicle Length (mm) | Minimum Continuation Quality -50% | 43 | 50 | 17.95 | 22.43 | -3.63 | 91 | 0.000 |
| Martes americana | Digastric | Fascicle Length (mm) | Direction Coefficient 0.2 | 43 | 37 | 17.95 | 18.97 | -0.94 | 77.4 | 0.352 |
| Martes americana | Digastric | Fascicle Length (mm) | Direction Coefficient 0.1 | 43 | 35 | 17.95 | 16.19 | 1.62 | 75.7 | 0.111 |
| Martes americana | Digastric | Fascicle Length (mm) | Minimum Length 100% Cylinder Length | 43 | 26 | 17.95 | 21.00 | -2.80 | 66.4 | 0.007 |
| Martes americana | Digastric | Fascicle Length (mm) | Minimum Length 50% Cylinder Length | 43 | 54 | 17.95 | 16.67 | 1.09 | 93.8 | 0.280 |
| Martes americana | Deep masseter | Fascicle Length (mm) | Minimum Seed Correlation +50% | 36 | 2 | 10.32 | 9.02 | 0.33 | 1 | 0.796 |
| Martes americana | Deep masseter | Fascicle Length (mm) | Minimum Seed Correlation -50% | 36 | 47 | 10.32 | 9.25 | 1.36 | 76.8 | 0.177 |
| Martes americana | Deep masseter | Fascicle Length (mm) | Minimum Continuation Quality +50% | 36 | 15 | 10.32 | 6.81 | 5.01 | 48.8 | 0.000 |
| Martes americana | Deep masseter | Fascicle Length (mm) | Minimum Continuation Quality -50% | 36 | 53 | 10.32 | 11.78 | -1.54 | 86.8 | 0.128 |
| Martes americana | Deep masseter | Fascicle Length (mm) | Direction Coefficient 0.2 | 36 | 38 | 10.32 | 8.30 | 2.74 | 67.2 | 0.008 |
| Martes americana | Deep masseter | Fascicle Length (mm) | Direction Coefficient 0.1 | 36 | 24 | 10.32 | 7.11 | 4.58 | 56.1 | 0.000 |
| Martes americana | Deep masseter | Fascicle Length (mm) | Minimum Length 100% Cylinder Length | 36 | 28 | 10.32 | 11.76 | -1.91 | 61.7 | 0.060 |
| Martes americana | Deep masseter | Fascicle Length (mm) | Minimum Length 50% Cylinder Length | 36 | 52 | 10.32 | 8.50 | 2.25 | 81.7 | 0.027 |
| Martes americana | Deep temporalis | Fascicle Length (mm) | Minimum Seed Correlation +50% | 64 | 3 | 14.50 | 19.83 | -0.92 | 2.1 | 0.452 |
| Martes americana | Deep temporalis | Fascicle Length (mm) | Minimum Seed Correlation -50% | 64 | 75 | 14.50 | 13.95 | 0.54 | 131.4 | 0.592 |
| Martes americana | Deep temporalis | Fascicle Length (mm) | Minimum Continuation Quality +50% | 64 | 20 | 14.50 | 12.40 | 2.21 | 74.9 | 0.031 |
| Martes americana | Deep temporalis | Fascicle Length (mm) | Minimum Continuation Quality -50% | 64 | 109 | 14.50 | 15.80 | -1.23 | 157.3 | 0.222 |
| Martes americana | Deep temporalis | Fascicle Length (mm) | Direction Coefficient 0.3 | 64 | 96 | 14.50 | 16.13 | -1.48 | 154.1 | 0.141 |
| Martes americana | Deep temporalis | Fascicle Length (mm) | Direction Coefficient 0.1 | 64 | 42 | 14.50 | 12.60 | 1.98 | 103.7 | 0.050 |
| Martes americana | Deep temporalis | Fascicle Length (mm) | Minimum Length 100% Cylinder Length | 64 | 30 | 14.50 | 18.69 | -3.10 | 56.5 | 0.003 |
| Martes americana | Deep temporalis | Fascicle Length (mm) | Minimum Length 50% Cylinder Length | 64 | 118 | 14.50 | 11.09 | 3.77 | 115.5 | 0.000 |
| Martes americana | Lateral pterygoid | Fascicle Length (mm) | Minimum Seed Correlation +50% | 28 | 8 | 4.27 | 3.83 | 0.92 | 23.9 | 0.366 |
| Martes americana | Lateral pterygoid | Fascicle Length (mm) | Minimum Seed Correlation -50% | 28 | 23 | 4.27 | 4.06 | 0.52 | 43 | 0.608 |
| Martes americana | Lateral pterygoid | Fascicle Length (mm) | Minimum Continuation Quality +50% | 28 | 10 | 4.27 | 3.50 | 2.13 | 31.4 | 0.041 |
| Martes americana | Lateral pterygoid | Fascicle Length (mm) | Minimum Continuation Quality -50% | 28 | 27 | 4.27 | 4.50 | -0.49 | 52.5 | 0.623 |
| Martes americana | Lateral pterygoid | Fascicle Length (mm) | Direction Coefficient 0.2 | 28 | 18 | 4.27 | 3.56 | 1.88 | 36.4 | 0.069 |
| Martes americana | Lateral pterygoid | Fascicle Length (mm) | Direction Coefficient 0.1 | 28 | 12 | 4.27 | 3.06 | 3.30 | 32.8 | 0.002 |
| Martes americana | Lateral pterygoid | Fascicle Length (mm) | Minimum Length 100% Cylinder Length | 28 | 12 | 4.27 | 4.74 | -1.11 | 37.9 | 0.272 |
| Martes americana | Lateral pterygoid | Fascicle Length (mm) | Minimum Length 50% Cylinder Length | 28 | 28 | 4.27 | 3.45 | 1.96 | 47.2 | 0.056 |
| Martes americana | Medial pterygoid | Fascicle Length (mm) | Minimum Seed Correlation +50% | 36 | 3 | 13.04 | 16.85 | -5.14 | 36.9 | 0.000 |
| Martes americana | Medial pterygoid | Fascicle Length (mm) | Minimum Seed Correlation -50% | 36 | 50 | 13.04 | 12.30 | 0.79 | 74.8 | 0.432 |
| Martes americana | Medial pterygoid | Fascicle Length (mm) | Minimum Continuation Quality +50% | 36 | 24 | 13.04 | 10.86 | 2.30 | 57.9 | 0.025 |
| Martes americana | Medial pterygoid | Fascicle Length (mm) | Minimum Continuation Quality -50% | 36 | 41 | 13.04 | 16.43 | -2.90 | 73 | 0.005 |
| Martes americana | Medial pterygoid | Fascicle Length (mm) | Direction Coefficient 0.2 | 36 | 29 | 13.04 | 12.08 | 0.97 | 62.8 | 0.336 |
| Martes americana | Medial pterygoid | Fascicle Length (mm) | Direction Coefficient 0.1 | 36 | 21 | 13.04 | 9.70 | 3.81 | 54.5 | 0.000 |
| Martes americana | Medial pterygoid | Fascicle Length (mm) | Minimum Length 100% Cylinder Length | 36 | 29 | 13.04 | 14.73 | -1.74 | 63 | 0.088 |
| Martes americana | Medial pterygoid | Fascicle Length (mm) | Minimum Length 50% Cylinder Length | 36 | 41 | 13.04 | 12.17 | 0.85 | 74.5 | 0.396 |
| Martes americana | Superficial masseter | Fascicle Length (mm) | Minimum Seed Correlation +50% | 67 | 5 | 7.92 | 8.77 | -0.46 | 4.3 | 0.670 |
| Martes americana | Superficial masseter | Fascicle Length (mm) | Minimum Seed Correlation -50% | 67 | 93 | 7.92 | 7.07 | 2.05 | 131.3 | 0.042 |
| Martes americana | Superficial masseter | Fascicle Length (mm) | Minimum Continuation Quality +50% | 67 | 23 | 7.92 | 5.80 | 4.55 | 67.7 | 0.000 |
| Martes americana | Superficial masseter | Fascicle Length (mm) | Minimum Continuation Quality -50% | 67 | 89 | 7.92 | 11.14 | -5.14 | 141.9 | 0.000 |
| Martes americana | Superficial masseter | Fascicle Length (mm) | Direction Coefficient 0.2 | 67 | 71 | 7.92 | 8.42 | -1.00 | 135.1 | 0.320 |
| Martes americana | Superficial masseter | Fascicle Length (mm) | Direction Coefficient 0.1 | 67 | 62 | 7.92 | 7.27 | 1.44 | 126.6 | 0.152 |
| Martes americana | Superficial masseter | Fascicle Length (mm) | Minimum Length 100% Cylinder Length | 67 | 48 | 7.92 | 8.73 | -1.74 | 111.1 | 0.085 |
| Martes americana | Superficial masseter | Fascicle Length (mm) | Minimum Length 50% Cylinder Length | 67 | 88 | 7.92 | 6.95 | 2.11 | 148.1 | 0.037 |
| Martes americana | Superficial temporalis | Fascicle Length (mm) | Minimum Seed Correlation +50% | 54 | 3 | 11.19 | 10.89 | 0.28 | 6.1 | 0.789 |
| Martes americana | Superficial temporalis | Fascicle Length (mm) | Minimum Seed Correlation -50% | 54 | 63 | 11.19 | 10.77 | 0.45 | 110.8 | 0.656 |
| Martes americana | Superficial temporalis | Fascicle Length (mm) | Minimum Continuation Quality +50% | 54 | 27 | 11.19 | 7.75 | 4.57 | 67.4 | 0.000 |
| Martes americana | Superficial temporalis | Fascicle Length (mm) | Minimum Continuation Quality -50% | 54 | 67 | 11.19 | 13.36 | -1.58 | 104 | 0.118 |
| Martes americana | Superficial temporalis | Fascicle Length (mm) | Direction Coefficient 0.3 | 54 | 68 | 11.19 | 13.54 | -1.79 | 108.7 | 0.076 |
| Martes americana | Superficial temporalis | Fascicle Length (mm) | Direction Coefficient 0.1 | 54 | 46 | 11.19 | 9.18 | 2.45 | 86.4 | 0.017 |
| Martes americana | Superficial temporalis | Fascicle Length (mm) | Minimum Length 100% Cylinder Length | 54 | 34 | 11.19 | 15.37 | -3.40 | 62.9 | 0.001 |
| Martes americana | Superficial temporalis | Fascicle Length (mm) | Minimum Length 50% Cylinder Length | 54 | 82 | 11.19 | 8.70 | 2.77 | 113.4 | 0.007 |
| Martes americana | Zygomaticomandibularis | Fascicle Length (mm) | Minimum Seed Correlation -50% | 39 | 45 | 10.64 | 10.32 | 0.43 | 80 | 0.672 |
| Martes americana | Zygomaticomandibularis | Fascicle Length (mm) | Minimum Continuation Quality +50% | 39 | 19 | 10.64 | 9.05 | 2.12 | 50.9 | 0.039 |
| Martes americana | Zygomaticomandibularis | Fascicle Length (mm) | Minimum Continuation Quality -50% | 39 | 54 | 10.64 | 13.85 | -2.83 | 79.6 | 0.006 |
| Martes americana | Zygomaticomandibularis | Fascicle Length (mm) | Direction Coefficient 0.2 | 39 | 35 | 10.64 | 10.40 | 0.34 | 69.9 | 0.735 |
| Martes americana | Zygomaticomandibularis | Fascicle Length (mm) | Direction Coefficient 0.1 | 39 | 21 | 10.64 | 8.14 | 3.83 | 57.4 | 0.000 |
| Martes americana | Zygomaticomandibularis | Fascicle Length (mm) | Minimum Length 100% Cylinder Length | 39 | 25 | 10.64 | 12.74 | -2.62 | 56.6 | 0.011 |
| Martes americana | Zygomaticomandibularis | Fascicle Length (mm) | Minimum Length 50% Cylinder Length | 39 | 51 | 10.64 | 9.30 | 1.80 | 83.9 | 0.076 |
| Mephitis mephitis | Digastric | Fascicle Length (mm) | Minimum Seed Correlation +50% | 54 | 17 | 11.84 | 16.06 | -2.34 | 23.5 | 0.028 |
| Mephitis mephitis | Digastric | Fascicle Length (mm) | Minimum Seed Correlation -50% | 54 | 61 | 11.84 | 11.43 | 0.40 | 110.5 | 0.692 |
| Mephitis mephitis | Digastric | Fascicle Length (mm) | Minimum Continuation Quality +50% | 54 | 31 | 11.84 | 8.52 | 3.60 | 82 | 0.001 |
| Mephitis mephitis | Digastric | Fascicle Length (mm) | Minimum Continuation Quality -50% | 54 | 65 | 11.84 | 14.48 | -2.08 | 113.5 | 0.040 |
| Mephitis mephitis | Digastric | Fascicle Length (mm) | Direction Coefficient 0.2 | 54 | 52 | 11.84 | 10.93 | 0.87 | 103.6 | 0.389 |
| Mephitis mephitis | Digastric | Fascicle Length (mm) | Direction Coefficient 0.1 | 54 | 43 | 11.84 | 10.04 | 1.68 | 94.3 | 0.096 |
| Mephitis mephitis | Digastric | Fascicle Length (mm) | Minimum Length 100% Cylinder Length | 54 | 34 | 11.84 | 14.86 | -2.56 | 74.1 | 0.013 |
| Mephitis mephitis | Digastric | Fascicle Length (mm) | Minimum Length 50% Cylinder Length | 54 | 70 | 11.84 | 10.23 | 1.55 | 115.7 | 0.124 |
| Mephitis mephitis | Deep masseter | Fascicle Length (mm) | Minimum Seed Correlation +50% | 60 | 39 | 6.36 | 6.48 | -0.61 | 74 | 0.541 |
| Mephitis mephitis | Deep masseter | Fascicle Length (mm) | Minimum Seed Correlation -50% | 60 | 60 | 6.36 | 6.36 | 0.00 | 118 | 1.000 |
| Mephitis mephitis | Deep masseter | Fascicle Length (mm) | Minimum Continuation Quality -50% | 60 | 527 | 6.36 | 8.13 | -11.76 | 140 | 0.000 |
| Mephitis mephitis | Deep masseter | Fascicle Length (mm) | Direction Coefficient 0.3 | 60 | 118 | 6.36 | 6.75 | -2.39 | 151.8 | 0.018 |
| Mephitis mephitis | Deep masseter | Fascicle Length (mm) | Direction Coefficient 0.1 | 60 | 6 | 6.36 | 5.95 | 2.11 | 12.6 | 0.056 |
| Mephitis mephitis | Deep masseter | Fascicle Length (mm) | Minimum Length 100% Cylinder Length | 60 | 3 | 6.36 | 9.35 | -4.45 | 2.1 | 0.042 |
| Mephitis mephitis | Deep masseter | Fascicle Length (mm) | Minimum Length 50% Cylinder Length | 60 | 212 | 6.36 | 5.12 | 8.92 | 100.7 | 0.000 |
| Mephitis mephitis | Deep temporalis | Fascicle Length (mm) | Minimum Seed Correlation -50% | 59 | 110 | 14.63 | 13.35 | 2.10 | 111.3 | 0.038 |
| Mephitis mephitis | Deep temporalis | Fascicle Length (mm) | Minimum Continuation Quality +50% | 59 | 2 | 14.63 | 12.80 | 3.16 | 14.2 | 0.007 |
| Mephitis mephitis | Deep temporalis | Fascicle Length (mm) | Minimum Continuation Quality -50% | 59 | 181 | 14.63 | 22.43 | -9.91 | 206.5 | 0.000 |
| Mephitis mephitis | Deep temporalis | Fascicle Length (mm) | Direction Coefficient 0.2 | 59 | 40 | 14.63 | 13.65 | 1.38 | 93.4 | 0.170 |
| Mephitis mephitis | Deep temporalis | Fascicle Length (mm) | Direction Coefficient 0.1 | 59 | 13 | 14.63 | 12.45 | 2.60 | 27.6 | 0.015 |
| Mephitis mephitis | Deep temporalis | Fascicle Length (mm) | Minimum Length 100% Cylinder Length | 59 | 27 | 14.63 | 18.26 | -4.92 | 68.4 | 0.000 |
| Mephitis mephitis | Deep temporalis | Fascicle Length (mm) | Minimum Length 50% Cylinder Length | 59 | 96 | 14.63 | 11.94 | 3.99 | 134.4 | 0.000 |
| Mephitis mephitis | Lateral pterygoid | Fascicle Length (mm) | Minimum Seed Correlation +50% | 39 | 24 | 3.85 | 4.11 | -1.23 | 52.2 | 0.225 |
| Mephitis mephitis | Lateral pterygoid | Fascicle Length (mm) | Minimum Seed Correlation -50% | 39 | 40 | 3.85 | 3.81 | 0.17 | 77 | 0.868 |
| Mephitis mephitis | Lateral pterygoid | Fascicle Length (mm) | Minimum Continuation Quality +50% | 39 | 18 | 3.85 | 3.18 | 3.39 | 47 | 0.001 |
| Mephitis mephitis | Lateral pterygoid | Fascicle Length (mm) | Minimum Continuation Quality -50% | 39 | 61 | 3.85 | 4.14 | -1.50 | 92.7 | 0.138 |
| Mephitis mephitis | Lateral pterygoid | Fascicle Length (mm) | Direction Coefficient 0.2 | 39 | 38 | 3.85 | 3.49 | 1.89 | 74.8 | 0.062 |
| Mephitis mephitis | Lateral pterygoid | Fascicle Length (mm) | Direction Coefficient 0.1 | 39 | 23 | 3.85 | 3.43 | 1.91 | 48.6 | 0.062 |
| Mephitis mephitis | Lateral pterygoid | Fascicle Length (mm) | Minimum Length 100% Cylinder Length | 39 | 20 | 3.85 | 4.39 | -3.18 | 57 | 0.002 |
| Mephitis mephitis | Lateral pterygoid | Fascicle Length (mm) | Minimum Length 50% Cylinder Length | 39 | 64 | 3.85 | 3.24 | 3.14 | 92.3 | 0.002 |
| Mephitis mephitis | Medial pterygoid | Fascicle Length (mm) | Minimum Seed Correlation +50% | 34 | 9 | 7.36 | 7.89 | -0.63 | 9.9 | 0.543 |
| Mephitis mephitis | Medial pterygoid | Fascicle Length (mm) | Minimum Seed Correlation -50% | 34 | 38 | 7.36 | 7.20 | 0.42 | 69.1 | 0.673 |
| Mephitis mephitis | Medial pterygoid | Fascicle Length (mm) | Minimum Continuation Quality +50% | 34 | 7 | 7.36 | 6.14 | 2.77 | 14.9 | 0.014 |
| Mephitis mephitis | Medial pterygoid | Fascicle Length (mm) | Minimum Continuation Quality -50% | 34 | 75 | 7.36 | 9.32 | -4.76 | 99.5 | 0.000 |
| Mephitis mephitis | Medial pterygoid | Fascicle Length (mm) | Direction Coefficient 0.2 | 34 | 24 | 7.36 | 7.05 | 0.80 | 54 | 0.430 |
| Mephitis mephitis | Medial pterygoid | Fascicle Length (mm) | Direction Coefficient 0.1 | 34 | 11 | 7.36 | 6.54 | 2.03 | 27.9 | 0.052 |
| Mephitis mephitis | Medial pterygoid | Fascicle Length (mm) | Minimum Length 100% Cylinder Length | 34 | 18 | 7.36 | 8.58 | -3.02 | 42.2 | 0.004 |
| Mephitis mephitis | Medial pterygoid | Fascicle Length (mm) | Minimum Length 50% Cylinder Length | 34 | 38 | 7.36 | 6.83 | 1.31 | 70 | 0.195 |
| Mephitis mephitis | Superficial masseter | Fascicle Length (mm) | Minimum Seed Correlation -50% | 57 | 71 | 6.58 | 6.37 | 0.71 | 120 | 0.479 |
| Mephitis mephitis | Superficial masseter | Fascicle Length (mm) | Minimum Continuation Quality +50% | 57 | 6 | 6.58 | 5.19 | 2.77 | 7.5 | 0.026 |
| Mephitis mephitis | Superficial masseter | Fascicle Length (mm) | Minimum Continuation Quality -50% | 57 | 117 | 6.58 | 10.14 | -6.32 | 150.2 | 0.000 |
| Mephitis mephitis | Superficial masseter | Fascicle Length (mm) | Direction Coefficient 0.2 | 57 | 46 | 6.58 | 6.19 | 1.31 | 100.1 | 0.195 |
| Mephitis mephitis | Superficial masseter | Fascicle Length (mm) | Direction Coefficient 0.1 | 57 | 26 | 6.58 | 5.56 | 3.13 | 62.6 | 0.003 |
| Mephitis mephitis | Superficial masseter | Fascicle Length (mm) | Minimum Length 100% Cylinder Length | 57 | 30 | 6.58 | 7.71 | -3.27 | 64.6 | 0.002 |
| Mephitis mephitis | Superficial masseter | Fascicle Length (mm) | Minimum Length 50% Cylinder Length | 57 | 96 | 6.58 | 5.50 | 3.69 | 134.1 | 0.000 |
| Mephitis mephitis | Superficial temporalis | Fascicle Length (mm) | Minimum Seed Correlation +50% | 65 | 20 | 9.15 | 10.79 | -1.64 | 28.6 | 0.111 |
| Mephitis mephitis | Superficial temporalis | Fascicle Length (mm) | Minimum Seed Correlation -50% | 65 | 67 | 9.15 | 9.07 | 0.13 | 129.8 | 0.896 |
| Mephitis mephitis | Superficial temporalis | Fascicle Length (mm) | Minimum Continuation Quality +50% | 65 | 21 | 9.15 | 6.90 | 4.67 | 82.6 | 0.000 |
| Mephitis mephitis | Superficial temporalis | Fascicle Length (mm) | Minimum Continuation Quality -50% | 65 | 77 | 9.15 | 11.39 | -2.54 | 118.5 | 0.012 |
| Mephitis mephitis | Superficial temporalis | Fascicle Length (mm) | Direction Coefficient 0.3 | 65 | 69 | 9.15 | 10.42 | -1.68 | 121.4 | 0.096 |
| Mephitis mephitis | Superficial temporalis | Fascicle Length (mm) | Direction Coefficient 0.1 | 65 | 45 | 9.15 | 7.52 | 3.08 | 104.7 | 0.003 |
| Mephitis mephitis | Superficial temporalis | Fascicle Length (mm) | Minimum Length 100% Cylinder Length | 65 | 34 | 9.15 | 12.18 | -4.42 | 75.4 | 0.000 |
| Mephitis mephitis | Superficial temporalis | Fascicle Length (mm) | Minimum Length 50% Cylinder Length | 65 | 95 | 9.15 | 7.83 | 2.34 | 137.6 | 0.021 |
| Mephitis mephitis | Zygomaticomandibularis | Fascicle Length (mm) | Minimum Seed Correlation +50% | 55 | 3 | 5.64 | 5.99 | -0.33 | 2.2 | 0.772 |
| Mephitis mephitis | Zygomaticomandibularis | Fascicle Length (mm) | Minimum Seed Correlation -50% | 55 | 71 | 5.64 | 5.42 | 0.81 | 113.6 | 0.422 |
| Mephitis mephitis | Zygomaticomandibularis | Fascicle Length (mm) | Minimum Continuation Quality +50% | 55 | 5 | 5.64 | 4.19 | 4.89 | 15.7 | 0.000 |
| Mephitis mephitis | Zygomaticomandibularis | Fascicle Length (mm) | Minimum Continuation Quality -50% | 55 | 84 | 5.64 | 8.29 | -6.01 | 123.9 | 0.000 |
| Mephitis mephitis | Zygomaticomandibularis | Fascicle Length (mm) | Direction Coefficient 0.2 | 55 | 37 | 5.64 | 5.59 | 0.16 | 84.5 | 0.874 |
| Mephitis mephitis | Zygomaticomandibularis | Fascicle Length (mm) | Direction Coefficient 0.1 | 55 | 25 | 5.64 | 4.72 | 3.41 | 76.2 | 0.001 |
| Mephitis mephitis | Zygomaticomandibularis | Fascicle Length (mm) | Minimum Length 100% Cylinder Length | 55 | 28 | 5.64 | 6.85 | -3.71 | 64.4 | 0.000 |
| Mephitis mephitis | Zygomaticomandibularis | Fascicle Length (mm) | Minimum Length 50% Cylinder Length | 55 | 69 | 5.64 | 5.03 | 2.00 | 121.5 | 0.048 |
| Daubentonia madagascariensis | Deep masseter | Fascicle Length (mm) | Minimum Seed Correlation +50% | 64 | 2 | 8.47 | 5.73 | 2.71 | 1.4 | 0.169 |
| Daubentonia madagascariensis | Deep masseter | Fascicle Length (mm) | Minimum Seed Correlation -50% | 64 | 119 | 8.47 | 7.62 | 1.85 | 120.5 | 0.067 |
| Daubentonia madagascariensis | Deep masseter | Fascicle Length (mm) | Minimum Continuation Quality +50% | 64 | 2 | 8.47 | 4.88 | 7.84 | 9.1 | 0.000 |
| Daubentonia madagascariensis | Deep masseter | Fascicle Length (mm) | Minimum Continuation Quality -50% | 64 | 75 | 8.47 | 12.66 | -5.68 | 118.2 | 0.000 |
| Daubentonia madagascariensis | Deep masseter | Fascicle Length (mm) | Direction Coefficient 0.2 | 64 | 57 | 8.47 | 7.77 | 1.33 | 119 | 0.185 |
| Daubentonia madagascariensis | Deep masseter | Fascicle Length (mm) | Direction Coefficient 0.1 | 64 | 44 | 8.47 | 6.56 | 4.09 | 104.4 | 0.000 |
| Daubentonia madagascariensis | Deep masseter | Fascicle Length (mm) | Minimum Length 100% Cylinder Length | 64 | 44 | 8.47 | 10.12 | -3.03 | 100.4 | 0.003 |
| Daubentonia madagascariensis | Deep masseter | Fascicle Length (mm) | Minimum Length 50% Cylinder Length | 64 | 81 | 8.47 | 7.51 | 1.82 | 139.5 | 0.071 |
| Daubentonia madagascariensis | Deep temporalis | Fascicle Length (mm) | Minimum Seed Correlation +50% | 93 | 28 | 13.34 | 15.13 | -1.37 | 38.6 | 0.178 |
| Daubentonia madagascariensis | Deep temporalis | Fascicle Length (mm) | Minimum Seed Correlation -50% | 93 | 110 | 13.34 | 12.93 | 0.57 | 194.1 | 0.570 |
| Daubentonia madagascariensis | Deep temporalis | Fascicle Length (mm) | Minimum Continuation Quality +50% | 93 | 60 | 13.34 | 10.88 | 4.05 | 135.5 | 0.000 |
| Daubentonia madagascariensis | Deep temporalis | Fascicle Length (mm) | Minimum Continuation Quality -50% | 93 | 109 | 13.34 | 16.12 | -3.06 | 190.2 | 0.003 |
| Daubentonia madagascariensis | Deep temporalis | Fascicle Length (mm) | Direction Coefficient 0.2 | 93 | 106 | 13.34 | 13.72 | -0.52 | 194.5 | 0.604 |
| Daubentonia madagascariensis | Deep temporalis | Fascicle Length (mm) | Direction Coefficient 0.1 | 93 | 77 | 13.34 | 11.64 | 2.82 | 137.3 | 0.006 |
| Daubentonia madagascariensis | Deep temporalis | Fascicle Length (mm) | Minimum Length 100% Cylinder Length | 93 | 57 | 13.34 | 15.70 | -2.73 | 119.2 | 0.007 |
| Daubentonia madagascariensis | Deep temporalis | Fascicle Length (mm) | Minimum Length 50% Cylinder Length | 93 | 128 | 13.34 | 11.88 | 2.03 | 203.6 | 0.043 |
| Daubentonia madagascariensis | Lateral pterygoid | Fascicle Length (mm) | Minimum Seed Correlation +50% | 52 | 17 | 11.01 | 15.31 | -3.91 | 30.3 | 0.000 |
| Daubentonia madagascariensis | Lateral pterygoid | Fascicle Length (mm) | Minimum Seed Correlation -50% | 52 | 55 | 11.01 | 10.76 | 0.30 | 104.7 | 0.762 |
| Daubentonia madagascariensis | Lateral pterygoid | Fascicle Length (mm) | Minimum Continuation Quality +50% | 52 | 21 | 11.01 | 9.03 | 2.40 | 59.4 | 0.019 |
| Daubentonia madagascariensis | Lateral pterygoid | Fascicle Length (mm) | Minimum Continuation Quality -50% | 52 | 59 | 11.01 | 12.26 | -1.30 | 105.5 | 0.197 |
| Daubentonia madagascariensis | Lateral pterygoid | Fascicle Length (mm) | Direction Coefficient 0.2 | 52 | 50 | 11.01 | 10.02 | 1.24 | 99 | 0.218 |
| Daubentonia madagascariensis | Lateral pterygoid | Fascicle Length (mm) | Direction Coefficient 0.1 | 52 | 37 | 11.01 | 8.60 | 3.11 | 87 | 0.003 |
| Daubentonia madagascariensis | Lateral pterygoid | Fascicle Length (mm) | Minimum Length 100% Cylinder Length | 52 | 35 | 11.01 | 12.52 | -1.65 | 74.6 | 0.103 |
| Daubentonia madagascariensis | Lateral pterygoid | Fascicle Length (mm) | Minimum Length 50% Cylinder Length | 52 | 68 | 11.01 | 9.55 | 1.79 | 113.7 | 0.077 |
| Daubentonia madagascariensis | Medial pterygoid | Fascicle Length (mm) | Minimum Seed Correlation +50% | 55 | 13 | 8.78 | 8.71 | 0.09 | 21.9 | 0.931 |
| Daubentonia madagascariensis | Medial pterygoid | Fascicle Length (mm) | Minimum Seed Correlation -50% | 55 | 66 | 8.78 | 8.40 | 0.67 | 113.1 | 0.507 |
| Daubentonia madagascariensis | Medial pterygoid | Fascicle Length (mm) | Minimum Continuation Quality +50% | 55 | 14 | 8.78 | 6.49 | 4.08 | 54.2 | 0.000 |
| Daubentonia madagascariensis | Medial pterygoid | Fascicle Length (mm) | Minimum Continuation Quality -50% | 55 | 94 | 8.78 | 11.87 | -3.96 | 145.5 | 0.000 |
| Daubentonia madagascariensis | Medial pterygoid | Fascicle Length (mm) | Direction Coefficient 0.2 | 55 | 46 | 8.78 | 8.15 | 1.14 | 97 | 0.259 |
| Daubentonia madagascariensis | Medial pterygoid | Fascicle Length (mm) | Direction Coefficient 0.1 | 55 | 39 | 8.78 | 7.24 | 3.23 | 72.6 | 0.002 |
| Daubentonia madagascariensis | Medial pterygoid | Fascicle Length (mm) | Minimum Length 100% Cylinder Length | 55 | 27 | 8.78 | 11.37 | -3.39 | 52.2 | 0.001 |
| Daubentonia madagascariensis | Medial pterygoid | Fascicle Length (mm) | Minimum Length 50% Cylinder Length | 55 | 95 | 8.78 | 7.12 | 3.08 | 107.5 | 0.003 |
| Daubentonia madagascariensis | Superficial masseter | Fascicle Length (mm) | Minimum Seed Correlation +50% | 60 | 14 | 8.90 | 9.88 | -1.23 | 21.7 | 0.232 |
| Daubentonia madagascariensis | Superficial masseter | Fascicle Length (mm) | Minimum Seed Correlation -50% | 60 | 63 | 8.90 | 8.85 | 0.10 | 120.6 | 0.919 |
| Daubentonia madagascariensis | Superficial masseter | Fascicle Length (mm) | Minimum Continuation Quality +50% | 60 | 9 | 8.90 | 6.29 | 4.90 | 29.8 | 0.000 |
| Daubentonia madagascariensis | Superficial masseter | Fascicle Length (mm) | Minimum Continuation Quality -50% | 60 | 90 | 8.90 | 13.78 | -4.90 | 117.2 | 0.000 |
| Daubentonia madagascariensis | Superficial masseter | Fascicle Length (mm) | Direction Coefficient 0.2 | 60 | 55 | 8.90 | 8.11 | 1.59 | 111.1 | 0.116 |
| Daubentonia madagascariensis | Superficial masseter | Fascicle Length (mm) | Direction Coefficient 0.1 | 60 | 33 | 8.90 | 7.17 | 3.70 | 90.8 | 0.000 |
| Daubentonia madagascariensis | Superficial masseter | Fascicle Length (mm) | Minimum Length 100% Cylinder Length | 60 | 32 | 8.90 | 11.19 | -3.71 | 68 | 0.000 |
| Daubentonia madagascariensis | Superficial masseter | Fascicle Length (mm) | Minimum Length 50% Cylinder Length | 60 | 79 | 8.90 | 7.65 | 2.39 | 131.1 | 0.018 |
| Daubentonia madagascariensis | Superficial temporalis | Fascicle Length (mm) | Minimum Seed Correlation +50% | 95 | 26 | 12.97 | 16.83 | -1.93 | 31 | 0.062 |
| Daubentonia madagascariensis | Superficial temporalis | Fascicle Length (mm) | Minimum Seed Correlation -50% | 95 | 99 | 12.97 | 12.83 | 0.15 | 191.4 | 0.879 |
| Daubentonia madagascariensis | Superficial temporalis | Fascicle Length (mm) | Minimum Continuation Quality +50% | 95 | 55 | 12.97 | 10.59 | 2.90 | 147.9 | 0.004 |
| Daubentonia madagascariensis | Superficial temporalis | Fascicle Length (mm) | Minimum Continuation Quality -50% | 95 | 108 | 12.97 | 14.91 | -1.99 | 200.1 | 0.048 |
| Daubentonia madagascariensis | Superficial temporalis | Fascicle Length (mm) | Direction Coefficient 0.3 | 95 | 101 | 12.97 | 14.80 | -1.98 | 194 | 0.049 |
| Daubentonia madagascariensis | Superficial temporalis | Fascicle Length (mm) | Direction Coefficient 0.1 | 95 | 65 | 12.97 | 11.62 | 1.57 | 157.7 | 0.117 |
| Daubentonia madagascariensis | Superficial temporalis | Fascicle Length (mm) | Minimum Length 100% Cylinder Length | 95 | 48 | 12.97 | 17.15 | -3.50 | 86.2 | 0.001 |
| Daubentonia madagascariensis | Superficial temporalis | Fascicle Length (mm) | Minimum Length 50% Cylinder Length | 95 | 141 | 12.97 | 10.74 | 2.72 | 194.2 | 0.007 |
| Daubentonia madagascariensis | Zygomaticomandibularis | Fascicle Length (mm) | Minimum Seed Correlation +50% | 43 | 16 | 9.93 | 10.88 | -0.94 | 25.5 | 0.354 |
| Daubentonia madagascariensis | Zygomaticomandibularis | Fascicle Length (mm) | Minimum Seed Correlation -50% | 43 | 43 | 9.93 | 9.93 | 0.00 | 84 | 1.000 |
| Daubentonia madagascariensis | Zygomaticomandibularis | Fascicle Length (mm) | Minimum Continuation Quality +50% | 43 | 18 | 9.93 | 8.02 | 2.82 | 53 | 0.007 |
| Daubentonia madagascariensis | Zygomaticomandibularis | Fascicle Length (mm) | Minimum Continuation Quality -50% | 43 | 49 | 9.93 | 11.34 | -1.82 | 89.3 | 0.072 |
| Daubentonia madagascariensis | Zygomaticomandibularis | Fascicle Length (mm) | Direction Coefficient 0.2 | 43 | 37 | 9.93 | 9.26 | 0.99 | 77.9 | 0.327 |
| Daubentonia madagascariensis | Zygomaticomandibularis | Fascicle Length (mm) | Direction Coefficient 0.1 | 43 | 25 | 9.93 | 7.45 | 4.18 | 63.8 | 0.000 |
| Daubentonia madagascariensis | Zygomaticomandibularis | Fascicle Length (mm) | Minimum Length 100% Cylinder Length | 43 | 23 | 9.93 | 12.05 | -2.95 | 57 | 0.005 |
| Daubentonia madagascariensis | Zygomaticomandibularis | Fascicle Length (mm) | Minimum Length 50% Cylinder Length | 43 | 52 | 9.93 | 8.99 | 1.31 | 92.1 | 0.195 |
| Eulemur coronatus | Anterior digastric | Tortuosity | Minimum Seed Correlation +50% | 41 | 15 | 1.01 | 1.01 | 0.67 | 29.8 | 0.507 |
| Eulemur coronatus | Anterior digastric | Tortuosity | Minimum Seed Correlation -50% | 41 | 45 | 1.01 | 1.01 | -0.05 | 83.6 | 0.959 |
| Eulemur coronatus | Anterior digastric | Tortuosity | Minimum Continuation Quality +50% | 41 | 25 | 1.01 | 1.00 | 3.41 | 63.7 | 0.001 |
| Eulemur coronatus | Anterior digastric | Tortuosity | Minimum Continuation Quality -50% | 41 | 42 | 1.01 | 1.01 | -2.09 | 77.8 | 0.040 |
| Eulemur coronatus | Anterior digastric | Tortuosity | Direction Coefficient 0.2 | 41 | 39 | 1.01 | 1.01 | 1.78 | 77.6 | 0.079 |
| Eulemur coronatus | Anterior digastric | Tortuosity | Direction Coefficient 0.1 | 41 | 19 | 1.01 | 1.00 | 4.71 | 55 | 0.000 |
| Eulemur coronatus | Anterior digastric | Tortuosity | Minimum Length 100% Cylinder Length | 41 | 25 | 1.01 | 1.01 | -1.23 | 51.8 | 0.225 |
| Eulemur coronatus | Anterior digastric | Tortuosity | Minimum Length 50% Cylinder Length | 41 | 47 | 1.01 | 1.01 | 0.38 | 84.3 | 0.707 |
| Eulemur coronatus | Deep masseter | Tortuosity | Minimum Seed Correlation +50% | 52 | 5 | 1.04 | 0.00 | 111.85 | 51 | 0.000 |
| Eulemur coronatus | Deep masseter | Tortuosity | Minimum Seed Correlation -50% | 52 | 55 | 1.04 | 0.98 | 1.64 | 62.3 | 0.106 |
| Eulemur coronatus | Deep masseter | Tortuosity | Minimum Continuation Quality +50% | 52 | 30 | 1.04 | 1.01 | 3.21 | 53.1 | 0.002 |
| Eulemur coronatus | Deep masseter | Tortuosity | Minimum Continuation Quality -50% | 52 | 45 | 1.04 | 1.18 | -1.94 | 45.5 | 0.059 |
| Eulemur coronatus | Deep masseter | Tortuosity | Direction Coefficient 0.2 | 52 | 49 | 1.04 | 1.02 | 2.36 | 59.3 | 0.021 |
| Eulemur coronatus | Deep masseter | Tortuosity | Direction Coefficient 0.1 | 52 | 26 | 1.04 | 1.01 | 3.54 | 53.9 | 0.001 |
| Eulemur coronatus | Deep masseter | Tortuosity | Minimum Length 100% Cylinder Length | 52 | 35 | 1.04 | 1.05 | -0.87 | 65.3 | 0.386 |
| Eulemur coronatus | Deep masseter | Tortuosity | Minimum Length 50% Cylinder Length | 52 | 56 | 1.04 | 1.04 | 0.32 | 104.9 | 0.753 |
| Eulemur coronatus | Deep temporalis | Tortuosity | Minimum Seed Correlation +50% | 58 | 14 | 1.04 | 1.05 | -0.57 | 17.2 | 0.575 |
| Eulemur coronatus | Deep temporalis | Tortuosity | Minimum Seed Correlation -50% | 58 | 61 | 1.04 | 1.04 | 0.17 | 116.5 | 0.869 |
| Eulemur coronatus | Deep temporalis | Tortuosity | Minimum Continuation Quality +50% | 58 | 39 | 1.04 | 1.02 | 2.05 | 93.1 | 0.043 |
| Eulemur coronatus | Deep temporalis | Tortuosity | Minimum Continuation Quality -50% | 58 | 62 | 1.04 | 1.05 | -1.21 | 109.2 | 0.231 |
| Eulemur coronatus | Deep temporalis | Tortuosity | Direction Coefficient 0.3 | 58 | 57 | 1.04 | 1.12 | -1.35 | 57.7 | 0.183 |
| Eulemur coronatus | Deep temporalis | Tortuosity | Direction Coefficient 0.1 | 58 | 50 | 1.04 | 1.01 | 3.48 | 71.2 | 0.001 |
| Eulemur coronatus | Deep temporalis | Tortuosity | Minimum Length 100% Cylinder Length | 58 | 37 | 1.04 | 1.05 | -0.88 | 67.6 | 0.385 |
| Eulemur coronatus | Deep temporalis | Tortuosity | Minimum Length 50% Cylinder Length | 58 | 74 | 1.04 | 1.03 | 1.03 | 115.1 | 0.305 |
| Eulemur coronatus | Lateral pterygoid | Tortuosity | Minimum Seed Correlation +50% | 33 | 15 | 1.02 | 1.02 | 0.18 | 34.1 | 0.857 |
| Eulemur coronatus | Lateral pterygoid | Tortuosity | Minimum Seed Correlation -50% | 33 | 36 | 1.02 | 1.02 | 0.04 | 65.8 | 0.972 |
| Eulemur coronatus | Lateral pterygoid | Tortuosity | Minimum Continuation Quality +50% | 33 | 14 | 1.02 | 1.01 | 3.30 | 38.3 | 0.002 |
| Eulemur coronatus | Lateral pterygoid | Tortuosity | Minimum Continuation Quality -50% | 33 | 57 | 1.02 | 1.04 | -2.84 | 83.8 | 0.006 |
| Eulemur coronatus | Lateral pterygoid | Tortuosity | Direction Coefficient 0.2 | 33 | 36 | 1.02 | 1.01 | 2.64 | 44 | 0.011 |
| Eulemur coronatus | Lateral pterygoid | Tortuosity | Direction Coefficient 0.1 | 33 | 20 | 1.02 | 1.00 | 3.72 | 36.8 | 0.001 |
| Eulemur coronatus | Lateral pterygoid | Tortuosity | Minimum Length 100% Cylinder Length | 33 | 18 | 1.02 | 1.03 | -1.62 | 31.7 | 0.115 |
| Eulemur coronatus | Lateral pterygoid | Tortuosity | Minimum Length 50% Cylinder Length | 33 | 51 | 1.02 | 1.02 | 0.95 | 61.3 | 0.348 |
| Eulemur coronatus | Medial pterygoid | Tortuosity | Minimum Seed Correlation +50% | 46 | 9 | 1.01 | 1.02 | -0.57 | 9.7 | 0.580 |
| Eulemur coronatus | Medial pterygoid | Tortuosity | Minimum Seed Correlation -50% | 46 | 46 | 1.01 | 1.01 | 0.00 | 90 | 1.000 |
| Eulemur coronatus | Medial pterygoid | Tortuosity | Minimum Continuation Quality +50% | 46 | 8 | 1.01 | 1.00 | 3.71 | 41.9 | 0.001 |
| Eulemur coronatus | Medial pterygoid | Tortuosity | Minimum Continuation Quality -50% | 46 | 116 | 1.01 | 1.05 | -7.10 | 139.8 | 0.000 |
| Eulemur coronatus | Medial pterygoid | Tortuosity | Direction Coefficient 0.2 | 46 | 38 | 1.01 | 1.01 | 2.82 | 71.7 | 0.006 |
| Eulemur coronatus | Medial pterygoid | Tortuosity | Direction Coefficient 0.1 | 46 | 16 | 1.01 | 1.00 | 5.24 | 56.3 | 0.000 |
| Eulemur coronatus | Medial pterygoid | Tortuosity | Minimum Length 100% Cylinder Length | 46 | 24 | 1.01 | 1.02 | -1.47 | 39.8 | 0.150 |
| Eulemur coronatus | Medial pterygoid | Tortuosity | Minimum Length 50% Cylinder Length | 46 | 85 | 1.01 | 1.01 | 1.35 | 81.6 | 0.180 |
| Eulemur coronatus | Posterior digastric | Tortuosity | Minimum Seed Correlation +50% | 30 | 14 | 1.01 | 1.01 | -0.17 | 25.7 | 0.865 |
| Eulemur coronatus | Posterior digastric | Tortuosity | Minimum Seed Correlation -50% | 30 | 35 | 1.01 | 1.01 | 0.14 | 61.5 | 0.893 |
| Eulemur coronatus | Posterior digastric | Tortuosity | Minimum Continuation Quality +50% | 30 | 24 | 1.01 | 1.01 | 2.05 | 51.7 | 0.046 |
| Eulemur coronatus | Posterior digastric | Tortuosity | Minimum Continuation Quality -50% | 30 | 38 | 1.01 | 1.02 | -1.55 | 65.8 | 0.127 |
| Eulemur coronatus | Posterior digastric | Tortuosity | Direction Coefficient 0.2 | 30 | 33 | 1.01 | 1.01 | 1.35 | 60 | 0.181 |
| Eulemur coronatus | Posterior digastric | Tortuosity | Direction Coefficient 0.1 | 30 | 26 | 1.01 | 1.00 | 4.41 | 43.4 | 0.000 |
| Eulemur coronatus | Posterior digastric | Tortuosity | Minimum Length 100% Cylinder Length | 30 | 24 | 1.01 | 1.02 | -0.95 | 47 | 0.348 |
| Eulemur coronatus | Posterior digastric | Tortuosity | Minimum Length 50% Cylinder Length | 30 | 43 | 1.01 | 1.01 | 0.94 | 61.3 | 0.351 |
| Eulemur coronatus | Superficial masseter | Tortuosity | Minimum Seed Correlation +50% | 62 | 12 | 1.02 | 1.01 | 1.69 | 41.9 | 0.099 |
| Eulemur coronatus | Superficial masseter | Tortuosity | Minimum Seed Correlation -50% | 62 | 66 | 1.02 | 1.02 | 0.23 | 125.2 | 0.816 |
| Eulemur coronatus | Superficial masseter | Tortuosity | Minimum Continuation Quality +50% | 62 | 4 | 1.02 | 1.00 | 5.79 | 59.2 | 0.000 |
| Eulemur coronatus | Superficial masseter | Tortuosity | Minimum Continuation Quality -50% | 62 | 107 | 1.02 | 1.06 | -4.63 | 131.7 | 0.000 |
| Eulemur coronatus | Superficial masseter | Tortuosity | Direction Coefficient 0.2 | 62 | 55 | 1.02 | 1.01 | 2.85 | 94.7 | 0.005 |
| Eulemur coronatus | Superficial masseter | Tortuosity | Direction Coefficient 0.1 | 62 | 12 | 1.02 | 1.00 | 5.92 | 64.6 | 0.000 |
| Eulemur coronatus | Superficial masseter | Tortuosity | Minimum Length 100% Cylinder Length | 62 | 30 | 1.02 | 1.03 | -1.23 | 48.8 | 0.223 |
| Eulemur coronatus | Superficial masseter | Tortuosity | Minimum Length 50% Cylinder Length | 62 | 96 | 1.02 | 1.02 | 1.24 | 115.9 | 0.217 |
| Eulemur coronatus | Superficial temporalis | Tortuosity | Minimum Seed Correlation +50% | 30 | 4 | 1.03 | 1.04 | -0.56 | 3.5 | 0.612 |
| Eulemur coronatus | Superficial temporalis | Tortuosity | Minimum Seed Correlation -50% | 30 | 36 | 1.03 | 1.03 | 0.24 | 59.9 | 0.810 |
| Eulemur coronatus | Superficial temporalis | Tortuosity | Minimum Continuation Quality +50% | 30 | 9 | 1.03 | 1.01 | 2.13 | 32 | 0.041 |
| Eulemur coronatus | Superficial temporalis | Tortuosity | Minimum Continuation Quality -50% | 30 | 54 | 1.03 | 1.06 | -3.40 | 81.7 | 0.001 |
| Eulemur coronatus | Superficial temporalis | Tortuosity | Direction Coefficient 0.3 | 30 | 41 | 1.03 | 1.05 | -2.18 | 68.9 | 0.033 |
| Eulemur coronatus | Superficial temporalis | Tortuosity | Direction Coefficient 0.1 | 30 | 15 | 1.03 | 1.01 | 3.02 | 34 | 0.005 |
| Eulemur coronatus | Superficial temporalis | Tortuosity | Minimum Length 100% Cylinder Length | 30 | 13 | 1.03 | 1.04 | -0.93 | 28.4 | 0.359 |
| Eulemur coronatus | Superficial temporalis | Tortuosity | Minimum Length 50% Cylinder Length | 30 | 51 | 1.03 | 1.02 | 0.77 | 52 | 0.445 |
| Eulemur coronatus | Zygomaticomandibularis | Tortuosity | Minimum Seed Correlation +50% | 44 | 10 | 1.02 | 1.03 | -0.91 | 14 | 0.381 |
| Eulemur coronatus | Zygomaticomandibularis | Tortuosity | Minimum Seed Correlation -50% | 44 | 18 | 1.02 | 1.02 | 0.42 | 28.5 | 0.681 |
| Eulemur coronatus | Zygomaticomandibularis | Tortuosity | Minimum Continuation Quality +50% | 44 | 3 | 1.02 | 1.01 | 3.19 | 3.8 | 0.036 |
| Eulemur coronatus | Zygomaticomandibularis | Tortuosity | Minimum Continuation Quality -50% | 44 | 45 | 1.02 | 1.10 | -4.74 | 46.1 | 0.000 |
| Eulemur coronatus | Zygomaticomandibularis | Tortuosity | Direction Coefficient 0.2 | 44 | 11 | 1.02 | 1.01 | 1.77 | 17.6 | 0.093 |
| Eulemur coronatus | Zygomaticomandibularis | Tortuosity | Direction Coefficient 0.1 | 44 | 3 | 1.02 | 1.00 | 8.42 | 44 | 0.000 |
| Eulemur coronatus | Zygomaticomandibularis | Tortuosity | Minimum Length 100% Cylinder Length | 44 | 3 | 1.02 | 1.02 | 0.54 | 2.2 | 0.640 |
| Eulemur coronatus | Zygomaticomandibularis | Tortuosity | Minimum Length 50% Cylinder Length | 44 | 3 | 1.02 | 1.02 | 0.54 | 2.2 | 0.640 |
| Eulemur coronatus | Zygomatic temporalis | Tortuosity | Minimum Seed Correlation +50% | 41 | 15 | 1.04 | 1.06 | -1.70 | 26.3 | 0.101 |
| Eulemur coronatus | Zygomatic temporalis | Tortuosity | Minimum Seed Correlation -50% | 41 | 34 | 1.04 | 1.04 | -0.19 | 68.6 | 0.849 |
| Eulemur coronatus | Zygomatic temporalis | Tortuosity | Minimum Continuation Quality +50% | 41 | 24 | 1.04 | 1.02 | 3.27 | 59.9 | 0.002 |
| Eulemur coronatus | Zygomatic temporalis | Tortuosity | Minimum Continuation Quality -50% | 41 | 48 | 1.04 | 1.07 | -2.47 | 81.4 | 0.016 |
| Eulemur coronatus | Zygomatic temporalis | Tortuosity | Direction Coefficient 0.2 | 41 | 30 | 1.04 | 1.02 | 2.87 | 57.9 | 0.006 |
| Eulemur coronatus | Zygomatic temporalis | Tortuosity | Direction Coefficient 0.1 | 41 | 18 | 1.04 | 1.01 | 5.64 | 42.6 | 0.000 |
| Eulemur coronatus | Zygomatic temporalis | Tortuosity | Minimum Length 100% Cylinder Length | 41 | 22 | 1.04 | 1.06 | -1.39 | 38 | 0.173 |
| Eulemur coronatus | Zygomatic temporalis | Tortuosity | Minimum Length 50% Cylinder Length | 41 | 51 | 1.04 | 1.03 | 0.84 | 83.5 | 0.405 |
| Otolemur crassicaudatus | Anterior digastric | Tortuosity | Minimum Seed Correlation +50% | 34 | 3 | 1.02 | 1.02 | -1.53 | 31.8 | 0.136 |
| Otolemur crassicaudatus | Anterior digastric | Tortuosity | Minimum Seed Correlation -50% | 34 | 52 | 1.02 | 1.01 | 0.66 | 65.7 | 0.513 |
| Otolemur crassicaudatus | Anterior digastric | Tortuosity | Minimum Continuation Quality +50% | 34 | 23 | 1.02 | 1.01 | 2.62 | 50.7 | 0.012 |
| Otolemur crassicaudatus | Anterior digastric | Tortuosity | Minimum Continuation Quality -50% | 34 | 40 | 1.02 | 1.02 | -1.19 | 69 | 0.236 |
| Otolemur crassicaudatus | Anterior digastric | Tortuosity | Direction Coefficient 0.2 | 34 | 33 | 1.02 | 1.01 | 1.82 | 53.2 | 0.074 |
| Otolemur crassicaudatus | Anterior digastric | Tortuosity | Direction Coefficient 0.1 | 34 | 32 | 1.02 | 1.00 | 3.99 | 40 | 0.000 |
| Otolemur crassicaudatus | Anterior digastric | Tortuosity | Minimum Length 100% Cylinder Length | 34 | 27 | 1.02 | 1.02 | -0.28 | 58.3 | 0.777 |
| Otolemur crassicaudatus | Anterior digastric | Tortuosity | Minimum Length 50% Cylinder Length | 34 | 36 | 1.02 | 1.02 | 0.10 | 67.6 | 0.918 |
| Otolemur crassicaudatus | Deep masseter | Tortuosity | Minimum Seed Correlation +50% | 43 | 37 | 1.01 | 1.01 | -0.09 | 74.8 | 0.928 |
| Otolemur crassicaudatus | Deep masseter | Tortuosity | Minimum Seed Correlation -50% | 43 | 43 | 1.01 | 1.01 | 0.00 | 84 | 1.000 |
| Otolemur crassicaudatus | Deep masseter | Tortuosity | Minimum Continuation Quality -50% | 43 | 355 | 1.01 | 1.03 | -8.76 | 379.5 | 0.000 |
| Otolemur crassicaudatus | Deep masseter | Tortuosity | Direction Coefficient 0.2 | 43 | 29 | 1.01 | 1.00 | 3.48 | 61.9 | 0.001 |
| Otolemur crassicaudatus | Deep masseter | Tortuosity | Direction Coefficient 0.1 | 43 | 10 | 1.01 | 1.00 | 4.49 | 47 | 0.000 |
| Otolemur crassicaudatus | Deep masseter | Tortuosity | Minimum Length 100% Cylinder Length | 43 | 19 | 1.01 | 1.01 | -1.28 | 28.2 | 0.212 |
| Otolemur crassicaudatus | Deep masseter | Tortuosity | Minimum Length 50% Cylinder Length | 43 | 85 | 1.01 | 1.01 | 1.96 | 70.7 | 0.053 |
| Otolemur crassicaudatus | Deep temporalis | Tortuosity | Minimum Seed Correlation +50% | 74 | 17 | 1.01 | 1.03 | -1.87 | 18.5 | 0.078 |
| Otolemur crassicaudatus | Deep temporalis | Tortuosity | Minimum Seed Correlation -50% | 74 | 77 | 1.01 | 1.01 | 0.05 | 148.5 | 0.959 |
| Otolemur crassicaudatus | Deep temporalis | Tortuosity | Minimum Continuation Quality +50% | 74 | 33 | 1.01 | 1.00 | 4.06 | 77.5 | 0.000 |
| Otolemur crassicaudatus | Deep temporalis | Tortuosity | Minimum Continuation Quality -50% | 74 | 82 | 1.01 | 1.04 | -1.94 | 90 | 0.056 |
| Otolemur crassicaudatus | Deep temporalis | Tortuosity | Direction Coefficient 0.3 | 74 | 78 | 1.01 | 1.03 | -2.41 | 109.7 | 0.018 |
| Otolemur crassicaudatus | Deep temporalis | Tortuosity | Direction Coefficient 0.1 | 74 | 65 | 1.01 | 1.00 | 4.24 | 75.6 | 0.000 |
| Otolemur crassicaudatus | Deep temporalis | Tortuosity | Minimum Length 100% Cylinder Length | 74 | 50 | 1.01 | 1.04 | -3.09 | 59.2 | 0.003 |
| Otolemur crassicaudatus | Deep temporalis | Tortuosity | Minimum Length 50% Cylinder Length | 74 | 101 | 1.01 | 1.02 | -2.09 | 156.5 | 0.038 |
| Otolemur crassicaudatus | Lateral pterygoid | Tortuosity | Minimum Seed Correlation +50% | 40 | 4 | 1.02 | 1.03 | -0.40 | 3.5 | 0.710 |
| Otolemur crassicaudatus | Lateral pterygoid | Tortuosity | Minimum Seed Correlation -50% | 40 | 52 | 1.02 | 1.02 | 0.18 | 82 | 0.856 |
| Otolemur crassicaudatus | Lateral pterygoid | Tortuosity | Minimum Continuation Quality +50% | 40 | 18 | 1.02 | 1.01 | 2.93 | 55.3 | 0.005 |
| Otolemur crassicaudatus | Lateral pterygoid | Tortuosity | Minimum Continuation Quality -50% | 40 | 61 | 1.02 | 1.05 | -3.28 | 93.2 | 0.001 |
| Otolemur crassicaudatus | Lateral pterygoid | Tortuosity | Direction Coefficient 0.2 | 40 | 32 | 1.02 | 1.01 | 2.22 | 64.8 | 0.030 |
| Otolemur crassicaudatus | Lateral pterygoid | Tortuosity | Direction Coefficient 0.1 | 40 | 13 | 1.02 | 1.01 | 4.07 | 50.9 | 0.000 |
| Otolemur crassicaudatus | Lateral pterygoid | Tortuosity | Minimum Length 100% Cylinder Length | 40 | 22 | 1.02 | 1.03 | -1.02 | 38.4 | 0.314 |
| Otolemur crassicaudatus | Lateral pterygoid | Tortuosity | Minimum Length 50% Cylinder Length | 40 | 53 | 1.02 | 1.02 | 0.92 | 80.7 | 0.363 |
| Otolemur crassicaudatus | Medial pterygoid | Tortuosity | Minimum Seed Correlation -50% | 45 | 51 | 1.01 | 1.01 | 0.49 | 92 | 0.625 |
| Otolemur crassicaudatus | Medial pterygoid | Tortuosity | Minimum Continuation Quality +50% | 45 | 3 | 1.01 | 1.01 | 3.35 | 8.2 | 0.010 |
| Otolemur crassicaudatus | Medial pterygoid | Tortuosity | Minimum Continuation Quality -50% | 45 | 75 | 1.01 | 1.05 | -5.53 | 82.9 | 0.000 |
| Otolemur crassicaudatus | Medial pterygoid | Tortuosity | Direction Coefficient 0.2 | 45 | 27 | 1.01 | 1.01 | 2.18 | 67.6 | 0.033 |
| Otolemur crassicaudatus | Medial pterygoid | Tortuosity | Direction Coefficient 0.1 | 45 | 17 | 1.01 | 1.00 | 6.47 | 49.4 | 0.000 |
| Otolemur crassicaudatus | Medial pterygoid | Tortuosity | Minimum Length 100% Cylinder Length | 45 | 20 | 1.01 | 1.02 | -1.95 | 28.7 | 0.061 |
| Otolemur crassicaudatus | Medial pterygoid | Tortuosity | Minimum Length 50% Cylinder Length | 45 | 67 | 1.01 | 1.01 | 1.52 | 88.9 | 0.132 |
| Otolemur crassicaudatus | Posterior digastric | Tortuosity | Minimum Seed Correlation +50% | 37 | 6 | 1.02 | 1.03 | -0.95 | 6.6 | 0.374 |
| Otolemur crassicaudatus | Posterior digastric | Tortuosity | Minimum Seed Correlation -50% | 37 | 39 | 1.02 | 1.02 | 0.06 | 73.7 | 0.951 |
| Otolemur crassicaudatus | Posterior digastric | Tortuosity | Minimum Continuation Quality +50% | 37 | 21 | 1.02 | 1.00 | 5.50 | 38.4 | 0.000 |
| Otolemur crassicaudatus | Posterior digastric | Tortuosity | Minimum Continuation Quality -50% | 37 | 42 | 1.02 | 1.02 | -0.28 | 76.9 | 0.778 |
| Otolemur crassicaudatus | Posterior digastric | Tortuosity | Direction Coefficient 0.2 | 37 | 41 | 1.02 | 1.01 | 2.60 | 60.3 | 0.012 |
| Otolemur crassicaudatus | Posterior digastric | Tortuosity | Direction Coefficient 0.1 | 37 | 40 | 1.02 | 1.00 | 6.35 | 36.5 | 0.000 |
| Otolemur crassicaudatus | Posterior digastric | Tortuosity | Minimum Length 100% Cylinder Length | 37 | 30 | 1.02 | 1.02 | -0.57 | 61.4 | 0.570 |
| Otolemur crassicaudatus | Posterior digastric | Tortuosity | Minimum Length 50% Cylinder Length | 37 | 49 | 1.02 | 1.02 | 1.07 | 76.7 | 0.289 |
| Otolemur crassicaudatus | Superficial masseter | Tortuosity | Minimum Seed Correlation +50% | 56 | 3 | 1.01 | 1.00 | 1.47 | 3.7 | 0.222 |
| Otolemur crassicaudatus | Superficial masseter | Tortuosity | Minimum Seed Correlation -50% | 56 | 58 | 1.01 | 1.01 | -0.22 | 112 | 0.827 |
| Otolemur crassicaudatus | Superficial masseter | Tortuosity | Minimum Continuation Quality +50% | 56 | 7 | 1.01 | 1.00 | 1.79 | 9.5 | 0.105 |
| Otolemur crassicaudatus | Superficial masseter | Tortuosity | Minimum Continuation Quality -50% | 56 | 126 | 1.01 | 1.02 | -6.88 | 151 | 0.000 |
| Otolemur crassicaudatus | Superficial masseter | Tortuosity | Direction Coefficient 0.3 | 56 | 74 | 1.01 | 1.01 | -2.16 | 93.9 | 0.033 |
| Otolemur crassicaudatus | Superficial masseter | Tortuosity | Direction Coefficient 0.1 | 56 | 39 | 1.01 | 1.00 | 4.44 | 72.7 | 0.000 |
| Otolemur crassicaudatus | Superficial masseter | Tortuosity | Minimum Length 100% Cylinder Length | 56 | 26 | 1.01 | 1.01 | -0.55 | 40.7 | 0.584 |
| Otolemur crassicaudatus | Superficial masseter | Tortuosity | Minimum Length 50% Cylinder Length | 56 | 98 | 1.01 | 1.00 | 1.64 | 99.8 | 0.103 |
| Otolemur crassicaudatus | Zygomatic temporalis | Tortuosity | Minimum Seed Correlation +50% | 43 | 3 | 1.02 | 1.01 | 1.93 | 5.1 | 0.110 |
| Otolemur crassicaudatus | Zygomatic temporalis | Tortuosity | Minimum Seed Correlation -50% | 43 | 59 | 1.02 | 1.02 | 0.55 | 85.5 | 0.585 |
| Otolemur crassicaudatus | Zygomatic temporalis | Tortuosity | Minimum Continuation Quality +50% | 43 | 22 | 1.02 | 1.01 | 4.37 | 47.3 | 0.000 |
| Otolemur crassicaudatus | Zygomatic temporalis | Tortuosity | Minimum Continuation Quality -50% | 43 | 38 | 1.02 | 1.08 | -4.72 | 43.3 | 0.000 |
| Otolemur crassicaudatus | Zygomatic temporalis | Tortuosity | Direction Coefficient 0.2 | 43 | 37 | 1.02 | 1.01 | 2.76 | 64.2 | 0.007 |
| Otolemur crassicaudatus | Zygomatic temporalis | Tortuosity | Direction Coefficient 0.1 | 43 | 28 | 1.02 | 1.00 | 5.02 | 45.1 | 0.000 |
| Otolemur crassicaudatus | Zygomatic temporalis | Tortuosity | Minimum Length 100% Cylinder Length | 43 | 35 | 1.02 | 1.03 | -1.34 | 65.4 | 0.184 |
| Otolemur crassicaudatus | Zygomatic temporalis | Tortuosity | Minimum Length 50% Cylinder Length | 43 | 46 | 1.02 | 1.02 | 0.83 | 82.6 | 0.409 |
| Lontra canadensis | Digastric | Tortuosity | Minimum Seed Correlation +50% | 56 | 3 | 1.01 | 1.01 | 2.70 | 6.7 | 0.032 |
| Lontra canadensis | Digastric | Tortuosity | Minimum Seed Correlation -50% | 56 | 81 | 1.01 | 1.01 | -0.06 | 120.8 | 0.949 |
| Lontra canadensis | Digastric | Tortuosity | Minimum Continuation Quality +50% | 56 | 46 | 1.01 | 1.00 | 5.66 | 73.1 | 0.000 |
| Lontra canadensis | Digastric | Tortuosity | Minimum Continuation Quality -50% | 56 | 61 | 1.01 | 1.02 | -3.99 | 80.8 | 0.000 |
| Lontra canadensis | Digastric | Tortuosity | Direction Coefficient 0.2 | 56 | 56 | 1.01 | 1.01 | 2.07 | 105.1 | 0.041 |
| Lontra canadensis | Digastric | Tortuosity | Direction Coefficient 0.1 | 56 | 55 | 1.01 | 1.00 | 7.69 | 60.3 | 0.000 |
| Lontra canadensis | Digastric | Tortuosity | Minimum Length 100% Cylinder Length | 56 | 45 | 1.01 | 1.01 | -0.93 | 94.7 | 0.355 |
| Lontra canadensis | Digastric | Tortuosity | Minimum Length 50% Cylinder Length | 56 | 59 | 1.01 | 1.01 | 0.06 | 112.9 | 0.953 |
| Lontra canadensis | Deep masseter | Tortuosity | Minimum Seed Correlation +50% | 60 | 8 | 1.02 | 1.01 | 1.55 | 45.4 | 0.127 |
| Lontra canadensis | Deep masseter | Tortuosity | Minimum Seed Correlation -50% | 60 | 62 | 1.02 | 1.02 | 0.03 | 119.7 | 0.979 |
| Lontra canadensis | Deep masseter | Tortuosity | Minimum Continuation Quality +50% | 60 | 13 | 1.02 | 1.00 | 4.31 | 68.3 | 0.000 |
| Lontra canadensis | Deep masseter | Tortuosity | Minimum Continuation Quality -50% | 60 | 91 | 1.02 | 1.08 | -4.92 | 99.6 | 0.000 |
| Lontra canadensis | Deep masseter | Tortuosity | Direction Coefficient 0.2 | 60 | 52 | 1.02 | 1.01 | 3.08 | 70.6 | 0.003 |
| Lontra canadensis | Deep masseter | Tortuosity | Direction Coefficient 0.1 | 60 | 32 | 1.02 | 1.00 | 5.17 | 60.5 | 0.000 |
| Lontra canadensis | Deep masseter | Tortuosity | Minimum Length 100% Cylinder Length | 60 | 30 | 1.02 | 1.02 | -0.51 | 45.8 | 0.612 |
| Lontra canadensis | Deep masseter | Tortuosity | Minimum Length 50% Cylinder Length | 60 | 97 | 1.02 | 1.01 | 1.19 | 108.7 | 0.239 |
| Lontra canadensis | Deep temporalis | Tortuosity | Minimum Seed Correlation +50% | 66 | 13 | 1.01 | 1.02 | -0.59 | 13.1 | 0.564 |
| Lontra canadensis | Deep temporalis | Tortuosity | Minimum Seed Correlation -50% | 66 | 70 | 1.01 | 1.01 | -0.06 | 133 | 0.949 |
| Lontra canadensis | Deep temporalis | Tortuosity | Minimum Continuation Quality +50% | 66 | 29 | 1.01 | 1.00 | 3.13 | 73 | 0.003 |
| Lontra canadensis | Deep temporalis | Tortuosity | Minimum Continuation Quality -50% | 66 | 79 | 1.01 | 1.05 | -3.96 | 84.9 | 0.000 |
| Lontra canadensis | Deep temporalis | Tortuosity | Direction Coefficient 0.3 | 66 | 79 | 1.01 | 1.02 | -2.56 | 140.5 | 0.011 |
| Lontra canadensis | Deep temporalis | Tortuosity | Direction Coefficient 0.1 | 66 | 57 | 1.01 | 1.00 | 3.70 | 71.3 | 0.000 |
| Lontra canadensis | Deep temporalis | Tortuosity | Minimum Length 100% Cylinder Length | 66 | 39 | 1.01 | 1.01 | -0.93 | 68 | 0.356 |
| Lontra canadensis | Deep temporalis | Tortuosity | Minimum Length 50% Cylinder Length | 66 | 96 | 1.01 | 1.01 | 0.75 | 124.9 | 0.456 |
| Lontra canadensis | Lateral pterygoid | Tortuosity | Minimum Seed Correlation +50% | 36 | 3 | 1.02 | 1.05 | -0.80 | 2.1 | 0.505 |
| Lontra canadensis | Lateral pterygoid | Tortuosity | Minimum Seed Correlation -50% | 36 | 51 | 1.02 | 1.03 | -0.13 | 74.2 | 0.899 |
| Lontra canadensis | Lateral pterygoid | Tortuosity | Minimum Continuation Quality +50% | 36 | 6 | 1.02 | 1.01 | 3.17 | 38.5 | 0.003 |
| Lontra canadensis | Lateral pterygoid | Tortuosity | Minimum Continuation Quality -50% | 36 | 81 | 1.02 | 1.08 | -5.30 | 112.9 | 0.000 |
| Lontra canadensis | Lateral pterygoid | Tortuosity | Direction Coefficient 0.2 | 36 | 22 | 1.02 | 1.01 | 3.25 | 43 | 0.002 |
| Lontra canadensis | Lateral pterygoid | Tortuosity | Direction Coefficient 0.1 | 36 | 10 | 1.02 | 1.00 | 4.50 | 35.4 | 0.000 |
| Lontra canadensis | Lateral pterygoid | Tortuosity | Minimum Length 100% Cylinder Length | 36 | 9 | 1.02 | 1.05 | -1.91 | 10.3 | 0.085 |
| Lontra canadensis | Lateral pterygoid | Tortuosity | Minimum Length 50% Cylinder Length | 36 | 81 | 1.02 | 1.01 | 1.82 | 52.5 | 0.075 |
| Lontra canadensis | Medial pterygoid | Tortuosity | Minimum Seed Correlation +50% | 44 | 8 | 1.02 | 1.05 | -1.47 | 7.9 | 0.180 |
| Lontra canadensis | Medial pterygoid | Tortuosity | Minimum Seed Correlation -50% | 44 | 45 | 1.02 | 1.02 | 0.05 | 86.9 | 0.958 |
| Lontra canadensis | Medial pterygoid | Tortuosity | Minimum Continuation Quality +50% | 44 | 9 | 1.02 | 1.01 | 2.92 | 47.1 | 0.005 |
| Lontra canadensis | Medial pterygoid | Tortuosity | Minimum Continuation Quality -50% | 44 | 77 | 1.02 | 1.06 | -3.72 | 110.7 | 0.000 |
| Lontra canadensis | Medial pterygoid | Tortuosity | Direction Coefficient 0.3 | 44 | 55 | 1.02 | 1.05 | -3.70 | 94.6 | 0.000 |
| Lontra canadensis | Medial pterygoid | Tortuosity | Direction Coefficient 0.1 | 44 | 24 | 1.02 | 1.00 | 4.17 | 46.5 | 0.000 |
| Lontra canadensis | Medial pterygoid | Tortuosity | Minimum Length 100% Cylinder Length | 44 | 18 | 1.02 | 1.04 | -1.48 | 24.7 | 0.151 |
| Lontra canadensis | Medial pterygoid | Tortuosity | Minimum Length 50% Cylinder Length | 44 | 76 | 1.02 | 1.02 | 1.22 | 77 | 0.226 |
| Lontra canadensis | Superficial masseter | Tortuosity | Minimum Seed Correlation +50% | 64 | 7 | 1.02 | 1.03 | -0.36 | 7.3 | 0.729 |
| Lontra canadensis | Superficial masseter | Tortuosity | Minimum Seed Correlation -50% | 64 | 69 | 1.02 | 1.02 | 0.24 | 129.7 | 0.809 |
| Lontra canadensis | Superficial masseter | Tortuosity | Minimum Continuation Quality +50% | 64 | 29 | 1.02 | 1.01 | 4.73 | 79.5 | 0.000 |
| Lontra canadensis | Superficial masseter | Tortuosity | Minimum Continuation Quality -50% | 64 | 97 | 1.02 | 1.06 | -4.48 | 123.8 | 0.000 |
| Lontra canadensis | Superficial masseter | Tortuosity | Direction Coefficient 0.2 | 64 | 54 | 1.02 | 1.01 | 4.36 | 89 | 0.000 |
| Lontra canadensis | Superficial masseter | Tortuosity | Direction Coefficient 0.1 | 64 | 39 | 1.02 | 1.00 | 6.45 | 67.5 | 0.000 |
| Lontra canadensis | Superficial masseter | Tortuosity | Minimum Length 100% Cylinder Length | 64 | 33 | 1.02 | 1.03 | -1.17 | 56.8 | 0.248 |
| Lontra canadensis | Superficial masseter | Tortuosity | Minimum Length 50% Cylinder Length | 64 | 105 | 1.02 | 1.02 | 1.79 | 116.9 | 0.076 |
| Lontra canadensis | Superficial temporalis | Tortuosity | Minimum Seed Correlation +50% | 72 | 33 | 1.03 | 1.03 | -0.64 | 54.2 | 0.523 |
| Lontra canadensis | Superficial temporalis | Tortuosity | Minimum Seed Correlation -50% | 72 | 72 | 1.03 | 1.03 | 0.00 | 142 | 1.000 |
| Lontra canadensis | Superficial temporalis | Tortuosity | Minimum Continuation Quality +50% | 72 | 19 | 1.03 | 1.01 | 3.78 | 69 | 0.000 |
| Lontra canadensis | Superficial temporalis | Tortuosity | Minimum Continuation Quality -50% | 72 | 102 | 1.03 | 1.05 | -4.14 | 141.4 | 0.000 |
| Lontra canadensis | Superficial temporalis | Tortuosity | Direction Coefficient 0.2 | 72 | 69 | 1.03 | 1.01 | 3.39 | 119.7 | 0.001 |
| Lontra canadensis | Superficial temporalis | Tortuosity | Direction Coefficient 0.1 | 72 | 30 | 1.03 | 1.00 | 7.52 | 74.8 | 0.000 |
| Lontra canadensis | Superficial temporalis | Tortuosity | Minimum Length 100% Cylinder Length | 72 | 36 | 1.03 | 1.03 | -1.60 | 63.2 | 0.116 |
| Lontra canadensis | Superficial temporalis | Tortuosity | Minimum Length 50% Cylinder Length | 72 | 119 | 1.03 | 1.02 | 1.26 | 137.3 | 0.211 |
| Lontra canadensis | Zygomaticomandibularis | Tortuosity | Minimum Seed Correlation +50% | 52 | 18 | 1.02 | 1.01 | 1.35 | 45.3 | 0.183 |
| Lontra canadensis | Zygomaticomandibularis | Tortuosity | Minimum Seed Correlation -50% | 52 | 54 | 1.02 | 1.02 | -0.04 | 103.8 | 0.967 |
| Lontra canadensis | Zygomaticomandibularis | Tortuosity | Minimum Continuation Quality +50% | 52 | 25 | 1.02 | 1.01 | 4.49 | 68.1 | 0.000 |
| Lontra canadensis | Zygomaticomandibularis | Tortuosity | Minimum Continuation Quality -50% | 52 | 68 | 1.02 | 1.05 | -4.15 | 81.2 | 0.000 |
| Lontra canadensis | Zygomaticomandibularis | Tortuosity | Direction Coefficient 0.2 | 52 | 47 | 1.02 | 1.01 | 3.62 | 79.9 | 0.001 |
| Lontra canadensis | Zygomaticomandibularis | Tortuosity | Direction Coefficient 0.1 | 52 | 33 | 1.02 | 1.00 | 6.71 | 52.6 | 0.000 |
| Lontra canadensis | Zygomaticomandibularis | Tortuosity | Minimum Length 100% Cylinder Length | 52 | 30 | 1.02 | 1.03 | -1.27 | 51.7 | 0.210 |
| Lontra canadensis | Zygomaticomandibularis | Tortuosity | Minimum Length 50% Cylinder Length | 52 | 74 | 1.02 | 1.02 | 1.24 | 104 | 0.218 |
| Mustela nivalis | Digastric | Tortuosity | Minimum Seed Correlation +50% | 47 | 19 | 1.01 | 1.01 | -0.07 | 42.8 | 0.943 |
| Mustela nivalis | Digastric | Tortuosity | Minimum Seed Correlation -50% | 47 | 58 | 1.01 | 1.01 | 0.50 | 95.3 | 0.619 |
| Mustela nivalis | Digastric | Tortuosity | Minimum Continuation Quality +50% | 47 | 46 | 1.01 | 1.01 | 2.40 | 82.1 | 0.019 |
| Mustela nivalis | Digastric | Tortuosity | Minimum Continuation Quality -50% | 47 | 57 | 1.01 | 1.02 | -0.93 | 97 | 0.354 |
| Mustela nivalis | Digastric | Tortuosity | Direction Coefficient 0.2 | 47 | 52 | 1.01 | 1.01 | 2.17 | 73.6 | 0.033 |
| Mustela nivalis | Digastric | Tortuosity | Direction Coefficient 0.1 | 47 | 46 | 1.01 | 1.00 | 4.32 | 52.4 | 0.000 |
| Mustela nivalis | Digastric | Tortuosity | Minimum Length 100% Cylinder Length | 47 | 46 | 1.01 | 1.01 | 0.25 | 91 | 0.801 |
| Mustela nivalis | Digastric | Tortuosity | Minimum Length 50% Cylinder Length | 47 | 50 | 1.01 | 1.01 | 0.15 | 94.4 | 0.885 |
| Mustela nivalis | Deep masseter | Tortuosity | Minimum Seed Correlation +50% | 30 | 3 | 1.01 | 1.01 | -0.01 | 2.5 | 0.993 |
| Mustela nivalis | Deep masseter | Tortuosity | Minimum Seed Correlation -50% | 30 | 34 | 1.01 | 1.01 | 0.23 | 60.2 | 0.816 |
| Mustela nivalis | Deep masseter | Tortuosity | Minimum Continuation Quality +50% | 30 | 2 | 1.01 | 1.00 | 2.99 | 29.8 | 0.006 |
| Mustela nivalis | Deep masseter | Tortuosity | Minimum Continuation Quality -50% | 30 | 85 | 1.01 | 1.06 | -4.43 | 97.7 | 0.000 |
| Mustela nivalis | Deep masseter | Tortuosity | Direction Coefficient 0.2 | 30 | 24 | 1.01 | 1.01 | 1.40 | 47.8 | 0.167 |
| Mustela nivalis | Deep masseter | Tortuosity | Direction Coefficient 0.1 | 30 | 13 | 1.01 | 1.00 | 3.37 | 29.9 | 0.002 |
| Mustela nivalis | Deep masseter | Tortuosity | Minimum Length 100% Cylinder Length | 30 | 9 | 1.01 | 1.02 | -0.73 | 11.4 | 0.478 |
| Mustela nivalis | Deep masseter | Tortuosity | Minimum Length 50% Cylinder Length | 30 | 58 | 1.01 | 1.01 | 1.23 | 44.8 | 0.225 |
| Mustela nivalis | Deep temporalis | Tortuosity | Minimum Seed Correlation +50% | 62 | 3 | 1.01 | 1.01 | -0.70 | 2.1 | 0.553 |
| Mustela nivalis | Deep temporalis | Tortuosity | Minimum Seed Correlation -50% | 62 | 68 | 1.01 | 1.01 | 0.25 | 126.2 | 0.802 |
| Mustela nivalis | Deep temporalis | Tortuosity | Minimum Continuation Quality +50% | 62 | 4 | 1.01 | 1.00 | 1.73 | 5 | 0.144 |
| Mustela nivalis | Deep temporalis | Tortuosity | Minimum Continuation Quality -50% | 62 | 107 | 1.01 | 1.04 | -5.51 | 113.4 | 0.000 |
| Mustela nivalis | Deep temporalis | Tortuosity | Direction Coefficient 0.2 | 62 | 60 | 1.01 | 1.01 | 1.62 | 116.3 | 0.109 |
| Mustela nivalis | Deep temporalis | Tortuosity | Direction Coefficient 0.1 | 62 | 34 | 1.01 | 1.00 | 4.62 | 86.2 | 0.000 |
| Mustela nivalis | Deep temporalis | Tortuosity | Minimum Length 100% Cylinder Length | 62 | 33 | 1.01 | 1.01 | -1.78 | 57.8 | 0.080 |
| Mustela nivalis | Deep temporalis | Tortuosity | Minimum Length 50% Cylinder Length | 62 | 78 | 1.01 | 1.01 | 1.07 | 121.5 | 0.285 |
| Mustela nivalis | Lateral pterygoid | Tortuosity | Minimum Seed Correlation +50% | 41 | 20 | 1.01 | 1.01 | -0.66 | 38.7 | 0.514 |
| Mustela nivalis | Lateral pterygoid | Tortuosity | Minimum Seed Correlation -50% | 41 | 43 | 1.01 | 1.01 | 0.15 | 81.7 | 0.878 |
| Mustela nivalis | Lateral pterygoid | Tortuosity | Minimum Continuation Quality +50% | 41 | 8 | 1.01 | 1.01 | 2.03 | 16.7 | 0.058 |
| Mustela nivalis | Lateral pterygoid | Tortuosity | Minimum Continuation Quality -50% | 41 | 69 | 1.01 | 1.02 | -2.34 | 106.8 | 0.021 |
| Mustela nivalis | Lateral pterygoid | Tortuosity | Direction Coefficient 0.2 | 41 | 36 | 1.01 | 1.01 | 1.09 | 74.7 | 0.278 |
| Mustela nivalis | Lateral pterygoid | Tortuosity | Direction Coefficient 0.1 | 41 | 19 | 1.01 | 1.00 | 5.09 | 41.5 | 0.000 |
| Mustela nivalis | Lateral pterygoid | Tortuosity | Minimum Length 100% Cylinder Length | 41 | 20 | 1.01 | 1.01 | -0.39 | 33.5 | 0.701 |
| Mustela nivalis | Lateral pterygoid | Tortuosity | Minimum Length 50% Cylinder Length | 41 | 54 | 1.01 | 1.01 | 0.86 | 81.5 | 0.394 |
| Mustela nivalis | Medial pterygoid | Tortuosity | Minimum Seed Correlation +50% | 45 | 25 | 1.01 | 1.01 | 0.03 | 45.3 | 0.978 |
| Mustela nivalis | Medial pterygoid | Tortuosity | Minimum Seed Correlation -50% | 45 | 48 | 1.01 | 1.01 | 0.24 | 90.4 | 0.811 |
| Mustela nivalis | Medial pterygoid | Tortuosity | Minimum Continuation Quality +50% | 45 | 4 | 1.01 | 1.00 | 5.84 | 45.9 | 0.000 |
| Mustela nivalis | Medial pterygoid | Tortuosity | Minimum Continuation Quality -50% | 45 | 113 | 1.01 | 1.03 | -6.86 | 152.8 | 0.000 |
| Mustela nivalis | Medial pterygoid | Tortuosity | Direction Coefficient 0.2 | 45 | 29 | 1.01 | 1.01 | 1.49 | 68.9 | 0.142 |
| Mustela nivalis | Medial pterygoid | Tortuosity | Direction Coefficient 0.1 | 45 | 16 | 1.01 | 1.00 | 5.20 | 48.8 | 0.000 |
| Mustela nivalis | Medial pterygoid | Tortuosity | Minimum Length 100% Cylinder Length | 45 | 24 | 1.01 | 1.01 | -0.87 | 40 | 0.391 |
| Mustela nivalis | Medial pterygoid | Tortuosity | Minimum Length 50% Cylinder Length | 45 | 59 | 1.01 | 1.01 | 1.04 | 91.9 | 0.303 |
| Mustela nivalis | Superficial masseter | Tortuosity | Minimum Seed Correlation -50% | 35 | 85 | 1.03 | 1.02 | 1.56 | 48.9 | 0.126 |
| Mustela nivalis | Superficial masseter | Tortuosity | Minimum Continuation Quality +50% | 35 | 15 | 1.03 | 1.00 | 3.85 | 34.8 | 0.000 |
| Mustela nivalis | Superficial masseter | Tortuosity | Minimum Continuation Quality -50% | 35 | 56 | 1.03 | 1.07 | -3.62 | 85.2 | 0.001 |
| Mustela nivalis | Superficial masseter | Tortuosity | Direction Coefficient 0.2 | 35 | 37 | 1.03 | 1.01 | 2.76 | 43.3 | 0.009 |
| Mustela nivalis | Superficial masseter | Tortuosity | Direction Coefficient 0.1 | 35 | 30 | 1.03 | 1.00 | 4.09 | 34.1 | 0.000 |
| Mustela nivalis | Superficial masseter | Tortuosity | Minimum Length 100% Cylinder Length | 35 | 26 | 1.03 | 1.03 | -0.21 | 55.5 | 0.837 |
| Mustela nivalis | Superficial masseter | Tortuosity | Minimum Length 50% Cylinder Length | 35 | 49 | 1.03 | 1.02 | 1.34 | 60.4 | 0.186 |
| Mustela nivalis | Superficial temporalis | Tortuosity | Minimum Seed Correlation +50% | 61 | 35 | 1.01 | 1.01 | -1.41 | 67.5 | 0.162 |
| Mustela nivalis | Superficial temporalis | Tortuosity | Minimum Seed Correlation -50% | 61 | 62 | 1.01 | 1.01 | 0.05 | 120.9 | 0.957 |
| Mustela nivalis | Superficial temporalis | Tortuosity | Minimum Continuation Quality +50% | 61 | 21 | 1.01 | 1.01 | 0.23 | 35.6 | 0.817 |
| Mustela nivalis | Superficial temporalis | Tortuosity | Minimum Continuation Quality -50% | 61 | 109 | 1.01 | 1.02 | -4.33 | 134.1 | 0.000 |
| Mustela nivalis | Superficial temporalis | Tortuosity | Direction Coefficient 0.3 | 61 | 74 | 1.01 | 1.01 | -3.04 | 132.9 | 0.003 |
| Mustela nivalis | Superficial temporalis | Tortuosity | Direction Coefficient 0.1 | 61 | 50 | 1.01 | 1.00 | 4.10 | 84.5 | 0.000 |
| Mustela nivalis | Superficial temporalis | Tortuosity | Minimum Length 100% Cylinder Length | 61 | 28 | 1.01 | 1.01 | -2.04 | 48.4 | 0.047 |
| Mustela nivalis | Superficial temporalis | Tortuosity | Minimum Length 50% Cylinder Length | 61 | 94 | 1.01 | 1.01 | 1.07 | 122.2 | 0.287 |
| Mustela nivalis | Zygomaticomandibularis | Tortuosity | Minimum Seed Correlation +50% | 46 | 11 | 1.03 | 1.05 | -1.93 | 14.1 | 0.074 |
| Mustela nivalis | Zygomaticomandibularis | Tortuosity | Minimum Seed Correlation -50% | 46 | 53 | 1.03 | 1.03 | 0.37 | 93.5 | 0.712 |
| Mustela nivalis | Zygomaticomandibularis | Tortuosity | Minimum Continuation Quality +50% | 46 | 5 | 1.03 | 1.02 | 1.20 | 6.5 | 0.272 |
| Mustela nivalis | Zygomaticomandibularis | Tortuosity | Minimum Continuation Quality -50% | 46 | 80 | 1.03 | 1.05 | -3.41 | 116.7 | 0.001 |
| Mustela nivalis | Zygomaticomandibularis | Tortuosity | Direction Coefficient 0.2 | 46 | 28 | 1.03 | 1.01 | 3.59 | 66.7 | 0.001 |
| Mustela nivalis | Zygomaticomandibularis | Tortuosity | Direction Coefficient 0.1 | 46 | 12 | 1.03 | 1.00 | 6.92 | 45.5 | 0.000 |
| Mustela nivalis | Zygomaticomandibularis | Tortuosity | Minimum Length 100% Cylinder Length | 46 | 24 | 1.03 | 1.04 | -2.05 | 48 | 0.046 |
| Mustela nivalis | Zygomaticomandibularis | Tortuosity | Minimum Length 50% Cylinder Length | 46 | 63 | 1.03 | 1.02 | 1.06 | 93.6 | 0.293 |
| Procyon lotor | Digastric | Tortuosity | Minimum Seed Correlation +50% | 55 | 12 | 1.00 | 1.00 | -0.08 | 18.1 | 0.941 |
| Procyon lotor | Digastric | Tortuosity | Minimum Seed Correlation -50% | 55 | 58 | 1.00 | 1.00 | 0.04 | 110.4 | 0.970 |
| Procyon lotor | Digastric | Tortuosity | Minimum Continuation Quality +50% | 55 | 25 | 1.00 | 1.00 | 3.92 | 71.5 | 0.000 |
| Procyon lotor | Digastric | Tortuosity | Minimum Continuation Quality -50% | 55 | 64 | 1.00 | 1.01 | -3.87 | 94.2 | 0.000 |
| Procyon lotor | Digastric | Tortuosity | Direction Coefficient 0.2 | 55 | 60 | 1.00 | 1.00 | 1.66 | 87.9 | 0.101 |
| Procyon lotor | Digastric | Tortuosity | Direction Coefficient 0.1 | 55 | 51 | 1.00 | 1.00 | 4.66 | 64.7 | 0.000 |
| Procyon lotor | Digastric | Tortuosity | Minimum Length 100% Cylinder Length | 55 | 35 | 1.00 | 1.01 | -0.79 | 85.2 | 0.432 |
| Procyon lotor | Digastric | Tortuosity | Minimum Length 50% Cylinder Length | 55 | 66 | 1.00 | 1.00 | -0.14 | 119 | 0.892 |
| Procyon lotor | Deep masseter | Tortuosity | Minimum Seed Correlation +50% | 48 | 32 | 1.01 | 1.01 | 0.02 | 66.5 | 0.984 |
| Procyon lotor | Deep masseter | Tortuosity | Minimum Seed Correlation -50% | 48 | 48 | 1.01 | 1.01 | 0.00 | 94 | 1.000 |
| Procyon lotor | Deep masseter | Tortuosity | Minimum Continuation Quality +50% | 48 | 10 | 1.01 | 1.00 | 3.33 | 45.8 | 0.002 |
| Procyon lotor | Deep masseter | Tortuosity | Minimum Continuation Quality -50% | 48 | 108 | 1.01 | 1.06 | -4.46 | 110.8 | 0.000 |
| Procyon lotor | Deep masseter | Tortuosity | Direction Coefficient 0.2 | 48 | 37 | 1.01 | 1.01 | 2.50 | 76.9 | 0.015 |
| Procyon lotor | Deep masseter | Tortuosity | Direction Coefficient 0.1 | 48 | 14 | 1.01 | 1.00 | 5.01 | 57.9 | 0.000 |
| Procyon lotor | Deep masseter | Tortuosity | Minimum Length 100% Cylinder Length | 48 | 5 | 1.01 | 1.02 | -1.00 | 4.3 | 0.372 |
| Procyon lotor | Deep masseter | Tortuosity | Minimum Length 50% Cylinder Length | 48 | 90 | 1.01 | 1.01 | 0.65 | 94.6 | 0.518 |
| Procyon lotor | Deep temporalis | Tortuosity | Minimum Seed Correlation +50% | 72 | 17 | 1.02 | 1.02 | -1.13 | 21.2 | 0.273 |
| Procyon lotor | Deep temporalis | Tortuosity | Minimum Seed Correlation -50% | 72 | 78 | 1.02 | 1.02 | -0.33 | 147.3 | 0.743 |
| Procyon lotor | Deep temporalis | Tortuosity | Minimum Continuation Quality -50% | 72 | 130 | 1.02 | 1.05 | -6.65 | 175.5 | 0.000 |
| Procyon lotor | Deep temporalis | Tortuosity | Direction Coefficient 0.2 | 72 | 64 | 1.02 | 1.01 | 0.86 | 132.7 | 0.393 |
| Procyon lotor | Deep temporalis | Tortuosity | Direction Coefficient 0.1 | 72 | 25 | 1.02 | 1.00 | 4.22 | 79.3 | 0.000 |
| Procyon lotor | Deep temporalis | Tortuosity | Minimum Length 100% Cylinder Length | 72 | 39 | 1.02 | 1.02 | -1.74 | 63.3 | 0.087 |
| Procyon lotor | Deep temporalis | Tortuosity | Minimum Length 50% Cylinder Length | 72 | 120 | 1.02 | 1.01 | 1.56 | 110.7 | 0.122 |
| Procyon lotor | Lateral pterygoid | Tortuosity | Minimum Seed Correlation -50% | 43 | 61 | 1.01 | 1.01 | 0.25 | 85.7 | 0.802 |
| Procyon lotor | Lateral pterygoid | Tortuosity | Minimum Continuation Quality +50% | 43 | 7 | 1.01 | 1.00 | 4.08 | 47.8 | 0.000 |
| Procyon lotor | Lateral pterygoid | Tortuosity | Minimum Continuation Quality -50% | 43 | 73 | 1.01 | 1.04 | -5.27 | 78 | 0.000 |
| Procyon lotor | Lateral pterygoid | Tortuosity | Direction Coefficient 0.2 | 43 | 37 | 1.01 | 1.00 | 3.02 | 55.7 | 0.004 |
| Procyon lotor | Lateral pterygoid | Tortuosity | Direction Coefficient 0.1 | 43 | 28 | 1.01 | 1.00 | 4.83 | 43.8 | 0.000 |
| Procyon lotor | Lateral pterygoid | Tortuosity | Minimum Length 100% Cylinder Length | 43 | 26 | 1.01 | 1.01 | -1.21 | 48.3 | 0.232 |
| Procyon lotor | Lateral pterygoid | Tortuosity | Minimum Length 50% Cylinder Length | 43 | 62 | 1.01 | 1.01 | 0.80 | 85.5 | 0.425 |
| Procyon lotor | Medial pterygoid | Tortuosity | Minimum Seed Correlation +50% | 49 | 4 | 1.01 | 1.01 | 1.98 | 9.4 | 0.078 |
| Procyon lotor | Medial pterygoid | Tortuosity | Minimum Seed Correlation -50% | 49 | 58 | 1.01 | 1.01 | 0.25 | 99.9 | 0.802 |
| Procyon lotor | Medial pterygoid | Tortuosity | Minimum Continuation Quality +50% | 49 | 17 | 1.01 | 1.01 | 3.15 | 63.4 | 0.002 |
| Procyon lotor | Medial pterygoid | Tortuosity | Minimum Continuation Quality -50% | 49 | 66 | 1.01 | 1.04 | -4.30 | 83.4 | 0.000 |
| Procyon lotor | Medial pterygoid | Tortuosity | Direction Coefficient 0.2 | 49 | 42 | 1.01 | 1.01 | 1.95 | 72.2 | 0.055 |
| Procyon lotor | Medial pterygoid | Tortuosity | Direction Coefficient 0.1 | 49 | 33 | 1.01 | 1.00 | 4.06 | 60.7 | 0.000 |
| Procyon lotor | Medial pterygoid | Tortuosity | Minimum Length 100% Cylinder Length | 49 | 28 | 1.01 | 1.02 | -1.17 | 64.2 | 0.245 |
| Procyon lotor | Medial pterygoid | Tortuosity | Minimum Length 50% Cylinder Length | 49 | 76 | 1.01 | 1.01 | 1.16 | 90.6 | 0.251 |
| Procyon lotor | Superficial masseter | Tortuosity | Minimum Seed Correlation +50% | 50 | 17 | 1.02 | 1.03 | -0.33 | 21.5 | 0.745 |
| Procyon lotor | Superficial masseter | Tortuosity | Minimum Seed Correlation -50% | 50 | 61 | 1.02 | 1.02 | 0.12 | 101.6 | 0.906 |
| Procyon lotor | Superficial masseter | Tortuosity | Minimum Continuation Quality +50% | 50 | 16 | 1.02 | 1.00 | 4.64 | 57.5 | 0.000 |
| Procyon lotor | Superficial masseter | Tortuosity | Minimum Continuation Quality -50% | 50 | 76 | 1.02 | 1.07 | -3.44 | 85.4 | 0.001 |
| Procyon lotor | Superficial masseter | Tortuosity | Direction Coefficient 0.2 | 50 | 57 | 1.02 | 1.02 | 1.29 | 98.4 | 0.201 |
| Procyon lotor | Superficial masseter | Tortuosity | Direction Coefficient 0.1 | 50 | 24 | 1.02 | 1.00 | 4.97 | 57.1 | 0.000 |
| Procyon lotor | Superficial masseter | Tortuosity | Minimum Length 100% Cylinder Length | 50 | 32 | 1.02 | 1.03 | -0.81 | 63.7 | 0.422 |
| Procyon lotor | Superficial masseter | Tortuosity | Minimum Length 50% Cylinder Length | 50 | 77 | 1.02 | 1.02 | 1.38 | 93.4 | 0.172 |
| Procyon lotor | Superficial temporalis | Tortuosity | Minimum Seed Correlation +50% | 75 | 43 | 1.02 | 1.02 | -0.62 | 72.6 | 0.539 |
| Procyon lotor | Superficial temporalis | Tortuosity | Minimum Seed Correlation -50% | 75 | 76 | 1.02 | 1.02 | 0.04 | 148.9 | 0.970 |
| Procyon lotor | Superficial temporalis | Tortuosity | Minimum Continuation Quality +50% | 75 | 12 | 1.02 | 1.01 | 2.74 | 38.8 | 0.009 |
| Procyon lotor | Superficial temporalis | Tortuosity | Minimum Continuation Quality -50% | 75 | 129 | 1.02 | 1.06 | -7.79 | 176.8 | 0.000 |
| Procyon lotor | Superficial temporalis | Tortuosity | Direction Coefficient 0.3 | 75 | 98 | 1.02 | 1.03 | -3.66 | 151.5 | 0.000 |
| Procyon lotor | Superficial temporalis | Tortuosity | Direction Coefficient 0.1 | 75 | 40 | 1.02 | 1.00 | 4.93 | 80 | 0.000 |
| Procyon lotor | Superficial temporalis | Tortuosity | Minimum Length 100% Cylinder Length | 75 | 35 | 1.02 | 1.03 | -1.81 | 53.5 | 0.076 |
| Procyon lotor | Superficial temporalis | Tortuosity | Minimum Length 50% Cylinder Length | 75 | 138 | 1.02 | 1.01 | 1.64 | 127.5 | 0.103 |
| Procyon lotor | Zygomaticomandibularis | Tortuosity | Minimum Seed Correlation +50% | 53 | 28 | 1.01 | 1.01 | -0.24 | 47.8 | 0.811 |
| Procyon lotor | Zygomaticomandibularis | Tortuosity | Minimum Seed Correlation -50% | 53 | 56 | 1.01 | 1.01 | 0.16 | 106.4 | 0.877 |
| Procyon lotor | Zygomaticomandibularis | Tortuosity | Minimum Continuation Quality +50% | 53 | 30 | 1.01 | 1.00 | 4.26 | 63.4 | 0.000 |
| Procyon lotor | Zygomaticomandibularis | Tortuosity | Minimum Continuation Quality -50% | 53 | 59 | 1.01 | 1.04 | -3.17 | 64.3 | 0.002 |
| Procyon lotor | Zygomaticomandibularis | Tortuosity | Direction Coefficient 0.2 | 53 | 49 | 1.01 | 1.01 | 3.31 | 69 | 0.001 |
| Procyon lotor | Zygomaticomandibularis | Tortuosity | Direction Coefficient 0.1 | 53 | 42 | 1.01 | 1.00 | 5.54 | 54.1 | 0.000 |
| Procyon lotor | Zygomaticomandibularis | Tortuosity | Minimum Length 100% Cylinder Length | 53 | 42 | 1.01 | 1.02 | -0.36 | 86.6 | 0.717 |
| Procyon lotor | Zygomaticomandibularis | Tortuosity | Minimum Length 50% Cylinder Length | 53 | 68 | 1.01 | 1.01 | 0.98 | 107.5 | 0.327 |
| Martes americana | Digastric | Tortuosity | Minimum Seed Correlation +50% | 43 | 5 | 1.00 | 1.01 | -0.44 | 4.2 | 0.679 |
| Martes americana | Digastric | Tortuosity | Minimum Seed Correlation -50% | 43 | 48 | 1.00 | 1.01 | -0.60 | 84.9 | 0.548 |
| Martes americana | Digastric | Tortuosity | Minimum Continuation Quality +50% | 43 | 31 | 1.00 | 1.00 | 3.62 | 53.6 | 0.001 |
| Martes americana | Digastric | Tortuosity | Minimum Continuation Quality -50% | 43 | 50 | 1.00 | 1.01 | -4.81 | 77.6 | 0.000 |
| Martes americana | Digastric | Tortuosity | Direction Coefficient 0.2 | 43 | 37 | 1.00 | 1.00 | 0.39 | 76.5 | 0.695 |
| Martes americana | Digastric | Tortuosity | Direction Coefficient 0.1 | 43 | 35 | 1.00 | 1.00 | 5.22 | 50.7 | 0.000 |
| Martes americana | Digastric | Tortuosity | Minimum Length 100% Cylinder Length | 43 | 26 | 1.00 | 1.01 | -1.06 | 50 | 0.293 |
| Martes americana | Digastric | Tortuosity | Minimum Length 50% Cylinder Length | 43 | 54 | 1.00 | 1.00 | 0.03 | 90.8 | 0.974 |
| Martes americana | Deep masseter | Tortuosity | Minimum Seed Correlation +50% | 36 | 2 | 1.03 | 1.01 | 2.71 | 35.2 | 0.010 |
| Martes americana | Deep masseter | Tortuosity | Minimum Seed Correlation -50% | 36 | 47 | 1.03 | 1.02 | 0.51 | 71.3 | 0.614 |
| Martes americana | Deep masseter | Tortuosity | Minimum Continuation Quality +50% | 36 | 15 | 1.03 | 1.01 | 3.42 | 36.3 | 0.002 |
| Martes americana | Deep masseter | Tortuosity | Minimum Continuation Quality -50% | 36 | 53 | 1.03 | 1.05 | -1.84 | 83.1 | 0.070 |
| Martes americana | Deep masseter | Tortuosity | Direction Coefficient 0.2 | 36 | 38 | 1.03 | 1.01 | 2.26 | 41.3 | 0.029 |
| Martes americana | Deep masseter | Tortuosity | Direction Coefficient 0.1 | 36 | 24 | 1.03 | 1.00 | 3.54 | 36.7 | 0.001 |
| Martes americana | Deep masseter | Tortuosity | Minimum Length 100% Cylinder Length | 36 | 28 | 1.03 | 1.04 | -0.60 | 55.7 | 0.554 |
| Martes americana | Deep masseter | Tortuosity | Minimum Length 50% Cylinder Length | 36 | 52 | 1.03 | 1.02 | 0.77 | 68.3 | 0.446 |
| Martes americana | Deep temporalis | Tortuosity | Minimum Seed Correlation +50% | 64 | 3 | 1.01 | 1.02 | -0.46 | 2.1 | 0.689 |
| Martes americana | Deep temporalis | Tortuosity | Minimum Seed Correlation -50% | 64 | 75 | 1.01 | 1.01 | 0.47 | 131.4 | 0.636 |
| Martes americana | Deep temporalis | Tortuosity | Minimum Continuation Quality +50% | 64 | 20 | 1.01 | 1.01 | 4.32 | 80.1 | 0.000 |
| Martes americana | Deep temporalis | Tortuosity | Minimum Continuation Quality -50% | 64 | 109 | 1.01 | 1.03 | -4.98 | 138.5 | 0.000 |
| Martes americana | Deep temporalis | Tortuosity | Direction Coefficient 0.3 | 64 | 96 | 1.01 | 1.05 | -6.11 | 108 | 0.000 |
| Martes americana | Deep temporalis | Tortuosity | Direction Coefficient 0.1 | 64 | 42 | 1.01 | 1.01 | 4.13 | 101 | 0.000 |
| Martes americana | Deep temporalis | Tortuosity | Minimum Length 100% Cylinder Length | 64 | 30 | 1.01 | 1.02 | -1.19 | 53.3 | 0.239 |
| Martes americana | Deep temporalis | Tortuosity | Minimum Length 50% Cylinder Length | 64 | 118 | 1.01 | 1.01 | 1.86 | 112.4 | 0.065 |
| Martes americana | Lateral pterygoid | Tortuosity | Minimum Seed Correlation +50% | 28 | 8 | 1.02 | 1.01 | 0.86 | 14.9 | 0.403 |
| Martes americana | Lateral pterygoid | Tortuosity | Minimum Seed Correlation -50% | 28 | 23 | 1.02 | 1.02 | 0.15 | 48.2 | 0.882 |
| Martes americana | Lateral pterygoid | Tortuosity | Minimum Continuation Quality +50% | 28 | 10 | 1.02 | 1.01 | 2.09 | 27.7 | 0.046 |
| Martes americana | Lateral pterygoid | Tortuosity | Minimum Continuation Quality -50% | 28 | 27 | 1.02 | 1.02 | -0.92 | 52.4 | 0.362 |
| Martes americana | Lateral pterygoid | Tortuosity | Direction Coefficient 0.2 | 28 | 18 | 1.02 | 1.01 | 3.50 | 34.4 | 0.001 |
| Martes americana | Lateral pterygoid | Tortuosity | Direction Coefficient 0.1 | 28 | 12 | 1.02 | 1.00 | 4.83 | 28 | 0.000 |
| Martes americana | Lateral pterygoid | Tortuosity | Minimum Length 100% Cylinder Length | 28 | 12 | 1.02 | 1.01 | 1.36 | 37.8 | 0.181 |
| Martes americana | Lateral pterygoid | Tortuosity | Minimum Length 50% Cylinder Length | 28 | 28 | 1.02 | 1.01 | 0.88 | 53.8 | 0.383 |
| Martes americana | Medial pterygoid | Tortuosity | Minimum Seed Correlation +50% | 36 | 3 | 1.04 | 1.02 | 2.75 | 9.9 | 0.021 |
| Martes americana | Medial pterygoid | Tortuosity | Minimum Seed Correlation -50% | 36 | 50 | 1.04 | 1.04 | -0.05 | 76.7 | 0.959 |
| Martes americana | Medial pterygoid | Tortuosity | Minimum Continuation Quality +50% | 36 | 24 | 1.04 | 1.02 | 3.75 | 52.8 | 0.000 |
| Martes americana | Medial pterygoid | Tortuosity | Minimum Continuation Quality -50% | 36 | 41 | 1.04 | 1.07 | -3.03 | 65.8 | 0.004 |
| Martes americana | Medial pterygoid | Tortuosity | Direction Coefficient 0.2 | 36 | 29 | 1.04 | 1.02 | 3.08 | 57.3 | 0.003 |
| Martes americana | Medial pterygoid | Tortuosity | Direction Coefficient 0.1 | 36 | 21 | 1.04 | 1.01 | 6.81 | 37.7 | 0.000 |
| Martes americana | Medial pterygoid | Tortuosity | Minimum Length 100% Cylinder Length | 36 | 29 | 1.04 | 1.05 | -0.82 | 59.3 | 0.417 |
| Martes americana | Medial pterygoid | Tortuosity | Minimum Length 50% Cylinder Length | 36 | 41 | 1.04 | 1.04 | 0.60 | 73.7 | 0.551 |
| Martes americana | Superficial masseter | Tortuosity | Minimum Seed Correlation +50% | 67 | 5 | 1.00 | 1.00 | 1.07 | 5 | 0.335 |
| Martes americana | Superficial masseter | Tortuosity | Minimum Seed Correlation -50% | 67 | 93 | 1.00 | 1.00 | 1.10 | 133.4 | 0.272 |
| Martes americana | Superficial masseter | Tortuosity | Minimum Continuation Quality +50% | 67 | 23 | 1.00 | 1.00 | 6.42 | 75.6 | 0.000 |
| Martes americana | Superficial masseter | Tortuosity | Minimum Continuation Quality -50% | 67 | 89 | 1.00 | 1.02 | -4.80 | 91.3 | 0.000 |
| Martes americana | Superficial masseter | Tortuosity | Direction Coefficient 0.2 | 67 | 71 | 1.00 | 1.01 | -3.11 | 91 | 0.003 |
| Martes americana | Superficial masseter | Tortuosity | Direction Coefficient 0.1 | 67 | 62 | 1.00 | 1.00 | 3.57 | 109.3 | 0.001 |
| Martes americana | Superficial masseter | Tortuosity | Minimum Length 100% Cylinder Length | 67 | 48 | 1.00 | 1.00 | -0.40 | 98.2 | 0.687 |
| Martes americana | Superficial masseter | Tortuosity | Minimum Length 50% Cylinder Length | 67 | 88 | 1.00 | 1.00 | 0.68 | 140.5 | 0.497 |
| Martes americana | Superficial temporalis | Tortuosity | Minimum Seed Correlation +50% | 54 | 3 | 1.01 | 1.01 | 0.24 | 3.1 | 0.824 |
| Martes americana | Superficial temporalis | Tortuosity | Minimum Seed Correlation -50% | 54 | 63 | 1.01 | 1.01 | 0.20 | 109.6 | 0.841 |
| Martes americana | Superficial temporalis | Tortuosity | Minimum Continuation Quality +50% | 54 | 27 | 1.01 | 1.00 | 3.84 | 60.3 | 0.000 |
| Martes americana | Superficial temporalis | Tortuosity | Minimum Continuation Quality -50% | 54 | 67 | 1.01 | 1.03 | -2.56 | 80.1 | 0.012 |
| Martes americana | Superficial temporalis | Tortuosity | Direction Coefficient 0.3 | 54 | 68 | 1.01 | 1.04 | -5.37 | 90.9 | 0.000 |
| Martes americana | Superficial temporalis | Tortuosity | Direction Coefficient 0.1 | 54 | 46 | 1.01 | 1.00 | 3.17 | 60.4 | 0.002 |
| Martes americana | Superficial temporalis | Tortuosity | Minimum Length 100% Cylinder Length | 54 | 34 | 1.01 | 1.02 | -1.15 | 59.2 | 0.256 |
| Martes americana | Superficial temporalis | Tortuosity | Minimum Length 50% Cylinder Length | 54 | 82 | 1.01 | 1.01 | 1.17 | 100.6 | 0.243 |
| Martes americana | Zygomaticomandibularis | Tortuosity | Minimum Seed Correlation -50% | 39 | 45 | 1.02 | 1.02 | -0.16 | 80.8 | 0.870 |
| Martes americana | Zygomaticomandibularis | Tortuosity | Minimum Continuation Quality +50% | 39 | 19 | 1.02 | 1.01 | 3.75 | 56 | 0.000 |
| Martes americana | Zygomaticomandibularis | Tortuosity | Minimum Continuation Quality -50% | 39 | 54 | 1.02 | 2.14 | -1.04 | 53 | 0.304 |
| Martes americana | Zygomaticomandibularis | Tortuosity | Direction Coefficient 0.2 | 39 | 35 | 1.02 | 1.01 | 2.29 | 68.2 | 0.025 |
| Martes americana | Zygomaticomandibularis | Tortuosity | Direction Coefficient 0.1 | 39 | 21 | 1.02 | 1.00 | 6.89 | 40.1 | 0.000 |
| Martes americana | Zygomaticomandibularis | Tortuosity | Minimum Length 100% Cylinder Length | 39 | 25 | 1.02 | 1.03 | -1.02 | 50.9 | 0.311 |
| Martes americana | Zygomaticomandibularis | Tortuosity | Minimum Length 50% Cylinder Length | 39 | 51 | 1.02 | 1.02 | 0.75 | 79.5 | 0.454 |
| Mephitis mephitis | Digastric | Tortuosity | Minimum Seed Correlation +50% | 54 | 17 | 1.01 | 1.01 | -1.50 | 26.2 | 0.147 |
| Mephitis mephitis | Digastric | Tortuosity | Minimum Seed Correlation -50% | 54 | 61 | 1.01 | 1.01 | 0.28 | 111 | 0.781 |
| Mephitis mephitis | Digastric | Tortuosity | Minimum Continuation Quality +50% | 54 | 31 | 1.01 | 1.00 | 4.36 | 82.4 | 0.000 |
| Mephitis mephitis | Digastric | Tortuosity | Minimum Continuation Quality -50% | 54 | 65 | 1.01 | 1.02 | -3.03 | 94.4 | 0.003 |
| Mephitis mephitis | Digastric | Tortuosity | Direction Coefficient 0.2 | 54 | 52 | 1.01 | 1.01 | 2.48 | 104 | 0.015 |
| Mephitis mephitis | Digastric | Tortuosity | Direction Coefficient 0.1 | 54 | 43 | 1.01 | 1.00 | 6.08 | 78.7 | 0.000 |
| Mephitis mephitis | Digastric | Tortuosity | Minimum Length 100% Cylinder Length | 54 | 34 | 1.01 | 1.01 | -1.10 | 73.4 | 0.276 |
| Mephitis mephitis | Digastric | Tortuosity | Minimum Length 50% Cylinder Length | 54 | 70 | 1.01 | 1.01 | 0.96 | 114.1 | 0.339 |
| Mephitis mephitis | Deep masseter | Tortuosity | Minimum Seed Correlation +50% | 60 | 39 | 1.02 | 1.03 | -0.71 | 72.1 | 0.480 |
| Mephitis mephitis | Deep masseter | Tortuosity | Minimum Seed Correlation -50% | 60 | 60 | 1.02 | 1.02 | 0.00 | 118 | 1.000 |
| Mephitis mephitis | Deep masseter | Tortuosity | Minimum Continuation Quality -50% | 60 | 527 | 1.02 | 1.05 | -6.03 | 150.2 | 0.000 |
| Mephitis mephitis | Deep masseter | Tortuosity | Direction Coefficient 0.3 | 60 | 118 | 1.02 | 1.04 | -4.05 | 167.6 | 0.000 |
| Mephitis mephitis | Deep masseter | Tortuosity | Direction Coefficient 0.1 | 60 | 6 | 1.02 | 1.00 | 4.71 | 62.1 | 0.000 |
| Mephitis mephitis | Deep masseter | Tortuosity | Minimum Length 100% Cylinder Length | 60 | 3 | 1.02 | 1.09 | -2.00 | 2 | 0.181 |
| Mephitis mephitis | Deep masseter | Tortuosity | Minimum Length 50% Cylinder Length | 60 | 212 | 1.02 | 1.01 | 2.51 | 72.4 | 0.014 |
| Mephitis mephitis | Deep temporalis | Tortuosity | Minimum Seed Correlation -50% | 59 | 110 | 1.02 | 1.02 | 1.11 | 111.2 | 0.270 |
| Mephitis mephitis | Deep temporalis | Tortuosity | Minimum Continuation Quality +50% | 59 | 2 | 1.02 | 1.01 | 4.31 | 4 | 0.012 |
| Mephitis mephitis | Deep temporalis | Tortuosity | Minimum Continuation Quality -50% | 59 | 181 | 1.02 | 1.12 | -9.24 | 190.9 | 0.000 |
| Mephitis mephitis | Deep temporalis | Tortuosity | Direction Coefficient 0.2 | 59 | 40 | 1.02 | 1.01 | 3.85 | 90.5 | 0.000 |
| Mephitis mephitis | Deep temporalis | Tortuosity | Direction Coefficient 0.1 | 59 | 13 | 1.02 | 1.00 | 7.07 | 69.9 | 0.000 |
| Mephitis mephitis | Deep temporalis | Tortuosity | Minimum Length 100% Cylinder Length | 59 | 27 | 1.02 | 1.03 | -2.79 | 48.9 | 0.008 |
| Mephitis mephitis | Deep temporalis | Tortuosity | Minimum Length 50% Cylinder Length | 59 | 96 | 1.02 | 1.01 | 2.42 | 112.5 | 0.017 |
| Mephitis mephitis | Lateral pterygoid | Tortuosity | Minimum Seed Correlation +50% | 39 | 24 | 1.01 | 1.01 | 0.26 | 57.8 | 0.797 |
| Mephitis mephitis | Lateral pterygoid | Tortuosity | Minimum Seed Correlation -50% | 39 | 40 | 1.01 | 1.01 | 0.10 | 76.9 | 0.918 |
| Mephitis mephitis | Lateral pterygoid | Tortuosity | Minimum Continuation Quality +50% | 39 | 18 | 1.01 | 1.01 | 1.14 | 50.1 | 0.258 |
| Mephitis mephitis | Lateral pterygoid | Tortuosity | Minimum Continuation Quality -50% | 39 | 61 | 1.01 | 1.03 | -4.02 | 77.5 | 0.000 |
| Mephitis mephitis | Lateral pterygoid | Tortuosity | Direction Coefficient 0.2 | 39 | 38 | 1.01 | 1.01 | 1.47 | 69.8 | 0.146 |
| Mephitis mephitis | Lateral pterygoid | Tortuosity | Direction Coefficient 0.1 | 39 | 23 | 1.01 | 1.00 | 4.46 | 41 | 0.000 |
| Mephitis mephitis | Lateral pterygoid | Tortuosity | Minimum Length 100% Cylinder Length | 39 | 20 | 1.01 | 1.02 | -1.24 | 32.2 | 0.223 |
| Mephitis mephitis | Lateral pterygoid | Tortuosity | Minimum Length 50% Cylinder Length | 39 | 64 | 1.01 | 1.01 | 0.54 | 81.1 | 0.589 |
| Mephitis mephitis | Medial pterygoid | Tortuosity | Minimum Seed Correlation +50% | 34 | 9 | 1.02 | 1.02 | 0.56 | 14.4 | 0.587 |
| Mephitis mephitis | Medial pterygoid | Tortuosity | Minimum Seed Correlation -50% | 34 | 38 | 1.02 | 1.02 | 0.23 | 68.5 | 0.822 |
| Mephitis mephitis | Medial pterygoid | Tortuosity | Minimum Continuation Quality +50% | 34 | 7 | 1.02 | 1.01 | 3.64 | 36.3 | 0.001 |
| Mephitis mephitis | Medial pterygoid | Tortuosity | Minimum Continuation Quality -50% | 34 | 75 | 1.02 | 1.05 | -4.64 | 105.7 | 0.000 |
| Mephitis mephitis | Medial pterygoid | Tortuosity | Direction Coefficient 0.2 | 34 | 24 | 1.02 | 1.01 | 1.96 | 54 | 0.055 |
| Mephitis mephitis | Medial pterygoid | Tortuosity | Direction Coefficient 0.1 | 34 | 11 | 1.02 | 1.00 | 4.91 | 36.7 | 0.000 |
| Mephitis mephitis | Medial pterygoid | Tortuosity | Minimum Length 100% Cylinder Length | 34 | 18 | 1.02 | 1.03 | -1.24 | 30.5 | 0.225 |
| Mephitis mephitis | Medial pterygoid | Tortuosity | Minimum Length 50% Cylinder Length | 34 | 38 | 1.02 | 1.02 | 0.44 | 68.8 | 0.662 |
| Mephitis mephitis | Superficial masseter | Tortuosity | Minimum Seed Correlation -50% | 57 | 71 | 1.01 | 1.01 | 0.21 | 121.2 | 0.833 |
| Mephitis mephitis | Superficial masseter | Tortuosity | Minimum Continuation Quality +50% | 57 | 6 | 1.01 | 1.00 | 5.36 | 59.8 | 0.000 |
| Mephitis mephitis | Superficial masseter | Tortuosity | Minimum Continuation Quality -50% | 57 | 117 | 1.01 | 1.10 | -2.64 | 116.4 | 0.010 |
| Mephitis mephitis | Superficial masseter | Tortuosity | Direction Coefficient 0.2 | 57 | 46 | 1.01 | 1.01 | 2.50 | 85.4 | 0.015 |
| Mephitis mephitis | Superficial masseter | Tortuosity | Direction Coefficient 0.1 | 57 | 26 | 1.01 | 1.00 | 5.18 | 63.4 | 0.000 |
| Mephitis mephitis | Superficial masseter | Tortuosity | Minimum Length 100% Cylinder Length | 57 | 30 | 1.01 | 1.02 | -1.65 | 47.2 | 0.105 |
| Mephitis mephitis | Superficial masseter | Tortuosity | Minimum Length 50% Cylinder Length | 57 | 96 | 1.01 | 1.01 | 1.09 | 111.8 | 0.278 |
| Mephitis mephitis | Superficial temporalis | Tortuosity | Minimum Seed Correlation +50% | 65 | 20 | 1.01 | 1.02 | -0.77 | 26.6 | 0.448 |
| Mephitis mephitis | Superficial temporalis | Tortuosity | Minimum Seed Correlation -50% | 65 | 67 | 1.01 | 1.01 | 0.10 | 129.8 | 0.918 |
| Mephitis mephitis | Superficial temporalis | Tortuosity | Minimum Continuation Quality +50% | 65 | 21 | 1.01 | 1.01 | 3.68 | 73.8 | 0.000 |
| Mephitis mephitis | Superficial temporalis | Tortuosity | Minimum Continuation Quality -50% | 65 | 77 | 1.01 | 1.05 | -4.54 | 83 | 0.000 |
| Mephitis mephitis | Superficial temporalis | Tortuosity | Direction Coefficient 0.3 | 65 | 69 | 1.01 | 1.03 | -3.15 | 76.8 | 0.002 |
| Mephitis mephitis | Superficial temporalis | Tortuosity | Direction Coefficient 0.1 | 65 | 45 | 1.01 | 1.01 | 4.20 | 106.2 | 0.000 |
| Mephitis mephitis | Superficial temporalis | Tortuosity | Minimum Length 100% Cylinder Length | 65 | 34 | 1.01 | 1.02 | -2.86 | 54 | 0.006 |
| Mephitis mephitis | Superficial temporalis | Tortuosity | Minimum Length 50% Cylinder Length | 65 | 95 | 1.01 | 1.01 | 1.10 | 129.9 | 0.272 |
| Mephitis mephitis | Zygomaticomandibularis | Tortuosity | Minimum Seed Correlation +50% | 55 | 3 | 1.01 | 1.00 | 4.89 | 51.9 | 0.000 |
| Mephitis mephitis | Zygomaticomandibularis | Tortuosity | Minimum Seed Correlation -50% | 55 | 71 | 1.01 | 1.01 | 0.15 | 114.3 | 0.878 |
| Mephitis mephitis | Zygomaticomandibularis | Tortuosity | Minimum Continuation Quality +50% | 55 | 5 | 1.01 | 1.00 | 4.95 | 50.1 | 0.000 |
| Mephitis mephitis | Zygomaticomandibularis | Tortuosity | Minimum Continuation Quality -50% | 55 | 84 | 1.01 | 1.06 | -5.43 | 88.4 | 0.000 |
| Mephitis mephitis | Zygomaticomandibularis | Tortuosity | Direction Coefficient 0.2 | 55 | 37 | 1.01 | 1.01 | 2.78 | 88.8 | 0.007 |
| Mephitis mephitis | Zygomaticomandibularis | Tortuosity | Direction Coefficient 0.1 | 55 | 25 | 1.01 | 1.00 | 6.56 | 56.8 | 0.000 |
| Mephitis mephitis | Zygomaticomandibularis | Tortuosity | Minimum Length 100% Cylinder Length | 55 | 28 | 1.01 | 1.02 | -1.34 | 46.1 | 0.188 |
| Mephitis mephitis | Zygomaticomandibularis | Tortuosity | Minimum Length 50% Cylinder Length | 55 | 69 | 1.01 | 1.01 | 0.23 | 116.4 | 0.816 |
| Daubentonia madagascariensis | Deep masseter | Tortuosity | Minimum Seed Correlation +50% | 64 | 2 | 1.01 | 1.00 | 4.24 | 2.5 | 0.035 |
| Daubentonia madagascariensis | Deep masseter | Tortuosity | Minimum Seed Correlation -50% | 64 | 119 | 1.01 | 1.01 | 0.75 | 114.2 | 0.454 |
| Daubentonia madagascariensis | Deep masseter | Tortuosity | Minimum Continuation Quality +50% | 64 | 2 | 1.01 | 1.00 | 0.68 | 1.1 | 0.610 |
| Daubentonia madagascariensis | Deep masseter | Tortuosity | Minimum Continuation Quality -50% | 64 | 75 | 1.01 | 1.03 | -5.33 | 77.6 | 0.000 |
| Daubentonia madagascariensis | Deep masseter | Tortuosity | Direction Coefficient 0.2 | 64 | 57 | 1.01 | 1.00 | 3.11 | 106.3 | 0.002 |
| Daubentonia madagascariensis | Deep masseter | Tortuosity | Direction Coefficient 0.1 | 64 | 44 | 1.01 | 1.00 | 6.82 | 72 | 0.000 |
| Daubentonia madagascariensis | Deep masseter | Tortuosity | Minimum Length 100% Cylinder Length | 64 | 44 | 1.01 | 1.01 | -1.38 | 96 | 0.171 |
| Daubentonia madagascariensis | Deep masseter | Tortuosity | Minimum Length 50% Cylinder Length | 64 | 81 | 1.01 | 1.01 | 1.02 | 132.2 | 0.312 |
| Daubentonia madagascariensis | Deep temporalis | Tortuosity | Minimum Seed Correlation +50% | 93 | 28 | 1.01 | 1.01 | -0.95 | 42.6 | 0.348 |
| Daubentonia madagascariensis | Deep temporalis | Tortuosity | Minimum Seed Correlation -50% | 93 | 110 | 1.01 | 1.01 | 0.28 | 190.2 | 0.781 |
| Daubentonia madagascariensis | Deep temporalis | Tortuosity | Minimum Continuation Quality +50% | 93 | 60 | 1.01 | 1.00 | 3.77 | 97 | 0.000 |
| Daubentonia madagascariensis | Deep temporalis | Tortuosity | Minimum Continuation Quality -50% | 93 | 109 | 1.01 | 1.02 | -3.70 | 175.8 | 0.000 |
| Daubentonia madagascariensis | Deep temporalis | Tortuosity | Direction Coefficient 0.2 | 93 | 106 | 1.01 | 1.01 | -0.49 | 178.9 | 0.623 |
| Daubentonia madagascariensis | Deep temporalis | Tortuosity | Direction Coefficient 0.1 | 93 | 77 | 1.01 | 1.00 | 3.94 | 96 | 0.000 |
| Daubentonia madagascariensis | Deep temporalis | Tortuosity | Minimum Length 100% Cylinder Length | 93 | 57 | 1.01 | 1.01 | -1.18 | 100.2 | 0.241 |
| Daubentonia madagascariensis | Deep temporalis | Tortuosity | Minimum Length 50% Cylinder Length | 93 | 128 | 1.01 | 1.01 | 0.73 | 182.3 | 0.470 |
| Daubentonia madagascariensis | Lateral pterygoid | Tortuosity | Minimum Seed Correlation +50% | 52 | 17 | 1.02 | 1.02 | -0.72 | 23.9 | 0.479 |
| Daubentonia madagascariensis | Lateral pterygoid | Tortuosity | Minimum Seed Correlation -50% | 52 | 55 | 1.02 | 1.02 | 0.26 | 104.6 | 0.793 |
| Daubentonia madagascariensis | Lateral pterygoid | Tortuosity | Minimum Continuation Quality +50% | 52 | 21 | 1.02 | 1.01 | 4.67 | 71 | 0.000 |
| Daubentonia madagascariensis | Lateral pterygoid | Tortuosity | Minimum Continuation Quality -50% | 52 | 59 | 1.02 | 1.03 | -2.46 | 84.5 | 0.016 |
| Daubentonia madagascariensis | Lateral pterygoid | Tortuosity | Direction Coefficient 0.2 | 52 | 50 | 1.02 | 1.01 | 2.31 | 88.8 | 0.023 |
| Daubentonia madagascariensis | Lateral pterygoid | Tortuosity | Direction Coefficient 0.1 | 52 | 37 | 1.02 | 1.00 | 6.85 | 53 | 0.000 |
| Daubentonia madagascariensis | Lateral pterygoid | Tortuosity | Minimum Length 100% Cylinder Length | 52 | 35 | 1.02 | 1.02 | -0.69 | 64.9 | 0.492 |
| Daubentonia madagascariensis | Lateral pterygoid | Tortuosity | Minimum Length 50% Cylinder Length | 52 | 68 | 1.02 | 1.02 | 0.25 | 111.3 | 0.806 |
| Daubentonia madagascariensis | Medial pterygoid | Tortuosity | Minimum Seed Correlation +50% | 55 | 13 | 1.01 | 1.01 | 0.63 | 26.8 | 0.533 |
| Daubentonia madagascariensis | Medial pterygoid | Tortuosity | Minimum Seed Correlation -50% | 55 | 66 | 1.01 | 1.01 | 0.19 | 112 | 0.854 |
| Daubentonia madagascariensis | Medial pterygoid | Tortuosity | Minimum Continuation Quality +50% | 55 | 14 | 1.01 | 1.00 | 2.99 | 67 | 0.004 |
| Daubentonia madagascariensis | Medial pterygoid | Tortuosity | Minimum Continuation Quality -50% | 55 | 94 | 1.01 | 1.03 | -4.91 | 115.5 | 0.000 |
| Daubentonia madagascariensis | Medial pterygoid | Tortuosity | Direction Coefficient 0.2 | 55 | 46 | 1.01 | 1.00 | 2.33 | 74.6 | 0.023 |
| Daubentonia madagascariensis | Medial pterygoid | Tortuosity | Direction Coefficient 0.1 | 55 | 39 | 1.01 | 1.00 | 4.23 | 55.5 | 0.000 |
| Daubentonia madagascariensis | Medial pterygoid | Tortuosity | Minimum Length 100% Cylinder Length | 55 | 27 | 1.01 | 1.01 | -0.99 | 67.1 | 0.324 |
| Daubentonia madagascariensis | Medial pterygoid | Tortuosity | Minimum Length 50% Cylinder Length | 55 | 95 | 1.01 | 1.01 | 0.94 | 105.8 | 0.349 |
| Daubentonia madagascariensis | Superficial masseter | Tortuosity | Minimum Seed Correlation +50% | 60 | 14 | 1.02 | 1.01 | 0.93 | 62.6 | 0.358 |
| Daubentonia madagascariensis | Superficial masseter | Tortuosity | Minimum Seed Correlation -50% | 60 | 63 | 1.02 | 1.02 | -0.05 | 120.5 | 0.964 |
| Daubentonia madagascariensis | Superficial masseter | Tortuosity | Minimum Continuation Quality +50% | 60 | 9 | 1.02 | 1.01 | 2.06 | 53.1 | 0.044 |
| Daubentonia madagascariensis | Superficial masseter | Tortuosity | Minimum Continuation Quality -50% | 60 | 90 | 1.02 | 1.05 | -3.82 | 137.6 | 0.000 |
| Daubentonia madagascariensis | Superficial masseter | Tortuosity | Direction Coefficient 0.2 | 60 | 55 | 1.02 | 1.01 | 2.24 | 63.6 | 0.028 |
| Daubentonia madagascariensis | Superficial masseter | Tortuosity | Direction Coefficient 0.1 | 60 | 33 | 1.02 | 1.00 | 3.21 | 59.3 | 0.002 |
| Daubentonia madagascariensis | Superficial masseter | Tortuosity | Minimum Length 100% Cylinder Length | 60 | 32 | 1.02 | 1.03 | -1.09 | 49.9 | 0.280 |
| Daubentonia madagascariensis | Superficial masseter | Tortuosity | Minimum Length 50% Cylinder Length | 60 | 79 | 1.02 | 1.01 | 1.17 | 71.1 | 0.245 |
| Daubentonia madagascariensis | Superficial temporalis | Tortuosity | Minimum Seed Correlation +50% | 95 | 26 | 1.03 | 1.05 | -1.79 | 30.2 | 0.083 |
| Daubentonia madagascariensis | Superficial temporalis | Tortuosity | Minimum Seed Correlation -50% | 95 | 99 | 1.03 | 1.02 | 0.13 | 191.4 | 0.900 |
| Daubentonia madagascariensis | Superficial temporalis | Tortuosity | Minimum Continuation Quality +50% | 95 | 55 | 1.03 | 1.02 | 2.06 | 138.3 | 0.041 |
| Daubentonia madagascariensis | Superficial temporalis | Tortuosity | Minimum Continuation Quality -50% | 95 | 108 | 1.03 | 1.05 | -2.48 | 138.6 | 0.014 |
| Daubentonia madagascariensis | Superficial temporalis | Tortuosity | Direction Coefficient 0.3 | 95 | 101 | 1.03 | 1.06 | -3.82 | 138.3 | 0.000 |
| Daubentonia madagascariensis | Superficial temporalis | Tortuosity | Direction Coefficient 0.1 | 95 | 65 | 1.03 | 1.01 | 3.52 | 136.1 | 0.001 |
| Daubentonia madagascariensis | Superficial temporalis | Tortuosity | Minimum Length 100% Cylinder Length | 95 | 48 | 1.03 | 1.04 | -1.83 | 75.5 | 0.072 |
| Daubentonia madagascariensis | Superficial temporalis | Tortuosity | Minimum Length 50% Cylinder Length | 95 | 141 | 1.03 | 1.02 | 1.20 | 181.7 | 0.232 |
| Daubentonia madagascariensis | Zygomaticomandibularis | Tortuosity | Minimum Seed Correlation +50% | 43 | 16 | 1.02 | 1.02 | -0.09 | 25.7 | 0.926 |
| Daubentonia madagascariensis | Zygomaticomandibularis | Tortuosity | Minimum Seed Correlation -50% | 43 | 43 | 1.02 | 1.02 | 0.00 | 84 | 1.000 |
| Daubentonia madagascariensis | Zygomaticomandibularis | Tortuosity | Minimum Continuation Quality +50% | 43 | 18 | 1.02 | 1.01 | 3.40 | 58.5 | 0.001 |
| Daubentonia madagascariensis | Zygomaticomandibularis | Tortuosity | Minimum Continuation Quality -50% | 43 | 49 | 1.02 | 1.03 | -2.49 | 88.7 | 0.015 |
| Daubentonia madagascariensis | Zygomaticomandibularis | Tortuosity | Direction Coefficient 0.2 | 43 | 37 | 1.02 | 1.01 | 2.18 | 69.1 | 0.033 |
| Daubentonia madagascariensis | Zygomaticomandibularis | Tortuosity | Direction Coefficient 0.1 | 43 | 25 | 1.02 | 1.00 | 5.77 | 47.9 | 0.000 |
| Daubentonia madagascariensis | Zygomaticomandibularis | Tortuosity | Minimum Length 100% Cylinder Length | 43 | 23 | 1.02 | 1.03 | -1.75 | 41.6 | 0.087 |
| Daubentonia madagascariensis | Zygomaticomandibularis | Tortuosity | Minimum Length 50% Cylinder Length | 43 | 52 | 1.02 | 1.02 | 0.54 | 85.3 | 0.592 |

**Supplemental Table 2. Statistical results derived from comparisons of fascicle length and tortuosity between a standardized GoodFibes parameter input and locally optimized input variables that utilized slice-specific threshold values for each muscle region and applied a more aggressive upper limit to the cutoff criteria (see materials and methods in full text for details).**

| Species | Muscle | Measure | N_LocallyOptimal | N_StandardProtocol | mean_LocallyOptimal | mean_StandardProtocol |  | t_statistic | df | p_value |
| --- | --- | --- | --- | --- | --- | --- | --- | --- | --- | --- |
| Daubentonia Madagascariensis | Deep masseter | Fascicle Length (mm) | 29 | 33 | 8.55 | 12.25 |  | -4.00 | 58.5 | 0.000 |
| Daubentonia Madagascariensis | Deep temporalis | Fascicle Length (mm) | 44 | 44 | 25.04 | 36.45 |  | -4.60 | 85.9 | 0.000 |
| Daubentonia Madagascariensis | Lateral pterygoid | Fascicle Length (mm) | 18 | 21 | 11.13 | 18.47 |  | -4.28 | 37 | 0.000 |
| Daubentonia Madagascariensis | Medial pterygoid | Fascicle Length (mm) | 34 | 34 | 21.17 | 30.42 |  | -4.96 | 53.1 | 0.000 |
| Daubentonia Madagascariensis | Superficial masseter | Fascicle Length (mm) | 39 | 46 | 10.09 | 25.19 |  | -11.78 | 75 | 0.000 |
| Daubentonia Madagascariensis | Superficial temporalis | Fascicle Length (mm) | 35 | 47 | 25.61 | 48.46 |  | -4.72 | 67.8 | 0.000 |
| Daubentonia Madagascariensis | Zygomaticomandibularis | Fascicle Length (mm) | 20 | 20 | 10.61 | 16.30 |  | -4.66 | 38 | 0.000 |
| Eulemur Coronatus | Deep masseter | Fascicle Length (mm) | 22 | 25 | 16.71 | 17.12 |  | -0.36 | 25.2 | 0.722 |
| Eulemur Coronatus | Deep temporalis | Fascicle Length (mm) | 24 | 32 | 34.77 | 37.21 |  | -1.65 | 53.7 | 0.104 |
| Eulemur Coronatus | Lateral pterygoid | Fascicle Length (mm) | 22 | 19 | 15.06 | 14.22 |  | 1.39 | 26.8 | 0.176 |
| Eulemur Coronatus | Medial pterygoid | Fascicle Length (mm) | 22 | 36 | 6.37 | 9.51 |  | -4.12 | 50.3 | 0.000 |
| Eulemur Coronatus | Superficial masseter | Fascicle Length (mm) | 26 | 26 | 17.91 | 23.06 |  | 3.11 | 36.37 | 0.003 |
| Eulemur Coronatus | Superficial temporalis | Fascicle Length (mm) | 20 | 24 | 25.65 | 28.43 |  | -3.59 | 34.6 | 0.001 |
| Eulemur Coronatus | Zygomaticomandibularis | Fascicle Length (mm) | 26 | 24 | 11.08 | 11.85 |  | -0.79 | 43.9 | 0.437 |
| Eulemur Coronatus | Anterior digastric | Fascicle Length (mm) | 12 | 22 | 22.36 | 23.41 |  | -2.39 | 12.1 | 0.034 |
| Eulemur Coronatus | Posterior digastric | Fascicle Length (mm) | 19 | 21 | 21.57 | 22.64 |  | -1.20 | 24.6 | 0.241 |
| Eulemur Coronatus | Zygomatic temporalis | Fascicle Length (mm) | 18 | 18 | 14.59 | 15.86 |  | -1.67 | 33.9 | 0.105 |
| Lontra Canadensis | Deep masseter | Fascicle Length (mm) | 17 | 23 | 7.98 | 11.91 |  | -3.84 | 37.6 | 0.000 |
| Lontra Canadensis | Deep temporalis | Fascicle Length (mm) | 40 | 40 | 37.53 | 41.29 |  | -1.21 | 77.8 | 0.229 |
| Lontra Canadensis | Lateral pterygoid | Fascicle Length (mm) | 14 | 14 | 12.20 | 12.18 |  | 0.05 | 24.9 | 0.963 |
| Lontra Canadensis | Medial pterygoid | Fascicle Length (mm) | 34 | 52 | 14.28 | 20.04 |  | -6.03 | 76.7 | 0.000 |
| Lontra Canadensis | Superficial masseter | Fascicle Length (mm) | 24 | 66 | 18.11 | 18.42 |  | -0.33 | 69 | 0.745 |
| Lontra Canadensis | Superficial temporalis | Fascicle Length (mm) | 41 | 43 | 34.16 | 67.45 |  | -9.96 | 79 | 0.000 |
| Lontra Canadensis | Zygomaticomandibularis | Fascicle Length (mm) | 10 | 29 | 5.00 | 6.88 |  | -2.93 | 17.2 | 0.009 |
| Lontra Canadensis | Digastric | Fascicle Length (mm) | 44 | 39 | 41.85 | 55.11 |  | -12.74 | 48.2 | 0.000 |
| Martes Americana | Deep masseter | Fascicle Length (mm) | 24 | 28 | 18.72 | 19.25 |  | -0.45 | 47 | 0.654 |
| Martes Americana | Deep temporalis | Fascicle Length (mm) | 35 | 67 | 33.35 | 35.77 |  | -1.21 | 62.8 | 0.232 |
| Martes Americana | Lateral pterygoid | Fascicle Length (mm) | 12 | 9 | 9.54 | 9.75 |  | -0.24 | 17.5 | 0.810 |
| Martes Americana | Medial pterygoid | Fascicle Length (mm) | 28 | 39 | 11.50 | 12.15 |  | -0.72 | 52.2 | 0.477 |
| Martes Americana | Superficial masseter | Fascicle Length (mm) | 31 | 59 | 20.45 | 21.50 |  | -1.01 | 51.3 | 0.319 |
| Martes Americana | Superficial temporalis | Fascicle Length (mm) | 36 | 49 | 31.13 | 33.80 |  | -1.08 | 67.9 | 0.284 |
| Martes Americana | Zygomaticomandibularis | Fascicle Length (mm) | 17 | 36 | 19.83 | 20.62 |  | -0.52 | 37.7 | 0.603 |
| Martes Americana | Digastric | Fascicle Length (mm) | 37 | 37 | 39.80 | 39.80 |  | 0.00 | 72 | 1.000 |
| Mephitis Mephitis | Deep masseter | Fascicle Length (mm) | 16 | 25 | 8.37 | 9.97 |  | -2.04 | 32.5 | 0.050 |
| Mephitis Mephitis | Deep temporalis | Fascicle Length (mm) | 30 | 36 | 38.11 | 34.78 |  | 2.58 | 47 | 0.013 |
| Mephitis Mephitis | Lateral pterygoid | Fascicle Length (mm) | 7 | 10 | 3.83 | 5.17 |  | -2.75 | 11.8 | 0.018 |
| Mephitis Mephitis | Medial pterygoid | Fascicle Length (mm) | 19 | 28 | 10.20 | 10.73 |  | -0.76 | 42.6 | 0.450 |
| Mephitis Mephitis | Superficial masseter | Fascicle Length (mm) | 24 | 41 | 12.44 | 12.40 |  | 0.04 | 55.4 | 0.968 |
| Mephitis Mephitis | Superficial temporalis | Fascicle Length (mm) | 29 | 35 | 41.03 | 42.56 |  | -0.89 | 33.5 | 0.379 |
| Mephitis Mephitis | Zygomaticomandibularis | Fascicle Length (mm) | 25 | 44 | 9.04 | 10.03 |  | -1.43 | 56.4 | 0.158 |
| Mephitis Mephitis | Digastric | Fascicle Length (mm) | 19 | 22 | 33.90 | 33.70 |  | 0.95 | 38.7 | 0.347 |
| Mustela Nivalis | Deep masseter | Fascicle Length (mm) | 20 | 17 | 5.93 | 4.14 |  | 6.80 | 31.1 | 0.000 |
| Mustela Nivalis | Deep temporalis | Fascicle Length (mm) | 36 | 15 | 21.52 | 20.67 |  | 1.16 | 42.5 | 0.251 |
| Mustela Nivalis | Lateral pterygoid | Fascicle Length (mm) | 13 | 15 | 2.74 | 2.34 |  | 3.20 | 25.1 | 0.004 |
| Mustela Nivalis | Medial pterygoid | Fascicle Length (mm) | 27 | 19 | 6.23 | 1.69 |  | 15.62 | 42.5 | 0.000 |
| Mustela Nivalis | Superficial masseter | Fascicle Length (mm) | 30 | 19 | 6.03 | 5.35 |  | 1.50 | 43.3 | 0.141 |
| Mustela Nivalis | Superficial temporalis | Fascicle Length (mm) | 28 | 20 | 11.47 | 7.21 |  | 5.91 | 27.6 | 0.000 |
| Mustela Nivalis | Digastric | Fascicle Length (mm) | 27 | 12 | 17.70 | 12.93 |  | 5.05 | 17 | 0.000 |
| Otolemur Crassicaudatus | Deep masseter | Fascicle Length (mm) | 25 | 36 | 14.53 | 16.55 |  | -1.85 | 41.8 | 0.071 |
| Otolemur Crassicaudatus | Deep temporalis | Fascicle Length (mm) | 14 | 46 | 13.96 | 17.77 |  | -2.19 | 24.3 | 0.038 |
| Otolemur Crassicaudatus | Lateral pterygoid | Fascicle Length (mm) | 17 | 26 | 15.87 | 15.16 |  | 0.86 | 35.6 | 0.394 |
| Otolemur Crassicaudatus | Medial pterygoid | Fascicle Length (mm) | 22 | 36 | 9.52 | 8.08 |  | 1.70 | 32.6 | 0.099 |
| Otolemur Crassicaudatus | Superficial masseter | Fascicle Length (mm) | 19 | 35 | 21.31 | 28.32 |  | -5.87 | 42.7 | 0.000 |
| Otolemur Crassicaudatus | Superficial temporalis | Fascicle Length (mm) | 28 | 34 | 16.23 | 20.30 |  | -1.92 | 55.3 | 0.060 |
| Otolemur Crassicaudatus | Zygomaticomandibularis | Fascicle Length (mm) | 22 | 43 | 9.52 | 13.02 |  | -3.93 | 38.4 | 0.000 |
| Otolemur Crassicaudatus | Anterior digastric | Fascicle Length (mm) | 16 | 28 | 13.90 | 18.93 |  | -4.51 | 20.1 | 0.000 |
| Otolemur Crassicaudatus | Posterior digastric | Fascicle Length (mm) | 20 | 17 | 24.02 | 27.01 |  | -4.23 | 33.9 | 0.000 |
| Otolemur Crassicaudatus | Zygomatic temporalis | Fascicle Length (mm) | 22 | 33 | 20.22 | 17.97 |  | 1.85 | 51 | 0.070 |
| Procyon Lotor | Deep masseter | Fascicle Length (mm) | 43 | 52 | 27.13 | 27.66 |  | -1.16 | 92.1 | 0.247 |
| Procyon Lotor | Deep temporalis | Fascicle Length (mm) | 45 | 70 | 76.85 | 74.58 |  | 2.39 | 100.1 | 0.019 |
| Procyon Lotor | Lateral pterygoid | Fascicle Length (mm) | 28 | 40 | 16.09 | 15.72 |  | 1.06 | 54.1 | 0.296 |
| Procyon Lotor | Medial pterygoid | Fascicle Length (mm) | 24 | 52 | 23.60 | 30.10 |  | -3.67 | 47.8 | 0.001 |
| Procyon Lotor | Superficial masseter | Fascicle Length (mm) | 41 | 38 | 15.23 | 22.00 |  | -4.18 | 61.4 | 0.000 |
| Procyon Lotor | Superficial temporalis | Fascicle Length (mm) | 34 | 50 | 61.01 | 74.52 |  | -8.06 | 49 | 0.000 |
| Procyon Lotor | Zygomaticomandibularis | Fascicle Length (mm) | 38 | 43 | 21.03 | 22.86 |  | -1.32 | 74.8 | 0.190 |
| Procyon Lotor | Digastric | Fascicle Length (mm) | 28 | 36 | 55.48 | 54.88 |  | 1.84 | 61.6 | 0.071 |
| Daubentonia Madagascariensis | Deep masseter | Tortuosity | 29 | 33 | 1.01 | 1.02 |  | -3.96 | 43.3 | 0.000 |
| Daubentonia Madagascariensis | Deep temporalis | Tortuosity | 44 | 44 | 1.04 | 1.08 |  | -4.33 | 64.3 | 0.000 |
| Daubentonia Madagascariensis | Lateral pterygoid | Tortuosity | 18 | 21 | 1.04 | 1.05 |  | -1.13 | 35.1 | 0.266 |
| Daubentonia Madagascariensis | Medial pterygoid | Tortuosity | 34 | 34 | 1.02 | 1.02 |  | 2.27 | 50.8 | 0.027 |
| Daubentonia Madagascariensis | Superficial masseter | Tortuosity | 39 | 46 | 1.02 | 1.03 |  | -2.89 | 74.8 | 0.005 |
| Daubentonia Madagascariensis | Superficial temporalis | Tortuosity | 35 | 47 | 1.06 | 1.12 |  | -4.50 | 79.3 | 0.000 |
| Daubentonia Madagascariensis | Zygomaticomandibularis | Tortuosity | 20 | 20 | 1.02 | 1.02 |  | 0.02 | 35.3 | 0.987 |
| Eulemur Coronatus | Deep masseter | Tortuosity | 22 | 25 | 1.15 | 1.04 |  | 2.72 | 21.4 | 0.013 |
| Eulemur Coronatus | Deep temporalis | Tortuosity | 24 | 32 | 1.10 | 1.10 |  | -0.11 | 44.9 | 0.910 |
| Eulemur Coronatus | Lateral pterygoid | Tortuosity | 22 | 19 | 1.03 | 1.03 |  | -0.56 | 30.3 | 0.582 |
| Eulemur Coronatus | Medial pterygoid | Tortuosity | 22 | 36 | 1.09 | 1.08 |  | 0.48 | 36.6 | 0.638 |
| Eulemur Coronatus | Superficial masseter | Tortuosity | 26 | 26 | 1.08 | 1.15 |  | -2.74 | 38.64 | 0.009 |
| Eulemur Coronatus | Superficial temporalis | Tortuosity | 20 | 24 | 1.06 | 1.06 |  | -0.63 | 40.1 | 0.534 |
| Eulemur Coronatus | Zygomaticomandibularis | Tortuosity | 26 | 24 | 1.04 | 1.04 |  | -0.12 | 42.1 | 0.904 |
| Eulemur Coronatus | Anterior digastric | Tortuosity | 12 | 22 | 1.01 | 1.01 |  | 0.63 | 17.2 | 0.539 |
| Eulemur Coronatus | Posterior digastric | Tortuosity | 19 | 21 | 1.01 | 1.01 |  | 0.81 | 28.6 | 0.424 |
| Eulemur Coronatus | Zygomatic temporalis | Tortuosity | 18 | 18 | 1.07 | 1.05 |  | 1.01 | 27.7 | 0.322 |
| Lontra Canadensis | Deep masseter | Tortuosity | 17 | 23 | 1.03 | 1.02 |  | 1.53 | 24.2 | 0.138 |
| Lontra Canadensis | Deep temporalis | Tortuosity | 40 | 40 | 1.07 | 1.10 |  | -1.58 | 75.9 | 0.118 |
| Lontra Canadensis | Lateral pterygoid | Tortuosity | 14 | 14 | 1.13 | 1.12 |  | 0.26 | 21.9 | 0.801 |
| Lontra Canadensis | Medial pterygoid | Tortuosity | 34 | 52 | 1.05 | 1.08 |  | -2.83 | 78.8 | 0.006 |
| Lontra Canadensis | Superficial masseter | Tortuosity | 24 | 66 | 1.04 | 1.09 |  | -4.68 | 88 | 0.000 |
| Lontra Canadensis | Superficial temporalis | Tortuosity | 41 | 43 | 1.05 | 1.08 |  | -3.52 | 81.5 | 0.001 |
| Lontra Canadensis | Zygomaticomandibularis | Tortuosity | 10 | 29 | 1.02 | 1.17 |  | -4.87 | 32.8 | 0.000 |
| Lontra Canadensis | Digastric | Tortuosity | 44 | 39 | 1.01 | 1.03 |  | -4.35 | 52 | 0.000 |
| Martes Americana | Deep masseter | Tortuosity | 24 | 28 | 1.04 | 1.02 |  | 1.94 | 30.5 | 0.061 |
| Martes Americana | Deep temporalis | Tortuosity | 35 | 67 | 1.11 | 1.12 |  | -0.87 | 87 | 0.387 |
| Martes Americana | Lateral pterygoid | Tortuosity | 12 | 9 | 1.04 | 1.03 |  | 1.01 | 18.3 | 0.327 |
| Martes Americana | Medial pterygoid | Tortuosity | 28 | 39 | 1.03 | 1.06 |  | -3.05 | 52.7 | 0.004 |
| Martes Americana | Superficial masseter | Tortuosity | 31 | 59 | 1.06 | 1.04 |  | 1.67 | 40.6 | 0.102 |
| Martes Americana | Superficial temporalis | Tortuosity | 36 | 49 | 1.11 | 1.13 |  | -1.06 | 68.9 | 0.294 |
| Martes Americana | Zygomaticomandibularis | Tortuosity | 17 | 36 | 1.02 | 1.02 |  | -0.62 | 32.5 | 0.543 |
| Martes Americana | Digastric | Tortuosity | 37 | 37 | 1.02 | 1.02 |  | 0.00 | 72 | 1.000 |
| Mephitis Mephitis | Deep masseter | Tortuosity | 16 | 25 | 1.03 | 1.03 |  | -0.32 | 38.1 | 0.748 |
| Mephitis Mephitis | Deep temporalis | Tortuosity | 30 | 36 | 1.07 | 1.06 |  | 1.49 | 64 | 0.141 |
| Mephitis Mephitis | Lateral pterygoid | Tortuosity | 7 | 10 | 1.20 | 1.24 |  | -0.44 | 13.3 | 0.668 |
| Mephitis Mephitis | Medial pterygoid | Tortuosity | 19 | 28 | 1.07 | 1.04 |  | 1.40 | 22.3 | 0.177 |
| Mephitis Mephitis | Superficial masseter | Tortuosity | 24 | 41 | 1.15 | 1.13 |  | 0.83 | 41.7 | 0.411 |
| Mephitis Mephitis | Superficial temporalis | Tortuosity | 29 | 35 | 1.09 | 1.12 |  | -2.05 | 60.8 | 0.045 |
| Mephitis Mephitis | Zygomaticomandibularis | Tortuosity | 25 | 44 | 1.06 | 1.05 |  | 0.69 | 35.6 | 0.494 |
| Mephitis Mephitis | Digastric | Tortuosity | 19 | 22 | 1.02 | 1.02 |  | -0.34 | 38.8 | 0.736 |
| Mustela Nivalis | Deep masseter | Tortuosity | 20 | 17 | 1.04 | 1.05 |  | -0.64 | 25.1 | 0.527 |
| Mustela Nivalis | Deep temporalis | Tortuosity | 36 | 15 | 1.05 | 1.04 |  | 2.28 | 30.1 | 0.030 |
| Mustela Nivalis | Lateral pterygoid | Tortuosity | 13 | 15 | 1.08 | 1.11 |  | -0.87 | 19.9 | 0.394 |
| Mustela Nivalis | Medial pterygoid | Tortuosity | 27 | 19 | 1.04 | 1.11 |  | -2.84 | 21.4 | 0.010 |
| Mustela Nivalis | Superficial masseter | Tortuosity | 30 | 19 | 1.05 | 1.01 |  | 4.85 | 37.9 | 0.000 |
| Mustela Nivalis | Superficial temporalis | Tortuosity | 28 | 20 | 1.02 | 1.03 |  | -2.05 | 32.2 | 0.048 |
| Mustela Nivalis | Digastric | Tortuosity | 27 | 12 | 1.01 | 1.01 |  | 2.07 | 24.8 | 0.049 |
| Otolemur Crassicaudatus | Deep masseter | Tortuosity | 25 | 36 | 1.01 | 1.01 |  | -1.51 | 58.5 | 0.137 |
| Otolemur Crassicaudatus | Deep temporalis | Tortuosity | 14 | 46 | 1.05 | 1.06 |  | -1.08 | 20.4 | 0.292 |
| Otolemur Crassicaudatus | Lateral pterygoid | Tortuosity | 17 | 26 | 1.04 | 1.05 |  | -0.51 | 31 | 0.612 |
| Otolemur Crassicaudatus | Medial pterygoid | Tortuosity | 22 | 36 | 1.02 | 1.19 |  | -6.20 | 35.7 | 0.000 |
| Otolemur Crassicaudatus | Superficial masseter | Tortuosity | 19 | 35 | 1.04 | 1.05 |  | -1.05 | 41.1 | 0.301 |
| Otolemur Crassicaudatus | Superficial temporalis | Tortuosity | 28 | 34 | 1.06 | 1.08 |  | -1.31 | 60 | 0.196 |
| Otolemur Crassicaudatus | Zygomaticomandibularis | Tortuosity | 22 | 43 | 1.02 | 1.11 |  | -7.39 | 46.9 | 0.000 |
| Otolemur Crassicaudatus | Anterior digastric | Tortuosity | 16 | 28 | 1.02 | 1.03 |  | -1.86 | 24.2 | 0.076 |
| Otolemur Crassicaudatus | Posterior digastric | Tortuosity | 20 | 17 | 1.01 | 1.02 |  | -2.23 | 24.8 | 0.035 |
| Otolemur Crassicaudatus | Zygomatic temporalis | Tortuosity | 22 | 33 | 1.12 | 1.10 |  | 1.67 | 53 | 0.102 |
| Procyon Lotor | Deep masseter | Tortuosity | 43 | 52 | 1.02 | 1.02 |  | -1.36 | 92.1 | 0.178 |
| Procyon Lotor | Deep temporalis | Tortuosity | 45 | 70 | 1.22 | 1.18 |  | 2.97 | 100.4 | 0.004 |
| Procyon Lotor | Lateral pterygoid | Tortuosity | 28 | 40 | 1.08 | 1.07 |  | 0.58 | 53.2 | 0.566 |
| Procyon Lotor | Medial pterygoid | Tortuosity | 24 | 52 | 1.01 | 1.03 |  | -5.78 | 59.1 | 0.000 |
| Procyon Lotor | Superficial masseter | Tortuosity | 41 | 38 | 1.05 | 1.04 |  | 1.25 | 76.6 | 0.216 |
| Procyon Lotor | Superficial temporalis | Tortuosity | 34 | 50 | 1.13 | 1.16 |  | -4.22 | 80 | 0.000 |
| Procyon Lotor | Zygomaticomandibularis | Tortuosity | 38 | 43 | 1.04 | 1.07 |  | -3.51 | 66.7 | 0.001 |
| Procyon Lotor | Digastric | Tortuosity | 28 | 36 | 1.02 | 1.01 |  | 2.13 | 42.7 | 0.039 |

**Supplemental Table 3.** Means and standard deviations of fascicle length and tortuosity for the various reconstruction methods assessed in this study. Gross dissection values for primates were collected from Dickinson et al. (2024) and carnivores from Hartstone-Rose et al. (2022). Note that two major differences exist within the gross dissection data collected from primates and carnivores. First, a zygomatic portion of the temporalis was observed within primates, but not carnivores. Secondly, data from the masseteric and temporalis complex were aggregated together within carnivores (i.e., presented at the “masseter” and “temporalis” levels); whereas data were collected for each individual component (e.g., superficial masseter, deep masseter, zygomaticomandibularis, superficial temporalis, and deep temporalis) in primates, allowing for more direct comparison of muscle architectural properties between individual muscle layers within these species.

| Species | Muscle | Method | N | Mean_Fascicle_Length | SD_Fascicle_Length | Mean_Tortuosity | SD_Tortuosity |
| --- | --- | --- | --- | --- | --- | --- | --- |
| Daubentonia madagascariensis | Deep masseter | GoodFibes | 29 | 8.55 | 3.1 | 1.01 | 0.01 |
| Daubentonia madagascariensis | Deep masseter | Gross Dissection | 1 | 8.27 |  |  |  |
| Daubentonia madagascariensis | Deep masseter | Manual Segmentation | 14 | 10.48 | 2.53 | 1.05 | 0.03 |
| Daubentonia madagascariensis | Deep masseter | XFIBER | 64 | 8.47 | 3.02 | 1.01 | 0.01 |
| Daubentonia madagascariensis | Deep temporalis | GoodFibes | 44 | 25.04 | 11.48 | 1.04 | 0.03 |
| Daubentonia madagascariensis | Deep temporalis | Gross Dissection | 1 | 14.74 |  |  |  |
| Daubentonia madagascariensis | Deep temporalis | Manual Segmentation | 26 | 13.24 | 6.4 | 1.12 | 0.08 |
| Daubentonia madagascariensis | Deep temporalis | XFIBER | 93 | 13.34 | 5.15 | 1.01 | 0.02 |
| Daubentonia madagascariensis | Lateral pterygoid | GoodFibes | 18 | 11.13 | 5.01 | 1.04 | 0.04 |
| Daubentonia madagascariensis | Lateral pterygoid | Manual Segmentation | 12 | 11.7 | 4.04 | 1.04 | 0.03 |
| Daubentonia madagascariensis | Lateral pterygoid | XFIBER | 52 | 11.01 | 4.29 | 1.02 | 0.01 |
| Daubentonia madagascariensis | Medial pterygoid | GoodFibes | 34 | 21.17 | 9.4 | 1.02 | 0.02 |
| Daubentonia madagascariensis | Medial pterygoid | Gross Dissection | 1 | 5.9 |  |  |  |
| Daubentonia madagascariensis | Medial pterygoid | Manual Segmentation | 12 | 11.55 | 2.32 | 1.07 | 0.04 |
| Daubentonia madagascariensis | Medial pterygoid | XFIBER | 55 | 8.78 | 3.26 | 1.01 | 0.01 |
| Daubentonia madagascariensis | Superficial masseter | GoodFibes | 39 | 10.09 | 4.35 | 1.02 | 0.02 |
| Daubentonia madagascariensis | Superficial masseter | Gross Dissection | 1 | 8.42 |  |  |  |
| Daubentonia madagascariensis | Superficial masseter | Manual Segmentation | 16 | 12.53 | 2.75 | 1.07 | 0.03 |
| Daubentonia madagascariensis | Superficial masseter | XFIBER | 60 | 8.9 | 2.97 | 1.02 | 0.04 |
| Daubentonia madagascariensis | Superficial temporalis | GoodFibes | 35 | 25.61 | 22.8 | 1.06 | 0.05 |
| Daubentonia madagascariensis | Superficial temporalis | Gross Dissection | 1 | 12.96 |  |  |  |
| Daubentonia madagascariensis | Superficial temporalis | Manual Segmentation | 28 | 16.88 | 6.59 | 1.11 | 0.09 |
| Daubentonia madagascariensis | Superficial temporalis | XFIBER | 95 | 12.97 | 6.29 | 1.02 | 0.04 |
| Daubentonia madagascariensis | Zygomaticomandibularis | GoodFibes | 20 | 10.61 | 3.89 | 1.02 | 0.01 |
| Daubentonia madagascariensis | Zygomaticomandibularis | Gross Dissection | 1 | 9.26 |  |  |  |
| Daubentonia madagascariensis | Zygomaticomandibularis | Manual Segmentation | 18 | 10.38 | 3.01 | 1.07 | 0.07 |
| Daubentonia madagascariensis | Zygomaticomandibularis | XFIBER | 43 | 9.93 | 3.31 | 1.02 | 0.02 |
| Eulemur coronatus | Anterior digastric | GoodFibes | 12 | 22.36 | 1.49 | 1.01 | 0.01 |
| Eulemur coronatus | Anterior digastric | Manual Segmentation | 10 | 8.76 | 4.01 | 1.05 | 0.04 |
| Eulemur coronatus | Anterior digastric | XFIBER | 41 | 10.47 | 4.55 | 1.01 | 0.01 |
| Eulemur coronatus | Deep masseter | GoodFibes | 22 | 16.71 | 4.98 | 1.15 | 0.19 |
| Eulemur coronatus | Deep masseter | Gross Dissection | 1 | 7.8 |  |  |  |
| Eulemur coronatus | Deep masseter | Manual Segmentation | 10 | 6.87 | 1.88 | 1.07 | 0.04 |
| Eulemur coronatus | Deep masseter | XFIBER | 52 | 8.26 | 2.88 | 1.04 | 0.07 |
| Eulemur coronatus | Deep temporalis | GoodFibes | 24 | 34.77 | 4.92 | 1.1 | 0.04 |
| Eulemur coronatus | Deep temporalis | Gross Dissection | 1 | 9.6 |  |  |  |
| Eulemur coronatus | Deep temporalis | Manual Segmentation | 26 | 10.87 | 5.7 | 1.09 | 0.08 |
| Eulemur coronatus | Deep temporalis | XFIBER | 58 | 13.52 | 7.75 | 1.04 | 0.05 |
| Eulemur coronatus | Lateral pterygoid | GoodFibes | 22 | 15.06 | 1.27 | 1.03 | 0.02 |
| Eulemur coronatus | Lateral pterygoid | Manual Segmentation | 10 | 7.08 | 1.45 | 1.04 | 0.03 |
| Eulemur coronatus | Lateral pterygoid | XFIBER | 33 | 7.5 | 2.11 | 1.02 | 0.03 |
| Eulemur coronatus | Medial pterygoid | GoodFibes | 22 | 6.37 | 2.62 | 1.09 | 0.08 |
| Eulemur coronatus | Medial pterygoid | Gross Dissection | 1 | 5.8 |  |  |  |
| Eulemur coronatus | Medial pterygoid | Manual Segmentation | 13 | 6.59 | 2.86 | 1.09 | 0.04 |
| Eulemur coronatus | Medial pterygoid | XFIBER | 46 | 9.08 | 2.53 | 1.01 | 0.01 |
| Eulemur coronatus | Posterior digastric | GoodFibes | 19 | 21.57 | 3.56 | 1.01 | 0.01 |
| Eulemur coronatus | Posterior digastric | Manual Segmentation | 13 | 10.27 | 5.01 | 1.05 | 0.03 |
| Eulemur coronatus | Posterior digastric | XFIBER | 30 | 9.8 | 3.97 | 1.01 | 0.01 |
| Eulemur coronatus | Superficial masseter | GoodFibes | 26 | 17.91 | 7.57 | 1.08 | 0.07 |
| Eulemur coronatus | Superficial masseter | Gross Dissection | 1 | 8.2 |  |  |  |
| Eulemur coronatus | Superficial masseter | Manual Segmentation | 17 | 8.91 | 2.5 | 1.04 | 0.04 |
| Eulemur coronatus | Superficial masseter | XFIBER | 62 | 9.36 | 3.13 | 1.02 | 0.02 |
| Eulemur coronatus | Superficial temporalis | GoodFibes | 20 | 25.65 | 2.86 | 1.06 | 0.03 |
| Eulemur coronatus | Superficial temporalis | Gross Dissection | 1 | 11.2 |  |  |  |
| Eulemur coronatus | Superficial temporalis | Manual Segmentation | 26 | 12.6 | 4.61 | 1.09 | 0.05 |
| Eulemur coronatus | Superficial temporalis | XFIBER | 30 | 11.83 | 4.2 | 1.03 | 0.03 |
| Eulemur coronatus | Zygomatic temporalis | GoodFibes | 18 | 14.59 | 2.36 | 1.07 | 0.06 |
| Eulemur coronatus | Zygomatic temporalis | Gross Dissection | 1 | 7.2 |  |  |  |
| Eulemur coronatus | Zygomatic temporalis | Manual Segmentation | 18 | 7.86 | 3.06 | 1.1 | 0.06 |
| Eulemur coronatus | Zygomatic temporalis | XFIBER | 41 | 9.4 | 3.19 | 1.04 | 0.04 |
| Eulemur coronatus | Zygomaticomandibularis | GoodFibes | 26 | 11.08 | 3.02 | 1.04 | 0.02 |
| Eulemur coronatus | Zygomaticomandibularis | Gross Dissection | 1 | 6.2 |  |  |  |
| Eulemur coronatus | Zygomaticomandibularis | Manual Segmentation | 9 | 5.95 | 1.37 | 1.09 | 0.04 |
| Eulemur coronatus | Zygomaticomandibularis | XFIBER | 44 | 6.35 | 1.93 | 1.02 | 0.02 |
| Lontra canadensis | Deep masseter | GoodFibes | 17 | 7.98 | 2.89 | 1.03 | 0.02 |
| Lontra canadensis | Deep masseter | Gross Dissection | 1 | 11.66 |  |  |  |
| Lontra canadensis | Deep masseter | Manual Segmentation | 13 | 10.07 | 1.83 | 1.04 | 0.02 |
| Lontra canadensis | Deep masseter | XFIBER | 60 | 9.06 | 3.1 | 1.02 | 0.02 |
| Lontra canadensis | Deep temporalis | GoodFibes | 40 | 37.53 | 14.23 | 1.07 | 0.06 |
| Lontra canadensis | Deep temporalis | Gross Dissection | 1 | 18.74 |  |  |  |
| Lontra canadensis | Deep temporalis | Manual Segmentation | 28 | 21.19 | 8.65 | 1.11 | 0.04 |
| Lontra canadensis | Deep temporalis | XFIBER | 66 | 17.75 | 5.61 | 1.01 | 0.02 |
| Lontra canadensis | Digastric | GoodFibes | 44 | 41.85 | 6.7 | 1.01 | 0.01 |
| Lontra canadensis | Digastric | Manual Segmentation | 14 | 35.09 | 6.19 | 1.03 | 0.02 |
| Lontra canadensis | Digastric | XFIBER | 56 | 24.04 | 6.63 | 1.01 | 0.01 |
| Lontra canadensis | Lateral pterygoid | GoodFibes | 14 | 12.2 | 1.38 | 1.13 | 0.14 |
| Lontra canadensis | Lateral pterygoid | Manual Segmentation | 7 | 6.25 | 2.03 | 1.02 | 0.01 |
| Lontra canadensis | Lateral pterygoid | XFIBER | 36 | 5.45 | 1.12 | 1.02 | 0.03 |
| Lontra canadensis | Medial pterygoid | GoodFibes | 34 | 14.28 | 4.1 | 1.05 | 0.03 |
| Lontra canadensis | Medial pterygoid | Gross Dissection | 1 | 8.2 |  |  |  |
| Lontra canadensis | Medial pterygoid | Manual Segmentation | 12 | 9.45 | 1.35 | 1.05 | 0.03 |
| Lontra canadensis | Medial pterygoid | XFIBER | 44 | 11.56 | 4.26 | 1.02 | 0.03 |
| Lontra canadensis | Superficial masseter | GoodFibes | 24 | 18.11 | 3.27 | 1.03 | 0.03 |
| Lontra canadensis | Superficial masseter | Gross Dissection | 1 | 11.66 |  |  |  |
| Lontra canadensis | Superficial masseter | Manual Segmentation | 26 | 14.16 | 3.59 | 1.06 | 0.05 |
| Lontra canadensis | Superficial masseter | XFIBER | 64 | 12.01 | 4.38 | 1.02 | 0.02 |
| Lontra canadensis | Superficial temporalis | GoodFibes | 41 | 34.16 | 16.39 | 1.05 | 0.04 |
| Lontra canadensis | Superficial temporalis | Gross Dissection | 1 | 18.74 |  |  |  |
| Lontra canadensis | Superficial temporalis | Manual Segmentation | 57 | 16.35 | 9.11 | 1.08 | 0.05 |
| Lontra canadensis | Superficial temporalis | XFIBER | 72 | 17.64 | 7.24 | 1.02 | 0.02 |
| Lontra canadensis | Zygomaticomandibularis | GoodFibes | 10 | 5 | 1.7 | 1.02 | 0.03 |
| Lontra canadensis | Zygomaticomandibularis | Gross Dissection | 1 | 11.66 |  |  |  |
| Lontra canadensis | Zygomaticomandibularis | Manual Segmentation | 16 | 8.78 | 3.31 | 1.09 | 0.05 |
| Lontra canadensis | Zygomaticomandibularis | XFIBER | 52 | 10.12 | 2.93 | 1.02 | 0.02 |
| Martes americana | Deep masseter | GoodFibes | 24 | 18.72 | 4.42 | 1.04 | 0.03 |
| Martes americana | Deep masseter | Gross Dissection | 1 | 8.09 |  |  |  |
| Martes americana | Deep masseter | Manual Segmentation | 16 | 8.61 | 3.37 | 1.03 | 0.02 |
| Martes americana | Deep masseter | XFIBER | 36 | 10.32 | 3.5 | 1.03 | 0.04 |
| Martes americana | Deep temporalis | GoodFibes | 35 | 33.35 | 9.95 | 1.11 | 0.04 |
| Martes americana | Deep temporalis | Gross Dissection | 1 | 10.7 |  |  |  |
| Martes americana | Deep temporalis | Manual Segmentation | 24 | 17.73 | 5.51 | 1.04 | 0.02 |
| Martes americana | Deep temporalis | XFIBER | 64 | 14.5 | 6.09 | 1.01 | 0.01 |
| Martes americana | Digastric | GoodFibes | 37 | 39.8 | 1.55 | 1.02 | 0.01 |
| Martes americana | Digastric | Manual Segmentation | 22 | 24.59 | 10.01 | 1.03 | 0.01 |
| Martes americana | Digastric | XFIBER | 43 | 17.95 | 5.45 | 1 | 0 |
| Martes americana | Lateral pterygoid | GoodFibes | 12 | 9.54 | 1.96 | 1.04 | 0.03 |
| Martes americana | Lateral pterygoid | Manual Segmentation | 12 | 5.85 | 1.84 | 1.03 | 0.01 |
| Martes americana | Lateral pterygoid | XFIBER | 28 | 4.27 | 1.84 | 1.02 | 0.02 |
| Martes americana | Medial pterygoid | GoodFibes | 28 | 11.5 | 3.91 | 1.03 | 0.02 |
| Martes americana | Medial pterygoid | Gross Dissection | 1 | 5.27 |  |  |  |
| Martes americana | Medial pterygoid | Manual Segmentation | 28 | 11.05 | 4.36 | 1.04 | 0.02 |
| Martes americana | Medial pterygoid | XFIBER | 36 | 13.04 | 4.35 | 1.04 | 0.03 |
| Martes americana | Superficial masseter | GoodFibes | 31 | 20.45 | 4.97 | 1.06 | 0.07 |
| Martes americana | Superficial masseter | Gross Dissection | 1 | 8.09 |  |  |  |
| Martes americana | Superficial masseter | Manual Segmentation | 34 | 11.54 | 3.09 | 1.06 | 0.03 |
| Martes americana | Superficial masseter | XFIBER | 67 | 7.92 | 2.73 | 1 | 0 |
| Martes americana | Superficial temporalis | GoodFibes | 36 | 31.13 | 11.99 | 1.11 | 0.07 |
| Martes americana | Superficial temporalis | Gross Dissection | 1 | 10.7 |  |  |  |
| Martes americana | Superficial temporalis | Manual Segmentation | 33 | 19.9 | 6.96 | 1.06 | 0.03 |
| Martes americana | Superficial temporalis | XFIBER | 54 | 11.19 | 5.13 | 1.01 | 0.02 |
| Martes americana | Zygomaticomandibularis | GoodFibes | 17 | 19.83 | 4.8 | 1.02 | 0.02 |
| Martes americana | Zygomaticomandibularis | Gross Dissection | 1 | 8.09 |  |  |  |
| Martes americana | Zygomaticomandibularis | Manual Segmentation | 15 | 9.51 | 1.72 | 1.05 | 0.02 |
| Martes americana | Zygomaticomandibularis | XFIBER | 39 | 10.64 | 3.41 | 1.02 | 0.02 |
| Mephitis mephitis | Deep masseter | GoodFibes | 16 | 8.37 | 2.45 | 1.03 | 0.02 |
| Mephitis mephitis | Deep masseter | Gross Dissection | 1 | 5.83 |  |  |  |
| Mephitis mephitis | Deep masseter | Manual Segmentation | 13 | 7.11 | 2.16 | 1.05 | 0.02 |
| Mephitis mephitis | Deep masseter | XFIBER | 60 | 6.36 | 0.93 | 1.02 | 0.03 |
| Mephitis mephitis | Deep temporalis | GoodFibes | 30 | 38.11 | 2.77 | 1.07 | 0.02 |
| Mephitis mephitis | Deep temporalis | Gross Dissection | 1 | 9.53 |  |  |  |
| Mephitis mephitis | Deep temporalis | Manual Segmentation | 32 | 10.74 | 3.3 | 1.08 | 0.03 |
| Mephitis mephitis | Deep temporalis | XFIBER | 59 | 14.63 | 3.88 | 1.02 | 0.01 |
| Mephitis mephitis | Digastric | GoodFibes | 19 | 33.9 | 0.57 | 1.02 | 0.01 |
| Mephitis mephitis | Digastric | Manual Segmentation | 13 | 6.28 | 1.73 | 1.01 | 0.01 |
| Mephitis mephitis | Digastric | XFIBER | 54 | 11.84 | 5.64 | 1.01 | 0.01 |
| Mephitis mephitis | Lateral pterygoid | GoodFibes | 7 | 3.83 | 1.04 | 1.2 | 0.2 |
| Mephitis mephitis | Lateral pterygoid | Manual Segmentation | 7 | 3.3 | 0.59 | 1.03 | 0.01 |
| Mephitis mephitis | Lateral pterygoid | XFIBER | 39 | 3.85 | 0.87 | 1.01 | 0.01 |
| Mephitis mephitis | Medial pterygoid | GoodFibes | 19 | 10.2 | 2.18 | 1.07 | 0.08 |
| Mephitis mephitis | Medial pterygoid | Gross Dissection | 1 | 4.86 |  |  |  |
| Mephitis mephitis | Medial pterygoid | Manual Segmentation | 14 | 5.25 | 1.92 | 1.04 | 0.04 |
| Mephitis mephitis | Medial pterygoid | XFIBER | 34 | 7.36 | 1.59 | 1.02 | 0.02 |
| Mephitis mephitis | Superficial masseter | GoodFibes | 24 | 12.44 | 3.76 | 1.15 | 0.12 |
| Mephitis mephitis | Superficial masseter | Gross Dissection | 1 | 5.83 |  |  |  |
| Mephitis mephitis | Superficial masseter | Manual Segmentation | 21 | 8.11 | 2.65 | 1.06 | 0.07 |
| Mephitis mephitis | Superficial masseter | XFIBER | 57 | 6.58 | 1.63 | 1.01 | 0.01 |
| Mephitis mephitis | Superficial temporalis | GoodFibes | 29 | 41.03 | 8.79 | 1.09 | 0.04 |
| Mephitis mephitis | Superficial temporalis | Gross Dissection | 1 | 9.53 |  |  |  |
| Mephitis mephitis | Superficial temporalis | Manual Segmentation | 26 | 11.55 | 2.71 | 1.07 | 0.06 |
| Mephitis mephitis | Superficial temporalis | XFIBER | 65 | 9.15 | 3.52 | 1.01 | 0.01 |
| Mephitis mephitis | Zygomaticomandibularis | GoodFibes | 25 | 9.04 | 2.61 | 1.06 | 0.06 |
| Mephitis mephitis | Zygomaticomandibularis | Gross Dissection | 1 | 5.83 |  |  |  |
| Mephitis mephitis | Zygomaticomandibularis | Manual Segmentation | 16 | 4.11 | 1.67 | 1.05 | 0.01 |
| Mephitis mephitis | Zygomaticomandibularis | XFIBER | 55 | 5.64 | 1.58 | 1.01 | 0.01 |
| Mustela nivalis | Deep masseter | GoodFibes | 20 | 5.93 | 1 | 1.04 | 0.03 |
| Mustela nivalis | Deep masseter | Gross Dissection | 1 | 3.3 |  |  |  |
| Mustela nivalis | Deep masseter | Manual Segmentation | 8 | 2.72 | 0.51 | 1.06 | 0.01 |
| Mustela nivalis | Deep masseter | XFIBER | 30 | 3.4 | 0.68 | 1.01 | 0.02 |
| Mustela nivalis | Deep temporalis | GoodFibes | 36 | 21.52 | 3.22 | 1.05 | 0.02 |
| Mustela nivalis | Deep temporalis | Gross Dissection | 1 | 7.6 |  |  |  |
| Mustela nivalis | Deep temporalis | Manual Segmentation | 24 | 3.08 | 0.42 | 1.12 | 0.03 |
| Mustela nivalis | Deep temporalis | XFIBER | 62 | 6.74 | 2.95 | 1.01 | 0.01 |
| Mustela nivalis | Digastric | GoodFibes | 27 | 17.7 | 2.24 | 1.01 | 0 |
| Mustela nivalis | Digastric | Manual Segmentation | 10 | 4.49 | 1.75 | 1.02 | 0.01 |
| Mustela nivalis | Digastric | XFIBER | 47 | 8.74 | 4.34 | 1.01 | 0.01 |
| Mustela nivalis | Lateral pterygoid | GoodFibes | 13 | 2.74 | 0.28 | 1.08 | 0.05 |
| Mustela nivalis | Lateral pterygoid | Manual Segmentation | 5 | 1.6 | 0.21 | 1.01 | 0.01 |
| Mustela nivalis | Lateral pterygoid | XFIBER | 41 | 2.14 | 0.52 | 1.01 | 0.01 |
| Mustela nivalis | Medial pterygoid | GoodFibes | 27 | 6.23 | 1.07 | 1.04 | 0.04 |
| Mustela nivalis | Medial pterygoid | Gross Dissection | 1 | 4.1 |  |  |  |
| Mustela nivalis | Medial pterygoid | Manual Segmentation | 8 | 2.6 | 0.62 | 1.06 | 0.02 |
| Mustela nivalis | Medial pterygoid | XFIBER | 45 | 3.56 | 1.2 | 1.01 | 0.01 |
| Mustela nivalis | Superficial masseter | GoodFibes | 30 | 6.03 | 1.7 | 1.05 | 0.04 |
| Mustela nivalis | Superficial masseter | Gross Dissection | 1 | 3.3 |  |  |  |
| Mustela nivalis | Superficial masseter | Manual Segmentation | 9 | 3.09 | 0.67 | 1.05 | 0.03 |
| Mustela nivalis | Superficial masseter | XFIBER | 35 | 4.16 | 1.28 | 1.03 | 0.04 |
| Mustela nivalis | Superficial temporalis | GoodFibes | 28 | 11.47 | 1.64 | 1.02 | 0.01 |
| Mustela nivalis | Superficial temporalis | Gross Dissection | 1 | 7.6 |  |  |  |
| Mustela nivalis | Superficial temporalis | Manual Segmentation | 14 | 3.56 | 0.7 | 1.13 | 0.07 |
| Mustela nivalis | Superficial temporalis | XFIBER | 61 | 5.92 | 2.03 | 1.01 | 0.01 |
| Mustela nivalis | Zygomaticomandibularis | GoodFibes | 25 | 4.13 | 0.57 | 1.05 | 0.04 |
| Mustela nivalis | Zygomaticomandibularis | Gross Dissection | 1 | 3.3 |  |  |  |
| Mustela nivalis | Zygomaticomandibularis | Manual Segmentation | 8 | 2.73 | 0.71 | 1.07 | 0.02 |
| Mustela nivalis | Zygomaticomandibularis | XFIBER | 46 | 3.77 | 1 | 1.03 | 0.03 |
| Otolemur crassicaudatus | Anterior digastric | GoodFibes | 16 | 13.9 | 4.13 | 1.02 | 0.02 |
| Otolemur crassicaudatus | Anterior digastric | Manual Segmentation | 8 | 3.52 | 1.43 | 1.04 | 0.01 |
| Otolemur crassicaudatus | Anterior digastric | XFIBER | 34 | 7.65 | 3.63 | 1.02 | 0.02 |
| Otolemur crassicaudatus | Deep masseter | GoodFibes | 25 | 14.53 | 4.64 | 1.01 | 0.01 |
| Otolemur crassicaudatus | Deep masseter | Gross Dissection | 1 | 8.8 |  |  |  |
| Otolemur crassicaudatus | Deep masseter | Manual Segmentation | 8 | 6.88 | 0.8 | 1.05 | 0.03 |
| Otolemur crassicaudatus | Deep masseter | XFIBER | 43 | 7.97 | 1.97 | 1.01 | 0.01 |
| Otolemur crassicaudatus | Deep temporalis | GoodFibes | 14 | 13.96 | 5.49 | 1.05 | 0.04 |
| Otolemur crassicaudatus | Deep temporalis | Gross Dissection | 1 | 7.4 |  |  |  |
| Otolemur crassicaudatus | Deep temporalis | Manual Segmentation | 14 | 8.18 | 1.88 | 1.1 | 0.05 |
| Otolemur crassicaudatus | Deep temporalis | XFIBER | 74 | 9.21 | 5.58 | 1.01 | 0.02 |
| Otolemur crassicaudatus | Lateral pterygoid | GoodFibes | 17 | 15.87 | 2.6 | 1.04 | 0.04 |
| Otolemur crassicaudatus | Lateral pterygoid | Manual Segmentation | 15 | 4.11 | 1.47 | 1.04 | 0.03 |
| Otolemur crassicaudatus | Lateral pterygoid | XFIBER | 40 | 7.1 | 2.6 | 1.02 | 0.02 |
| Otolemur crassicaudatus | Medial pterygoid | GoodFibes | 22 | 9.52 | 3.52 | 1.02 | 0.01 |
| Otolemur crassicaudatus | Medial pterygoid | Gross Dissection | 1 | 5.7 |  |  |  |
| Otolemur crassicaudatus | Medial pterygoid | Manual Segmentation | 11 | 6.02 | 0.89 | 1.06 | 0.04 |
| Otolemur crassicaudatus | Medial pterygoid | XFIBER | 45 | 7.62 | 2.52 | 1.01 | 0.01 |
| Otolemur crassicaudatus | Posterior digastric | GoodFibes | 20 | 24.02 | 2.52 | 1.01 | 0.01 |
| Otolemur crassicaudatus | Posterior digastric | Manual Segmentation | 10 | 5.49 | 1.26 | 1.05 | 0.03 |
| Otolemur crassicaudatus | Posterior digastric | XFIBER | 37 | 8.9 | 4.93 | 1.02 | 0.02 |
| Otolemur crassicaudatus | Superficial masseter | GoodFibes | 19 | 21.31 | 3.92 | 1.04 | 0.03 |
| Otolemur crassicaudatus | Superficial masseter | Gross Dissection | 1 | 8.5 |  |  |  |
| Otolemur crassicaudatus | Superficial masseter | Manual Segmentation | 10 | 8.39 | 1.65 | 1.08 | 0.05 |
| Otolemur crassicaudatus | Superficial masseter | XFIBER | 56 | 9.33 | 2.79 | 1.01 | 0.01 |
| Otolemur crassicaudatus | Superficial temporalis | GoodFibes | 28 | 16.23 | 8.65 | 1.06 | 0.04 |
| Otolemur crassicaudatus | Superficial temporalis | Gross Dissection | 1 | 8.8 |  |  |  |
| Otolemur crassicaudatus | Superficial temporalis | Manual Segmentation | 15 | 8.29 | 1.64 | 1.07 | 0.03 |
| Otolemur crassicaudatus | Superficial temporalis | XFIBER | 60 | 7.84 | 2.24 | 1.01 | 0.01 |
| Otolemur crassicaudatus | Zygomatic temporalis | GoodFibes | 22 | 20.22 | 3.13 | 1.12 | 0.03 |
| Otolemur crassicaudatus | Zygomatic temporalis | Gross Dissection | 1 | 9 |  |  |  |
| Otolemur crassicaudatus | Zygomatic temporalis | Manual Segmentation | 8 | 6.05 | 1.12 | 1.06 | 0.04 |
| Otolemur crassicaudatus | Zygomatic temporalis | XFIBER | 43 | 7.06 | 2.83 | 1.02 | 0.02 |
| Otolemur crassicaudatus | Zygomaticomandibularis | GoodFibes | 22 | 9.52 | 3.52 | 1.02 | 0.01 |
| Otolemur crassicaudatus | Zygomaticomandibularis | Gross Dissection | 1 | 7 |  |  |  |
| Otolemur crassicaudatus | Zygomaticomandibularis | Manual Segmentation | 7 | 5.38 | 0.81 | 1.05 | 0.03 |
| Otolemur crassicaudatus | Zygomaticomandibularis | XFIBER | 38 | 5.15 | 2.13 | 1 | 0.01 |
| Procyon lotor | Deep masseter | GoodFibes | 43 | 27.13 | 2.12 | 1.02 | 0.02 |
| Procyon lotor | Deep masseter | Gross Dissection | 1 | 10.1 |  |  |  |
| Procyon lotor | Deep masseter | Manual Segmentation | 14 | 12.95 | 4.14 | 1.03 | 0.02 |
| Procyon lotor | Deep masseter | XFIBER | 48 | 12.5 | 1.74 | 1.01 | 0.01 |
| Procyon lotor | Deep temporalis | GoodFibes | 45 | 76.85 | 4.79 | 1.22 | 0.06 |
| Procyon lotor | Deep temporalis | Gross Dissection | 1 | 15.67 |  |  |  |
| Procyon lotor | Deep temporalis | Manual Segmentation | 30 | 18.18 | 5.13 | 1.13 | 0.08 |
| Procyon lotor | Deep temporalis | XFIBER | 72 | 19.21 | 5.71 | 1.01 | 0.02 |
| Procyon lotor | Digastric | GoodFibes | 28 | 55.48 | 1.19 | 1.02 | 0.02 |
| Procyon lotor | Digastric | Manual Segmentation | 20 | 12.25 | 9.35 | 1.01 | 0.02 |
| Procyon lotor | Digastric | XFIBER | 55 | 16.12 | 7.2 | 1 | 0 |
| Procyon lotor | Lateral pterygoid | GoodFibes | 28 | 16.09 | 1.46 | 1.08 | 0.07 |
| Procyon lotor | Lateral pterygoid | Manual Segmentation | 12 | 5.26 | 1.69 | 1.04 | 0.02 |
| Procyon lotor | Lateral pterygoid | XFIBER | 43 | 4.89 | 1.74 | 1.01 | 0.01 |
| Procyon lotor | Medial pterygoid | GoodFibes | 24 | 23.6 | 7.01 | 1.01 | 0.01 |
| Procyon lotor | Medial pterygoid | Gross Dissection | 1 | 7.71 |  |  |  |
| Procyon lotor | Medial pterygoid | Manual Segmentation | 29 | 7.23 | 2.53 | 1.04 | 0.02 |
| Procyon lotor | Medial pterygoid | XFIBER | 49 | 9.69 | 4.03 | 1.01 | 0.02 |
| Procyon lotor | Superficial masseter | GoodFibes | 41 | 15.23 | 5.37 | 1.05 | 0.04 |
| Procyon lotor | Superficial masseter | Gross Dissection | 1 | 10.1 |  |  |  |
| Procyon lotor | Superficial masseter | Manual Segmentation | 21 | 12.62 | 2.66 | 1.05 | 0.03 |
| Procyon lotor | Superficial masseter | XFIBER | 50 | 13.06 | 5.11 | 1.02 | 0.03 |
| Procyon lotor | Superficial temporalis | GoodFibes | 34 | 61.01 | 8.76 | 1.13 | 0.03 |
| Procyon lotor | Superficial temporalis | Gross Dissection | 1 | 15.67 |  |  |  |
| Procyon lotor | Superficial temporalis | Manual Segmentation | 34 | 16.09 | 4.6 | 1.08 | 0.21 |
| Procyon lotor | Superficial temporalis | XFIBER | 75 | 14.56 | 5.88 | 1.02 | 0.02 |
| Procyon lotor | Zygomaticomandibularis | GoodFibes | 38 | 21.03 | 6.52 | 1.04 | 0.03 |
| Procyon lotor | Zygomaticomandibularis | Gross Dissection | 1 | 10.1 |  |  |  |
| Procyon lotor | Zygomaticomandibularis | Manual Segmentation | 24 | 7.35 | 1.25 | 1.07 | 0.03 |
| Procyon lotor | Zygomaticomandibularis | XFIBER | 53 | 9.43 | 4.54 | 1.01 | 0.01 |

**Supplemental Table 4.** Means and standard deviations of percent change (%) subdivided by species and muscle for the various reconstruction methods assessed in this study.

| Species | Muscle | Method | N | Mean_Diff_GrossDissection | SD_Diff_GrossDissection | Mean_Diff_Manual_FL | SD_Diff_Manual_FL | Mean_Diff_Manual_Tort | SD_Diff_Manual_Tort |
| --- | --- | --- | --- | --- | --- | --- | --- | --- | --- |
| Daubentonia madagascariensis | Deep masseter | GoodFibes | 29 | 3.43 | 37.49 | -18.37 | 29.58 | -3.81 | 0.71 |
| Daubentonia madagascariensis | Deep masseter | Manual Segmentation | 14 | 26.71 | 30.64 |  |  |  |  |
| Daubentonia madagascariensis | Deep masseter | XFIBER | 64 | 2.35 | 36.49 | -19.22 | 28.80 | -4.13 | 0.60 |
| Daubentonia madagascariensis | Deep temporalis | GoodFibes | 44 | 69.85 | 77.87 | 89.12 | 86.70 | -7.62 | 2.54 |
| Daubentonia madagascariensis | Deep temporalis | Manual Segmentation | 26 | -10.19 | 43.39 |  |  |  |  |
| Daubentonia madagascariensis | Deep temporalis | XFIBER | 93 | -9.50 | 34.97 | 0.77 | 38.93 | -9.95 | 1.63 |
| Daubentonia madagascariensis | Lateral pterygoid | GoodFibes | 18 |  |  | -4.84 | 42.78 | -0.76 | 3.37 |
| Daubentonia madagascariensis | Lateral pterygoid | XFIBER | 52 |  |  | -5.93 | 36.62 | -2.70 | 1.30 |
| Daubentonia madagascariensis | Medial pterygoid | GoodFibes | 34 | 258.85 | 159.29 | 83.32 | 81.37 | -3.87 | 1.95 |
| Daubentonia madagascariensis | Medial pterygoid | Manual Segmentation | 12 | 95.75 | 39.36 |  |  |  |  |
| Daubentonia madagascariensis | Medial pterygoid | XFIBER | 55 | 48.88 | 55.24 | -23.94 | 28.22 | -5.40 | 1.13 |
| Daubentonia madagascariensis | Superficial masseter | GoodFibes | 39 | 19.85 | 51.67 | -19.45 | 34.73 | -4.92 | 1.59 |
| Daubentonia madagascariensis | Superficial masseter | Manual Segmentation | 16 | 48.79 | 32.70 |  |  |  |  |
| Daubentonia madagascariensis | Superficial masseter | XFIBER | 60 | 5.69 | 35.22 | -28.97 | 23.67 | -5.23 | 3.43 |
| Daubentonia madagascariensis | Superficial temporalis | GoodFibes | 35 | 97.58 | 175.93 | 51.74 | 135.11 | -4.77 | 4.75 |
| Daubentonia madagascariensis | Superficial temporalis | Manual Segmentation | 28 | 30.21 | 50.85 |  |  |  |  |
| Daubentonia madagascariensis | Superficial temporalis | XFIBER | 95 | 0.04 | 48.51 | -23.17 | 37.25 | -7.75 | 3.31 |
| Daubentonia madagascariensis | Zygomaticomandibularis | GoodFibes | 20 | 14.59 | 42.04 | 2.26 | 37.52 | -5.15 | 1.13 |
| Daubentonia madagascariensis | Zygomaticomandibularis | Manual Segmentation | 18 | 12.05 | 32.46 |  |  |  |  |
| Daubentonia madagascariensis | Zygomaticomandibularis | XFIBER | 43 | 7.18 | 35.77 | -4.35 | 31.92 | -5.10 | 1.67 |
| Eulemur corotus | Anterior digastric | GoodFibes | 12 |  |  | 155.14 | 17.01 | -3.43 | 0.77 |
| Eulemur corotus | Anterior digastric | XFIBER | 41 |  |  | 19.47 | 51.87 | -3.67 | 0.59 |
| Eulemur corotus | Deep masseter | GoodFibes | 22 | 114.28 | 63.80 | 143.39 | 72.47 | 7.52 | 17.31 |
| Eulemur corotus | Deep masseter | Manual Segmentation | 10 | -11.96 | 24.13 |  |  |  |  |
| Eulemur corotus | Deep masseter | XFIBER | 52 | 5.84 | 36.97 | 20.22 | 41.99 | -2.92 | 6.26 |
| Eulemur corotus | Deep temporalis | GoodFibes | 24 | 262.22 | 51.30 | 219.99 | 45.32 | 0.02 | 3.46 |
| Eulemur corotus | Deep temporalis | Manual Segmentation | 26 | 13.20 | 59.36 |  |  |  |  |
| Eulemur corotus | Deep temporalis | XFIBER | 58 | 40.83 | 80.68 | 24.41 | 71.28 | -5.10 | 4.86 |
| Eulemur corotus | Lateral pterygoid | GoodFibes | 22 |  |  | 112.68 | 17.93 | -1.53 | 1.88 |
| Eulemur corotus | Lateral pterygoid | Manual Segmentation | 10 |  |  |  |  |  |  |
| Eulemur corotus | Lateral pterygoid | XFIBER | 33 |  |  | 5.89 | 29.80 | -2.10 | 2.43 |
| Eulemur corotus | Medial pterygoid | GoodFibes | 22 | 9.85 | 45.22 | -3.36 | 39.78 | -0.75 | 7.11 |
| Eulemur corotus | Medial pterygoid | Manual Segmentation | 13 | 13.67 | 49.29 |  |  |  |  |
| Eulemur corotus | Medial pterygoid | XFIBER | 46 | 56.51 | 43.63 | 37.69 | 38.38 | -7.49 | 1.05 |
| Eulemur corotus | Posterior digastric | GoodFibes | 19 |  |  | 109.98 | 34.62 | -3.52 | 0.66 |
| Eulemur corotus | Posterior digastric | XFIBER | 30 |  |  | -4.59 | 38.67 | -3.36 | 0.94 |
| Eulemur corotus | Superficial masseter | GoodFibes | 26 | 118.44 | 92.29 | 100.96 | 84.91 | 10.98 | 12.13 |
| Eulemur corotus | Superficial masseter | Manual Segmentation | 17 | 8.70 | 30.43 |  |  |  |  |
| Eulemur corotus | Superficial masseter | XFIBER | 62 | 14.18 | 38.16 | 5.04 | 35.11 | -1.83 | 2.21 |
| Eulemur corotus | Superficial temporalis | GoodFibes | 20 | 129.00 | 25.57 | 103.50 | 22.73 | -2.88 | 2.80 |
| Eulemur corotus | Superficial temporalis | Manual Segmentation | 26 | 12.53 | 41.17 |  |  |  |  |
| Eulemur corotus | Superficial temporalis | XFIBER | 30 | 5.61 | 37.47 | -6.15 | 33.30 | -5.41 | 3.05 |
| Eulemur corotus | Zygomatic temporalis | GoodFibes | 18 | 102.65 | 32.75 | 85.75 | 30.01 | -2.40 | 5.49 |
| Eulemur corotus | Zygomatic temporalis | Manual Segmentation | 18 | 9.10 | 42.55 |  |  |  |  |
| Eulemur corotus | Zygomatic temporalis | XFIBER | 41 | 30.61 | 44.36 | 19.71 | 40.66 | -5.17 | 3.51 |
| Eulemur corotus | Zygomaticomandibularis | GoodFibes | 26 | 78.76 | 48.78 | 86.29 | 50.83 | -4.90 | 1.95 |
| Eulemur corotus | Zygomaticomandibularis | Manual Segmentation | 9 | -4.04 | 22.05 |  |  |  |  |
| Eulemur corotus | Zygomaticomandibularis | XFIBER | 44 | 2.42 | 31.17 | 6.73 | 32.48 | -6.15 | 1.52 |
| Lontra cadensis | Deep masseter | GoodFibes | 17 | -31.55 | 24.81 | -20.76 | 28.72 | -1.76 | 2.23 |
| Lontra cadensis | Deep masseter | Manual Segmentation | 13 | -13.61 | 15.68 |  |  |  |  |
| Lontra cadensis | Deep masseter | XFIBER | 60 | -22.30 | 26.56 | -10.06 | 30.74 | -2.65 | 2.10 |
| Lontra cadensis | Deep temporalis | GoodFibes | 40 | 100.26 | 75.91 | 77.14 | 67.15 | -2.99 | 5.61 |
| Lontra cadensis | Deep temporalis | Manual Segmentation | 28 | 13.05 | 46.13 |  |  |  |  |
| Lontra cadensis | Deep temporalis | XFIBER | 66 | -5.28 | 29.93 | -16.21 | 26.48 | -8.67 | 1.47 |
| Lontra cadensis | Digastric | GoodFibes | 44 |  |  | 19.26 | 19.10 | -1.94 | 1.14 |
| Lontra cadensis | Digastric | Manual Segmentation | 14 |  |  |  |  |  |  |
| Lontra cadensis | Digastric | XFIBER | 56 |  |  | -31.51 | 18.89 | -2.44 | 0.66 |
| Lontra cadensis | Lateral pterygoid | GoodFibes | 14 |  |  | 95.19 | 22.08 | 10.88 | 13.22 |
| Lontra cadensis | Lateral pterygoid | Manual Segmentation | 7 |  |  |  |  |  |  |
| Lontra cadensis | Lateral pterygoid | XFIBER | 36 |  |  | -12.87 | 17.85 | 0.23 | 2.91 |
| Lontra cadensis | Medial pterygoid | GoodFibes | 34 | 74.15 | 50.05 | 51.20 | 43.45 | -0.20 | 3.31 |
| Lontra cadensis | Medial pterygoid | Manual Segmentation | 12 | 15.18 | 16.41 |  |  |  |  |
| Lontra cadensis | Medial pterygoid | XFIBER | 44 | 40.97 | 51.99 | 22.39 | 45.13 | -2.76 | 2.83 |
| Lontra cadensis | Superficial masseter | GoodFibes | 24 | 55.33 | 28.05 | 27.90 | 23.10 | -2.45 | 2.73 |
| Lontra cadensis | Superficial masseter | Manual Segmentation | 26 | 21.45 | 30.78 |  |  |  |  |
| Lontra cadensis | Superficial masseter | XFIBER | 64 | 2.96 | 37.55 | -15.23 | 30.92 | -3.70 | 2.20 |
| Lontra cadensis | Superficial temporalis | GoodFibes | 41 | 82.28 | 87.44 | 108.98 | 100.25 | -3.15 | 3.39 |
| Lontra cadensis | Superficial temporalis | Manual Segmentation | 57 | -12.78 | 48.60 |  |  |  |  |
| Lontra cadensis | Superficial temporalis | XFIBER | 72 | -5.89 | 38.62 | 7.89 | 44.28 | -5.38 | 2.30 |
| Lontra cadensis | Zygomaticomandibularis | GoodFibes | 10 | -57.09 | 14.59 | -42.99 | 19.39 | -6.04 | 2.62 |
| Lontra cadensis | Zygomaticomandibularis | Manual Segmentation | 16 | -24.74 | 28.42 |  |  |  |  |
| Lontra cadensis | Zygomaticomandibularis | XFIBER | 52 | -13.20 | 25.15 | 15.33 | 33.41 | -6.38 | 1.72 |
| Martes america | Deep masseter | GoodFibes | 24 | 131.43 | 54.66 | 117.55 | 51.38 | 0.67 | 3.08 |
| Martes america | Deep masseter | Manual Segmentation | 16 | 6.38 | 41.60 |  |  |  |  |
| Martes america | Deep masseter | XFIBER | 36 | 27.60 | 43.23 | 19.95 | 40.63 | -0.02 | 4.06 |
| Martes america | Deep temporalis | GoodFibes | 35 | 211.68 | 92.96 | 88.10 | 56.10 | 6.19 | 3.89 |
| Martes america | Deep temporalis | Manual Segmentation | 24 | 65.70 | 51.54 |  |  |  |  |
| Martes america | Deep temporalis | XFIBER | 64 | 35.47 | 56.87 | -18.24 | 34.32 | -3.10 | 1.15 |
| Martes america | Digastric | GoodFibes | 37 |  |  | 61.81 | 6.28 | -0.96 | 1.35 |
| Martes america | Digastric | Manual Segmentation | 22 |  |  |  |  |  |  |
| Martes america | Digastric | XFIBER | 43 |  |  | -27.03 | 22.17 | -2.22 | 0.33 |
| Martes america | Lateral pterygoid | GoodFibes | 12 |  |  | 63.06 | 33.44 | 1.11 | 2.90 |
| Martes america | Lateral pterygoid | Manual Segmentation | 12 |  |  |  |  |  |  |
| Martes america | Lateral pterygoid | XFIBER | 28 |  |  | -26.99 | 31.45 | -1.48 | 1.71 |
| Martes america | Medial pterygoid | GoodFibes | 28 | 118.26 | 74.25 | 4.06 | 35.40 | -1.41 | 2.17 |
| Martes america | Medial pterygoid | Manual Segmentation | 28 | 109.75 | 82.74 |  |  |  |  |
| Martes america | Medial pterygoid | XFIBER | 36 | 147.47 | 82.54 | 17.99 | 39.35 | -0.01 | 2.90 |
| Martes america | Superficial masseter | GoodFibes | 31 | 152.83 | 61.47 | 77.26 | 43.10 | 0.51 | 6.38 |
| Martes america | Superficial masseter | Manual Segmentation | 34 | 42.64 | 38.20 |  |  |  |  |
| Martes america | Superficial masseter | XFIBER | 67 | -2.07 | 33.80 | -31.34 | 23.69 | -4.85 | 0.35 |
| Martes america | Superficial temporalis | GoodFibes | 36 | 190.95 | 112.06 | 56.41 | 60.24 | 5.04 | 6.77 |
| Martes america | Superficial temporalis | Manual Segmentation | 33 | 86.01 | 65.07 |  |  |  |  |
| Martes america | Superficial temporalis | XFIBER | 54 | 4.54 | 47.98 | -43.80 | 25.79 | -4.34 | 1.65 |
| Martes america | Zygomaticomandibularis | GoodFibes | 17 | 145.09 | 59.34 | 108.57 | 50.50 | -3.09 | 1.64 |
| Martes america | Zygomaticomandibularis | Manual Segmentation | 15 | 17.51 | 21.31 |  |  |  |  |
| Martes america | Zygomaticomandibularis | XFIBER | 39 | 31.48 | 42.19 | 11.89 | 35.90 | -2.72 | 1.59 |
| Mephitis mephitis | Deep masseter | GoodFibes | 16 | 43.50 | 41.96 | 17.68 | 34.41 | -1.69 | 2.38 |
| Mephitis mephitis | Deep masseter | Manual Segmentation | 13 | 21.95 | 36.97 |  |  |  |  |
| Mephitis mephitis | Deep masseter | XFIBER | 60 | 9.04 | 15.96 | -10.59 | 13.09 | -2.58 | 2.69 |
| Mephitis mephitis | Deep temporalis | GoodFibes | 30 | 299.91 | 29.07 | 255.03 | 25.81 | -0.62 | 2.30 |
| Mephitis mephitis | Deep temporalis | Manual Segmentation | 32 | 12.64 | 34.66 |  |  |  |  |
| Mephitis mephitis | Deep temporalis | XFIBER | 59 | 53.55 | 40.66 | 36.31 | 36.10 | -5.90 | 1.34 |
| Mephitis mephitis | Digastric | GoodFibes | 19 |  |  | 439.58 | 9.07 | 0.35 | 1.15 |
| Mephitis mephitis | Digastric | Manual Segmentation | 13 |  |  |  |  |  |  |
| Mephitis mephitis | Digastric | XFIBER | 54 |  |  | 88.45 | 89.74 | -0.12 | 0.77 |
| Mephitis mephitis | Lateral pterygoid | GoodFibes | 7 |  |  | 16.18 | 31.58 | 16.59 | 19.49 |
| Mephitis mephitis | Lateral pterygoid | Manual Segmentation | 7 |  |  |  |  |  |  |
| Mephitis mephitis | Lateral pterygoid | XFIBER | 39 |  |  | 16.54 | 26.30 | -1.59 | 1.08 |
| Mephitis mephitis | Medial pterygoid | GoodFibes | 19 | 109.77 | 44.93 | 94.30 | 41.62 | 2.18 | 7.42 |
| Mephitis mephitis | Medial pterygoid | Manual Segmentation | 14 | 7.96 | 39.45 |  |  |  |  |
| Mephitis mephitis | Medial pterygoid | XFIBER | 34 | 51.37 | 32.70 | 40.21 | 30.29 | -1.99 | 2.05 |
| Mephitis mephitis | Superficial masseter | GoodFibes | 24 | 113.43 | 64.46 | 53.46 | 46.35 | 8.79 | 11.16 |
| Mephitis mephitis | Superficial masseter | Manual Segmentation | 21 | 39.08 | 45.50 |  |  |  |  |
| Mephitis mephitis | Superficial masseter | XFIBER | 57 | 12.87 | 27.99 | -18.84 | 20.13 | -4.44 | 1.07 |
| Mephitis mephitis | Superficial temporalis | GoodFibes | 29 | 330.58 | 92.21 | 255.42 | 76.12 | 1.61 | 3.93 |
| Mephitis mephitis | Superficial temporalis | Manual Segmentation | 26 | 21.15 | 28.47 |  |  |  |  |
| Mephitis mephitis | Superficial temporalis | XFIBER | 65 | -3.95 | 36.95 | -20.72 | 30.50 | -5.53 | 1.20 |
| Mephitis mephitis | Zygomaticomandibularis | GoodFibes | 25 | 54.98 | 44.75 | 119.63 | 63.43 | 0.92 | 5.69 |
| Mephitis mephitis | Zygomaticomandibularis | Manual Segmentation | 16 | -29.44 | 28.66 |  |  |  |  |
| Mephitis mephitis | Zygomaticomandibularis | XFIBER | 55 | -3.21 | 27.15 | 37.18 | 38.48 | -3.52 | 1.15 |
| Mustela nivalis | Deep masseter | GoodFibes | 20 | 79.59 | 30.28 | 117.55 | 36.68 | -2.16 | 2.35 |
| Mustela nivalis | Deep masseter | Manual Segmentation | 8 | -17.45 | 15.54 |  |  |  |  |
| Mustela nivalis | Deep masseter | XFIBER | 30 | 2.99 | 20.48 | 24.75 | 24.81 | -4.69 | 1.58 |
| Mustela nivalis | Deep temporalis | GoodFibes | 36 | 183.18 | 42.39 | 599.51 | 104.72 | -5.70 | 1.51 |
| Mustela nivalis | Deep temporalis | Manual Segmentation | 24 | -59.52 | 5.51 |  |  |  |  |
| Mustela nivalis | Deep temporalis | XFIBER | 62 | -11.30 | 38.81 | 119.10 | 95.87 | -9.84 | 0.75 |
| Mustela nivalis | Digastric | GoodFibes | 27 |  |  | 294.18 | 49.79 | -1.48 | 0.46 |
| Mustela nivalis | Digastric | Manual Segmentation | 10 |  |  |  |  |  |  |
| Mustela nivalis | Digastric | XFIBER | 47 |  |  | 94.62 | 96.67 | -1.04 | 1.35 |
| Mustela nivalis | Lateral pterygoid | GoodFibes | 13 |  |  | 71.89 | 17.23 | 6.54 | 4.89 |
| Mustela nivalis | Lateral pterygoid | Manual Segmentation | 5 |  |  |  |  |  |  |
| Mustela nivalis | Lateral pterygoid | XFIBER | 41 |  |  | 33.83 | 32.36 | -0.12 | 1.36 |
| Mustela nivalis | Medial pterygoid | GoodFibes | 27 | 51.90 | 25.97 | 139.61 | 40.97 | -1.39 | 3.56 |
| Mustela nivalis | Medial pterygoid | Manual Segmentation | 8 | -36.60 | 15.12 |  |  |  |  |
| Mustela nivalis | Medial pterygoid | XFIBER | 45 | -13.16 | 29.32 | 36.98 | 46.24 | -4.75 | 0.75 |
| Mustela nivalis | Superficial masseter | GoodFibes | 30 | 82.68 | 51.60 | 95.31 | 55.17 | -0.36 | 3.62 |
| Mustela nivalis | Superficial masseter | Manual Segmentation | 9 | -6.46 | 20.22 |  |  |  |  |
| Mustela nivalis | Superficial masseter | XFIBER | 35 | 26.03 | 38.85 | 34.74 | 41.53 | -2.57 | 3.37 |
| Mustela nivalis | Superficial temporalis | GoodFibes | 28 | 50.90 | 21.58 | 222.20 | 46.07 | -9.28 | 1.21 |
| Mustela nivalis | Superficial temporalis | Manual Segmentation | 14 | -53.16 | 9.27 |  |  |  |  |
| Mustela nivalis | Superficial temporalis | XFIBER | 61 | -22.12 | 26.68 | 66.28 | 56.97 | -10.44 | 0.80 |
| Mustela nivalis | Zygomaticomandibularis | GoodFibes | 25 | 25.07 | 17.20 | 50.99 | 20.77 | -2.14 | 3.31 |
| Mustela nivalis | Zygomaticomandibularis | Manual Segmentation | 8 | -17.17 | 21.58 |  |  |  |  |
| Mustela nivalis | Zygomaticomandibularis | XFIBER | 46 | 14.13 | 30.23 | 37.78 | 36.50 | -3.92 | 2.42 |
| Otolemur crassicaudatus | Anterior digastric | GoodFibes | 16 |  |  | 295.11 | 117.26 | -1.97 | 1.72 |
| Otolemur crassicaudatus | Anterior digastric | Manual Segmentation | 8 |  |  |  |  |  |  |
| Otolemur crassicaudatus | Anterior digastric | XFIBER | 34 |  |  | 117.32 | 103.06 | -2.16 | 1.76 |
| Otolemur crassicaudatus | Deep masseter | GoodFibes | 25 | 65.11 | 52.68 | 111.21 | 67.39 | -4.06 | 0.63 |
| Otolemur crassicaudatus | Deep masseter | Manual Segmentation | 8 | -21.82 | 9.12 |  |  |  |  |
| Otolemur crassicaudatus | Deep masseter | XFIBER | 43 | -9.43 | 22.38 | 15.85 | 28.63 | -4.29 | 0.71 |
| Otolemur crassicaudatus | Deep temporalis | GoodFibes | 14 | 88.64 | 74.22 | 70.58 | 67.11 | -4.74 | 3.94 |
| Otolemur crassicaudatus | Deep temporalis | Manual Segmentation | 14 | 10.59 | 25.34 |  |  |  |  |
| Otolemur crassicaudatus | Deep temporalis | XFIBER | 74 | 24.43 | 75.43 | 12.52 | 68.21 | -7.79 | 2.04 |
| Otolemur crassicaudatus | Lateral pterygoid | GoodFibes | 17 |  |  | 286.46 | 63.20 | -0.36 | 4.02 |
| Otolemur crassicaudatus | Lateral pterygoid | Manual Segmentation | 15 |  |  |  |  |  |  |
| Otolemur crassicaudatus | Lateral pterygoid | XFIBER | 40 |  |  | 72.94 | 63.18 | -2.09 | 2.28 |
| Otolemur crassicaudatus | Medial pterygoid | GoodFibes | 22 | 66.95 | 61.78 | 58.18 | 58.53 | -3.20 | 1.25 |
| Otolemur crassicaudatus | Medial pterygoid | Manual Segmentation | 11 | 5.55 | 15.65 |  |  |  |  |
| Otolemur crassicaudatus | Medial pterygoid | XFIBER | 45 | 33.70 | 44.23 | 26.68 | 41.91 | -4.16 | 1.05 |
| Otolemur crassicaudatus | Posterior digastric | GoodFibes | 20 |  |  | 337.86 | 45.95 | -3.35 | 0.89 |
| Otolemur crassicaudatus | Posterior digastric | Manual Segmentation | 10 |  |  |  |  |  |  |
| Otolemur crassicaudatus | Posterior digastric | XFIBER | 37 |  |  | 62.29 | 89.84 | -2.63 | 1.57 |
| Otolemur crassicaudatus | Superficial masseter | GoodFibes | 19 | 150.71 | 46.16 | 154.14 | 46.79 | -3.48 | 2.49 |
| Otolemur crassicaudatus | Superficial masseter | Manual Segmentation | 10 | -1.35 | 19.43 |  |  |  |  |
| Otolemur crassicaudatus | Superficial masseter | XFIBER | 56 | 9.82 | 32.77 | 11.32 | 33.21 | -6.84 | 0.55 |
| Otolemur crassicaudatus | Superficial temporalis | GoodFibes | 28 | 84.44 | 98.32 | 95.87 | 104.41 | -0.59 | 3.60 |
| Otolemur crassicaudatus | Superficial temporalis | Manual Segmentation | 15 | -5.84 | 18.62 |  |  |  |  |
| Otolemur crassicaudatus | Superficial temporalis | XFIBER | 60 | -10.86 | 25.49 | -5.34 | 27.07 | -5.58 | 1.09 |
| Otolemur crassicaudatus | Zygomatic temporalis | GoodFibes | 22 | 124.69 | 34.75 | 234.01 | 51.65 | 5.12 | 3.20 |
| Otolemur crassicaudatus | Zygomatic temporalis | Manual Segmentation | 8 | -32.73 | 12.49 |  |  |  |  |
| Otolemur crassicaudatus | Zygomatic temporalis | XFIBER | 43 | -21.56 | 31.48 | 16.60 | 46.79 | -3.93 | 2.14 |
| Otolemur crassicaudatus | Zygomaticomandibularis | GoodFibes | 22 | 35.94 | 50.30 | 77.00 | 65.50 | -2.41 | 1.26 |
| Otolemur crassicaudatus | Zygomaticomandibularis | Manual Segmentation | 7 | -23.20 | 11.63 |  |  |  |  |
| Otolemur crassicaudatus | Zygomaticomandibularis | XFIBER | 38 | -26.48 | 30.39 | -4.28 | 39.56 | -4.21 | 0.56 |
| Procyon lotor | Deep masseter | GoodFibes | 43 | 168.58 | 20.96 | 109.45 | 16.35 | -0.80 | 1.52 |
| Procyon lotor | Deep masseter | Manual Segmentation | 14 | 28.23 | 40.97 |  |  |  |  |
| Procyon lotor | Deep masseter | XFIBER | 48 | 23.78 | 17.27 | -3.47 | 13.47 | -1.73 | 0.91 |
| Procyon lotor | Deep temporalis | GoodFibes | 45 | 390.42 | 30.58 | 322.78 | 26.36 | 7.59 | 5.43 |
| Procyon lotor | Deep temporalis | Manual Segmentation | 30 | 16.00 | 32.76 |  |  |  |  |
| Procyon lotor | Deep temporalis | XFIBER | 72 | 22.57 | 36.41 | 5.66 | 31.39 | -10.25 | 1.86 |
| Procyon lotor | Digastric | GoodFibes | 28 |  |  | 352.75 | 9.71 | 0.68 | 1.57 |
| Procyon lotor | Digastric | Manual Segmentation | 20 |  |  |  |  |  |  |
| Procyon lotor | Digastric | XFIBER | 55 |  |  | 31.54 | 58.77 | -0.75 | 0.42 |
| Procyon lotor | Lateral pterygoid | GoodFibes | 28 |  |  | 205.89 | 27.71 | 3.85 | 6.69 |
| Procyon lotor | Lateral pterygoid | Manual Segmentation | 12 |  |  |  |  |  |  |
| Procyon lotor | Lateral pterygoid | XFIBER | 43 |  |  | -7.04 | 33.13 | -2.93 | 0.83 |
| Procyon lotor | Medial pterygoid | GoodFibes | 24 | 206.14 | 90.89 | 226.49 | 96.93 | -3.40 | 0.54 |
| Procyon lotor | Medial pterygoid | Manual Segmentation | 29 | -6.23 | 32.87 |  |  |  |  |
| Procyon lotor | Medial pterygoid | XFIBER | 49 | 25.65 | 52.30 | 34.00 | 55.78 | -3.05 | 1.53 |
| Procyon lotor | Superficial masseter | GoodFibes | 41 | 50.82 | 53.13 | 20.68 | 42.51 | 0.02 | 3.45 |
| Procyon lotor | Superficial masseter | Manual Segmentation | 21 | 24.98 | 26.37 |  |  |  |  |
| Procyon lotor | Superficial masseter | XFIBER | 50 | 29.30 | 50.62 | 3.46 | 40.50 | -2.44 | 2.47 |
| Procyon lotor | Superficial temporalis | GoodFibes | 34 | 289.35 | 55.92 | 279.10 | 54.45 | 4.20 | 2.40 |
| Procyon lotor | Superficial temporalis | Manual Segmentation | 34 | 2.70 | 29.33 |  |  |  |  |
| Procyon lotor | Superficial temporalis | XFIBER | 75 | -7.08 | 37.51 | -9.52 | 36.52 | -6.14 | 1.95 |
| Procyon lotor | Zygomaticomandibularis | GoodFibes | 38 | 108.26 | 64.55 | 186.29 | 88.73 | -2.92 | 3.04 |
| Procyon lotor | Zygomaticomandibularis | Manual Segmentation | 24 | -27.26 | 12.40 |  |  |  |  |
| Procyon lotor | Zygomaticomandibularis | XFIBER | 53 | -6.62 | 44.91 | 28.37 | 61.74 | -5.00 | 1.39 |

**Supplemental Table 5.** Results from Mann-Whitney *U*-tests comparing whether fascicle lengths and tortuosity estimated from *XFiber* differed significantly from those derived from manual segmentation.

| Species | Muscle | FL_U | FL_Z | FL_P | T_U | T_Z | T_P |
| --- | --- | --- | --- | --- | --- | --- | --- |
| Daubentonia madagascariensis | Deep masseter | 632 | -2.39 | 0.017 | 836 | -5.05 | <0.001 |
| Daubentonia madagascariensis | Deep temporalis | 1226 | -0.11 | 0.915 | 2370 | -7.46 | <0.001 |
| Daubentonia madagascariensis | Lateral pterygoid | 379 | -1.14 | 0.253 | 497 | -3.17 | 0.002 |
| Daubentonia madagascariensis | Medial pterygoid | 536 | -3.36 | <0.001 | 623 | -4.78 | <0.001 |
| Daubentonia madagascariensis | Superficial masseter | 791 | -3.96 | <0.001 | 906 | -5.42 | <0.001 |
| Daubentonia madagascariensis | Superficial temporalis | 1954 | -3.76 | <0.001 | 2452 | -6.76 | <0.001 |
| Daubentonia madagascariensis | Zygomaticomandibularis | 430 | -0.67 | 0.502 | 599 | -3.34 | <0.001 |
| Eulemur coronatus | Deep masseter | 199 | -1.16 | 0.247 | 414 | -2.94 | 0.003 |
| Eulemur coronatus | Deep temporalis | 620 | -1.29 | 0.196 | 1164 | -3.96 | <0.001 |
| Eulemur coronatus | Lateral pterygoid | 160 | -0.13 | 0.897 | 261 | -2.75 | 0.006 |
| Eulemur coronatus | Medial pterygoid | 172 | -2.31 | 0.021 | 591 | -5.33 | <0.001 |
| Eulemur coronatus | Superficial masseter | 509 | -0.21 | 0.835 | 765 | -2.83 | 0.005 |
| Eulemur coronatus | Superficial temporalis | 463 | -1.19 | 0.234 | 677 | -4.71 | <0.001 |
| Eulemur coronatus | Zygomaticomandibularis | 189 | -0.2 | 0.84 | 362 | -3.87 | <0.001 |
| Eulemur coronatus | Anterior digastric | 164 | -0.96 | 0.337 | 361 | -3.69 | <0.001 |
| Eulemur coronatus | Posterior digastric | 205 | -0.25 | 0.802 | 325 | -3.42 | <0.001 |
| Eulemur coronatus | Zygomatic temporalis | 253 | -1.9 | 0.057 | 605 | -3.88 | <0.001 |
| Lontra canadensis | Deep masseter | 511 | -1.74 | 0.082 | 730 | -4.9 | <0.001 |
| Lontra canadensis | Deep temporalis | 1143 | -1.81 | 0.071 | 1827 | -7.46 | <0.001 |
| Lontra canadensis | Lateral pterygoid | 157 | -1 | 0.316 | 148 | -0.71 | 0.479 |
| Lontra canadensis | Medial pterygoid | 180 | -1.67 | 0.095 | 447 | -3.64 | <0.001 |
| Lontra canadensis | Superficial masseter | 1157 | -2.89 | 0.004 | 1405 | -5.1 | <0.001 |
| Lontra canadensis | Superficial temporalis | 1824 | -1.08 | 0.281 | 3722 | -7.92 | <0.001 |
| Lontra canadensis | Zygomaticomandibularis | 300 | -1.67 | 0.095 | 734 | -4.59 | <0.001 |
| Lontra canadensis | Digastric | 705 | -4.59 | <0.001 | 765 | -5.47 | <0.001 |
| Martes americana | Deep masseter | 198 | -1.77 | 0.076 | 347 | -1.16 | 0.246 |
| Martes americana | Deep temporalis | 1093 | -3.04 | 0.002 | 1443 | -6.32 | <0.001 |
| Martes americana | Lateral pterygoid | 259 | -2.67 | 0.008 | 259 | -2.67 | 0.008 |
| Martes americana | Medial pterygoid | 381 | -1.66 | 0.097 | 566 | -0.83 | 0.405 |
| Martes americana | Superficial masseter | 1869 | -5.24 | <0.001 | 2211 | -7.7 | <0.001 |
| Martes americana | Superficial temporalis | 1519 | -5.49 | <0.001 | 1670 | -6.81 | <0.001 |
| Martes americana | Zygomaticomandibularis | 256 | -0.7 | 0.487 | 513 | -4.25 | <0.001 |
| Martes americana | Digastric | 666 | -2.67 | 0.008 | 925 | -6.26 | <0.001 |
| Mephitis mephitis | Deep masseter | 520 | -1.87 | 0.062 | 664 | -3.94 | <0.001 |
| Mephitis mephitis | Deep temporalis | 453 | -4.08 | <0.001 | 1861 | -7.62 | <0.001 |
| Mephitis mephitis | Lateral pterygoid | 84 | -1.59 | 0.112 | 248 | -3.39 | <0.001 |
| Mephitis mephitis | Medial pterygoid | 96 | -3.21 | 0.001 | 350 | -2.53 | 0.011 |
| Mephitis mephitis | Superficial masseter | 811 | -2.39 | 0.017 | 1101 | -5.66 | <0.001 |
| Mephitis mephitis | Superficial temporalis | 1197 | -3.09 | 0.002 | 1595 | -6.58 | <0.001 |
| Mephitis mephitis | Zygomaticomandibularis | 170 | -3.71 | <0.001 | 851 | -5.65 | <0.001 |
| Mephitis mephitis | Digastric | 91 | -4.11 | <0.001 | 411 | -0.94 | 0.345 |
| Mustela nivalis | Deep masseter | 46 | -2.63 | 0.008 | 229 | -3.88 | <0.001 |
| Mustela nivalis | Deep temporalis | 7 | 7.09 | <0.001 | 1488 | -7.16 | <0.001 |
| Mustela nivalis | Lateral pterygoid | 35 | -2.36 | 0.018 | 130 | -0.95 | 0.341 |
| Mustela nivalis | Medial pterygoid | 82 | -2.42 | 0.015 | 360 | -4.46 | <0.001 |
| Mustela nivalis | Superficial masseter | 72 | -2.47 | 0.013 | 255 | -2.82 | 0.005 |
| Mustela nivalis | Superficial temporalis | 100 | -4.44 | <0.001 | 854 | -5.8 | <0.001 |
| Mustela nivalis | Zygomaticomandibularis | 77 | -2.59 | 0.01 | 329 | -3.52 | <0.001 |
| Mustela nivalis | Digastric | 99 | -2.84 | 0.004 | 393 | -3.3 | <0.001 |
| Otolemur crassicaudatus | Deep masseter | 126 | -1.18 | 0.239 | 338 | -4.29 | <0.001 |
| Otolemur crassicaudatus | Deep temporalis | 553 | -0.39 | 0.694 | 995 | -5.44 | <0.001 |
| Otolemur crassicaudatus | Lateral pterygoid | 65 | -4.43 | <0.001 | 440 | -2.64 | 0.008 |
| Otolemur crassicaudatus | Medial pterygoid | 161 | -1.77 | 0.076 | 395 | -3.03 | 0.002 |
| Otolemur crassicaudatus | Superficial masseter | 252 | -0.49 | 0.623 | 522 | -4.32 | <0.001 |
| Otolemur crassicaudatus | Superficial temporalis | 547 | -1.28 | 0.201 | 884 | -5.74 | <0.001 |
| Otolemur crassicaudatus | Zygomaticomandibularis | 179 | -1.42 | 0.154 | 263 | -4.06 | <0.001 |
| Otolemur crassicaudatus | Anterior digastric | 30 | -3.38 | <0.001 | 233 | -3.09 | 0.002 |
| Otolemur crassicaudatus | Posterior digastric | 94 | -2.35 | 0.019 | 287 | -2.64 | 0.008 |
| Otolemur crassicaudatus | Zygomatic temporalis | 150 | -0.56 | 0.578 | 297 | -3.22 | 0.001 |
| Procyon lotor | Deep masseter | 418 | -1.37 | 0.17 | 574 | -4 | <0.001 |
| Procyon lotor | Deep temporalis | 1019 | -0.44 | 0.657 | 1981 | -6.61 | <0.001 |
| Procyon lotor | Lateral pterygoid | 307 | -0.99 | 0.323 | 478 | -4.47 | <0.001 |
| Procyon lotor | Medial pterygoid | 445 | -2.74 | 0.006 | 1279 | -5.87 | <0.001 |
| Procyon lotor | Superficial masseter | 531 | -0.07 | 0.945 | 779 | -3.19 | 0.001 |
| Procyon lotor | Superficial temporalis | 1598 | -2.11 | 0.035 | 2188 | -5.97 | <0.001 |
| Procyon lotor | Zygomaticomandibularis | 516 | -1.31 | 0.189 | 1219 | -6.41 | <0.001 |
| Procyon lotor | Digastric | 302 | -2.97 | 0.003 | 741 | -2.28 | 0.022 |

**Supplemental Table 6.** Results from Mann-Whitney *U*-tests comparing whether fascicle lengths and tortuosity estimated from *GoodFibes* (Arbour, 2023) differed significantly from those derived from manual segmentation. Blank spaces represent muscles that could not be algorithmically generated by *GoodFibes*.

| Species | Muscle | FL_U | FL_Z | FL_P | T_U | T_Z | T_P |
| --- | --- | --- | --- | --- | --- | --- | --- |
| Daubentonia madagascariensis | Deep masseter | 125 | -2.01 | 0.045 | 42 | -4.16 | <0.001 |
| Daubentonia madagascariensis | Deep temporalis | 912 | -4.13 | <0.001 | 128 | -5.39 | <0.001 |
| Daubentonia madagascariensis | Lateral pterygoid | 96 | -0.49 | 0.626 | 86 | -0.91 | 0.363 |
| Daubentonia madagascariensis | Medial pterygoid | 330 | -3.14 | 0.002 | 67 | -3.41 | <0.001 |
| Daubentonia madagascariensis | Superficial masseter | 170 | -2.62 | 0.009 | 54 | -4.77 | <0.001 |
| Daubentonia madagascariensis | Superficial temporalis | 504 | -0.19 | 0.852 | 278 | -2.93 | 0.003 |
| Daubentonia madagascariensis | Zygomaticomandibularis | 187 | -0.19 | 0.849 | 94 | -2.5 | 0.012 |
| Eulemur coronatus | Deep masseter | 216 | -4.29 | <0.001 | 103 | -0.26 | 0.792 |
| Eulemur coronatus | Deep temporalis | 618 | -5.93 | <0.001 | 363 | -0.98 | 0.327 |
| Eulemur coronatus | Lateral pterygoid | 220 | -4.45 | <0.001 | 71 | -1.57 | 0.118 |
| Eulemur coronatus | Medial pterygoid | 145 | -0.05 | 0.959 | 110 | -1.11 | 0.267 |
| Eulemur coronatus | Superficial masseter | 376 | 3.85 | <0.001 | 357 | 3.38 | <0.001 |
| Eulemur coronatus | Superficial temporalis | 518 | -5.71 | <0.001 | 175 | -1.87 | 0.061 |
| Eulemur coronatus | Zygomaticomandibularis | 224 | -4.02 | <0.001 | 36 | -3.04 | 0.002 |
| Eulemur coronatus | Anterior digastric | 120 | -3.92 | <0.001 | 21 | -2.54 | 0.011 |
| Eulemur coronatus | Posterior digastric | 237 | -4.34 | <0.001 | 46 | -2.95 | 0.003 |
| Eulemur coronatus | Zygomatic temporalis | 301 | -4.38 | <0.001 | 103 | -1.85 | 0.064 |
| Lontra canadensis | Deep masseter | 61 | -2.05 | 0.04 | 52 | -2.43 | 0.015 |
| Lontra canadensis | Deep temporalis | 907 | -4.32 | <0.001 | 318 | -3.01 | 0.003 |
| Lontra canadensis | Lateral pterygoid | 98 | -3.62 | <0.001 | 68 | -1.38 | 0.168 |
| Lontra canadensis | Medial pterygoid | 358 | -3.84 | <0.001 | 192 | -0.29 | 0.774 |
| Lontra canadensis | Superficial masseter | 506 | -3.76 | <0.001 | 194 | -2.28 | 0.023 |
| Lontra canadensis | Superficial temporalis | 2002 | -6 | <0.001 | 634 | -3.85 | <0.001 |
| Lontra canadensis | Zygomaticomandibularis | 21 | -3.08 | 0.002 | 20 | -3.14 | 0.002 |
| Lontra canadensis | Digastric | 469 | -2.92 | 0.004 | 93 | -3.9 | <0.001 |
| Martes americana | Deep masseter | 368 | -4.85 | <0.001 | 198 | -0.15 | 0.879 |
| Martes americana | Deep temporalis | 758 | -5.21 | <0.001 | 783 | -5.59 | <0.001 |
| Martes americana | Lateral pterygoid | 133 | -3.49 | <0.001 | 93 | -1.18 | 0.237 |
| Martes americana | Medial pterygoid | 440 | -0.78 | 0.436 | 230 | -2.65 | 0.008 |
| Martes americana | Superficial masseter | 959 | -5.67 | <0.001 | 439 | -1.15 | 0.25 |
| Martes americana | Superficial temporalis | 932 | -4.05 | <0.001 | 855 | -3.13 | 0.002 |
| Martes americana | Zygomaticomandibularis | 252 | -4.68 | <0.001 | 24 | -3.89 | <0.001 |
| Martes americana | Digastric | 754 | -5.43 | <0.001 | 254 | -2.39 | 0.017 |
| Mephitis mephitis | Deep masseter | 136 | -1.38 | 0.167 | 53 | -2.21 | 0.027 |
| Mephitis mephitis | Deep temporalis | 960 | -6.75 | <0.001 | 412 | -0.95 | 0.342 |
| Mephitis mephitis | Lateral pterygoid | 35 | -1.28 | 0.201 | 35 | -1.28 | 0.201 |
| Mephitis mephitis | Medial pterygoid | 256 | -4.46 | <0.001 | 130 | -0.09 | 0.927 |
| Mephitis mephitis | Superficial masseter | 410 | -3.58 | <0.001 | 381 | -2.92 | 0.003 |
| Mephitis mephitis | Superficial temporalis | 733 | -5.99 | <0.001 | 506 | -2.17 | 0.03 |
| Mephitis mephitis | Zygomaticomandibularis | 376 | -4.69 | <0.001 | 159 | -1.08 | 0.279 |
| Mephitis mephitis | Digastric | 247 | -4.72 | <0.001 | 131 | -0.27 | 0.788 |
| Mustela nivalis | Deep masseter | 160 | -4.04 | <0.001 | 34 | -2.31 | 0.021 |
| Mustela nivalis | Deep temporalis | 864 | -6.51 | <0.001 | 27 | 6.1 | <0.001 |
| Mustela nivalis | Lateral pterygoid | 65 | -3.15 | 0.002 | 56 | -2.27 | 0.023 |
| Mustela nivalis | Medial pterygoid | 216 | -4.22 | <0.001 | 70 | -1.47 | 0.141 |
| Mustela nivalis | Superficial masseter | 239 | -3.45 | <0.001 | 118 | -0.55 | 0.582 |
| Mustela nivalis | Superficial temporalis | 392 | -5.22 | <0.001 | 1 | 5.19 | <0.001 |
| Mustela nivalis | Zygomaticomandibularis | 188 | -3.68 | <0.001 | 49 | -2.12 | 0.034 |
| Mustela nivalis | Digastric | 269 | -4.57 | <0.001 | 15 | 4.09 | <0.001 |
| Otolemur crassicaudatus | Deep masseter | 192 | -3.84 | <0.001 | 9 | 3.8 | <0.001 |
| Otolemur crassicaudatus | Deep temporalis | 169 | -3.24 | 0.001 | 36 | -2.83 | 0.005 |
| Otolemur crassicaudatus | Lateral pterygoid | 255 | -4.8 | <0.001 | 104 | -0.87 | 0.385 |
| Otolemur crassicaudatus | Medial pterygoid | 183 | -2.35 | 0.019 | 64 | -2.16 | 0.031 |
| Otolemur crassicaudatus | Superficial masseter | 190 | -4.34 | <0.001 | 49 | -2.09 | 0.037 |
| Otolemur crassicaudatus | Superficial temporalis | 360 | -3.81 | <0.001 | 172 | -0.96 | 0.339 |
| Otolemur crassicaudatus | Zygomaticomandibularis | 124 | -2.37 | 0.018 | 32 | -2.27 | 0.023 |
| Otolemur crassicaudatus | Anterior digastric | 128 | -3.89 | <0.001 | 27 | -2.24 | 0.025 |
| Otolemur crassicaudatus | Posterior digastric | 200 | -4.38 | <0.001 | 27 | -3.19 | 0.001 |
| Otolemur crassicaudatus | Zygomatic temporalis | 175 | -4.06 | <0.001 | 150 | -2.88 | 0.004 |
| Procyon lotor | Deep masseter | 602 | -5.57 | <0.001 | 216 | -1.57 | 0.117 |
| Procyon lotor | Deep temporalis | 1350 | -7.29 | <0.001 | 1096 | -4.55 | <0.001 |
| Procyon lotor | Lateral pterygoid | 336 | -4.94 | <0.001 | 214 | -1.34 | 0.179 |
| Procyon lotor | Medial pterygoid | 694 | -6.17 | <0.001 | 27 | -5.73 | <0.001 |
| Procyon lotor | Superficial masseter | 552 | -1.8 | 0.072 | 421 | -0.13 | 0.894 |
| Procyon lotor | Superficial temporalis | 1156 | -7.08 | <0.001 | 1064 | -5.95 | <0.001 |
| Procyon lotor | Zygomaticomandibularis | 862 | -5.86 | <0.001 | 208 | -3.58 | <0.001 |
| Procyon lotor | Digastric | 560 | -5.84 | <0.001 | 364 | -1.75 | 0.081 |
