# Supplementary material for: Multi‐method analysis for the three‐dimensional reconstruction of muscle fascicles from DiceCT datasets
Source: Anat Rec (Hoboken). 2025 Dec 4;309(12):3491–510. doi: 10.1002/ar.70088 (PMC13749328; doi:10.1002/ar.70088)
Supplement: Supplementary file 8 [file AR-309-3491-s006.docx]

Supplemental Document 1

Multi-method analysis of three-dimensional techniques for the reconstruction of muscle architecture

Aleksandra Ratkiewicz, Michael C. Granatosky, Cassidy E. Davis, Julia Molnar, Adam Hartstone-Rose, Edwin Dickinson

Isolating individual muscles

1) Segment muscles from scan and make a separate file for each muscle
   a) Open CT stack (e.g., “Specimen_stack.am”)
   b) Segment muscles, labeling every individual muscle (e.g., “muscleA.labels”)
   c) Copy labeled muscle file, delete all but one muscle – rename file with muscle name (e.g., “Specimen_stack_muscleA.am”)
2) Isolate the voxels associated with a single muscle
   a) In the Segmentation tab, select the labeled voxels and click “grow selection” in the Selection window
   b) Repeat approximately 4 times (5 “grows” is best for most muscles, 3x for smaller muscles, 7x for complex muscles)
   c) Create a new label field with title including number of grows (e.g., “muscleA_plus5.labels”)
   d) Add selection to a material in the new label field and save it
   e) Return to Project tab, select your image stack, and create an Arithmetic module (Compute>Arithmetic)
   f) In the Properties field of the Arithmetic module, enter the following properties (Fig. 1a):
      - Input A: image stack (Specimen_muscleA.am)
      - Input B: “grown” label field (muscleA_plus5.labels)
      - Expression: a*(b>0)
      - All other properties can be left at their default values

Figure 1. Arithmetic module for isolating voxels of a single muscle (temporalis) (left) and preview of resulting file (right)

g) Apply Arithmetic and save the result (e.g., “Result_Specimen_muscleA”). Your result preview should be a black field with a greyscale image where you can begin to see some muscle fascicles (Fig. 1b)

**Project view: Isolating individual muscle fibers (step 2)**

h) Delete the original image stack and labels file, leaving only the Result stack

3) Crop the Result stack (if the scans are large, this will significantly cut down on processing time)
   a) In the toolbar, switch from Perspective (default) to Orthographic view and turn on 4 viewers. Set each viewer with a different pre-set view (xy, xy, yz) (Fig. 2a)
   b) Create a Volume Rendering module
   c) Select the Result stack and select Crop Editor in the Properties window
   d) Select the Interact tool from the Toolbar and visually adjust the crop box in all 3 views to include only muscle A by dragging the green boxes (make sure that the crop box is approximately cubical – if one side is shorter it can cause problems with cylinder correlation) (Fig. 2b)

*Note: cropping is irreversible, so be careful not to cut off part of your muscle.*

Figure 2. Toolbar settings for cropping the results file (top) and crop boxes adjusted to size of muscle (bottom)

# Cylinder correlation

*Note: instructions for cylinder tracing using multiple cylinders (used for multipennate muscles) is located at the end of this document.*

1) Delete the Volume Rendering module and return to Single Viewer (Toolbar)
2) Select the cropped Result file and create a Cylinder Correlation module (Fiber Tracing>Visual Cylinder)
3) In the Properties window, turn on “Visual Cylinder”
4) Adjust Slice Number and Orientation to try to find a representative fascicle.
5) To refine your search, check the “Rotate” box and rotate the slice by dragging on the sphere with the Interact pointer
6) You can also adjust the Colormap to show the fascicles better, although this does not affect the cylinder tracing function.
7) Still using the Interact pointer, use the middle mouse button/mouse wheel to click once on the beginning of the fascicle and once on the end. A red cylinder should appear between the two points you clicked (Fig. 3)

FIGURE 3. CHOOSING A FASCICLE, AND SETTING THE VISUAL CYLINDER, AND ADJUSTING CYLINDER PROPERTIES. AFTER ADJUSTING THE COLOR MAP TO MAKE THE FASCICLES EASIER TO VISUALIZE (BRINGING DOWN THE RIGHT END MAKES THE BRIGHTS BRIGHTER AND BRINGING UP THE LEFT END MAKES THE DARKS DARKER), WE CHOOSE A FASCICLE THAT IS EASILY VISIBLE AND A GOOD AVERAGE FASCICLE TO SET AS THE EXAMPLE FOR THE “CYLINDER CORRELATION” PROGRAM TO USE TO FIND OTHER FASCICLES IN THE MUSCLE VOLUME.

8) In the Properties window (Fig. 3):
   a) Adjust Outer Cylinder Radius visually to match the thickness of the fascicle you have chosen
   b) Set Mask Cylinder Radius to 110% of the Outer Cylinder Radius
   c) Record Cylinder Length and Outer Cylinder Radius for future reference (we use the same radius for all muscles in a single animal)
   d) Leave other parameters at default values
   e) Click Apply

*Note: Depending on the size of the muscle and processing speed, correlation computing can take as few as a few minutes or potentially as long as a day. In our analysis, most muscles took about 20 minutes.*

9) Save resulting Correlation Field and Orientation Field

**Project view: Cylinder Correlation (step 9)**

# Fiber tracing

1) Re-create your original label field in the new cropped image

*Note: The purpose of this step is to generate a label field that matches the original dimensions of your muscle prior to “growing,” which will be used to mask artifacts in the next step. Alternatively, you can save your original label field from the beginning of the SOP, but you will need to crop it to the exact size of your cropped image stack.*

a) Select your Result stack (Result_Specimen_MuscleA.am) and open the Segmentation tab
b) Select the entire muscle using the Magic Wand tool (you may need to adjust the Masking threshold)
c) In the Selection window, Shrink Selection 5 times (or whatever number of grows you used originally)
d) Add your selection to the material and save your label field (e.g., Specimen_MuscleA.labels)

2) Mask the Correlation Field to remove artifacts
   a) Select the Correlation Field and add an Arithmetic module
   b) Input A is the Correlation Field, Input B is the label field without grows, expression is the same as before (a*(b>0)
   c) Save the result (e.g., Specimen_muscleA_CorrelationField.am)

**Project view: Fiber Tracing (step 2)**

3) Determine starting values for Minimum Seed Correlation
   a) Select the masked Correlation Field and create an Isosurface module (Display>Isosurface)
   b) In the Properties window, adjust the Threshold value visually (hitting Apply each time you change the threshold to show the result) to show the most fibers and fewest artifacts (Fig. 4, top). Record the threshold value.
4) Determine starting value for Minimum Distance
   a) In the Toolbar, select “3D Length” from the dropdown menu (2D Length is default)
   b) In the viewer showing the Isosurface, click on two adjacent but separate fascicles
   c) Record the length.
5)
Determine starting value for Minimum Continuation Quality

Figure 4. Determining starting values for Minimum Seed Correlation (MSC-top) and Minimum Continuation Quality (MCQ-bottom). Select cropped Correlation Field and create both Isosurface and Ortho Slice modules. Adjust slider in the Properties window of the Isosurface until you see mainly fibers in the viewer – the resulting Threshold value is your starting MSC. Then, Adjust left-hand slider in the Colormap of the Ortho Slice module until the background is black and only fibers are visible – the resulting left-hand slider value is your starting MCQ.

a) Select the masked Correlation Field again and create an Ortho Slice (Display>Ortho Slice)
b) In the Properties window, adjust the left-hand slider in the Colormap until the grey noise diminishes and the only visible bright spots are individual fascicles on a black background (Fig. 4, bottom). Record the value adjacent to the left-hand slider.

6) Trace fibers
   a) Select the masked Correlation Field and create a Trace Correlation Lines module (Fiber Tracing>Trace Correlation Lines)

**Project view: Fiber Tracing (step 6)**

b) In the Properties window:
   - Choose your Orientation Field from the Orientation Field dropdown menu
   - Set Minimum Seed Correlation to the value recorded in step 3 from the Isosurface
   - Set Minimum Continuation Quality to the value recorded in step 5 from the Ortho Slice
   - Keep Direction Coefficient at 0.3
   - Set Minimum Distance to the value you recorded in step 4 from the Isosurface
   - Set Minimum Length at 70% Cylinder Length (from Cylinder Correlation step - this ensures that we do not miss fascicles shorter than the average one we visually chose as an example).
   - Set Search Cone Length to 2 (if your scan is in micrometers, set it to 2000)
   - Set Search Cone Angle to 10
   - Set Minimum Step Size to 2
c) Apply

*Note: this step will take significantly longer than the Cylinder Correlation. In our examples it took 1-3 hours.*

# Reviewing results and making adjustments

1) Select the Correlation Lines file and create a Spatial Graph module
2)
Hit apply (Fig. 5)

Figure 5. Viewing properties of Correlation Lines (left) and visualizing them a Spatial Graph (right).

3) Check that fascicles have been traced throughout the muscle
   a) Select the Label field you used to mask the Correlation Field (Specimen_MuscleA.labels)
   b) Create a Volume Rendering (Display>Volume Rendering)
   c) Adjust Opacity so that you can see both the volume and the lines (about 0.05)
4) Adjust your results by making changes to the Trace Correlation Lines properties and re-run Correlation Lines module (Fig. 4):
   a) To decrease the number of fascicles (“Edges” in the Properties window; Fig. 5), increase Minimum Distance (we usually aim for 30-60 fascicles per muscle)
   b) To filter out noise that can produce biologically unrealistic fascicles, increase Minimum Seed Correlation (alternatively, if you there are areas of the muscle with no fascicles you might decrease this value).
   c) To reduce fascicles with artificial “breaks,” decrease Minimum Continuation Quality (alternatively, if correlation lines appear to be jumping from one fascicle to another, increase this value).
   d) To reduce the number of highly curved fascicles, decrease Direction Coefficient. Some consistent curvature of fascicles throughout the muscle is normal, but sometimes individual fascicles are highly curved in different directions.

Figure 6. Adjusting fiber tracing parameters using visualization tools. Images on left show spatial graphs with original and revised values. Images on right show effects of adjusting individual parameters from their original values.

5) You can also check fascicle orientation for outliers, which will be removed in the next step:
   a) Select Correlation Lines and click “Show” under Table in the Properties window
   b) In the Tables window, click the Segment tab
   c) Scroll to Orientation Theta

# Exporting and filtering data

1) Record the final parameters you used for Trace Correlation Lines for future reference
2) Export lines as a mesh file for visualization
   a) Select Correlation Lines and create an Extract Surface module (Fiber Tracing>Extract Surface from Spatial Graph)
   b) Set tube scale value as desired (we used 0.1-0.2)
   c) Set tube complexity to maximum value
   d) Apply the module
   e) Check appearance by selecting the .surf file and creating a Surface View module (Display>Surface View)

**Project view: Exporting and filtering data (step 2)**

f) Export .surf file as a .ply (File>Export Data As>Stanford PLY)

3) Under Table in the Properties window of the Correlation Lines file, click Export As … (the only choice is an XML file)
4) Filter outliers by orientation
   a) Open the .xml file in Excel
   b) Click on the Segments tab
   c) In the first empty column, enter the formula for z-score: =(E2-(AVERAGE(E:E)))/(STDEV.S(E:E)), where E2 is the Orientation Theta column. Title this column “z-score theta”
   d) Copy the formula to all rows with data
   e) Segments with a z-score of >1.3 or <-1.3 are considered outliers

# Multiscale Cylinder Correlation

*Note: for muscles that are multipennate and vary greatly in their fascicle orientation, the multiscale cylinder correlation plug-in has yielded significantly cleaner results than using one cylinder. We used this for muscles such as temporalis, which fan out with a wide angle on one end and taper at the other end.*

1) Set up a new Amira file for Multiscale Cylinder Correlation
   a) Follow all steps for “Isolating individual muscles” above and steps 1-8 of “Cylinder correlation,” but **do not apply the cylinder correlation**
   b) Download the Multiscale Cylinder Correlation file from the Amira extras library: [https://www.thermofisher.com/software-em-3d-vis/xtra-library/xtras/multi-scale-cylinder-correlation](https://nam10.safelinks.protection.outlook.com/?url=https%3A%2F%2Fwww.thermofisher.com%2Fsoftware-em-3d-vis%2Fxtra-library%2Fxtras%2Fmulti-scale-cylinder-correlation&data=05%7C02%7CJulia.Molnar%40nyit.edu%7C39b11714cc864d70e63408dcca68aac9%7C1a561373a3a14beda7c281410491a214%7C0%7C0%7C638607798111893222%7CUnknown%7CTWFpbGZsb3d8eyJWIjoiMC4wLjAwMDAiLCJQIjoiV2luMzIiLCJBTiI6Ik1haWwiLCJXVCI6Mn0%3D%7C0%7C%7C%7C&sdata=N%2FGPxzFb07Jxr7TwlDhhbSnOqXcDLERMZnQvgCEJTr8%3D&reserved=0)
   c) Extract the files and save to your computer
   d) Import the Multiscale Cylinder Correlation module (File> Open Data (navigate to the folder where you extracted the files)> Xtra_MultiscaleCylinderCorrelation> local> share> python_script_objects> MultiscaleCylinderCorrelation.pyscro
   e) Save the project file
2) Set properties for Multiscale Cylinder Correlation to create 3 cylinders of different sizes
   a) Select the Multiscale Cylinder Correlation module and enter the following settings in the Properties window (Fig. 7):
      - Data: select cropped Result file
      - Number of Cylinder Templates: 3
      - Length: copy the length from the Visual Cylinder module to Length 2 and set Length 1 to 20% longer and Length 3 to 20% shorter. Make sure that the longest cylinder is in Length 1.
      - Do the same for Mask Radius and Outer Radius (-20%, original from Visual Cylinder, +20%)
      - All other settings are left at their defaults
   b) Click Apply (this step will take approximately 3x as long as the regular Visual Cylinder)

3) Proceed with Fiber Tracing as usual, using “maxCorrelation” as the correlation field and “maxOrientation” as the orientation field. When you get to the step that asks for 70% of cylinder length, use Length 2 from MultiscaleCylinderCorrelation as the basis for calculating this value.

N*ote: Multiscale Cylinder works in millimeters. If your data use other units, convert the values from Visual Cylinder to millimeters before entering them into the Properties window.*

Figure 7. Property window for Multiscale Cylinder Correlation showing sample values for three cylinders with a range of lengths and diameters. Units are in millimeters.
